# Supplementary material for: Cobalt-catalyzed cross-electrophile coupling of alkynyl sulfides with unactivated chlorosilanes
Source: Nat Commun. 2024 May 27;15:4502. doi: 10.1038/s41467-024-48873-2 (PMC11130142; doi:10.1038/s41467-024-48873-2)
Supplement: Supplementary file 1 — Supplementary Information [file 41467_2024_48873_MOESM1_ESM.pdf]

Supplementary Information

Cobalt-Catalyzed Cross-Electrophile Coupling of Alkynyl Sulfides with Unactivated Chlorosilanes

Donghui Xing, Jinlin Liu, Dingxin Cai, Bin Huang, Huanfeng Jiang, Liangbin Huang*

State Key Laboratory of Pulp & Paper Engineering, Key Laboratory of Functional Molecular Engineering of Guangdong Province, School of Chemistry and Chemical Engineering, South China University of Technology, Guangzhou 510640, China.

E-mail: huanglb@scut.edu.cn.

Table of Contents

1. General Information 1

2. Optimization of Reaction Conditions 2

2.1 General Procedure for the Optimization Reactions 2

3. Synthesis of Substrates 9

3.1. Synthesis of Alkynyl Sulfides 9

3.2. Synthesis of Chlorosilanes 22

4. Experimental Procedures and Characterization of Products 23

4.1. General Procedure F 23

4.2. Characterization Data of Products 23

4.3. Gram-Scale Experiment 51

4.4. Product Derivatizations 51

4.5. Synthesis of bioactive molecule: 6-fluoroflavone 55

4.6. Extended to synthesis of alkynylgermanes 56

5. Mechanistic studies 59

5.1. Radical trapping experiments 59

5.2. Radical clock experiment 59

5.3. Control experiments 60

5.4. Study on the possibility of an alkynylzinc Intermediate 61

5.5. Cyclic voltammograms of Co^{II}(tpy) and *in-situ* generated Co^{I}(tpy) in DMF 62

5.6. Study on the reactivity of the *in-situ-formed* Co^{I}(tpy) towards alkynyl sulfide 63

6. NMR Spectra 65

7. Reference 195

1. General Information

Reagents and Solvents

Unless otherwise noted, all chemicals used in the preparation of starting materials were commercially available and were used as received without further purifications. Metal catalysts and ligands were purchased from Acros, Laajoo, Energy Chemical, Bidepharm, and Accela. Other chemicals were purchased from Macklin, Adamas, and Innochem, and were directly used without further purifications.

Anhydrous solvents were purchased from Energy Chemical and Innochem. Toluene was distilled from sodium/benzophenone and stored under nitrogen before use.

Analytical Methods

^{1}H, ^{13}C and ^{19}F NMR spectra were recorded using a Bruker DRX-400 spectrometer or a Bruker DRX-500 spectrometer using $CDCl_3$ as solvent. The chemical shifts are referenced to signals at 7.26 and 77.0 ppm, respectively, and chloroform is solvent with TMS as the internal standard. Chemical shifts are reported in parts per million (ppm), multiplicities are indicated by s (singlet), d (doublet), t (triplet), q (quartet), m (multiplet) and br (broad). Coupling constants (J) are reported in Hertz. Melting points were measured using a melting point instrument. Mass spectra were recorded on a Thermo Scientific ISQ gas chromatograph-mass spectrometer. GC analyses were performed on an Agilent 7890B GC equipped with HP-5 columns (30 m × 320 μm × 0.25 μm), FID detectors, and hydrogen as the carrier gas. GC/MS analyses were performed on a Shimadzu GCMS-QP2010SE equipped with an RTX-5MS column (30 m × 0.25 mm × 0.25 μm) with a quadrupole mass analyzer using helium as the carrier gas. TLC was performed by using commercially prepared 100-400 mesh silica gel plates and visualization was effected at 254 nm.

2. Optimization of Reaction Conditions

2.1 General Procedure for the Optimization Reactions

The procedure was conducted in a nitrogen-filled glove box. To a reaction vial equipped with a magnetic stir bar was added the indicated catalyst (10 mol%), ligand (15 mol%), and reductant (0.20 mmol, 2.0 equiv). A solution of substrate **1a** (17.8 mg, 0.10 mmol, 1.0 equiv) and TMSCl (32.6 mg, 0.30 mmol, 3.0 equiv) in 1.0 mL of the indicated solvent was then added by syringe, and the resultung mixture was maintained with stirring at room temperature for 4-6 h. After the indicated reaction time, the mixture was diluted with EtOAc (10 mL) and washed with water. The organic phase was then dried over anhydrous Na_2SO_4, filtered, and concentrated in vacuo. The residue was dissolved with 2 mL EtOAc and subjected to GC analysis with dodecane as the internal standard.

Supplementary Table 1: Screening of Additives

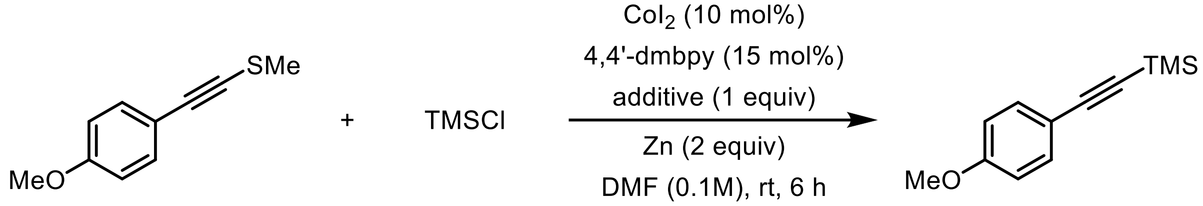

1a, 0.1 mmol   3 equiv   **3a**

Entry	additive	Yield (%)[a,b]
1	--	43
2	NaI	38
3	LiI	36
4	LiBr	40
5	LiCl	41
6	$MgCl_2$	35
7	$MgBr_2$	37
8	$ZnCl_2$	33
9	ZnI_2	31

[a] All reactions were performed on a 0.10 mmol scale. [b] Yield was determined by GC analysis with dodecane as an internal standard.

Supplementary Table 2: Screening of Solvents

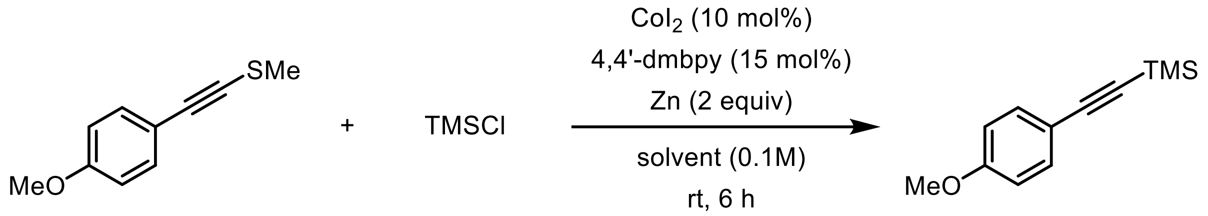

1a, 0.1 mmol | 3 equiv | **3a**

Entry	Solvent	Yield (%)[a,b]
1	DMF	43
2	DMA	31
3	NMP	39
4	DMSO	n.d.
5	MeCN	trace
6	DCE	n.d.
7	THF	trace
8	dioxane	n.d.
9	toluene	n.d.
10	hexane	n.d.

[a] All reactions were performed on a 0.10 mmol scale. [b] Yield was determined by GC analysis with dodecane as an internal standard.

Supplementary Table 3: Screening of Ligands

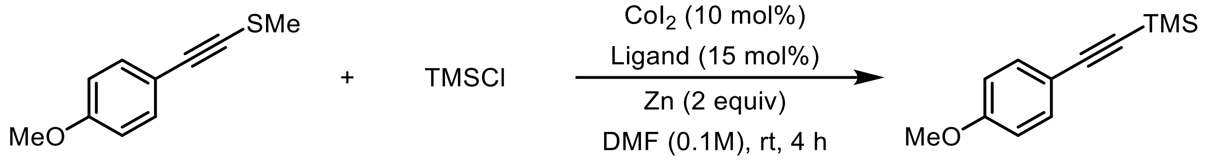

1a, 0.1 mmol   3 equiv   **3a**

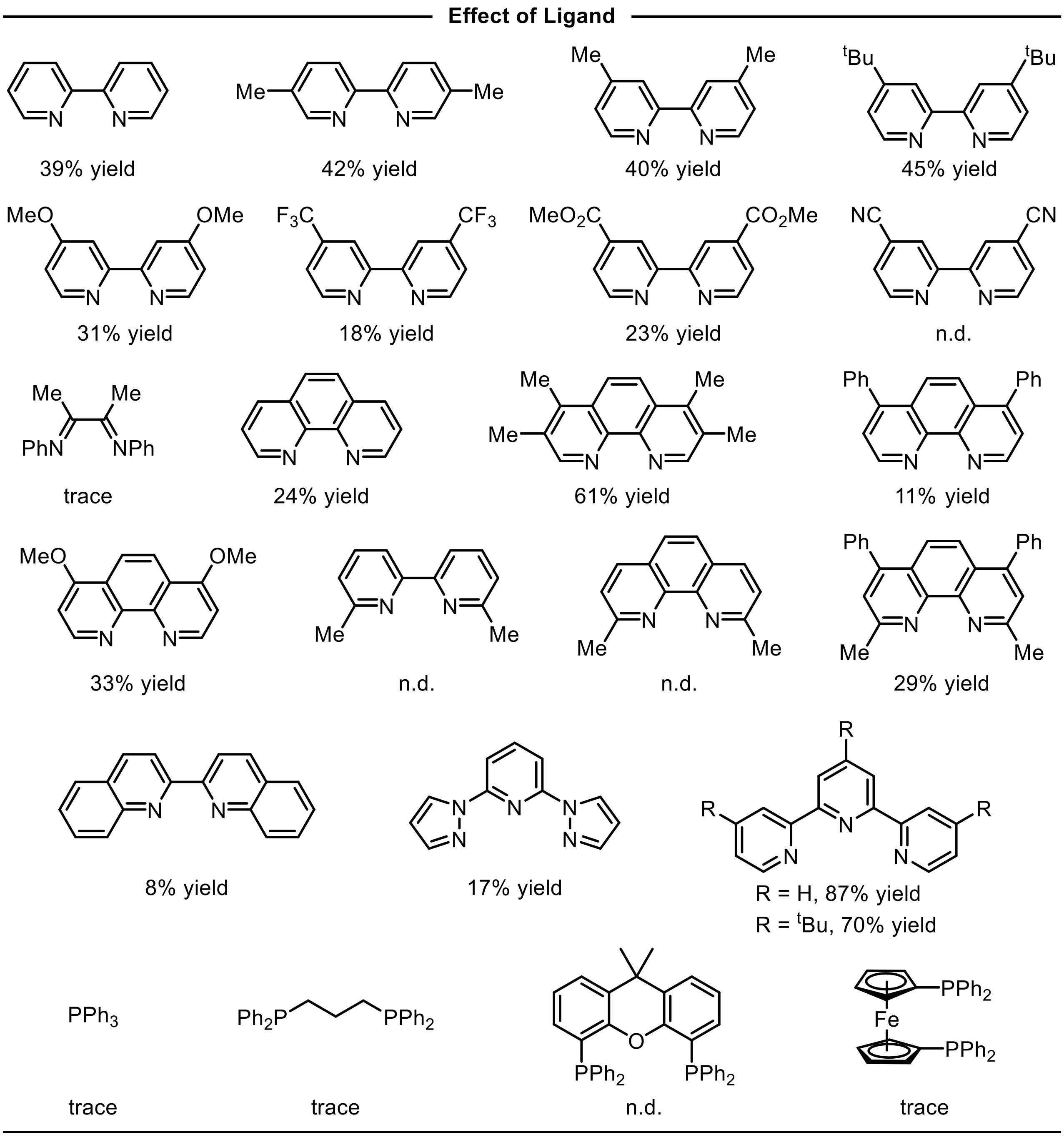

[a] All reactions were performed on a 0.10 mmol scale. [b] Yield was determined by GC analysis with dodecane as an internal standard.

Supplementary Table 4: Screening of Cobalt Catalysts

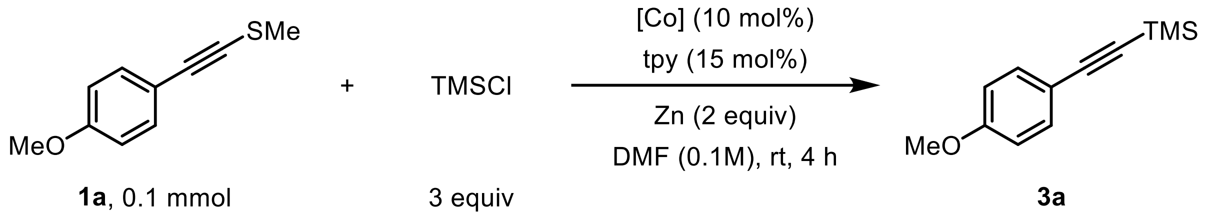

Entry	[Co]	Yield (%)[a,b]
1	CoI_2	87
2	$CoBr_2$	38
3	$CoCl_2$	69
4	$Co(acac)_2$	70
5	$Co(acac)_3$	73
6	$Co(OAc)_2$	72

[a] All reactions were performed on a 0.10 mmol scale. [b] Yield was determined by GC analysis with dodecane as an internal standard.

Supplementary Table 5: Screening of Other Reaction Parameters.

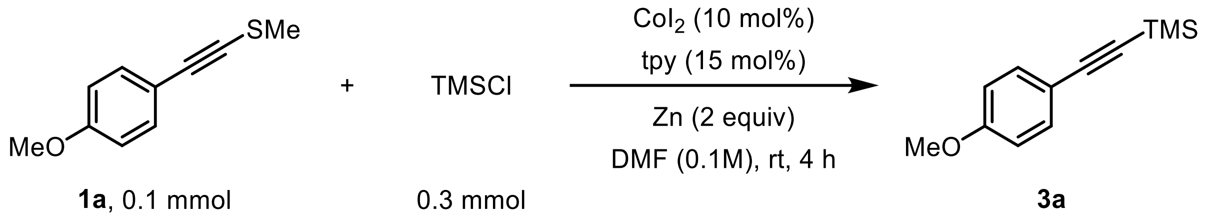

Entry	Variations	Yield (%)[a,b]
**1**	**none**	**87(85)**[c]
2	0 °C instead of rt	n.d.
3	70 °C instead of rt	72
4	Mn instead of Zn	n.d.
5	Mn instead of Zn	67[d]
6	Mn instead of Zn	35[e]
7	w/o Zn	n.d.
8	w/o CoI_2	n.d.
9	w/o tpy	n.d.
10	2 equiv TMSCl	86
11	3 equiv Zn	82
12	DMF(0.2M)	47
13	NiI_2	45
14	$Fe(acac)_2$	n.d.
15	$CrCl_2$	29

[a] All reactions were performed on a 0.10 mmol scale. [b] Yield was determined by GC analysis with dodecane as an internal standard. [c] Isolated yield after flash chromatography on silica gel. [d] Using 1 equiv ZnI_2 as the additive. [e] Using 1 equiv $Zn(OTf)_2$ as the additive; n.d. = no detected; w/o = without.

Supplementary Table 6: Screening of Different SR Group.

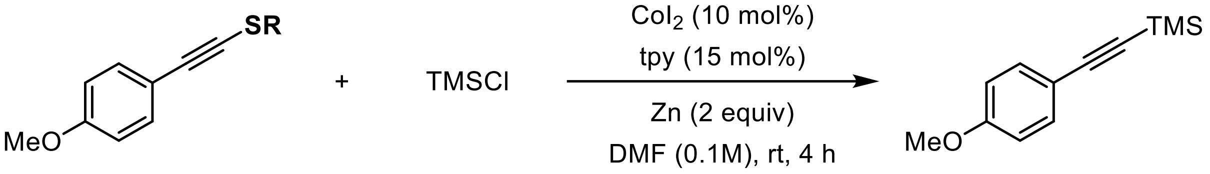

1, 0.1 mmol　　**3a**

Entry	SR	Yield (%)[a,b]
1	SMe	87
2	SPh	81
3	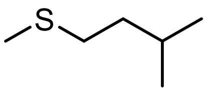	80
4	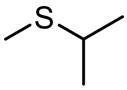	41
5	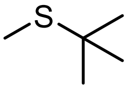	< 5

[a] All reactions were performed on a 0.10 mmol scale. [b]Yield was determined by GC analysis with dodecane as an internal standard.

3. Synthesis of Substrates

3.1. Synthesis of Alkynyl Sulfides

The alkynyl sulfides used in this paper:

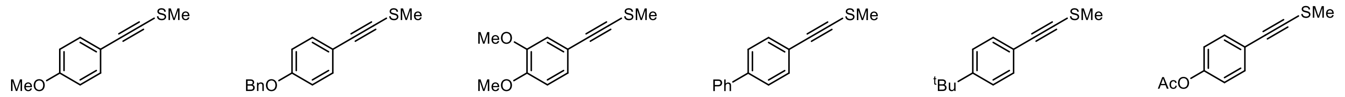

1a **1b** **1c** **1d** **1e** **1f**

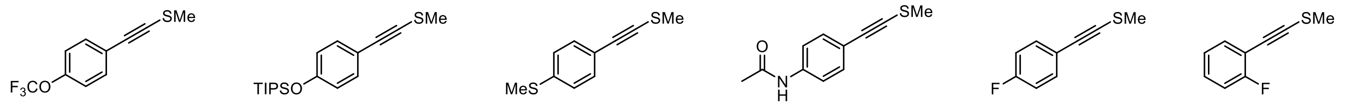

1g **1h** **1i** **1j** **1k** **1l**

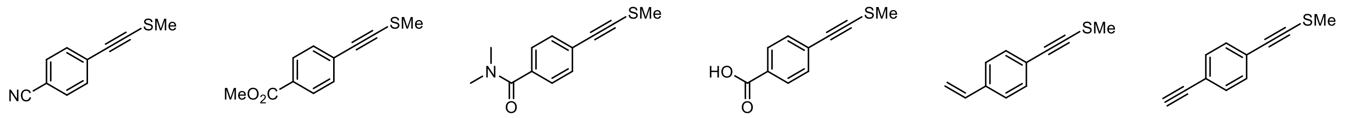

1m **1n** **1o** **1p** **1q** **1r**

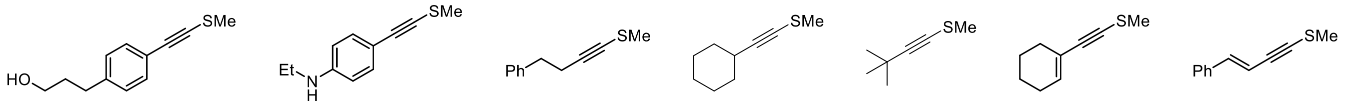

1s **1t** **1u** **1v** **1w** **1x** **1y**

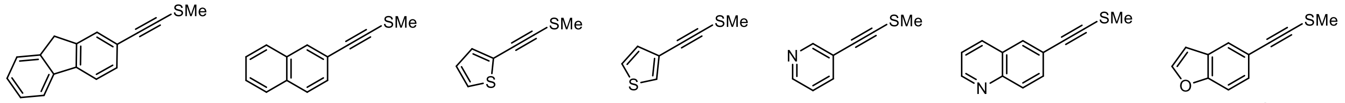

1z **1aa** **1ab** **1ac** **1ad** **1ae** **1af**

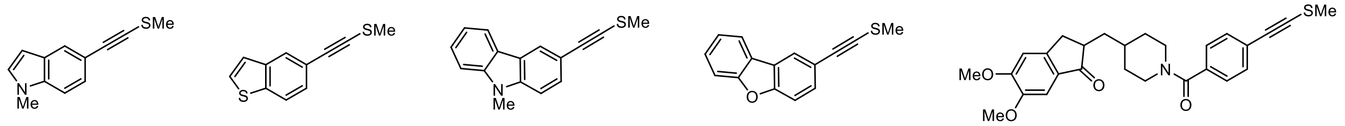

1ag **1ah** **1ai** **1aj** **1ak**

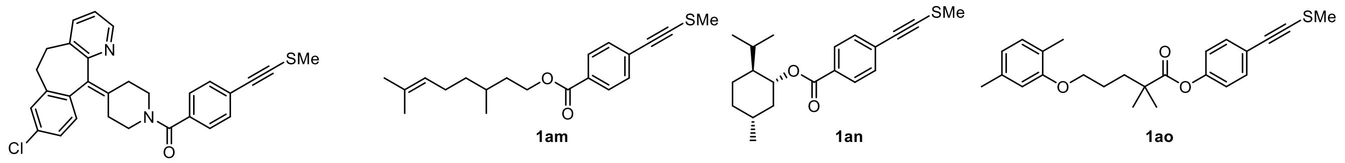

1al **1am** **1an** **1ao**

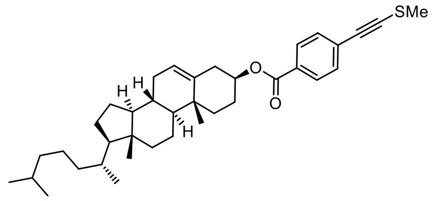

1ap

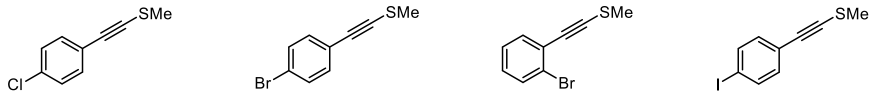

1aq **1ar** **1as** **1at**

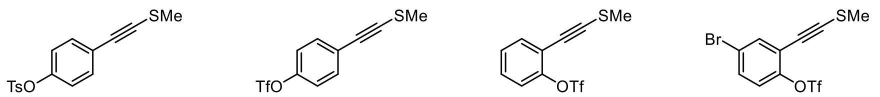

1au **1av** **1aw** **1ax**

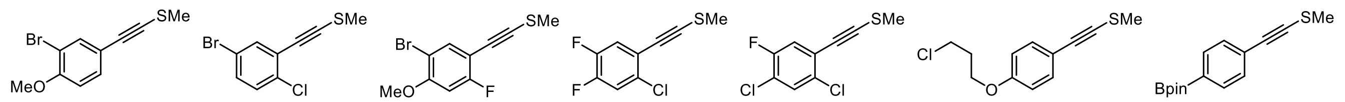

1ay **1az** **1ba** **1bb** **1bc** **1bd** **1be**

All the substrates above were synthesized according to the literature reports.[1] All spectroscopic data of known molecules match those previously reported in the literature. Some new alkynyl sulfides were synthesized with the following procedures.

General Procedure A (Most of the substrates were synthesized by this method)

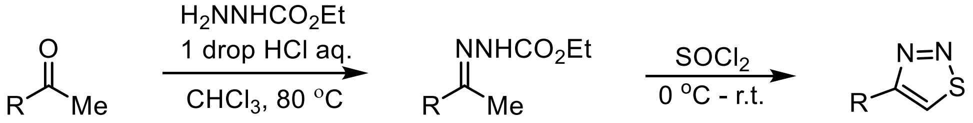

A mixture of acetophenone (1 equiv) and ethyl hydrazine carboxylate (1.2 equiv) was dissolved in dry, hot chloroform. When the reaction mixture started refluxing, one drop of concentrated hydrochloric acid were added and the mixture was then refluxed overnight with continuous removal of the water generated. The solvent was removed under vacuum and the residue was washed several times with diethyl ether or chloroform to remove excess reactants. Next, An excess amount of thionyl chloride was stirred at 0 °C and the hydrazones were added in several portions. The mixtures were stirred at room temperature overnight until no more hydrogen chloride was produced. The remaining thionyl chloride was evaporated under vacuum and the residue was washed with diethyl ether to give good yields of the corresponding 1,2,3-thiadiazoles as fine powders. A recrystallization from chloroform or dimethylsulfoxide was carried out when necessary.

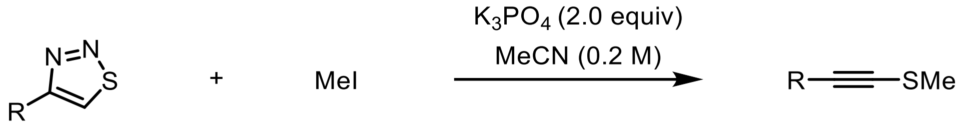

According to our previous literature procedure[1b], the respective 1,2,3-thiadiazole (1.0 equiv), K_3PO_4 (2.0 equiv) and MeI (2.0 equiv) were suspended in MeCN at room temperature and stirred for 24 h at 70 °C. The mixture was quenched by 1N-HCl. Then the reaction mixture was diluted with ethyl acetate, washed with water, the organic phases were dried over anhydrous Na_2SO_4, filtered and concentrated in vacuum. The residue was purified by flash column chromatography on silica gel with petroleum ether/ethyl acetate as the eluent to afford the corresponding products.

General Procedure B

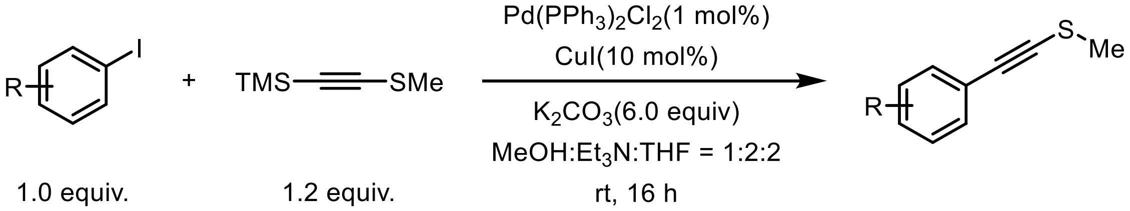

According to a literature procedure[1c], the respective (hetero)aryl iodide (1.0 equiv), $Pd(PPh_3)Cl_2$ (1 mol%), CuI (10 mol%) and K_2CO_3 (6.0 equiv) were added to a Schlenk flask equipped with a PTFE-coated stir bar under argon. 1-methylthio-2-(trimethylsilyl)-ethyne (1.2 equiv) and solvent $MeOH/Et_3N/THF$ (0.25 M, 1:2:2 v:v:v) were added and the reaction mixture was stirred for 16 h at rt. Upon completion, the reaction mixture was filtered, concentrated in vacuo and the crude material was purified by column chromatography on silica gel to afford the desired products.

General Procedure C

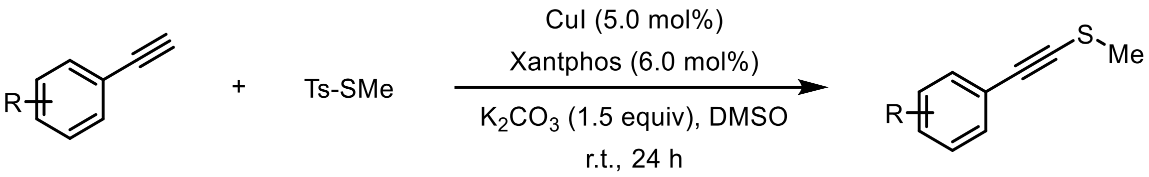

According to a literature procedure[1e], a mixture of arylacetylene (1.0 equiv), $TolSO_2SMe$ (1.5 equiv), CuI (5.0 mol %), Xantphos (6.0 mol %), and K_2CO_3 (1.5 equiv) was suspended in DMSO at room temperature and stirred for 24 h at the same temperature. After filtration of the mixture through a pad of Celite, the filtrate was concentrated under reduced pressure. To the residue was added ethyl acetate and the mixture was washed with aqueous saturated solution of sodium bicarbonate and brine, and then dried (Na_2SO_4). After filtration, the filtrate was concentrated under reduced pressure. The crude residue was purified by column chromatography using silica gel with petrol ether/ethyl acetate as the eluent to afford the corresponding products.

General Procedure D

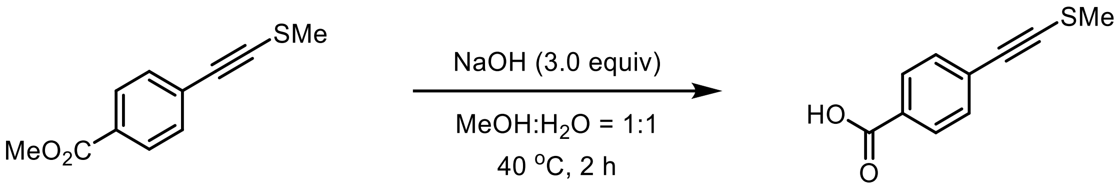

According to a literature procedure[2], a suspension of methyl 4-((methylthio)ethynyl)benzoate (1.0 equiv) in a 1:1 mixture of MeOH/H_2O (40 mL) was treated with NaOH (3.0 equiv). The mixture was stirred at 40 ^oC for 2 h. The mixture was then diluted with H_2O and the MeOH was mostly removed in vacuo. The H_2O solution was washed with ether, acidified to pH 1 with 2 M HCl solution and extracted with EtOAc. The combined EtOAc extracts were washed with brine, dried over $MgSO_4$, and concentrated in vacuo to give corresponding carboxylic acid as a white solid.

General Procedure E

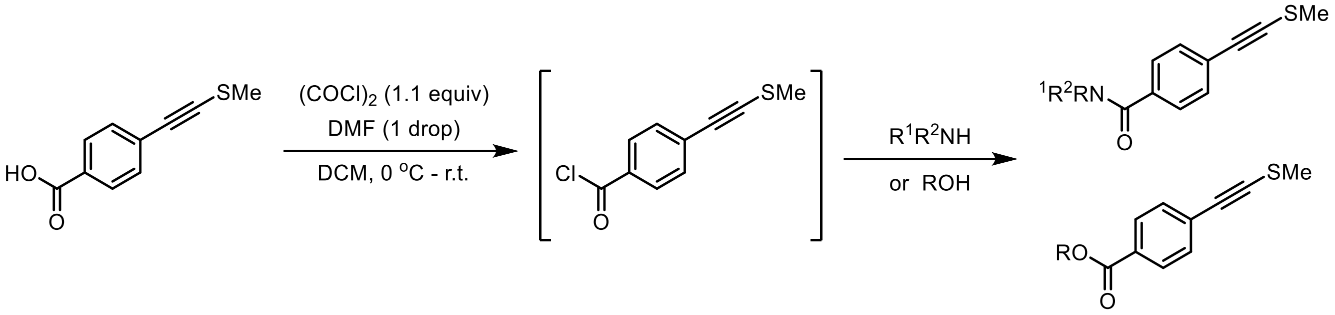

According to a literature procedure[3], to a 50 mL heat-gun dried round bottom flask were added 10 mL dry methylene chloride (DCM), 4-((methylthio)ethynyl)benzoic acid (1.0 equiv). The round bottom flask with the DCM and 4-((methylthio)ethynyl)benzoic acid were posed into an ice bath. Then oxalyl chloride (1.1 equiv) and two drops DMF were added into the flask. The gas formation was observed. The mixture remained stirring for another 1 hour at room temperature. Then desired coupling amine or alcohol (1.1 equiv), NEt_3 (1.1 equiv) in 3 mL DCM were added into the previous mixture dropwise at 0 ^oC. After the addition, the mixture were kept stirring for another 8 hours at room temperature. The mixture was

concentrated in vacuo, the residue was directly purified by flash column chromatography on silica gel to give the desired product (amide or ester).

Characterization of New Alkynyl Sulfides

((4-(benzyloxy)phenyl)ethynyl)(methyl)sulfane (1b)

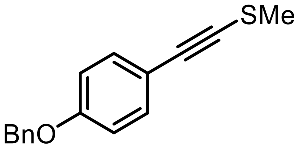

It's synthesized according to general **procedure A**. White solid, 73% yield (flash column chromatography eluent, petrol ether/ ethyl acetate = 100/1).

^{1}H NMR (400 MHz, Chloroform-*d*) δ 7.44 – 7.31 (m, 7H), 6.93 – 6.87 (m, 2H), 5.06 (s, 2H), 2.46 (s, 3H).

^{13}C NMR (101 MHz, Chloroform-*d*) δ 158.8, 136.5, 133.4, 128.6, 128.1, 127.4, 115.7, 114.8, 91.6, 79.1, 70.0, 19.5.

Melting point: 63-64 °C

HRMS-ESI (m/z): $[M+H]^+$ Calcd for $C_{16}H_{15}OS$ 255.0838; Found: 255.0833.

4-((methylthio)ethynyl)phenyl acetate (1f)

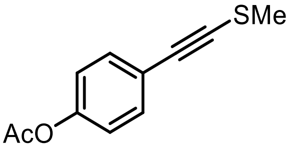

It's synthesized according to general **procedure A**. Yellow oil, 63% yield (flash column chromatography eluent, petrol ether/ ethyl acetate = 50/1).

^{1}H NMR (500 MHz, Chloroform-*d*) δ 7.33 (d, *J* = 8.6 Hz, 2H), 6.95 (d, *J* = 8.6 Hz, 2H), 2.37 (s, 3H), 2.19 (s, 3H).

^{13}C NMR (126 MHz, Chloroform-*d*) δ 169.0, 150.2, 132.5, 121.5, 120.9, 90.9, 81.0, 21.0, 19.2.

HRMS-ESI (m/z): $[M+H]^+$ Calcd for $C_{11}H_{11}O_2S$ 207.0474; Found: 207.0471.

methyl((4-(trifluoromethoxy)phenyl)ethynyl)sulfane (1g)

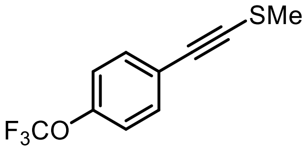

It's synthesized according to general **procedure A**. Yellow oil, 88% yield (flash column chromatography eluent, petrol ether/ ethyl acetate = 100/1).

^{1}H NMR (500 MHz, Chloroform-*d*) δ 7.42 (d, *J* = 8.8 Hz, 2H), 7.13 (d, *J* = 8.1 Hz, 2H), 2.47 (s, 3H).

^{13}C NMR (126 MHz, Chloroform-*d*) δ 148.7, 132.9, 122.2, 120.8, 120.4 (q, *J* = 257.7 Hz), 90.5, 82.2, 19.3.

^{19}F NMR (471 MHz, Chloroform-*d*) δ -57.85.

HRMS-ESI (m/z): $[M+H]^+$ Calcd for $C_{10}H_8F_3OS$ 233.0242; Found: 233.0239.

N,N-dimethyl-4-((methylthio)ethynyl)benzamide (1o)

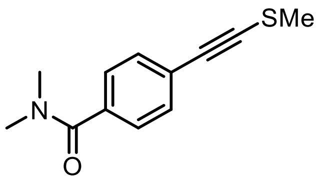

It's synthesized according to general **procedure E**. White solid, 51% yield (flash column chromatography eluent, petrol ether/ ethyl acetate = 5/1).

^{1}H NMR (500 MHz, Chloroform-*d*) δ 7.41 – 7.34 (m, 2H), 7.33 – 7.27 (m, 2H), 3.04 (s, 3H), 2.91 (s, 3H), 2.44 (s, 3H).

^{13}C NMR (126 MHz, Chloroform-*d*) δ 170.7, 135.3, 130.9, 127.0, 124.6, 91.1, 82.7, 39.3, 35.2, 19.2.

Melting point: 69-70 °C

HRMS-ESI (m/z): $[M+H]^+$ Calcd for $C_{12}H_{14}NOS$ 220.0791; Found: 220.0788.

4-((methylthio)ethynyl)benzoic acid (1p)

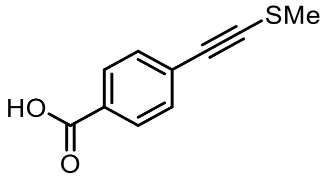

It's synthesized according to general **procedure D**. White solid, 96% yield.

^{1}H NMR (500 MHz, DMSO-d_6) δ 13.07 (s, 1H), 7.93 (d, *J* = 7.4 Hz, 2H), 7.52 (d, *J* = 7.5 Hz, 2H), 2.55 (s, 3H).

^{13}C NMR (126 MHz, DMSO-d_6) δ 166.8, 130.9, 130.1, 129.6, 127.0, 91.1, 85.4, 19.0.

Melting point: 209-210 °C

HRMS-ESI (m/z): $[M-H]^+$ Calcd for $C_{10}H_7O_2S$ 191.0172; Found: 191.0164.

((4-ethynylphenyl)ethynyl)(methyl)sulfane (1r)

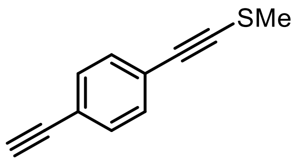

It's synthesized according to general **procedure B**. Yellow oil, 78% yield (flash column chromatography eluent, petrol ether/ ethyl acetate = 100/1).

^{1}H NMR (500 MHz, Chloroform-*d*) δ 7.41 (d, *J* = 8.4 Hz, 2H), 7.34 (d, *J* = 8.5 Hz, 2H), 3.15 (s, 1H), 2.48 (s, 3H).

^{13}C NMR (126 MHz, Chloroform-*d*) δ 132.0, 131.1, 123.9, 121.5, 91.4, 83.5, 83.2, 78.8, 19.3.

HRMS-ESI (m/z): $[M+H]^+$ Calcd for $C_{11}H_9S$ 173.0419; Found: 173.0418.

6-((methylthio)ethynyl)quinoline (1ae)

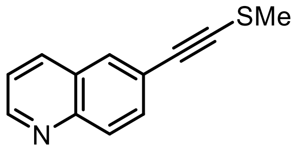

It's synthesized according to general **procedure B**. Brown solid, 68% yield (flash column chromatography eluent, petrol ether/ ethyl acetate = 100/1).

^{1}H NMR (400 MHz, Chloroform-*d*) δ 8.89 – 8.80 (m, 1H), 8.07 – 7.93 (m, 2H), 7.87 – 7.78 (m, 1H), 7.68 – 7.59 (m, 1H), 7.40 – 7.28 (m, 1H), 2.48 (s, 3H).

^{13}C NMR (101 MHz, Chloroform-*d*) δ 150.7, 147.3, 135.5, 131.8, 130.5, 129.4, 127.8, 121.6, 91.5, 82.7, 19.3.

Melting point: 38-39 °C

HRMS-ESI (m/z): $[M+H]^+$ Calcd for $C_{12}H_{10}NS$ 200.0528; Found: 200.0527.

5-((methylthio)ethynyl)benzo[b]thiophene (1ah)

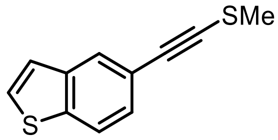

It's synthesized according to general **procedure A**. Yellow oil, 83% yield (flash column chromatography eluent, petrol ether/ ethyl acetate = 100/1).

^{1}H NMR (500 MHz, Chloroform-*d*) δ 7.91 – 7.87 (m, 1H), 7.79 (d, *J* = 8.3 Hz, 1H), 7.46 (d, *J* = 5.4 Hz, 1H), 7.38 (dd, *J* = 8.4, 1.6 Hz, 1H), 7.29 (d, *J* = 5.4 Hz, 1H), 2.50 (s, 3H).

^{13}C NMR (126 MHz, Chloroform-*d*) δ 139.46, 139.45, 127.32, 127.26, 126.9, 123.6, 122.3, 119.2, 92.0, 80.2, 19.4.

HRMS-ESI (m/z): $[M+H]^+$ Calcd for $C_{11}H_9S_2$ 205.0140; Found: 205.0139.

9-methyl-2-((methylthio)ethynyl)-9H-carbazole (1ai)

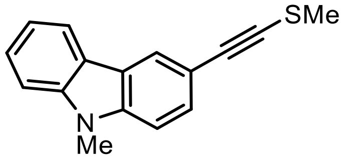

It's synthesized according to general **procedure B**. Yellow oil, 60% yield (flash column chromatography eluent, petrol ether/ ethyl acetate = 100/1).

^{1}H NMR (500 MHz, Chloroform-*d*) δ 8.07 (d, *J* = 1.1 Hz, 1H), 7.91 (d, *J* = 7.7 Hz, 1H), 7.42 (dd, *J* = 8.4, 1.6 Hz, 1H), 7.38 – 7.33 (m, 1H), 7.21 (d, *J* = 8.2 Hz, 1H), 7.14 – 7.11 (m, 2H), 3.61 (s, 3H), 2.39 (s, 3H).

^{13}C NMR (126 MHz, Chloroform-*d*) δ 141.2, 140.5, 129.6, 126.1, 124.4, 122.5, 122.2, 120.3, 119.3, 113.2, 108.6, 108.3, 93.0, 78.1, 29.0, 19.6.

HRMS-ESI (m/z): $[M+H]^+$ Calcd for $C_{16}H_{14}NS$ 252.0841; Found: 252.0837.

2-((methylthio)ethynyl)dibenzo[b,d]furan (1aj)

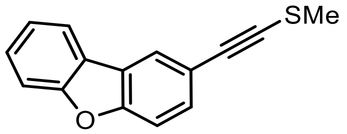

It's synthesized according to general **procedure A**. White solid, 51% yield (flash column chromatography eluent, petrol ether/ ethyl acetate = 100/1).

^{1}H NMR (400 MHz, Chloroform-*d*) δ 8.08 – 8.01 (m, 1H), 7.91 (d, *J* = 7.6 Hz, 1H), 7.57 – 7.44 (m, 4H), 7.35 (t, *J* = 7.8 Hz, 1H), 2.52 (s, 3H).

^{13}C NMR (101 MHz, Chloroform-*d*) δ 156.5, 155.7, 130.9, 127.6, 124.4, 124.2, 123.5, 123.0, 120.7, 117.9, 111.7, 111.7, 91.8, 79.7, 19.4.

Melting point: 63-64 °C

HRMS-ESI (m/z): $[M+H]^+$ Calcd for $C_{15}H_{11}OS$ 239.0525; Found: 239.0521.

5,6-dimethoxy-2-((1-(4-((methylthio)ethynyl)benzoyl)piperidin-4-yl)methyl)-2,3-dihydro-1H-inden-1-one (1ak)

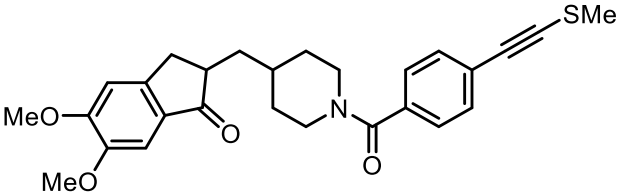

It's synthesized according to general **procedure E**. White solid, 74% yield (flash column chromatography eluent, petrol ether/ ethyl acetate = 3/1).

^{1}H NMR (500 MHz, Chloroform-*d*) δ 7.43 (d, *J* = 8.3 Hz, 2H), 7.34 (d, *J* = 8.3 Hz, 2H), 7.17 (s, 1H), 6.87 (s, 1H), 4.70 (s, 1H), 3.94 (d, *J* = 28.9 Hz, 6H), 3.84 – 3.64 (m, 1H), 3.33 – 3.23 (m, 1H), 3.02 (s, 1H), 2.86 – 2.66 (m, 3H), 2.49 (s, 3H), 2.01 – 1.58 (m, 5H), 1.46 – 1.29 (m, 2H).

^{13}C NMR (126 MHz, Chloroform-*d*) δ 207.3, 169.5, 155.5, 149.4, 148.5, 135.4, 131.1, 129.0, 126.9, 124.6, 107.2, 104.2, 91.2, 82.7, 56.2, 56.0, 47.9, 44.9, 42.4, 38.5, 34.5, 33.3, 32.3, 31.3, 19.3.

Melting point: 133-134 °C

HRMS-ESI (m/z): $[M+H]^+$ Calcd for $C_{27}H_{30}NO_4S$ 490.2408; Found: 490.2403.

(4-(8-chloro-5,6-dihydro-11H-benzo[5,6]cyclohepta[1,2-b]pyridin-11-ylidene)piperidin-1-yl)(4-((methylthio)ethynyl)phenyl)methanone (1al)

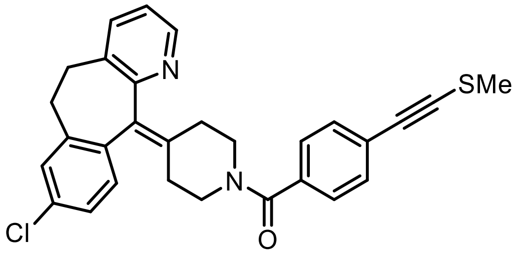

It's synthesized according to general **procedure E**. White solid, 83% yield (flash column chromatography eluent, petrol ether/ ethyl acetate = 1/1).

^{1}H NMR (500 MHz, Chloroform-*d*) δ 8.28 (s, 1H), 7.46 – 7.24 (m, 5H), 7.13 – 6.95 (m, 4H), 4.20 – 3.95 (m, 1H), 3.55 (s, 1H), 3.36 – 3.10 (m, 4H), 2.78 – 2.68 (m, 2H), 2.54 – 2.17 (m, 7H).

^{13}C NMR (126 MHz, Chloroform-*d*) δ 169.7, 156.4, 146.4, 139.4, 137.6, 136.0, 134.8, 134.4, 133.2, 131.9, 131.4, 131.0, 130.2, 128.8, 126.7, 126.0, 124.7, 122.2, 91.1, 82.9, 48.3, 43.1, 31.4, 31.3, 30.2, 19.1, 17.0.

Melting point: 91-92 °C

HRMS-ESI (m/z): $[M+H]^+$ Calcd for $C_{29}H_{26}ClN_2OS$ 511.1967; Found: 511.1964.

3,7-dimethyloct-6-en-1-yl 4-((methylthio)ethynyl)benzoate compound with 4-((methylthio)ethynyl)phenyl 5-(2,5-dimethylphenoxy)-2,2-dimethylpentanoate (1:1) (1ao)

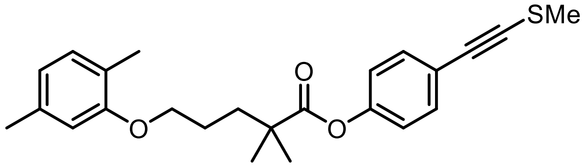

It's synthesized according to general **procedure B**. Yellow oil, 46% yield (flash column chromatography eluent, petrol ether/ ethyl acetate = 20/1).

^{1}H NMR (500 MHz, Chloroform-*d*) δ 7.41 (d, *J* = 6.8 Hz, 2H), 7.00 (d, *J* = 7.4 Hz, 1H), 6.97 (d, *J* = 6.9 Hz, 2H), 6.67 (d, *J* = 7.3 Hz, 1H), 6.62 (s, 1H), 3.98 (s, 2H), 2.48 (d, *J* = 1.8 Hz, 3H), 2.31 (s, 3H), 2.17 (s, 3H), 1.87 (s, 4H), 1.36 (s, 6H).

^{13}C NMR (126 MHz, Chloroform-*d*) δ 176.1, 156.8, 150.7, 136.5, 132.6, 130.3, 123.6, 121.6, 120.8, 120.7, 111.9, 91.0, 80.9, 67.7, 42.5, 37.1, 25.2, 25.1, 21.4, 19.3, 15.8.

HRMS-ESI (m/z): $[M+H]^+$ Calcd for $C_{24}H_{29}O_3S$ 357.2244; Found: 357.2240.

(3S,8S,9S,10R,13R,14S,17R)-10,13-dimethyl-17-((R)-6-methylheptan-2-yl)-2,3,4,7,8,9,10,11,12,13,14,15,16,17-tetradecahydro-1H-cyclopenta[a]phenanthren-3-yl 4-((methylthio)ethynyl)benzoate (1ap)

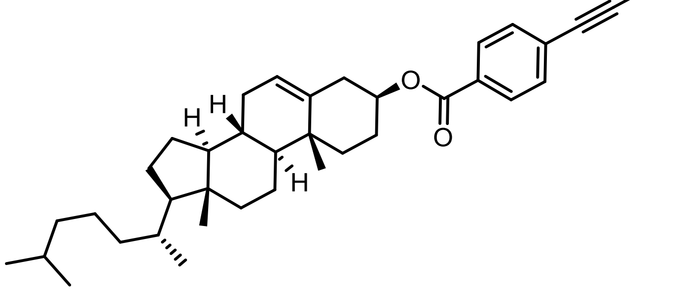

It's synthesized according to general **procedure E**. White solid, 50% yield (flash column chromatography eluent, petrol ether/ ethyl acetate = 20/1).

^{1}H NMR (500 MHz, Chloroform-*d*) δ 7.95 (d, *J* = 8.3 Hz, 2H), 7.42 (d, *J* = 8.2 Hz, 2H), 5.45 – 5.37 (m, 1H), 4.89 – 4.79 (m, 1H), 2.49 (s, 3H), 2.45 (d, *J* = 7.7 Hz, 2H), 2.04 – 1.95 (m, 3H), 1.90 (d, *J* = 13.4 Hz,

1H), 1.86 – 1.79 (m, 1H), 1.78 – 1.62 (m, 2H), 1.59 – 1.44 (m, 6H), 1.35 (dd, *J* = 15.3, 8.3 Hz, 3H), 1.24 – 1.08 (m, 7H), 1.06 (s, 3H), 1.03 – 0.96 (m, 3H), 0.92 (d, *J* = 6.5 Hz, 3H), 0.87 (dd, *J* = 6.6, 2.1 Hz, 6H), 0.68 (s, 3H).

^{13}C NMR (126 MHz, Chloroform-*d*) δ 165.4, 139.5, 130.7, 129.7, 129.4, 127.8, 122.8, 91.6, 84.8, 74.7, 56.6, 56.1, 50.0, 42.3, 39.9, 39.5, 38.2, 37.0, 36.6, 36.2, 35.8, 31.89, 31.82, 28.2, 28.0, 27.8, 24.3, 23.8, 22.8, 22.5, 21.0, 19.3, 18.7, 11.8.

Melting point: 132-133 °C

HRMS-ESI (m/z): $[M+H]^+$ Calcd for $C_{37}H_{53}O_2S$ 587.4279; Found: 587.4275.

((2-bromophenyl)ethynyl)(methyl)sulfane (1as)

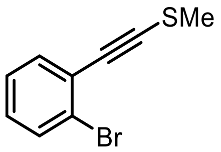

It's synthesized according to general **procedure A**. Yellow oil, 91% yield (flash column chromatography eluent, petrol ether/ ethyl acetate = 100/1).

^{1}H NMR (500 MHz, Chloroform-*d*) δ 7.59 – 7.53 (m, 1H), 7.41 (dd, *J* = 7.7, 1.5 Hz, 1H), 7.25 – 7.20 (m, 1H), 7.15 – 7.09 (m, 1H), 2.52 (s, 3H).

^{13}C NMR (126 MHz, Chloroform-*d*) δ 132.7, 132.3, 128.9, 126.9, 125.5, 124.9, 90.6, 86.5, 19.4.

HRMS-ESI (m/z): $[M+H]^+$ Calcd for C_9H_8BrS 226.9525; Found: 226.9521.

4-((methylthio)ethynyl)phenyl 4-methylbenzenesulfonate (1au)

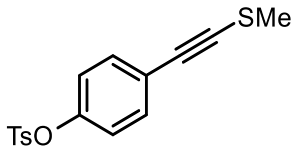

It's synthesized according to general **procedure A**. Yellow oil, 81% yield (flash column chromatography eluent, petrol ether/ ethyl acetate = 50/1).

^{1}H NMR (400 MHz, Chloroform-*d*) δ 7.65 (d, *J* = 8.3 Hz, 2H), 7.30 – 7.25 (m, 4H), 6.92 – 6.87 (m, 2H), 2.41 (s, 3H), 2.39 (s, 3H).

^{13}C NMR (101 MHz, Chloroform-*d*) δ 148.7, 145.4, 132.4, 131.7, 129.6, 128.2, 122.21, 122.19, 90.3, 82.4, 21.4, 19.0.

HRMS-ESI (m/z): $[M+H]^+$ Calcd for $C_{16}H_{15}O_3S_2$ 319.0457; Found: 319.0455.

4-((methylthio)ethynyl)phenyl trifluoromethanesulfonate (1av)

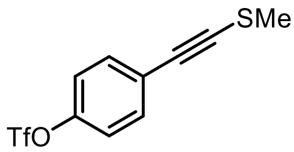

It’s synthesized according to general **procedure A**. Yellow oil, 70% yield (flash column chromatography eluent, petrol ether/ ethyl acetate = 100/1).

^{1}H NMR (400 MHz, Chloroform-*d*) δ 7.52 – 7.45 (m, 2H), 7.23 (d, *J* = 8.8 Hz, 2H), 2.51 (s, 3H).

^{13}C NMR (101 MHz, Chloroform-*d*) δ 148.6, 133.0, 124.1, 121.4, 120.3, 90.0, 83.7, 19.2.

^{19}F NMR (376 MHz, Chloroform-*d*) δ -72.79.

HRMS-ESI (m/z): $[M+H]^+$ Calcd for $C_{10}H_8F_3O_3S_2$ 296.9861; Found: 296.9857.

2-((methylthio)ethynyl)phenyl trifluoromethanesulfonate (1aw)

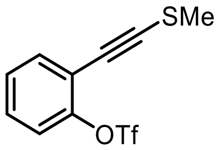

It’s synthesized according to general **procedure A**. Yellow oil, 67% yield (flash column chromatography eluent, petrol ether/ ethyl acetate = 100/1).

^{1}H NMR (500 MHz, Chloroform-*d*) δ 7.51 (dd, *J* = 7.5, 1.9 Hz, 1H), 7.39 – 7.31 (m, 2H), 7.29 – 7.26 (m, 1H), 2.54 (s, 3H).

^{13}C NMR (126 MHz, Chloroform-*d*) δ 149.5, 133.1, 129.2, 128.1, 121.5, 118.7 (q, *J* = 320.5 Hz), 118.5, 89.6, 85.1, 18.9.

^{19}F NMR (471 MHz, Chloroform-*d*) δ -73.67.

HRMS-ESI (m/z): $[M+H]^+$ Calcd for $C_{10}H_8F_3O_3S_2$ 296.9861; Found: 296.9860.

4-bromo-2-((methylthio)ethynyl)phenyl trifluoromethanesulfonate (1ax)

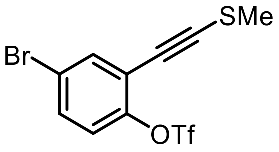

It’s synthesized according to general **procedure A**. Yellow oil, 45% yield (flash column chromatography eluent, petrol ether/ ethyl acetate = 100/1).

^{1}H NMR (500 MHz, Chloroform-*d*) δ 7.61 (d, *J* = 2.4 Hz, 1H), 7.44 (dd, *J* = 8.8, 2.4 Hz, 1H), 7.12 (d, *J* = 8.8 Hz, 1H), 2.52 (s, 3H).

^{13}C NMR (126 MHz, Chloroform-*d*) δ 148.3, 135.5, 132.0, 122.9, 121.4, 120.4, 118.6 (q, *J* = 320.6 Hz), 91.7, 84.1, 18.9.

^{19}F NMR (376 MHz, Chloroform-*d*) δ -73.49.

HRMS-ESI (m/z): $[M+H]^+$ Calcd for $C_{10}H_7BrF_3O_3S_2$ 374.8967; Found: 374.8961.

((3-bromo-4-methoxyphenyl)ethynyl)(methyl)sulfane (1ay)

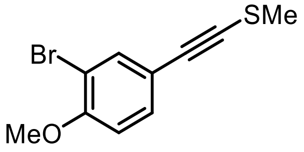

It's synthesized according to general **procedure A**. Yellow oil, 75% yield (flash column chromatography eluent, petrol ether/ ethyl acetate = 100/1).

^{1}H NMR (500 MHz, Chloroform-*d*) δ 7.60 (d, *J* = 1.9 Hz, 1H), 7.32 (dd, *J* = 8.5, 1.9 Hz, 1H), 6.78 (d, *J* = 8.5 Hz, 1H), 3.87 (s, 3H), 2.44 (s, 3H).

^{13}C NMR (126 MHz, Chloroform-*d*) δ 155.9, 136.3, 132.1, 116.9, 111.4, 111.2, 90.1, 80.5, 56.2, 19.3.

HRMS-ESI (m/z): $[M+H]^+$ Calcd for $C_{10}H_{10}BrOS$ 256.9630; Found: 256.9626.

((5-bromo-2-chlorophenyl)ethynyl)(methyl)sulfane (1az)

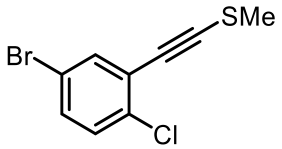

It's synthesized according to general **procedure A**. Yellow oil, 66% yield (flash column chromatography eluent, petrol ether/ ethyl acetate = 100/1).

^{1}H NMR (400 MHz, Chloroform-*d*) δ 7.54 (d, *J* = 2.3 Hz, 1H), 7.31 (dd, *J* = 8.6, 2.3 Hz, 1H), 7.22 (d, *J* = 8.6 Hz, 1H), 2.51 (s, 3H).

^{13}C NMR (101 MHz, Chloroform-*d*) δ 134.9, 134.2, 131.6, 130.4, 125.1, 119.7, 89.0, 87.7, 19.4.

HRMS-ESI (m/z): $[M+H]^+$ Calcd for C_9H_7BrClS 260.9135; Found: 260.9131.

((5-bromo-2-fluoro-4-methoxyphenyl)ethynyl)(methyl)sulfane (1ba)

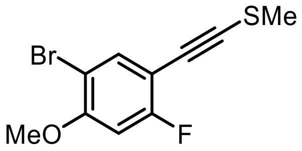

It's synthesized according to general **procedure A**. White solid, 98% yield (flash column chromatography eluent, petrol ether/ ethyl acetate = 100/1).

^{1}H NMR (500 MHz, Chloroform-*d*) δ 7.54 (d, *J* = 7.6 Hz, 1H), 6.60 (d, *J* = 10.8 Hz, 1H), 3.86 (s, 3H), 2.45 (s, 3H).

^{13}C NMR (126 MHz, Chloroform-*d*) δ 162.9 (d, *J* = 252.0 Hz), 156.8 (d, *J* = 9.7 Hz), 136.6 (d, *J* = 2.5 Hz), 105.6 (d, *J* = 3.6 Hz), 105.1 (d, *J* = 17.1 Hz), 100.0 (d, *J* = 26.6 Hz), 85.9 (d, *J* = 3.2 Hz), 83.5, 56.5, 19.3.

^{19}F NMR (471 MHz, Chloroform-*d*) δ -107.58.

Melting point: 39-40 °C

HRMS-ESI (m/z): $[M+H]^+$ Calcd for $C_{10}H_9BrFOS$ 274.9536; Found: 274.9532.

((2-chloro-4,5-difluorophenyl)ethynyl)(methyl)sulfane (1bb)

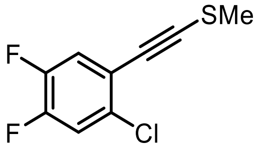

It's synthesized according to general **procedure A**. Yellow oil, 67% yield (flash column chromatography eluent, petrol ether/ ethyl acetate = 100/1).

^{1}H NMR (400 MHz, Chloroform-*d*) δ 7.25 – 7.18 (m, 2H), 2.51 (s, 3H).

^{13}C NMR (126 MHz, Chloroform-*d*) δ 150.1 (dd, *J* = 125.0, 13.5 Hz), 148.1 (dd, *J* = 119.8, 13.5 Hz), 130.9 (dd, *J* = 7.9, 3.5 Hz), 120.6 (d, *J* = 19.4 Hz), 119.9 (dd, *J* = 7.8, 4.1 Hz), 118.4 (d, *J* = 20.7 Hz), 88.2 (d, *J* = 2.1 Hz), 86.9 (t, *J* = 2.2 Hz), 19.2.

^{19}F NMR (376 MHz, Chloroform-*d*) δ -133.31 – -133.44 (m), -138.68 – -138.80 (m).

HRMS-ESI (m/z): $[M+H]^+$ Calcd for $C_9H_6ClF_2S$ 218.9841; Found: 218.9839.

((2,4-dichloro-5-fluorophenyl)ethynyl)(methyl)sulfane (1bc)

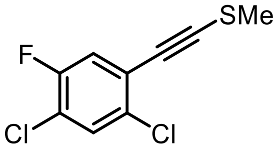

It's synthesized according to general **procedure A**. Yellow oil, 71% yield (flash column chromatography eluent, petrol ether/ ethyl acetate = 100/1).

^{1}H NMR (400 MHz, Chloroform-*d*) δ 7.42 (d, *J* = 6.8 Hz, 1H), 7.18 (d, *J* = 9.1 Hz, 1H), 2.52 (s, 3H).

^{13}C NMR (101 MHz, Chloroform-*d*) δ 156.2 (d, *J* = 249.5 Hz), 130.8 (d, *J* = 3.5 Hz), 130.6, 123.1 (d, *J* = 8.9 Hz), 121.2 (d, *J* = 19.3 Hz), 119.6 (d, *J* = 23.8 Hz), 89.7, 87.3 (d, *J* = 2.5 Hz), 19.3.

^{19}F NMR (376 MHz, Chloroform-*d*) δ -117.47.

HRMS-ESI (m/z): $[M+H]^+$ Calcd for $C_9H_6Cl_2FS$ 234.9546; Found: 234.9542.

4,4,5,5-tetramethyl-2-(4-((methylthio)ethynyl)phenyl)-1,3,2-dioxaborolane (1be)

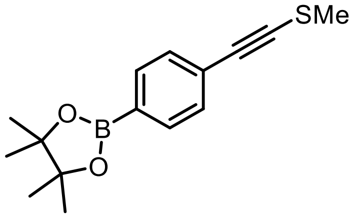

It's synthesized according to general **procedure A**. Yellow solid, 44% yield (flash column chromatography eluent, petrol ether/ ethyl acetate = 10/1).

^{1}H NMR (500 MHz, Chloroform-*d*) δ 7.72 (d, *J* = 8.0 Hz, 2H), 7.39 (d, *J* = 8.0 Hz, 2H), 2.48 (s, 3H), 1.34 (s, 12H).

^{13}C NMR (126 MHz, Chloroform-*d*) δ 134.5, 130.3, 126.0, 92.1, 83.9, 82.6, 24.8, 19.4.

Melting point: 77-78 °C

HRMS-ESI (m/z): $[M+H]^+$ Calcd for $C_{15}H_{19}BO_2S$ 274.1308; Found: 274.1301.

3.2. Synthesis of Chlorosilanes

The chlorosilanes used in this paper:

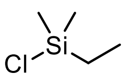

2a

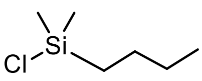

2b

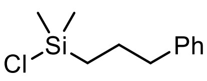

2c

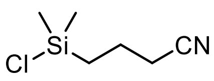

2d

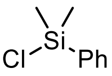

2e

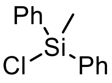

2f

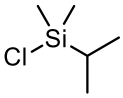

2g

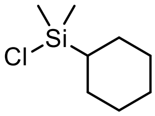

2h

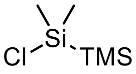

2i

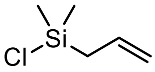

2j

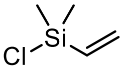

2k

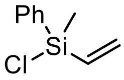

2l

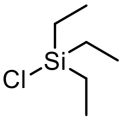

2m

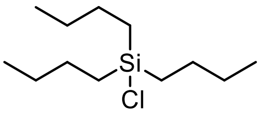

2n

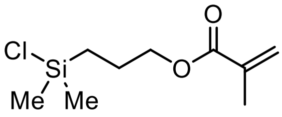

2o

All the substrates above were purchased from Macklin, Energy Chemical and Innochem and used directly.

4. Experimental Procedures and Characterization of Products

4.1. General Procedure F

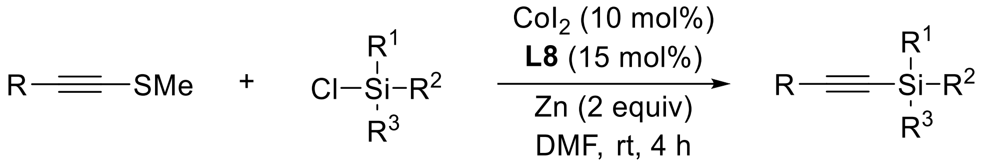

1, 0.2 mmol **2**, 2.0 equiv **3**

The procedure was conducted in a nitrogen-filled glove box. To a reaction vial equipped with a magnetic stir bar was added CoI_2 (6.3 mg, 0.02 mmol), **L8** (7.0 mg, 0.03 mmol), Zn (26.2 mg, 2 equiv). A solution of **1** (0.2 mmol) and chlorosilane **2** (0.4 mmol) in DMF (2.0 mL) was added. The reaction vial was sealed and removed from the glove box. The mixture was stirred at room temperature for 4 h, subsequently quenched with water (10.0 mL) and extracted with ethyl acetate (3 × 15.0 mL). The combined organic layers were washed with water, brine, dried over anhydrous Na_2SO_4, and concentrated under reduced pressure. The residue was purified by flash chromatography on silica gel to afford product.

4.2. Characterization Data of Products

((4-methoxyphenyl)ethynyl)trimethylsilane (3a)

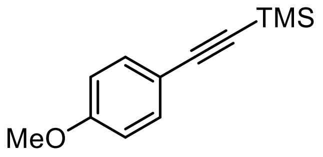

Prepared according to general procedure F using ((4-methoxyphenyl)ethynyl)(methyl)sulfane (35.6 mg, 0.2 mmol), TMSCl (43.5 mg, 0.4 mmol), CoI_2 (6.3 mg, 0.02 mmol), **L8** (7.0 mg, 0.03 mmol), Zn (26.2 mg, 0.4 mmol), DMF (2.0 mL) at room temperature for 4 h. The crude material was purified by flash chromatography on silica gel to provide the title compound **3a** as a colorless oil (35.0 mg, 85% yield)**.** Characterization data matched those reported in the literature.[4]

^{1}H NMR (500 MHz, Chloroform-*d*) δ 7.41 (d, *J* = 8.4 Hz, 2H), 6.82 (d, *J* = 8.4 Hz, 2H), 3.80 (s, 3H), 0.25 (s, 9H).

^{13}C NMR (126 MHz, Chloroform-*d*) δ 159.7, 133.4, 115.2, 113.8, 105.2, 92.4, 55.2, 0.0.

((4-(benzyloxy)phenyl)ethynyl)trimethylsilane (3b)

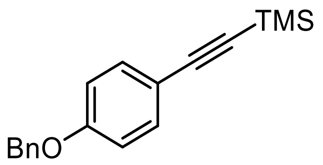

Prepared according to general procedure F using ((4-(benzyloxy)phenyl)ethynyl)(methyl)sulfane (50.8 mg, 0.2 mmol), TMSCl (43.5 mg, 0.4 mmol), CoI_2 (6.3 mg, 0.02 mmol), **L8** (7.0 mg, 0.03 mmol), Zn (26.2 mg, 0.4 mmol), DMF (2.0 mL) at room temperature for 4 h. The crude material was purified by flash chromatography on silica gel to provide the title compound **3b** as a yellow oil (48.0 mg, 86% yield)**.**

^{1}H NMR (400 MHz, Chloroform-*d*) δ 7.47 – 7.30 (m, 7H), 6.93 – 6.87 (m, 2H), 5.07 (s, 2H), 0.26 (s, 9H).

^{13}C NMR (126 MHz, Chloroform-*d*) δ 158.9, 136.5, 133.5, 128.6, 128.1, 127.5, 115.5, 114.7, 105.1, 92.5, 69.9, 0.0.
HRMS-ESI (m/z): $[M-H]^+$ Calcd for $C_{18}H_{19}OSi$ 279.1211; Found: 279.1210.

((3,4-dimethoxyphenyl)ethynyl)trimethylsilane (3c)

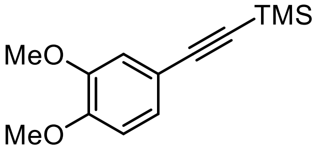

Prepared according to general procedure F using ((3,4-dimethoxyphenyl)ethynyl)(methyl)sulfane (41.6 mg, 0.2 mmol), TMSCl (43.5 mg, 0.4 mmol), CoI_2 (6.3 mg, 0.02 mmol), **L8** (7.0 mg, 0.03 mmol), Zn (26.2 mg, 0.4 mmol), DMF (2.0 mL) at room temperature for 4 h. The crude material was purified by flash chromatography on silica gel to provide the title compound **3c** as a yellow oil (43.0 mg, 93% yield). Characterization data matched those reported in the literature.[5]
^{1}H NMR (400 MHz, Chloroform-*d*) δ 7.08 – 7.04 (m, 1H), 6.95 (d, *J* = 1.8 Hz, 1H), 6.75 (d, *J* = 8.3 Hz, 1H), 3.85 (s, 6H), 0.23 (s, 9H).
^{13}C NMR (101 MHz, Chloroform-*d*) δ 149.6, 148.4, 125.3, 115.2, 114.5, 110.7, 105.2, 92.3, 55.78, 55.76, 0.0.

([1,1'-biphenyl]-4-ylethynyl)trimethylsilane (3d)

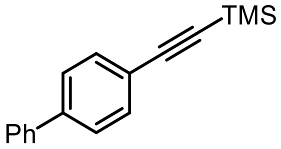

Prepared according to general procedure F using ([1,1'-biphenyl]-4-ylethynyl)(methyl)sulfane (44.8 mg, 0.2 mmol), TMSCl (43.5 mg, 0.4 mmol), CoI_2 (6.3 mg, 0.02 mmol), **L8** (7.0 mg, 0.03 mmol), Zn (26.2 mg, 0.4 mmol), DMF (2.0 mL) at room temperature for 4 h. The crude material was purified by flash chromatography on silica gel to provide the title compound **3d** as a white solid (45.0 mg, 90% yield). Characterization data matched those reported in the literature.[6]
^{1}H NMR (500 MHz, Chloroform-*d*) δ 7.44 – 7.41 (m, 2H), 7.38 (s, 4H), 7.28 (t, *J* = 7.6 Hz, 2H), 7.19 (t, *J* = 7.4 Hz, 1H), 0.12 (s, 9H).
^{13}C NMR (126 MHz, Chloroform-*d*) δ 141.2, 140.3, 132.4, 128.8, 127.6, 127.0, 126.8, 122.0, 105.0, 94.8, 0.0.

((4-(tert-butyl)phenyl)ethynyl)trimethylsilane (3e)

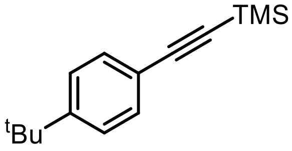

Prepared according to general procedure F using ((4-(tert-butyl)phenyl)ethynyl)(methyl)sulfane (40.8 mg, 0.2 mmol), TMSCl (43.5 mg, 0.4 mmol), CoI_2 (6.3 mg, 0.02 mmol), **L8** (7.0 mg, 0.03 mmol), Zn (26.2 mg, 0.4 mmol), DMF (2.0 mL) at room temperature for 4 h. The crude material was purified by flash chromatography on silica gel to provide the title compound **3e** as a yellow oil (41.0 mg, 89% yield). Characterization data matched those reported in the literature.[7]

^{1}H NMR (500 MHz, Chloroform-*d*) δ 7.41 (d, *J* = 8.4 Hz, 2H), 7.32 (d, *J* = 8.3 Hz, 2H), 1.31 (s, 9H), 0.25 (s, 9H).

^{13}C NMR (126 MHz, Chloroform-*d*) δ 151.7, 131.7, 125.2, 120.1, 105.4, 93.3, 34.8, 31.1, 0.0.

4-((trimethylsilyl)ethynyl)phenyl acetate (3f)

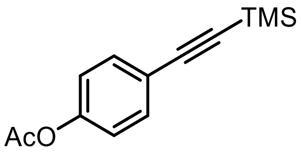

Prepared according to general procedure F using 4-((methylthio)ethynyl)phenyl acetate (41.2 mg, 0.2 mmol), TMSCl (43.5 mg, 0.4 mmol), CoI_2 (6.3 mg, 0.02 mmol), **L8** (7.0 mg, 0.03 mmol), Zn (26.2 mg, 0.4 mmol), DMF (2.0 mL) at room temperature for 4 h. The crude material was purified by flash chromatography on silica gel to provide the title compound **3f** as a colorless solid (30.0 mg, 65% yield). Characterization data matched those reported in the literature.[8]

^{1}H NMR (500 MHz, Chloroform-*d*) δ 7.47 (d, *J* = 8.7 Hz, 2H), 7.03 (d, *J* = 8.7 Hz, 2H), 2.29 (s, 3H), 0.24 (s, 9H).

^{13}C NMR (126 MHz, Chloroform-*d*) δ 169.0, 150.6, 133.1, 121.5, 120.8, 104.2, 94.3, 21.1, -0.1.

trimethyl((4-(trifluoromethoxy)phenyl)ethynyl)silane (3g)

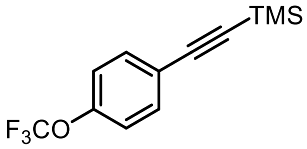

Prepared according to general procedure F using methyl((4-(trifluoromethoxy)phenyl)ethynyl)sulfane (46.4 mg, 0.2 mmol), TMSCl (43.5 mg, 0.4 mmol), CoI_2 (6.3 mg, 0.02 mmol), **L8** (7.0 mg, 0.03 mmol), Zn (26.2 mg, 0.4 mmol), DMF (2.0 mL) at room temperature for 4 h. The crude material was purified by flash chromatography on silica gel to provide the title compound **3g** as a colorless oil (41.0 mg, 79% yield). Characterization data matched those reported in the literature.[7]

^{1}H NMR (500 MHz, Chloroform-*d*) δ 7.50 – 7.46 (m, 2H), 7.14 (d, *J* = 8.1 Hz, 2H), 0.25 (s, 9H).

^{13}C NMR (126 MHz, Chloroform-*d*) δ 149.0, 133.5, 122.0, 120.7, 120.4 (q, *J* = 257.8 Hz), 103.5, 95.3, -0.2.

^{19}F NMR (471 MHz, Chloroform-*d*) δ -57.84.

triisopropyl(4-((trimethylsilyl)ethynyl)phenoxy)silane (3h)

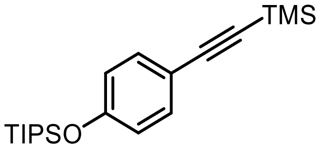

Prepared according to general procedure F using triisopropyl(4-((methylthio)ethynyl)phenoxy)silane (64.0 mg, 0.2 mmol), TMSCl (43.5 mg, 0.4 mmol), CoI_2 (6.3 mg, 0.02 mmol), **L8** (7.0 mg, 0.03 mmol), Zn (26.2 mg, 0.4 mmol), DMF (2.0 mL) at room temperature for 4 h. The crude material was purified by flash chromatography on silica gel to provide the title compound **3h** as a yellow oil (60.0 mg, 87% yield). Characterization data matched those reported in the literature.[9]

^{1}H NMR (500 MHz, Chloroform-*d*) δ 7.34 (d, *J* = 8.6 Hz, 2H), 6.80 (d, *J* = 8.6 Hz, 2H), 1.27 – 1.21 (m, 3H), 1.09 (d, *J* = 7.4 Hz, 18H), 0.24 (s, 9H).

^{13}C NMR (126 MHz, Chloroform-*d*) δ 156.6, 133.4, 119.9, 115.6, 105.3, 92.5, 17.9, 12.6, 0.1.

trimethyl((4-(methylthio)phenyl)ethynyl)silane (3i)

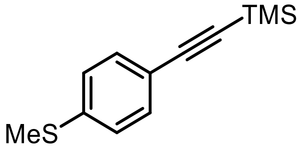

Prepared according to general procedure F using methyl(4-((methylthio)ethynyl)phenyl)sulfane (38.8 mg, 0.2 mmol), TMSCl (43.5 mg, 0.4 mmol), CoI_2 (6.3 mg, 0.02 mmol), **L8** (7.0 mg, 0.03 mmol), Zn (26.2 mg, 0.4 mmol), DMF (2.0 mL) at room temperature for 4 h. The crude material was purified by flash chromatography on silica gel to provide the title compound **3i** as a yellow oil (40.0 mg, 91% yield). Characterization data matched those reported in the literature.[10]

^{1}H NMR (500 MHz, Chloroform-*d*) δ 7.15 (d, *J* = 8.5 Hz, 2H), 6.93 (d, *J* = 8.4 Hz, 2H), 2.25 (s, 3H), 0.03 (s, 9H).

^{13}C NMR (126 MHz, Chloroform-*d*) δ 139.6, 132.2, 125.7, 119.4, 104.9, 94.1, 15.3, 0.0.

N-(4-((trimethylsilyl)ethynyl)phenyl)acetamide (3j)

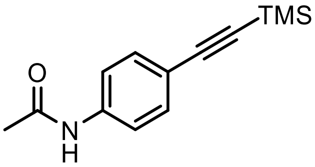

Prepared according to general procedure F using N-(4-((methylthio)ethynyl)phenyl)acetamide (41.0 mg, 0.2 mmol), TMSCl (43.5 mg, 0.4 mmol), CoI_2 (6.3 mg, 0.02 mmol), **L8** (7.0 mg, 0.03 mmol), Zn (26.2 mg, 0.4 mmol), DMF (2.0 mL) at room temperature for 4 h. The crude material was purified by flash chromatography on silica gel to provide the title compound **3j** as a white solid (19.0 mg, 41% yield). Characterization data matched those reported in the literature.[11]

^{1}H NMR (500 MHz, Chloroform-*d*) δ 7.30 (d, *J* = 8.5 Hz, 2H), 7.25 (d, *J* = 8.5 Hz, 2H), 2.01 (s, 3H), 1.51 (s, 1H), 0.08 (s, 9H).

^{13}C NMR (126 MHz, Chloroform-*d*) δ 168.3, 138.1, 132.8, 119.1, 118.8, 104.8, 93.7, 24.7, 0.0.

((4-fluorophenyl)ethynyl)dimethyl(phenyl)silane (3k)

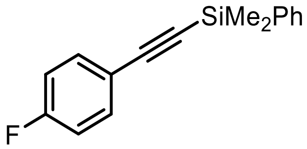

Prepared according to general procedure F using ((4-fluorophenyl)ethynyl)(methyl)sulfane (33.2 mg, 0.2 mmol), $PhMe_2SiCl$ (68.0 mg, 0.4 mmol), CoI_2 (6.3 mg, 0.02 mmol), **L8** (7.0 mg, 0.03 mmol), Zn (26.2 mg, 0.4 mmol), DMF (2.0 mL) at room temperature for 4 h. The crude material was purified by flash chromatography on silica gel to provide the title compound **3k** as a colorless oil (47.0 mg, 93% yield). Characterization data matched those reported in the literature.[12]

^{1}H NMR (400 MHz, Chloroform-*d*) δ 7.76 – 7.69 (m, 2H), 7.51 (ddd, *J* = 8.1, 5.2, 2.5 Hz, 2H), 7.43 (dd, *J* = 4.3, 2.2 Hz, 3H), 7.07 – 6.98 (m, 2H), 0.53 (s, 6H).

^{13}C NMR (126 MHz, Chloroform-*d*) δ 162.7 (d, *J* = 250.1 Hz), 136.9, 134.0 (d, *J* = 8.5 Hz), 133.7, 129.5, 127.9, 119.1 (d, *J* = 3.5 Hz), 115.5 (d, *J* = 22.1 Hz), 105.6, 91.8, -0.9.

^{19}F NMR (471 MHz, Chloroform-*d*) δ -109.99.

((2-fluorophenyl)ethynyl)dimethyl(phenyl)silane (3l)

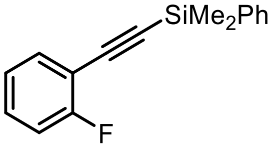

Prepared according to general procedure F using ((2-fluorophenyl)ethynyl)(methyl)sulfane (33.2 mg, 0.2 mmol), $PhMe_2SiCl$ (68.0 mg, 0.4 mmol), CoI_2 (6.3 mg, 0.02 mmol), **L8** (7.0 mg, 0.03 mmol), Zn (26.2 mg, 0.4 mmol), DMF (2.0 mL) at room temperature for 4 h. The crude material was purified by flash chromatography on silica gel to provide the title compound **3l** as a colorless oil (35.0 mg, 69% yield). Characterization data matched those reported in the literature.[13]

^{1}H NMR (500 MHz, Chloroform-*d*) δ 7.76 – 7.69 (m, 2H), 7.53 – 7.48 (m, 1H), 7.44 – 7.40 (m, 3H), 7.35 – 7.29 (m, 1H), 7.11 – 7.06 (m, 2H), 0.53 (s, 6H).

^{13}C NMR (126 MHz, Chloroform-*d*) δ 163.1 (d, *J* = 252.4 Hz), 136.7, 133.9, 133.8, 130.4 (d, *J* = 8.0 Hz), 129.5, 127.9, 123.8 (d, *J* = 3.8 Hz), 115.5 (d, *J* = 20.9 Hz), 111.7 (d, *J* = 15.8 Hz), 99.6, 98.0 (d, *J* = 3.3 Hz), -0.9.

^{19}F NMR (471 MHz, Chloroform-*d*) δ -109.23.

4-((trimethylsilyl)ethynyl)benzonitrile (3m)

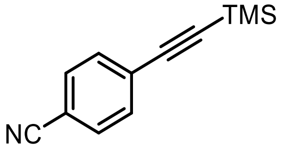

Prepared according to general procedure F using 4-((methylthio)ethynyl)benzonitrile (34.6 mg, 0.2 mmol), TMSCl (43.5 mg, 0.4 mmol), CoI_2 (6.3 mg, 0.02 mmol), **L8** (7.0 mg, 0.03 mmol), Zn (26.2 mg, 0.4 mmol), DMF (2.0 mL) at room temperature for 4 h. The crude material was purified by flash chromatography on silica gel to provide the title compound **3m** as a white solid (22.0 mg, 55% yield). Characterization data matched those reported in the literature.[4]

^{1}H NMR (500 MHz, Chloroform-*d*) δ 7.58 (d, *J* = 8.5 Hz, 2H), 7.53 (d, *J* = 8.5 Hz, 2H), 0.26 (s, 9H).

^{13}C NMR (126 MHz, Chloroform-*d*) δ 132.4, 131.9, 128.0, 118.4, 111.8, 103.0, 99.6, -0.3.

methyl 4-((trimethylsilyl)ethynyl)benzoate (3n)

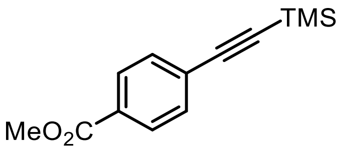

Prepared according to general procedure F using methyl 4-((methylthio)ethynyl)benzoate (41.2 mg, 0.2 mmol), TMSCl (43.5 mg, 0.4 mmol), CoI_2 (6.3 mg, 0.02 mmol), **L8** (7.0 mg, 0.03 mmol), Zn (26.2 mg, 0.4 mmol), DMF (2.0 mL) at room temperature for 4 h. The crude material was purified by flash chromatography on silica gel to provide the title compound **3n** as a yellow oil (26.0 mg, 57% yield). Characterization data matched those reported in the literature.[14]

^{1}H NMR (500 MHz, Chloroform-*d*) δ 7.96 (d, *J* = 8.2 Hz, 2H), 7.51 (d, *J* = 8.2 Hz, 2H), 3.91 (s, 3H), 0.26 (s, 9H).

^{13}C NMR (126 MHz, Chloroform-*d*) δ 166.5, 131.8, 129.7, 129.3, 127.7, 104.0, 97.7, 52.2, -0.2.

N,N-dimethyl-4-((trimethylsilyl)ethynyl)benzamide (3o)

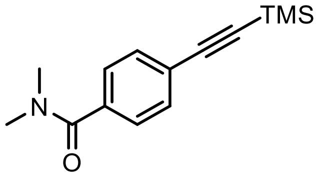

Prepared according to general procedure F using N,N-dimethyl-4-((methylthio)ethynyl)benzamide (43.8 mg, 0.2 mmol), TMSCl (43.5 mg, 0.4 mmol), CoI_2 (6.3 mg, 0.02 mmol), **L8** (7.0 mg, 0.03 mmol), Zn (26.2 mg, 0.4 mmol), DMF (2.0 mL) at room temperature for 4 h. The crude material was purified by flash chromatography on silica gel to provide the title compound **3o** as a yellow oil (41.0 mg, 84% yield). Characterization data matched those reported in the literature.[4]

^{1}H NMR (500 MHz, Chloroform-*d*) δ 7.45 (d, *J* = 8.3 Hz, 2H), 7.32 (d, *J* = 8.3 Hz, 2H), 3.03 (s, 3H), 2.93 (s, 3H), 0.22 (s, 9H).

^{13}C NMR (126 MHz, Chloroform-*d*) δ 170.8, 136.0, 131.8, 126.9, 124.4, 104.1, 95.7, 39.4, 35.3, -0.2.

4-((trimethylsilyl)ethynyl)benzoic acid (3p)

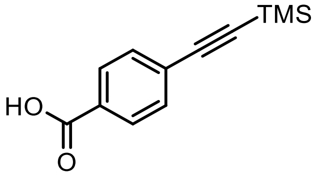

Prepared according to general procedure F using 4-((methylthio)ethynyl)benzoic acid (38.4 mg, 0.2 mmol), TMSCl (43.5 mg, 0.4 mmol), CoI_2 (6.3 mg, 0.02 mmol), **L8** (7.0 mg, 0.03 mmol), Zn (26.2 mg, 0.4 mmol), DMF (2.0 mL) at room temperature for 4 h. The crude material was purified by flash chromatography on silica gel to provide the title compound **3p** as a white solid (31.0 mg, 71% yield). Characterization data matched those reported in the literature.[15]

^{1}H NMR (500 MHz, Chloroform-*d*) δ 8.97 (s, 1H), 8.05 (d, *J* = 8.3 Hz, 2H), 7.55 (d, *J* = 8.3 Hz, 2H), 0.27 (s, 9H).

^{13}C NMR (126 MHz, Chloroform-*d*) δ 171.6, 132.0, 130.0, 128.8, 128.7, 103.9, 98.3, -0.2.

dimethyl(phenyl)((4-vinylphenyl)ethynyl)silane (3q)

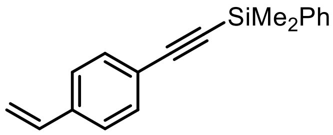

Prepared according to general procedure F using methyl((4-vinylphenyl)ethynyl)sulfane (34.8 mg, 0.2 mmol), $PhMe_2SiCl$ (68.0 mg, 0.4 mmol), CoI_2 (6.3 mg, 0.02 mmol), **L8** (7.0 mg, 0.03 mmol), Zn (26.2 mg, 0.4 mmol), DMF (2.0 mL) at room temperature for 4 h. The crude material was purified by flash chromatography on silica gel to provide the title compound **3q** as a yellow oil (39.0 mg, 75% yield)**.**

^{1}H NMR (500 MHz, Chloroform-*d*) δ 7.68 – 7.59 (m, 2H), 7.39 (d, *J* = 8.2 Hz, 2H), 7.35 – 7.30 (m, 3H), 7.27 (d, *J* = 8.2 Hz, 2H), 6.65 – 6.57 (m, 1H), 5.69 (d, *J* = 17.6 Hz, 1H), 5.22 (d, *J* = 10.9 Hz, 1H), 0.44 (s, 6H).

^{13}C NMR (126 MHz, Chloroform-*d*) δ 137.8, 136.2, 133.7, 132.2, 129.4, 127.9, 126.6, 126.0, 122.2, 115.0, 106.8, 92.7, -0.8.

HRMS-ESI (m/z): $[M+H]^+$ Calcd for $C_{18}H_{19}Si$ 263.1251; Found: 263.1249.

((4-ethynylphenyl)ethynyl)dimethyl(phenyl)silane (3r)

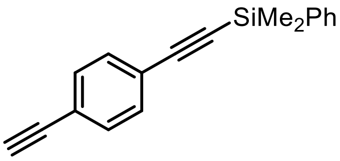

Prepared according to general procedure F using ((4-ethynylphenyl)ethynyl)(methyl)sulfane (34.4 mg, 0.2 mmol), $PhMe_2SiCl$ (68.0 mg, 0.4 mmol), CoI_2 (6.3 mg, 0.02 mmol), **L8** (7.0 mg, 0.03 mmol), Zn (26.2 mg, 0.4 mmol), DMF (2.0 mL) at room temperature for 4 h. The crude material was purified by flash chromatography on silica gel to provide the title compound **3r** as a white solid (46.0 mg, 88% yield)**.**

^{1}H NMR (400 MHz, Chloroform-*d*) δ 7.72 – 7.67 (m, 2H), 7.46 – 7.39 (m, 7H), 3.18 (s, 1H), 0.51 (s, 6H).

^{13}**C NMR** (126 MHz, Chloroform-*d*) δ 136.7, 133.7, 131.9, 129.5, 127.9, 126.0, 123.3, 122.3, 105.9, 94.4, 83.1, 79.1, -0.9.

Melting point: 85-86 °C

HRMS-ESI (m/z): $[M+H]^+$ Calcd for $C_{18}H_{17}Si$ 261.1094; Found: 261.1091.

3-(4-((trimethylsilyl)ethynyl)phenoxy)propan-1-ol (3s)

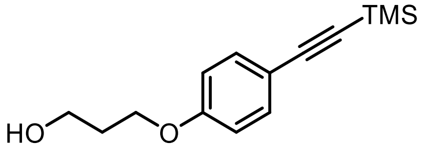

Prepared according to general procedure F using 3-(4-((methylthio)ethynyl)phenyl)propan-1-ol (41.2 mg, 0.2 mmol), TMSCl (43.5 mg, 0.4 mmol), CoI_2 (6.3 mg, 0.02 mmol), **L8** (7.0 mg, 0.03 mmol), Zn (26.2 mg, 0.4 mmol), DMF (2.0 mL) at room temperature for 4 h. The crude material was purified by flash chromatography on silica gel to provide the title compound **3s** as a yellow oil (36.0 mg, 73% yield). Characterization data matched those reported in the literature.[16]

^{1}**H NMR** (400 MHz, Chloroform-*d*) δ 7.42 – 7.37 (m, 2H), 6.83 – 6.79 (m, 2H), 4.10 (t, *J* = 6.0 Hz, 2H), 3.85 (t, *J* = 5.9 Hz, 2H), 2.05 – 2.01 (m, 2H), 1.88 (s, 1H), 0.23 (s, 9H).

^{13}**C NMR** (126 MHz, Chloroform-*d*) δ 158.9, 133.5, 115.3, 114.3, 105.1, 92.5, 65.5, 60.2, 31.8, 0.0.

N-ethyl-4-((trimethylsilyl)ethynyl)aniline (3t)

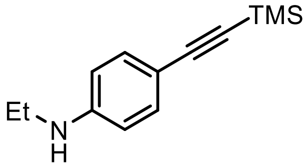

Prepared according to general procedure F using N-ethyl-4-((methylthio)ethynyl)aniline (38.2 mg, 0.2 mmol), TMSCl (43.5 mg, 0.4 mmol), CoI_2 (6.3 mg, 0.02 mmol), **L8** (7.0 mg, 0.03 mmol), Zn (26.2 mg, 0.4 mmol), DMF (2.0 mL) at room temperature for 4 h. The crude material was purified by flash chromatography on silica gel to provide the title compound **3t** as a brown solid (25.0 mg, 58% yield).

^{1}**H NMR** (500 MHz, Chloroform-*d*) δ 7.06 (d, *J* = 8.7 Hz, 2H), 6.25 (d, *J* = 8.6 Hz, 2H), 3.54 (s, 1H), 2.92 (q, *J* = 7.2 Hz, 2H), 1.02 (t, *J* = 7.1 Hz, 3H), 0.00 (s, 9H).

^{13}**C NMR** (126 MHz, Chloroform-*d*) δ 148.5, 133.3, 112.0, 110.8, 106.5, 91.0, 38.1, 14.7, 0.2.

Melting point: 88-89 °C

HRMS-ESI (m/z): $[M+H]^+$ Calcd for $C_{13}H_{20}NSi$ 218.1359; Found: 218.1360.

dimethyl(phenyl)(4-phenylbut-1-yn-1-yl)silane (3u)

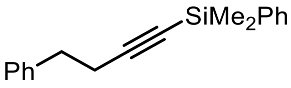

Prepared according to general procedure F using methyl(4-phenylbut-1-yn-1-yl)sulfane (35.2 mg, 0.2 mmol), $PhMe_2SiCl$ (68.0 mg, 0.4 mmol), CoI_2 (6.3 mg, 0.02 mmol), **L8** (7.0 mg, 0.03 mmol), Zn (26.2 mg,

0.4 mmol), DMF (2.0 mL) at room temperature for 4 h. The crude material was purified by flash chromatography on silica gel to provide the title compound **3u** as a colorless oil (51.0 mg, 97% yield). Characterization data matched those reported in the literature.[12]

^{1}H NMR (500 MHz, Chloroform-*d*) δ 7.57 – 7.49 (m, 2H), 7.33 – 7.27 (m, 3H), 7.22 (t, *J* = 7.5 Hz, 2H), 7.16 (d, *J* = 7.3 Hz, 3H), 2.80 (t, *J* = 7.5 Hz, 2H), 2.50 (t, *J* = 7.5 Hz, 2H), 0.33 (s, 6H).

^{13}C NMR (126 MHz, Chloroform-*d*) δ 140.5, 137.4, 133.6, 129.2, 128.5, 128.3, 127.8, 126.3, 108.5, 83.3, 34.9, 22.2, -0.7.

(cyclohexylethynyl)dimethyl(phenyl)silane (3v)

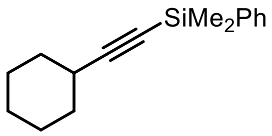

Prepared according to general procedure F using (cyclohexylethynyl)(methyl)sulfane (30.8 mg, 0.2 mmol), TMSCl (43.5 mg, 0.4 mmol), CoI_2 (6.3 mg, 0.02 mmol), **L8** (7.0 mg, 0.03 mmol), Zn (26.2 mg, 0.4 mmol), DMF (2.0 mL) at room temperature for 4 h. The crude material was purified by flash chromatography on silica gel to provide the title compound **3v** as a colorless oil (42.0 mg, 86% yield). Characterization data matched those reported in the literature.[12]

^{1}H NMR (400 MHz, Chloroform-*d*) δ 7.74 – 7.64 (m, 2H), 7.42 – 7.38 (m, 3H), 2.50 (t, *J* = 8.9 Hz, 1H), 1.93 – 1.83 (m, 2H), 1.82 – 1.72 (m, 2H), 1.59 – 1.49 (m, 3H), 1.41 – 1.30 (m, 3H), 0.43 (s, 6H).

^{13}C NMR (101 MHz, Chloroform-*d*) δ 137.9, 133.7, 129.1, 127.7, 113.7, 81.6, 32.5, 30.1, 25.8, 24.8, -0.5.

(3,3-dimethylbut-1-yn-1-yl)dimethyl(phenyl)silane (3w)

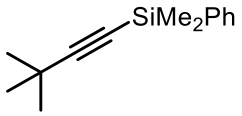

Prepared according to general procedure F using (3,3-dimethylbut-1-yn-1-yl)(methyl)sulfane (25.6 mg, 0.2 mmol), TMSCl (43.5 mg, 0.4 mmol), CoI_2 (6.3 mg, 0.02 mmol), **L8** (7.0 mg, 0.03 mmol), Zn (26.2 mg, 0.4 mmol), DMF (2.0 mL) at room temperature for 4 h. The crude material was purified by flash chromatography on silica gel to provide the title compound **3w** as a colorless oil (35.0 mg, 81% yield). Characterization data matched those reported in the literature.[17]

^{1}H NMR (400 MHz, Chloroform-*d*) δ 7.68 – 7.62 (m, 2H), 7.41 – 7.36 (m, 3H), 1.28 (s, 9H), 0.38 (s, 6H).

^{13}C NMR (101 MHz, Chloroform-*d*) δ 138.0, 133.7, 129.1, 127.7, 118.0, 79.6, 30.9, 28.2, -0.4.

(cyclohex-1-en-1-ylethynyl)dimethyl(phenyl)silane (3x)

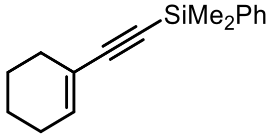

Prepared according to general procedure F using (cyclohex-1-en-1-ylethynyl)(methyl)sulfane (30.4 mg, 0.2 mmol), TMSCl (43.5 mg, 0.4 mmol), CoI_2 (6.3 mg, 0.02 mmol), **L8** (7.0 mg, 0.03 mmol), Zn (26.2 mg, 0.4 mmol), DMF (2.0 mL) at room temperature for 4 h. The crude material was purified by flash chromatography on silica gel to provide the title compound **3x** as a colorless oil (45.0 mg, 94% yield). Characterization data matched those reported in the literature.[13]

^{1}H NMR (400 MHz, Chloroform-*d*) δ 7.72 – 7.59 (m, 2H), 7.40 – 7.36 (m, 3H), 6.27 – 6.22 (m, 1H), 2.20 – 2.14 (m, 2H), 2.14 – 2.08 (m, 2H), 1.68 – 1.57 (m, 4H), 0.43 (s, 6H).

^{13}C NMR (101 MHz, Chloroform-*d*) δ 137.5, 136.7, 133.7, 129.2, 127.8, 120.7, 109.0, 88.7, 29.0, 25.7, 22.2, 21.4, -0.7.

(E)-trimethyl(4-phenylbut-3-en-1-yn-1-yl)silane (3y)

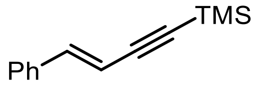

Prepared according to general procedure F using (E)-methyl(4-phenylbut-3-en-1-yn-1-yl)sulfane (34.8 mg, 0.2 mmol), TMSCl (43.5 mg, 0.4 mmol), CoI_2 (6.3 mg, 0.02 mmol), **L8** (7.0 mg, 0.03 mmol), Zn (26.2 mg, 0.4 mmol), DMF (2.0 mL) at room temperature for 4 h. The crude material was purified by flash chromatography on silica gel to provide the title compound **3y** as a yellow oil (31.0 mg, 78% yield). Characterization data matched those reported in the literature.[4]

^{1}H NMR (500 MHz, Chloroform-*d*) δ 7.38 (d, *J* = 7.1 Hz, 2H), 7.35 – 7.27 (m, 3H), 7.01 (d, *J* = 16.3 Hz, 1H), 6.18 (d, *J* = 16.3 Hz, 1H), 0.24 (s, 9H).

^{13}C NMR (126 MHz, Chloroform-*d*) δ 142.4, 136.1, 128.74, 128.7, 126.3, 108.0, 104.4, 96.9, -0.1.

((9H-fluoren-2-yl)ethynyl)trimethylsilane (3z)

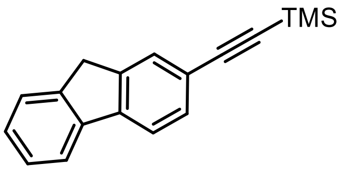

Prepared according to general procedure F using ((9H-fluoren-2-yl)ethynyl)(methyl)sulfane (47.2 mg, 0.2 mmol), TMSCl (43.5 mg, 0.4 mmol), CoI_2 (6.3 mg, 0.02 mmol), **L8** (7.0 mg, 0.03 mmol), Zn (26.2 mg, 0.4 mmol), DMF (2.0 mL) at room temperature for 4 h. The crude material was purified by flash chromatography on silica gel to provide the title compound **3z** as a white solid (46.0 mg, 88% yield). Characterization data matched those reported in the literature.[4]

^{1}H NMR (500 MHz, Chloroform-*d*) δ 7.77 (d, *J* = 7.5 Hz, 1H), 7.71 (d, *J* = 7.9 Hz, 1H), 7.67 (s, 1H), 7.58 – 7.50 (m, 2H), 7.39 (t, *J* = 7.4 Hz, 1H), 7.33 (t, *J* = 7.4 Hz, 1H), 3.88 (s, 2H), 0.31 (s, 9H).

^{13}C NMR (126 MHz, Chloroform-*d*) δ 143.6, 143.0, 142.0, 141.0, 130.8, 128.5, 127.2, 126.9, 125.0, 121.1, 120.2, 119.6, 105.9, 94.0, 36.7, 0.

trimethyl(naphthalen-2-ylethynyl)silane (3aa)

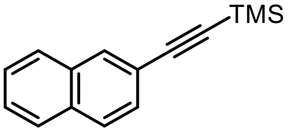

Prepared according to general procedure F using methyl(naphthalen-2-ylethynyl)sulfane (39.6 mg, 0.2 mmol), TMSCl (43.5 mg, 0.4 mmol), CoI_2 (6.3 mg, 0.02 mmol), **L8** (7.0 mg, 0.03 mmol), Zn (26.2 mg, 0.4 mmol), DMF (2.0 mL) at room temperature for 4 h. The crude material was purified by flash chromatography on silica gel to provide the title compound **3aa** as a white solid (36.0 mg, 80% yield). Characterization data matched those reported in the literature.[4]

^{1}H NMR (500 MHz, Chloroform-*d*) δ 8.02 (s, 1H), 7.82 – 7.75 (m, 3H), 7.53 – 7.47 (m, 3H), 0.31 (s, 9H).

^{13}C NMR (126 MHz, Chloroform-*d*) δ 132.89, 132.86, 132.0, 128.6, 127.8, 127.77, 127.72, 126.7, 126.5, 120.4, 105.5, 94.5, 0.0.

dimethyl(phenyl)(thiophen-2-ylethynyl)silane **(3ab)**

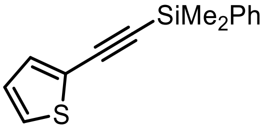

Prepared according to general procedure F using 2-((methylthio)ethynyl)thiophene (30.8 mg, 0.2 mmol), TMSCl (43.5 mg, 0.4 mmol), CoI_2 (6.3 mg, 0.02 mmol), **L8** (7.0 mg, 0.03 mmol), Zn (26.2 mg, 0.4 mmol), DMF (2.0 mL) at room temperature for 4 h. The crude material was purified by flash chromatography on silica gel to provide the title compound **3ab** as a yellow oil (38.0 mg, 78% yield). Characterization data matched those reported in the literature.[18]

^{1}H NMR (400 MHz, Chloroform-*d*) δ 7.74 – 7.67 (m, 2H), 7.45 – 7.40 (m, 3H), 7.32 – 7.29 (m, 1H), 7.28 – 7.26 (m, 1H), 7.00 – 6.96 (m, 1H), 0.53 (s, 6H).

^{13}C NMR (126 MHz, Chloroform-*d*) δ 136.6, 133.7, 132.9, 129.5, 127.9, 127.6, 126.9, 122.9, 99.1, 96.6, -1.0.

dimethyl(phenyl)(thiophen-3-ylethynyl)silane **(3ac)**

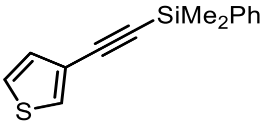

Prepared according to general procedure F using 3-((methylthio)ethynyl)thiophene (30.8 mg, 0.2 mmol), TMSCl (43.5 mg, 0.4 mmol), CoI_2 (6.3 mg, 0.02 mmol), **L8** (7.0 mg, 0.03 mmol), Zn (26.2 mg, 0.4 mmol), DMF (2.0 mL) at room temperature for 4 h. The crude material was purified by flash chromatography on silica gel to provide the title compound **3ac** as a colorless oil (41.0 mg, 85% yield). Characterization data matched those reported in the literature.[13]

^{1}H NMR (500 MHz, Chloroform-*d*) δ 7.65 – 7.60 (m, 2H), 7.45 (d, *J* = 2.8 Hz, 1H), 7.35 – 7.31 (m, 3H), 7.18 – 7.15 (m, 1H), 7.09 (d, *J* = 5.0 Hz, 1H), 0.43 (s, 6H).

^{13}C NMR (126 MHz, Chloroform-*d*) δ 136.9, 133.7, 130.1, 129.9, 129.4, 127.9, 125.2, 122.2, 101.6, 91.8, -0.8.

3-((dimethyl(phenyl)silyl)ethynyl)pyridine **(3ad)**

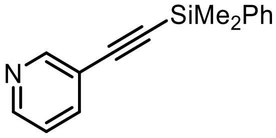

Prepared according to general procedure F using 3-((methylthio)ethynyl)pyridine (29.8 mg, 0.2 mmol), TMSCl (43.5 mg, 0.4 mmol), CoI_2 (6.3 mg, 0.02 mmol), **L8** (7.0 mg, 0.03 mmol), Zn (26.2 mg, 0.4 mmol), DMF (2.0 mL) at room temperature for 4 h. The crude material was purified by flash chromatography on silica gel to provide the title compound **3ad** as a colorless oil (31.0 mg, 65% yield). Characterization data matched those reported in the literature.[12]

^{1}H NMR (400 MHz, Chloroform-*d*) δ 8.78 – 8.74 (m, 1H), 8.57 (dd, *J* = 4.9, 1.6 Hz, 1H), 7.83 – 7.77 (m, 1H), 7.71 (dd, *J* = 7.3, 2.2 Hz, 2H), 7.43 (dd, *J* = 4.9, 1.8 Hz, 3H), 7.29 – 7.24 (m, 1H), 0.54 (s, 6H).

^{13}C NMR (101 MHz, Chloroform-*d*) δ 152.7, 148.9, 138.9, 136.4, 133.7, 129.6, 128.0, 122.9, 120.1, 103.0, 96.2, -1.0.

6-((trimethylsilyl)ethynyl)quinoline (3ae)

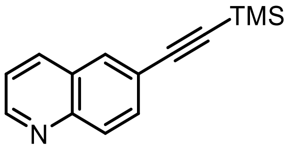

Prepared according to general procedure F using 6-((methylthio)ethynyl)quinoline (39.8 mg, 0.2 mmol), TMSCl (43.5 mg, 0.4 mmol), CoI_2 (6.3 mg, 0.02 mmol), **L8** (7.0 mg, 0.03 mmol), Zn (26.2 mg, 0.4 mmol), DMF (2.0 mL) at room temperature for 4 h. The crude material was purified by flash chromatography on silica gel to provide the title compound **3ae** as a yellow oil (20.0 mg, 44% yield)**.**

^{1}H NMR (500 MHz, Chloroform-*d*) δ 8.89 (d, *J* = 3.0 Hz, 1H), 8.09 (d, *J* = 8.2 Hz, 1H), 8.03 (d, *J* = 8.7 Hz, 1H), 7.96 (s, 1H), 7.78 – 7.71 (m, 1H), 7.43 – 7.38 (m, 1H), 0.28 (s, 9H).

^{13}C NMR (126 MHz, Chloroform-*d*) δ 150.9, 147.6, 135.9, 132.4, 131.6, 129.4, 127.9, 121.7, 121.5, 104.4, 95.9, -0.1.

HRMS-ESI (m/z): $[M+H]^+$ Calcd for $C_{14}H_{16}NSi$ 226.1047; Found: 226.1044.

(benzofuran-5-ylethynyl)trimethylsilane (3af)

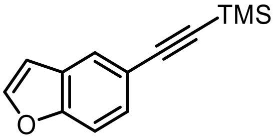

Prepared according to general procedure F using 5-((methylthio)ethynyl)benzofuran (37.6 mg, 0.2 mmol), TMSCl (43.5 mg, 0.4 mmol), CoI_2 (6.3 mg, 0.02 mmol), **L8** (7.0 mg, 0.03 mmol), Zn (26.2 mg, 0.4 mmol),

DMF (2.0 mL) at room temperature for 4 h. The crude material was purified by flash chromatography on silica gel to provide the title compound **3af** as a yellow oil (37.0 mg, 86% yield).

^{1}H NMR (500 MHz, Chloroform-*d*) δ 7.74 (s, 1H), 7.62 (d, *J* = 2.1 Hz, 1H), 7.42 (s, 2H), 6.73 (d, *J* = 2.1 Hz, 1H), 0.27 (s, 9H).

^{13}C NMR (126 MHz, Chloroform-*d*) δ 154.7, 145.8, 128.4, 127.4, 125.2, 117.7, 111.4, 106.5, 105.5, 92.5, 0.0.

HRMS-ESI (m/z): $[M+H]^+$ Calcd for $C_{13}H_{15}OSi$ 215.0887; Found: 215.0884.

1-methyl-5-((trimethylsilyl)ethynyl)-1H-indole (3ag)

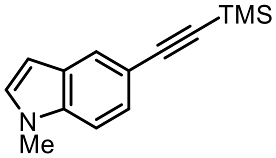

Prepared according to general procedure F using 1-methyl-5-((methylthio)ethynyl)-1H-indole (40.2 mg, 0.2 mmol), TMSCl (43.5 mg, 0.4 mmol), CoI_2 (6.3 mg, 0.02 mmol), **L8** (7.0 mg, 0.03 mmol), Zn (26.2 mg, 0.4 mmol), DMF (2.0 mL) at room temperature for 4 h. The crude material was purified by flash chromatography on silica gel to provide the title compound **3ag** as a dark-brown oil (38.0 mg, 84% yield). Characterization data matched those reported in the literature.[4]

^{1}H NMR (500 MHz, Chloroform-*d*) δ 7.81 (s, 1H), 7.37 – 7.33 (m, 1H), 7.23 (d, *J* = 8.5 Hz, 1H), 7.05 (d, *J* = 3.1 Hz, 1H), 6.46 (d, *J* = 3.0 Hz, 1H), 3.76 (s, 3H), 0.29 (s, 9H).

^{13}C NMR (126 MHz, Chloroform-*d*) δ 136.4, 129.7, 128.1, 125.4, 125.3, 113., 109.1, 107.1, 101.3, 91.0, 32.8, 0.2.

(benzo[b]thiophen-5-ylethynyl)trimethylsilane (3ah)

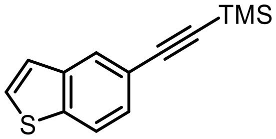

Prepared according to general procedure F using 5-((methylthio)ethynyl)benzo[b]thiophene (40.8 mg, 0.2 mmol), TMSCl (43.5 mg, 0.4 mmol), CoI_2 (6.3 mg, 0.02 mmol), **L8** (7.0 mg, 0.03 mmol), Zn (26.2 mg, 0.4 mmol), DMF (2.0 mL) at room temperature for 4 h. The crude material was purified by flash chromatography on silica gel to provide the title compound **3ah** as a white solid (35.0 mg, 76% yield). Characterization data matched those reported in the literature.[10]

^{1}H NMR (500 MHz, Chloroform-*d*) δ 7.96 (s, 1H), 7.80 (d, *J* = 8.3 Hz, 1H), 7.47 – 7.41 (m, 2H), 7.29 (d, *J* = 5.4 Hz, 1H), 0.28 (s, 9H).

^{13}C NMR (126 MHz, Chloroform-*d*) δ 139.8, 139.4, 127.6, 127.4, 127.3, 123.6, 122.3, 119.0, 105.4, 93.6, 0.0.

9-methyl-2-((trimethylsilyl)ethynyl)-9H-carbazole (3ai)

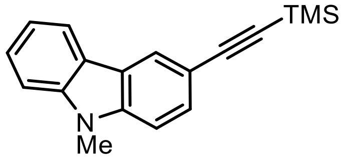

Prepared according to general procedure F using 9-methyl-2-((methylthio)ethynyl)-9H-carbazole (50.2 mg, 0.2 mmol), TMSCl (43.5 mg, 0.4 mmol), CoI_2 (6.3 mg, 0.02 mmol), **L8** (7.0 mg, 0.03 mmol), Zn (26.2 mg, 0.4 mmol), DMF (2.0 mL) at room temperature for 4 h. The crude material was purified by flash chromatography on silica gel to provide the title compound **3ai** as a yellow solid (49.0 mg, 88% yield).

^{1}H NMR (500 MHz, Chloroform-*d*) δ 8.05 – 8.03 (m, 1H), 7.85 (d, *J* = 7.7 Hz, 1H), 7.39 (dd, *J* = 8.4, 1.5 Hz, 1H), 7.30 – 7.26 (m, 1H), 7.15 (d, *J* = 8.2 Hz, 1H), 7.08 – 7.04 (m, 2H), 3.58 (s, 3H), 0.12 (s, 9H).

^{13}C NMR (126 MHz, Chloroform-*d*) δ 141.3, 140.6, 129.6, 126.1, 124.4, 122.6, 122.3, 120.4, 119.4, 113.1, 108.6, 108.3, 106.7, 91.6, 29.0, 0.2.

Melting point: 86-87 °C

HRMS-ESI (m/z): $[M+H]^+$ Calcd for $C_{18}H_{20}NSi$ 278.1360; Found: 278.1357.

(dibenzo[b,d]furan-3-ylethynyl)trimethylsilane (3aj)

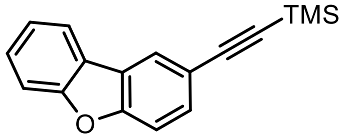

Prepared according to general procedure F using 3-((methylthio)ethynyl)dibenzo[b,d]furan (47.6 mg, 0.2 mmol), TMSCl (43.5 mg, 0.4 mmol), CoI_2 (6.3 mg, 0.02 mmol), **L8** (7.0 mg, 0.03 mmol), Zn (26.2 mg, 0.4 mmol), DMF (2.0 mL) at room temperature for 4 h. The crude material was purified by flash chromatography on silica gel to provide the title compound **3aj** as a white solid (45.0 mg, 85% yield). Characterization data matched those reported in the literature.[19]

^{1}H NMR (500 MHz, Chloroform-*d*) δ 7.90 (s, 1H), 7.73 (d, *J* = 7.7 Hz, 1H), 7.41 – 7.36 (m, 2H), 7.32 – 7.26 (m, 2H), 7.16 (d, *J* = 7.5 Hz, 1H), 0.12 (s, 9H).

^{13}C NMR (126 MHz, Chloroform-*d*) δ 156.6, 155.9, 131.2, 127.6, 124.5, 124.3, 123.6, 123.0, 120.7, 117.7, 111.8, 111.6, 105.2, 93.1, 0.0.

5,6-dimethoxy-2-((1-(4-((trimethylsilyl)ethynyl)benzoyl)piperidin-4-yl)methyl)-2,3-dihydro-1H-inden-1-one (3ak)

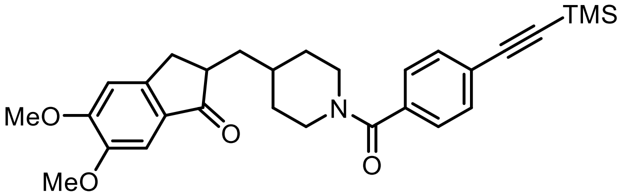

Prepared according to general procedure F using 5,6-dimethoxy-2-((1-(4-((methylthio)ethynyl)benzoyl)piperidin-4-yl)methyl)-2,3-dihydro-1H-inden-1-one (92.6 mg, 0.2 mmol), TMSCl (43.5 mg, 0.4 mmol), CoI_2 (6.3 mg, 0.02 mmol), **L8** (7.0 mg, 0.03 mmol), Zn (26.2 mg, 0.4 mmol),

DMF (2.0 mL) at room temperature for 4 h. The crude material was purified by flash chromatography on silica gel to provide the title compound **3ak** as a yellow solid (64.0 mg, 65% yield).

^{1}H NMR (500 MHz, Chloroform-*d*) δ 7.47 (d, *J* = 8.1 Hz, 2H), 7.32 (d, *J* = 8.1 Hz, 2H), 7.15 (s, 1H), 6.85 (s, 1H), 4.68 (s, 1H), 3.95 (s, 3H), 3.89 (s, 3H), 3.68 (s, 1H), 3.29 – 3.21 (m, 1H), 3.00 – 2.66 (m, 4H), 1.97 – 1.55 (m, 5H), 1.43 – 1.30 (m, 2H), 0.24 (d, *J* = 1.6 Hz, 9H).

^{13}C NMR (126 MHz, Chloroform-*d*) δ 207.2, 169.6, 149.5, 148.5, 136.0, 131.9, 129.1, 126.8, 124.4, 107.3, 104.4, 104.2, 95.8, 56.2, 56.1, 45.0, 38.5, 34.6, 33.3, 30.6, 29.6, -0.2.

Melting point: 147-148 °C

HRMS-ESI (m/z): $[M+H]^+$ Calcd for $C_{29}H_{36}NO_4Si$ 490.2408; Found: 490.2403.

(4-(8-chloro-5,6-dihydro-11H-benzo[5,6]cyclohepta[1,2-b]pyridin-11-ylidene)piperidin-1-yl)(4-((trimethylsilyl)ethynyl)phenyl)methanone (3al)

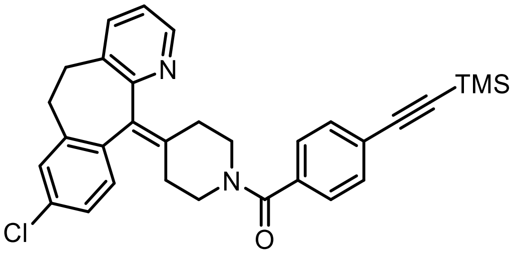

Prepared according to general procedure F using (4-(8-chloro-5,6-dihydro-11H-benzo[5,6]cyclohepta[1,2-b]pyridin-11-ylidene)piperidin-1-yl)(4-((methylthio)ethynyl)phenyl)methanone (96.8 mg, 0.2 mmol), TMSCl (43.5 mg, 0.4 mmol), CoI_2 (6.3 mg, 0.02 mmol), **L8** (7.0 mg, 0.03 mmol), Zn (26.2 mg, 0.4 mmol), DMF (2.0 mL) at room temperature for 4 h. The crude material was purified by flash chromatography on silica gel to provide the title compound **3al** as a yellow oil (42.0 mg, 41% yield).

^{1}H NMR (500 MHz, Chloroform-*d*) δ 8.23 (s, 1H), 7.32 (d, *J* = 8.2 Hz, 3H), 7.19 (d, *J* = 8.2 Hz, 2H), 6.99 (d, *J* = 26.2 Hz, 4H), 4.09 – 3.92 (m, 1H), 3.56 – 3.34 (m, 1H), 3.29 – 3.14 (m, 3H), 2.73 – 2.63 (m, 2H), 2.37 – 2.03 (m, 4H), 1.12 – 1.09 (m, 1H), 0.09 (s, 9H).

^{13}C NMR (126 MHz, Chloroform-*d*) δ 169.7, 156.5, 146.4, 139.4, 137.9, 137.4, 136.8, 135.7, 134.5, 133.5, 133.1, 131.9, 130.4, 129.0, 126.8, 126.2, 124.6, 122.4, 104.1, 95.9, 48.5, 43.3, 31.6, 31.5, 29.7, 14.2, -0.2.

HRMS-ESI (m/z): $[M+H]^+$ Calcd for $C_{31}H_{32}ClN_2OSi$ 511.1967; Found: 511.1964.

3,7-dimethyloct-6-en-1-yl 4-((trimethylsilyl)ethynyl)benzoate (3am)

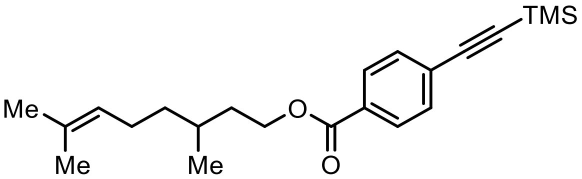

Prepared according to general procedure F using 3,7-dimethyloct-6-en-1-yl 4-((methylthio)ethynyl)benzoate (66.0 mg, 0.2 mmol), TMSCl (43.5 mg, 0.4 mmol), CoI_2 (6.3 mg, 0.02 mmol), **L8** (7.0 mg, 0.03 mmol), Zn (26.2 mg, 0.4 mmol), DMF (2.0 mL) at room temperature for 4 h. The

crude material was purified by flash chromatography on silica gel to provide the title compound **3am** as a yellow oil (37.0 mg, 52% yield).

^{1}H NMR (500 MHz, Chloroform-*d*) δ 7.96 (d, *J* = 8.5 Hz, 2H), 7.51 (d, *J* = 8.5 Hz, 2H), 5.12 – 5.07 (m, 1H), 4.38 – 4.32 (m, 2H), 2.06 – 1.95 (m, 2H), 1.85 – 1.77 (m, 1H), 1.67 (s, 3H), 1.66 – 1.62 (m, 1H), 1.60 (s, 3H), 1.43 – 1.37 (m, 1H), 1.32 – 1.17 (m, 2H), 0.97 (d, *J* = 6.5 Hz, 3H), 0.26 (s, 9H).

^{13}C NMR (126 MHz, Chloroform-*d*) δ 166.1, 131.8, 131.4, 130.1, 129.3, 127.6, 124.5, 104.1, 97.6, 63.7, 37.0, 35.5, 29.5, 25.7, 25.4, 19.5, 17.6, -0.2.

HRMS-ESI (m/z): $[M+H]^+$ Calcd for $C_{22}H_{33}O_2Si$ 357.2244; Found: 357.2240.

(1R,2S,5R)-2-isopropyl-5-methylcyclohexyl 4-((trimethylsilyl)ethynyl)benzoate (3an)

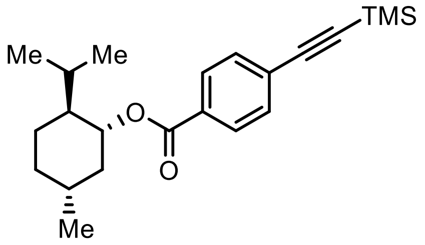

Prepared according to general procedure F using (1R,2S,5R)-2-isopropyl-5-methylcyclohexyl 4-((methylthio)ethynyl)benzoate (66.0 mg, 0.2 mmol), TMSCl (43.5 mg, 0.4 mmol), CoI_2 (6.3 mg, 0.02 mmol), **L8** (7.0 mg, 0.03 mmol), Zn (26.2 mg, 0.4 mmol), DMF (2.0 mL) at room temperature for 4 h. The crude material was purified by flash chromatography on silica gel to provide the title compound **3an** as a yellow oil (40.0 mg, 56% yield). Characterization data matched those reported in the literature.[20]

^{1}H NMR (500 MHz, Chloroform-*d*) δ 7.97 (d, *J* = 8.4 Hz, 2H), 7.51 (d, *J* = 8.4 Hz, 2H), 4.97 – 4.89 (m, 1H), 2.12 (d, *J* = 11.8 Hz, 1H), 1.99 – 1.90 (m, 1H), 1.73 (d, *J* = 11.5 Hz, 2H), 1.60 – 1.51 (m, 2H), 1.17 – 1.05 (m, 2H), 0.95 – 0.88 (m, 7H), 0.79 (d, *J* = 7.0 Hz, 3H), 0.26 (s, 9H).

^{13}C NMR (126 MHz, Chloroform-*d*) δ 165.5, 131.8, 130.4, 129.3, 127.5, 104.2, 97.4, 75.1, 47.2, 40.9, 34.3, 31.4, 26.5, 23.6, 22.0, 20.7, 16.5, -0.2.

4-((trimethylsilyl)ethynyl)phenyl 5-(2,5-dimethylphenoxy)-2,2-dimethylpentanoate (3ao)

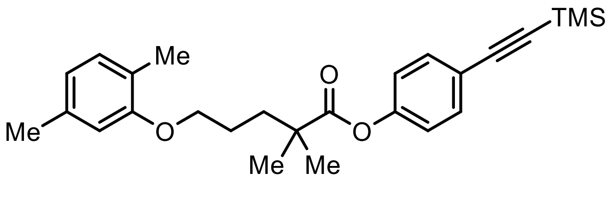

Prepared according to general procedure F using 4-((methylthio)ethynyl)phenyl 5-(2,5-dimethylphenoxy)-2,2-dimethylpentanoate (79.2 mg, 0.2 mmol), TMSCl (43.5 mg, 0.4 mmol), CoI_2 (6.3 mg, 0.02 mmol), **L8** (7.0 mg, 0.03 mmol), Zn (26.2 mg, 0.4 mmol), DMF (2.0 mL) at room temperature for 4 h. The crude material was purified by flash chromatography on silica gel to provide the title compound **3ao** as a white solid (70.0 mg, 83% yield).

^{1}H NMR (500 MHz, Chloroform-*d*) δ 7.48 (d, *J* = 8.7 Hz, 2H), 7.02 (d, *J* = 7.5 Hz, 1H), 6.99 (d, *J* = 8.7 Hz, 2H), 6.69 (d, *J* = 7.3 Hz, 1H), 6.65 (s, 1H), 4.00 (t, *J* = 5.1 Hz, 2H), 2.33 (s, 3H), 2.19 (s, 3H), 1.93 – 1.85 (m, 4H), 1.38 (s, 6H), 0.27 (s, 9H).

^{13}C NMR (126 MHz, Chloroform-*d*) δ 176.0, 156.9, 151.1, 136.5, 133.1, 130.4, 123.7, 121.6, 120.9, 120.7, 112.0, 104.4, 94.2, 67.8, 42.5, 37.2, 25.3, 25.2, 21.4, 15.8, 0.0.

Melting point: 66-67 °C

HRMS-ESI (m/z): $[M+H]^+$ Calcd for $C_{26}H_{35}O_3Si$ 423.2350; Found: 423.2344.

(3S,8S,9S,10R,13R,14S,17R)-10,13-dimethyl-17-((R)-6-methylheptan-2-yl)-2,3,4,7,8,9,10,11,12,13,14,15,16,17-tetradecahydro-1H-cyclopenta[a]phenanthren-3-yl 4-((trimethylsilyl)ethynyl)benzoate (3ap)

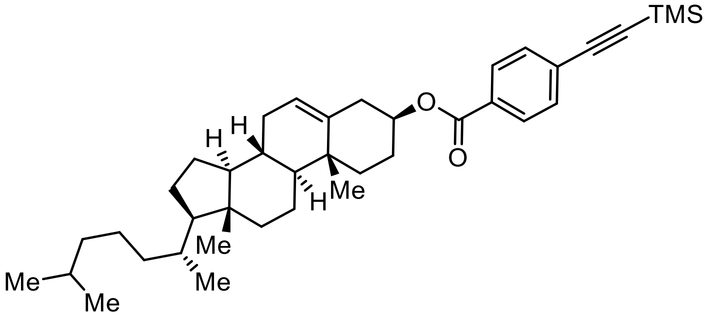

Prepared according to general procedure F using (3S,8S,9S,10R,13R,14S,17R)-10,13-dimethyl-17-((R)-6-methylheptan-2-yl)-2,3,4,7,8,9,10,11,12,13,14,15,16,17-tetradecahydro-1H-cyclopenta[a]phenanthren-3-yl 4-((methylthio)ethynyl)benzoate (112.0 mg, 0.2 mmol), TMSCl (43.5 mg, 0.4 mmol), CoI_2 (6.3 mg, 0.02 mmol), **L8** (7.0 mg, 0.03 mmol), Zn (26.2 mg, 0.4 mmol), DMF (2.0 mL) at room temperature for 4 h. The crude material was purified by flash chromatography on silica gel to provide the title compound **3ap** as a white solid (72.0 mg, 62% yield)**.**

^{1}H NMR (500 MHz, Chloroform-*d*) δ 7.96 (d, *J* = 8.4 Hz, 2H), 7.50 (d, *J* = 8.4 Hz, 2H), 5.42 (d, *J* = 3.6 Hz, 1H), 4.88 – 4.81 (m, 1H), 2.48 – 2.44 (m, 2H), 2.04 – 1.97 (m, 3H), 1.93 – 1.89 (m, 1H), 1.87 – 1.81 (m, 1H), 1.76 – 1.69 (m, 1H), 1.64 – 1.55 (m, 3H), 1.53 – 1.45 (m, 4H), 1.39 – 1.32 (m, 3H), 1.26 (t, *J* = 10.1 Hz, 2H), 1.22 – 1.17 (m, 2H), 1.14 – 1.09 (m, 3H), 1.06 (s, 3H), 1.03 – 0.98 (m, 3H), 0.92 (d, *J* = 6.5 Hz, 3H), 0.88 – 0.86 (m, 6H), 0.69 (s, 3H), 0.26 (s, 9H).

^{13}C NMR (126 MHz, Chloroform-*d*) δ 165.4, 139.6, 131.8, 130.4, 129.3, 127.5, 122.8, 104.2, 97.4, 74.8, 56.7, 56.2, 50.1, 42.3, 39.7, 39.5, 38.2, 37.0, 36.6, 36.2, 35.8, 31.9, 31.9, 28.2, 28.0, 27.9, 24.3, 23.8, 22.8, 22.6, 21.1, 19.4, 18.7, 11.9, -0.2.

Melting point: 200-201 °C

HRMS-ESI (m/z): $[M+H]^+$ Calcd for $C_{39}H_{59}O_2Si$ 587.4279; Found: 587.4275.

((4-chlorophenyl)ethynyl)trimethylsilane (3aq)

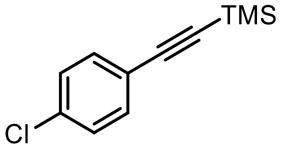

Prepared according to general procedure F using ((4-chlorophenyl)ethynyl)(methyl)sulfane (36.4 mg, 0.2 mmol), TMSCl (43.5 mg, 0.4 mmol), CoI_2 (6.3 mg, 0.02 mmol), **L8** (7.0 mg, 0.03 mmol), Zn (26.2 mg, 0.4 mmol), DMF (2.0 mL) at room temperature for 4 h. The crude material was purified by flash chromatography on silica gel to provide the title compound **3aq** as a colorless solid (31.0 mg, 75% yield). Characterization data matched those reported in the literature.[8]

^{1}H NMR (500 MHz, Chloroform-*d*) δ 7.26 (d, *J* = 8.5 Hz, 2H), 7.14 (d, *J* = 8.5 Hz, 2H), 0.12 (s, 9H).

^{13}C NMR (126 MHz, Chloroform-*d*) δ 134.5, 133.2, 128.5, 121.6, 103.8, 95.3, -0.1.

((4-bromophenyl)ethynyl)trimethylsilane (3ar)

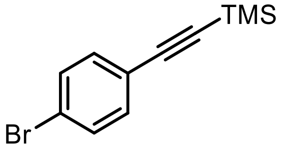

Prepared according to general procedure F using ((4-bromophenyl)ethynyl)(methyl)sulfane (45.2 mg, 0.2 mmol), TMSCl (43.5 mg, 0.4 mmol), CoI_2 (6.3 mg, 0.02 mmol), **L8** (7.0 mg, 0.03 mmol), Zn (26.2 mg, 0.4 mmol), DMF (2.0 mL) at room temperature for 4 h. The crude material was purified by flash chromatography on silica gel to provide the title compound **3ar** as a colorless solid (40.0 mg, 80% yield). Characterization data matched those reported in the literature.[8]

^{1}H NMR (500 MHz, Chloroform-*d*) δ 7.43 (d, *J* = 8.4 Hz, 2H), 7.32 (d, *J* = 8.4 Hz, 2H), 0.25 (s, 9H).

^{13}C NMR (126 MHz, Chloroform-*d*) δ 133.4, 131.5, 122.7, 122.1, 103.9, 95.6, -0.1.

((2-bromophenyl)ethynyl)trimethylsilane (3as)

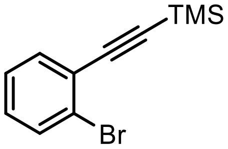

Prepared according to general procedure F using ((2-bromophenyl)ethynyl)(methyl)sulfane (45.2 mg, 0.2 mmol), TMSCl (43.5 mg, 0.4 mmol), CoI_2 (6.3 mg, 0.02 mmol), **L8** (7.0 mg, 0.03 mmol), Zn (26.2 mg, 0.4 mmol), DMF (2.0 mL) at room temperature for 4 h. The crude material was purified by flash chromatography on silica gel to provide the title compound **3as** as a yellow oil (37.0 mg, 74% yield). Characterization data matched those reported in the literature.[21]

^{1}H NMR (500 MHz, Chloroform-*d*) δ 7.58 – 7.55 (m, 1H), 7.49 (dd, *J* = 7.7, 1.6 Hz, 1H), 7.26 – 7.21 (m, 1H), 7.18 – 7.13 (m, 1H), 0.28 (s, 9H).

^{13}C NMR (126 MHz, Chloroform-*d*) δ 133.6, 132.4, 129.5, 126.9, 125.8, 125.3, 103.0, 99.6, -0.2.

((4-iodophenyl)ethynyl)trimethylsilane (3at)

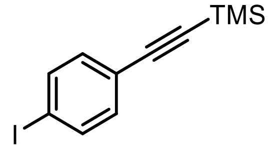

Prepared according to general procedure F using ((4-iodophenyl)ethynyl)(methyl)sulfane (54.8 mg, 0.2 mmol), TMSCl (43.5 mg, 0.4 mmol), CoI_2 (6.3 mg, 0.02 mmol), **L8** (7.0 mg, 0.03 mmol), Zn (26.2 mg, 0.4 mmol), DMF (2.0 mL) at room temperature for 4 h. The crude material was purified by flash chromatography on silica gel to provide the title compound **3at** as a white solid (35.0 mg, 58% yield). Characterization data matched those reported in the literature.[22]

^{1}H NMR (500 MHz, Chloroform-*d*) δ 7.63 (d, *J* = 8.4 Hz, 2H), 7.18 (d, *J* = 8.4 Hz, 2H), 0.24 (s, 9H).

^{13}C NMR (126 MHz, Chloroform-*d*) δ 137.4, 133.4, 122.6, 104.0, 95.9, 94.4, -0.1.

4-((trimethylsilyl)ethynyl)phenyl 4-methylbenzenesulfonate (3au)

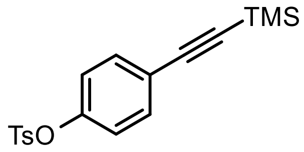

Prepared according to general procedure F using 4-((methylthio)ethynyl)phenyl 4-methylbenzenesulfonate (63.6 mg, 0.2 mmol), TMSCl (43.5 mg, 0.4 mmol), CoI_2 (6.3 mg, 0.02 mmol), **L8** (7.0 mg, 0.03 mmol), Zn (26.2 mg, 0.4 mmol), DMF (2.0 mL) at room temperature for 4 h. The crude material was purified by flash chromatography on silica gel to provide the title compound **3au** as a white solid (56.0 mg, 81% yield)**.**

^{1}H NMR (400 MHz, Chloroform-*d*) δ 7.66 (d, *J* = 8.3 Hz, 2H), 7.36 (d, *J* = 8.5 Hz, 2H), 7.28 (d, *J* = 8.3 Hz, 2H), 6.90 (d, *J* = 8.5 Hz, 2H), 2.42 (s, 3H), 0.22 (s, 9H).

^{13}C NMR (101 MHz, Chloroform-*d*) δ 149.3, 145.5, 133.2, 131.9, 129.7, 128.5, 122.3, 122.1, 103.5, 95.5, 21.6, -0.2.

Melting point: 98-99 °C

HRMS-ESI (m/z): $[M+H]^+$ Calcd for $C_{18}H_{21}O_3SSi$ 345.0975; Found: 345.0970.

4-((trimethylsilyl)ethynyl)phenyl trifluoromethanesulfonate (3av)

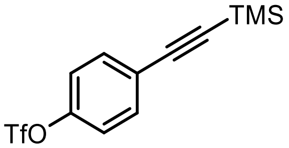

Prepared according to general procedure F using 4-((methylthio)ethynyl)phenyl trifluoromethanesulfonate (59.2 mg, 0.2 mmol), TMSCl (43.5 mg, 0.4 mmol), CoI_2 (6.3 mg, 0.02 mmol), **L8** (7.0 mg, 0.03 mmol), Zn (26.2 mg, 0.4 mmol), DMF (2.0 mL) at room temperature for 4 h. The crude material was purified by flash chromatography on silica gel to provide the title compound **3av** as a yellow oil (50.0 mg, 78% yield). Characterization data matched those reported in the literature.[23]

^{1}H NMR (400 MHz, Chloroform-*d*) δ 7.56 – 7.50 (m, 2H), 7.23 – 7.19 (m, 2H), 0.25 (s, 9H).

^{13}C NMR (101 MHz, Chloroform-*d*) δ 149.0, 133.8, 123.8, 121.3, 120.3, 102.8, 96.7, -0.2.

2-((trimethylsilyl)ethynyl)phenyl trifluoromethanesulfonate (3aw)

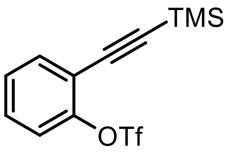

Prepared according to general procedure F using 2-((methylthio)ethynyl)phenyl trifluoromethanesulfonate (59.2 mg, 0.2 mmol), TMSCl (43.5 mg, 0.4 mmol), CoI_2 (6.3 mg, 0.02 mmol), **L8** (7.0 mg, 0.03 mmol), Zn (26.2 mg, 0.4 mmol), DMF (2.0 mL) at room temperature for 4 h. The crude material was purified by flash chromatography on silica gel to provide the title compound **3aw** as a yellow oil (48.0 mg, 75% yield).

^{1}H NMR (500 MHz, Chloroform-*d*) δ 7.42 (d, *J* = 7.5 Hz, 1H), 7.26 – 7.21 (m, 1H), 7.17 (t, *J* = 7.5 Hz, 1H), 7.12 (d, *J* = 8.1 Hz, 1H), 0.12 (s, 9H).

^{13}C NMR (126 MHz, Chloroform-*d*) δ 150.2, 134.1, 129.9, 128.0, 121.5, 120.0 (q, *J* = 321.1 Hz), 118.1, 102.8, 97.2, -0.5.

^{19}F NMR (471 MHz, Chloroform-*d*) δ -73.60.

HRMS-ESI (m/z): $[M+H]^+$ Calcd for $C_{12}H_{14}F_3O_3SSi$ 323.0380; Found: 323.0374.

4-bromo-2-((trimethylsilyl)ethynyl)phenyl trifluoromethanesulfonate (3ax)

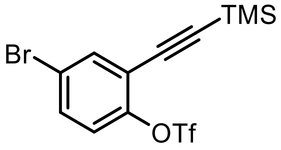

Prepared according to general procedure F using 4-bromo-2-((methylthio)ethynyl)phenyl trifluoromethanesulfonate (74.8 mg, 0.2 mmol), TMSCl (43.5 mg, 0.4 mmol), CoI_2 (6.3 mg, 0.02 mmol), **L8** (7.0 mg, 0.03 mmol), Zn (26.2 mg, 0.4 mmol), DMF (2.0 mL) at room temperature for 4 h. The crude material was purified by flash chromatography on silica gel to provide the title compound **3ax** as a yellow oil (51.0 mg, 64% yield).

^{1}H NMR (500 MHz, Chloroform-*d*) δ 7.70 (d, *J* = 2.3 Hz, 1H), 7.49 (dd, *J* = 8.8, 2.3 Hz, 1H), 7.14 (d, *J* = 8.8 Hz, 1H), 0.26 (s, 9H).

^{13}C NMR (126 MHz, Chloroform-*d*) δ 149.1, 136.6, 132.9, 123.0, 121.3, 120.0, 118.6 (q, *J* = 320.7 Hz), 104.6, 95.8, -0.6.

^{19}F NMR (471 MHz, Chloroform-*d*) δ -73.44.

HRMS-ESI (m/z): $[M+H]^+$ Calcd for $C_{12}H_{13}BrF_3O_3SSi$ 398.9339; Found: 398.9336.

((3-bromo-4-methoxyphenyl)ethynyl)trimethylsilane (3ay)

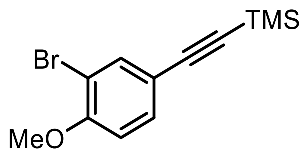

Prepared according to general procedure F using ((3-bromo-4-methoxyphenyl)ethynyl)(methyl)sulfane (51.2 mg, 0.2 mmol), TMSCl (43.5 mg, 0.4 mmol), CoI_2 (6.3 mg, 0.02 mmol), **L8** (7.0 mg, 0.03 mmol), Zn (26.2 mg, 0.4 mmol), DMF (2.0 mL) at room temperature for 4 h. The crude material was purified by flash chromatography on silica gel to provide the title compound **3ay** as a white solid (42.0 mg, 75% yield)**.**

^{1}H NMR (500 MHz, Chloroform-*d*) δ 7.66 (d, *J* = 2.0 Hz, 1H), 7.38 (dd, *J* = 8.5, 2.0 Hz, 1H), 6.79 (d, *J* = 8.5 Hz, 1H), 3.88 (s, 3H), 0.24 (s, 9H).

^{13}C NMR (126 MHz, Chloroform-*d*) δ 156.1, 136.7, 132.3, 116.7, 111.3, 111.2, 103.4, 93.8, 56.2, -0.1.

Melting point: 62-63 °C

HRMS-ESI (m/z): $[M+H]^+$ Calcd for $C_{12}H_{16}BrOSi$ 283.0148; Found: 283.0144.

((5-bromo-2-chlorophenyl)ethynyl)trimethylsilane (3az)

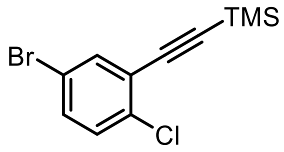

Prepared according to general procedure F using ((5-bromo-2-chlorophenyl)ethynyl)(methyl)sulfane (52.0 mg, 0.2 mmol), TMSCl (43.5 mg, 0.4 mmol), CoI_2 (6.3 mg, 0.02 mmol), **L8** (7.0 mg, 0.03 mmol), Zn (26.2 mg, 0.4 mmol), DMF (2.0 mL) at room temperature for 4 h. The crude material was purified by flash chromatography on silica gel to provide the title compound **3az** as a yellow oil (39.0 mg, 68% yield)**.**

^{1}H NMR (500 MHz, Chloroform-*d*) δ 7.63 (d, *J* = 2.4 Hz, 1H), 7.35 (dd, *J* = 8.6, 2.4 Hz, 1H), 7.24 (d, *J* = 8.6 Hz, 1H), 0.27 (s, 9H).

^{13}C NMR (126 MHz, Chloroform-*d*) δ 136.0, 135.1, 132.4, 130.5, 124.9, 119.6, 102.0, 99.8, -0.3.

HRMS-ESI (m/z): $[M+H]^+$ Calcd for $C_{11}H_{13}BrClSi$ 286.9653; Found: 286.9651.

((5-bromo-2-fluoro-4-methoxyphenyl)ethynyl)trimethylsilane (3ba)

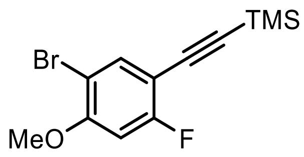

Prepared according to general procedure F using ((5-bromo-2-fluoro-4-methoxyphenyl)ethynyl)(methyl)sulfane (54.8 mg, 0.2 mmol), TMSCl (43.5 mg, 0.4 mmol), CoI_2 (6.3 mg, 0.02 mmol), **L8** (7.0 mg, 0.03 mmol), Zn (26.2 mg, 0.4 mmol), DMF (2.0 mL) at room temperature for 4 h. The crude material was purified by flash chromatography on silica gel to provide the title compound **3ba** as a yellow oil (36.0 mg, 60% yield)**.**

^{1}H NMR (500 MHz, Chloroform-*d*) δ 7.62 (d, *J* = 7.5 Hz, 1H), 6.62 (d, *J* = 10.7 Hz, 1H), 3.88 (s, 3H), 0.25 (s, 9H).
^{13}C NMR (126 MHz, Chloroform-*d*) δ 163.1 (d, *J* = 252.7 Hz), 157.1 (d, *J* = 9.7 Hz), 136.98 (d, *J* = 2.7 Hz), 136.97, 105.60 (d, *J* = 3.6 Hz), 105.6, 105.1 (d, *J* = 17.2 Hz), 100.1 (d, *J* = 26.6 Hz), 99.6 (d, *J* = 3.2 Hz), 96.5, 56.6, -0.2.
^{19}F NMR (471 MHz, Chloroform-*d*) δ -107.31 (dd, *J* = 10.7, 7.5 Hz).
HRMS-ESI (m/z): $[M+H]^+$ Calcd for $C_{12}H_{14}BrFOSi$ 301.0054; Found: 301.0050.

((2-chloro-4,5-difluorophenyl)ethynyl)trimethylsilane (3bb)

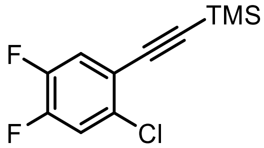

Prepared according to general procedure F using ((2-chloro-4,5-difluorophenyl)ethynyl)(methyl)sulfane (43.6 mg, 0.2 mmol), TMSCl (43.5 mg, 0.4 mmol), CoI_2 (6.3 mg, 0.02 mmol), **L8** (7.0 mg, 0.03 mmol), Zn (26.2 mg, 0.4 mmol), DMF (2.0 mL) at room temperature for 4 h. The crude material was purified by flash chromatography on silica gel to provide the title compound **3bb** as a yellow oil (35.0 mg, 71% yield)**.**
^{1}H NMR (400 MHz, Chloroform-*d*) δ 7.30 (dd, *J* = 10.3, 8.2 Hz, 1H), 7.22 (dd, *J* = 9.8, 7.3 Hz, 1H), 0.27 (s, 9H).
^{13}C NMR (126 MHz, Chloroform-*d*) δ 151.4 – 149.4 (m), 149.2 – 147.0 (m), 132.1 – 131.4 (m), 121.5 (d, *J* = 19.8 Hz), 119.8 – 119.6 (m), 118.5 (d, *J* = 20.6 Hz), 101.3 (d, *J* = 1.8 Hz), 99.1, -0.3.
^{19}F NMR (376 MHz, Chloroform-*d*) δ -132.53 (d, *J* = 21.4 Hz), -138.88 (d, *J* = 21.4 Hz).
HRMS-ESI (m/z): $[M-H]^+$ Calcd for $C_{11}H_{10}ClF_2Si$ 243.0214; Found: 243.0224.

((2,4-dichloro-5-fluorophenyl)ethynyl)trimethylsilane (3bc)

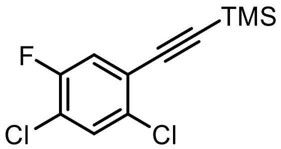

Prepared according to general procedure F using ((2,4-dichloro-5-fluorophenyl)ethynyl)(methyl)sulfane (46.8 mg, 0.2 mmol), TMSCl (43.5 mg, 0.4 mmol), CoI_2 (6.3 mg, 0.02 mmol), **L8** (7.0 mg, 0.03 mmol), Zn (26.2 mg, 0.4 mmol), DMF (2.0 mL) at room temperature for 4 h. The crude material was purified by flash chromatography on silica gel to provide the title compound **3bc** as a white solid (40.0 mg, 77% yield)**.**
^{1}H NMR (500 MHz, Chloroform-*d*) δ 7.41 (d, *J* = 6.7 Hz, 1H), 7.24 (d, *J* = 9.0 Hz, 1H), 0.26 (s, 9H).
^{13}C NMR (126 MHz, Chloroform-*d*) δ 156.2 (d, *J* = 249.7 Hz), 131.8, 130.7, 122.9 (d, *J* = 8.5 Hz), 122.1 (d, *J* = 19.2 Hz), 120.6 (d, *J* = 23.7 Hz), 102.6, 99.2 (d, *J* = 2.4 Hz), -0.3.
^{19}F NMR (471 MHz, Chloroform-*d*) δ -117.48 – -117.55 (m).
Melting point: 37-38 °C

HRMS-ESI (m/z): $[M+H]^+$ Calcd for $C_{11}H_{12}Cl_2FSi$ 258.9956; Found: 258.9951.

((4-(3-chloropropoxy)phenyl)ethynyl)trimethylsilane (3bd)

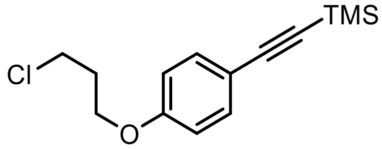

Prepared according to general procedure F using ((4-(3-chloropropoxy)phenyl)ethynyl)(methyl)sulfane (48.0 mg, 0.2 mmol), TMSCl (43.5 mg, 0.4 mmol), CoI_2 (6.3 mg, 0.02 mmol), **L8** (7.0 mg, 0.03 mmol), Zn (26.2 mg, 0.4 mmol), DMF (2.0 mL) at room temperature for 4 h. The crude material was purified by flash chromatography on silica gel to provide the title compound **3bd** as a white solid (36.0 mg, 72% yield)**.**

^{1}H NMR (500 MHz, Chloroform-*d*) δ 7.42 – 7.38 (m, 2H), 6.83 – 6.79 (m, 2H), 4.10 (t, *J* = 5.9 Hz, 2H), 3.73 (t, *J* = 6.3 Hz, 2H), 2.26 – 2.19 (m, 2H), 0.24 (s, 9H).

^{13}C NMR (126 MHz, Chloroform-*d*) δ 158.8, 133.5, 115.5, 114.3, 105.1, 92.5, 64.3, 41.4, 32.2, 0.0.

Melting point: 37-38 °C

HRMS-ESI (m/z): $[M+H]^+$ Calcd for $C_{14}H_{20}ClOSi$ 267.0966; Found: 267.0962.

trimethyl((4-(4,4,5,5-tetramethyl-1,3,2-dioxaborolan-2-yl)phenyl)ethynyl)silane (3be)

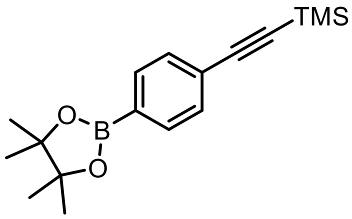

Prepared according to general procedure F using 4,4,5,5-tetramethyl-2-(4-((methylthio)ethynyl)phenyl)-1,3,2-dioxaborolane (54.8 mg, 0.2 mmol), TMSCl (43.5 mg, 0.4 mmol), CoI_2 (6.3 mg, 0.02 mmol), **L8** (7.0 mg, 0.03 mmol), Zn (26.2 mg, 0.4 mmol), DMF (2.0 mL) at room temperature for 4 h. The crude material was purified by flash chromatography on silica gel to provide the title compound **3be** as a white solid (51.0 mg, 85% yield). Characterization data matched those reported in the literature.[4]

^{1}H NMR (500 MHz, Chloroform-d) δ 7.73 (d, J = 8.1 Hz, 2H), 7.45 (d, J = 8.2 Hz, 2H), 1.34 (s, 12H), 0.25 (s, 9H).

^{13}C NMR (126 MHz, Chloroform-*d*) δ 134.4, 131.1, 125.8, 105.2, 95.5, 83.9, 24.9, -0.1.

ethyl((4-methoxyphenyl)ethynyl)dimethylsilane (4a)

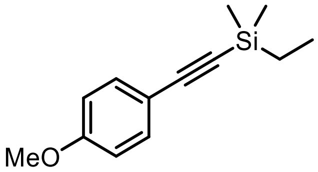

Prepared according to general procedure F using ((4-methoxyphenyl)ethynyl)(methyl)sulfane **1a** (35.6 mg, 0.2 mmol), chloro(ethyl)dimethylsilane (48.8 mg, 0.4 mmol), CoI_2 (6.3 mg, 0.02 mmol), **L8** (7.0 mg, 0.03 mmol), Zn (26.2 mg, 0.4 mmol), DMF (2.0 mL) at room temperature for 4 h. The crude material was purified by flash chromatography on silica gel to provide the title compound **4a** as a yellow oil (42.0 mg, 96% yield).

^{1}H NMR (500 MHz, Chloroform-*d*) δ 7.41 (d, *J* = 8.9 Hz, 2H), 6.82 (d, *J* = 8.8 Hz, 2H), 3.80 (s, 3H), 1.05 (t, *J* = 7.9 Hz, 3H), 0.67 (q, *J* = 7.9 Hz, 2H), 0.21 (s, 6H).

^{13}C NMR (126 MHz, Chloroform-*d*) δ 159.7, 133.5, 115.3, 113.8, 105.6, 91.5, 55.2, 8.2, 7.3, -2.2.

HRMS-ESI (m/z): $[M+H]^+$ Calcd for $C_{13}H_{19}OSi$ 219.1200; Found: 219.1197.

butyl((4-methoxyphenyl)ethynyl)dimethylsilane (4b)

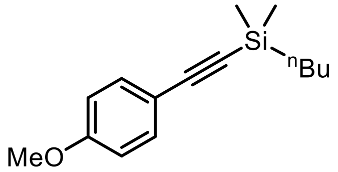

Prepared according to general procedure F using ((4-methoxyphenyl)ethynyl)(methyl)sulfane **1a** (35.6 mg, 0.2 mmol), butylchlorodimethylsilane (60.0 mg, 0.4 mmol), CoI_2 (6.3 mg, 0.02 mmol), **L8** (7.0 mg, 0.03 mmol), Zn (26.2 mg, 0.4 mmol), DMF (2.0 mL) at room temperature for 4 h. The crude material was purified by flash chromatography on silica gel to provide the title compound **4b** as a yellow oil (41.0 mg, 83% yield).

^{1}H NMR (500 MHz, Chloroform-*d*) δ 7.41 (d, *J* = 8.8 Hz, 2H), 6.82 (d, *J* = 8.8 Hz, 2H), 3.80 (s, 3H), 1.46 – 1.37 (m, 4H), 0.92 (t, *J* = 7.0 Hz, 3H), 0.74 – 0.65 (m, 2H), 0.22 (s, 6H).

^{13}C NMR (126 MHz, Chloroform-*d*) δ 159.7, 133.5, 115.4, 113.8, 105.5, 91.8, 55.2, 26.2, 26.1, 16.0, 13.8, -1.6.

HRMS-ESI (m/z): $[M+H]^+$ Calcd for $C_{15}H_{23}OSi$ 247.1513; Found: 247.1513.

((4-methoxyphenyl)ethynyl)dimethyl(3-phenylpropyl)silane (4c)

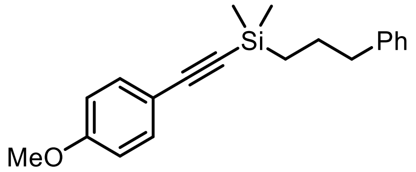

Prepared according to general procedure F using ((4-methoxyphenyl)ethynyl)(methyl)sulfane **1a** (35.6 mg, 0.2 mmol), chlorodimethyl(3-phenylpropyl)silane (84.8 mg, 0.4 mmol), CoI_2 (6.3 mg, 0.02 mmol), **L8** (7.0 mg, 0.03 mmol), Zn (26.2 mg, 0.4 mmol), DMF (2.0 mL) at room temperature for 4 h. The crude material was purified by flash chromatography on silica gel to provide the title compound **4c** as a yellow oil (59.0 mg, 96% yield).

^{1}H NMR (500 MHz, Chloroform-*d*) δ 7.30 (d, *J* = 8.7 Hz, 2H), 7.19 (t, *J* = 7.5 Hz, 2H), 7.14 – 7.07 (m, 3H), 6.72 (d, *J* = 8.8 Hz, 2H), 3.69 (s, 3H), 2.60 (t, *J* = 7.6 Hz, 2H), 1.73 – 1.65 (m, 2H), 0.67 – 0.61 (m, 2H), 0.12 (s, 6H).

^{13}**C NMR** (126 MHz, Chloroform-*d*) δ 159.7, 142.6, 133.5, 128.5, 128.2, 125.6, 115.3, 113.8, 105.8, 91.6, 55.2, 39.4, 25.9, 16.0, -1.6.

HRMS-ESI (m/z): $[M+H]^+$ Calcd for $C_{20}H_{25}OSi$ 308.1596; Found: 308.1586.

4-(((4-methoxyphenyl)ethynyl)dimethylsilyl)butanenitrile (4d)

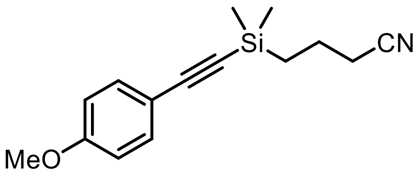

Prepared according to general procedure F using ((4-methoxyphenyl)ethynyl)(methyl)sulfane **1a** (35.6 mg, 0.2 mmol), 4-(chlorodimethylsilyl)butanenitrile (64.4 mg, 0.4 mmol), CoI_2 (6.3 mg, 0.02 mmol), **L8** (7.0 mg, 0.03 mmol), Zn (26.2 mg, 0.4 mmol), DMF (2.0 mL) at room temperature for 4 h. The crude material was purified by flash chromatography on silica gel to provide the title compound **4d** as a yellow oil (47.0 mg, 91% yield)**.**

^{1}**H NMR** (500 MHz, Chloroform-*d*) δ 7.39 (d, *J* = 8.8 Hz, 2H), 6.82 (d, *J* = 8.7 Hz, 2H), 3.80 (s, 3H), 2.42 (t, *J* = 7.0 Hz, 2H), 1.86 – 1.79 (m, 2H), 0.86 – 0.80 (m, 2H), 0.24 (s, 6H).

^{13}**C NMR** (126 MHz, Chloroform-*d*) δ 159.9, 133.5, 119.7, 114.8, 113.8, 106.6, 90.3, 55.2, 20.6, 20.4, 15.8, -1.8.

HRMS-ESI (m/z): $[M+H]^+$ Calcd for $C_{15}H_{20}NOSi$ 258.1309; Found: 258.1305.

((4-methoxyphenyl)ethynyl)dimethyl(phenyl)silane (4e)

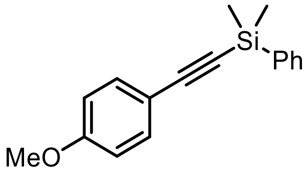

Prepared according to general procedure F using ((4-methoxyphenyl)ethynyl)(methyl)sulfane **1a** (35.6 mg, 0.2 mmol), chlorodimethyl(phenyl)silane (68.0 mg, 0.4 mmol), CoI_2 (6.3 mg, 0.02 mmol), **L8** (7.0 mg, 0.03 mmol), Zn (26.2 mg, 0.4 mmol), DMF (2.0 mL) at room temperature for 4 h. The crude material was purified by flash chromatography on silica gel to provide the title compound **4e** as a yellow oil (45.0 mg, 85% yield). Characterization data matched those reported in the literature.[12]

^{1}**H NMR** (500 MHz, Chloroform-*d*) δ 7.63 – 7.58 (m, 2H), 7.35 (d, *J* = 8.8 Hz, 2H), 7.32 – 7.27 (m, 3H), 6.73 (d, *J* = 8.8 Hz, 2H), 3.69 (s, 3H), 0.39 (s, 6H).

^{13}**C NMR** (126 MHz, Chloroform-*d*) δ 159.9, 137.3, 133.7, 133.6, 129.3, 127.9, 115.1, 113.8, 106.9, 90.3, 55.2, -0.7.

((4-methoxyphenyl)ethynyl)(methyl)diphenylsilane (4f)

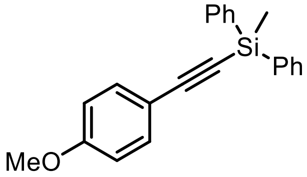

Prepared according to general procedure F using ((4-methoxyphenyl)ethynyl)(methyl)sulfane **1a** (35.6 mg, 0.2 mmol), chloro(methyl)diphenylsilane (92.8 mg, 0.4 mmol), CoI_2 (6.3 mg, 0.02 mmol), **L8** (7.0 mg, 0.03 mmol), Zn (26.2 mg, 0.4 mmol), DMF (2.0 mL) at room temperature for 4 h. The crude material was purified by flash chromatography on silica gel to provide the title compound **4f** as a yellow oil (44.0 mg, 67% yield). Characterization data matched those reported in the literature.[24]

^{1}H NMR (500 MHz, Chloroform-*d*) δ 7.70 (d, *J* = 6.9 Hz, 4H), 7.48 (d, *J* = 8.5 Hz, 2H), 7.38 (q, *J* = 5.5, 5.0 Hz, 6H), 6.84 (d, *J* = 8.3 Hz, 2H), 3.80 (s, 3H), 0.75 (s, 3H).

^{13}C NMR (126 MHz, Chloroform-*d*) δ 160.1, 135.6, 134.6, 133.7, 129.6, 127.9, 114.9, 113.9, 108.5, 88.6, 55.3, -1.9.

isopropyl((4-methoxyphenyl)ethynyl)dimethylsilane (4g)

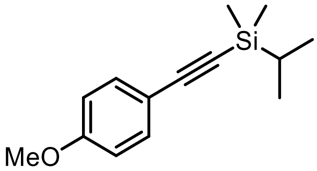

Prepared according to general procedure F using ((4-methoxyphenyl)ethynyl)(methyl)sulfane **1a** (35.6 mg, 0.2 mmol), chloro(isopropyl)dimethylsilane (54.4 mg, 0.4 mmol), CoI_2 (6.3 mg, 0.02 mmol), **L8** (7.0 mg, 0.03 mmol), Zn (26.2 mg, 0.4 mmol), DMF (2.0 mL) at room temperature for 4 h. The crude material was purified by flash chromatography on silica gel to provide the title compound **4g** as a yellow oil (28.0 mg, 61% yield)**.**

^{1}H NMR (500 MHz, Chloroform-*d*) δ 7.41 (d, *J* = 8.8 Hz, 2H), 6.82 (d, *J* = 8.8 Hz, 2H), 3.80 (s, 3H), 1.05 (d, *J* = 7.3 Hz, 6H), 0.92 – 0.86 (m, 1H), 0.18 (s, 6H).

^{13}C NMR (126 MHz, Chloroform-*d*) δ 159.7, 133.5, 115.4, 113.8, 105.8, 90.9, 55.3, 17.4, 14.1, -3.7.

HRMS-ESI (m/z): $[M+H]^+$ Calcd for $C_{14}H_{21}OSi$ 233.1356; Found: 233.1355.

cyclohexyl((4-methoxyphenyl)ethynyl)dimethylsilane (4h)

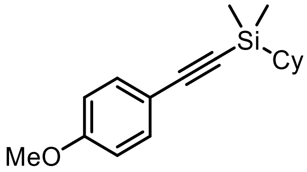

Prepared according to general procedure F using ((4-methoxyphenyl)ethynyl)(methyl)sulfane **1a** (35.6 mg, 0.2 mmol), chloro(cyclohexyl)dimethylsilane (70.4 mg, 0.4 mmol), CoI_2 (6.3 mg, 0.02 mmol), **L8** (7.0 mg, 0.03 mmol), Zn (26.2 mg, 0.4 mmol), DMF (2.0 mL) at room temperature for 4 h. The crude material was purified by flash chromatography on silica gel to provide the title compound **4h** as a yellow oil (29.0 mg, 54% yield)**.**

^{1}**H NMR** (500 MHz, Chloroform-*d*) δ 7.41 (d, *J* = 8.8 Hz, 2H), 6.82 (d, *J* = 8.8 Hz, 2H), 3.80 (s, 3H), 1.83 – 1.75 (m, 4H), 1.30 – 1.15 (m, 6H), 0.77 – 0.70 (m, 1H), 0.16 (s, 6H).
^{13}**C NMR** (126 MHz, Chloroform-*d*) δ 159.7, 133.5, 115.4, 113.8, 105.8, 91.1, 55.3, 27.9, 27.3, 26.9, 25.8, -3.6.
HRMS-ESI (m/z): $[M+H]^+$ Calcd for $C_{17}H_{25}OSi$ 273.1669; Found: 273.1664.

1-((4-methoxyphenyl)ethynyl)-1,1,2,2,2-pentamethyldisilane (4i)

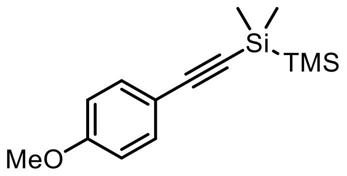

Prepared according to general procedure F using ((4-methoxyphenyl)ethynyl)(methyl)sulfane **1a** (35.6 mg, 0.2 mmol), 1-chloro-1,1,2,2,2-pentamethyldisilane (66.4 mg, 0.4 mmol), CoI_2 (6.3 mg, 0.02 mmol), **L8** (7.0 mg, 0.03 mmol), Zn (26.2 mg, 0.4 mmol), DMF (2.0 mL) at room temperature for 4 h. The crude material was purified by flash chromatography on silica gel to provide the title compound **4i** as a white solid (49.0 mg, 94% yield)**.**
^{1}**H NMR** (500 MHz, Chloroform-*d*) δ 7.23 (d, *J* = 8.9 Hz, 2H), 6.65 (d, *J* = 8.8 Hz, 2H), 3.63 (s, 3H), 0.10 (s, 6H), -0.00 (s, 9H).
^{13}**C NMR** (126 MHz, Chloroform-*d*) δ 159.6, 133.4, 115.7, 113.8, 107.3, 91.3, 55.2, -2.5, -2.9.
Melting point: 30-31 °C
HRMS-ESI (m/z): $[M+H]^+$ Calcd for $C_{14}H_{23}OSi_2$ 263.1282; Found: 263.1288.

allyl((4-methoxyphenyl)ethynyl)dimethylsilane (4j)

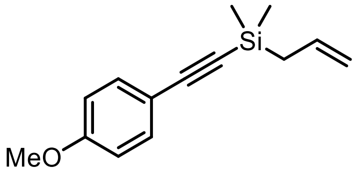

Prepared according to general procedure F using ((4-methoxyphenyl)ethynyl)(methyl)sulfane **1a** (35.6 mg, 0.2 mmol), allylchlorodimethylsilane (53.6 mg, 0.4 mmol), CoI_2 (6.3 mg, 0.02 mmol), **L8** (7.0 mg, 0.03 mmol), Zn (26.2 mg, 0.4 mmol), DMF (2.0 mL) at room temperature for 4 h. The crude material was purified by flash chromatography on silica gel to provide the title compound **4j** as a yellow oil (40.0 mg, 87% yield)**.**
^{1}**H NMR** (500 MHz, Chloroform-*d*) δ 7.41 (d, *J* = 8.7 Hz, 2H), 6.82 (d, *J* = 8.7 Hz, 2H), 5.93 – 5.82 (m, 1H), 4.98 – 4.90 (m, 2H), 3.80 (s, 3H), 1.71 (d, *J* = 8.0 Hz, 2H), 0.23 (s, 6H).
^{13}**C NMR** (126 MHz, Chloroform-*d*) δ 159.8, 134.1, 133.5, 115.1, 113.8, 113.8, 106.1, 90.9, 55.2, 24.1, -2.2.
HRMS-ESI (m/z): $[M+H]^+$ Calcd for $C_{14}H_{19}OSi$ 231.1200; Found: 231.1198.

((4-methoxyphenyl)ethynyl)dimethyl(vinyl)silane (4k)

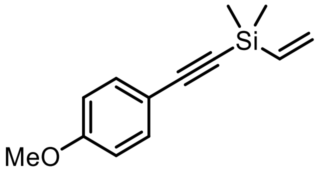

Prepared according to general procedure F using ((4-methoxyphenyl)ethynyl)(methyl)sulfane **1a** (35.6 mg, 0.2 mmol), chlorodimethyl(vinyl)silane (48.0 mg, 0.4 mmol), CoI_2 (6.3 mg, 0.02 mmol), **L8** (7.0 mg, 0.03 mmol), Zn (26.2 mg, 0.4 mmol), DMF (2.0 mL) at room temperature for 4 h. The crude material was purified by flash chromatography on silica gel to provide the title compound **4k** as a yellow oil (41.0 mg, 95% yield)**.**

^{1}H NMR (500 MHz, Chloroform-*d*) δ 7.21 (d, *J* = 8.9 Hz, 2H), 6.61 (d, *J* = 8.9 Hz, 2H), 6.07 – 5.95 (m, 1H), 5.87 – 5.81 (m, 1H), 5.74 – 5.66 (m, 1H), 3.59 (s, 3H), 0.10 (s, 6H).

^{13}C NMR (126 MHz, Chloroform-*d*) δ 159.8, 136.8, 133.5, 132.9, 115.1, 113.8, 106.3, 90.3, 55.2, -1.4.

HRMS-ESI (m/z): $[M+H]^+$ Calcd for $C_{13}H_{17}OSi$ 217.1043; Found: 217.1042.

((4-methoxyphenyl)ethynyl)(methyl)(phenyl)(vinyl)silane (4l)

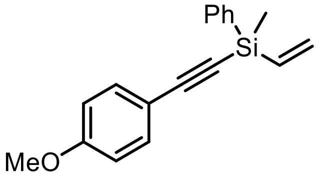

Prepared according to general procedure F using ((4-methoxyphenyl)ethynyl)(methyl)sulfane **1a** (35.6 mg, 0.2 mmol), chloro(methyl)(phenyl)(vinyl)silane (72.8 mg, 0.4 mmol), CoI_2 (6.3 mg, 0.02 mmol), **L8** (7.0 mg, 0.03 mmol), Zn (26.2 mg, 0.4 mmol), DMF (2.0 mL) at room temperature for 4 h. The crude material was purified by flash chromatography on silica gel to provide the title compound **4l** as a yellow oil (46.0 mg, 83% yield)**.**

^{1}H NMR (500 MHz, Chloroform-*d*) δ 7.77 – 7.70 (m, 2H), 7.50 (d, *J* = 8.8 Hz, 2H), 7.45 – 7.39 (m, 3H), 6.86 (d, *J* = 8.9 Hz, 2H), 6.40 – 6.30 (m, 1H), 6.23 – 6.15 (m, 1H), 6.07 – 5.98 (m, 1H), 3.83 (s, 3H), 0.60 (s, 3H).

^{13}C NMR (126 MHz, Chloroform-*d*) δ 160.0, 135.4, 135.0, 134.6, 134.2, 133.6, 129.5, 127.9, 114.9, 113.9, 108.0, 88.3, 55.2, -2.5.

HRMS-ESI (m/z): $[M+H]^+$ Calcd for $C_{18}H_{19}OSi$ 279.1199; Found: 279.1208.

triethyl((4-methoxyphenyl)ethynyl)silane (4m)

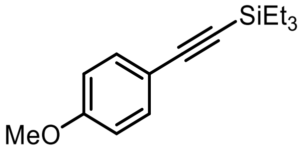

Prepared according to general procedure F using ((4-methoxyphenyl)ethynyl)(methyl)sulfane **1a** (35.6 mg, 0.2 mmol), chlorotriethylsilane (60.0 mg, 0.4 mmol), CoI_2 (6.3 mg, 0.02 mmol), **L8** (7.0 mg, 0.03 mmol), Zn (26.2 mg, 0.4 mmol), DMF (2.0 mL) at room temperature for 4 h. The crude material was

purified by flash chromatography on silica gel to provide the title compound **4m** as a colorless oil (35.0 mg, 71% yield). Characterization data matched those reported in the literature.[25]

^{1}H NMR (500 MHz, Chloroform-*d*) δ 7.33 (d, *J* = 8.8 Hz, 2H), 6.74 (d, *J* = 8.7 Hz, 2H), 3.72 (s, 3H), 0.97 (t, *J* = 7.9 Hz, 9H), 0.59 (q, *J* = 7.9 Hz, 6H).

^{13}C NMR (126 MHz, Chloroform-*d*) δ 159.7, 133.5, 115.6, 113.8, 106.4, 89.8, 55.3, 7.5, 4.5.

tributyl((4-methoxyphenyl)ethynyl)silane (4n)

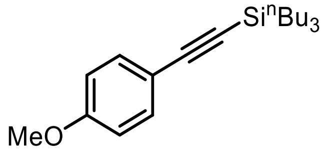

Prepared according to general procedure F using ((4-methoxyphenyl)ethynyl)(methyl)sulfane **1a** (35.6 mg, 0.2 mmol), tributylchlorosilane (93.6 mg, 0.4 mmol), CoI_2 (6.3 mg, 0.02 mmol), **L8** (7.0 mg, 0.03 mmol), Zn (26.2 mg, 0.4 mmol), DMF (2.0 mL) at room temperature for 4 h. The crude material was purified by flash chromatography on silica gel to provide the title compound **4n** as a yellow oil (45.0 mg, 69% yield).

^{1}H NMR (500 MHz, Chloroform-*d*) δ 7.22 (d, *J* = 8.7 Hz, 2H), 6.64 (d, *J* = 8.7 Hz, 2H), 3.63 (s, 3H), 1.26 – 1.17 (m, 12H), 0.74 (t, *J* = 6.9 Hz, 9H), 0.52 – 0.46 (m, 6H).

^{13}C NMR (126 MHz, Chloroform-*d*) δ 159.6, 133.5, 115.7, 113.8, 106.3, 90.7, 55.3, 26.5, 26.2, 13.8, 13.2.

HRMS-ESI (m/z): $[M+H]^+$ Calcd for $C_{21}H_{35}OSi$ 331.2452; Found: 331.2447.

4.3. Gram-Scale Experiment

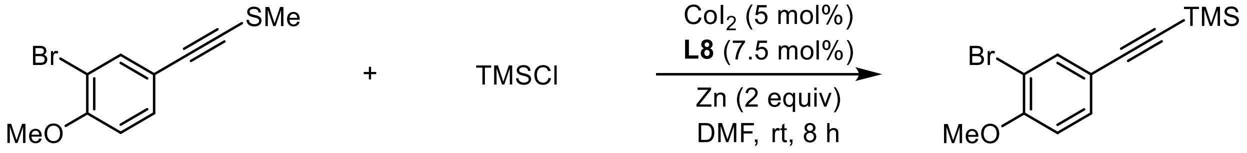

1ay, 6.0 mmol　　**2a**, 2.0 equiv　　**3ay**, 1.2 g, 71% yield

Prepared according to general procedure F using ((3-bromo-4-methoxyphenyl)ethynyl)(methyl)sulfane (1.54 g, 6 mmol), TMSCl (1.3 g, 12 mmol), CoI_2 (94.0 mg, 0.3 mmol), **L8** (105.0 mg, 0.45 mmol), Zn (0.79 g, 12 mmol), DMF (40.0 mL) at room temperature for 8 h. The crude material was purified by flash chromatography on silica gel to provide the title compound **3ay** as a white solid (1.2 g, 71% yield).

4.4. Product Derivatizations

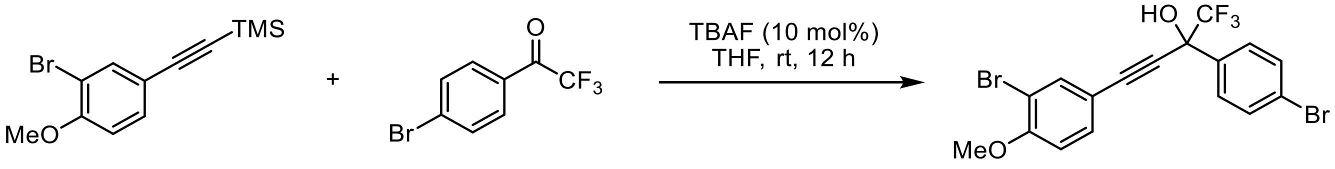

3ay, 0.25 mmol　　0.20 mmol　　**5a**, 64% yield

According to a literature procedure[26], to a solution of TBAF (10 mol % in dry THF) was added alkynylsilane **3ay** (0.25 mmol) at room temperature. To this was added 1-(4-bromophenyl)-2,2,2-

trifluoroethan-1-one (0.2 mmol) and then the reaction mixture was stirred at room temperature for 12 min. This was followed by the addition of 1 M aqueous HCl (1.0 mL) and further stirring for 15 min at room temperature. The reaction mixture was neutralized with aqueous $NaHCO_3$ and then it was extracted with ethyl acetate. The organic layers were collected and dried with anhydrous Na_2SO_4 followed by solvent removal under reduced pressure. The crude product was purified by flash chromatography on silica gel to give CF_3-substituted tertiary propargyl alcohols **5a** in 64% yield (59 mg) as a yellow oil.

^{1}H NMR (400 MHz, Chloroform-*d*) δ 7.72 – 7.63 (m, 3H), 7.58 – 7.53 (m, 2H), 7.43 (dd, *J* = 8.5, 2.0 Hz, 1H), 6.86 (d, *J* = 8.5 Hz, 1H), 3.92 (s, 3H), 3.34 (s, 1H).

^{13}C NMR (126 MHz, Chloroform-*d*) δ 157.1, 136.7, 134.3, 132.6, 131.4, 128.9, 124.0, 121.9 (q, *J* = 286.4 Hz), 114.0, 111.59, 111.55, 86.9, 83.6, 73.0 (q, *J* = 32.7 Hz), 56.4.

^{19}F NMR (376 MHz, Chloroform-*d*) δ -80.3.

HRMS-ESI (m/z): $[M+H]^+$ Calcd for $C_{17}H_{12}Br_2F_3O_2$ 462.9151; Found: 462.9149.

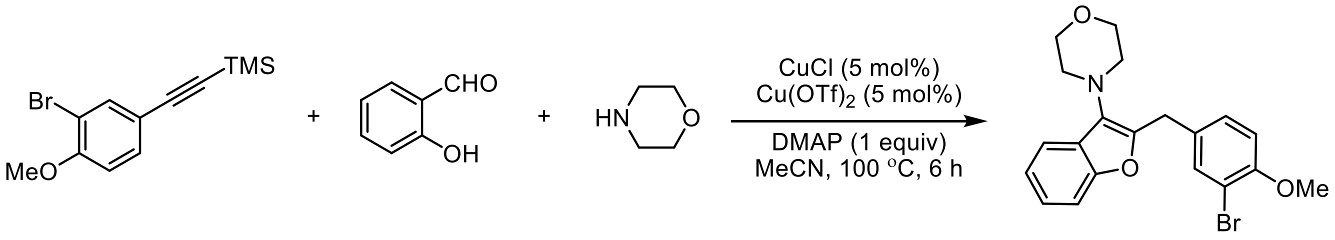

3ay, 0.25 mmol   0.30 mmol   0.20 mmol   **5b**, 76% yield

According to a literature procedure[27], to a CH_3CN solution (1 mL) in a screw-cap vial under N_2 atmosphere, alkynylsilane **3ay** (0.25 mmol), o-hydroxybenzaldehyde (0.3 mmol), secondary amine (0.20 mmol), DMAP (0.2 mmol), $Cu(OTf)_2$ (5 mol%), and CuCl (5 mol%) were successively added, and the vial was then sealed with a cap containing a PTFE septum. The reaction mixture was heated at 100 °C for 6 h. After completion of the reaction, the mixture was directly subjected to silica gel without the usual extraction, and was purified by flash column chromatography to give the corresponding 3-aminobenzofuran **5b** in 76% yield (61 mg) as a yellow oil.

^{1}H NMR (400 MHz, Chloroform-*d*) δ 7.72 – 7.64 (m, 2H), 7.48 (d, *J* = 1.7 Hz, 1H), 7.39 (d, *J* = 7.8 Hz, 1H), 7.25 – 7.14 (m, 5H), 6.82 (d, *J* = 8.4 Hz, 1H), 4.09 (s, 2H), 3.88 – 3.85 (m, 9H), 3.18 (t, 5H).

^{13}C NMR (101 MHz, Chloroform-*d*) δ 154.5, 153.4, 149.8, 133.3, 131.7, 128.7, 128.4, 125.9, 123.5, 122.1, 119.9, 111.9, 111.7, 111.6, 67.6, 56.2, 52.5, 31.0.

HRMS-ESI (m/z): $[M+H]^+$ Calcd for $C_{20}H_{21}BrNO_3$ 402.0699; Found: 402.0695.

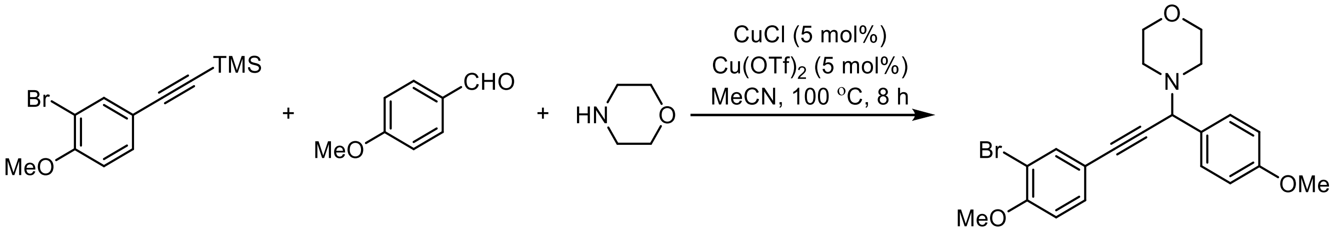

3ay, 0.24 mmol   0.20 mmol   0.24 mmol   **5c**, 84% yield

According to a literature procedure[28], to a MeCN solution (1 mL) in a screw-capped vial under an N_2 atmosphere, alkynylsilane **3ay** (0.24 mmol), aldehyde (0.20 mmol), amine (0.24 mmol), $Cu(OTf)_2$ (5 mol%) and CuCl (5 mol%) were successively added, and the vial was sealed with a cap containing a PTFE septum. The reaction mixture was heated at 100 °C until the reaction was completed as monitored by TLC. After the reaction, the mixture was directly subjected to SiO_2 gel without the usual extraction, and was purified by flash column chromatography (hexane–EtOAc) to give the corresponding propargyl amines **5c** in 84% yield (70mg) as a yellow oil.

^{1}H NMR (400 MHz, Chloroform-*d*) δ 7.70 (d, *J* = 2.0 Hz, 1H), 7.51 (d, *J* = 8.6 Hz, 2H), 7.44 – 7.39 (m, 1H), 6.94 – 6.87 (m, 2H), 6.83 (d, *J* = 8.5 Hz, 1H), 4.70 (s, 1H), 3.90 (s, 3H), 3.81 (s, 3H), 3.77 – 3.67 (m, 4H), 2.66 – 2.53 (m, 4H).

^{13}C NMR (101 MHz, Chloroform-*d*) δ 159.2, 155.9, 136.4, 132.1, 129.7, 129.6, 116.5, 113.5, 111.4, 111.3, 86.5, 85.0, 67.1, 61.4, 56.2, 55.2, 49.8.

HRMS-ESI (m/z): $[M+H]^+$ Calcd for $C_{21}H_{23}BrNO_3$ 416.0856; Found: 416.0847.

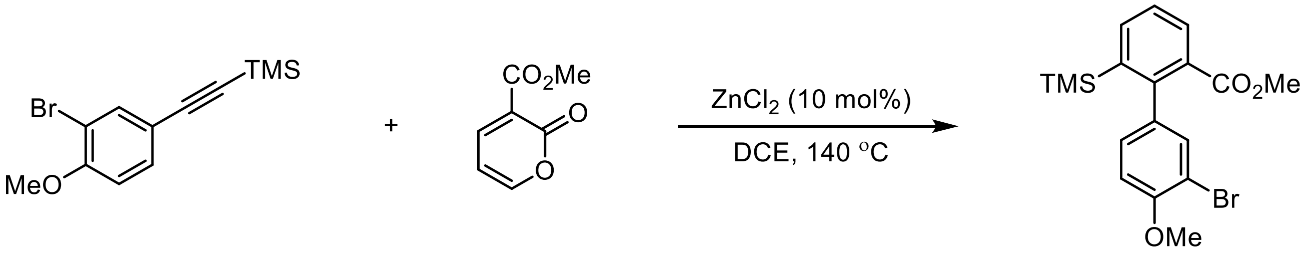

3ay, 0.20 mmol   0.40 mmol   **5d**, 73% yield

According to the modified literature procedure[29], a 10 mL sealed tube equipped with a magnetic stirring bar was charged with $ZnCl_2$ (10 mol%), alkynylsilane **3ay** (0.20 mmol, 1.0 equiv), 2-pyrone (0.40 mmol, 2.0 equiv) and DCE (1.0 mL). The reaction mixture was stirred at 140 °C for 12 h. The mixture was then filtered through silica gel pad. The filtrate was concentrated, and the residue was purified by column chromatography on silica gel to yield the desired product **5d** as a yellow oil in 73% yield (57 mg).

^{1}H NMR (400 MHz, Chloroform-*d*) δ 7.78 – 7.70 (m, 2H), 7.43 – 7.37 (m, 2H), 7.14 – 7.09 (m, 1H), 6.89 (d, *J* = 8.4 Hz, 1H), 3.94 (s, 3H), 3.59 (s, 3H), -0.01 (s, 9H).

^{13}C NMR (101 MHz, Chloroform-*d*) δ 168.8, 155.0, 146.1, 141.2, 137.4, 135.4, 134.4, 131.7, 129.6, 129.6, 126.7, 110.4, 110.3, 56.1, 51.9, 0.5.

HRMS-ESI (m/z): $[M+H]^+$ Calcd for $C_{18}H_{22}BrO_3Si$ 393.0516; Found: 393.0507.

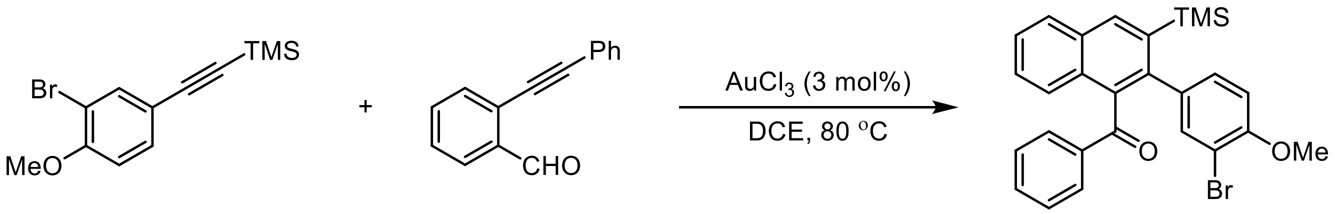

3ay, 0.20 mmol   0.40 mmol   **5e**, 49% yield

According to a literature procedure[30], a 10 mL sealed tube equipped with a magnetic stirring bar was charged with $AuCl_3$ (3 mol%), alkynylsilane **3ay** (0.20 mmol, 1.0 equiv), *o*-alkynylbenzaldehyde (0.40

mmol, 2.0 equiv) and DCE (1.0 mL). The reaction mixture was stirred at 80 °C for 12 h. The mixture was then filtered through silica gel pad. The filtrate was concentrated, and the residue was purified by column chromatography on silica gel to yield the desired product **5e** as a yellow oil in 49% yield (48 mg).

^{1}H NMR (400 MHz, Chloroform-*d*) δ 8.20 (s, 1H), 7.95 (d, *J* = 8.1 Hz, 1H), 7.72 – 7.38 (m, 7H), 7.29 (t, *J* = 7.7 Hz, 2H), 7.22 – 7.05 (m, 1H), 6.87 – 6.59 (m, 1H), 3.83 (s, 3H), 0.08 (s, 9H).

^{13}C NMR (101 MHz, Chloroform-*d*) δ 199.9, 155.0, 140.1, 138.2, 137.9, 136.9, 136.7, 135.5, 133.9, 133.2, 131.6, 130.3, 129.3, 128.8, 128.3, 128.3, 127.7, 126.4, 125.2, 110.5, 110.0, 56.1, 0.6.

HRMS-ESI (m/z): $[M+H]^+$ Calcd for $C_{27}H_{26}BrO_2Si$ 489.0880; Found: 489.0869.

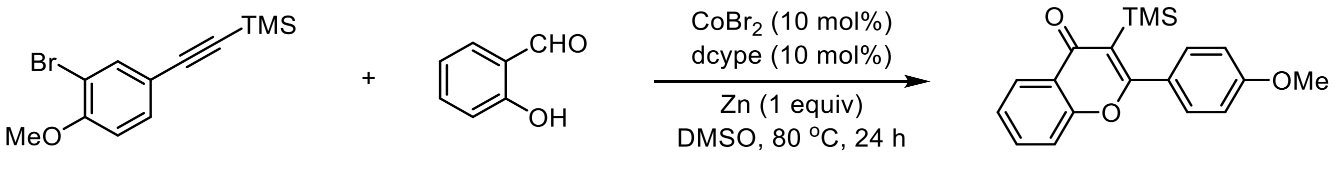

3ay, 0.20 mmol 0.60 mmol **5f**, 69% yield

According to a literature procedure[31], in an argon-filled glove box, a 4-mL vial equipped with a magnetic stirrer bar was charged sequentially with $CoBr_2$ (10 mol%), dcype (10 mol%), zinc powder (1 equiv), salicylaldehyde (0.60 mmol), and alkynylsilane **3ay** (0.20 mmol), followed by the addition of DMSO (0.5 mL). The vial was closed and removed from the glove box, and the mixture was stirred at 80 °C for 24 h. Upon cooling to room temperature, the reaction mixture was diluted with ethyl acetate (3 mL) and filtered through a pad of silica gel with additional ethyl acetate (10 mL) as an eluent. The organic solution was concentrated under reduced pressure, and the residue was purified by flash chromatography on silica gel to afford the desired product **5f** in 69% yield (45 mg) as a white solid.

^{1}H NMR (400 MHz, Chloroform-*d*) δ 8.16 (dd, *J* = 7.9, 1.5 Hz, 1H), 7.65 – 7.59 (m, 1H), 7.49 – 7.44 (m, 2H), 7.39 – 7.34 (m, 2H), 7.01 – 6.96 (m, 2H), 3.88 (s, 3H), 0.06 (s, 9H).

^{13}C NMR (101 MHz, Chloroform-*d*) δ 182.5, 169.1, 161.3, 156.2, 133.4, 130.8, 128.2, 125.6, 124.8, 122.8, 118.0, 117.6, 113.5, 55.4, -0.6.

Spectroscopic data for **5f** match those previously reported in the literature.[31]

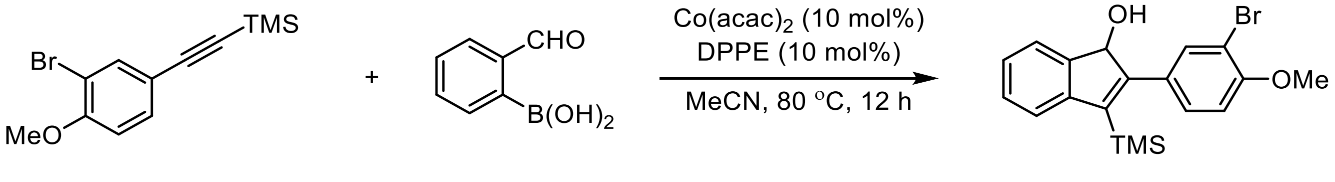

3ay, 0.20 mmol 0.30 mmol **5g**, 81% yield

According to a literature procedure[32], a 5 mL sealed tube equipped with a magnetic stirring bar was charged with $Co(acac)_2$ (10 mol%), DPPE (10 mol%), *ortho*-formylphenylboronic acid (0.30 mmol), alkynylsilane **3ay** (0.20 mmol, 1.0 equiv), and MeCN (1 mL). The reaction mixture was stirred at 80 °C for 12 h. The mixture was then filtered through silica gel pad. The filtrate was concentrated, and the residue was purified by column chromatography on silica gel to yield the desired product **5g** as a yellow oil in 81% yield (63 mg).

^{1}H NMR (400 MHz, Chloroform-*d*) δ 7.59 – 7.52 (m, 2H), 7.45 (d, *J* = 7.5 Hz, 1H), 7.37 – 7.31 (m, 1H), 7.29 – 7.22 (m, 2H), 6.95 (d, *J* = 8.4 Hz, 1H), 5.31 (s, 1H), 3.96 (s, 3H), 0.19 (s, 9H).
^{13}C NMR (101 MHz, Chloroform-*d*) δ 159.2, 155.6, 146.2, 144.6, 139.5, 134.0, 130.8, 129.4, 128.5, 125.5, 123.6, 122.7, 111.2, 111.2, 80.4, 56.2, 0.3.
HRMS-ESI (m/z): $[M-H]^+$ Calcd for $C_{19}H_{20}BrO_2Si$ 387.0421; Found: 387.0412.

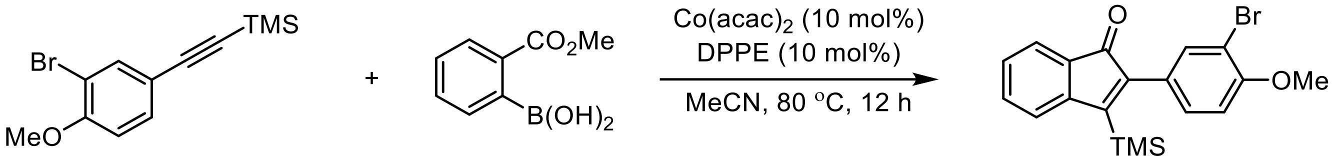

3ay, 0.20 mmol 0.30 mmol **5h**, 65% yield

According to a literature procedure[33], a 5 mL sealed tube equipped with a magnetic stirring bar was charged with $Co(acac)_2$ (10 mol%), DPPE (10 mol%), alkynylsilane **3ay** (0.20 mmol, 1.0 equiv), *o*-methoxyphenylboronic acid (0.30 mmol, 1.5 equiv), and MeCN (1.0 mL). The reaction mixture was stirred at 80 °C for 12 h. The mixture was then filtered through silica gel pad. The filtrate was concentrated, and the residue was purified by column chromatography on silica gel to yield the desired product **5h** as a yellow oil in 65% yield (50 mg).
^{1}H NMR (400 MHz, Chloroform-*d*) δ 7.50 (d, *J* = 7.1 Hz, 1H), 7.46 (d, *J* = 2.1 Hz, 1H), 7.44 – 7.37 (m, 1H), 7.28 – 7.19 (m, 3H), 6.95 (d, *J* = 8.4 Hz, 1H), 3.95 (s, 3H), 0.23 (s, 9H).
^{13}C NMR (101 MHz, Chloroform-*d*) δ 197.5, 156.9, 155.9, 148.5, 145.9, 134.6, 134.1, 130.0, 129.8, 128.1, 126.8, 123.2, 123.2, 111.2, 111.0, 56.2, 0.2.
HRMS-ESI (m/z): $[M+H]^+$ Calcd for $C_{19}H_{20}BrO_2Si$ 387.0410; Found: 387.0404.

4.5. Synthesis of bioactive molecule: 6-fluoroflavone

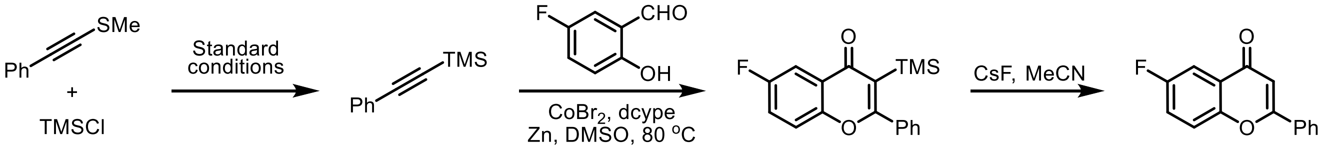

3bf, 83% **3bf-1,** 73% **6-fluoroflavone,** 88%

3bf was prepared according to general procedure F using methyl(phenylethynyl)sulfane (0.74 g, 5 mmol), TMSCl (1.08 g, 10 mmol), CoI_2 (156.0 mg, 0.5 mmol), **L8** (174.0 mg, 0.75 mmol), Zn (0.65 g, 10 mmol), DMF (25.0 mL) at room temperature for 4 h. The crude material was purified by flash chromatography on silica gel to provide the title compound **3bf** as a colorless oil (0.72 g, 83% yield). Characterization data matched those reported in the literature.[34]
^{1}H NMR (400 MHz, Chloroform-*d*) δ 7.51 – 7.46 (m, 2H), 7.34 – 7.28 (m, 3H), 0.27 (s, 9H).
^{13}C NMR (126 MHz, Chloroform-*d*) δ 131.9, 128.5, 128.2, 123.0, 105.1, 94.0, -0.0.

3bf-1 was synthesized following the known procedure. In an argon-filled glove box, a 25-mL vial equipped with a magnetic stirrer bar was charged sequentially with $CoBr_2$ (10 mol%), dcype (10 mol%),

zinc powder (1 equiv), 5-fluoro-2-hydroxybenzaldehyde (3 equiv), and alkynylsilane **3bf** (3.0 mmol), followed by the addition of DMSO (9.0 mL). The vial was closed and removed from the glove box, and the mixture was stirred at 80 °C for 24 h. Upon cooling to room temperature, the reaction mixture was quenched with water (20.0 mL) and extracted with ethyl acetate (3 × 15.0 mL). The combined organic layers were washed with water, brine, dried over anhydrous Na_2SO_4, and concentrated under reduced pressure. The residue was purified by flash chromatography on silica gel to afford the desired product **3bf-1** in 73% yield (0.68 g) as a white solid.

^{1}H NMR (400 MHz, Chloroform-*d*) δ 7.85 – 7.77 (m, 1H), 7.56 – 7.46 (m, 5H), 7.42 – 7.33 (m, 2H), 0.03 (s, 9H).

^{13}C NMR (126 MHz, Chloroform-*d*) δ 181.6 (d, *J* = 2.0 Hz), 169.4, 159.4 (d, *J* = 246.2 Hz), 152.4 (d, *J* = 1.3 Hz), 135.4, 130.5, 129.1, 128.2, 123.9 (d, *J* = 7.0 Hz), 121.6 (d, *J* = 25.4 Hz), 119.8 (d, *J* = 7.9 Hz), 117.7, 110.4 (d, *J* = 23.3 Hz), 0.3.

^{19}F NMR (376 MHz, Chloroform-*d*) δ -115.5.

Melting point: 95-96 °C

HRMS-ESI (m/z): $[M+H]^+$ Calcd for $C_{18}H_{18}FO_2Si$ 313.1055; Found: 313.1048.

6-fluoroflavone was synthesized following the known procedure. An oven-dried sealed tube (38 mL) containing **3bf-1** (0.2 mmol, 62.4 mg, 1.0 equiv), CsF (0.40 mmol, 61 mg, 2.0 equiv), and CH_3CN (2 mL) was evacuated and purged with argon gas three times. The reaction mixture was stirred at 50 °C for 24 h. Upon completion of the reaction, the solvent was removed under vacuo and the residue was purified with chromatography column on silica gel to give the product **6-fluoroflavone** (42 mg, 88% yield) as a white solid. Characterization data matched those reported in the literature.[35]

^{1}H NMR (400 MHz, Chloroform-*d*) δ 7.94 – 7.88 (m, 2H), 7.88 – 7.83 (m, 1H), 7.59 – 7.50 (m, 4H), 7.45 – 7.38 (m, 1H), 6.81 (s, 1H).

^{13}C NMR (101 MHz, Chloroform-*d*) δ 177.6 (d, *J* = 2.2 Hz), 163.7, 159.6 (d, *J* = 246.9 Hz), 152.4 (d, *J* = 1.8 Hz), 131.8, 131.5, 129.1, 126.3, 125.1 (d, *J* = 7.4 Hz), 121.9 (d, *J* = 25.5 Hz), 120.1 (d, *J* = 8.1 Hz), 110.6 (d, *J* = 23.6 Hz), 106.8.

^{19}F NMR (376 MHz, Chloroform-*d*) δ -115.0 – -115.1 (m).

4.6. Extended to synthesis of alkynylgermanes

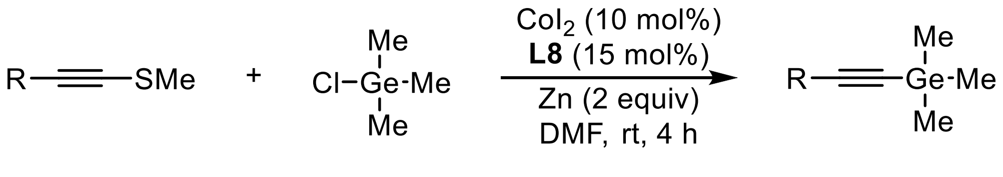

1, 0.2 mmol 2.0 equiv **6**

The procedure was conducted in a nitrogen-filled glove box. To a reaction vial equipped with a magnetic stir bar was added CoI_2 (6.3 mg, 0.02 mmol), **L8** (7.0 mg, 0.03 mmol), Zn (26.2 mg, 2 equiv). A solution of **1** (0.2 mmol) and chlorogermanes (0.4 mmol) in DMF (2.0 mL) was added. The reaction vial was

sealed and removed from the glove box. The mixture was stirred at room temperature for 4 h, subsequently quenched with water (10.0 mL) and extracted with ethyl acetate (3 × 15.0 mL). The combined organic layers were washed with water, brine, dried over anhydrous Na_2SO_4, and concentrated under reduced pressure. The residue was purified by flash chromatography on silica gel to afford product.

((4-methoxyphenyl)ethynyl)trimethylgermane

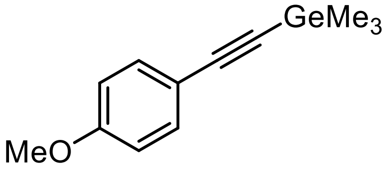

Prepared according to general procedure using ((4-methoxyphenyl)ethynyl)(methyl)sulfane (35.6 mg, 0.2 mmol), Me_3GeCl (61.2 mg, 0.4 mmol), CoI_2 (6.3 mg, 0.02 mmol), **L8** (7.0 mg, 0.03 mmol), Zn (26.2 mg, 0.4 mmol), DMF (2.0 mL) at room temperature for 4 h. The crude material was purified by flash chromatography on silica gel to provide the title compound **6a** as a yellow oil (46.0 mg, 92% yield)**.**

^{1}H NMR (400 MHz, Chloroform-*d*) δ 7.42 – 7.37 (m, 2H), 6.83 – 6.79 (m, 2H), 3.79 (s, 3H), 0.42 (s, 9H).

^{13}C NMR (101 MHz, Chloroform-*d*) δ 159.4, 133.3, 115.6, 113.7, 104.2, 92.7, 55.2, -0.1.

HRMS-ESI (m/z): $[M+H]^+$ Calcd for $C_{12}H_{17}GeO$ 247.0516; Found: 247.0514.

methyl 4-((trimethylgermyl)ethynyl)benzoate

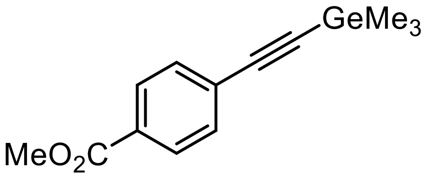

Prepared according to general procedure using methyl 4-((methylthio)ethynyl)benzoate (41.2 mg, 0.2 mmol), Me_3GeCl (61.2 mg, 0.4 mmol), CoI_2 (6.3 mg, 0.02 mmol), **L8** (7.0 mg, 0.03 mmol), Zn (26.2 mg, 0.4 mmol), DMF (2.0 mL) at room temperature for 4 h. The crude material was purified by flash chromatography on silica gel to provide the title compound **6b** as a colorless oil (35.0 mg, 63% yield).

^{1}H NMR (400 MHz, Chloroform-*d*) δ 7.97 – 7.93 (m, 2H), 7.52 – 7.47 (m, 2H), 3.90 (s, 3H), 0.43 (s, 9H).

^{13}C NMR (101 MHz, Chloroform-*d*) δ 166.5, 131.8, 129.3, 128.1, 103.3, 98.5, 52.2, -0.3.

HRMS-ESI (m/z): $[M+H]^+$ Calcd for $C_{13}H_{17}GeO_2$ 275.0466; Found: 275.0459.

((4-bromophenyl)ethynyl)trimethylgermane

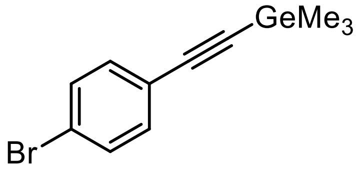

Prepared according to general procedure using ((4-bromophenyl)ethynyl)(methyl)sulfane (45.4 mg, 0.2 mmol), Me_3GeCl (61.2 mg, 0.4 mmol), CoI_2 (6.3 mg, 0.02 mmol), **L8** (7.0 mg, 0.03 mmol), Zn (26.2 mg,

0.4 mmol), DMF (2.0 mL) at room temperature for 4 h. The crude material was purified by flash chromatography on silica gel to provide the title compound **6c** as a colorless oil (53.0 mg, 89% yield)**.**

^{1}H NMR (400 MHz, Chloroform-*d*) δ 7.45 – 7.38 (m, 2H), 7.34 – 7.28 (m, 2H), 0.43 (s, 9H).

^{13}C NMR (101 MHz, Chloroform-*d*) δ 133.3, 131.4, 122.4, 122.3, 103.0, 96.2, -0.2.

HRMS-ESI (m/z): $[M+H]^+$ Calcd for $C_{11}H_{14}BrGe$ 294.9516; Found: 294.9508.

((2-bromophenyl)ethynyl)trimethylgermane

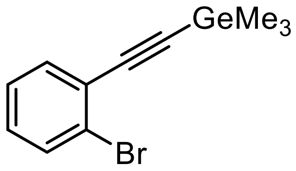

Prepared according to general procedure using ((2-bromophenyl)ethynyl)(methyl)sulfane (45.4 mg, 0.2 mmol), Me_3GeCl (61.2 mg, 0.4 mmol), CoI_2 (6.3 mg, 0.02 mmol), **L8** (7.0 mg, 0.03 mmol), Zn (26.2 mg, 0.4 mmol), DMF (2.0 mL) at room temperature for 4 h. The crude material was purified by flash chromatography on silica gel to provide the title compound **6d** as a colorless oil (48.0 mg, 81% yield)**.**

^{1}H NMR (400 MHz, Chloroform-*d*) δ 7.58 – 7.54 (m, 1H), 7.50 – 7.46 (m, 1H), 7.25 – 7.20 (m, 1H), 7.16 – 7.11 (m, 1H), 0.46 (s, 9H).

^{13}C NMR (101 MHz, Chloroform-*d*) δ 133.5, 132.3, 129.2, 126.8, 125.6, 125.5, 102.2, 100.4, -0.2.

HRMS-ESI (m/z): $[M+H]^+$ Calcd for $C_{11}H_{14}BrGe$ 294.9516; Found: 294.9509.

5. Mechanistic studies

5.1. Radical trapping experiments

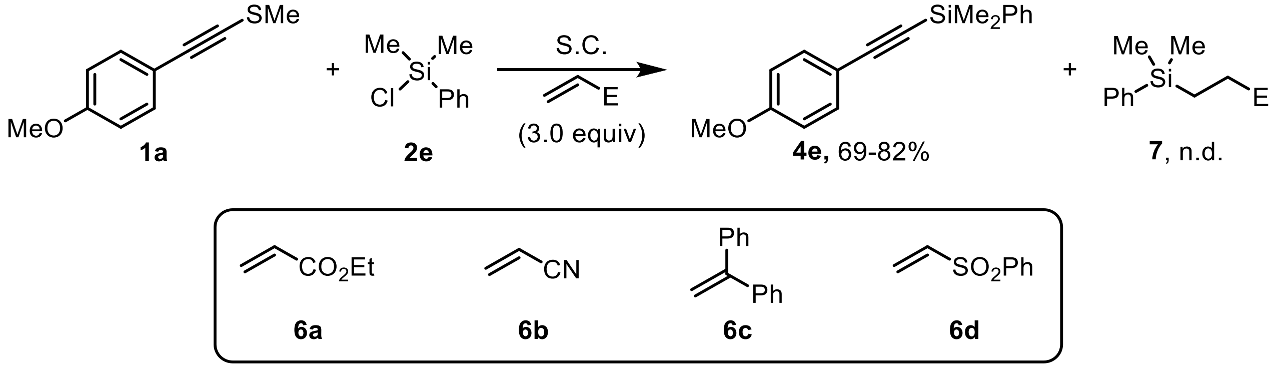

General Procedure. The procedure was conducted in an argon-filled glove box. To a reaction tube equipped with a magnetic stir bar was added CoI_2 (3.2 mg, 0.01 mmol), **L8** (3.5 mg, 0.015 mmol), and Zn (13 mg, 0.2 mmol). A solution of alkynyl sulfide **1a** (17.8 mg, 0.1 mmol), chlorosilane **2e** (34.0 mg, 0.2 mmol), and alkene **6** (0.3 mmol, 3 equiv) in DMF (1.0 mL) was added. The reaction tube was sealed and removed from the glove box. After stirring at room temperature for 4 h, the reaction mixture was diluted with ethyl acetate (5 mL) and washed with water. A 0.2 mL of solution was collected, diluted with ethyl acetate (1 mL), and used for GC analysis.

5.2. Radical clock experiment

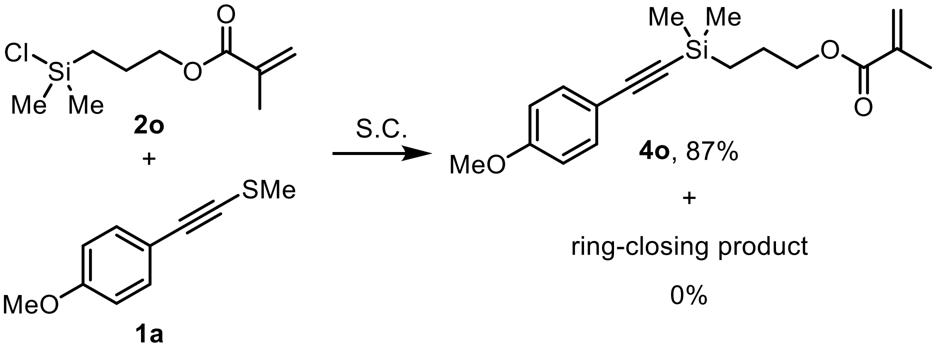

General Procedure. The procedure was conducted in an argon-filled glove box. To a reaction tube equipped with a magnetic stir bar was added CoI_2 (6.3 mg, 0.02 mmol), **L8** (7.0 mg, 0.03 mmol), and Zn (26.2 mg, 0.4 mmol). A solution of alkynyl sulfide **1a** (35.6 mg, 0.2 mmol) and chlorosilane **2o** (88.0 mg, 0.4 mmol) in DMF (2.0 mL) was added. The reaction tube was sealed and removed from the glove box. After stirring at room temperature for 4 h, the reaction was quenched with water (20 mL) and the mixture solution was extracted with ethyl acetate (3 × 15 mL). The combined organic layers were washed with water, brine, dried over anhydrous Na_2SO_4. A 0.4 mL of solution was collected, diluted with ethyl acetate (1 mL), and used for GC analysis. The rest was concentrated in vacuum, and the residue was purified by flash chromatography on silica gel to afford product **4o** (55.0 mg, 87% yield) as a yellow oil.

3-(((4-methoxyphenyl)ethynyl)dimethylsilyl)propyl methacrylate (4o)

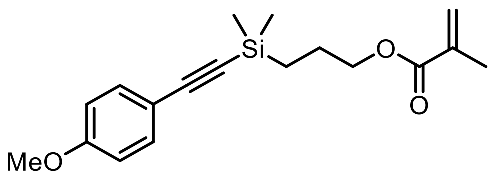

^{1}**H NMR** (400 MHz, Chloroform-*d*) δ 7.46 – 7.34 (m, 2H), 6.88 – 6.77 (m, 2H), 6.11 (s, 1H), 5.58 – 5.51 (m, 1H), 4.16 (t, *J* = 6.9 Hz, 2H), 3.80 (s, 3H), 1.95 (s, 3H), 1.87 – 1.76 (m, 2H), 0.78 – 0.67 (m, 2H), 0.23 (s, 6H).

^{13}**C NMR** (126 MHz, Chloroform-*d*) δ 167.5, 159.8, 136.5, 133.5, 125.1, 115.1, 113.8, 106.1, 90.9, 66.9, 55.2, 23.3, 18.3, 12.3, -1.7.

HRMS-ESI (m/z): $[M+H]^+$ Calcd for $C_{18}H_{25}O_3Si$ 317.1567; Found: 317.1566.

5.3. Control experiments

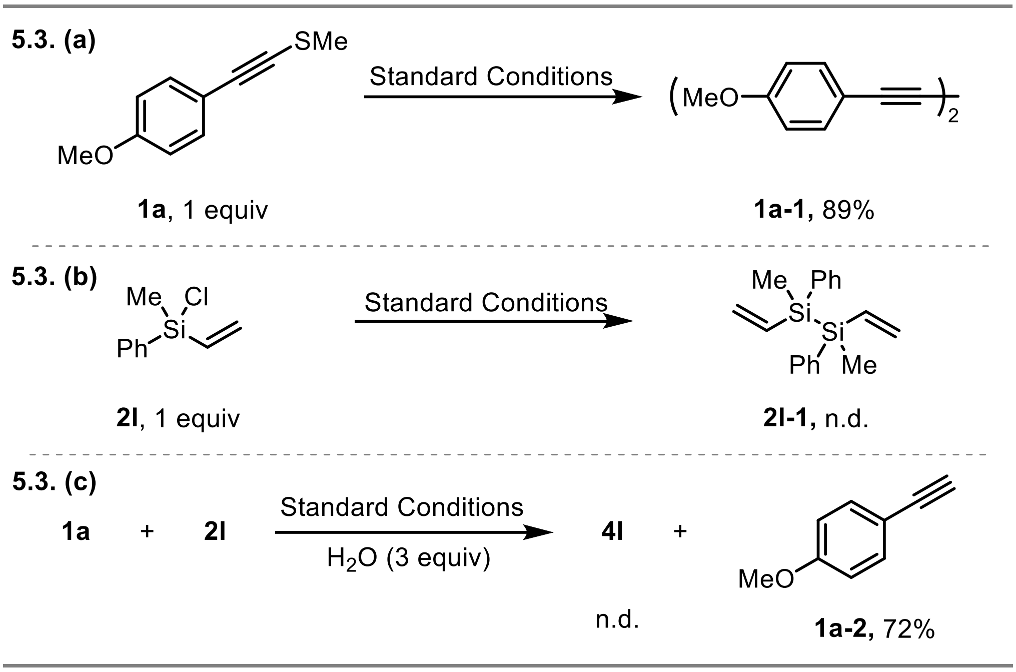

General Procedure 5.3. (a). The procedure was conducted in an argon-filled glove box. To a reaction tube equipped with a magnetic stir bar was added CoI_2 (3.2 mg, 0.01 mmol), **L8** (3.5 mg, 0.015 mmol), and Zn (13.1 mg, 0.2 mmol). A solution of alkynyl sulfide **1a** (17.8 mg, 0.1 mmol) in DMF (1.0 mL) was added. The reaction tube was sealed and removed from the glove box. After stirring at room temperature for 4 h, the reaction was quenched with water (10 mL) and the mixture solution was extracted with ethyl acetate (3 × 10 mL). The combined organic layers were washed with water, brine, dried over anhydrous Na_2SO_4. A 0.3 mL of solution was collected, diluted with ethyl acetate (1 mL). The yields were determined by GC-MS analysis with dodecane as an internal standard.

General Procedure 5.3. (b). The procedure was conducted in an argon-filled glove box. To a reaction tube equipped with a magnetic stir bar was added CoI_2 (3.2 mg, 0.01 mmol), **L8** (3.5 mg, 0.015 mmol), and Zn (13.1 mg, 0.2 mmol). A solution of chlorosilane **2l** (18.3 mg, 0.1 mmol) in DMF (1.0 mL) was added. The reaction tube was sealed and removed from the glove box. After stirring at room temperature

for 4 h, the reaction was quenched with water (10 mL) and the mixture solution was extracted with ethyl acetate (3 × 10 mL). The combined organic layers were washed with water, brine, dried over anhydrous Na_2SO_4. A 0.3 mL of solution was collected, diluted with ethyl acetate (1 mL), and used for GC-MS analysis.

General Procedure 5.3. (c). The procedure was conducted in an argon-filled glove box. To a reaction tube equipped with a magnetic stir bar was added CoI_2 (3.2 mg, 0.01 mmol), **L8** (3.5 mg, 0.015 mmol), and Zn (13.1 mg, 0.2 mmol). A solution of alkynyl sulfide **1a** (17.8 mg, 0.1 mmol), chlorosilane **2l** (36.6 mg, 0.2 mmol) and H_2O (5.4 mg, 0.3 mmol) in DMF (1.0 mL) was added. The reaction tube was sealed and removed from the glove box. After stirring at room temperature for 4 h, the reaction was quenched with water (10 mL) and the mixture solution was extracted with ethyl acetate (3 × 10 mL). The combined organic layers were washed with water, brine, dried over anhydrous Na_2SO_4. A 0.4 mL of solution was collected, diluted with ethyl acetate (1 mL), and used for GC-MS analysis.

5.4. Study on the possibility of an alkynylzinc Intermediate

Quenching experiments were perfomed using Knochel's Method.[36]

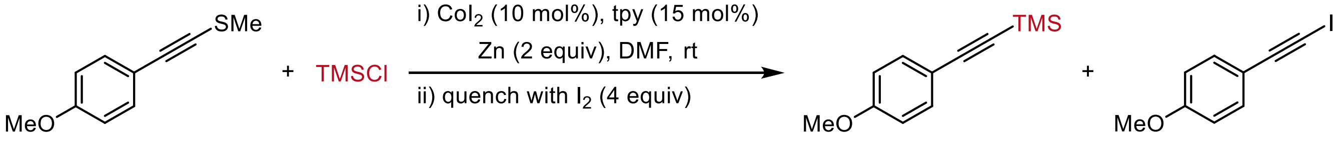

1a, 0.5 mmol   2 equiv   **3a**   **1a-3**

To an oven-dried scintillation vial fitted with a Teflon-coated stir-bar was added LiCl (3.0 mmol, 127.2 mg). The vial was capped with a rubber septa, flushed with argon, and THF (6 mL) was added via syringe. The solution was stirred at rt until all solids dissolved (approximately 4 h) resulting in a 0.5 M solution of LiCl. In a separate scintillation vial was weighted I_2 (2.0 mmol, 508 mg). The vial was capped with a rubber septa, flushed with N_2, and 4 mL of the 0.5 M solution of LiCl was added via syringe. The resulting brown solution was stirred at rt for 1 h. This iodine solution (0.1 mmol, 200 μL) was used to quench the catalytic reaction (0.025 mmol, 250 μL) at 15, 30, 45, 60, 80, and 100 minutes.

For convenience, the following reaction was set up in an argon-filled glove box. To a reaction tube equipped with a magnetic stir bar was added CoI_2 (16.0 mg, 0.05 mmol), **L8** (17.5 mg, 0.075 mmol), and Zn (65.5 mg, 1.0 mmol). A solution of alkynyl sulfide **1a** (89.0 mg, 0.5 mmol) and TMSCl (108.6 mg, 1.0 mmol) in DMF (5.0 mL) was added and stirred at rt. At 15, 30, 45, 60, 80, and 100 minutes, an aliquot of the catalytic reaction (0.025 mmol, 250 μL) was removed and added to a 1-dram vial containing iodine solution (0.1 mmol, 200 μL) and dodecane (0.025 mmol) as the internal standard. The resulting mixture was diluted with diethyl ether (0.50 mL), mixed, and filtered through a 2-cm silica plug in a Pasteur pipette directly into a GC vial. Samples were analyzed by GC.

Supplementary Figure 1. Kinetics of the reaction at 15, 30, 45, 60, 80, and 100 minutes after quenching with I_2

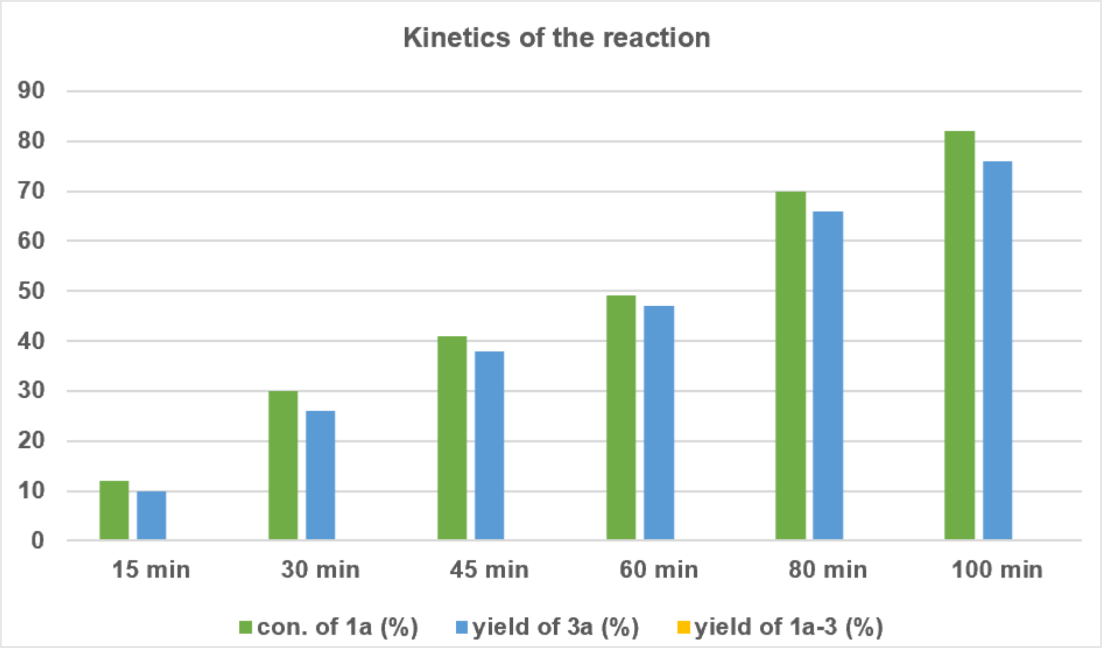

5.5. Cyclic voltammograms of Co^{II}(tpy) and *in-situ* generated Co^{I}(tpy) in DMF

The working electrode was a 3-mm diameter glassy carbon electrode disk carefully polished and ultrasonically rinsed in absolute ethanol before use. The electrode was then ultrasonically rinsed in absolute ethanol and dried over a stream of compressed air before use. The counter electrode was a carbon rod and the reference electrode was an Ag/AgCl electrode. All experiments were carried out under nitrogen atmosphere. Cyclic voltammograms were obtained with a CORRTEST-CS310M instrument. Experiments were made in DMF purchased from Energy Chemical, 99.8%, Extra Dry, EnergySeal and used without purification and using a 100 mM solution of tetrabutylammonium tetrafluoroborate in DMF as supporting electrolyte.

Procedure of preparation of A: In the glovebox, CoI_2 (15.6 mg, 0.05 mmol, 1.0 equiv.) and tpy (12.8 mg, 0.055 mmol, 1.1 equiv.) were added to a 20-mL vial equipped with a stirring bar and 8 mL of DMF was added. The vial was taken out of the glovebox and the mixture was allowed to stir for 6 h at room temperature. Then, the deep red solution was performed cyclic voltammetry experiments.

Procedure of preparation of B: In the glovebox, CoI_2 (15.6 mg, 0.05 mmol, 1.0 equiv.) and tpy (12.8 mg, 0.055 mmol, 1.1 equiv.) were added to a 20-mL vial equipped with a stirring bar and 8 mL of DMF was added. The vial was taken out of the glovebox and the mixture was allowed to stir for 6 h at room temperature. Then, the vial was taken back into the glovebox and Zn (49.0 mg, 15.0 equiv) was added to the deep red solution. Upon stirring, the color of solution turn from deep red to black in 20 min. After

12 h, the black supernatant characteristic of reduced cobalt Co^{I}(tpy) was filtered off remaining Zn to another 20-mL vial to perform cyclic voltammetry experiments.

Supplementary Figure 2. Reaction mixtures of cobalt complexes and cobalt intermediates

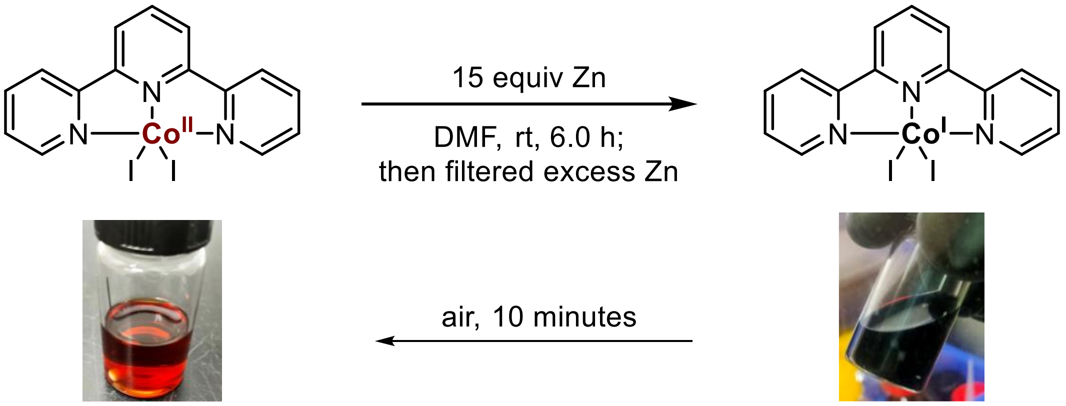

A) Co^{II}(tpy) **B) Co^{I}(tpy)**

Attention: **Co^{I}(tpy)** in DMF(black solution) generated *in-situ* is very unstable and easily oxidized by air to **Co^{II}(tpy)** (dark red solution).

Supplementary Figure 3. Cyclic voltammograms of A and B in DMF

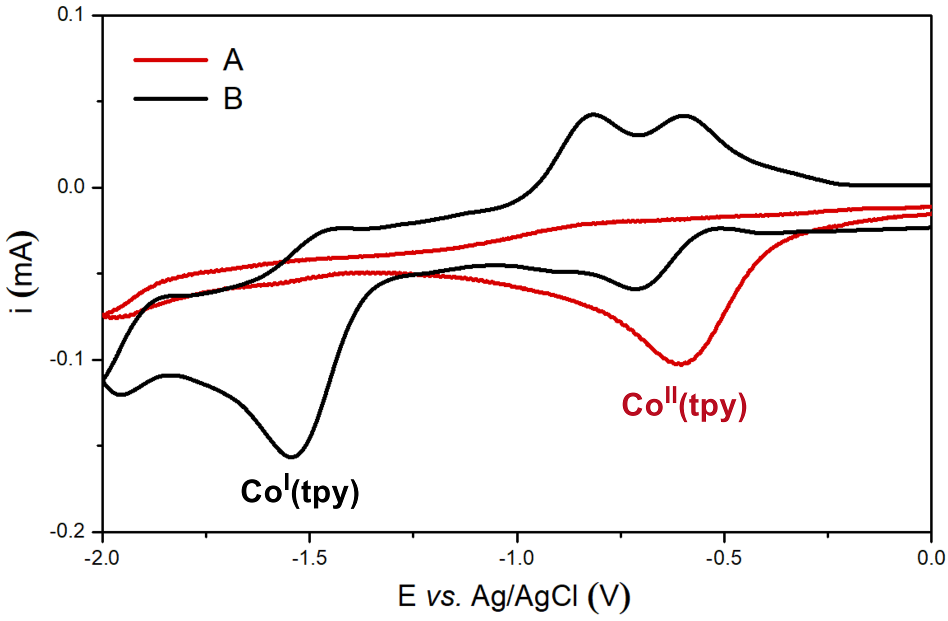

5.6. Study on the reactivity of the *in-situ-formed* Co^{I}(tpy) towards alkynyl sulfide

5.6.1. Stoichiometric reaction of *in-situ-formed* Co^{I}(tpy) with alkynyl sulfide

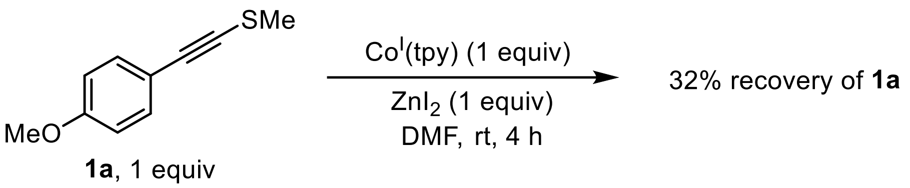

The procedure was conducted in an argon-filled glove box. To a reaction tube equipped with a magnetic stir bar was added *in-situ-formed* Co^{I}(tpy) (0.05 M, 1 mL) and alkynyl sulfide **1a** (9.0 mg, 0.05 mmol). The reaction tube was sealed and removed from the glove box. After stirring at room temperature for 4 h, the reaction was quenched with water (10 mL) and the mixture solution was extracted with ethyl acetate (3 × 10 mL). The combined organic layers were washed with water, brine, dried over anhydrous Na_2SO_4. A 0.3 mL of solution was collected, diluted with ethyl acetate (1 mL), and used for GC-MS analysis.

5.6.2. Using the *in-situ-formed* Co^{I}(tpy) to catalyze the reaction

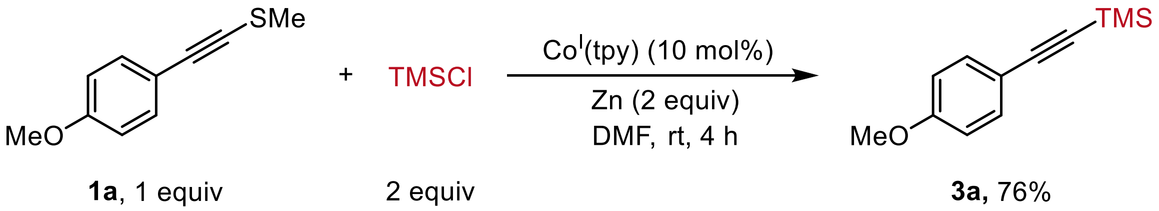

The procedure was conducted in an argon-filled glove box. To a reaction tube equipped with a magnetic stir bar was added the *in-situ-formed* Co^{I}(tpy) (0.05 M, 0.2 mL, 10 mol%). A solution of alkynyl sulfide **1a** (17.8 mg, 0.1 mmol) and TMSCl (21.7 mg, 0.2 mmol) in DMF (0.8 mL) was added. The reaction tube was sealed and removed from the glove box. After stirring at room temperature for 4 h, the reaction was quenched with water (10 mL) and the mixture solution was extracted with ethyl acetate (3 × 10 mL). The combined organic layers were washed with water, brine, dried over anhydrous Na_2SO_4. A 0.3 mL of solution was collected, diluted with ethyl acetate (1 mL), and used for GC-MS analysis.

6. NMR Spectra

1b; ^{1}H NMR (400 MHz, $CDCl_3$); ^{13}C NMR (101 MHz, $CDCl_3$)

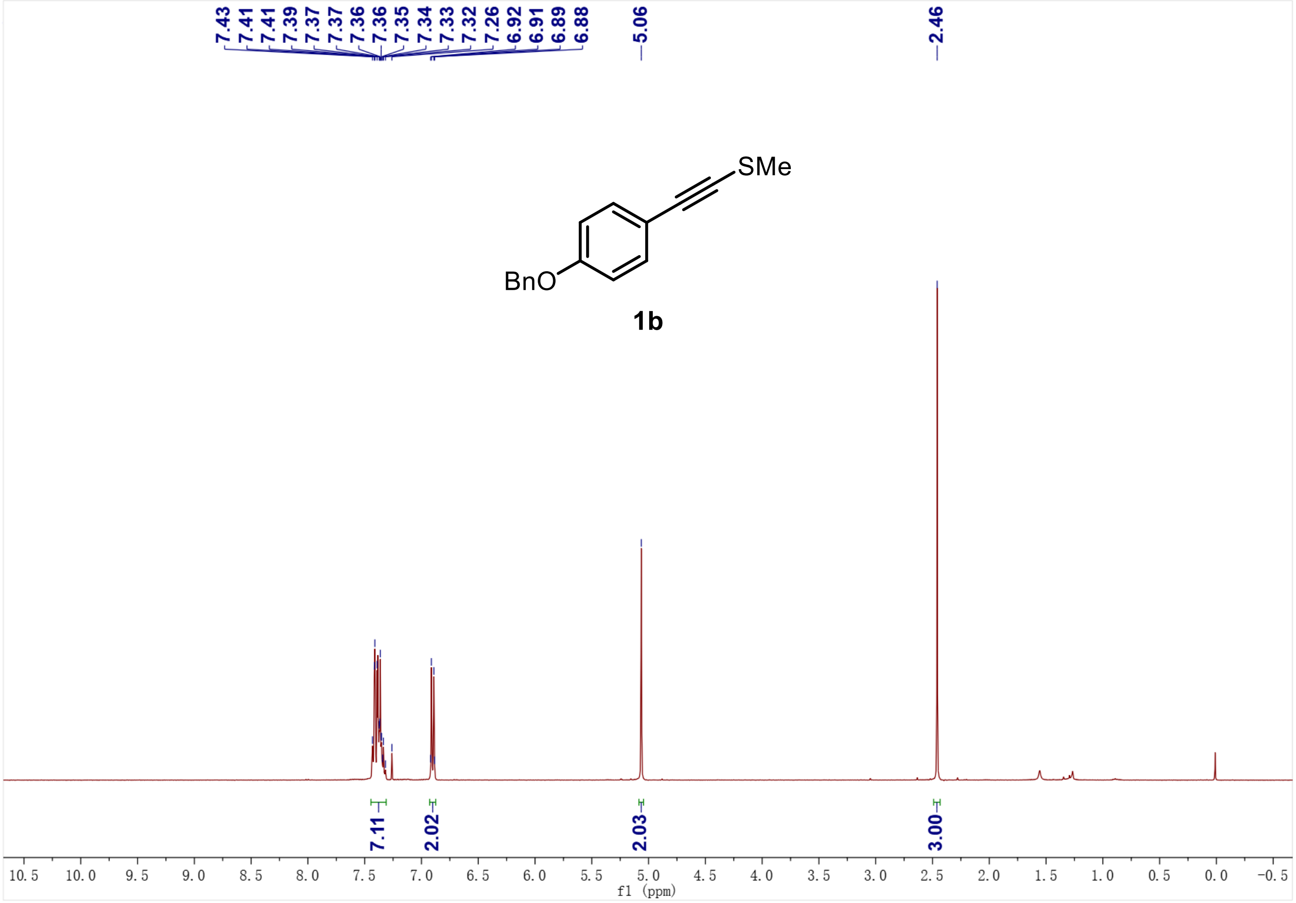

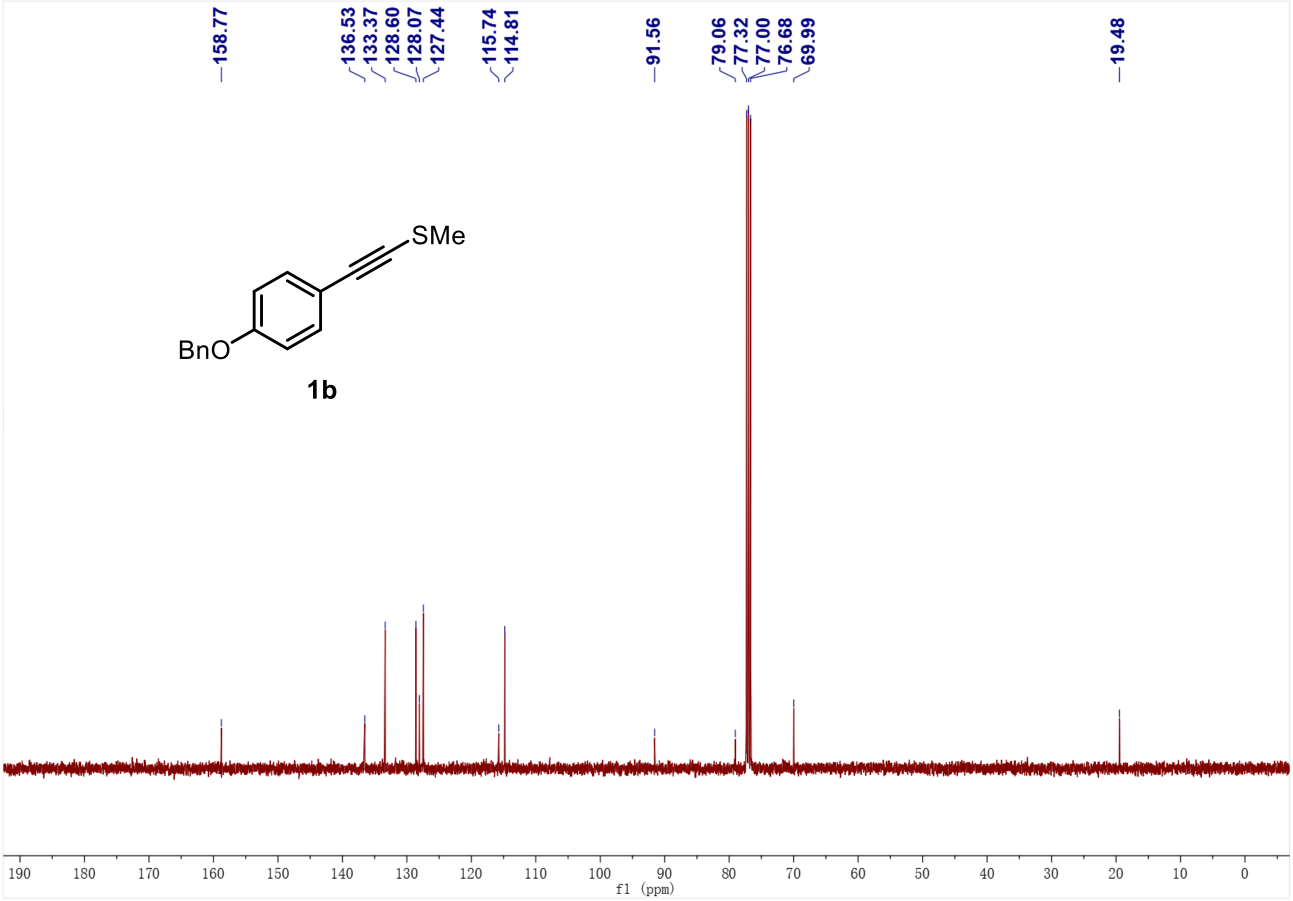

1f; ^{1}H NMR (500 MHz, $CDCl_3$); ^{13}C NMR (126 MHz, $CDCl_3$)

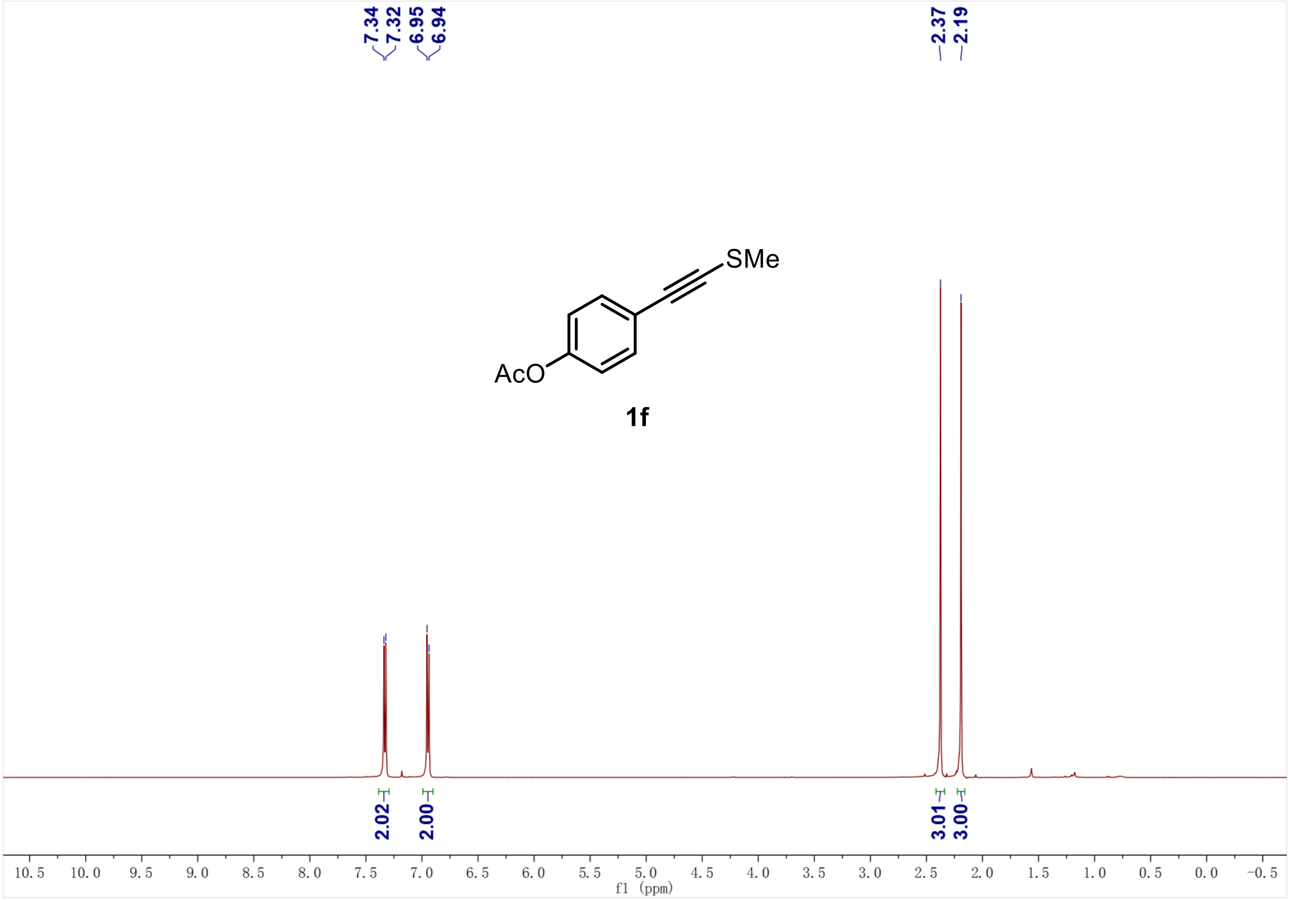

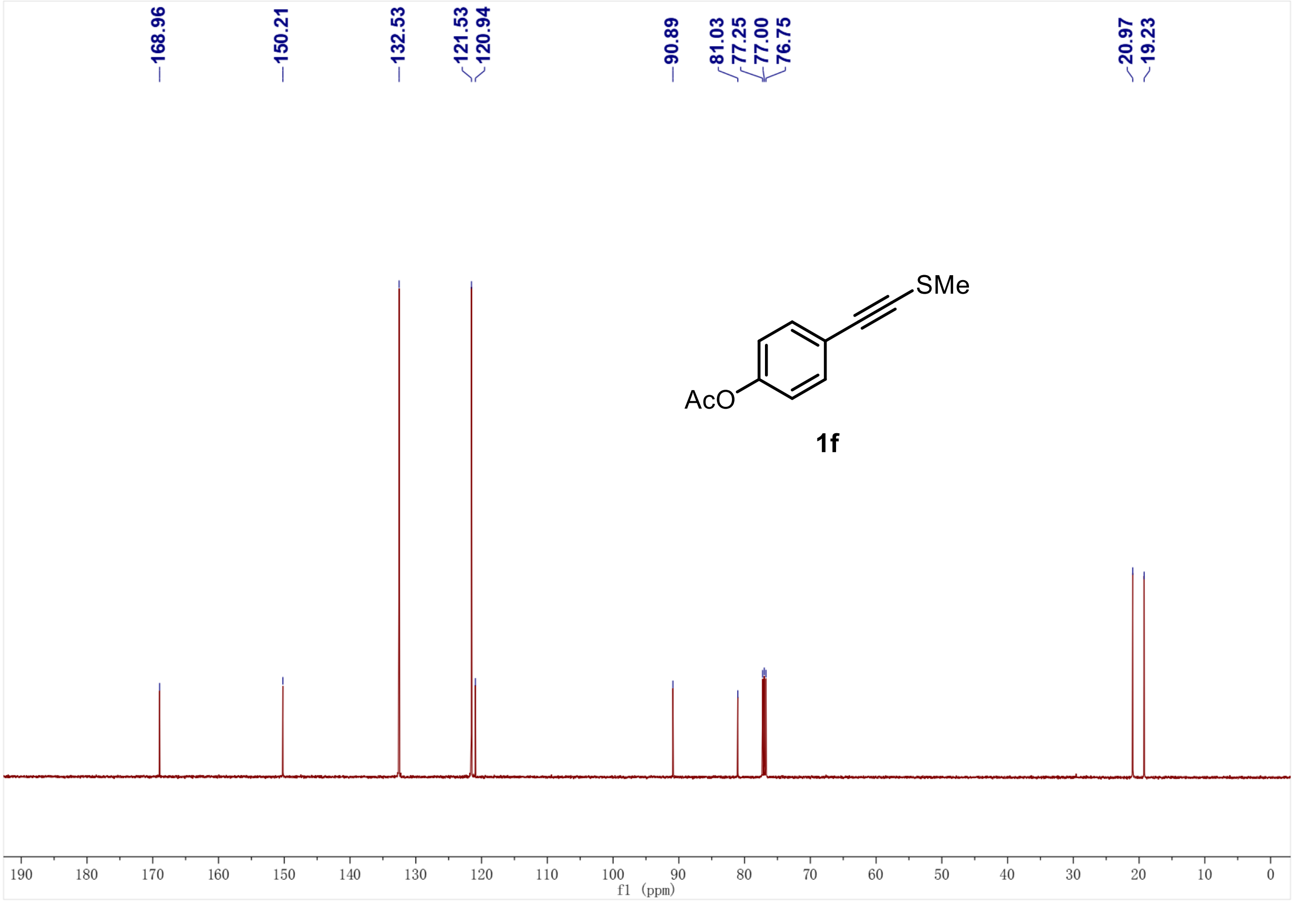

1g; ^1H NMR (500 MHz, $CDCl_3$); ^{13}C NMR (126 MHz, $CDCl_3$)

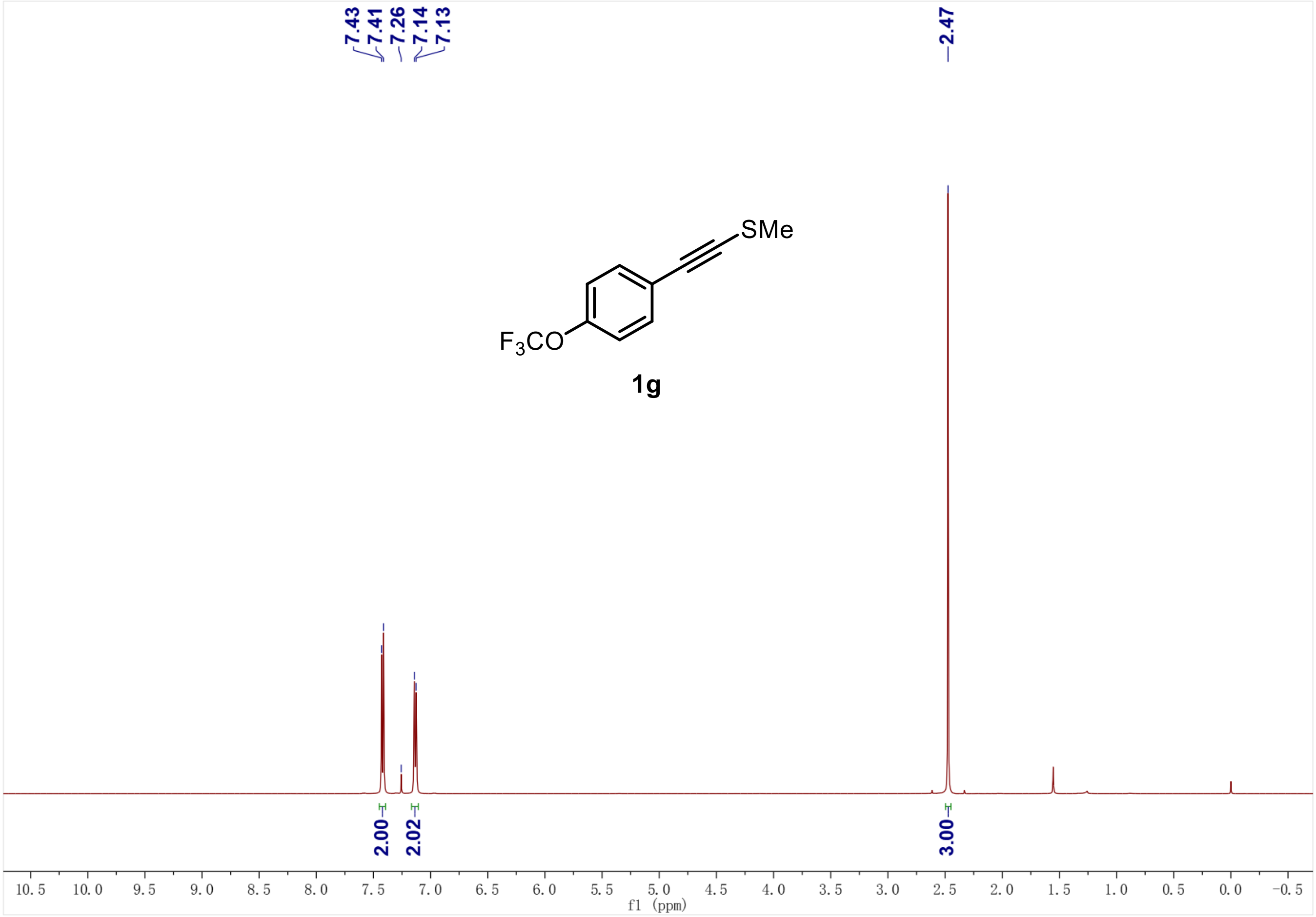

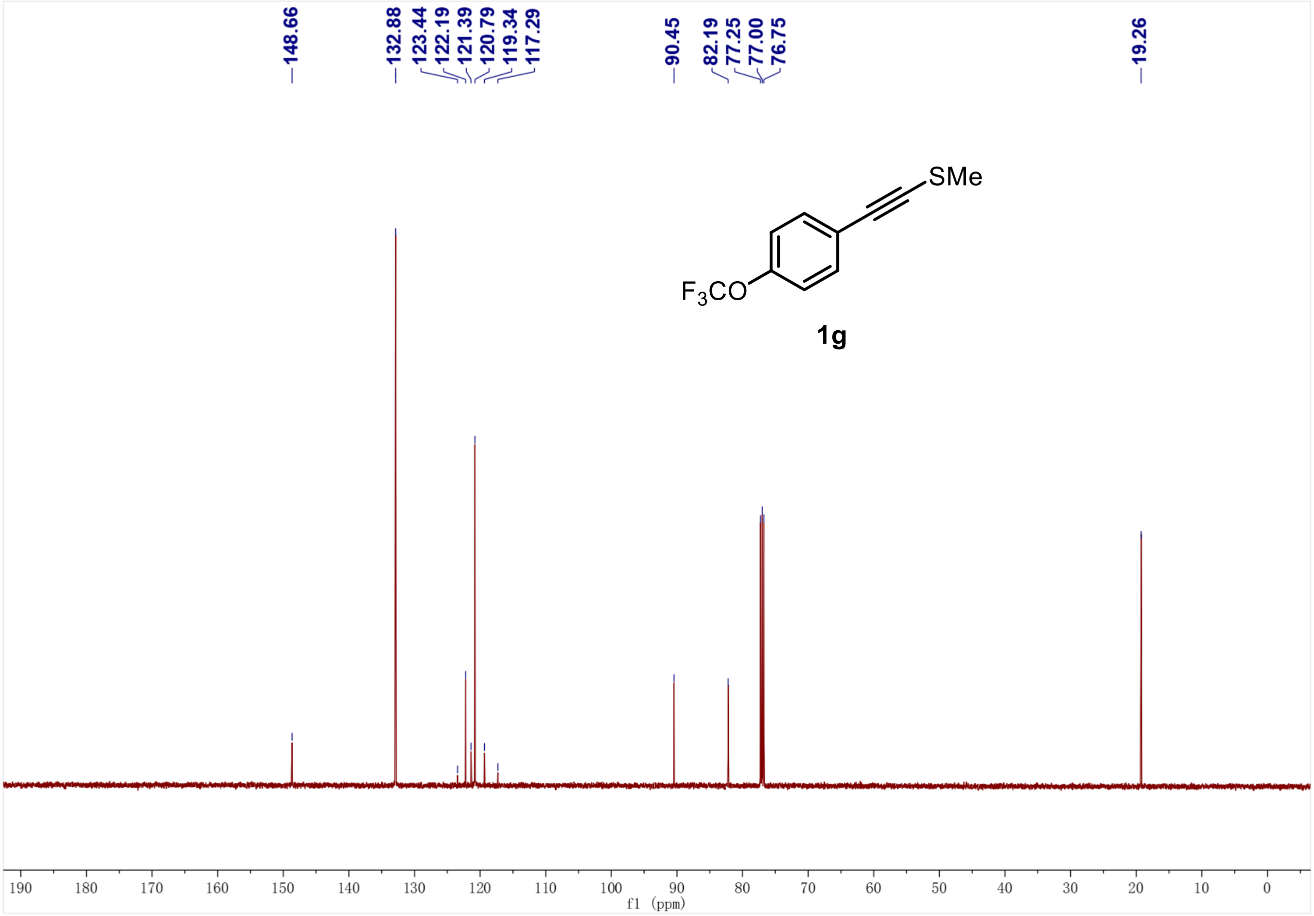

1g; ^{19}F NMR (471 MHz, $CDCl_3$)

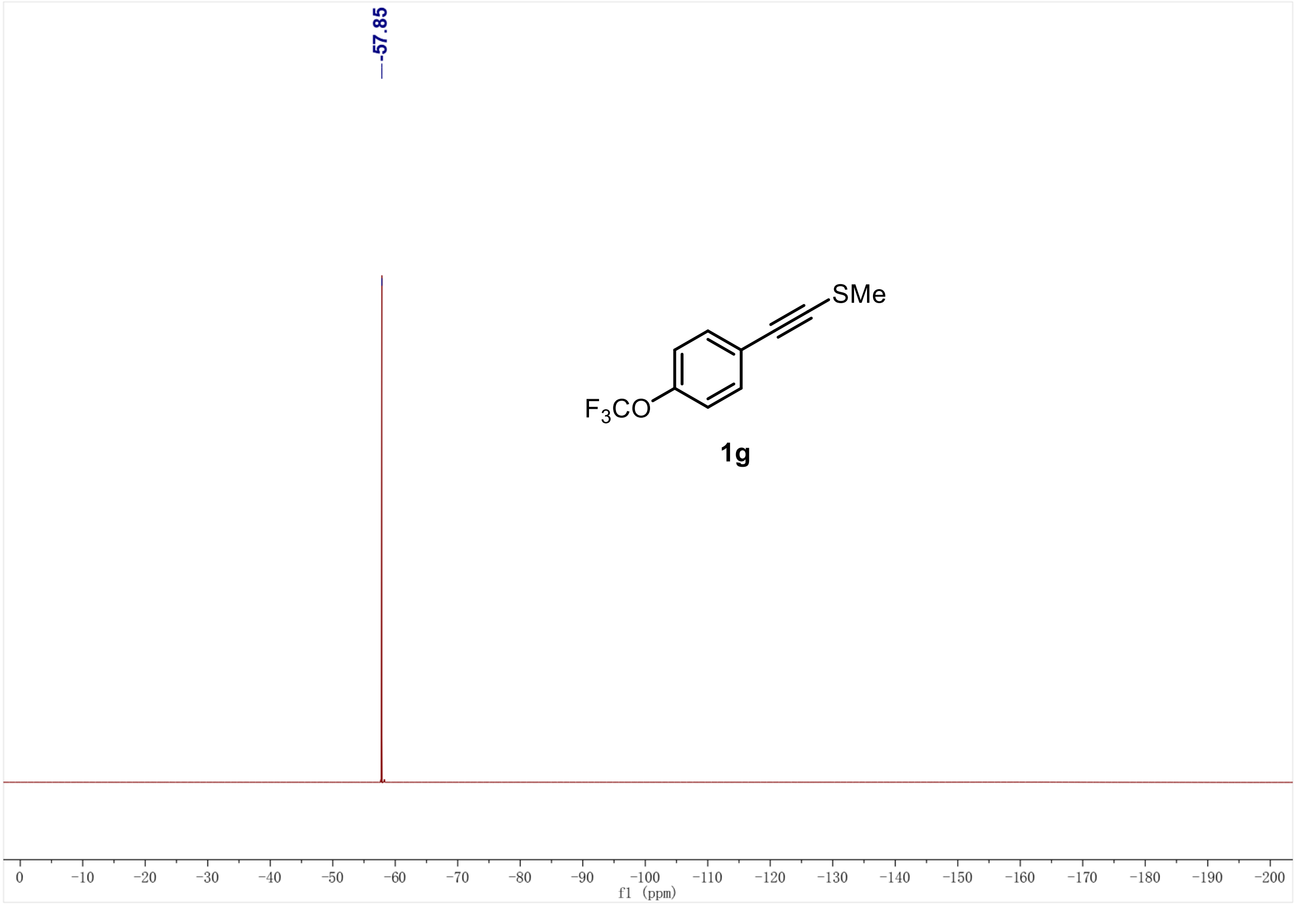

1o; ^{1}H NMR (500 MHz, $CDCl_3$); ^{13}C NMR (126 MHz, $CDCl_3$)

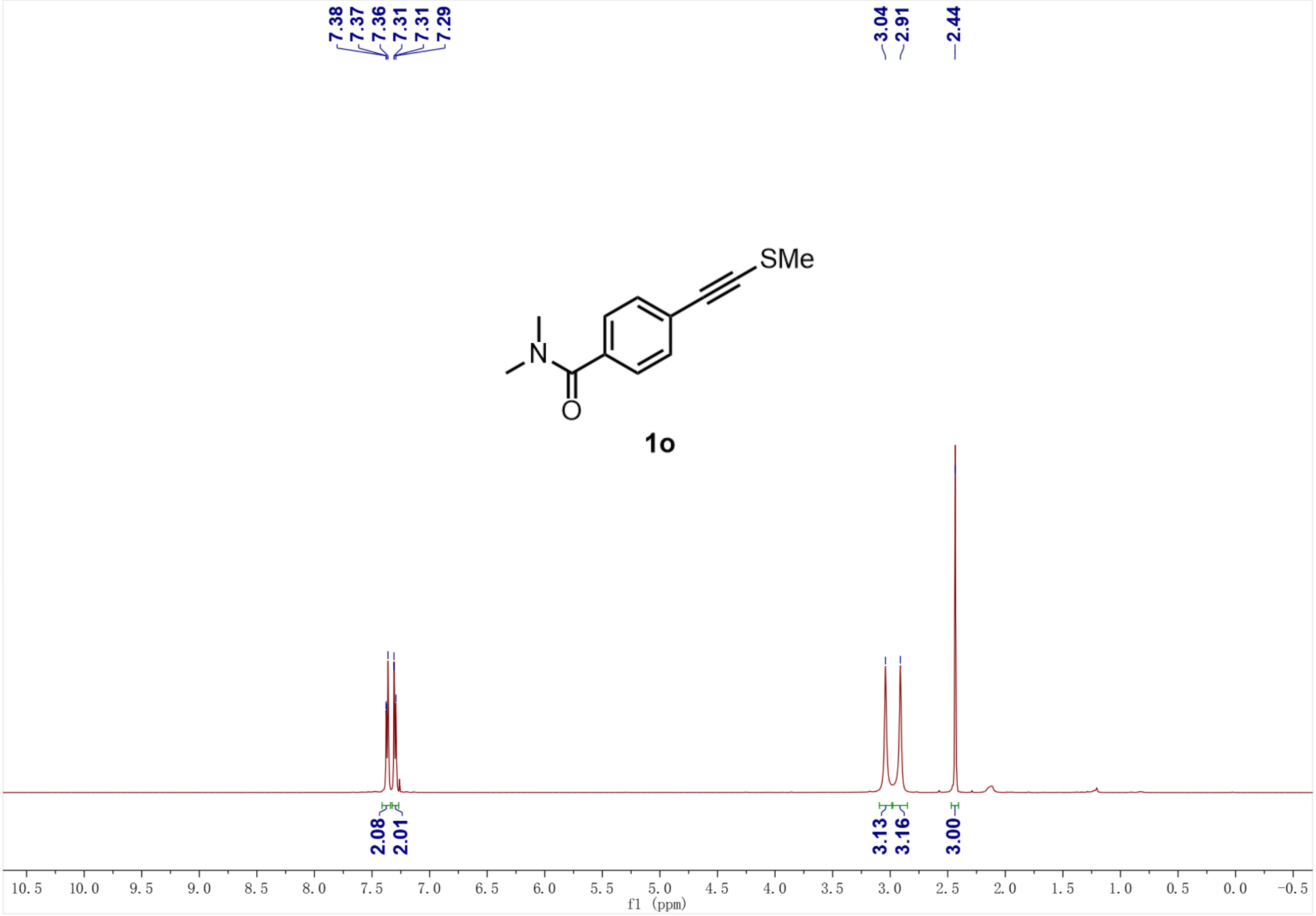

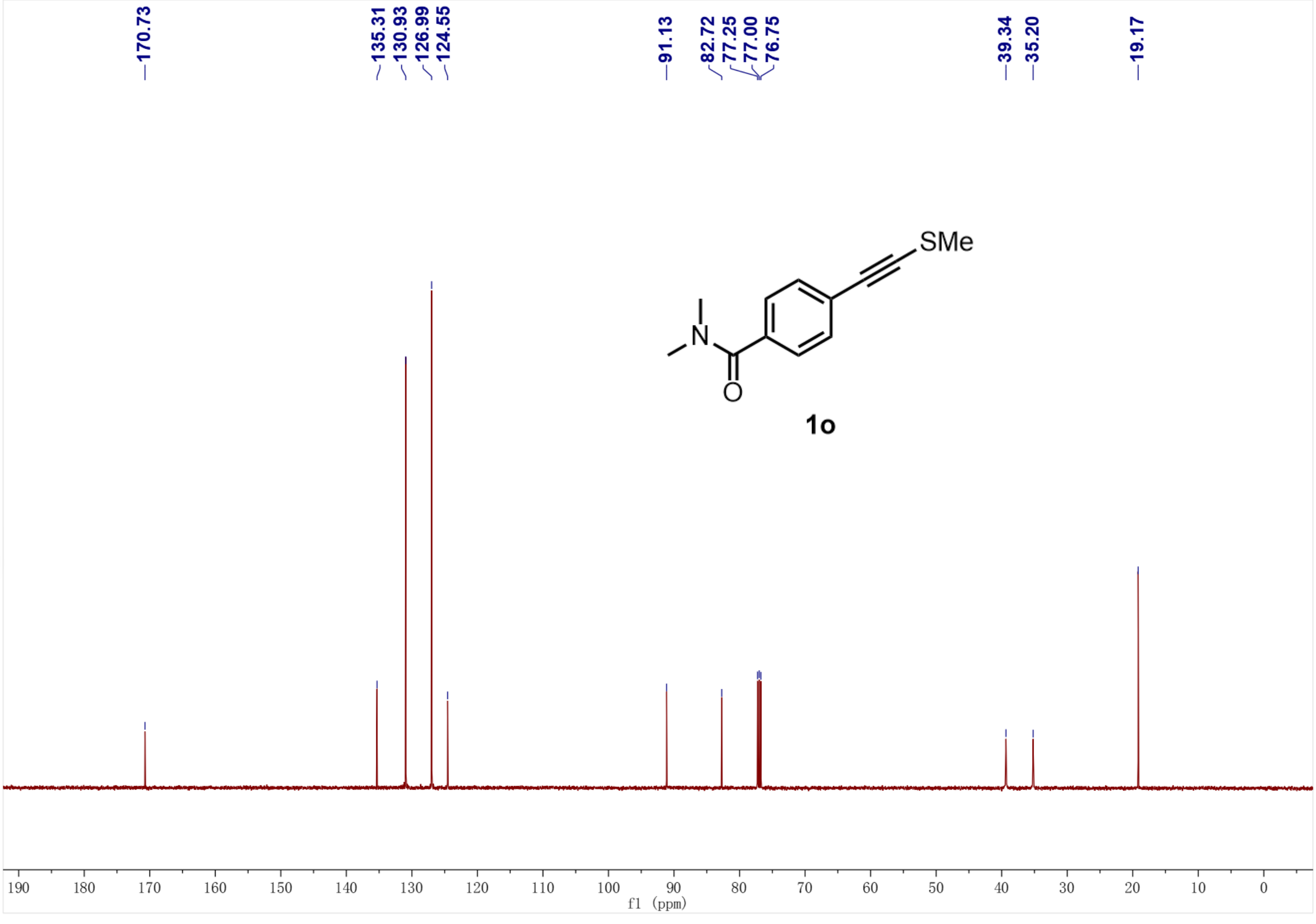

1p; ^{1}H NMR (500 MHz, $CDCl_3$); ^{13}C NMR (126 MHz, $CDCl_3$)

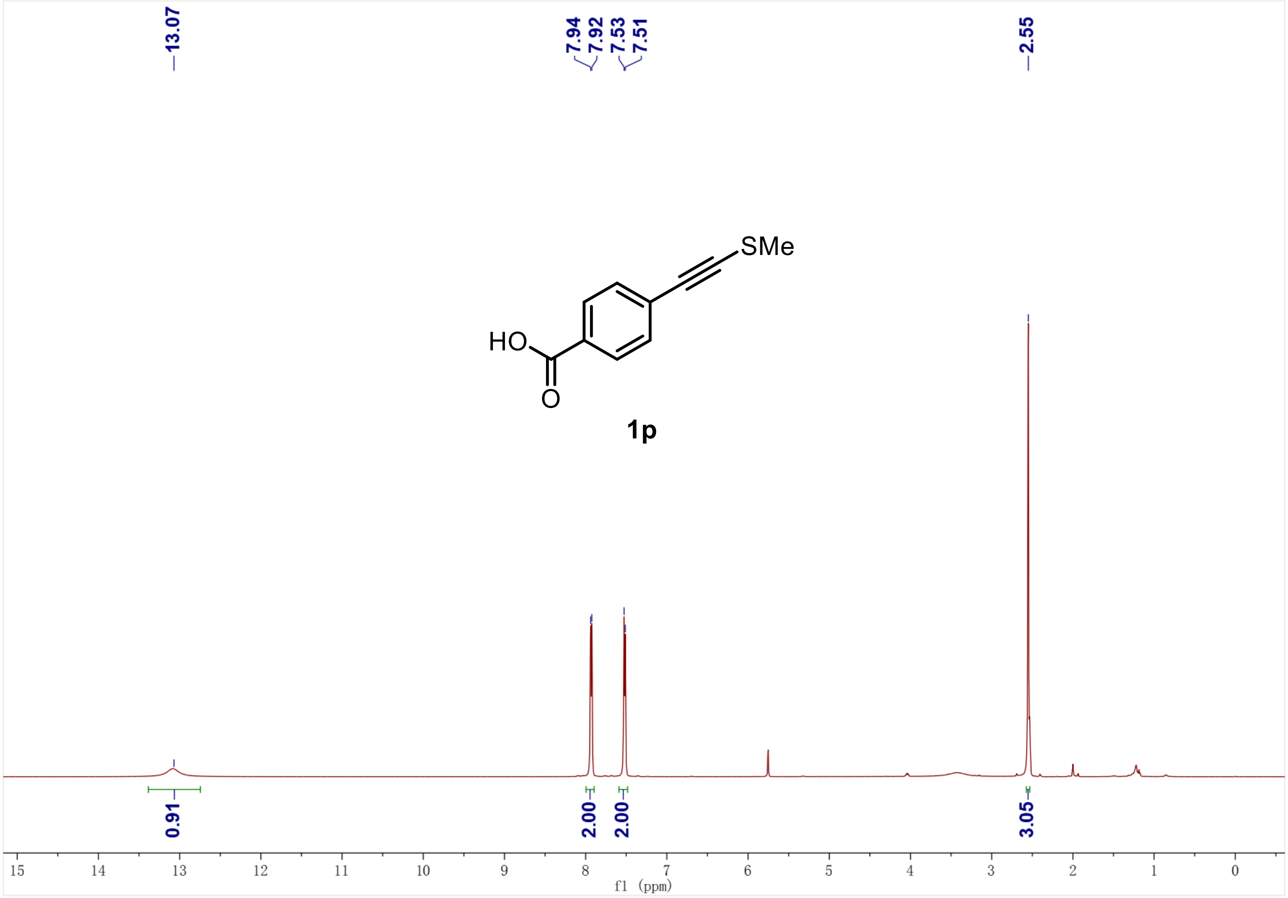

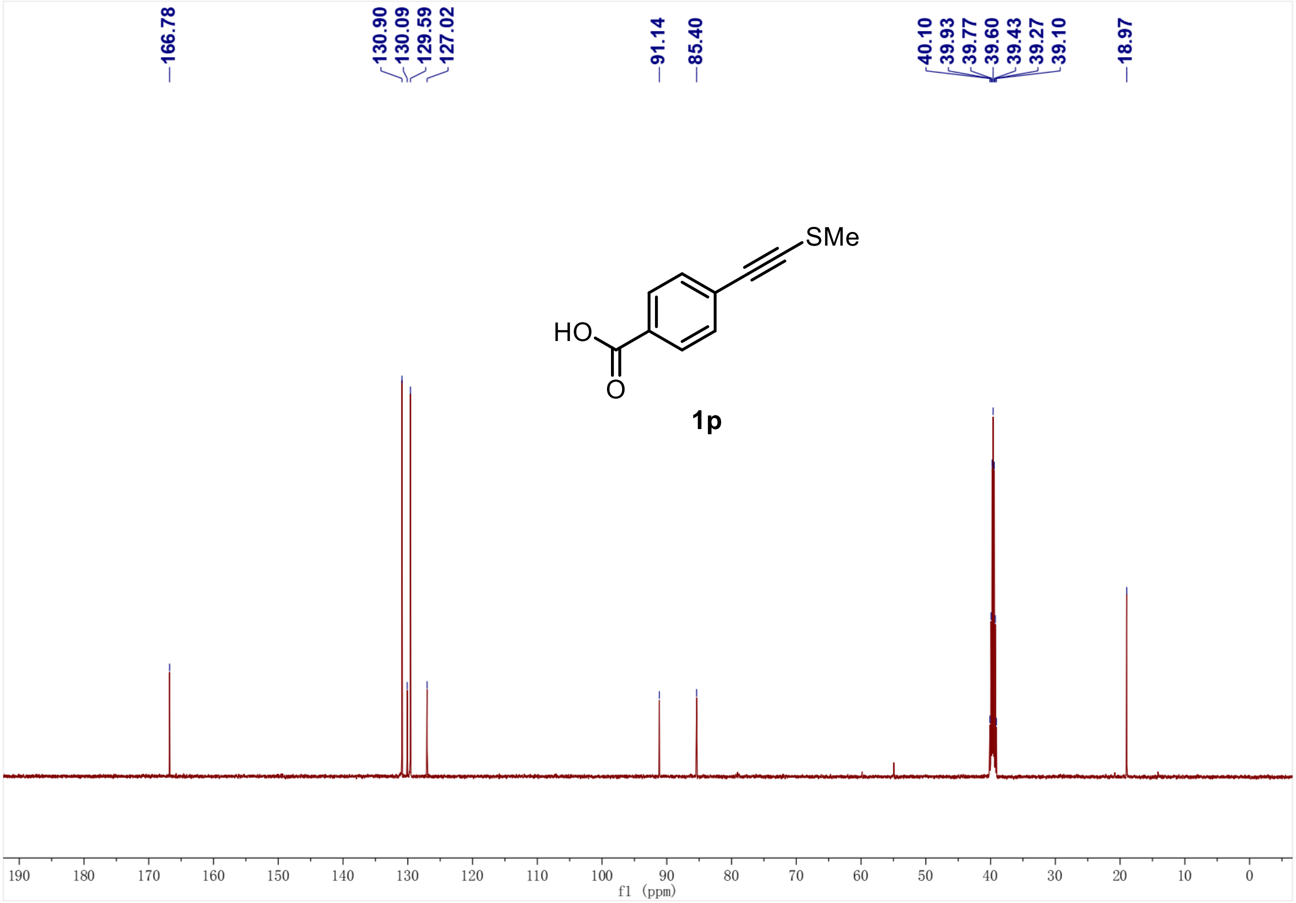

1r; ^{1}H NMR (500 MHz, $CDCl_3$); ^{13}C NMR (126 MHz, $CDCl_3$)

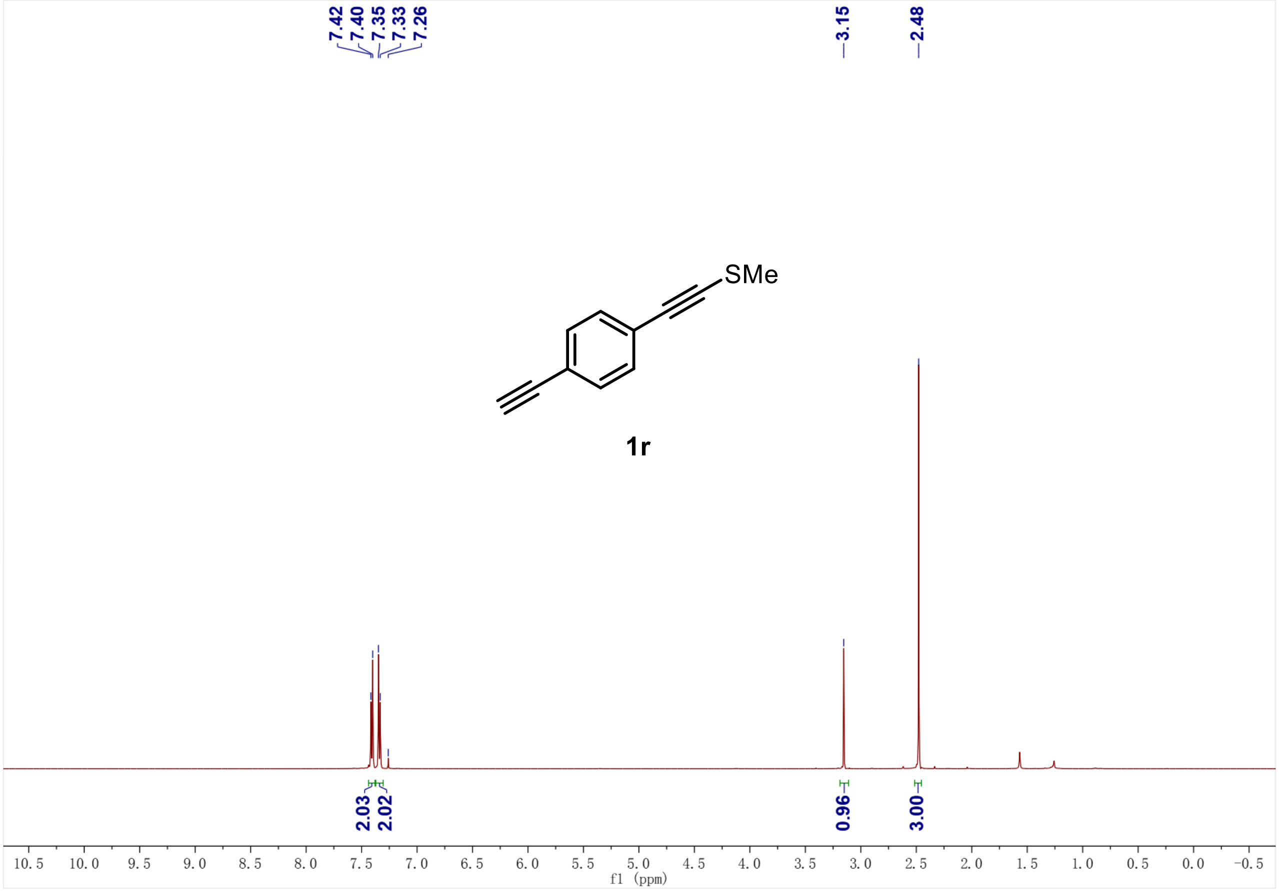

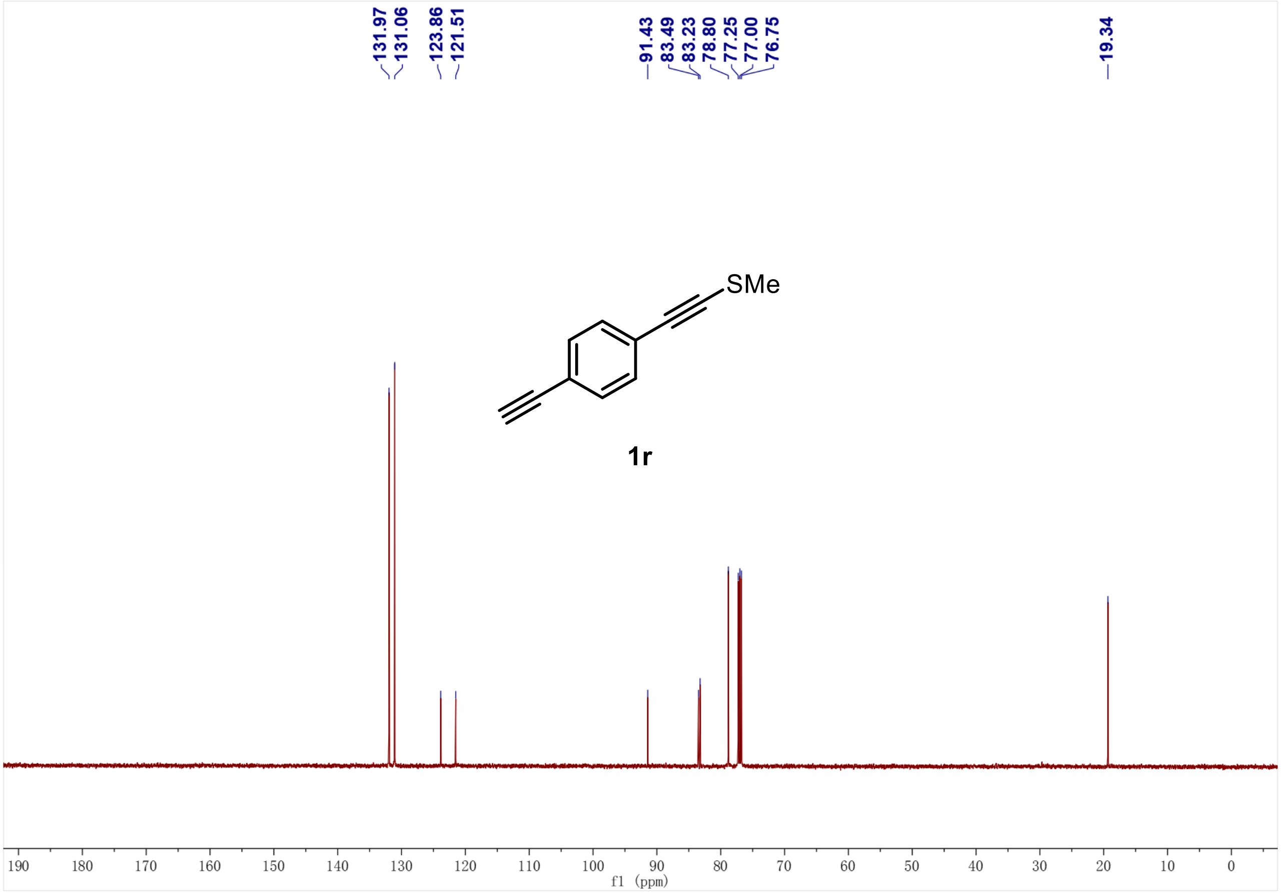

1ae; ^1H NMR (400 MHz, $CDCl_3$); ^{13}C NMR (101 MHz, $CDCl_3$)

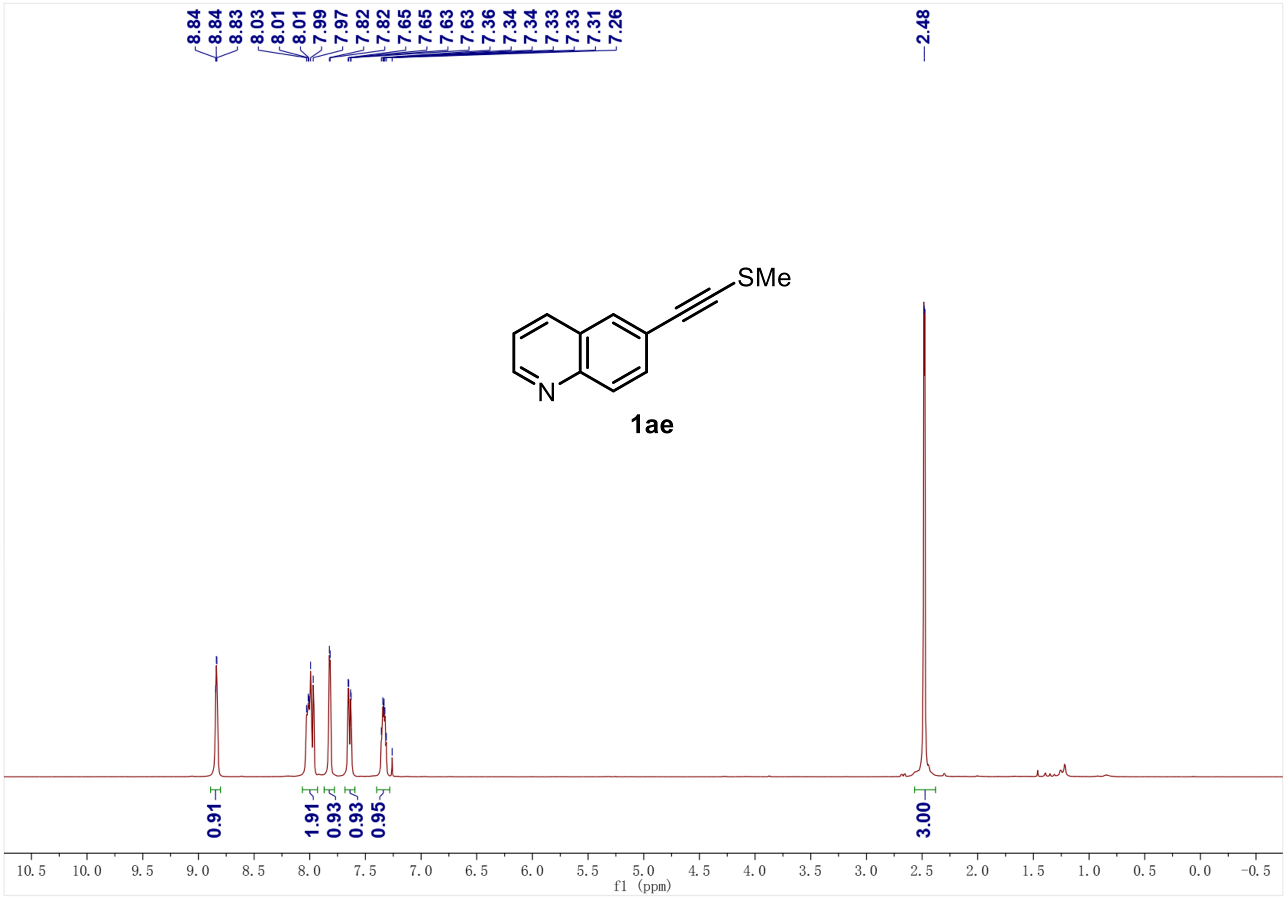

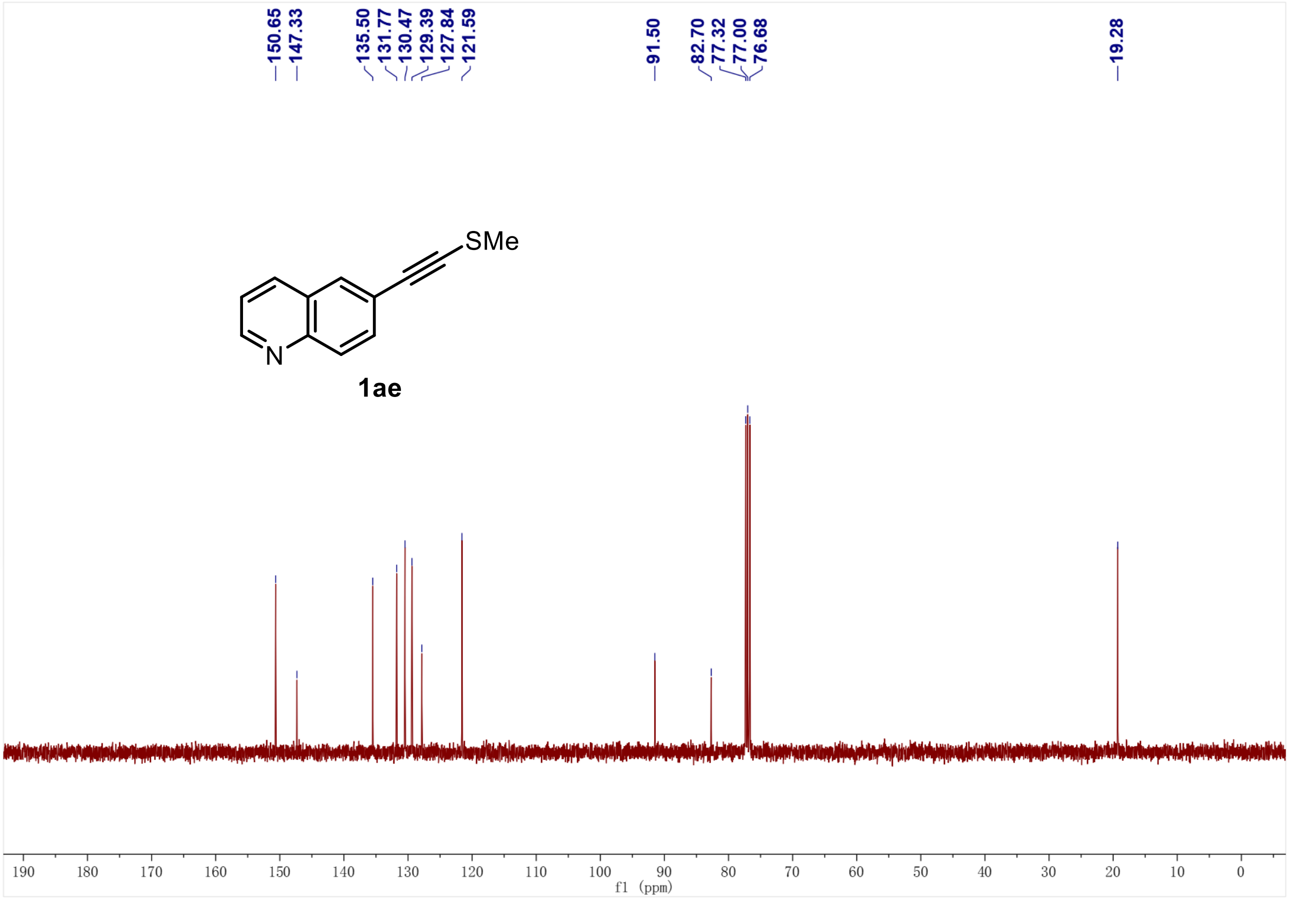

1ah; ^{1}H NMR (500 MHz, $CDCl_3$); ^{13}C NMR (126 MHz, $CDCl_3$)

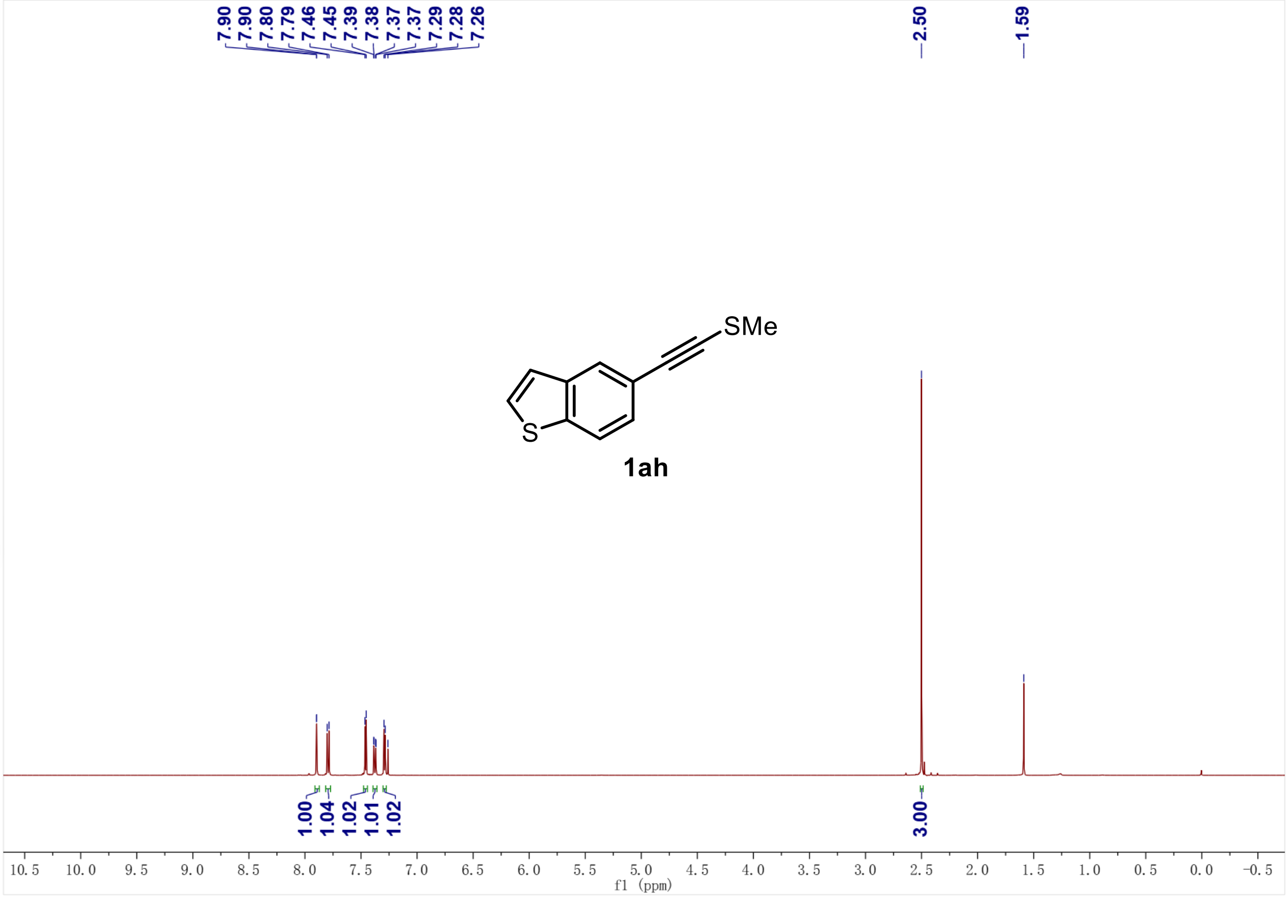

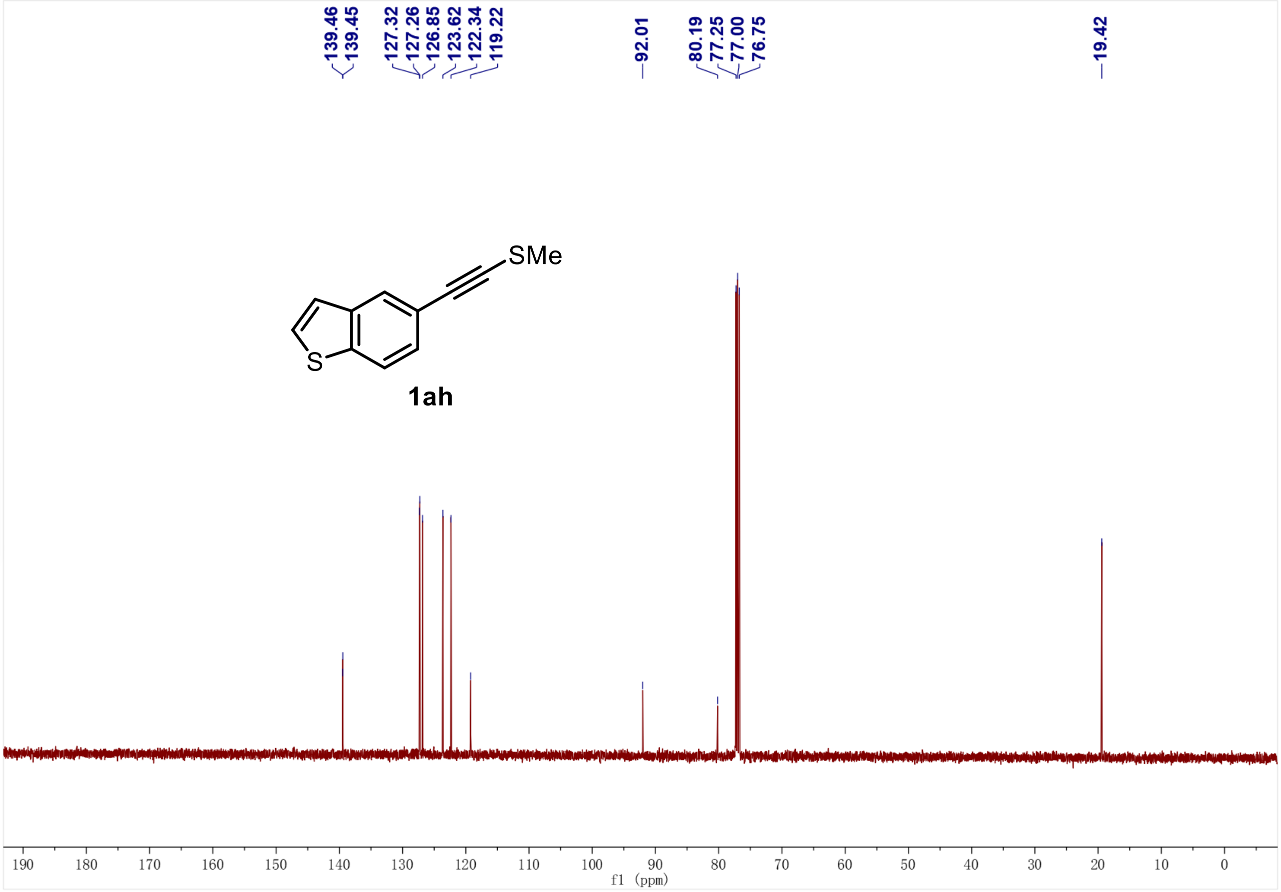

1ai; ^{1}H NMR (500 MHz, $CDCl_3$); ^{13}C NMR (126 MHz, $CDCl_3$)

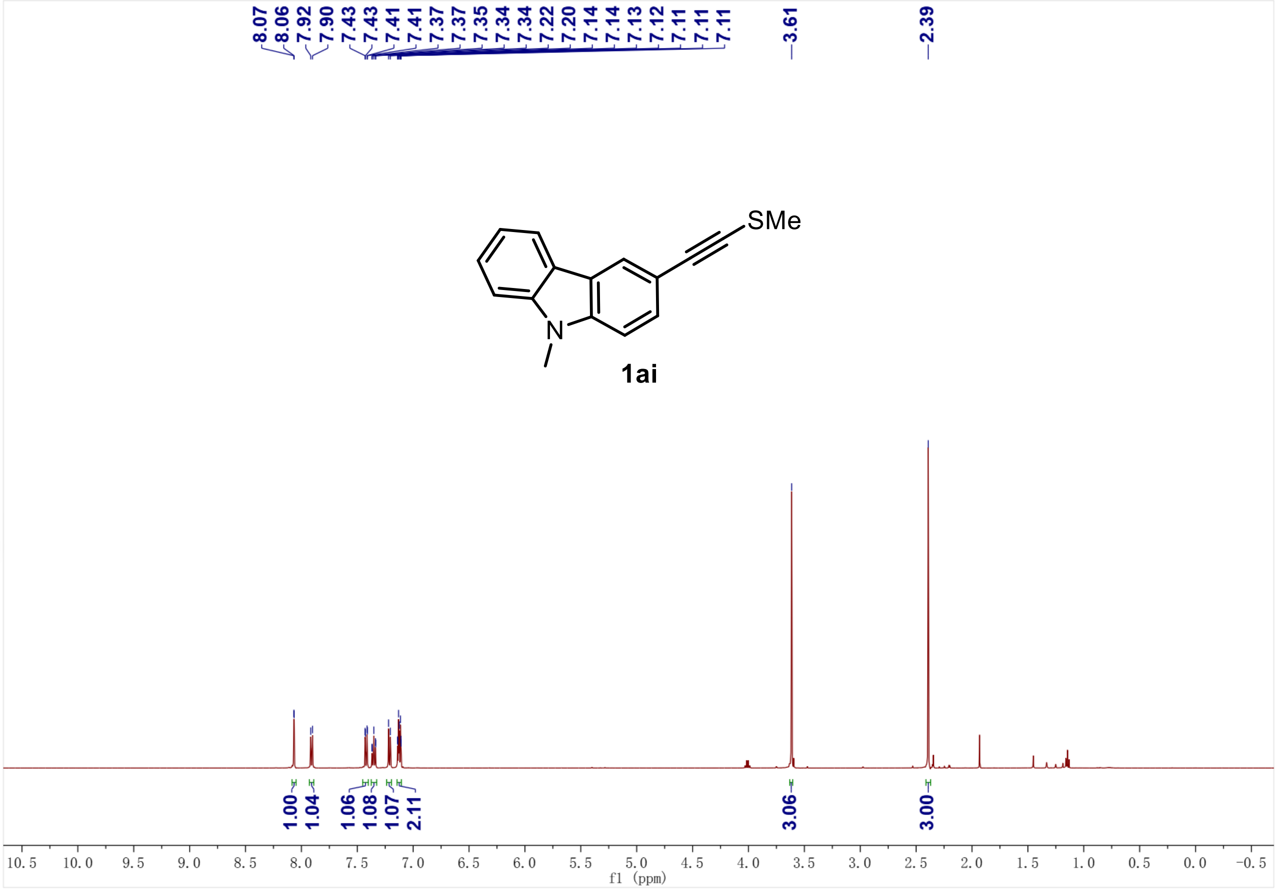

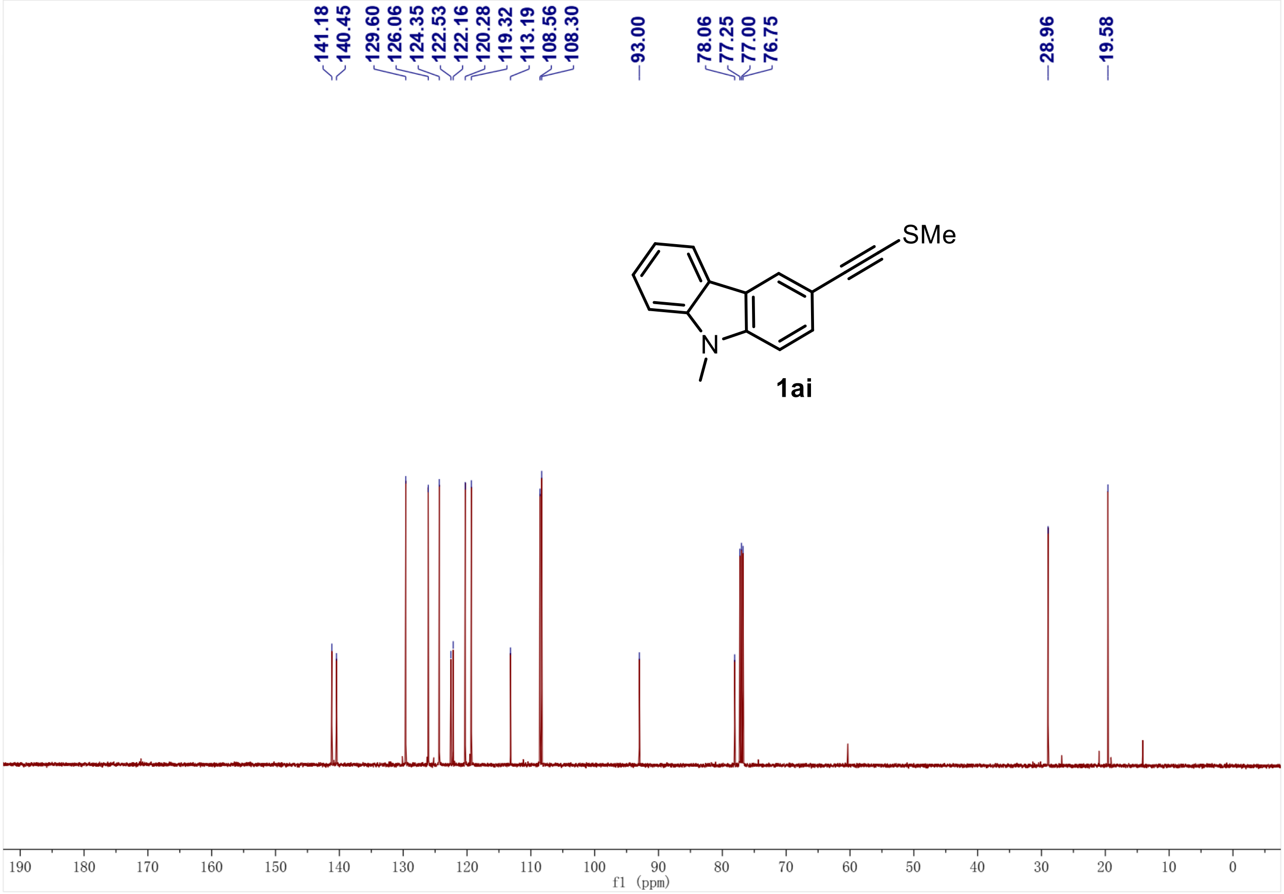

1aj; ^{1}H NMR (400 MHz, $CDCl_3$); ^{13}C NMR (101 MHz, $CDCl_3$)

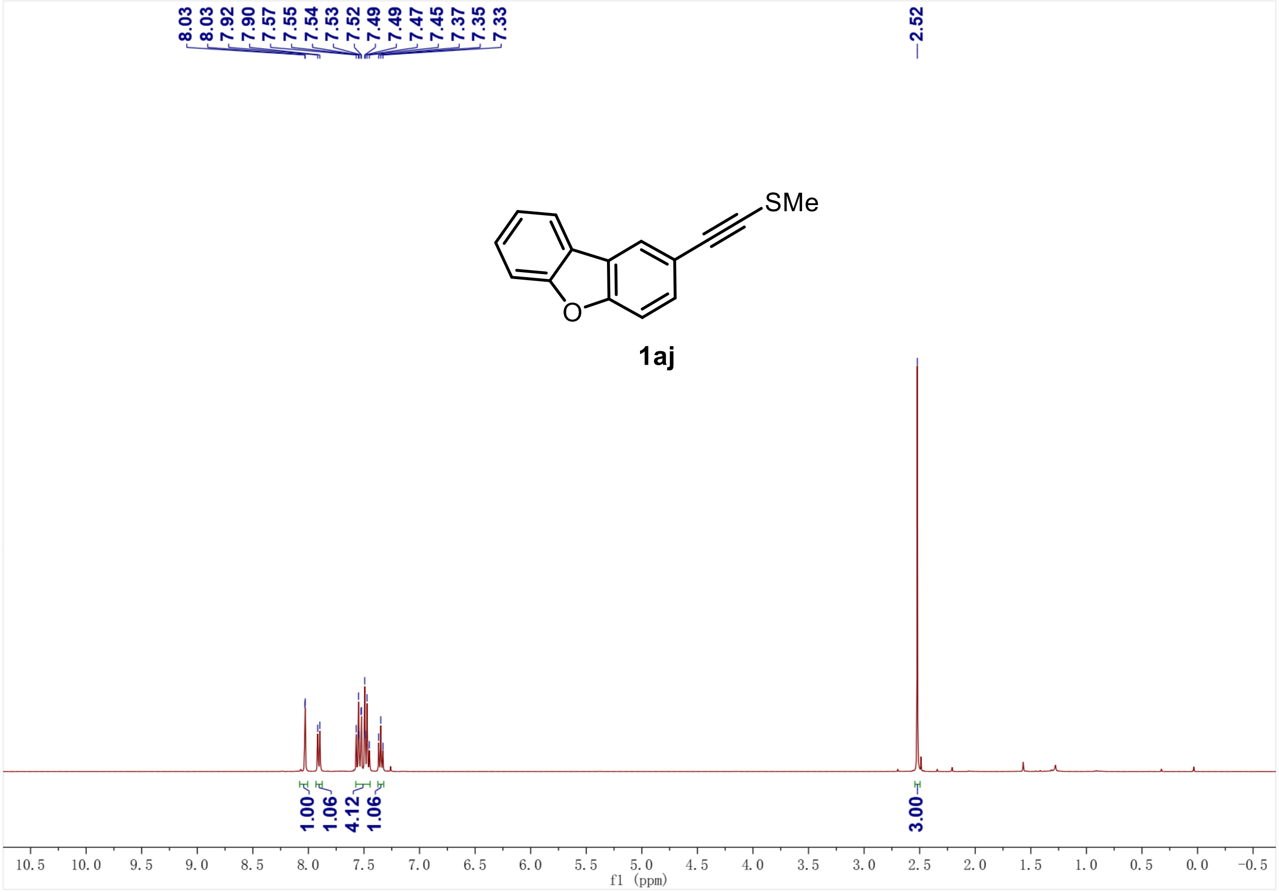

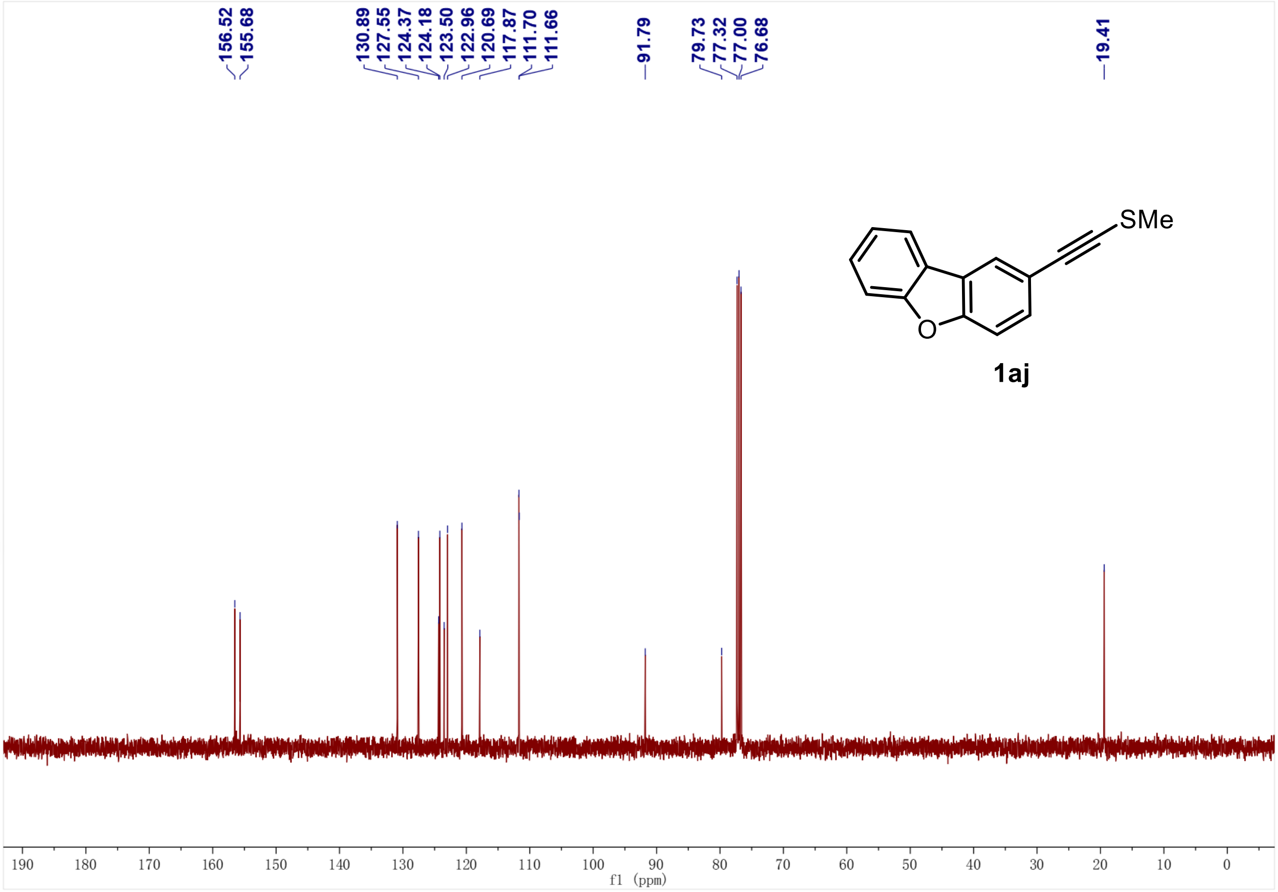

1ak; ^{1}H NMR (500 MHz, $CDCl_3$); ^{13}C NMR (126 MHz, $CDCl_3$)

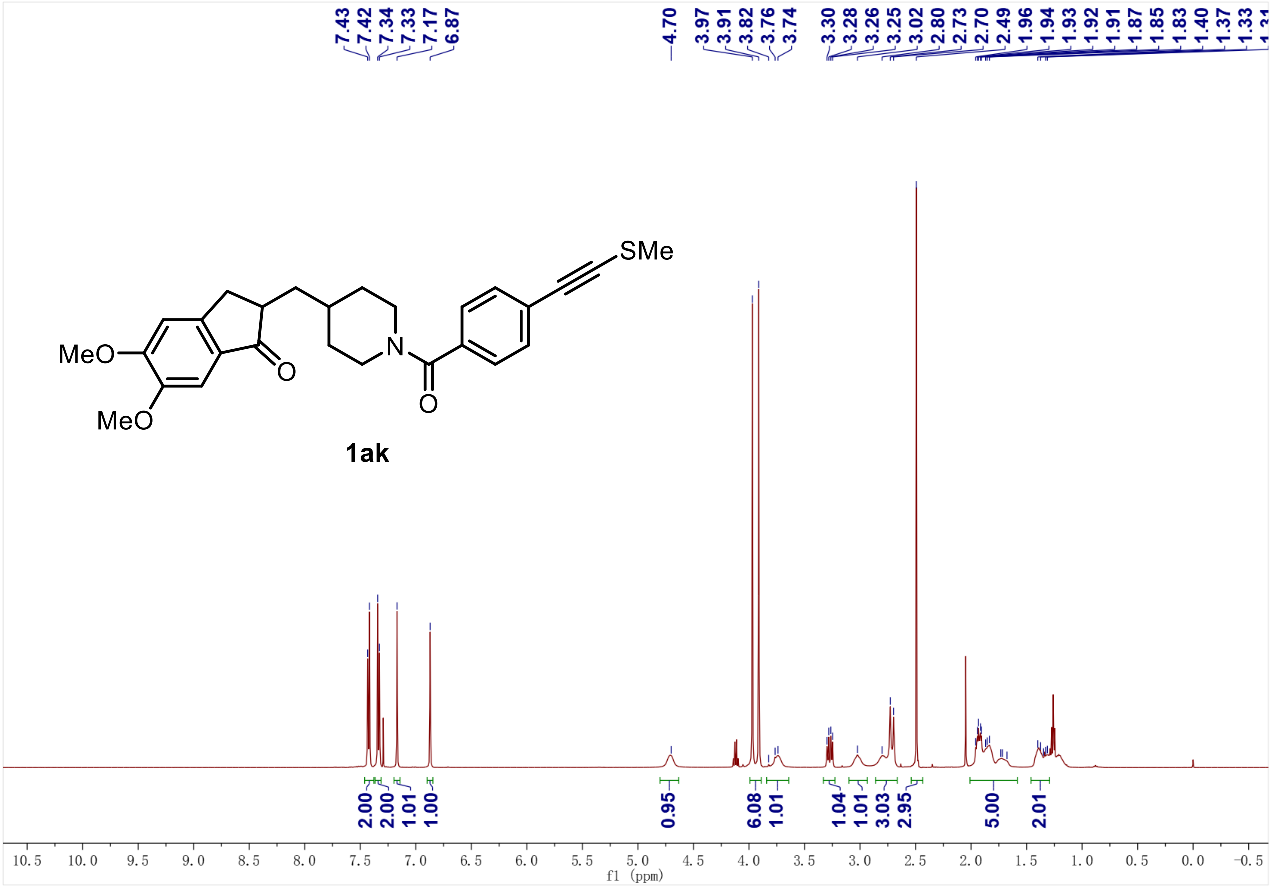

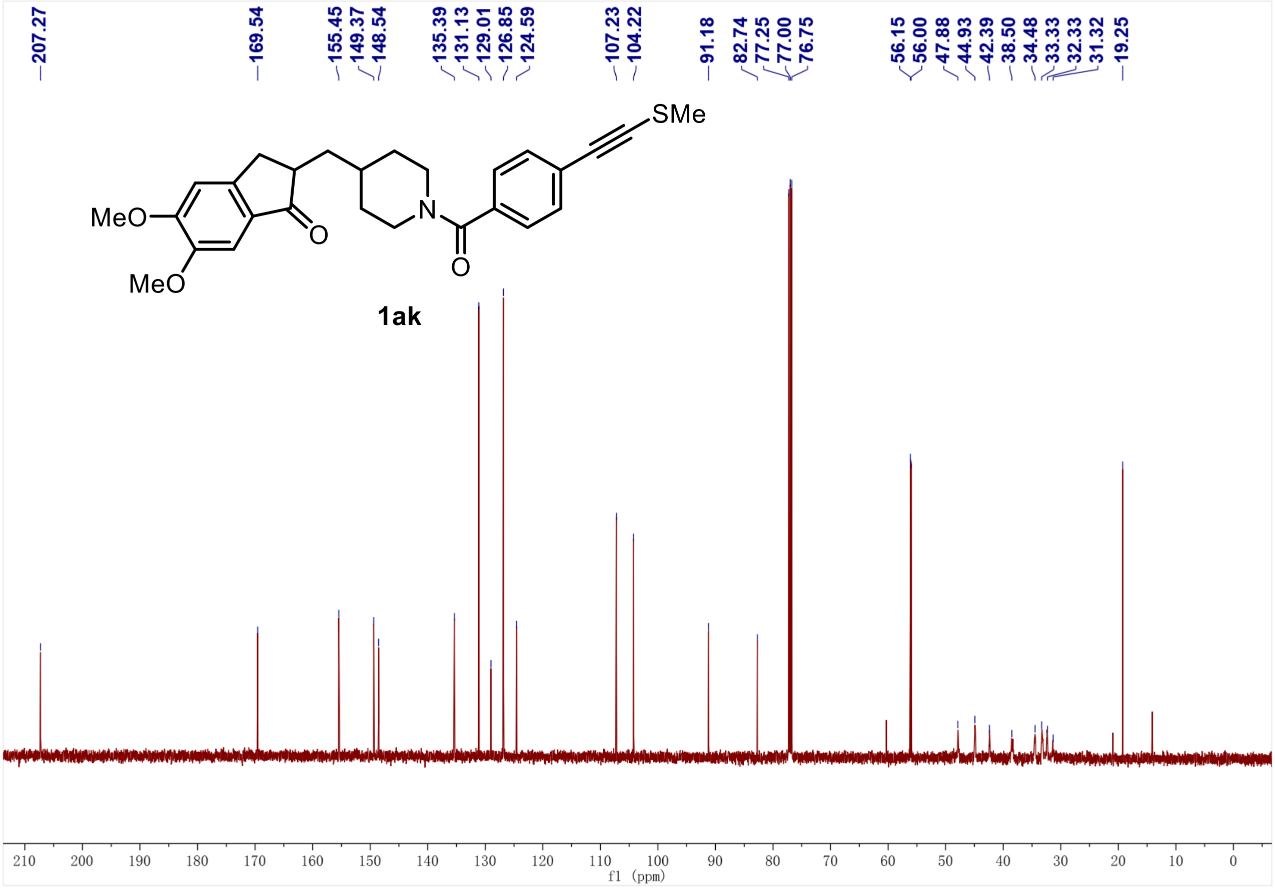

1al; ^{1}H NMR (500 MHz, CDCl$_3$); ^{13}C NMR (126 MHz, CDCl$_3$)

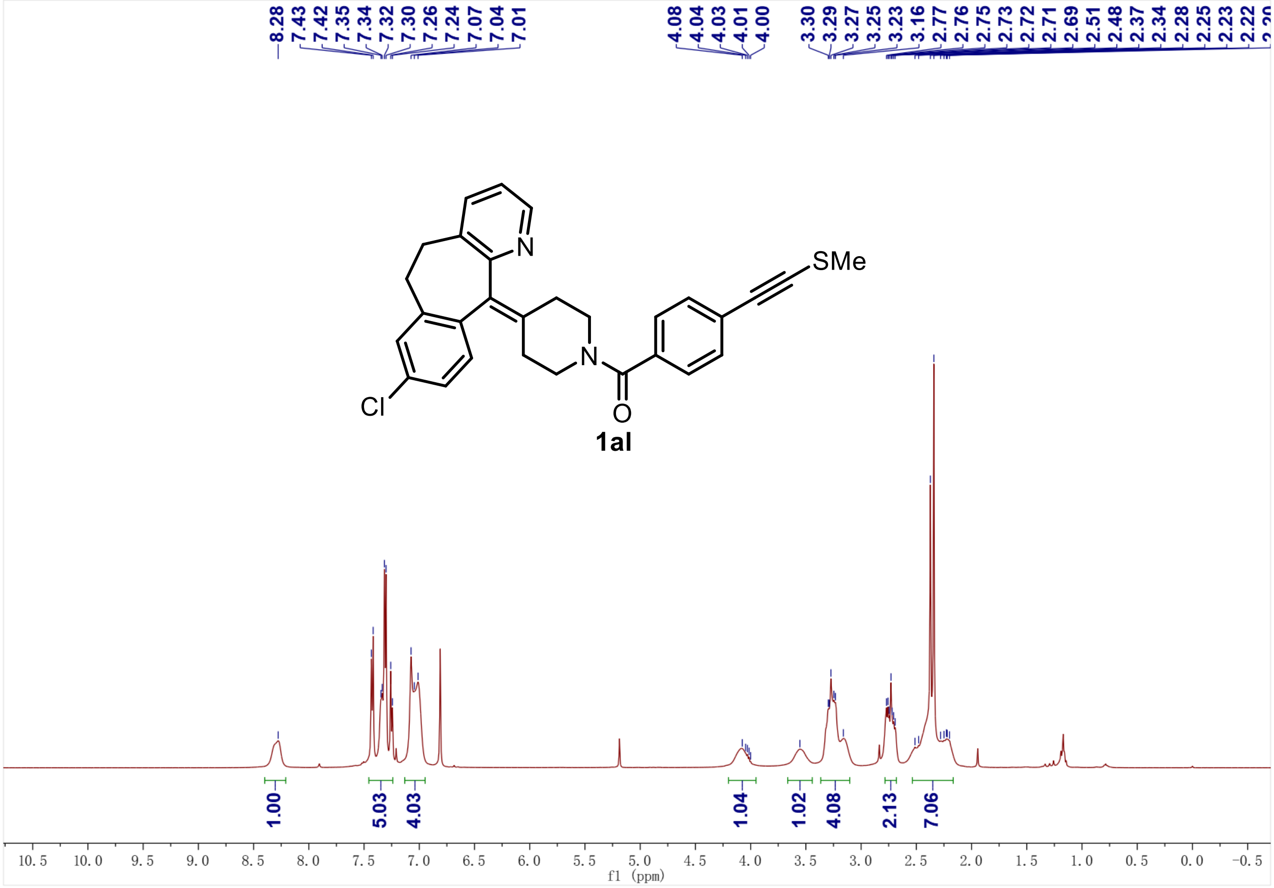

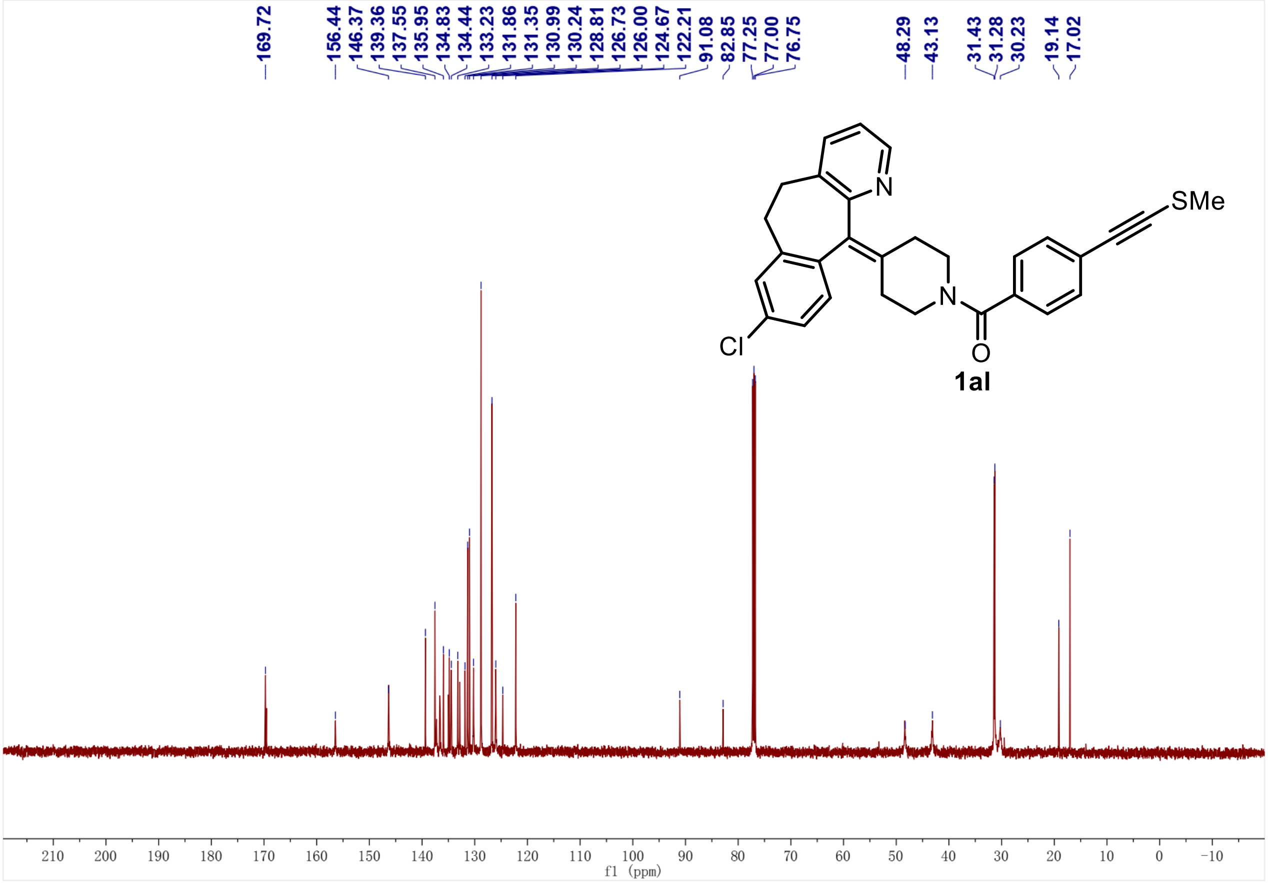

1ao; ^{1}H NMR (500 MHz, $CDCl_3$); ^{13}C NMR (126 MHz, $CDCl_3$)

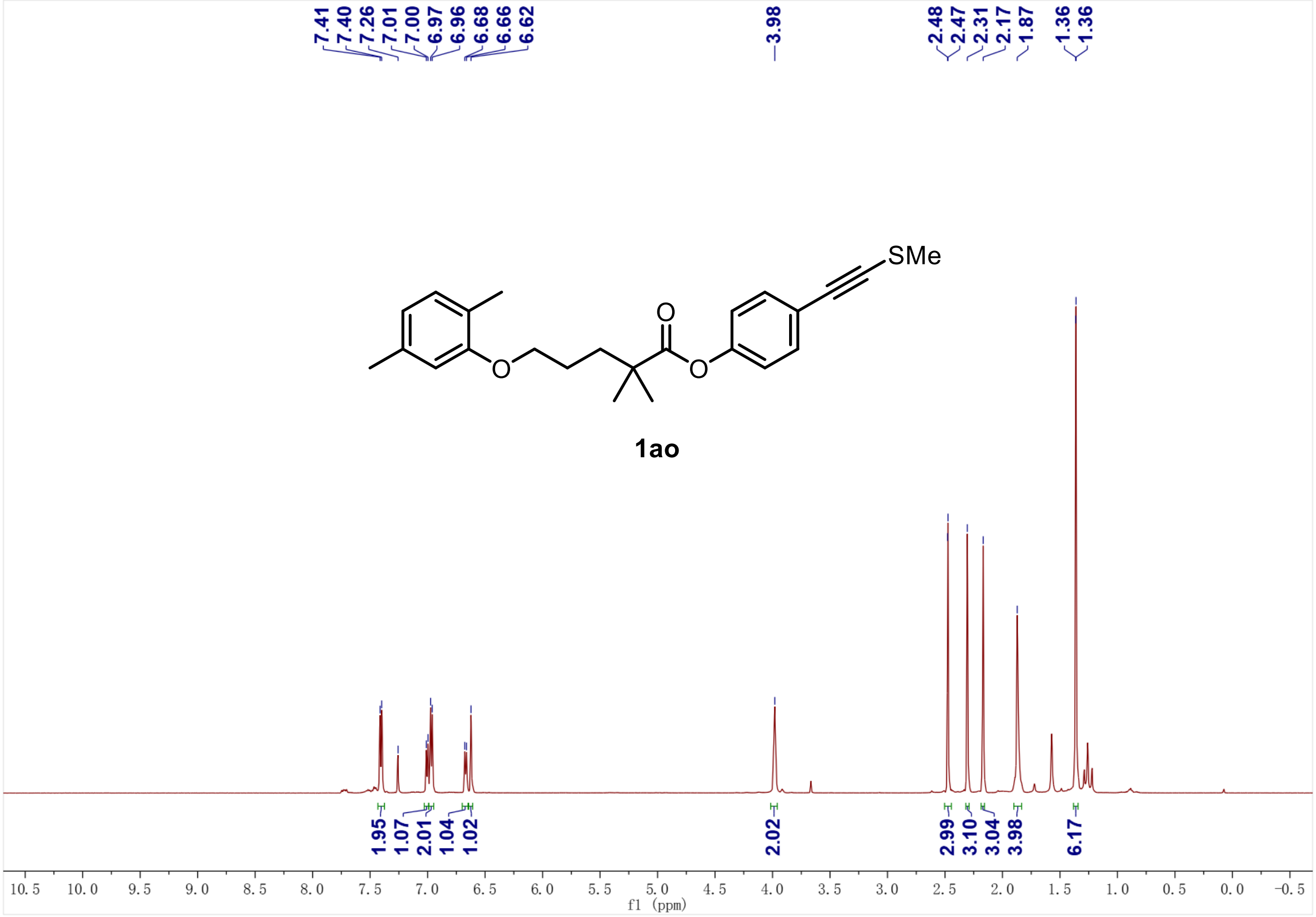

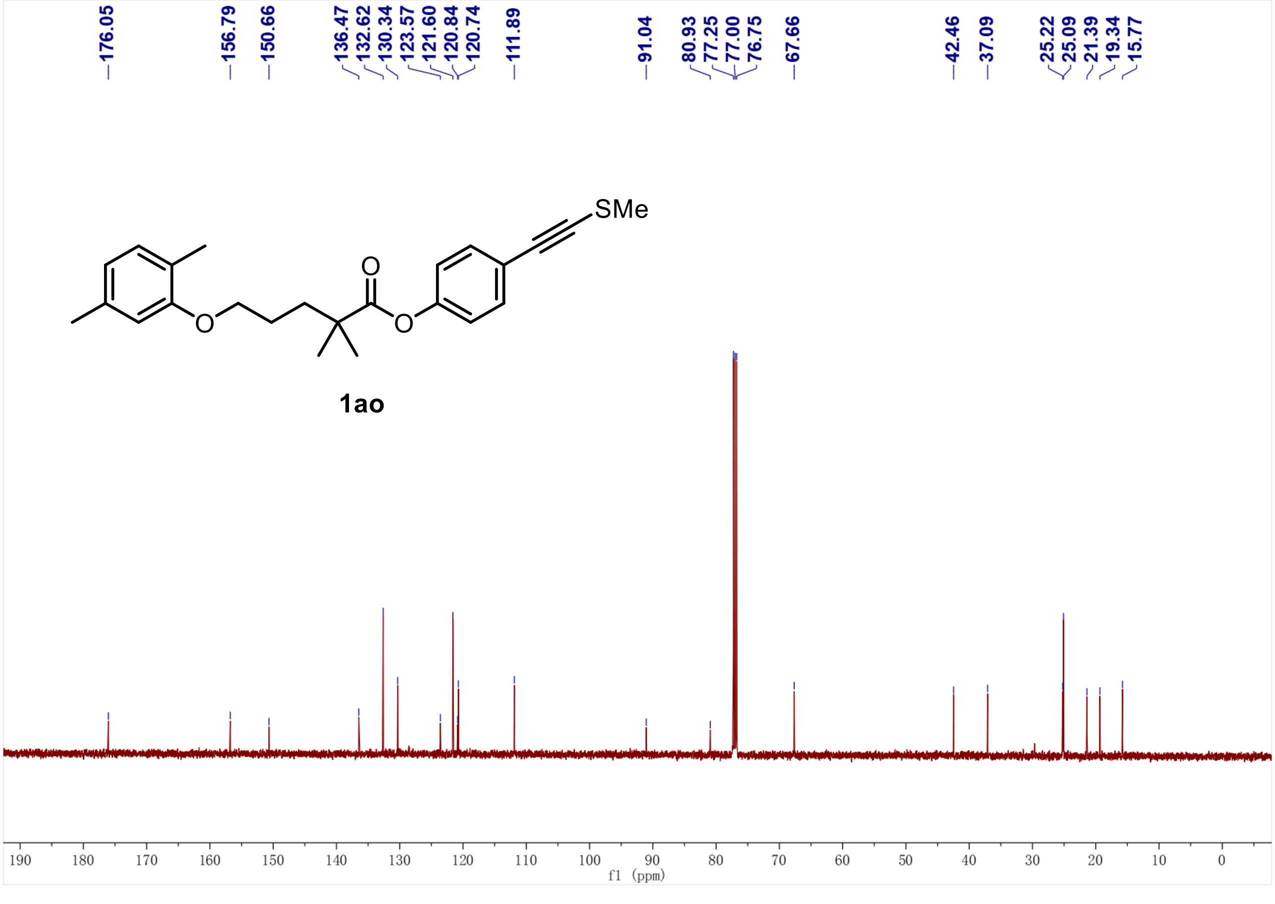

1ap; ^{1}H NMR (500 MHz, $CDCl_3$); ^{13}C NMR (126 MHz, $CDCl_3$)

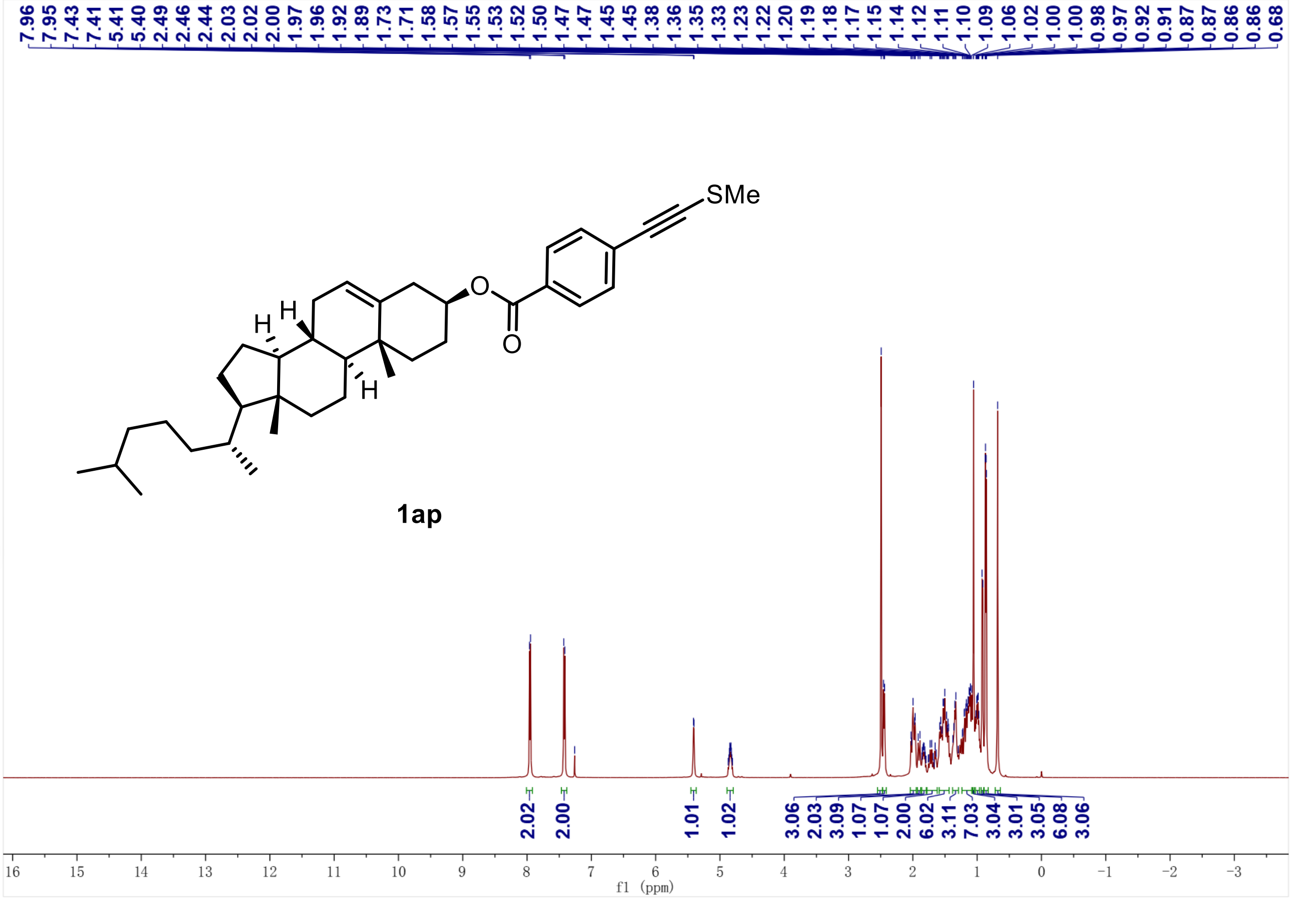

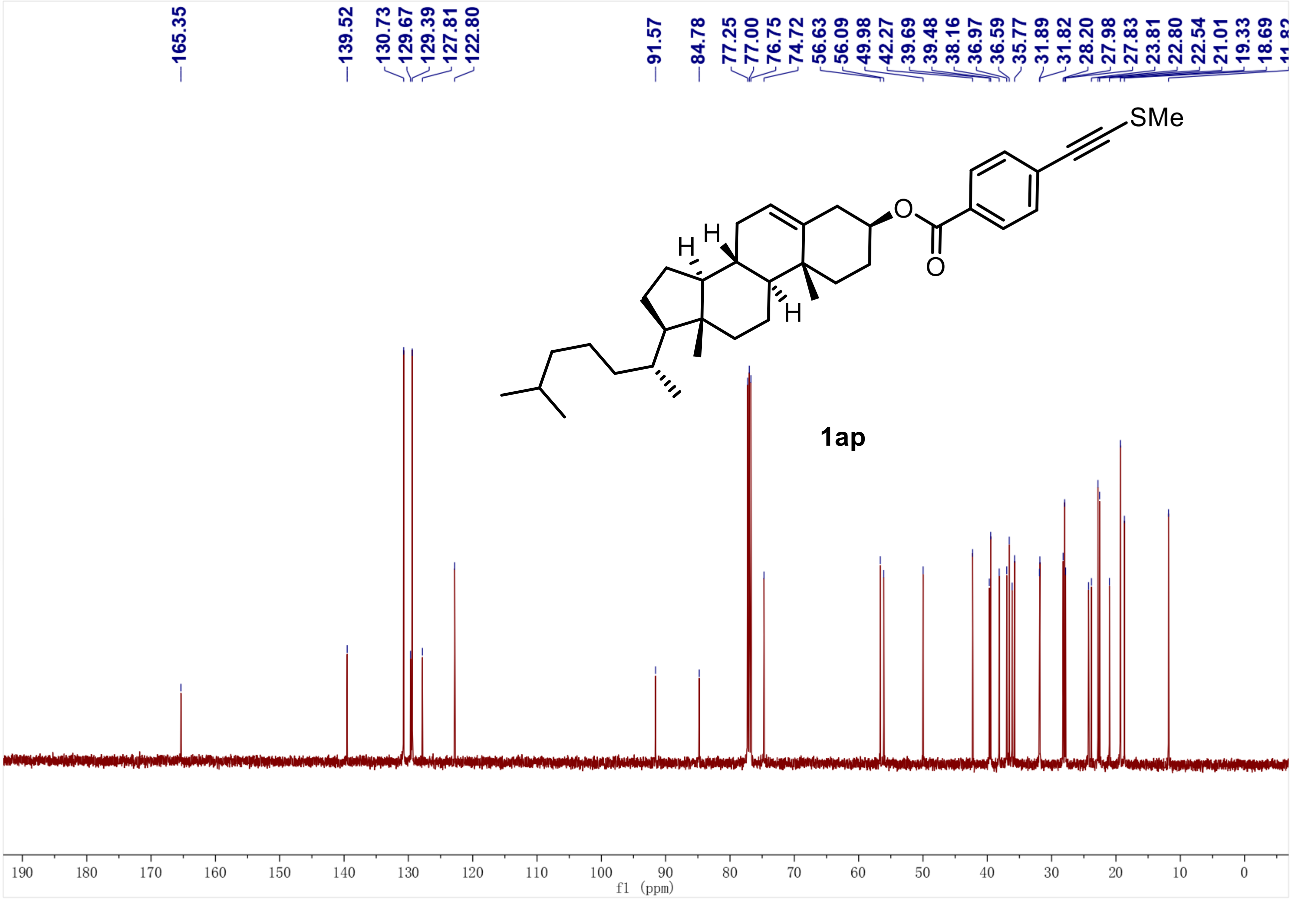

1as; ^{1}H NMR (500 MHz, $CDCl_3$); ^{13}C NMR (126 MHz, $CDCl_3$)

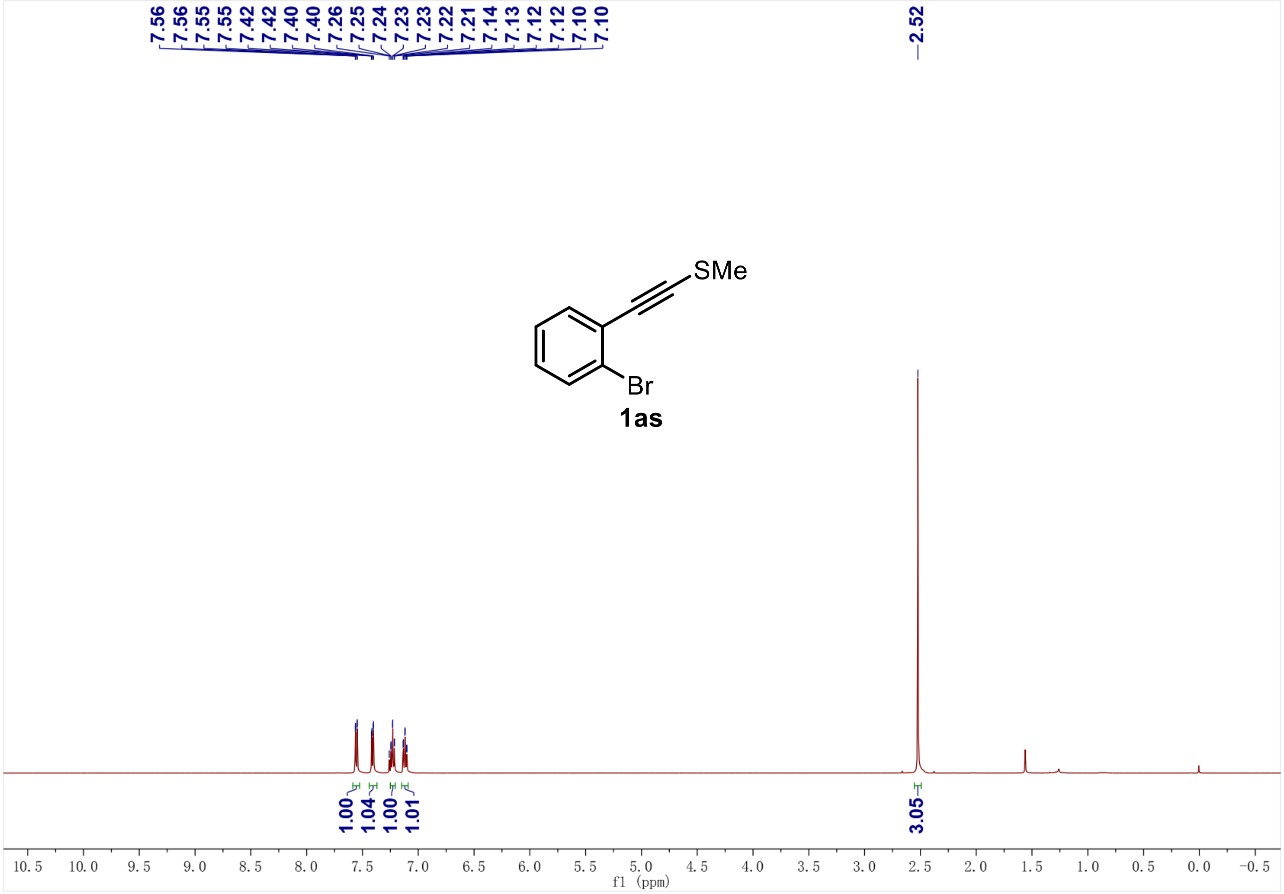

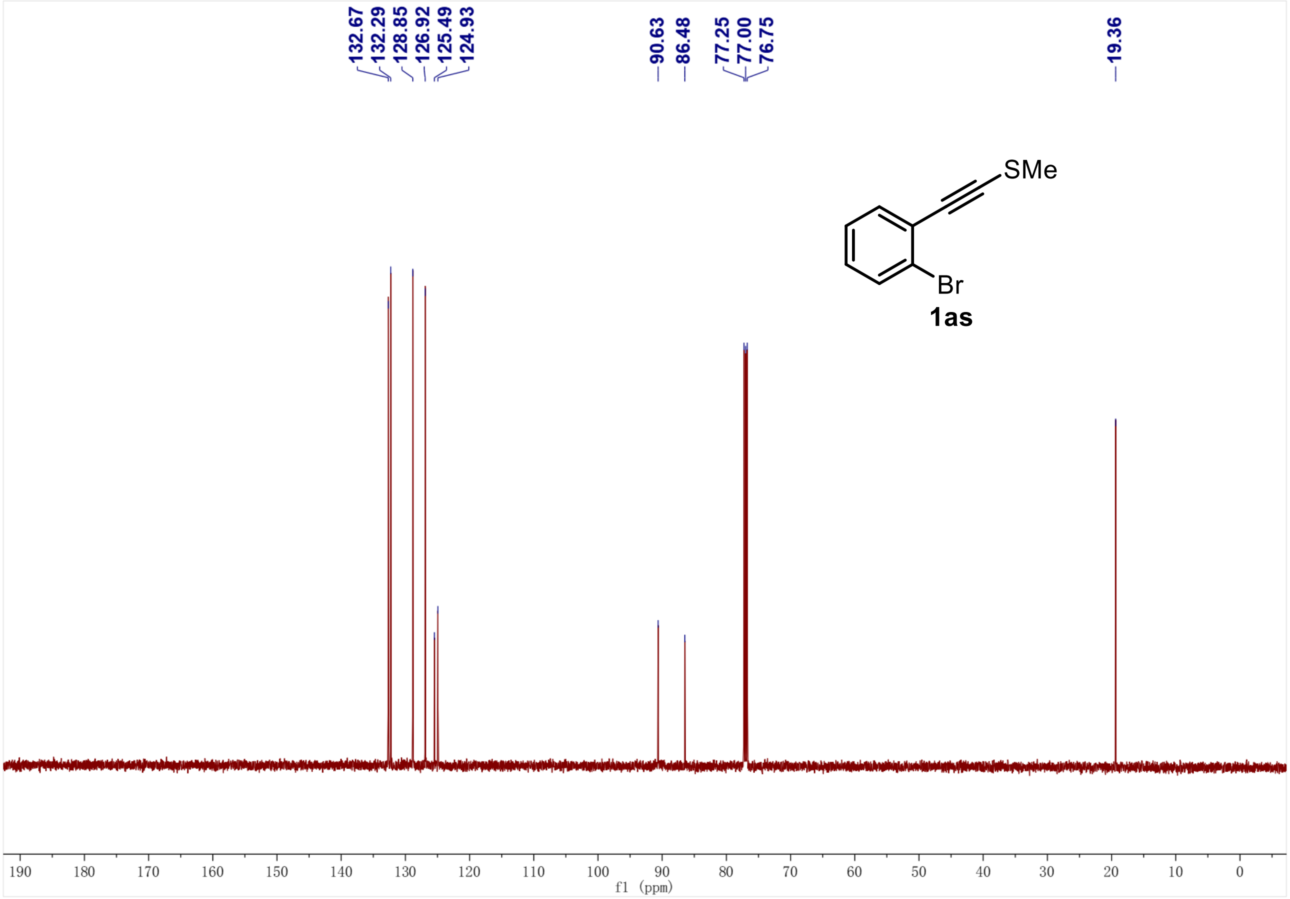

1au; ^{1}H NMR (400 MHz, $CDCl_3$); ^{13}C NMR (101 MHz, $CDCl_3$)

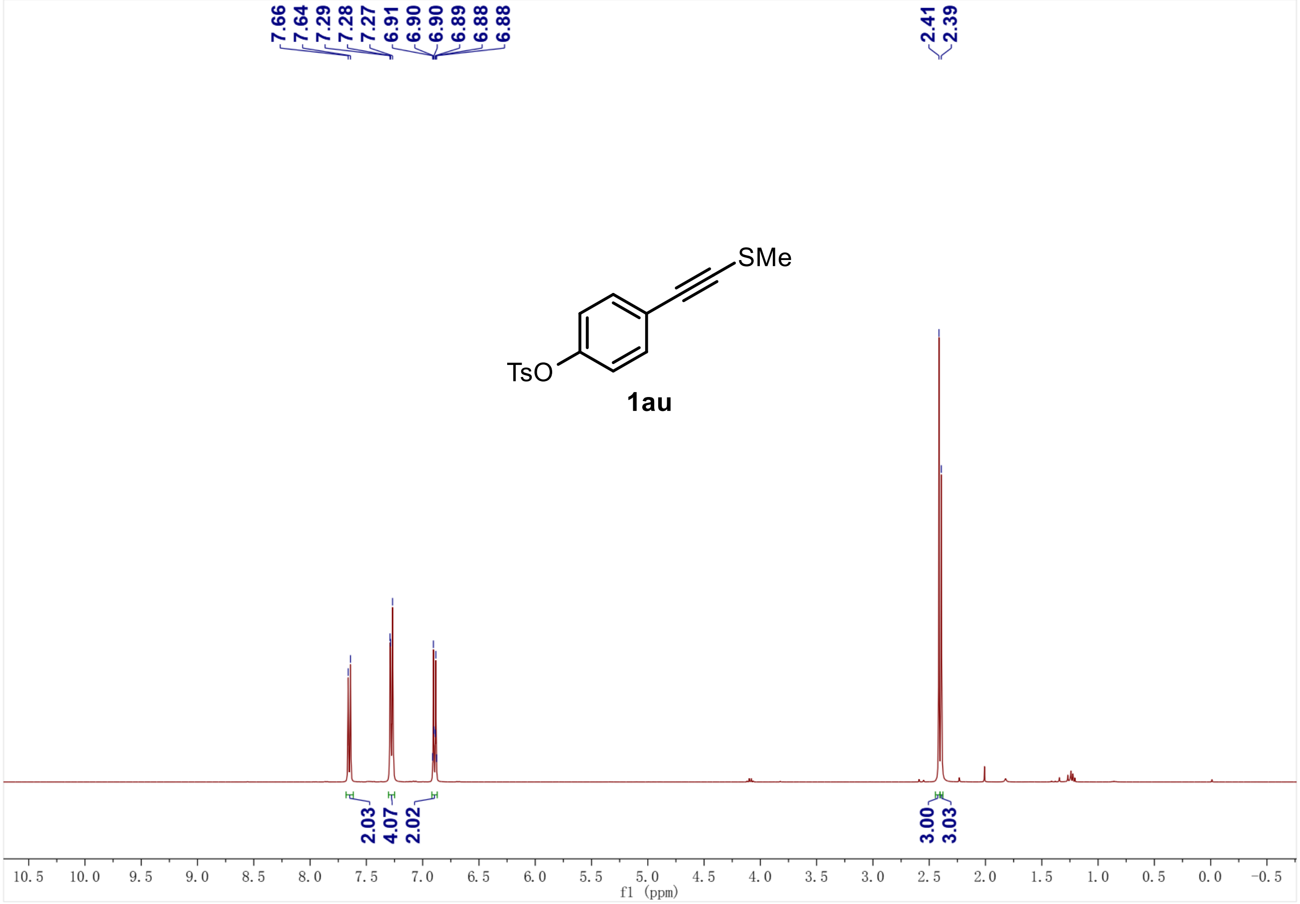

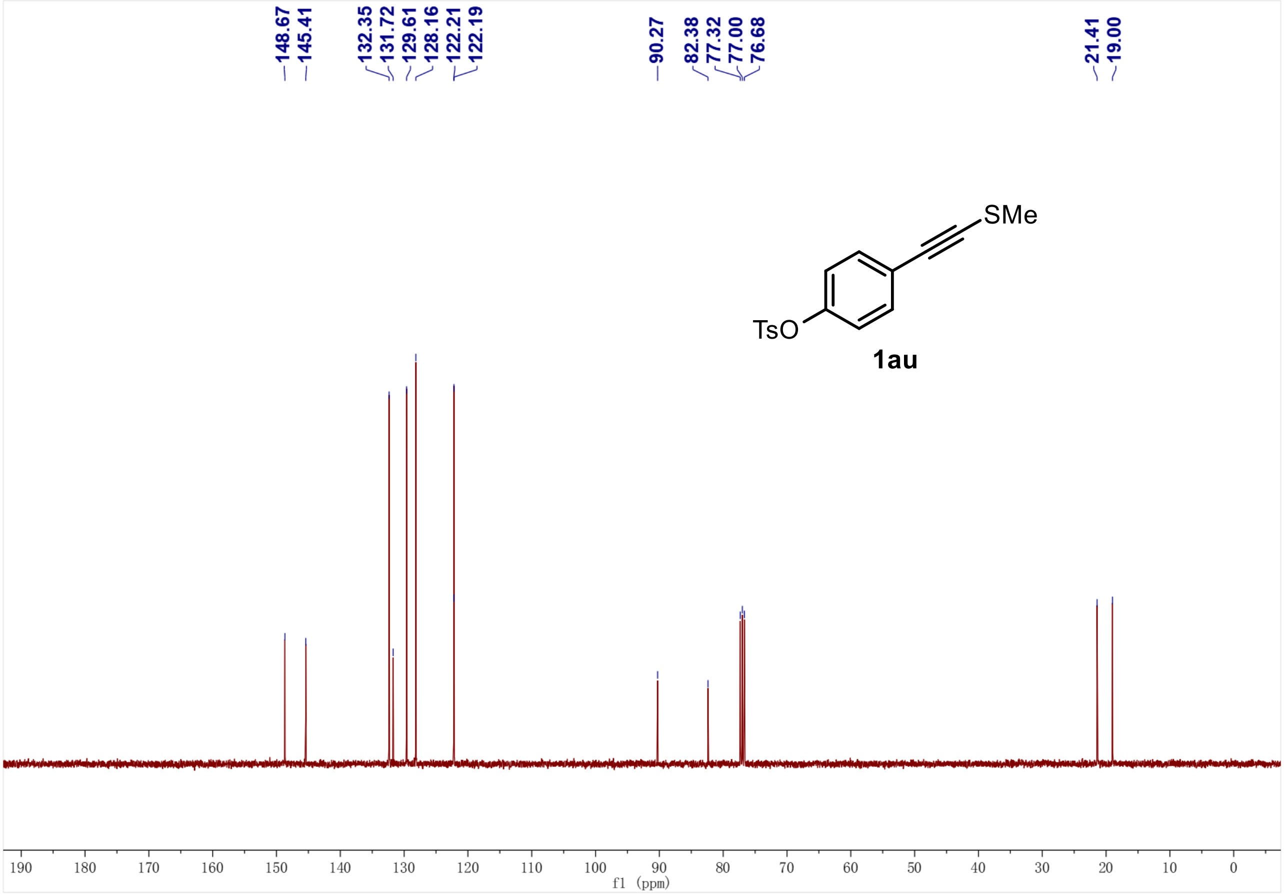

1av; ^{1}H NMR (400 MHz, $CDCl_3$); ^{13}C NMR (101 MHz, $CDCl_3$)

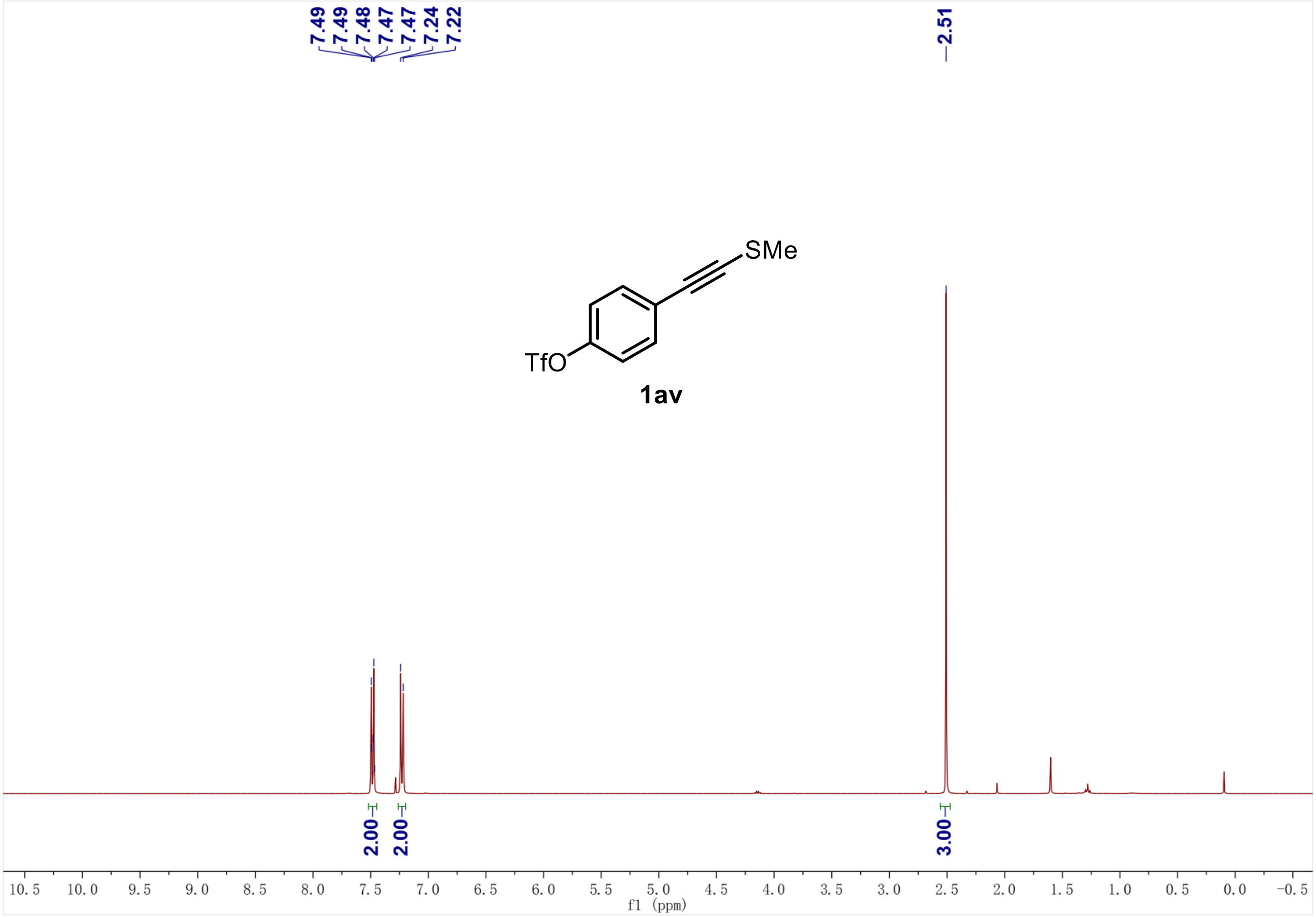

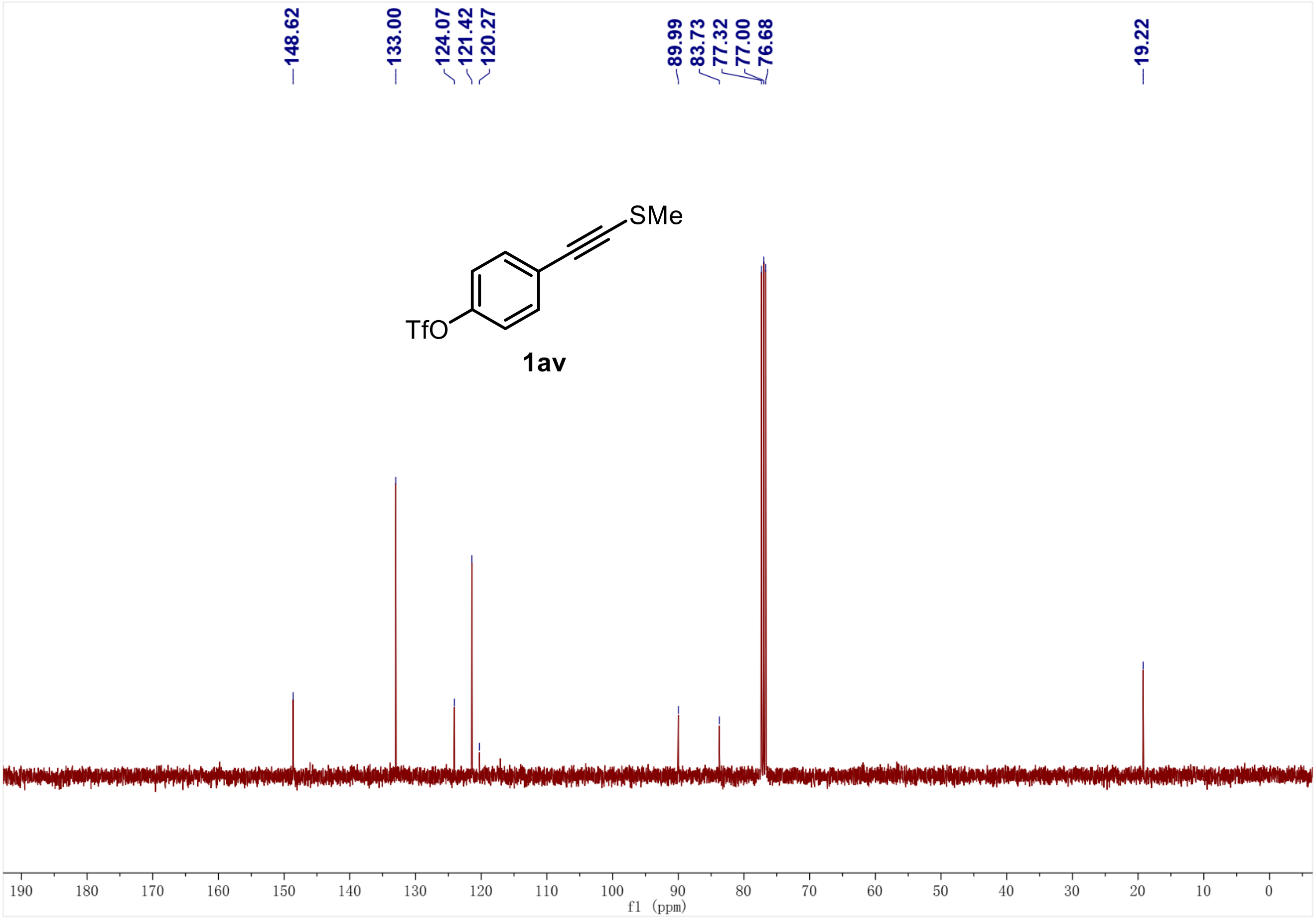

1av; ^{19}F NMR (376 MHz, $CDCl_3$)

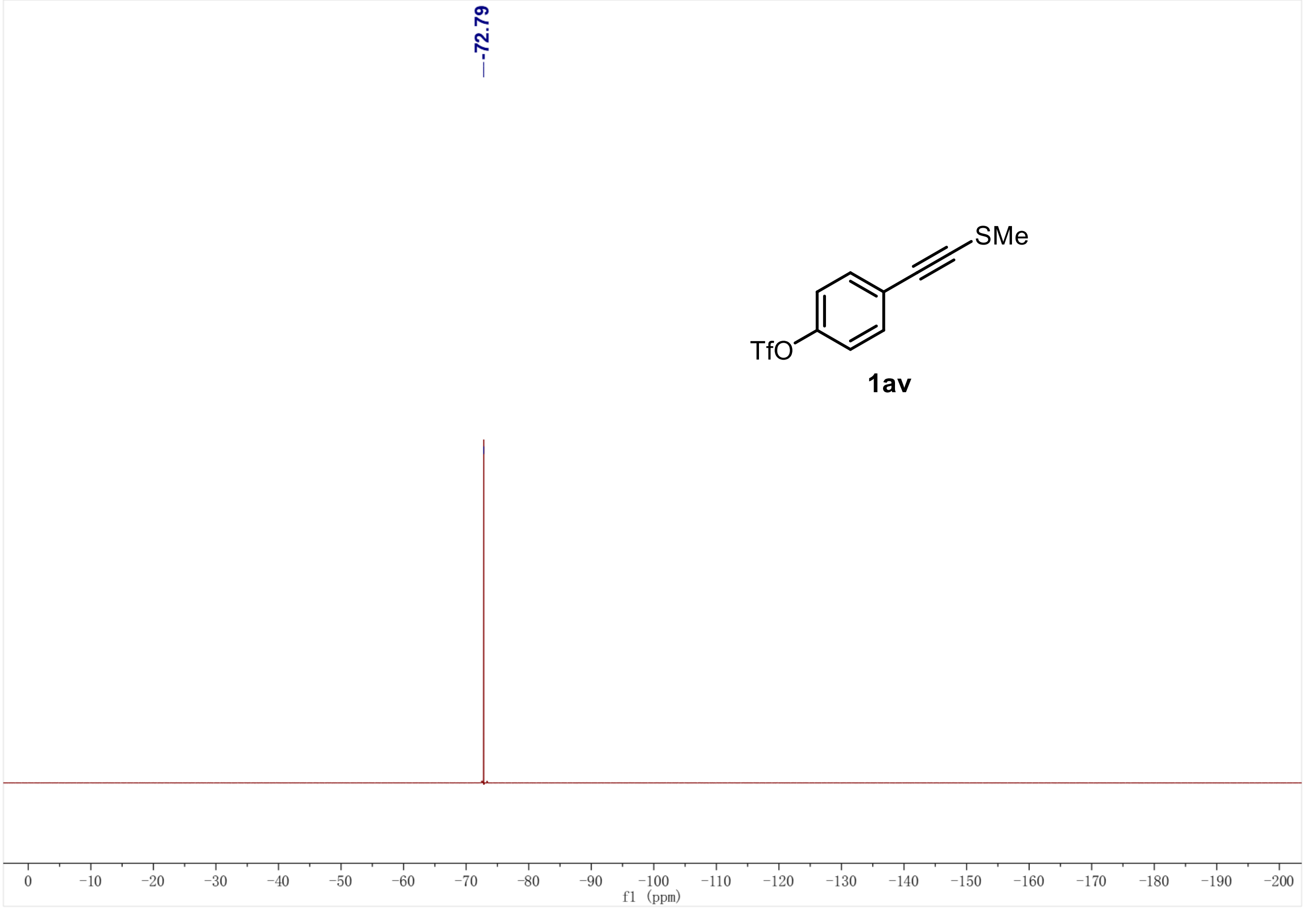

1aw; ^{1}H NMR (500 MHz, $CDCl_3$); ^{13}C NMR (126 MHz, $CDCl_3$)

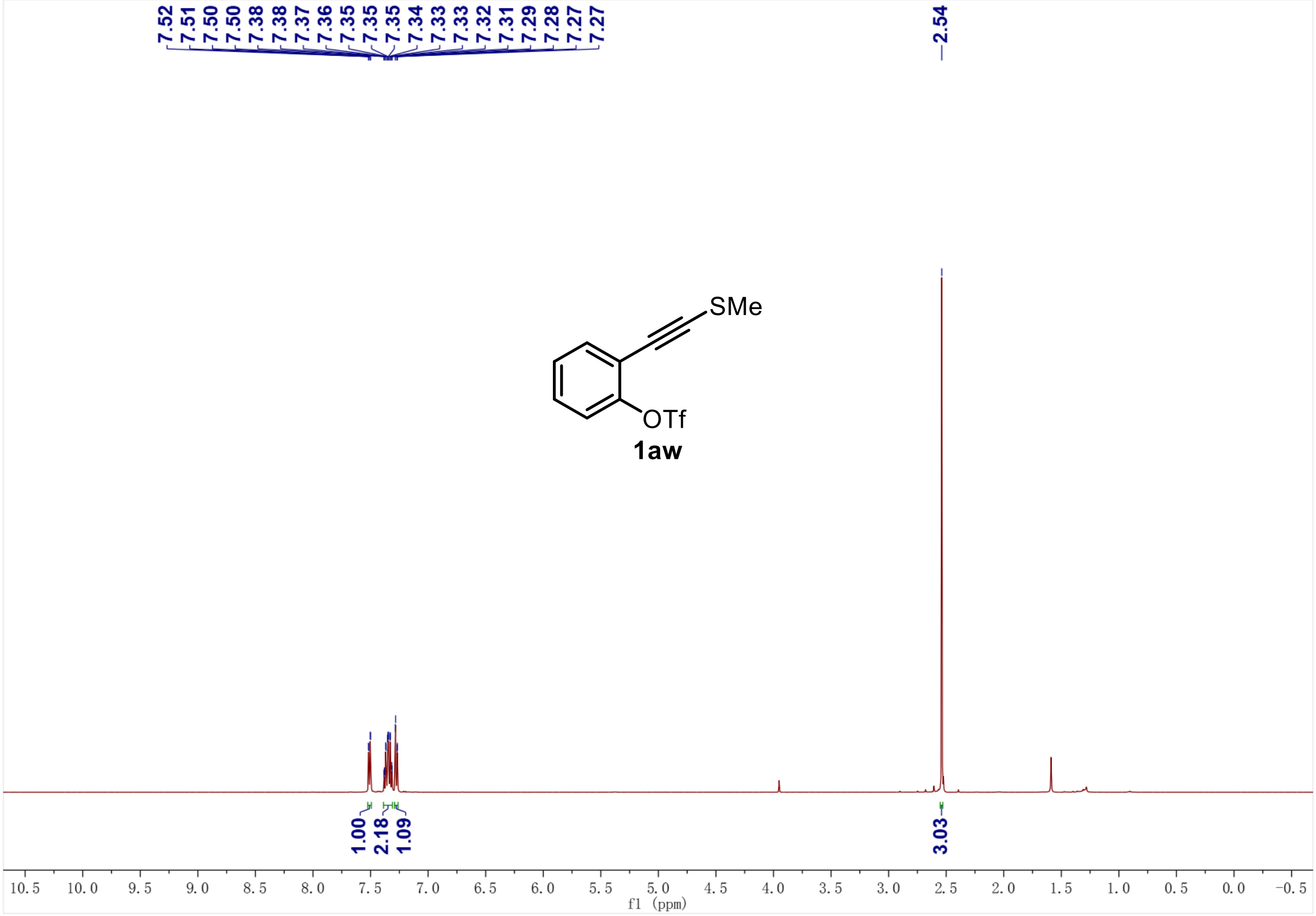

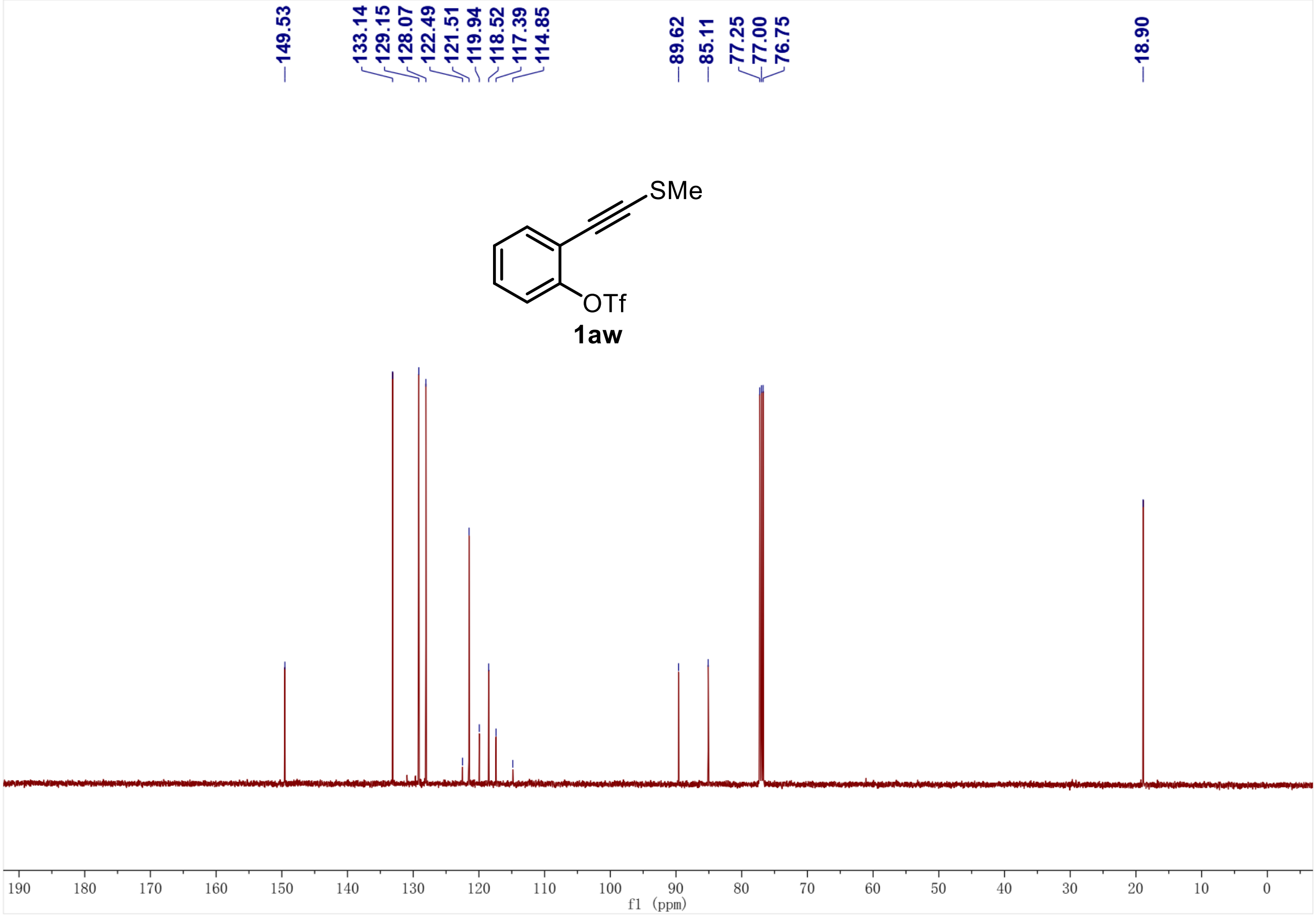

1aw; ^{19}F NMR (471 MHz, $CDCl_3$)

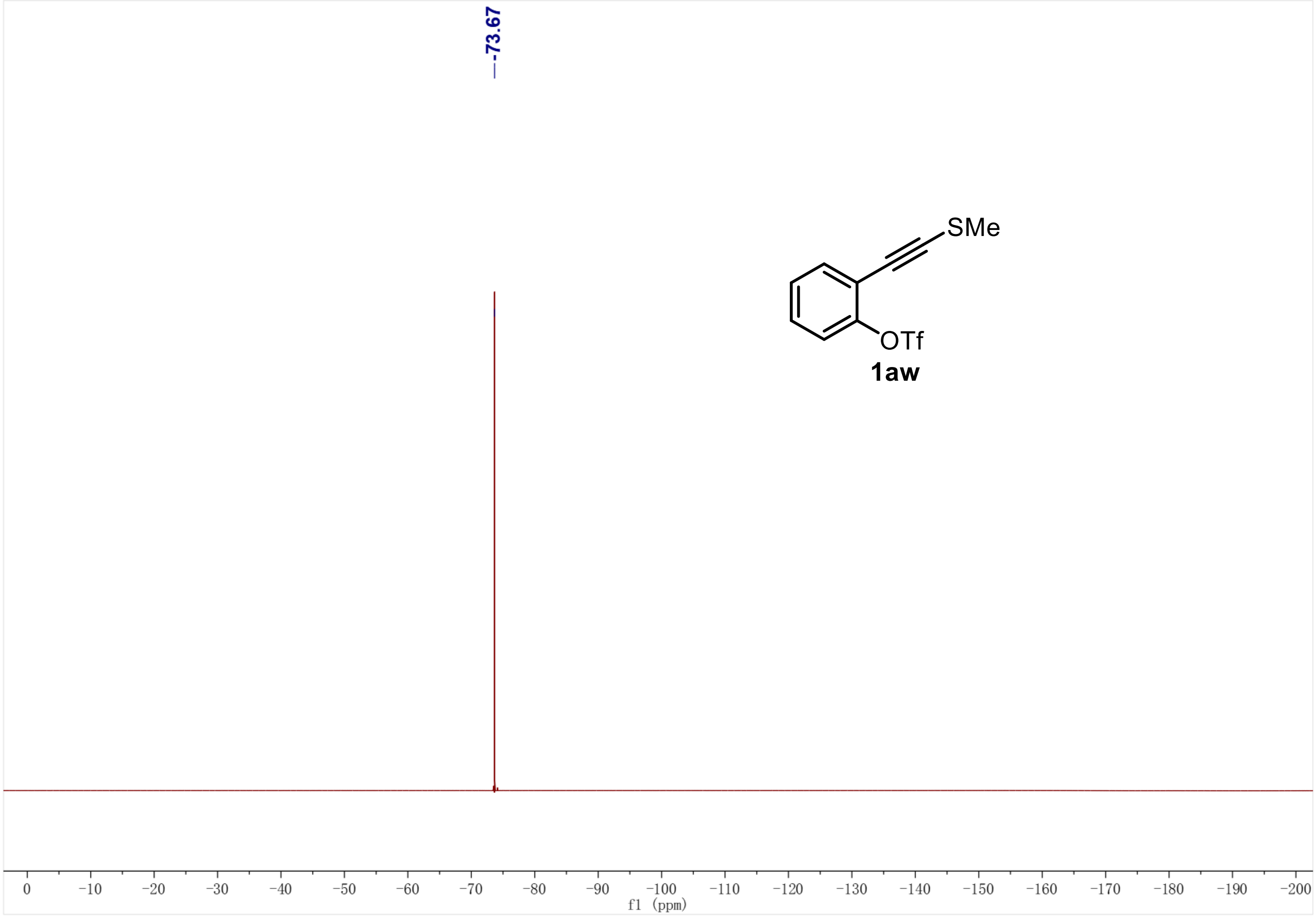

1ax; ^{1}H NMR (500 MHz, $CDCl_3$); ^{13}C NMR (126 MHz, $CDCl_3$)

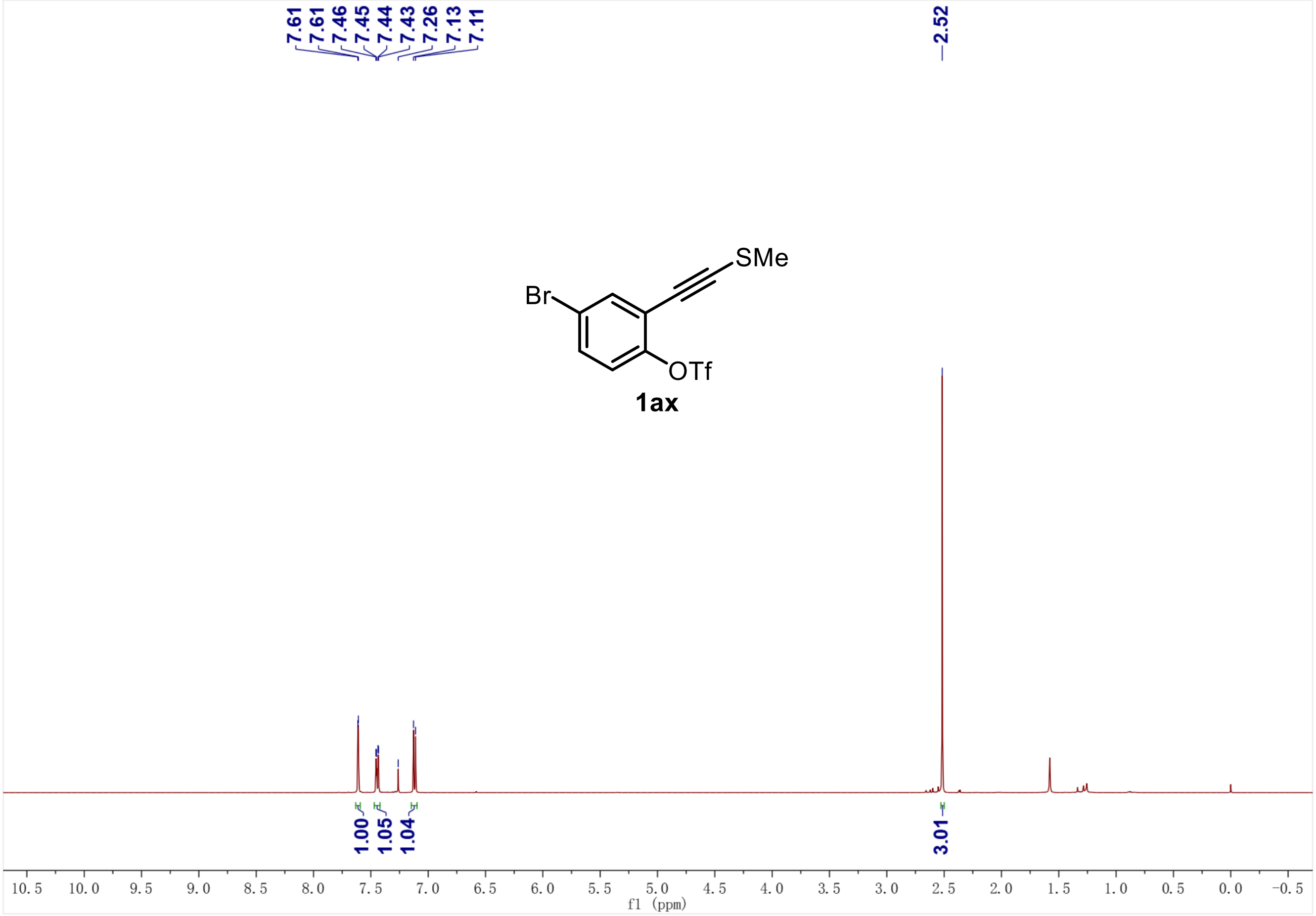

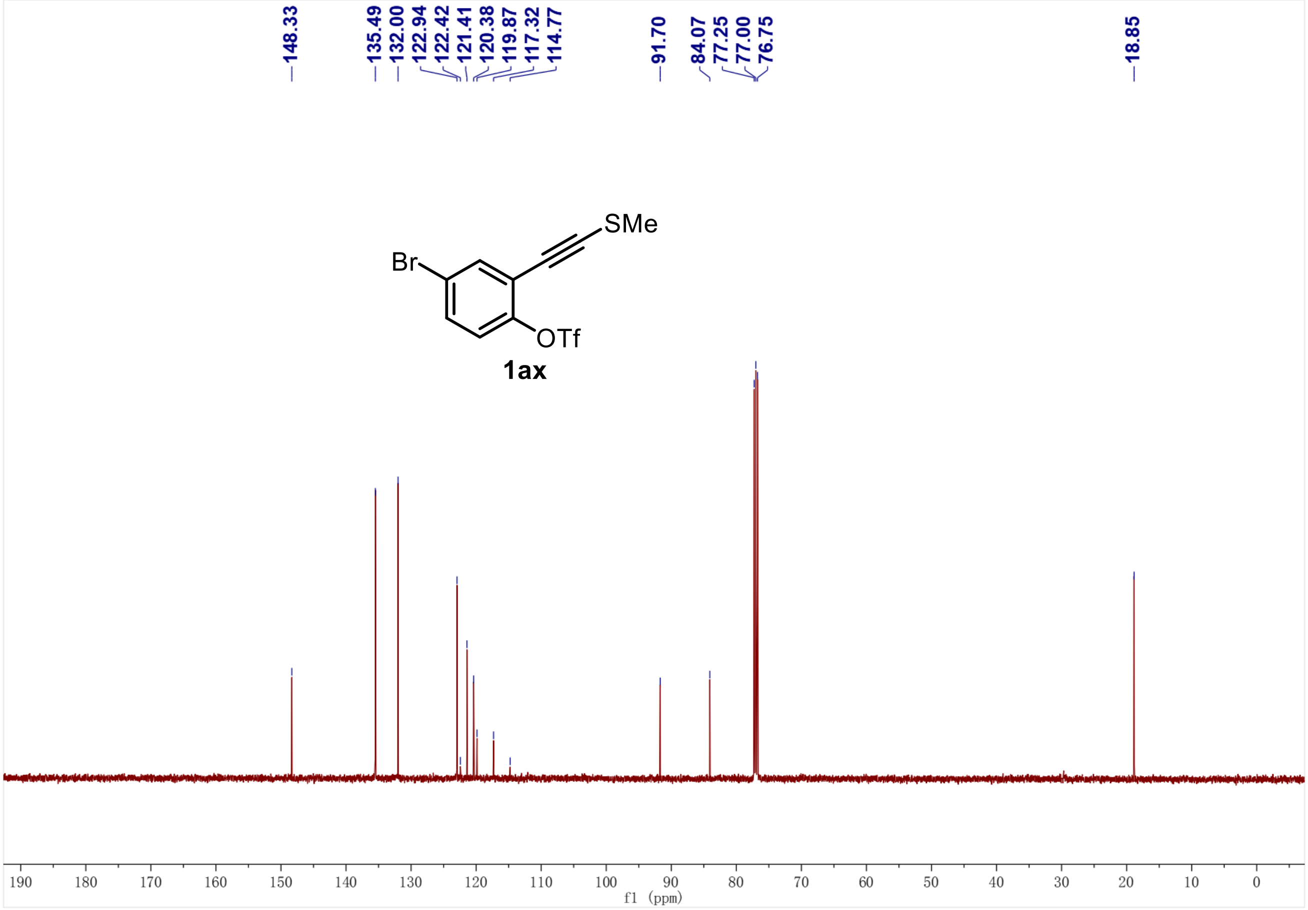

1ax; ^{19}F NMR (376 MHz, $CDCl_3$)

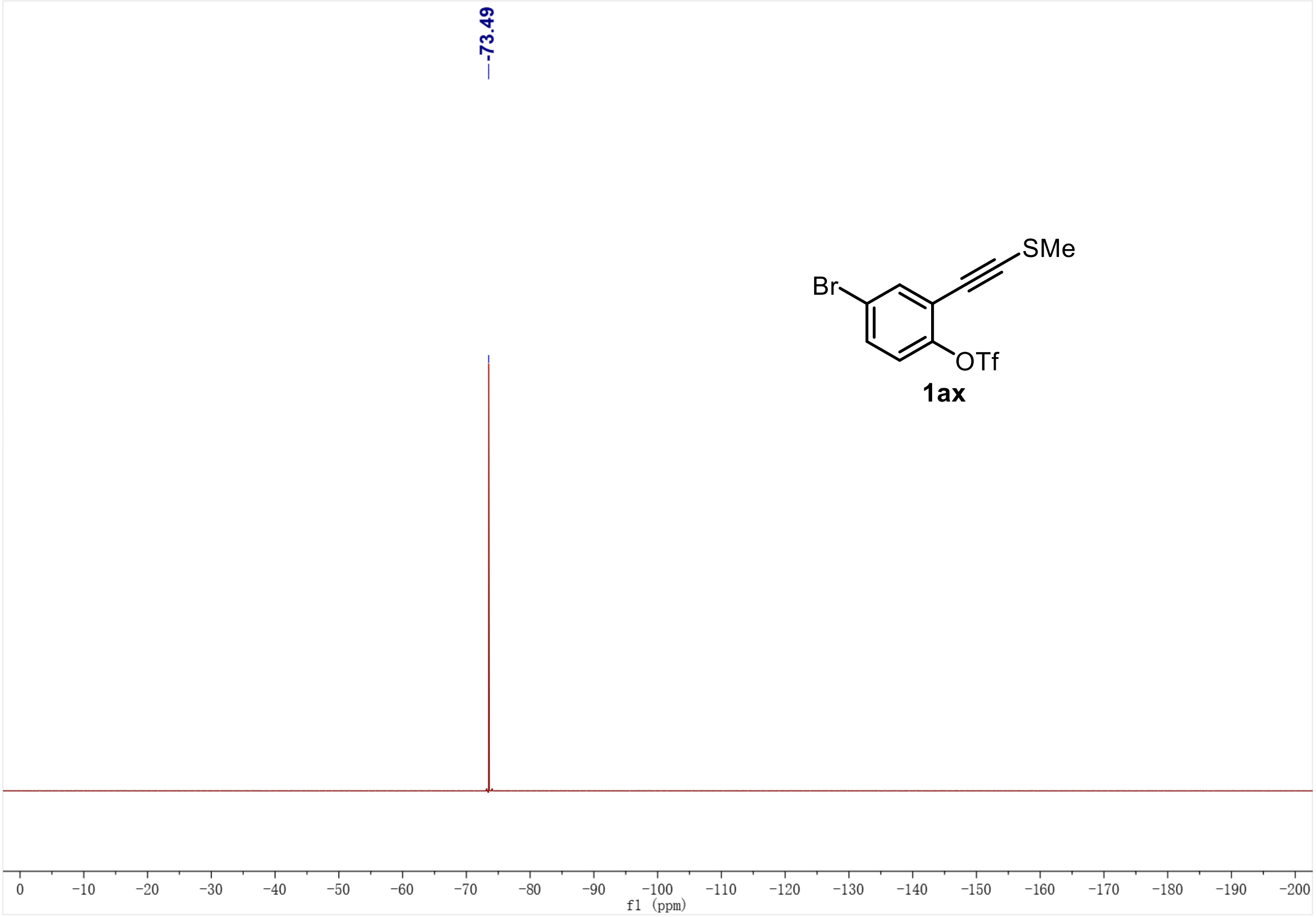

1ay; ^{1}H NMR (500 MHz, $CDCl_3$); ^{13}C NMR (126 MHz, $CDCl_3$)

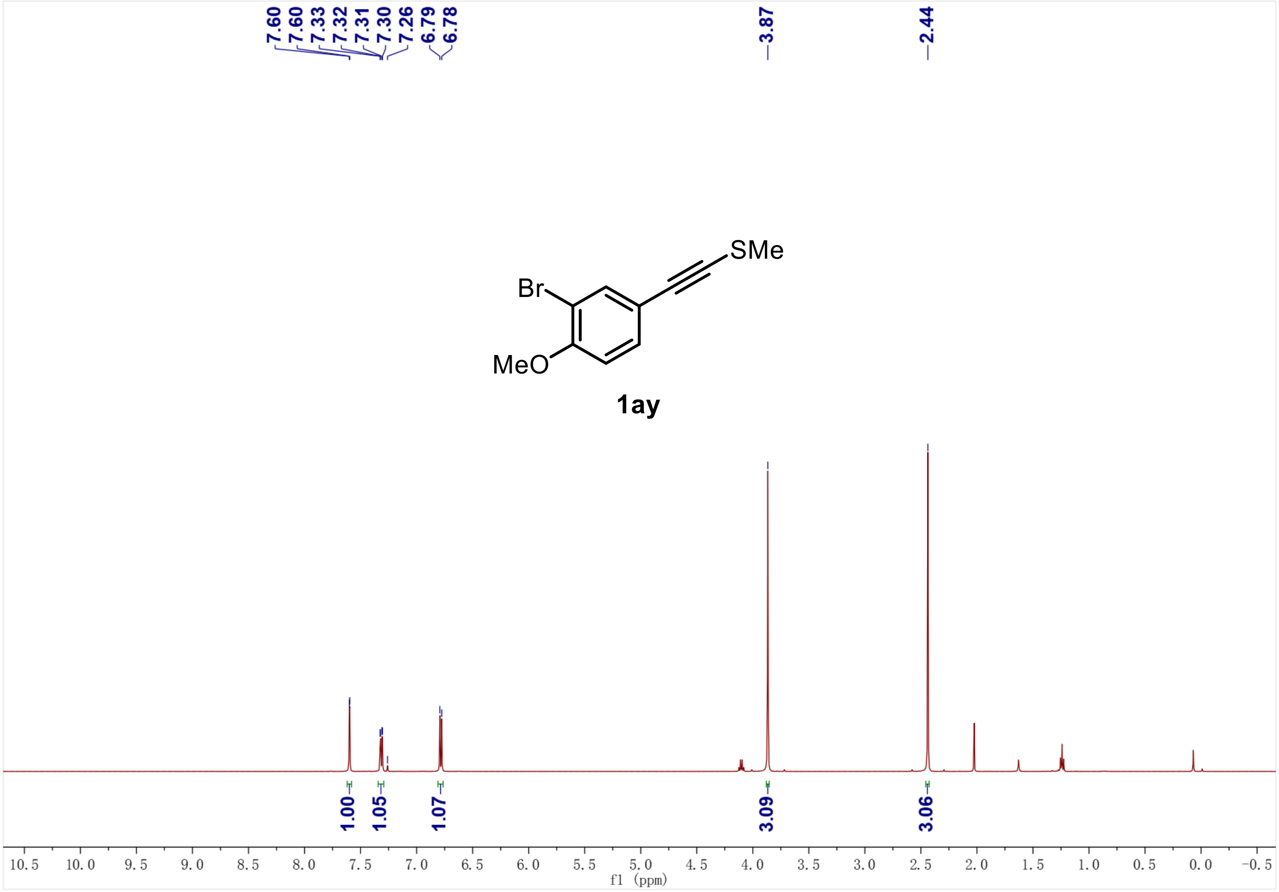

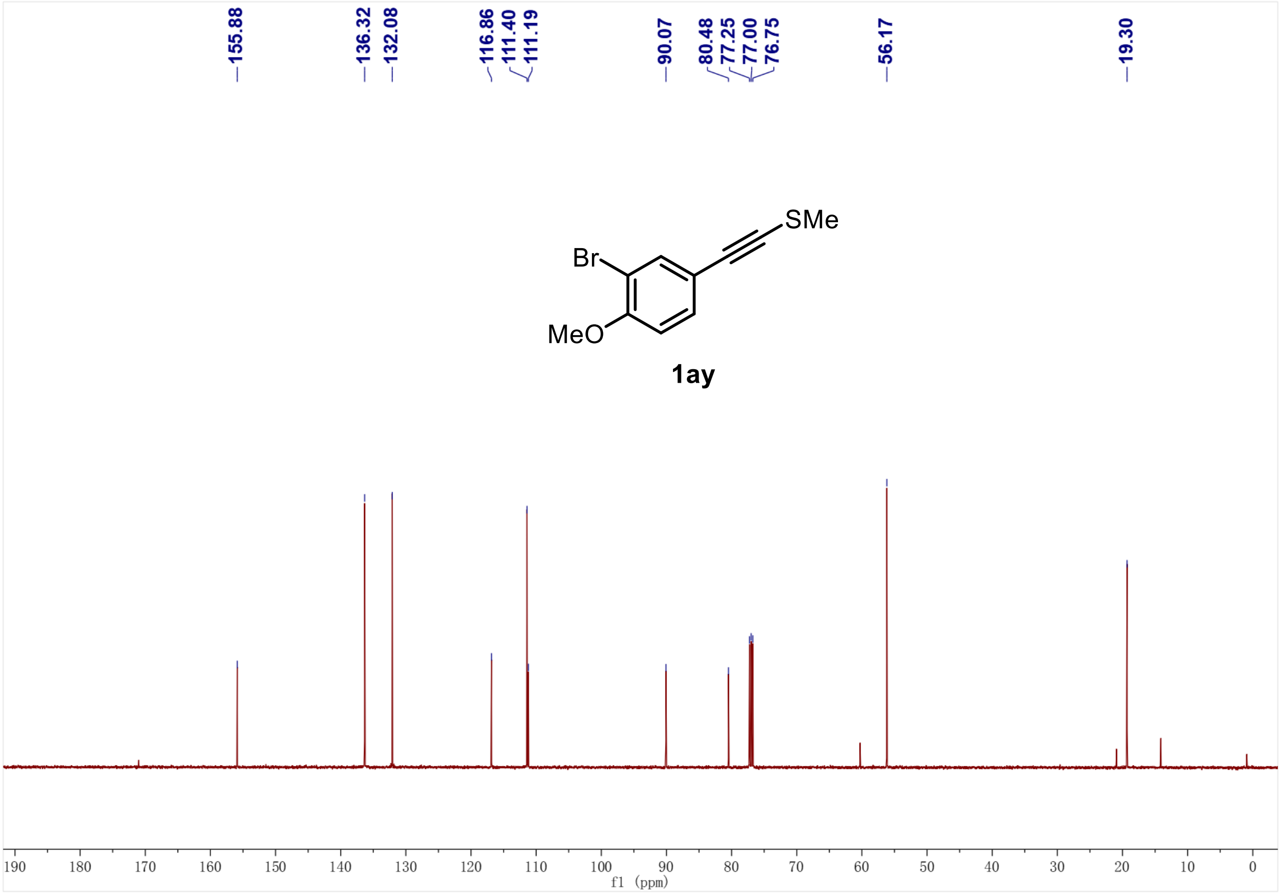

1az; ^{1}H NMR (400 MHz, $CDCl_3$); ^{13}C NMR (101 MHz, $CDCl_3$)

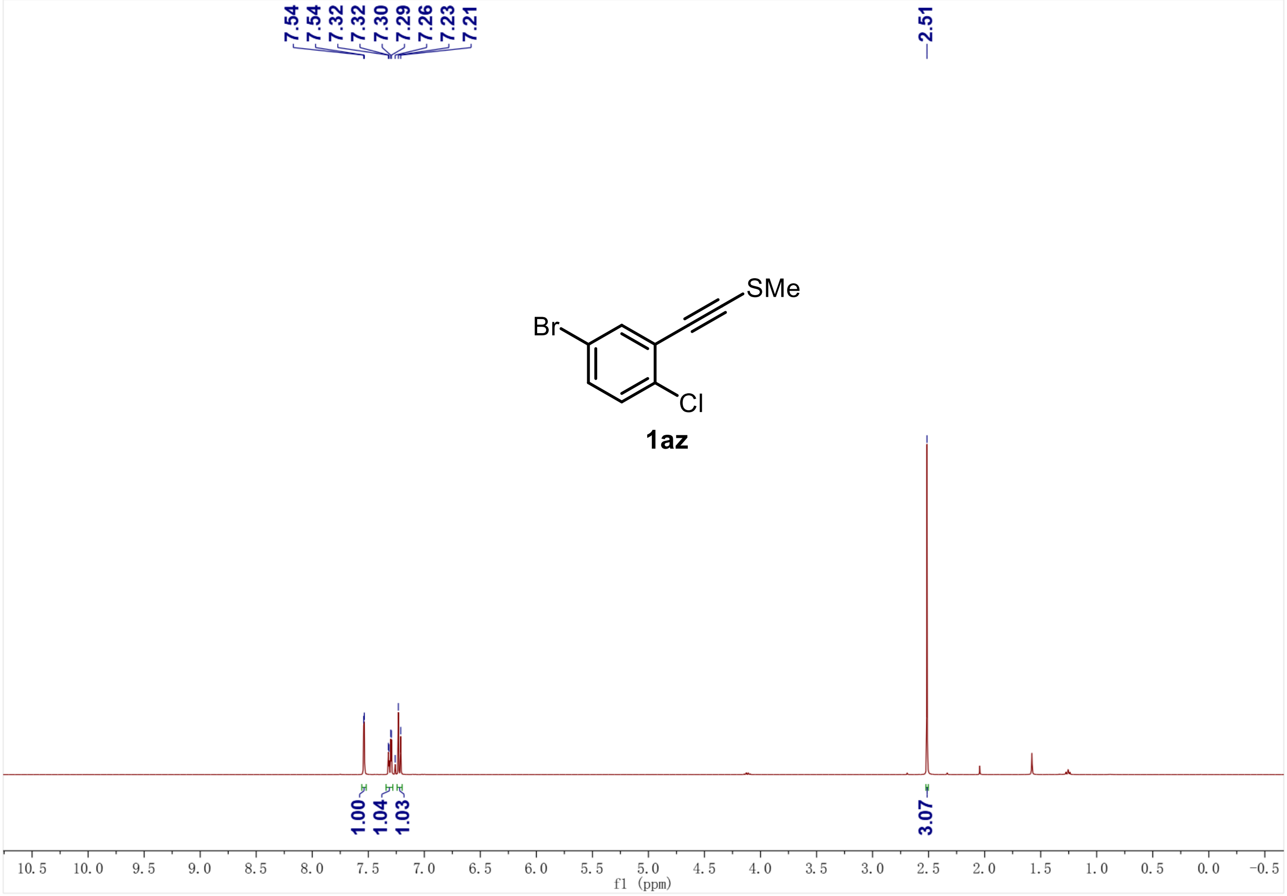

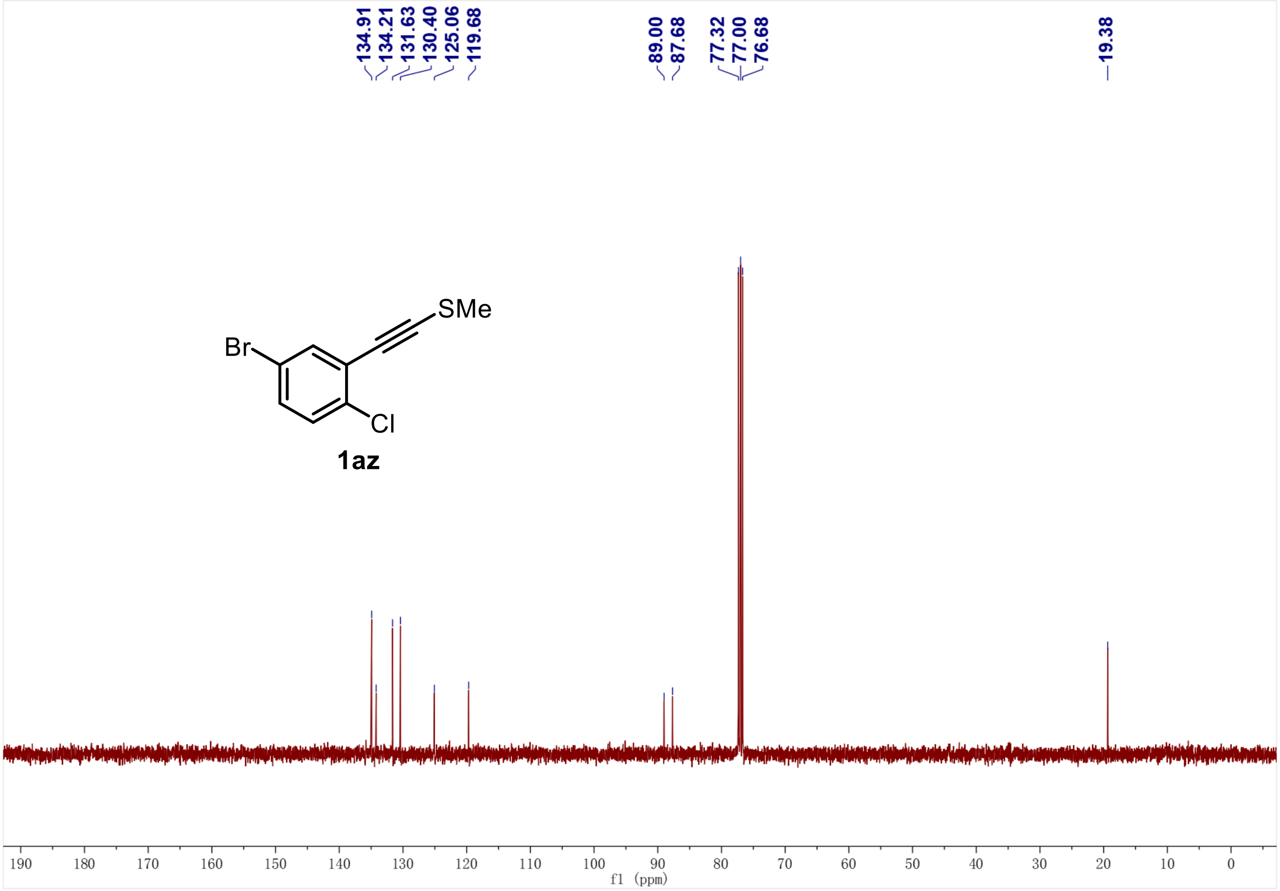

1ba; ^{1}H NMR (500 MHz, $CDCl_3$); ^{13}C NMR (126 MHz, $CDCl_3$)

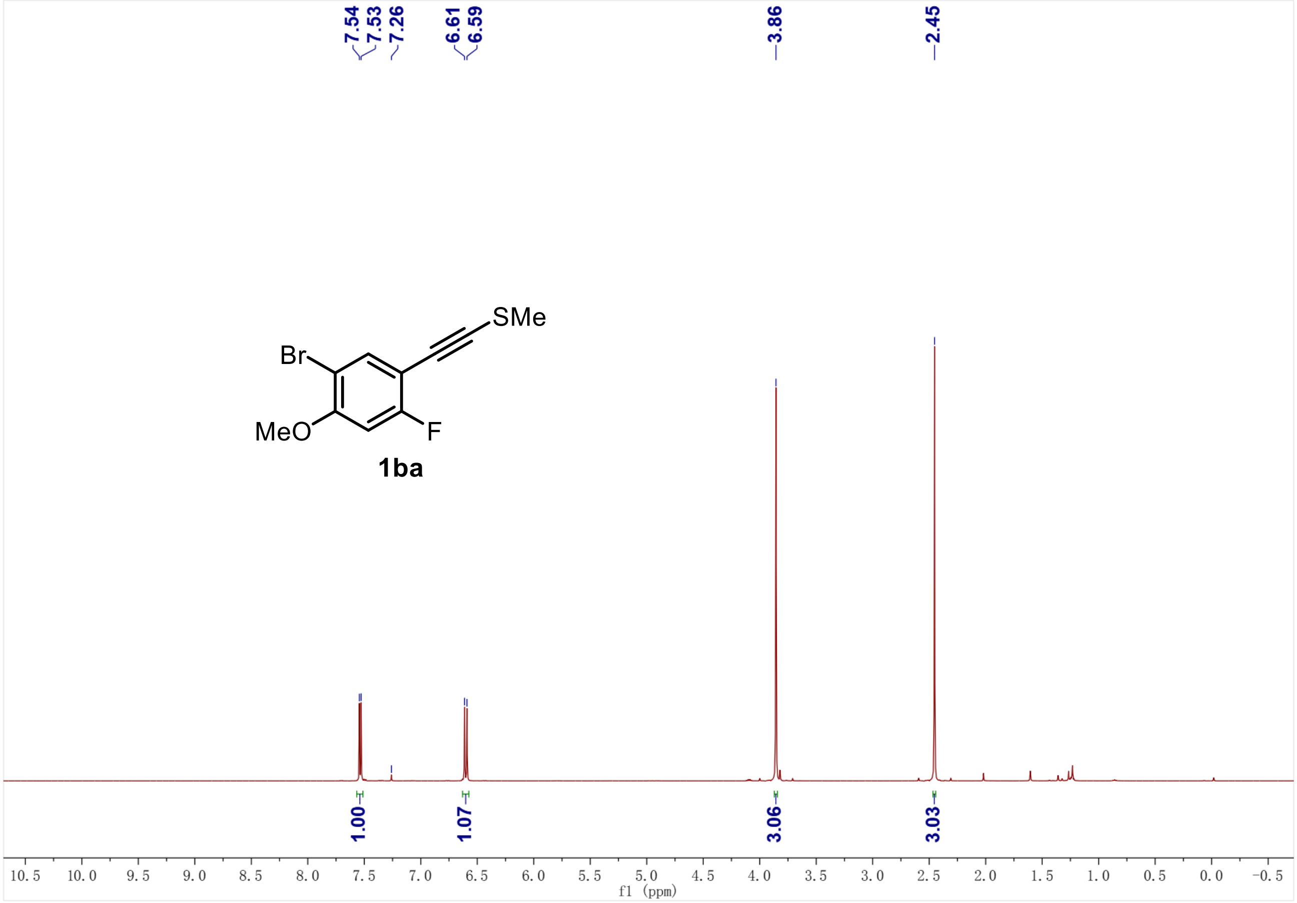

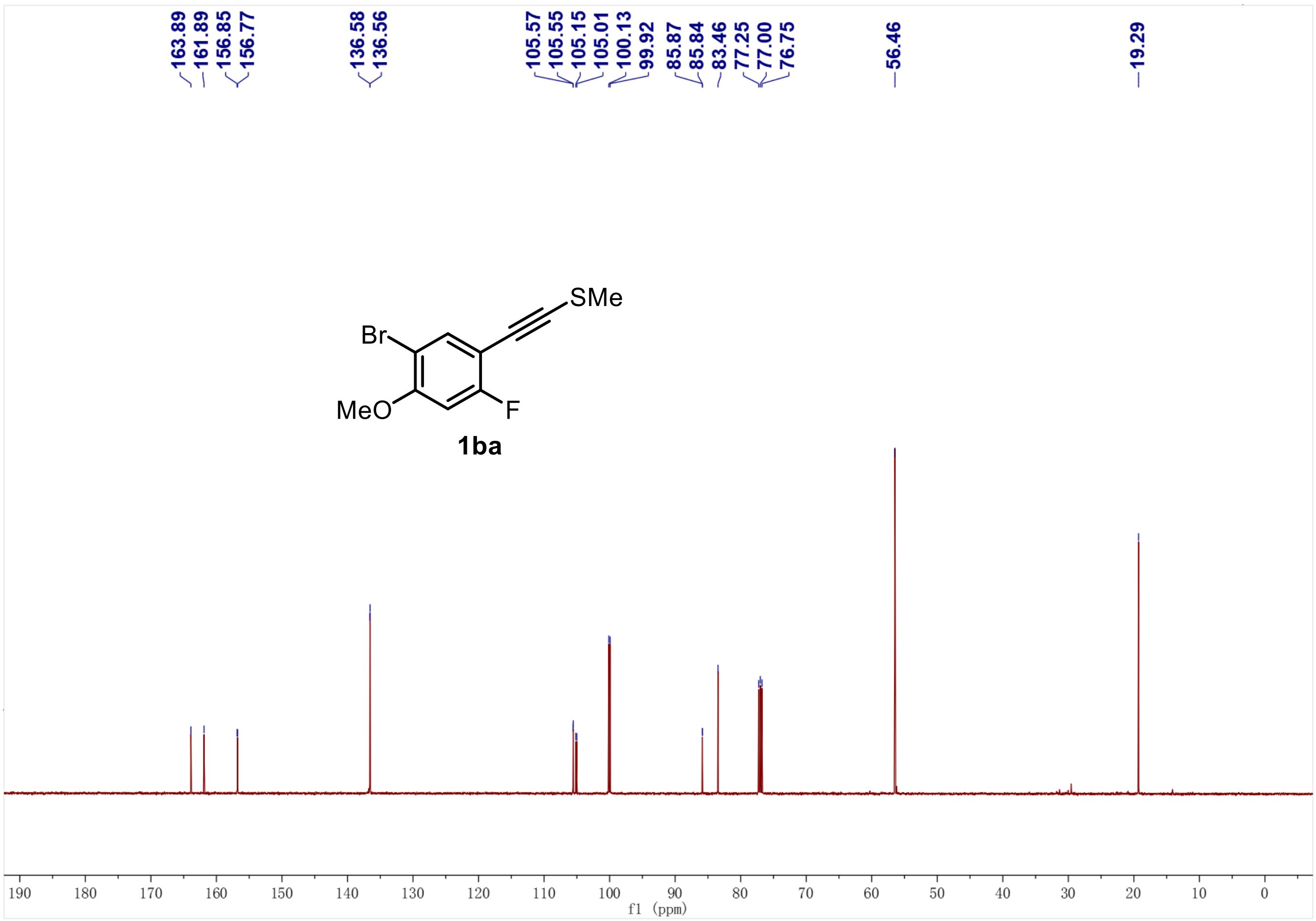

1ba; ^{19}F NMR (471 MHz, $CDCl_3$)

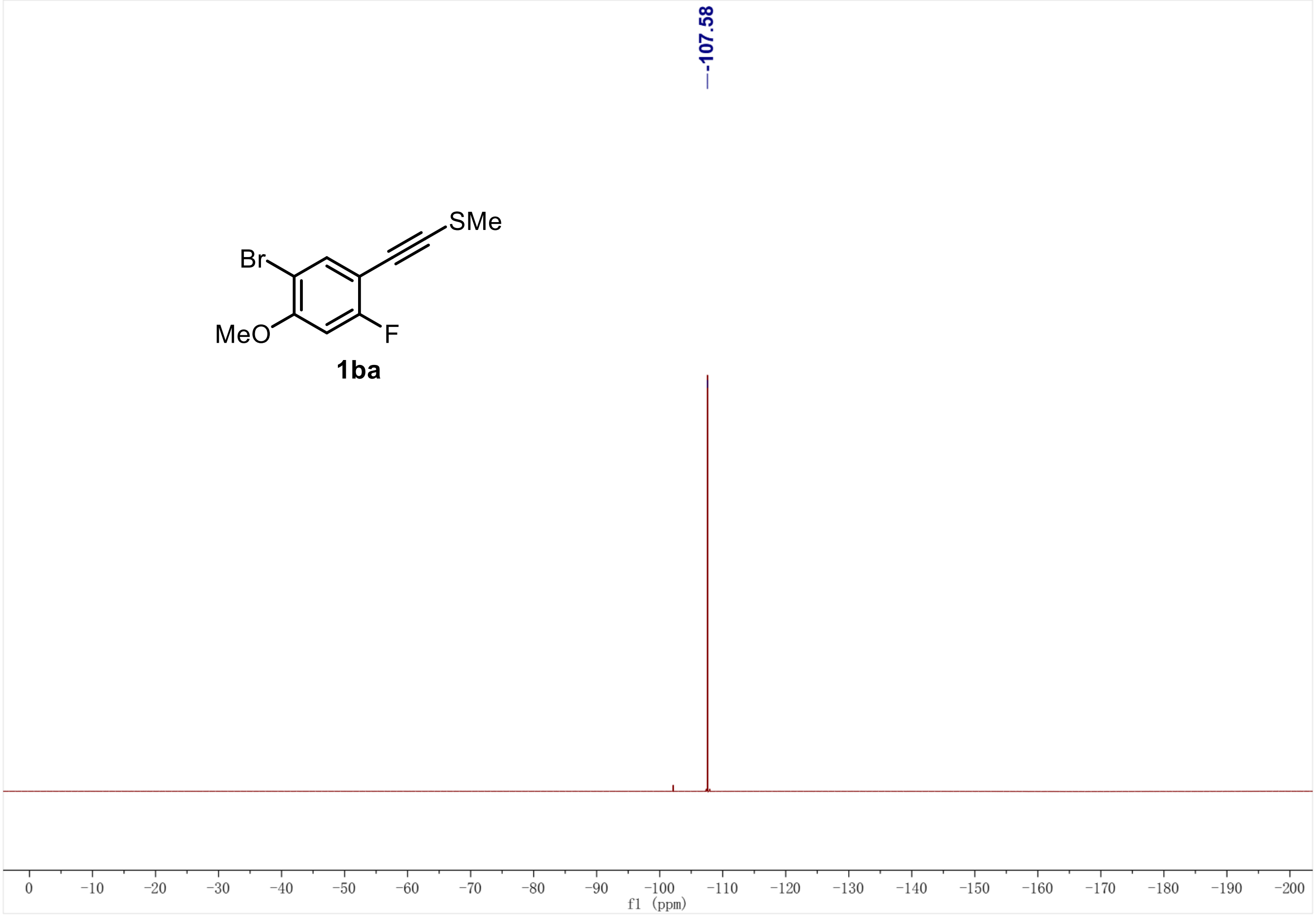

1bb; ^{1}H NMR (400 MHz, $CDCl_3$); ^{13}C NMR (126 MHz, $CDCl_3$)

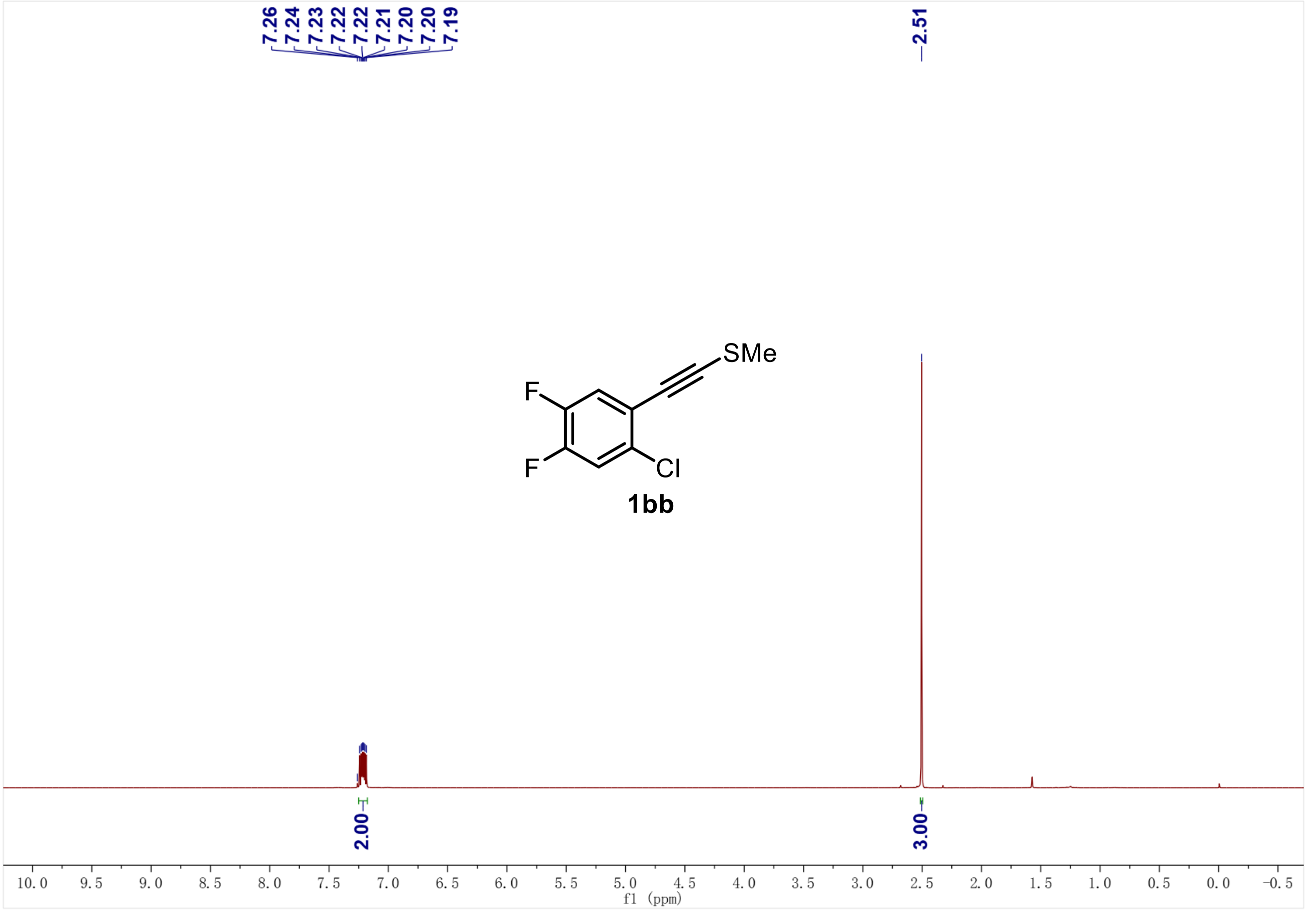

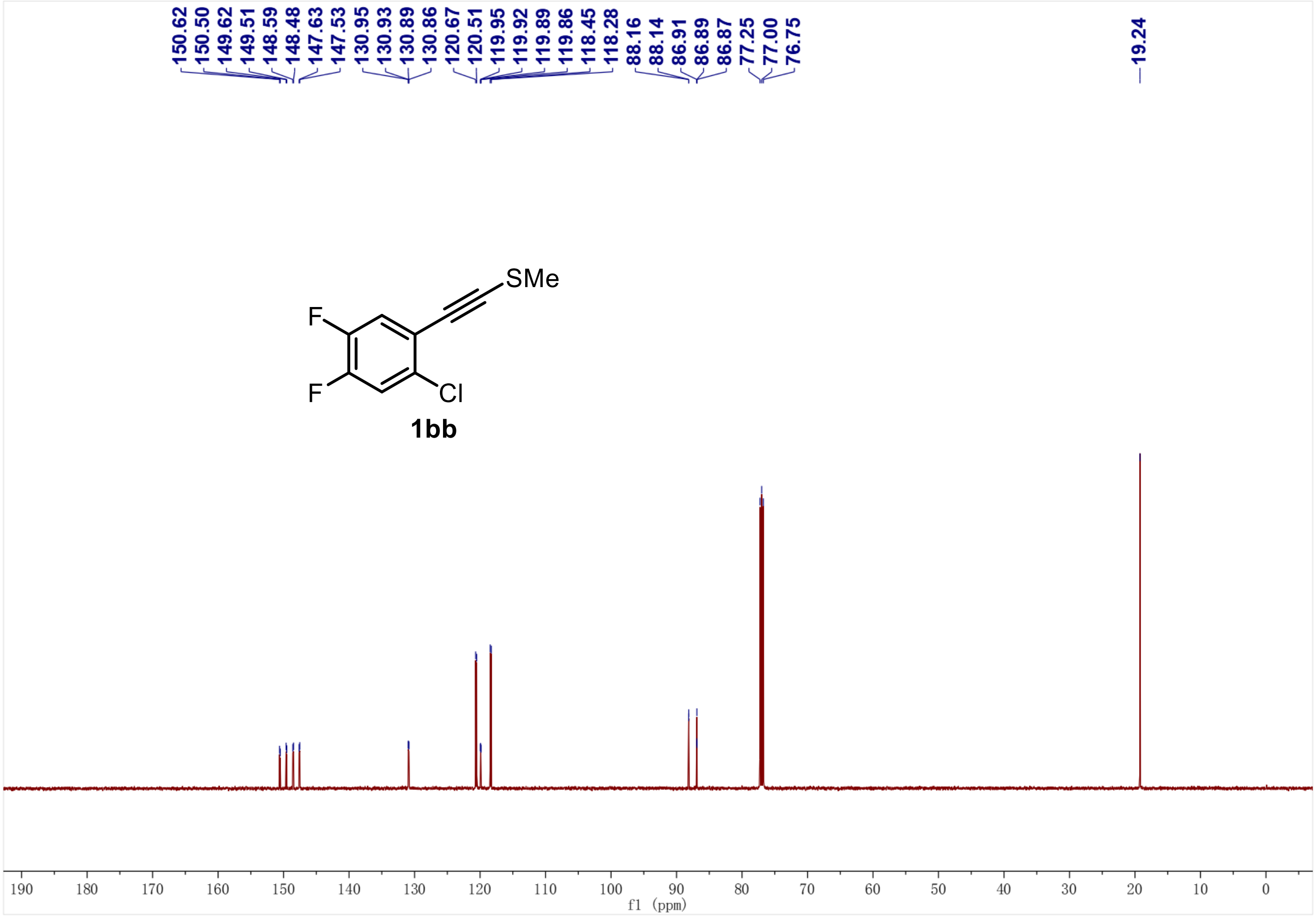

1bb; ^{19}F NMR (376 MHz, $CDCl_3$)

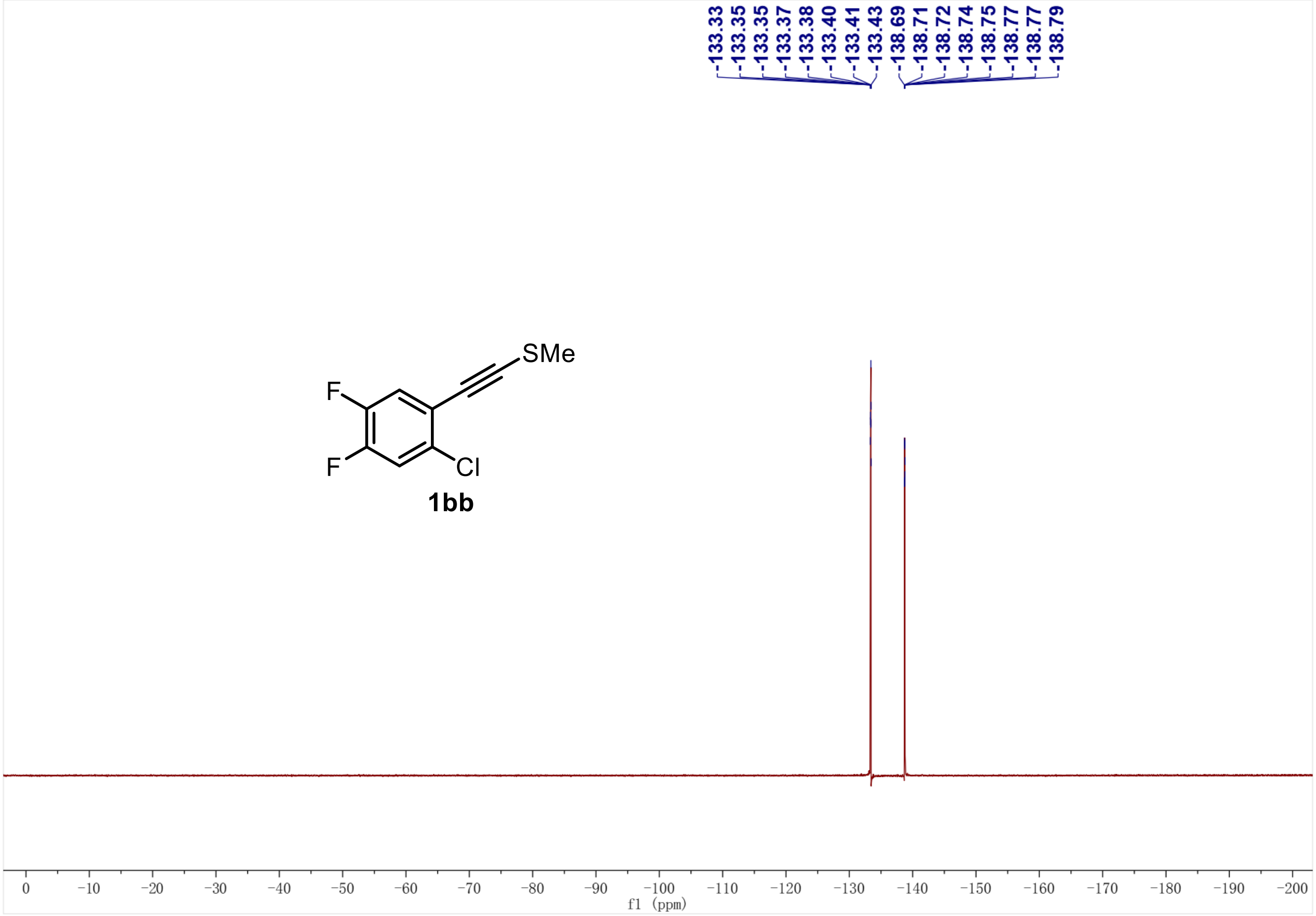

1bc; ^{1}H NMR (400 MHz, $CDCl_3$); ^{13}C NMR (101 MHz, $CDCl_3$)

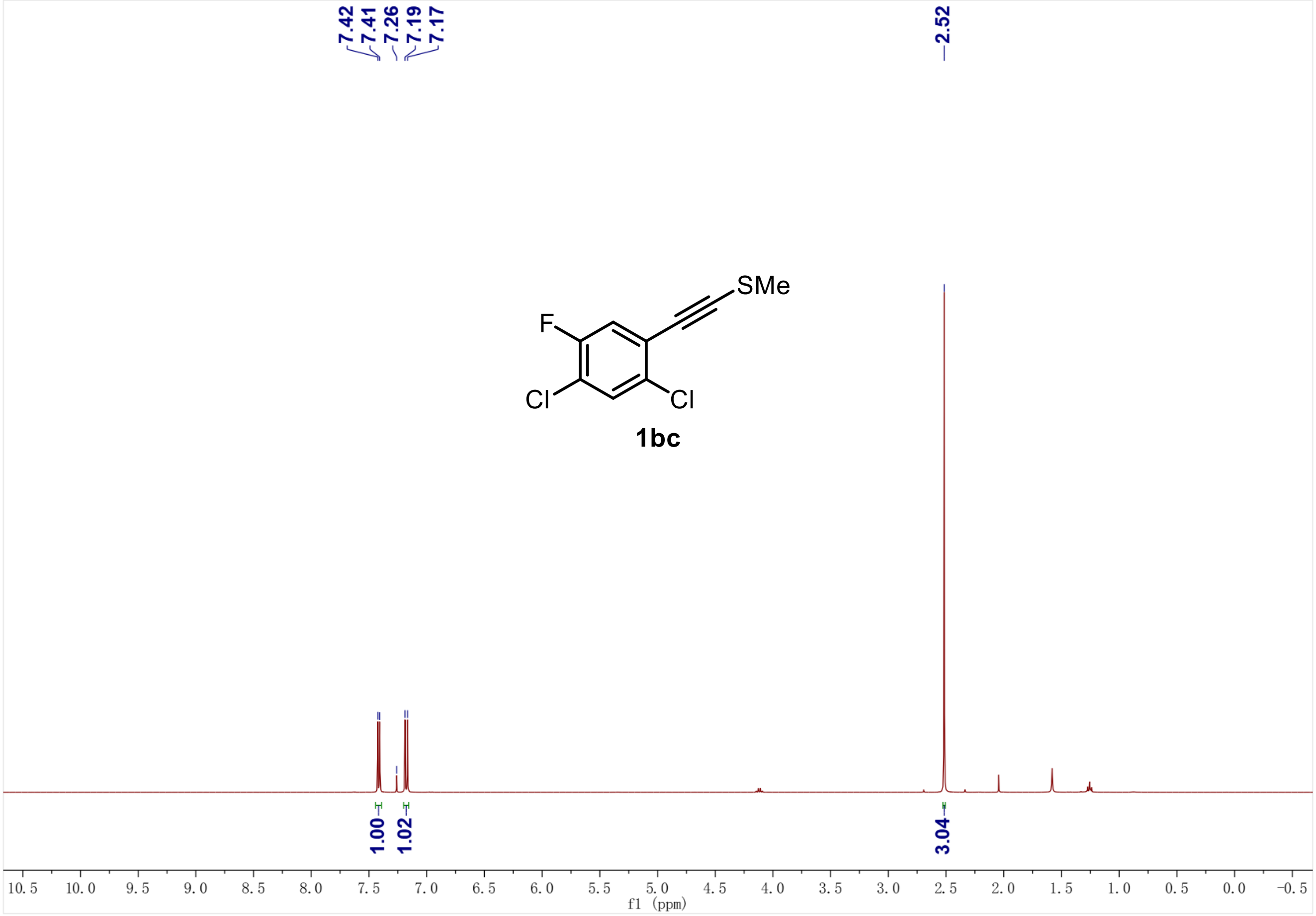

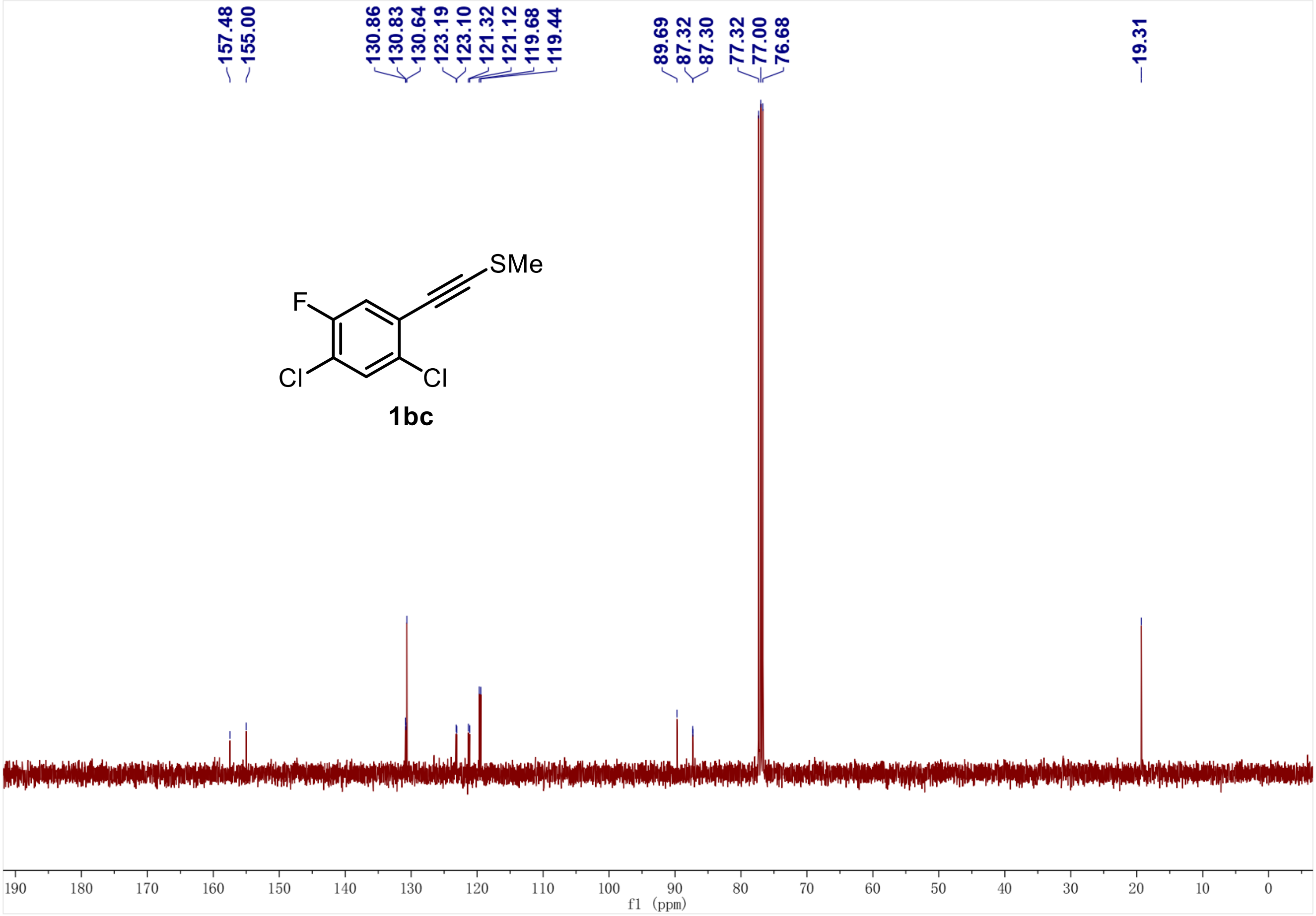

1bc; ^{19}F NMR (376 MHz, $CDCl_3$)

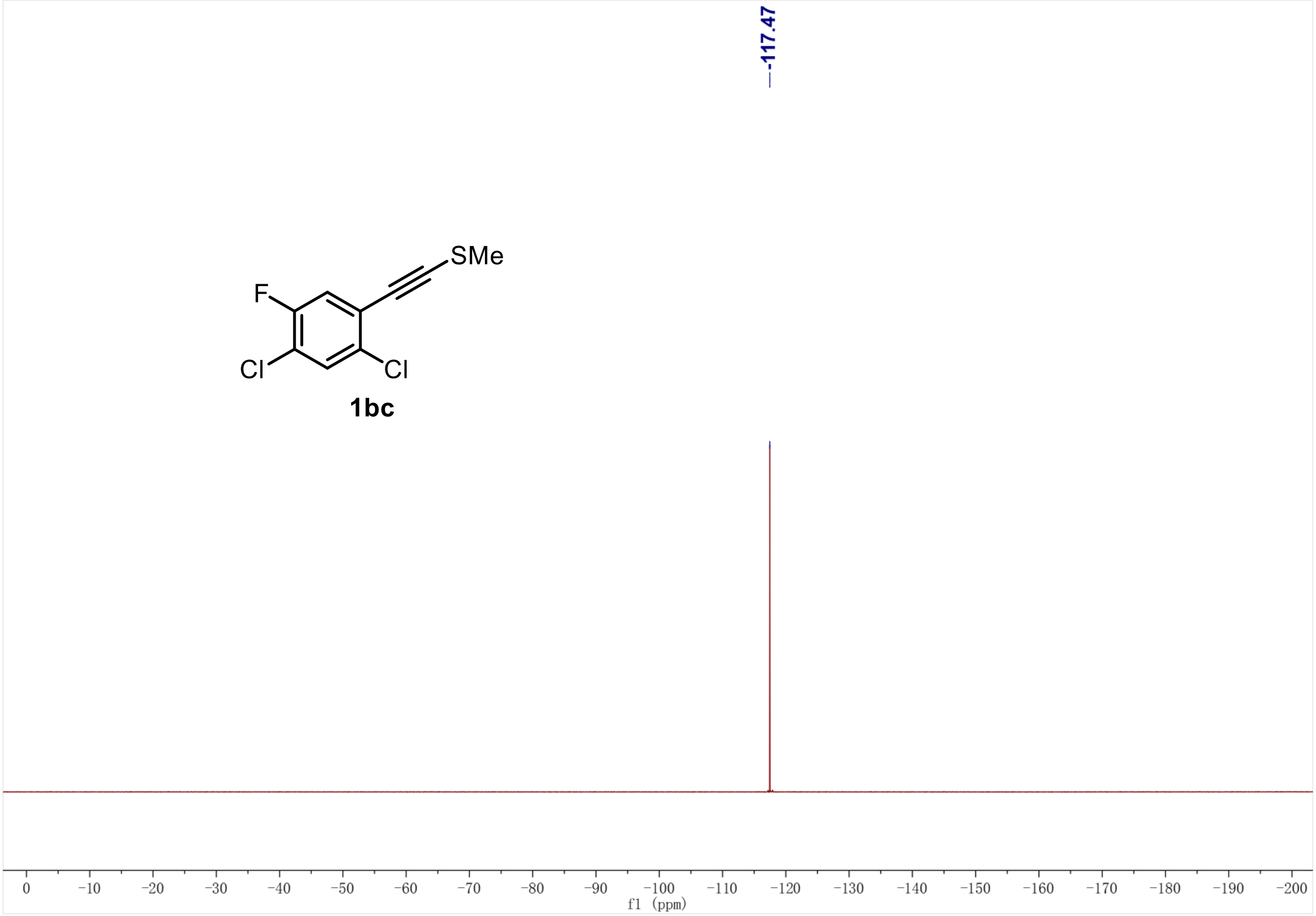

1be; ^1H NMR (500 MHz, $CDCl_3$); ^{13}C NMR (126 MHz, $CDCl_3$)

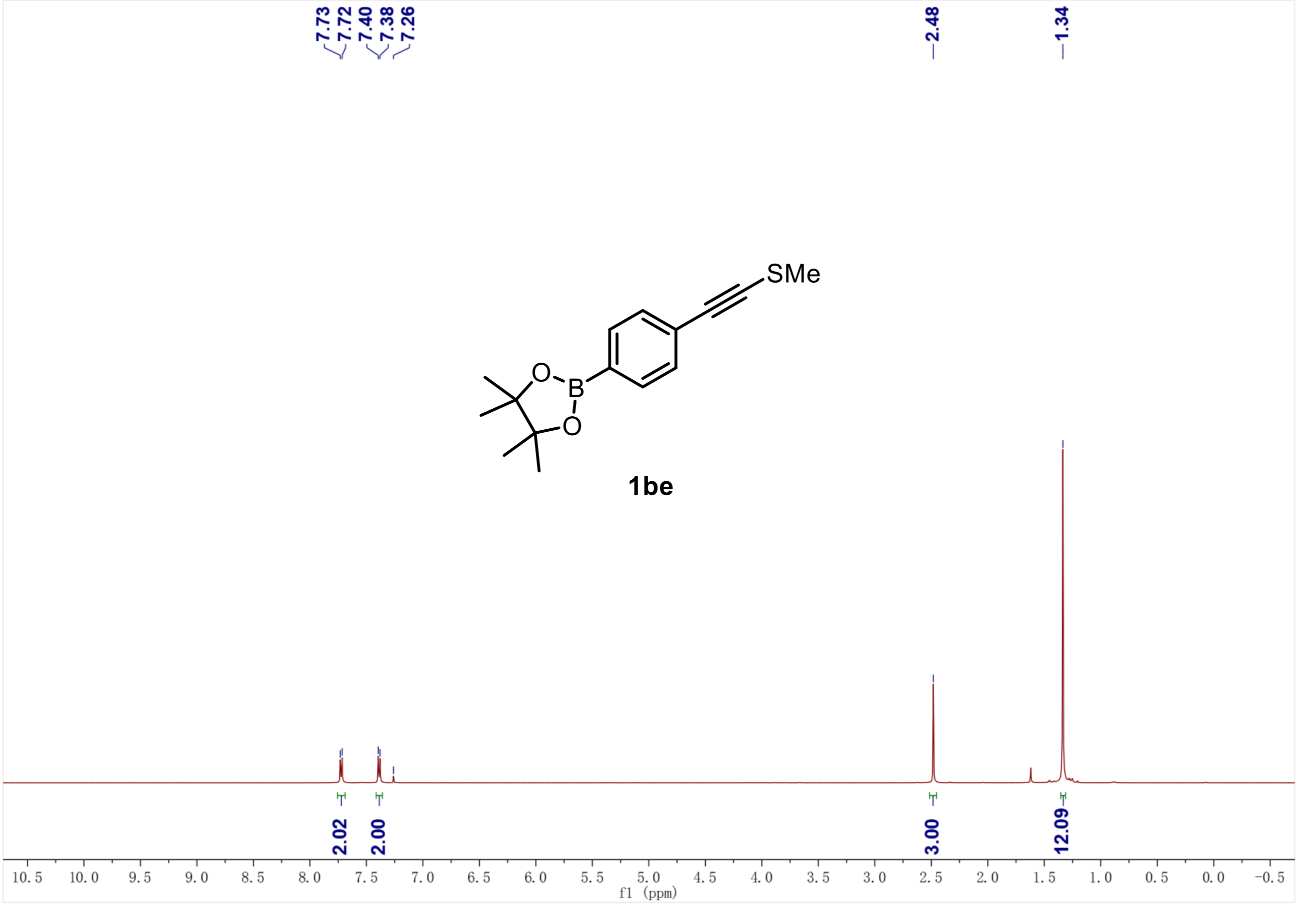

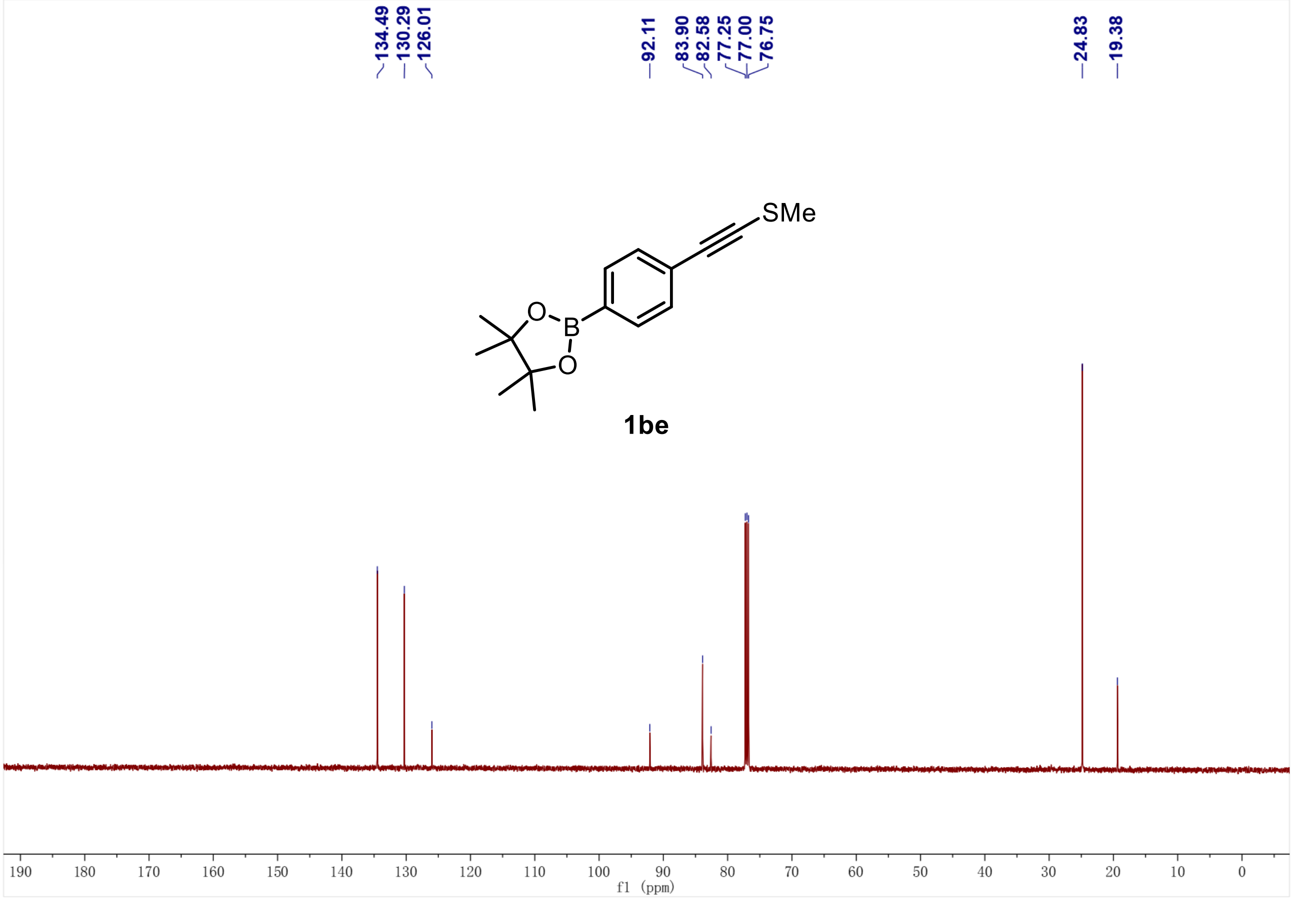

3a; ^{1}H NMR (500 MHz, $CDCl_3$); ^{13}C NMR (126 MHz, $CDCl_3$)

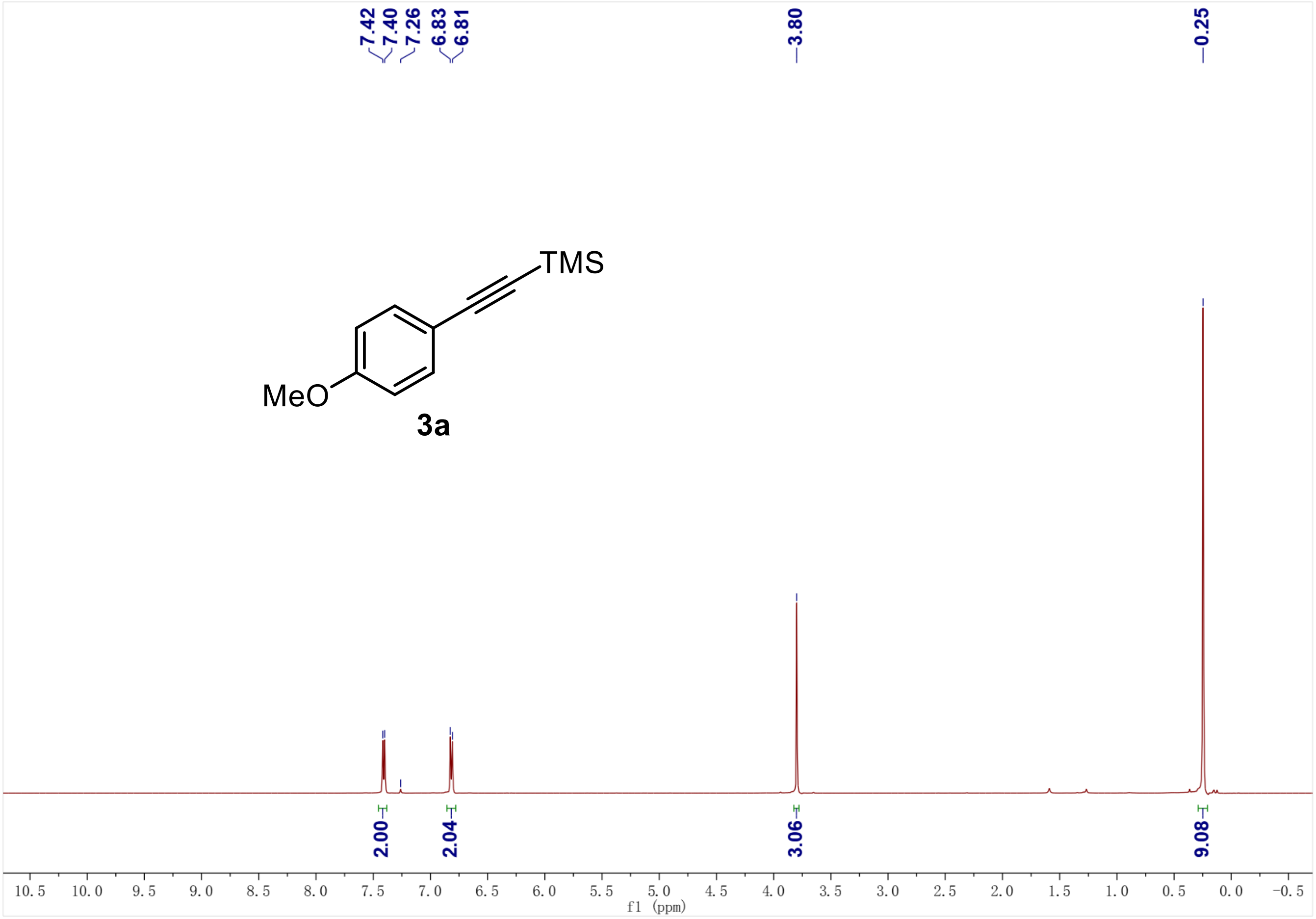

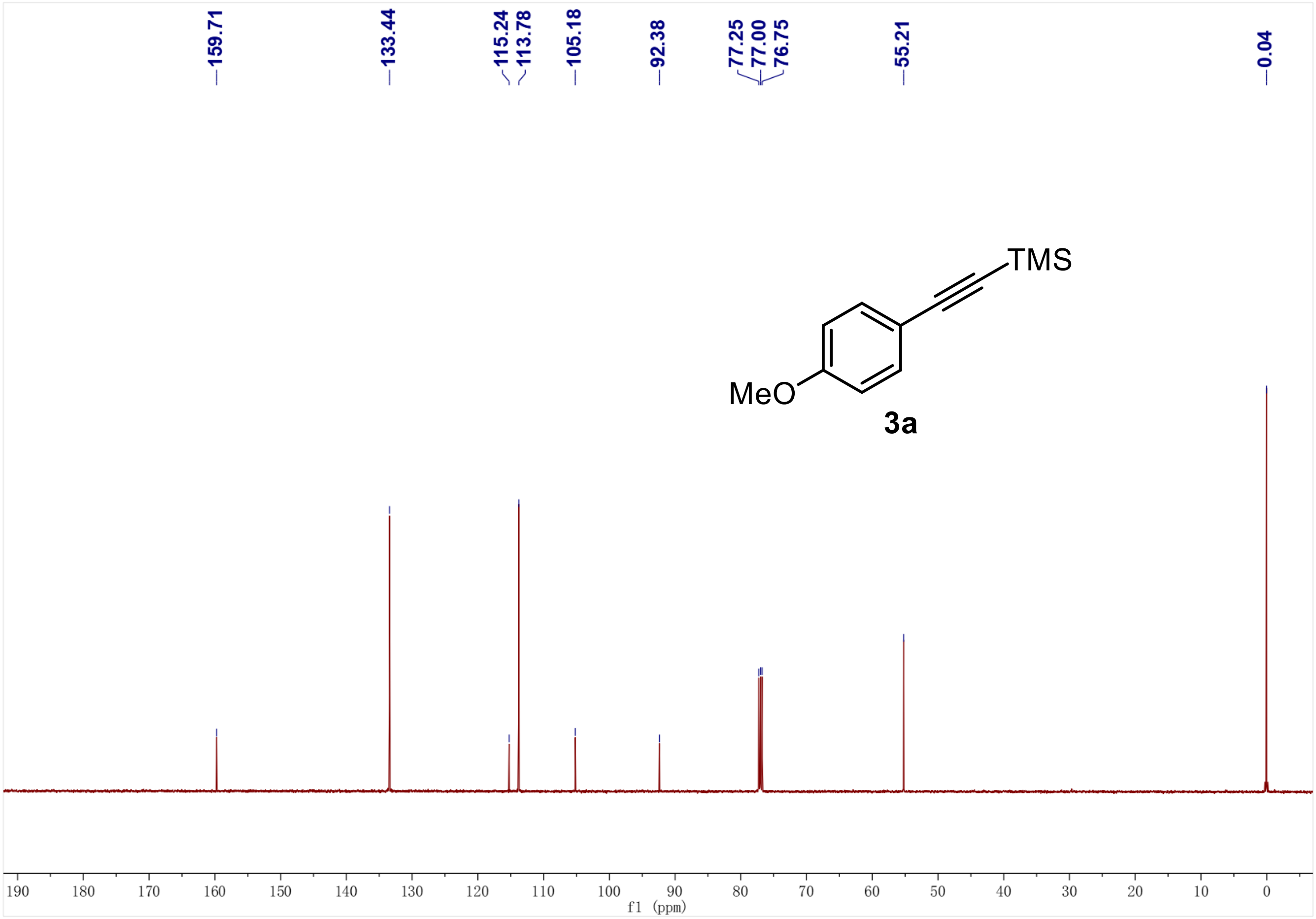

3b; ^{1}H NMR (400 MHz, $CDCl_3$); ^{13}C NMR (126 MHz, $CDCl_3$)

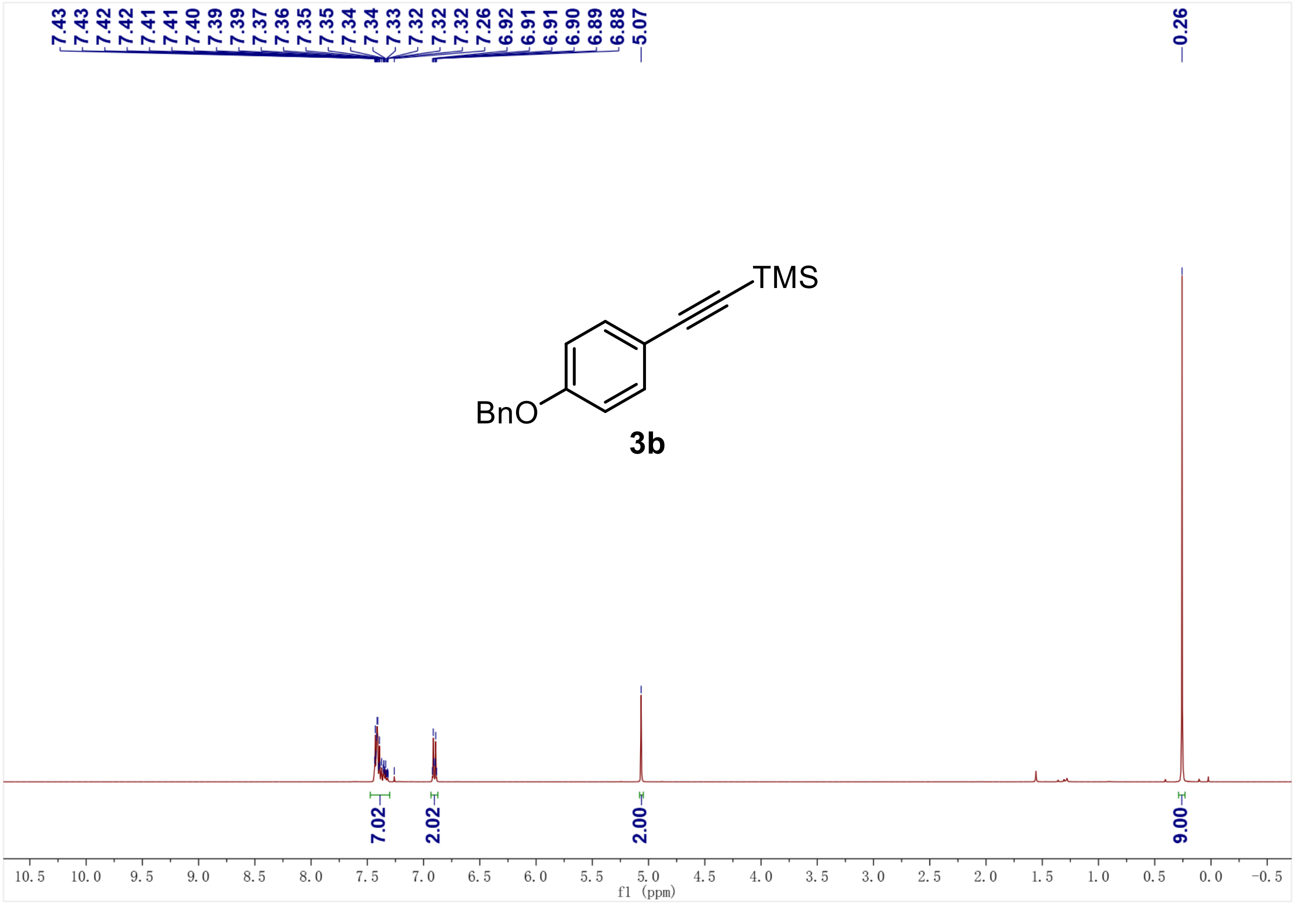

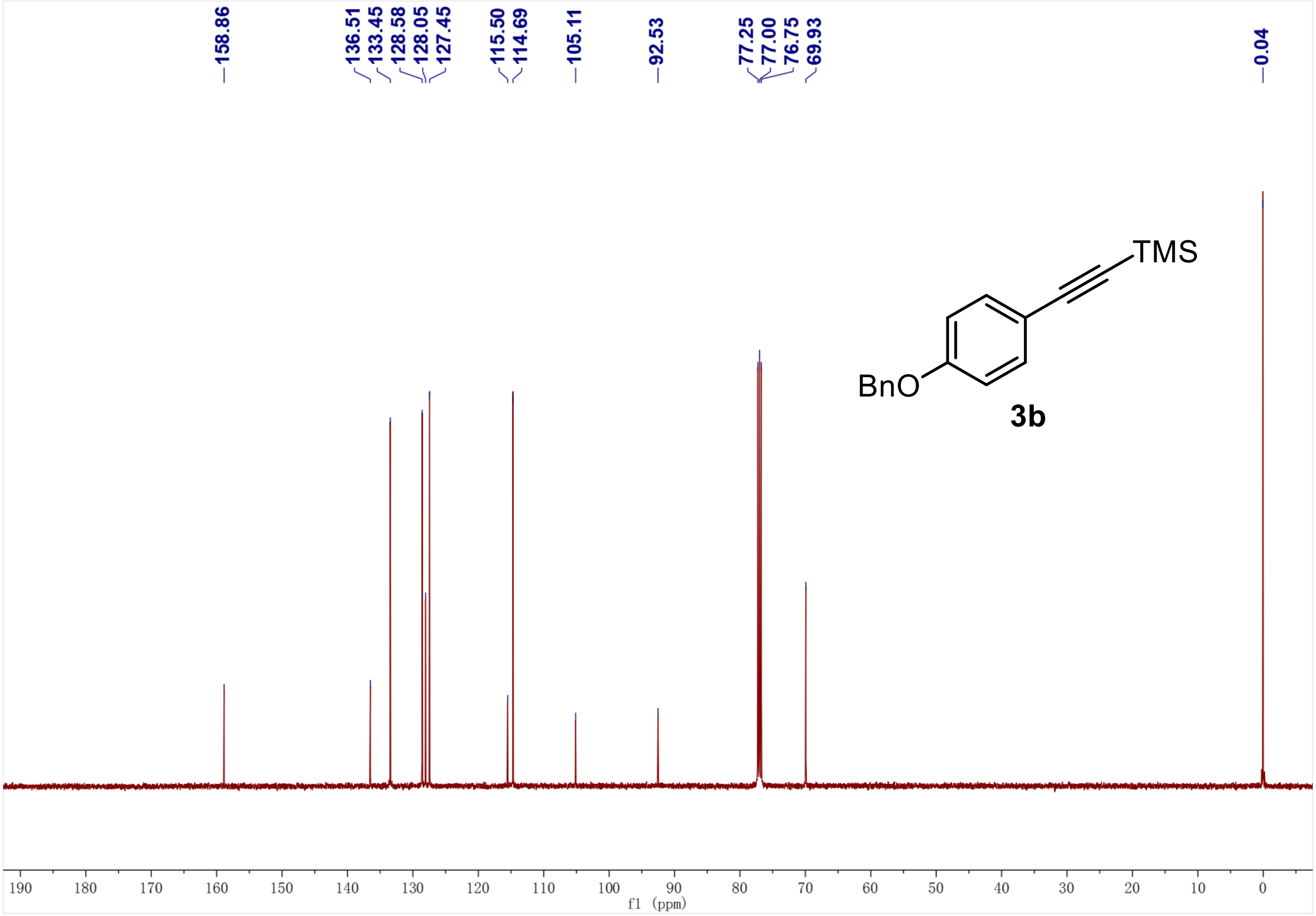

3c; ^{1}H NMR (400 MHz, $CDCl_3$); ^{13}C NMR (101 MHz, $CDCl_3$)

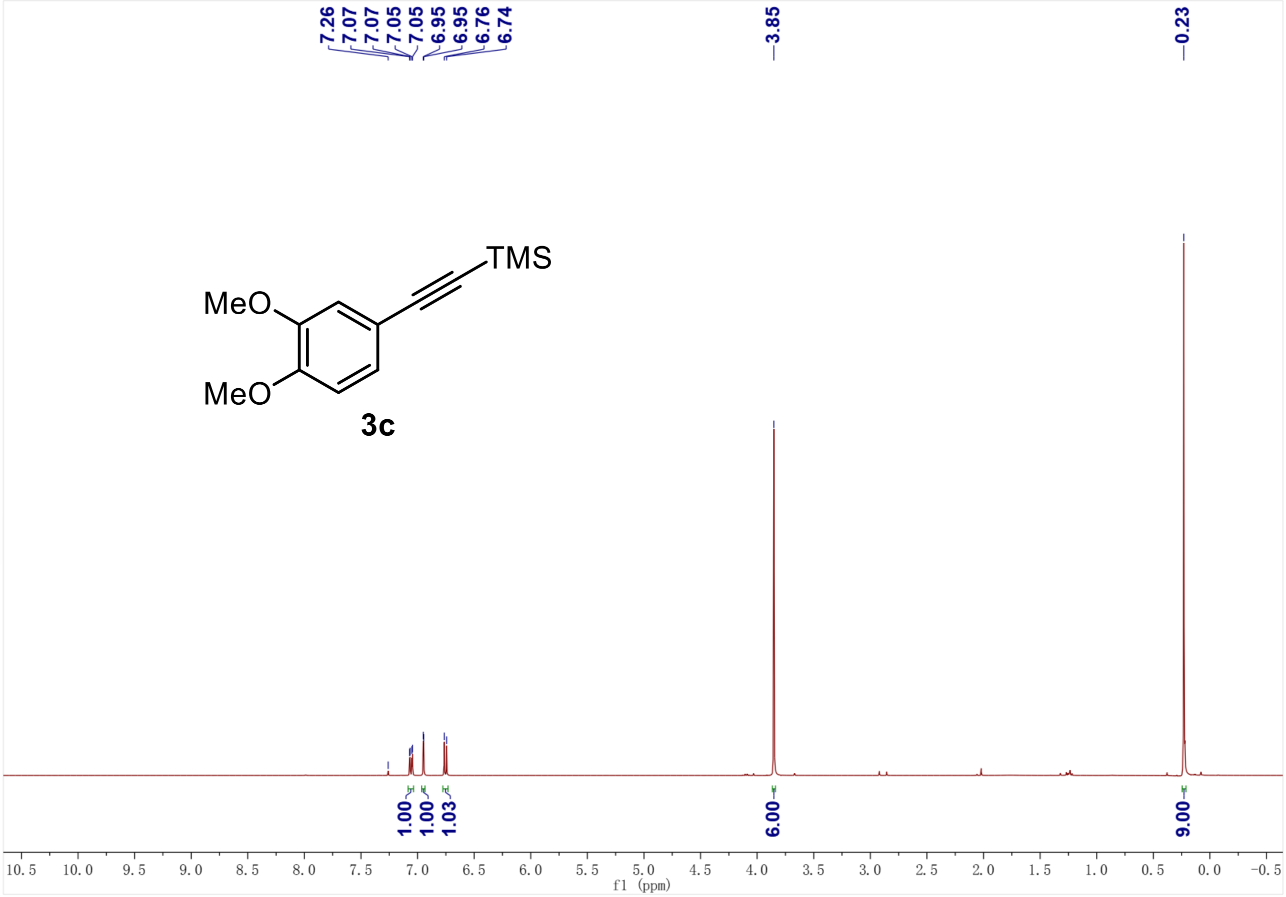

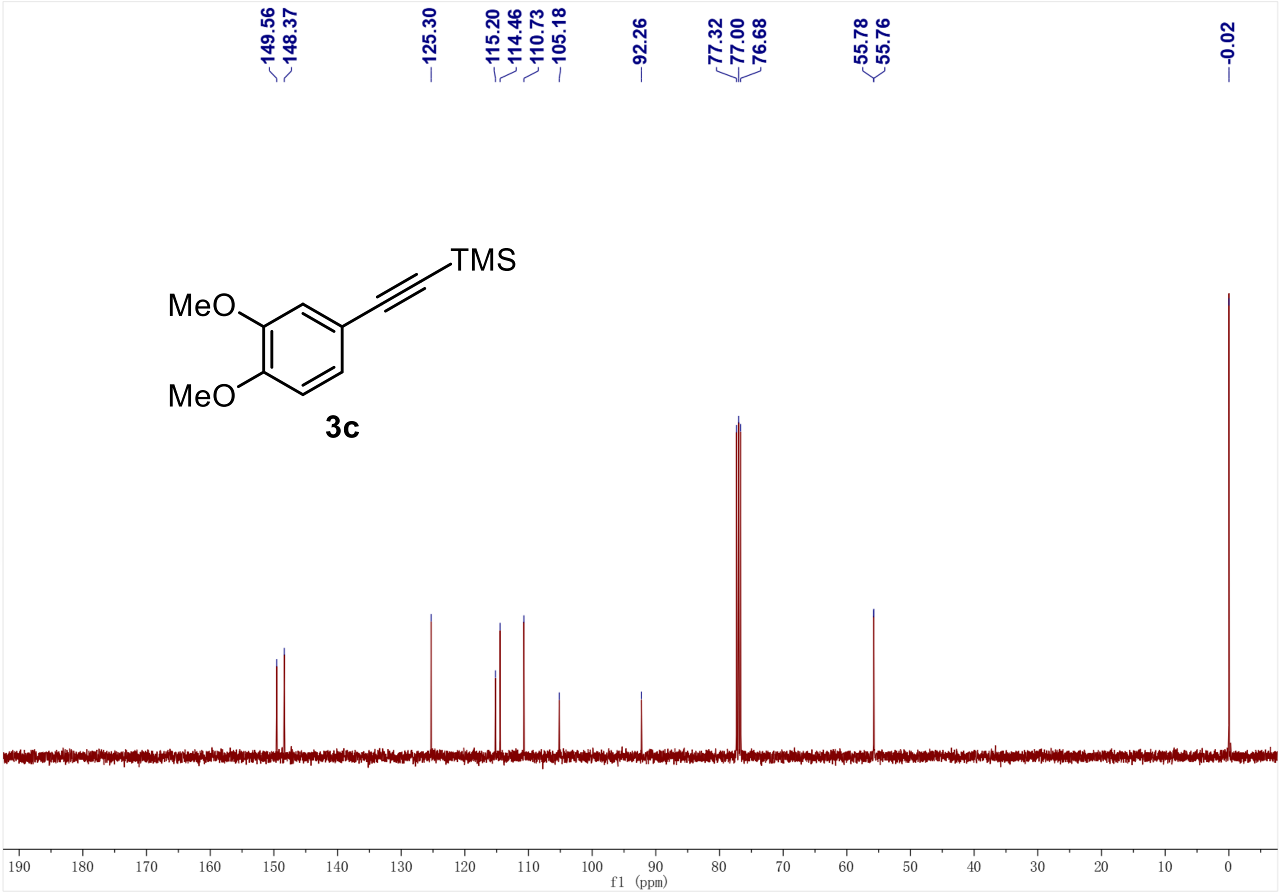

3d; ^{1}H NMR (500 MHz, $CDCl_3$); ^{13}C NMR (126 MHz, $CDCl_3$)

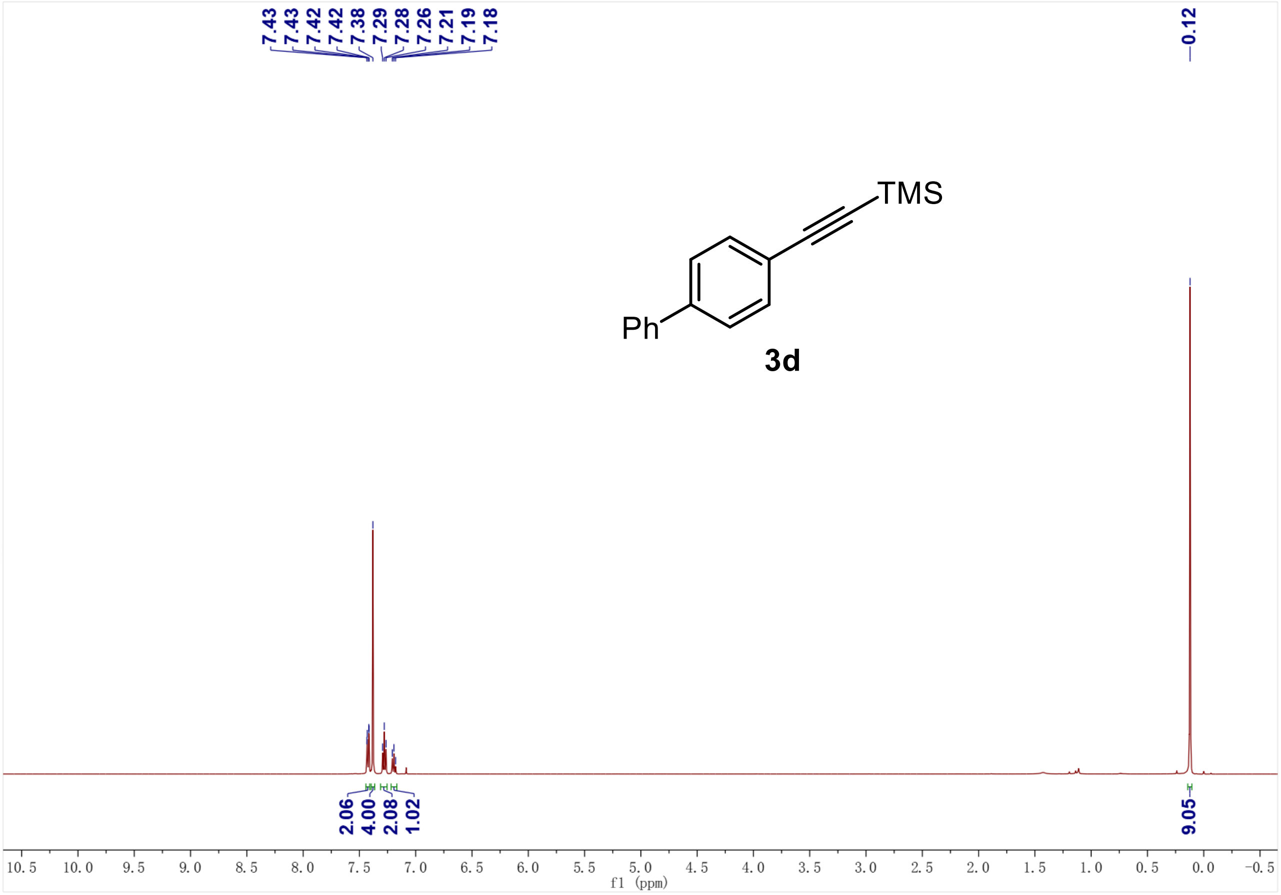

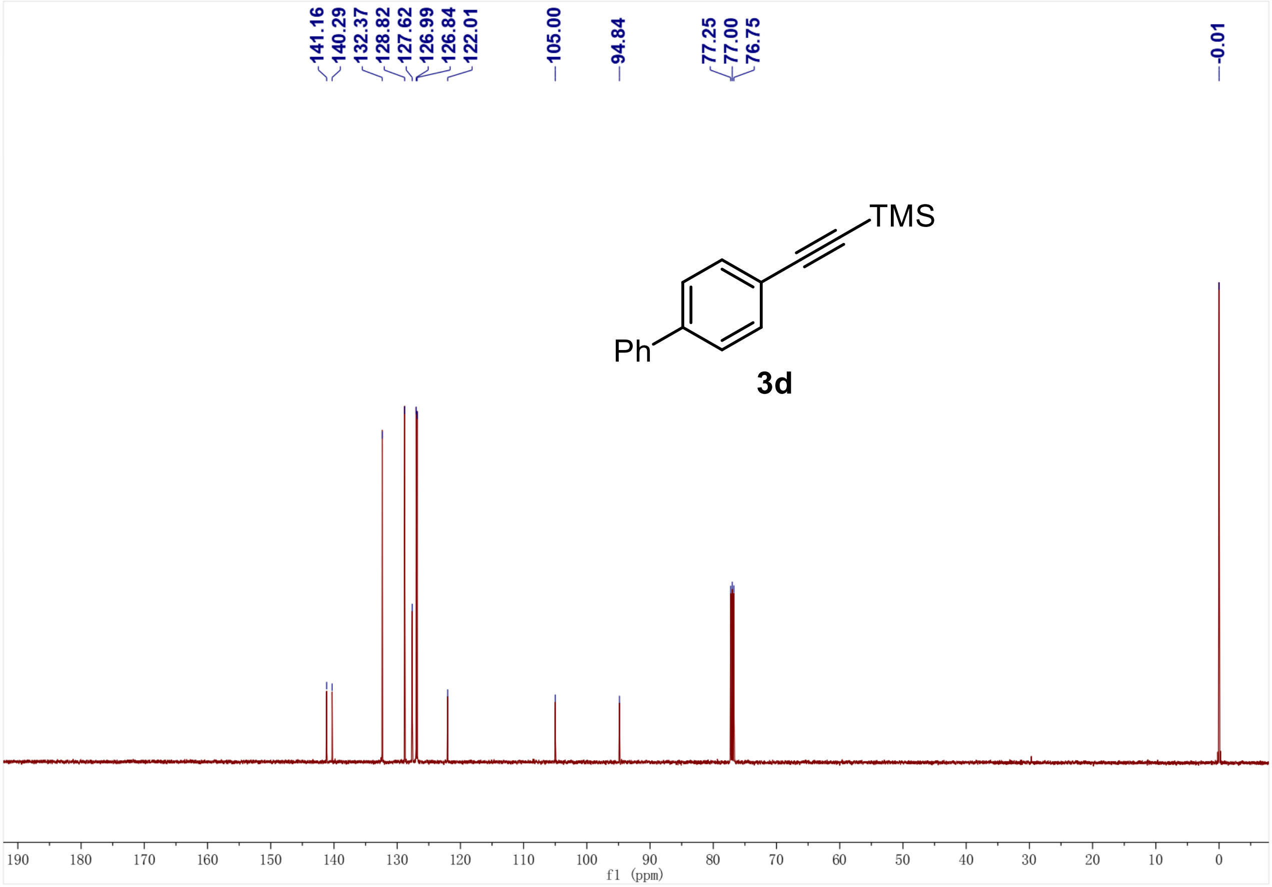

3e; ^{1}H NMR (500 MHz, $CDCl_3$); ^{13}C NMR (126 MHz, $CDCl_3$)

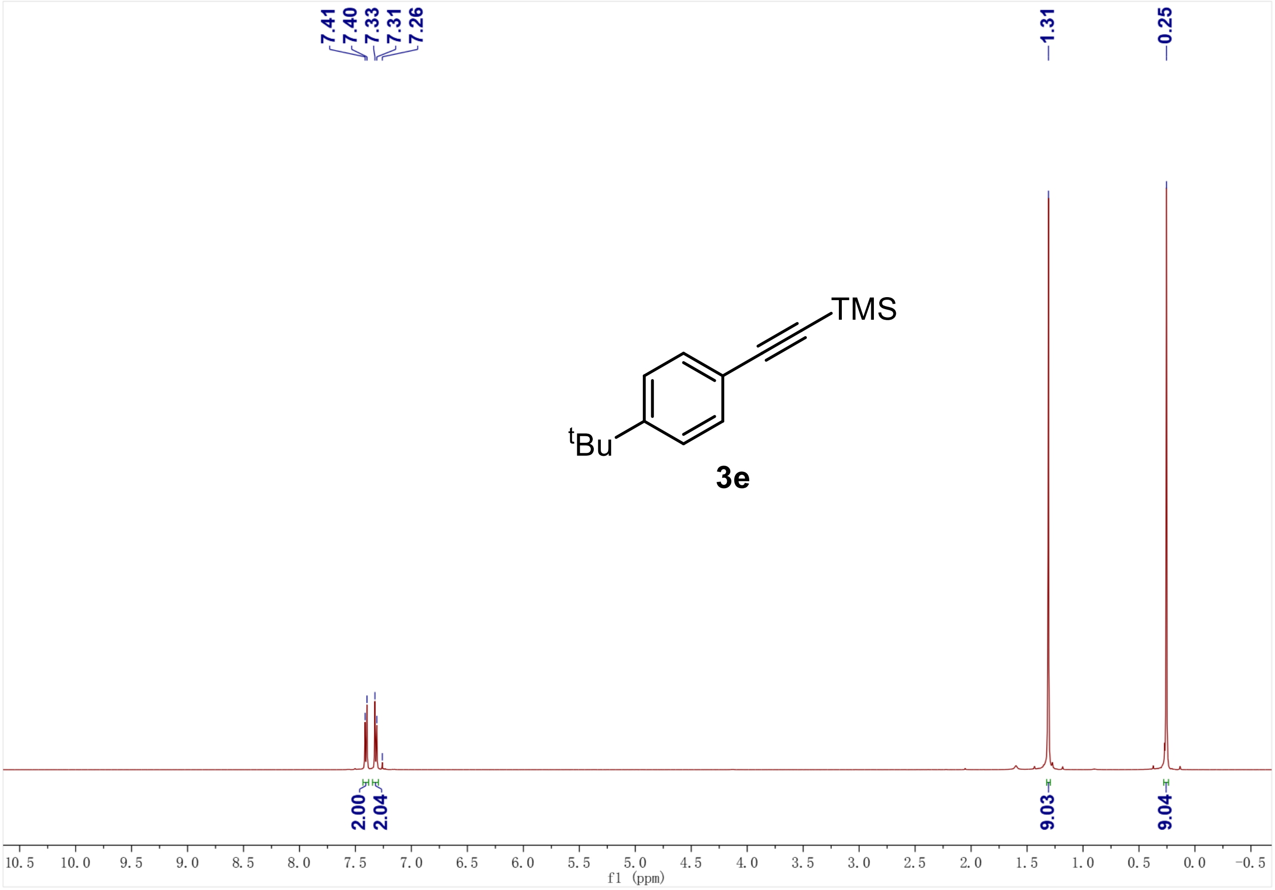

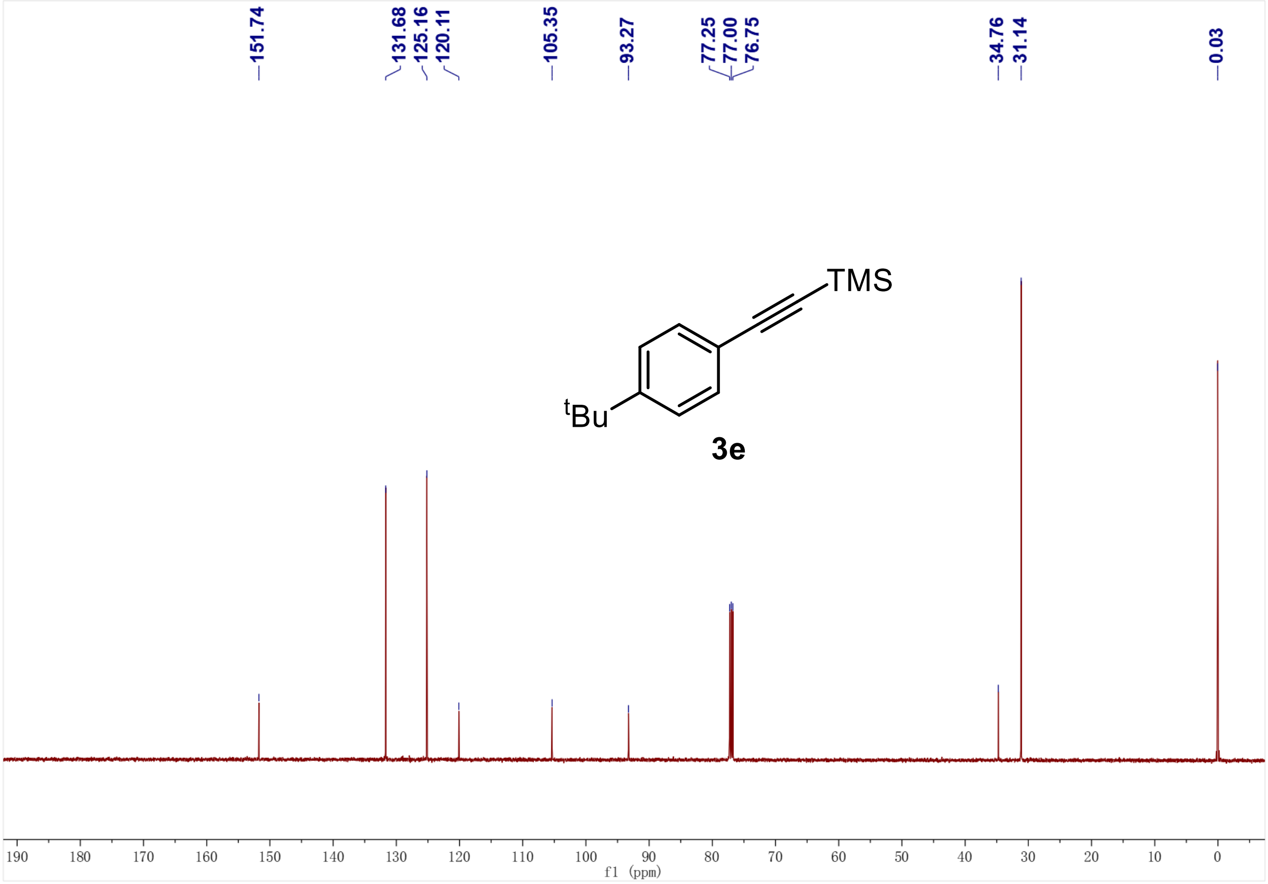

3f; ^{1}H NMR (500 MHz, $CDCl_3$); ^{13}C NMR (126 MHz, $CDCl_3$)

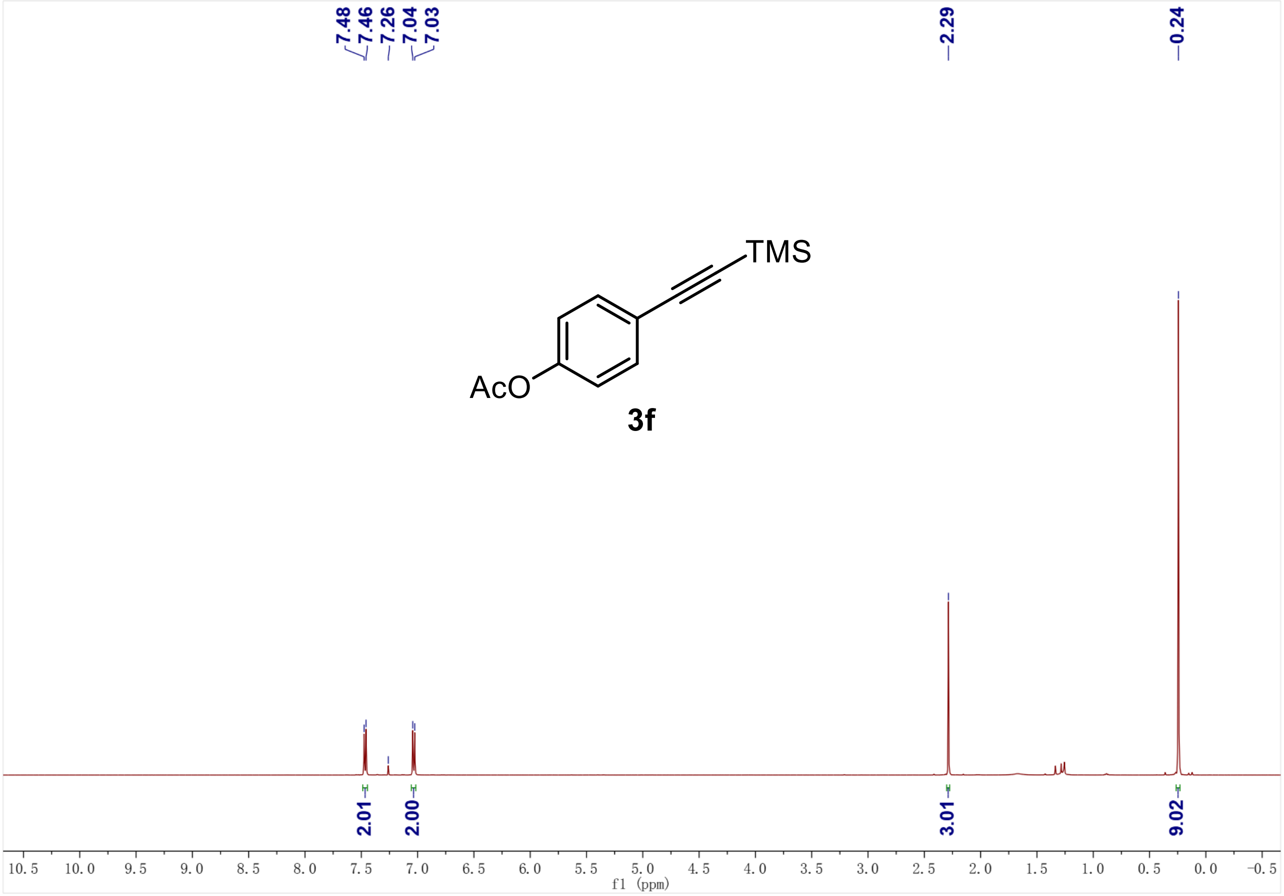

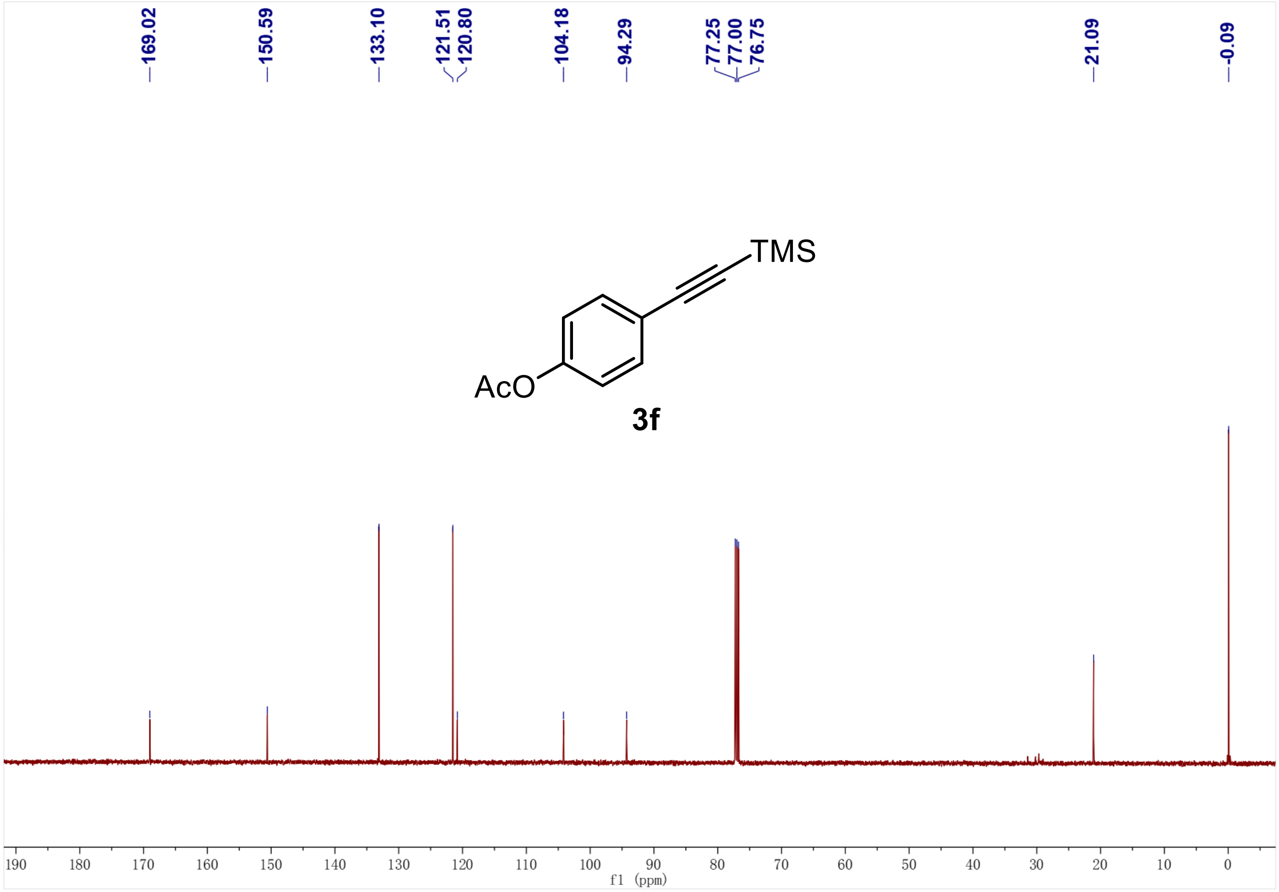

3g; ^1H NMR (500 MHz, $CDCl_3$); ^{13}C NMR (126 MHz, $CDCl_3$)

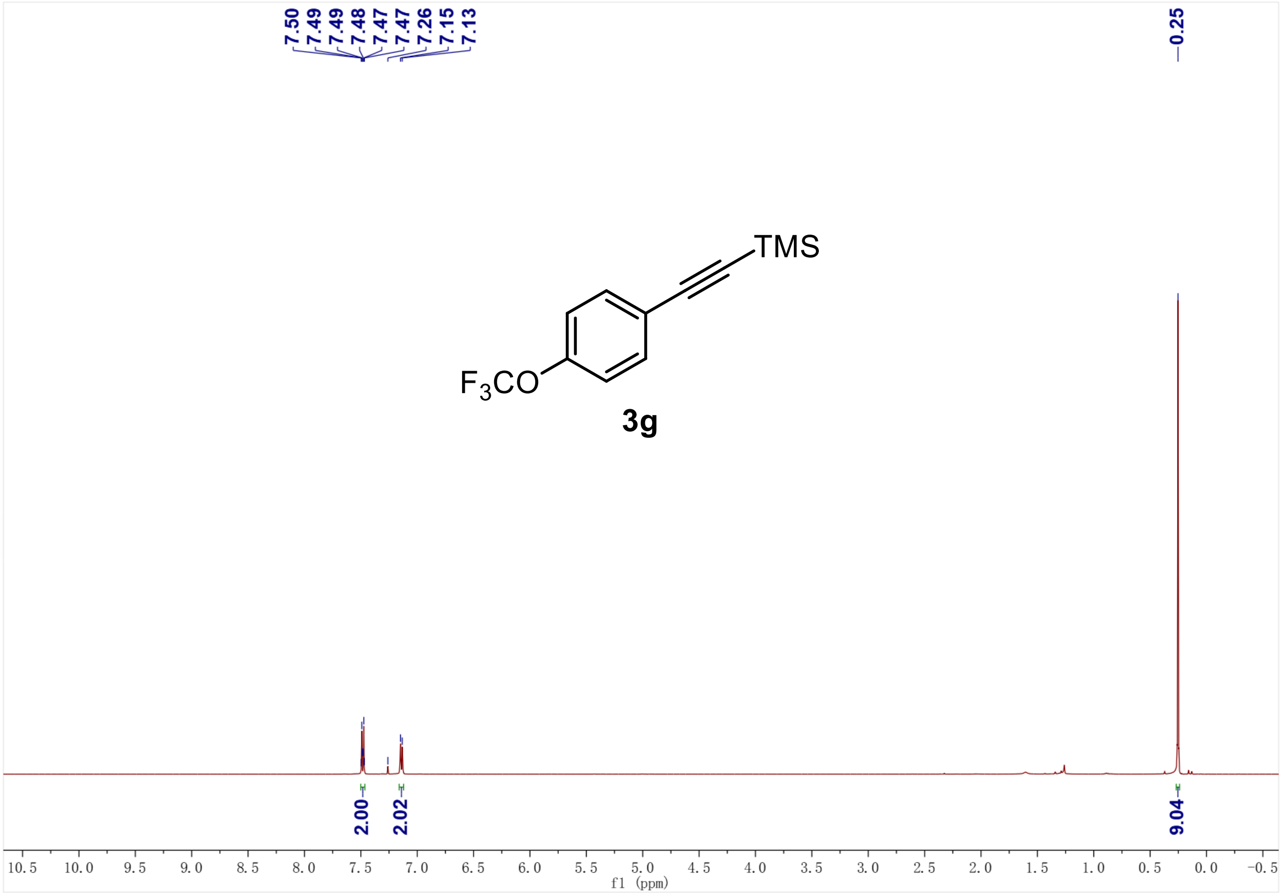

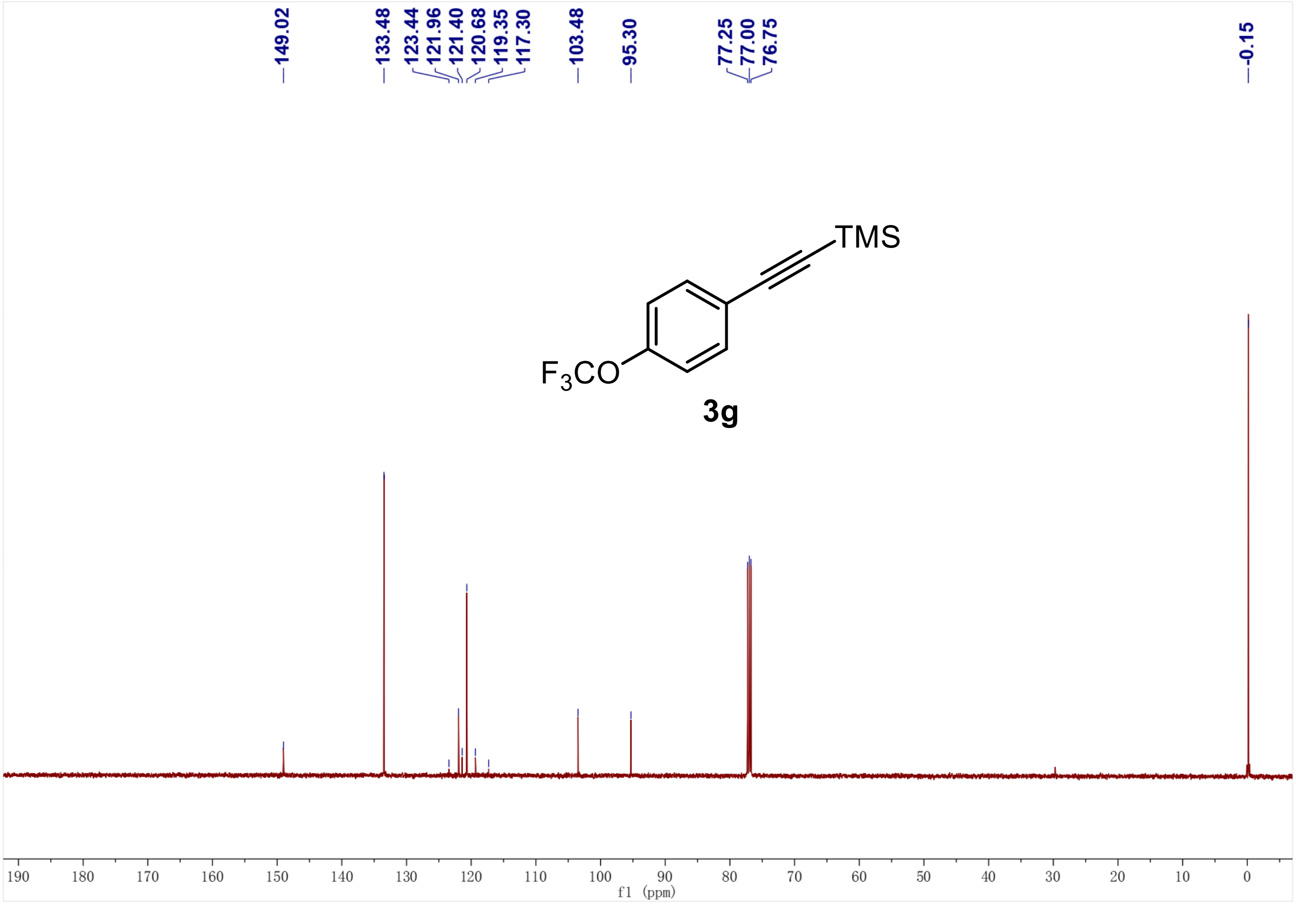

3g; ^{19}F NMR (471 MHz, $CDCl_3$)

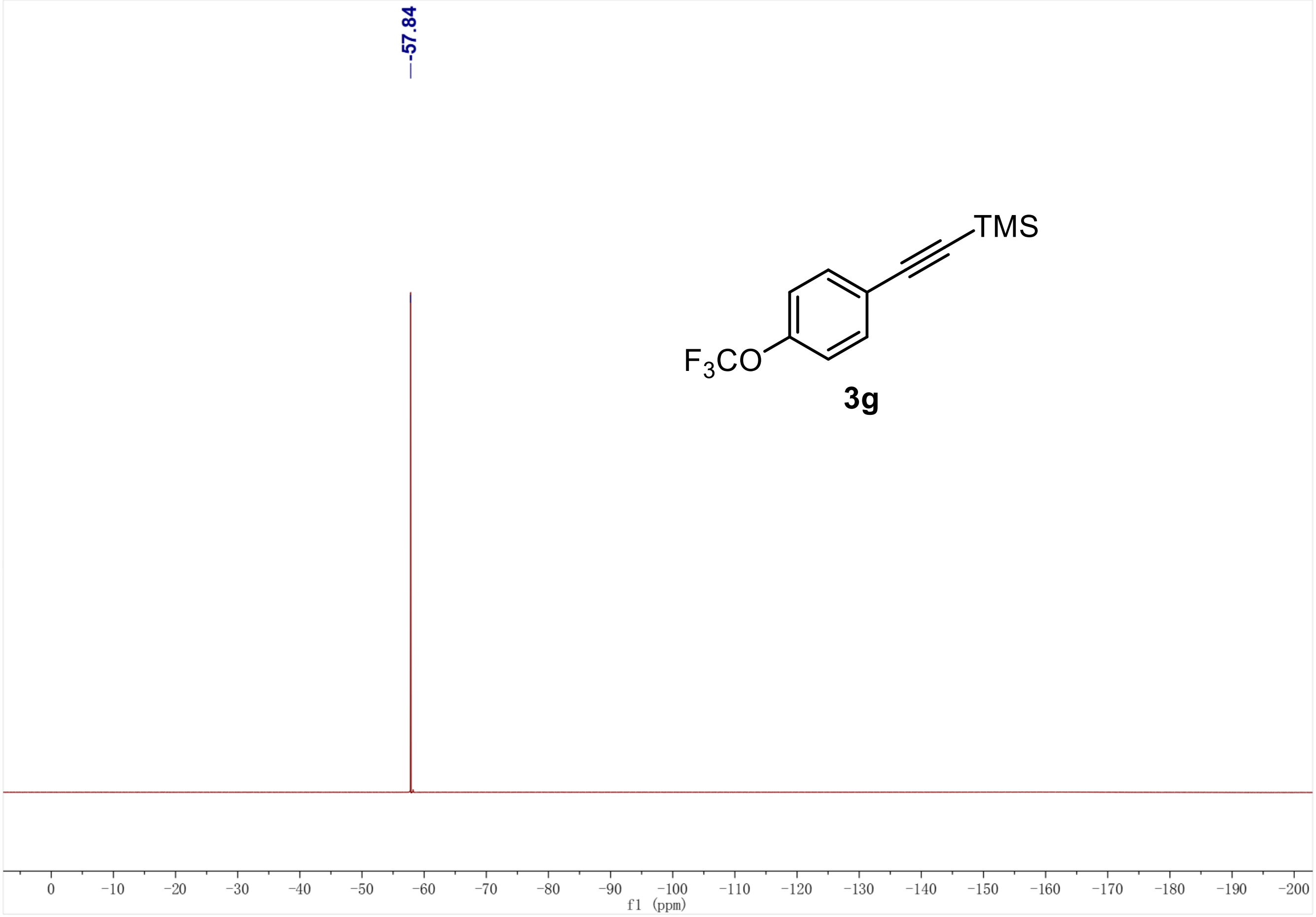

3h; ^{1}H NMR (500 MHz, $CDCl_3$); ^{13}C NMR (126 MHz, $CDCl_3$)

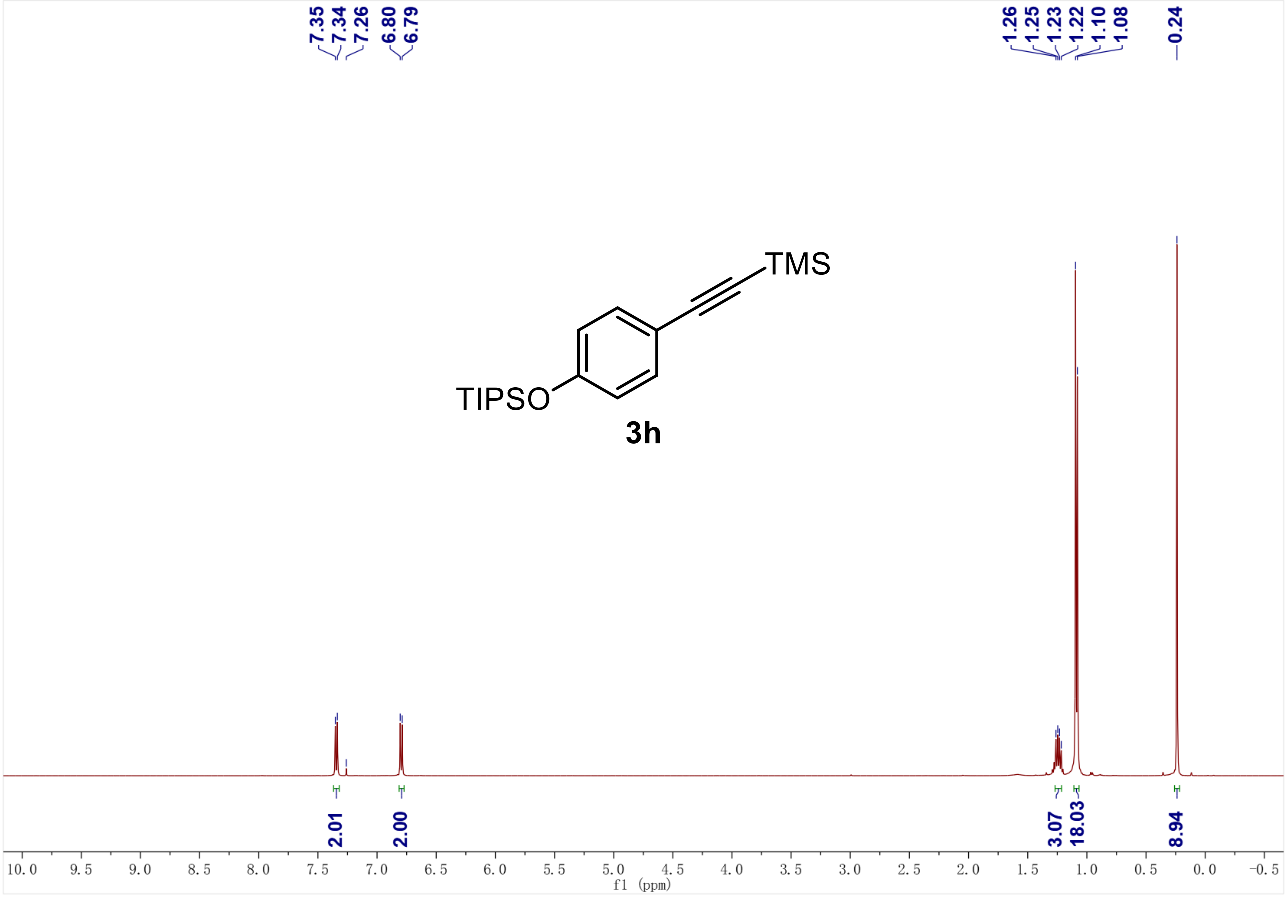

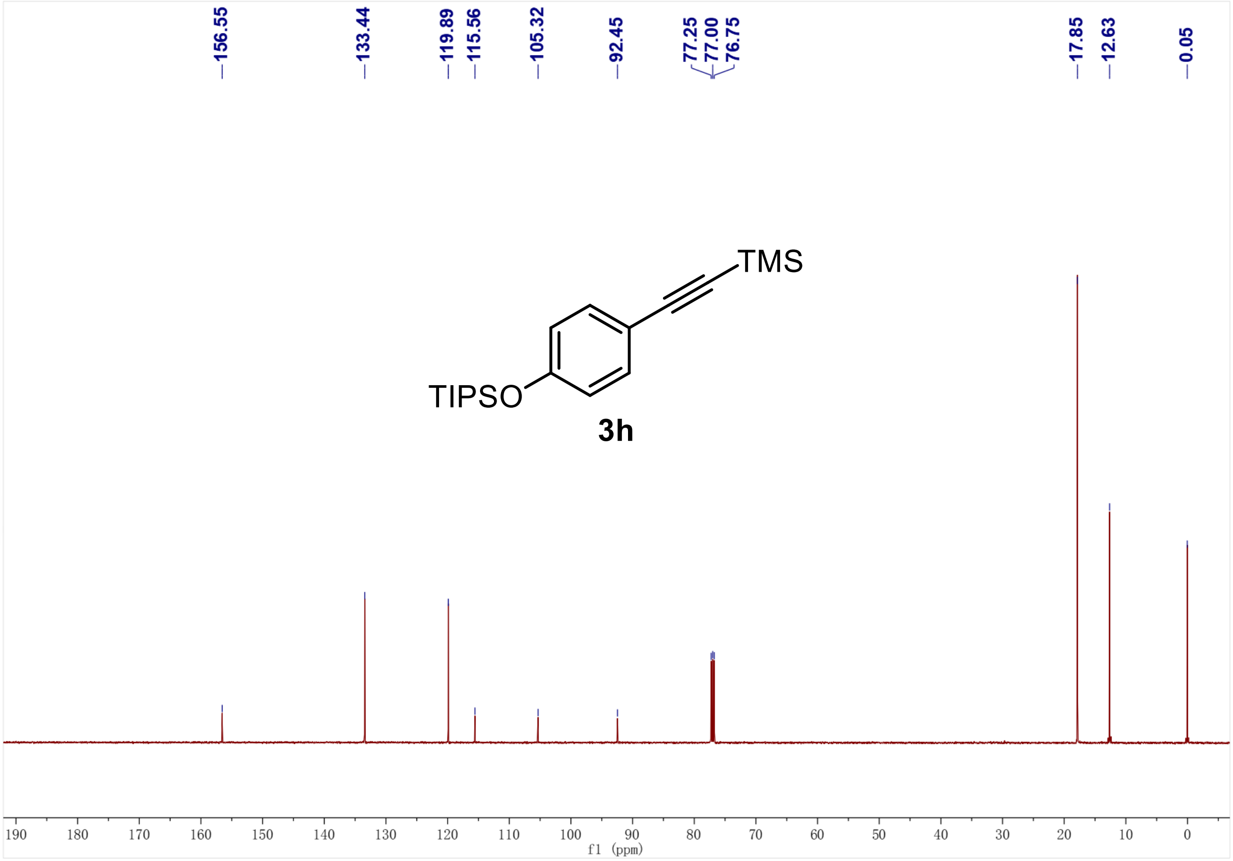

3i; ^{1}H NMR (500 MHz, $CDCl_3$); ^{13}C NMR (126 MHz, $CDCl_3$)

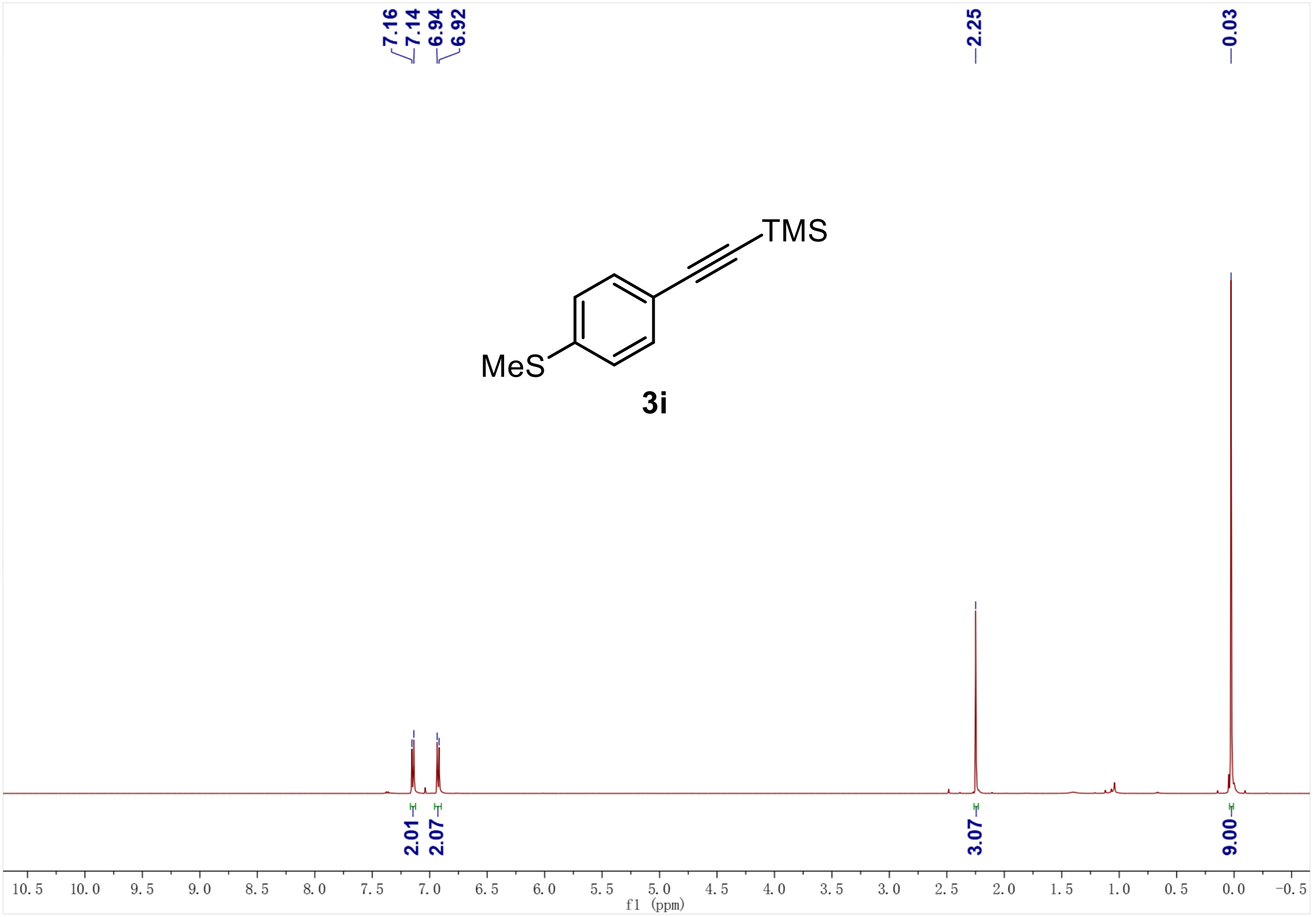

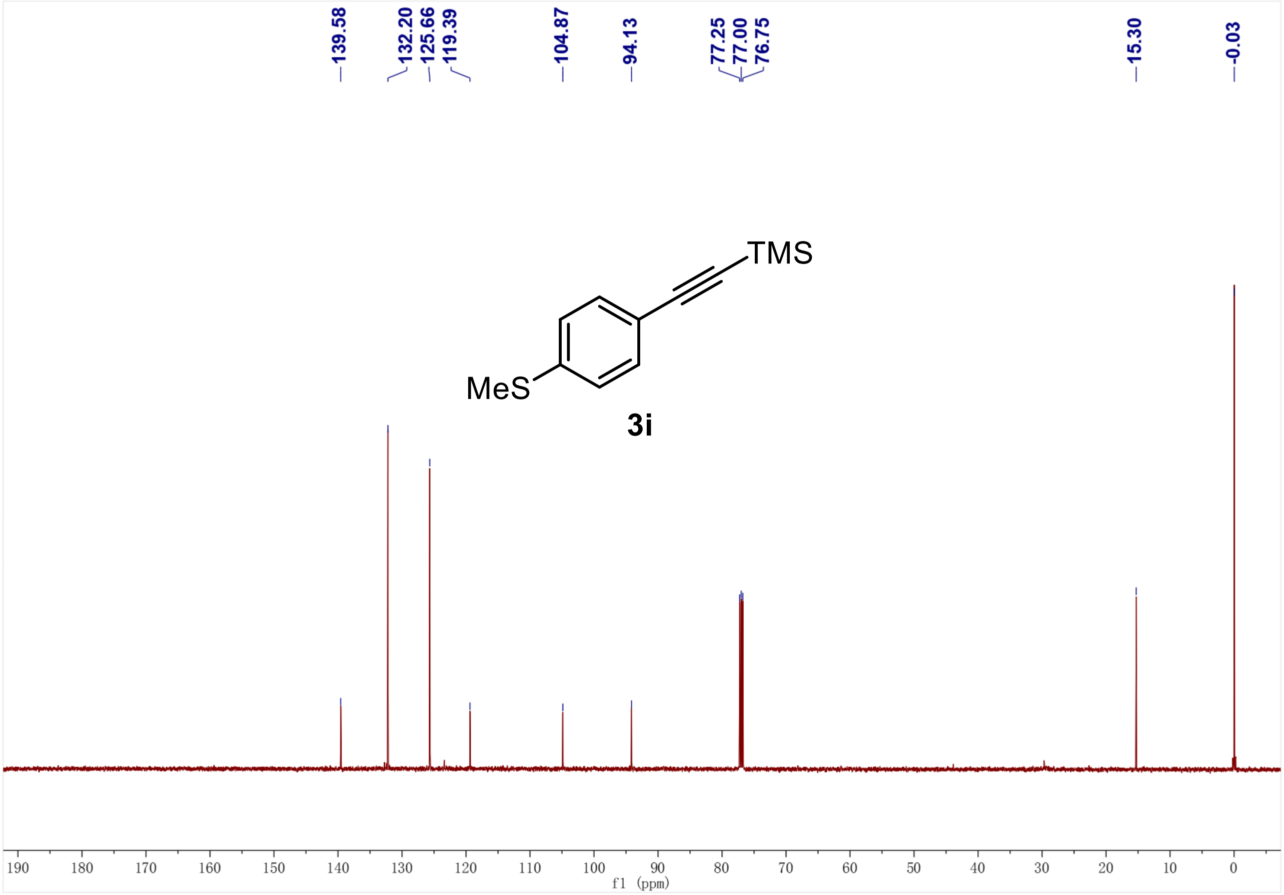

3j; ^{1}H NMR (500 MHz, $CDCl_3$); ^{13}C NMR (126 MHz, $CDCl_3$)

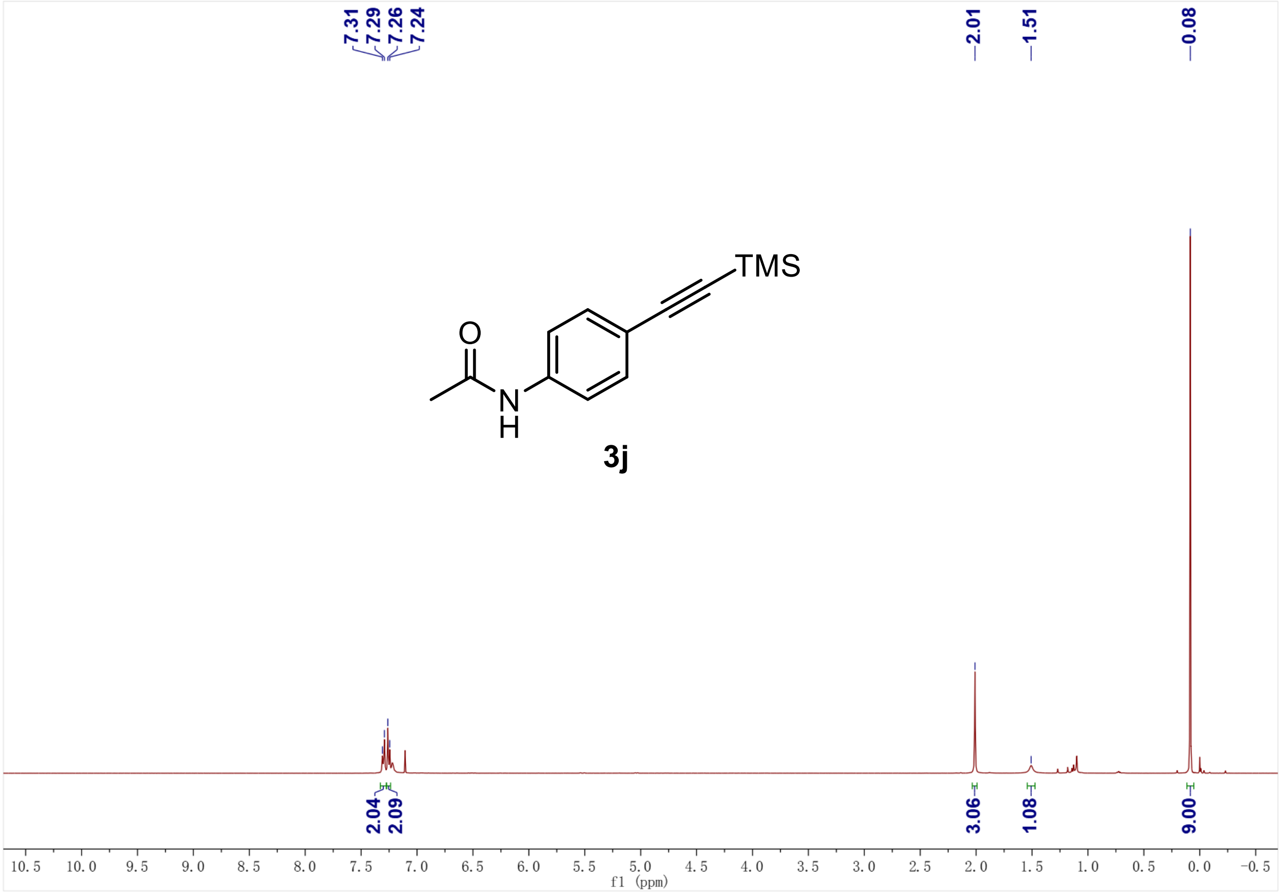

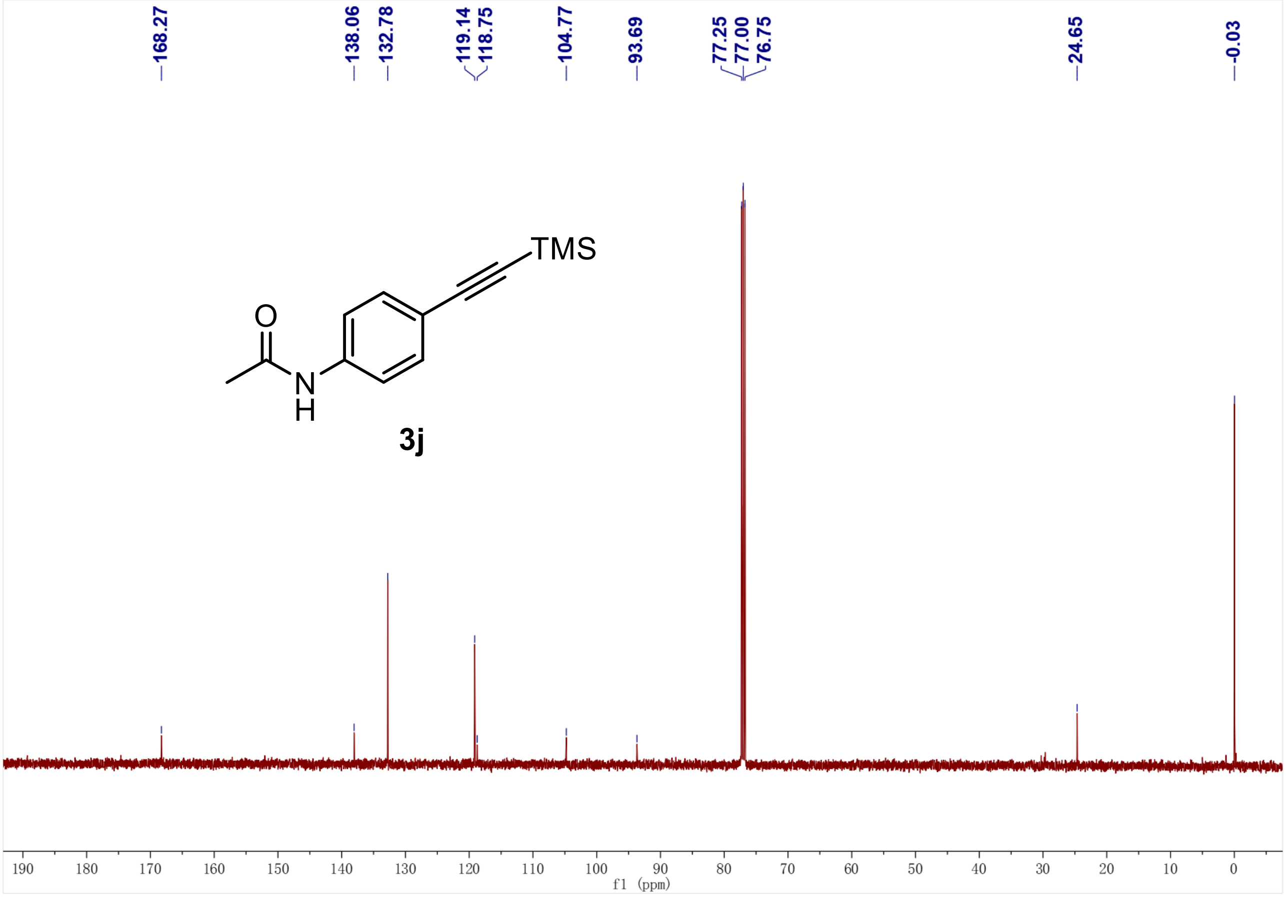

3k; ^{1}H NMR (400 MHz, $CDCl_3$); ^{13}C NMR (126 MHz, $CDCl_3$)

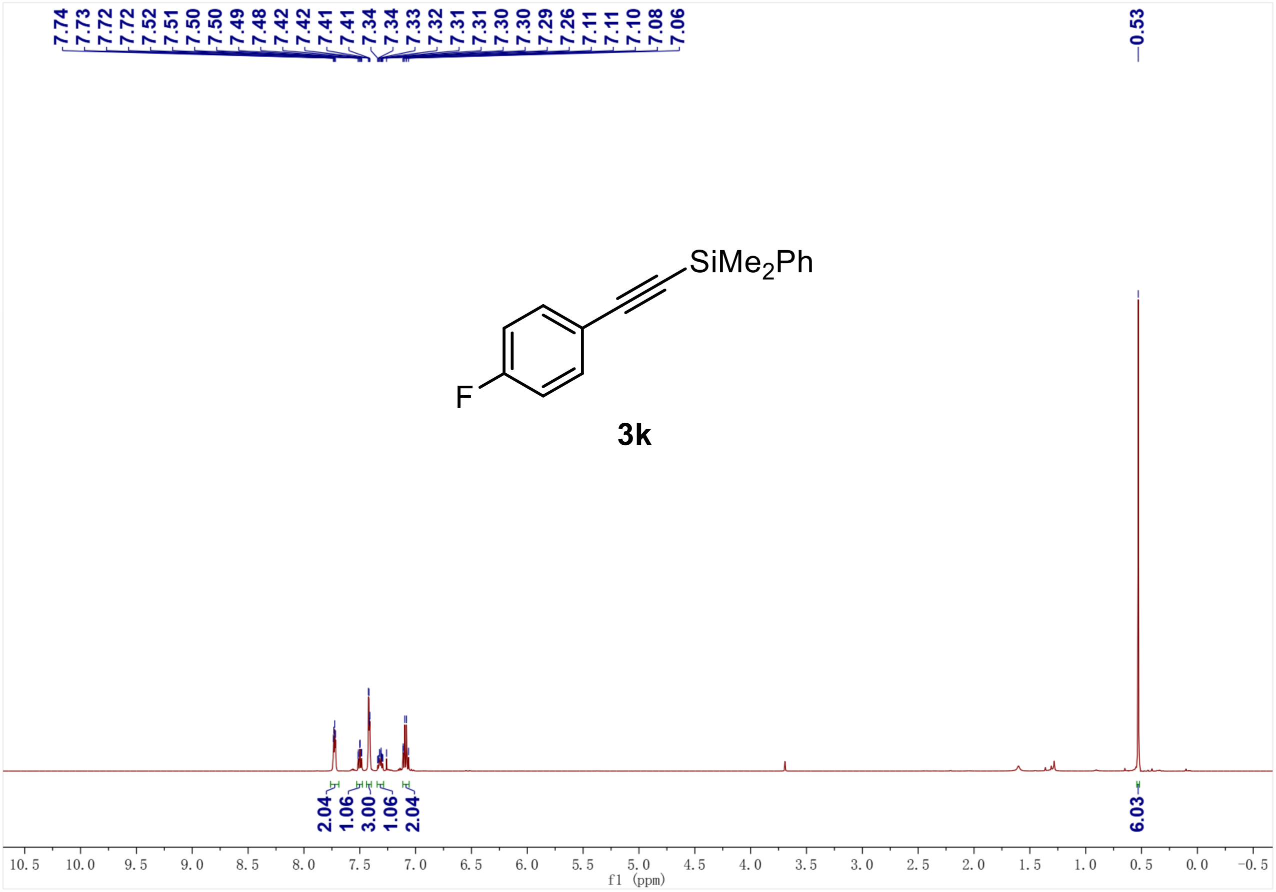

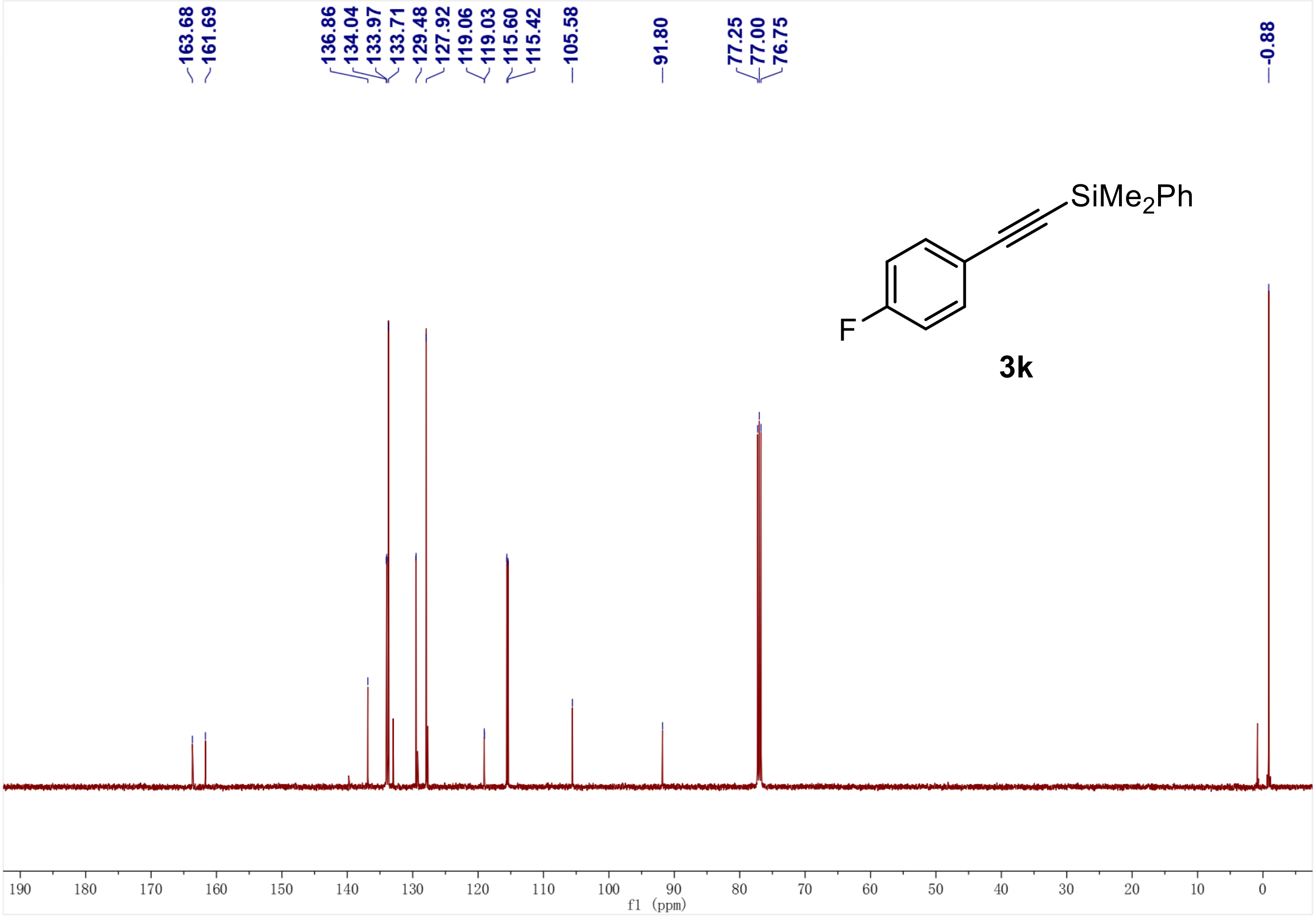

3k; ^{19}F NMR (471 MHz, CDCl$_3$)

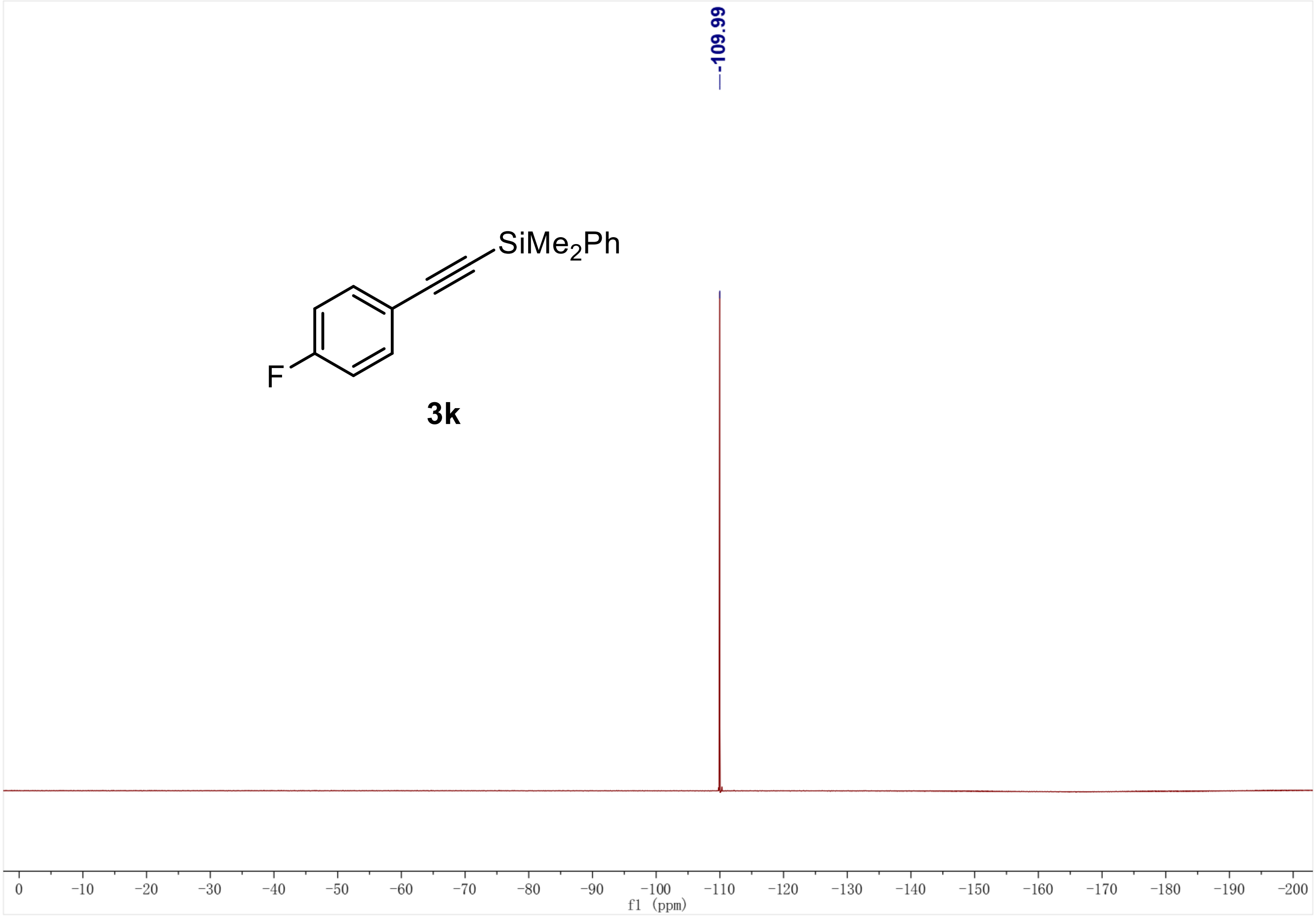

3l; ^{1}H NMR (500 MHz, $CDCl_3$); ^{13}C NMR (126 MHz, $CDCl_3$)

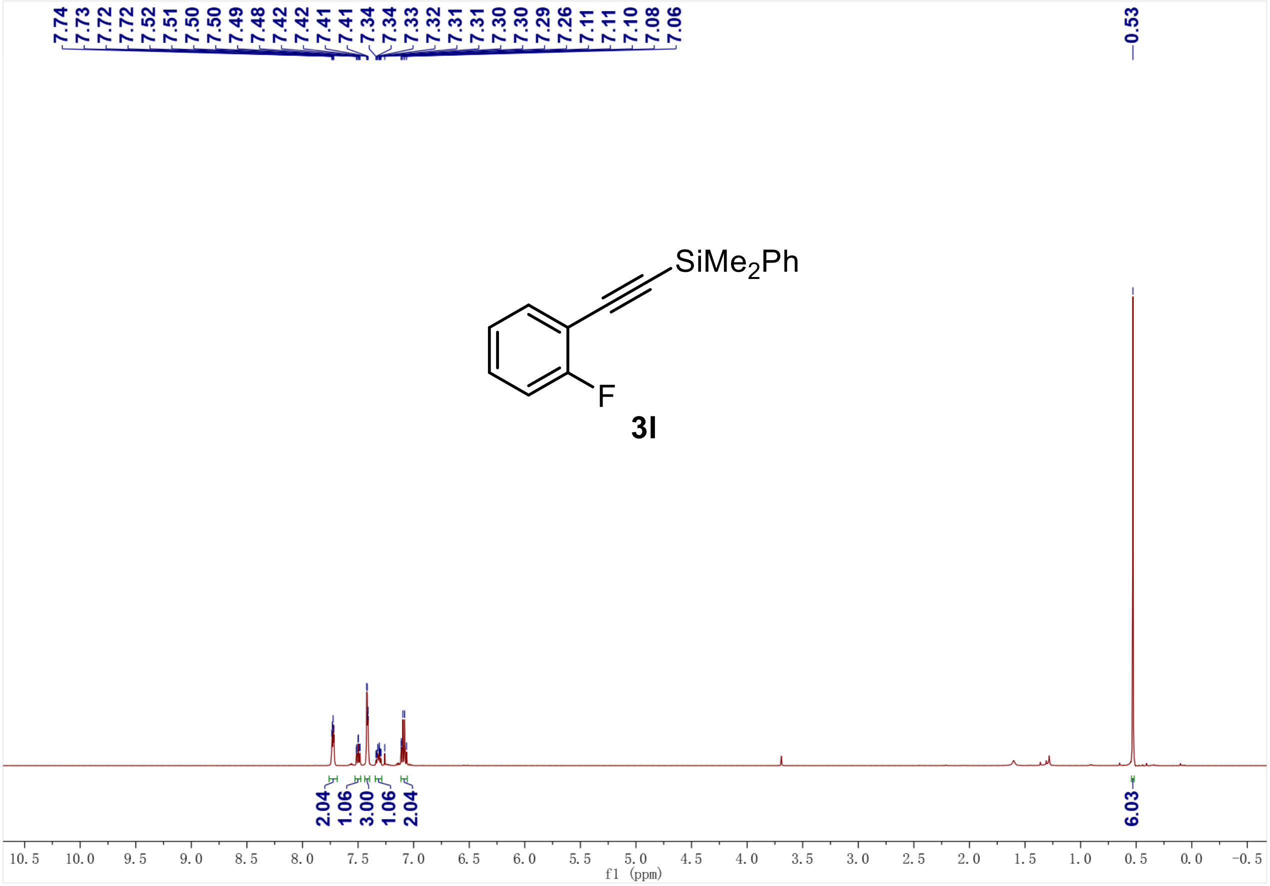

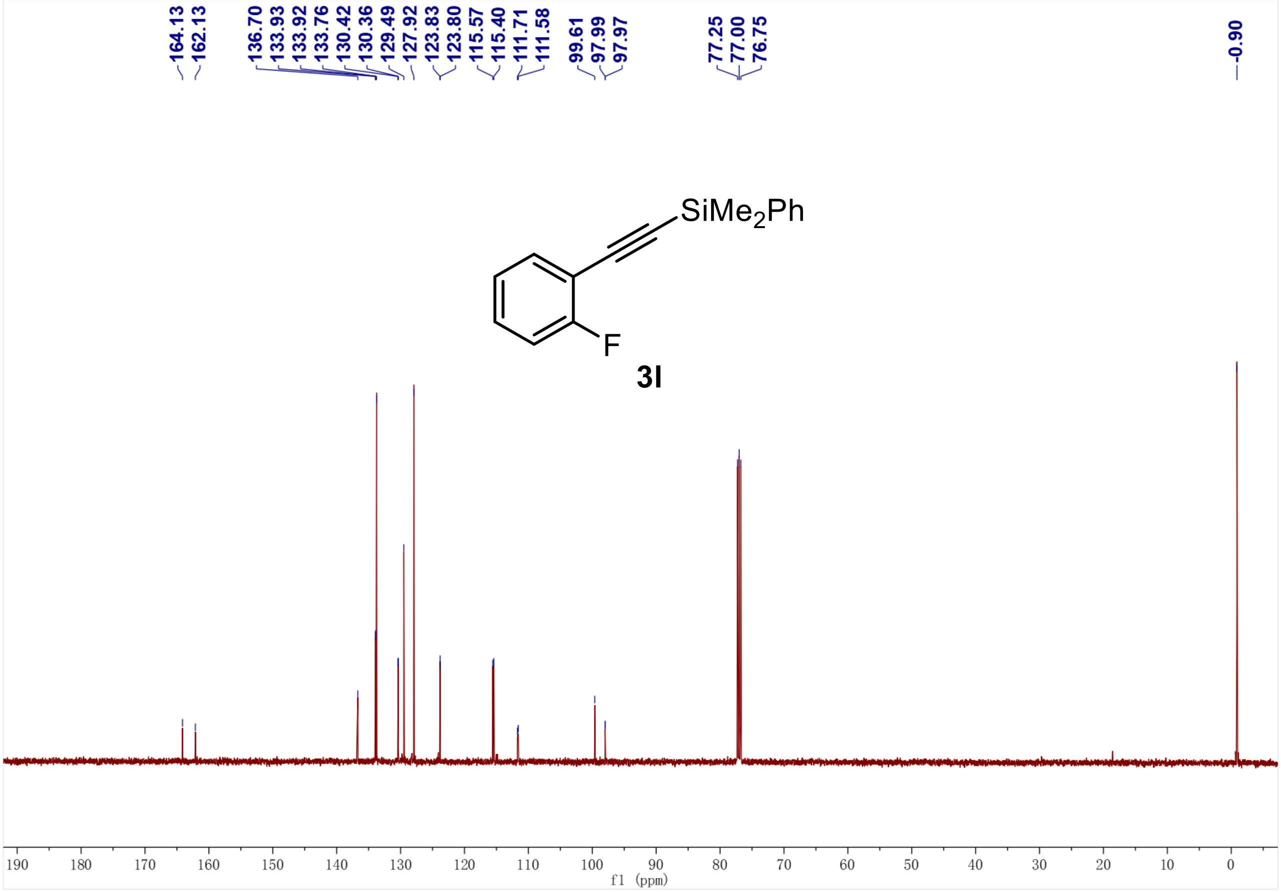

3l; ^{19}F NMR (471 MHz, $CDCl_3$)

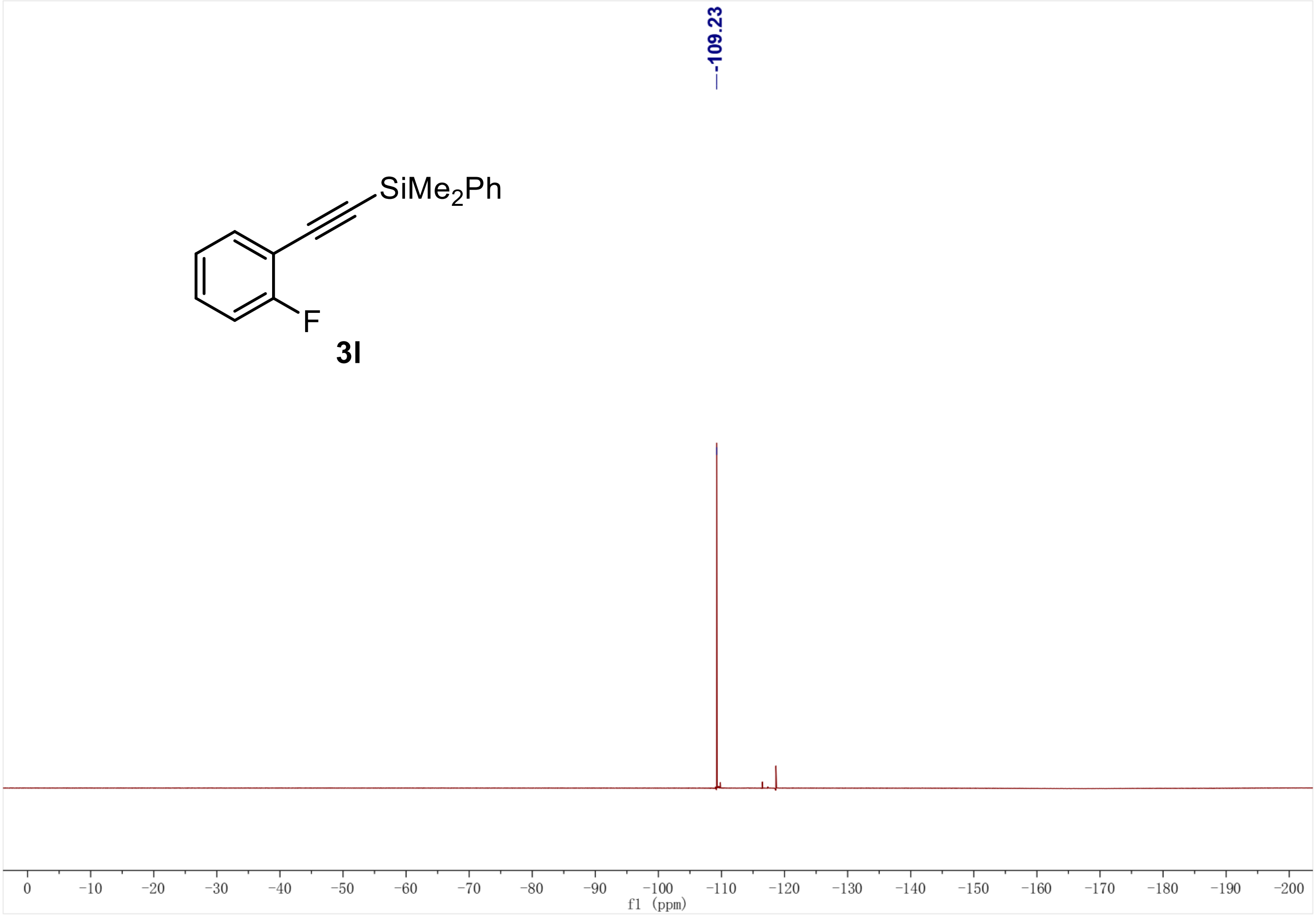
-109.23
SiMe2Ph
F
3l
0
−10
−20
−30
−40
−50
−60
−70
−80
−90
−100
−110
−120
−130
−140
−150
−160
−170
−180
−190
−200
f1 (ppm)

3m; ^{1}H NMR (500 MHz, CDCl$_3$); ^{13}C NMR (126 MHz, CDCl$_3$)

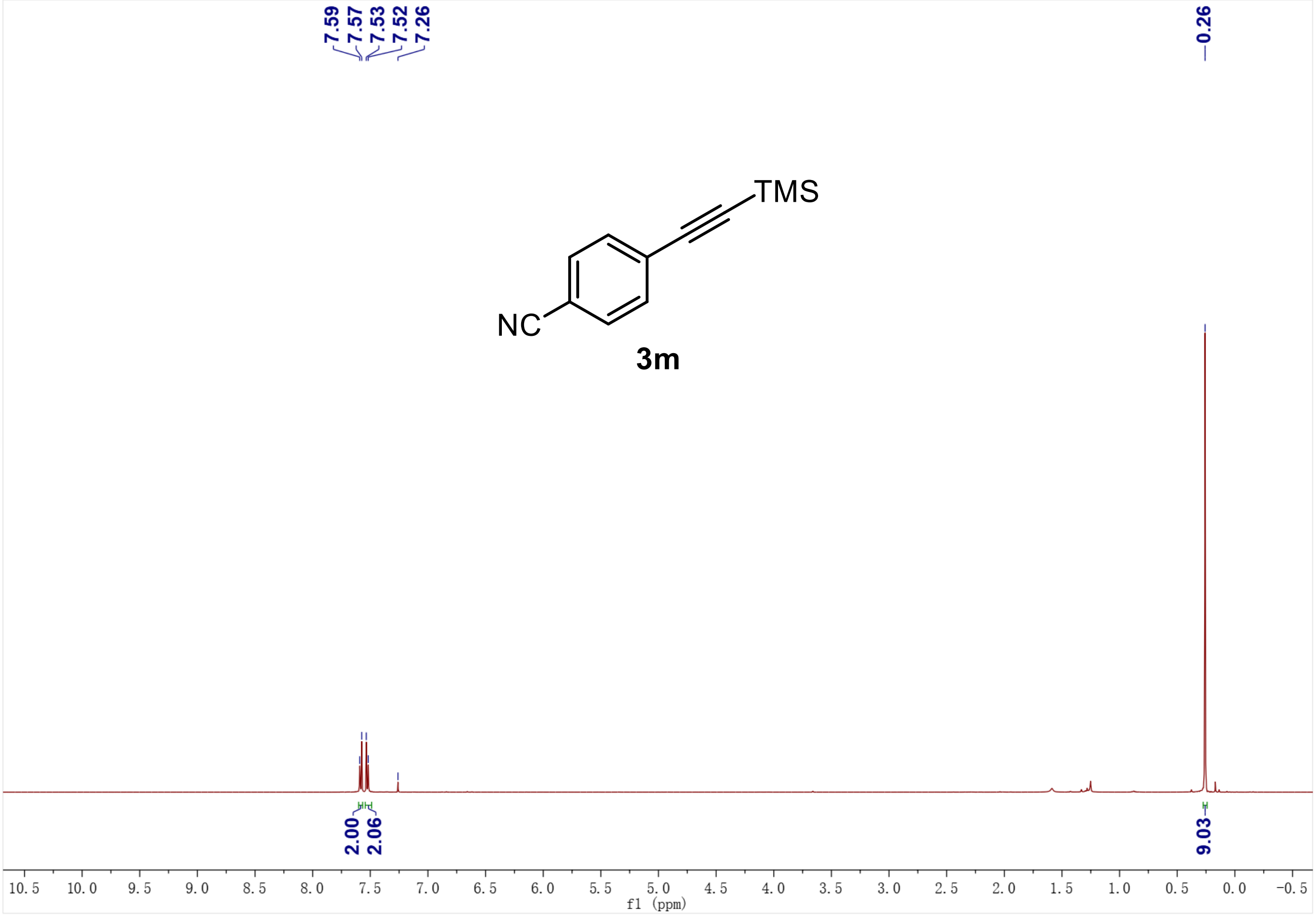

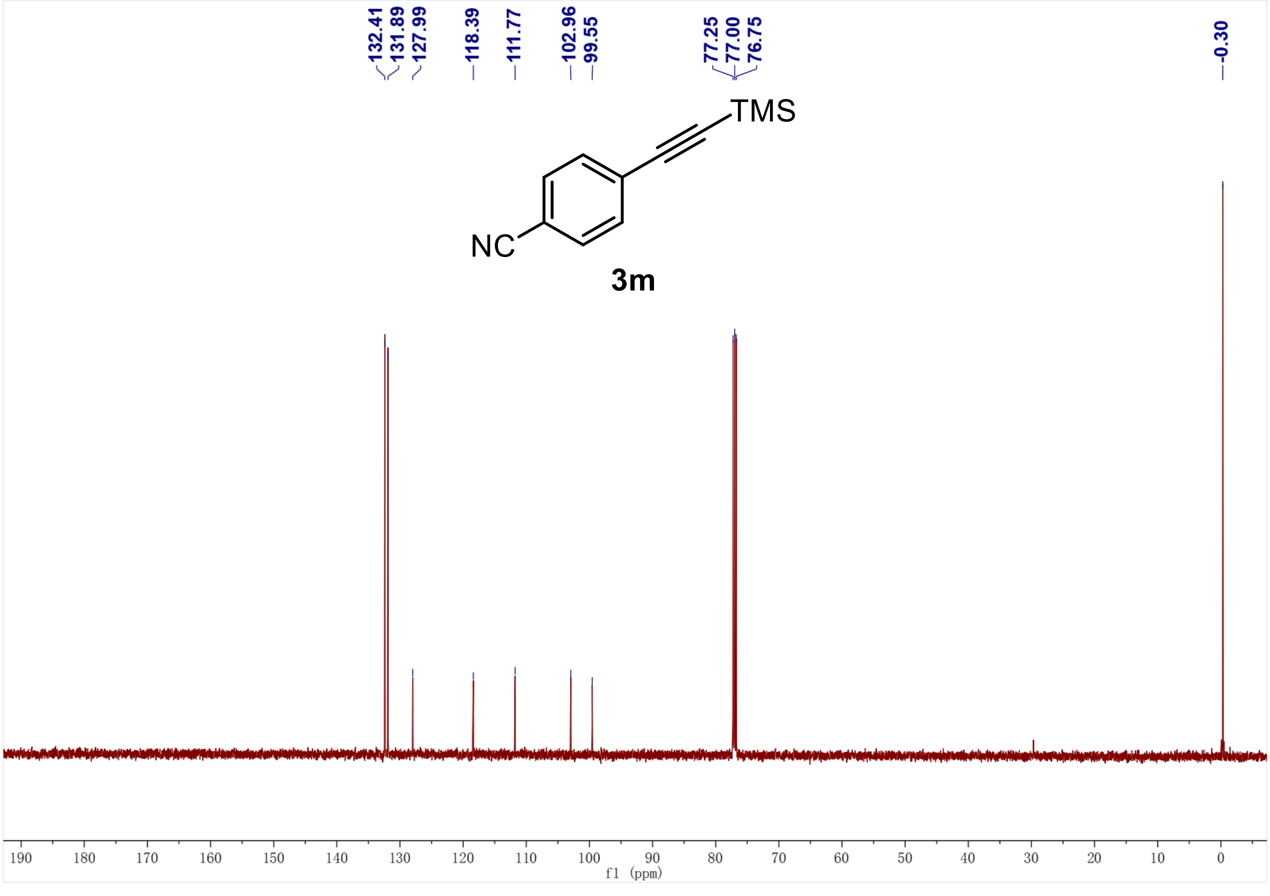

3n; ^{1}H NMR (500 MHz, $CDCl_3$); ^{13}C NMR (126 MHz, $CDCl_3$)

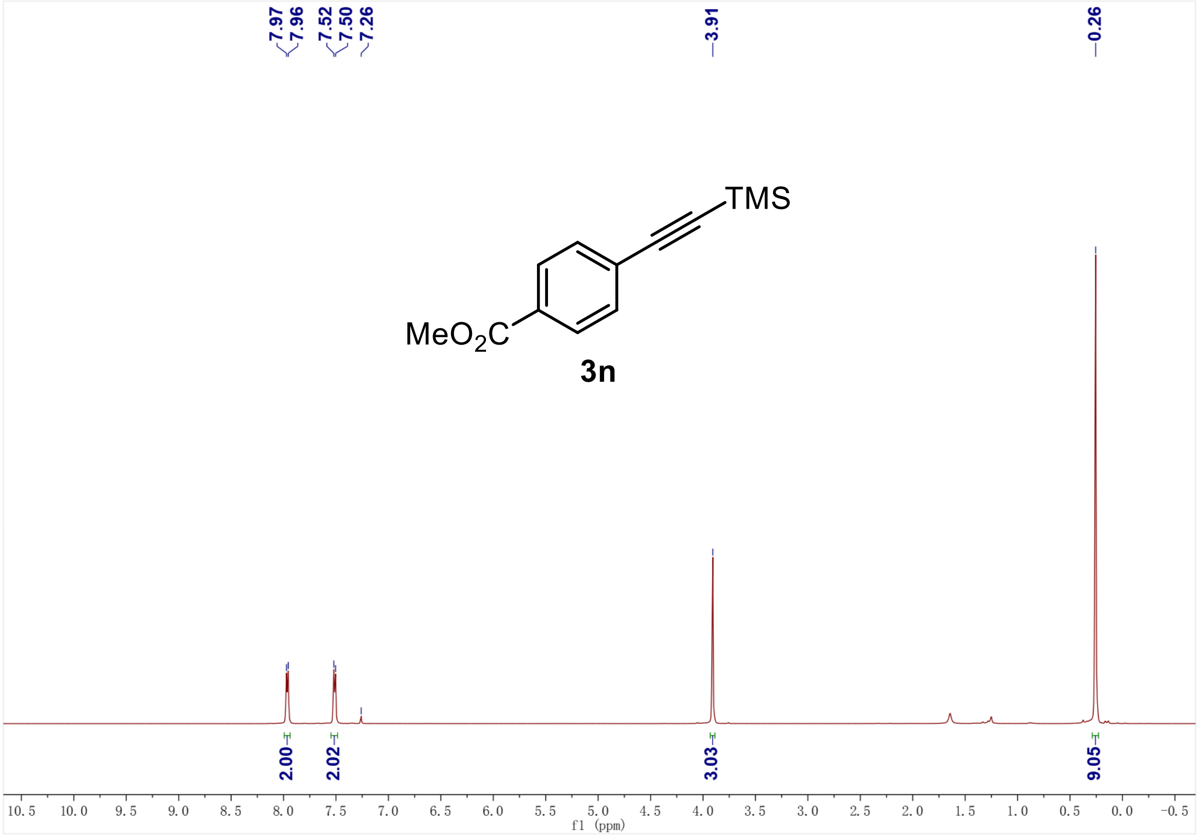

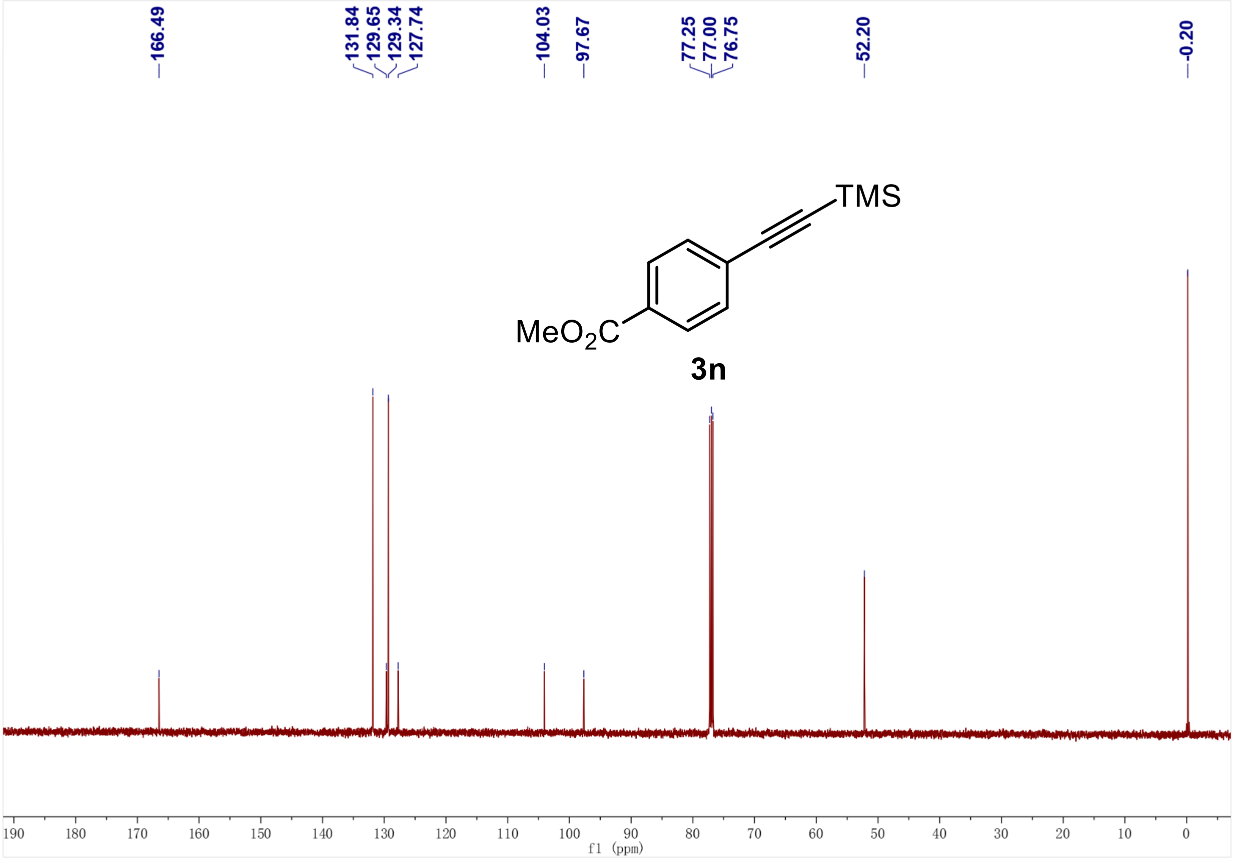

3o; ^{1}H NMR (500 MHz, $CDCl_3$); ^{13}C NMR (126 MHz, $CDCl_3$)

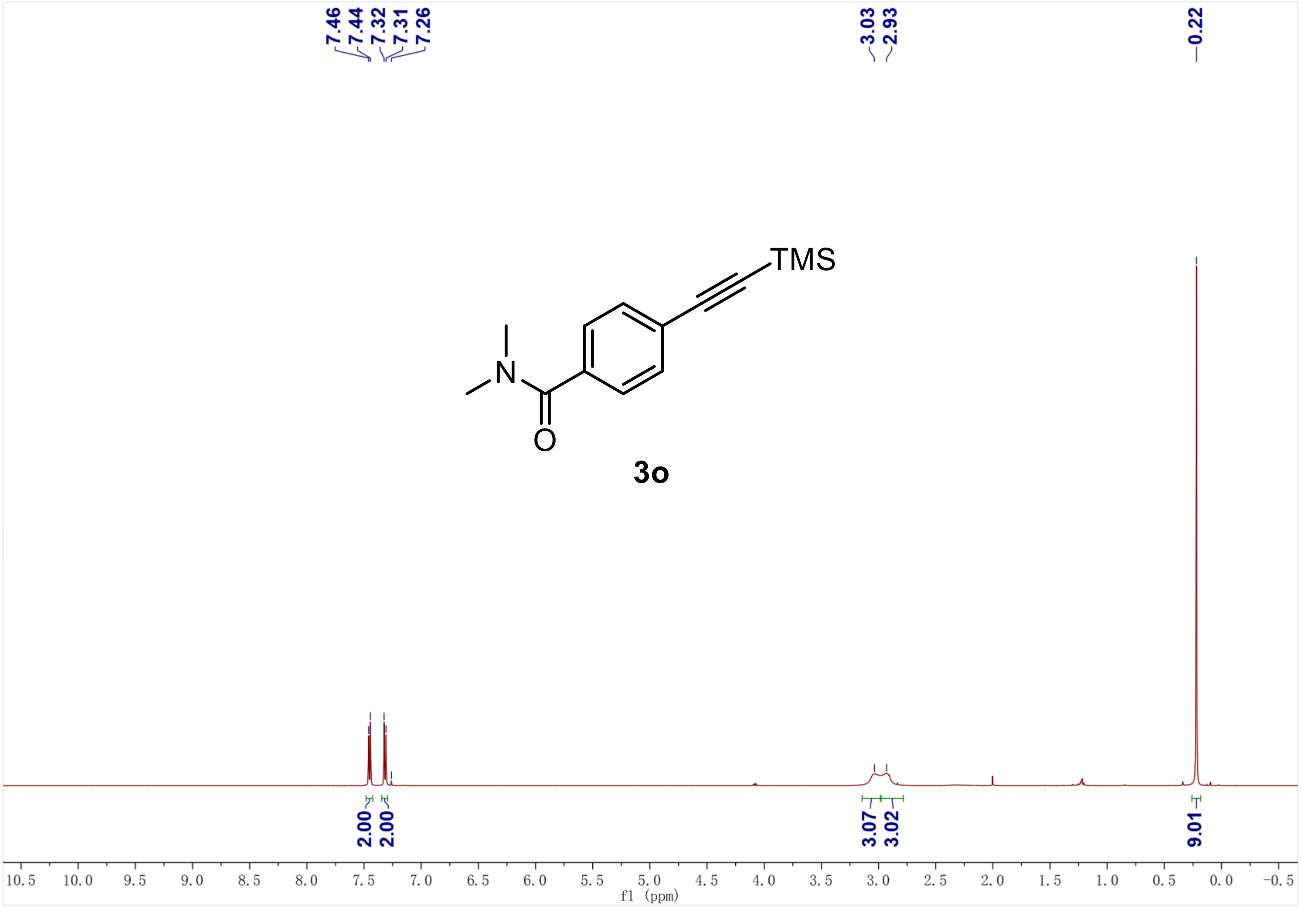

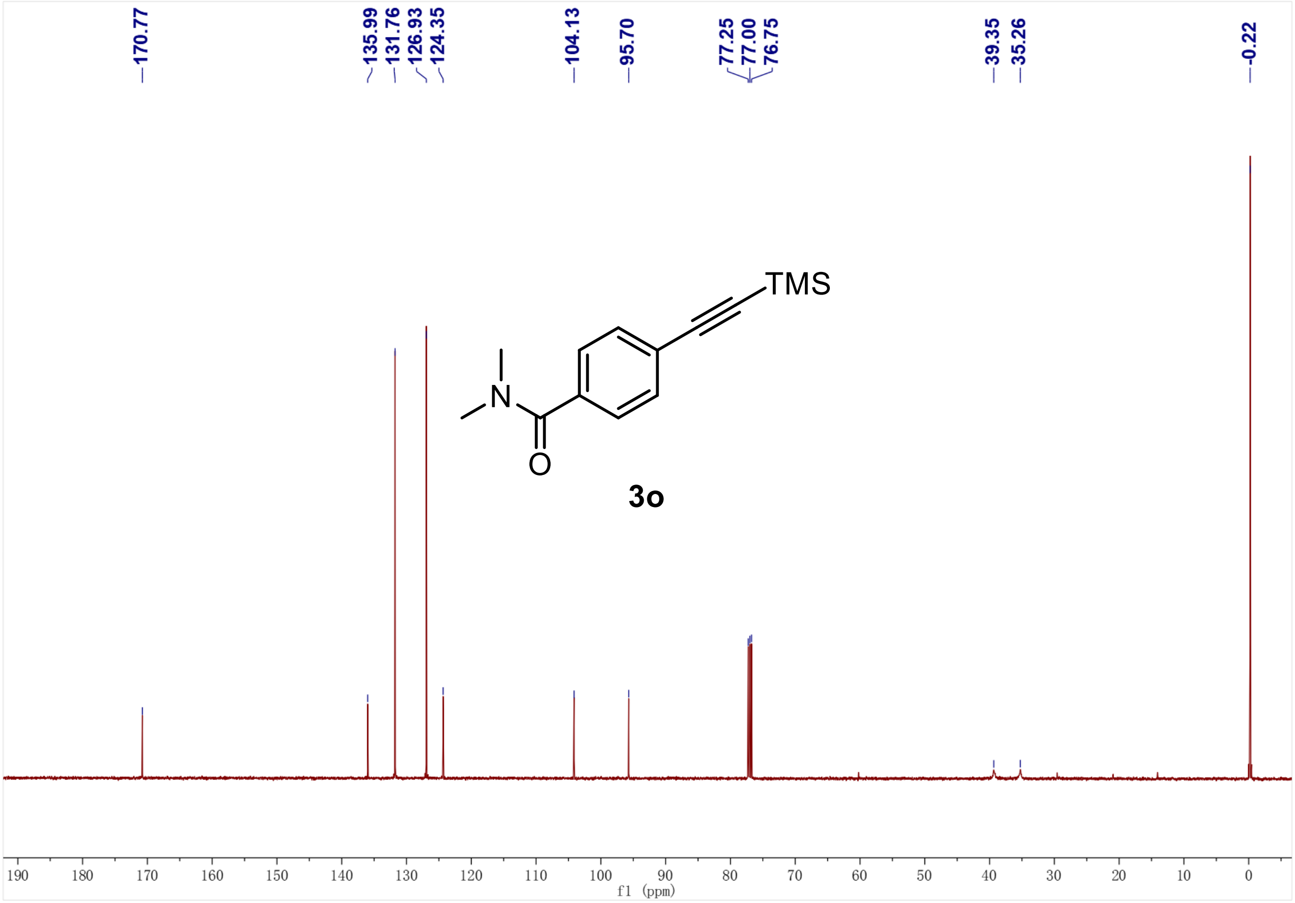

3p; ^{1}H NMR (500 MHz, $CDCl_3$); ^{13}C NMR (126 MHz, $CDCl_3$)

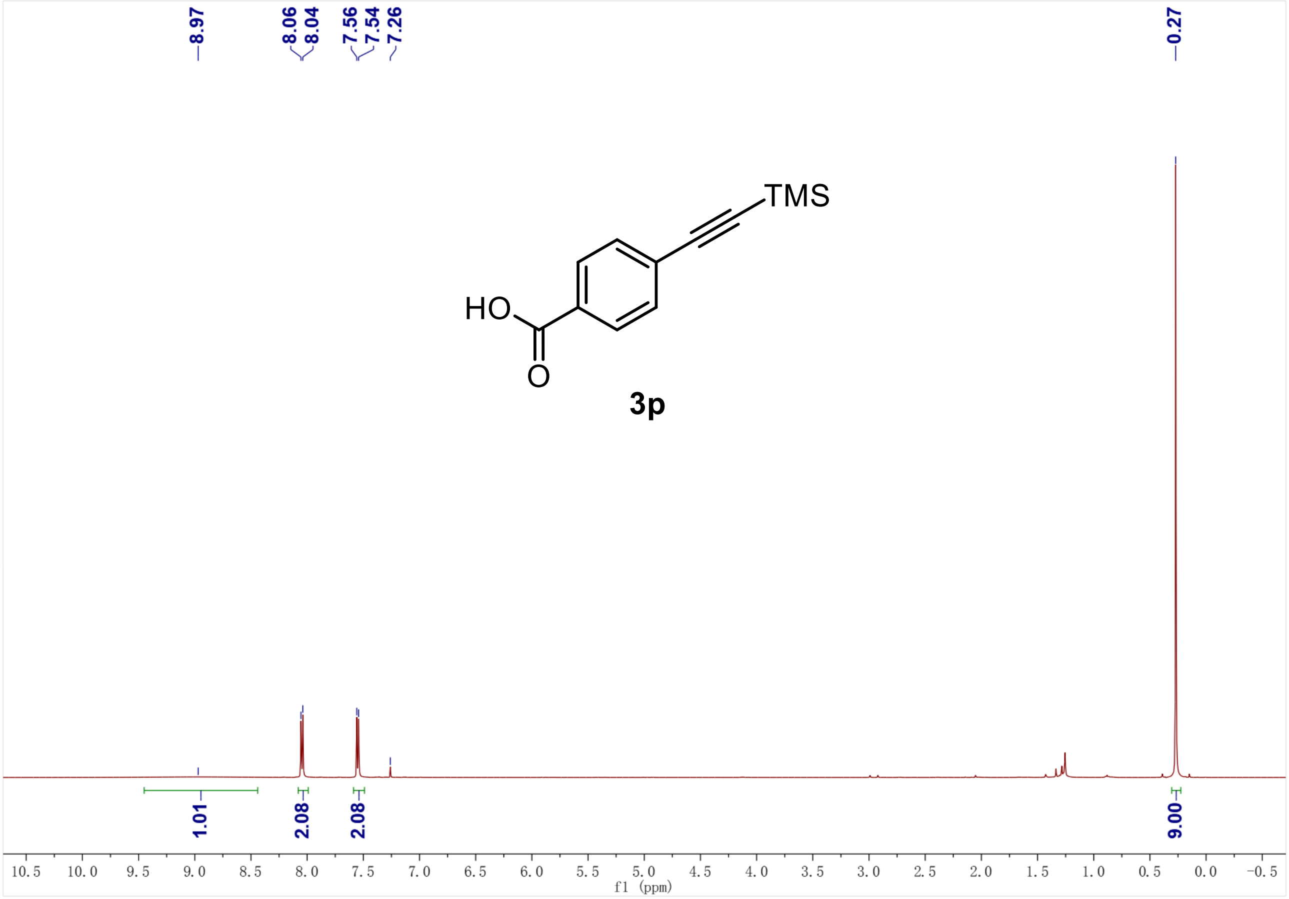

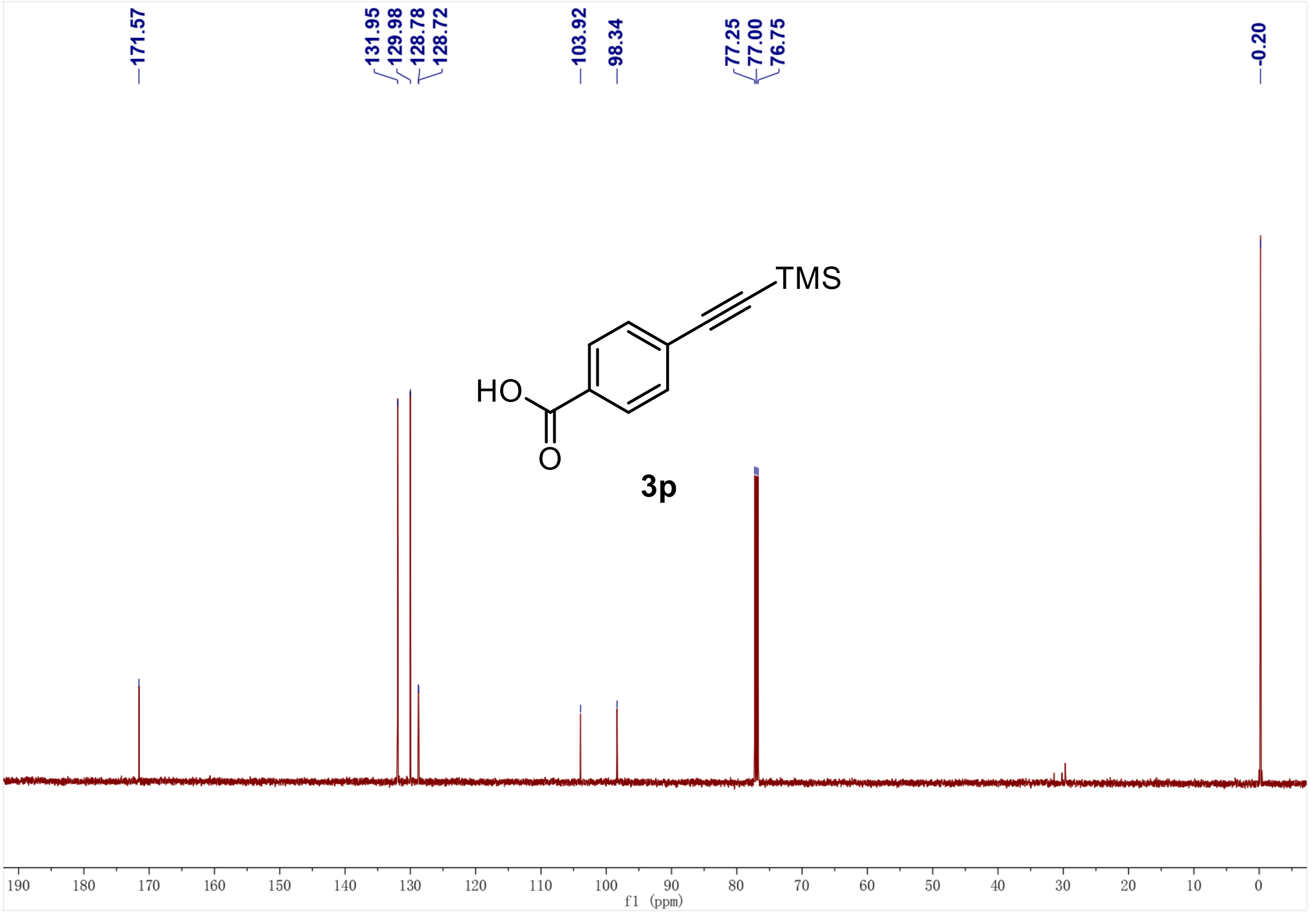

3q; ^{1}H NMR (500 MHz, $CDCl_3$); ^{13}C NMR (126 MHz, $CDCl_3$)

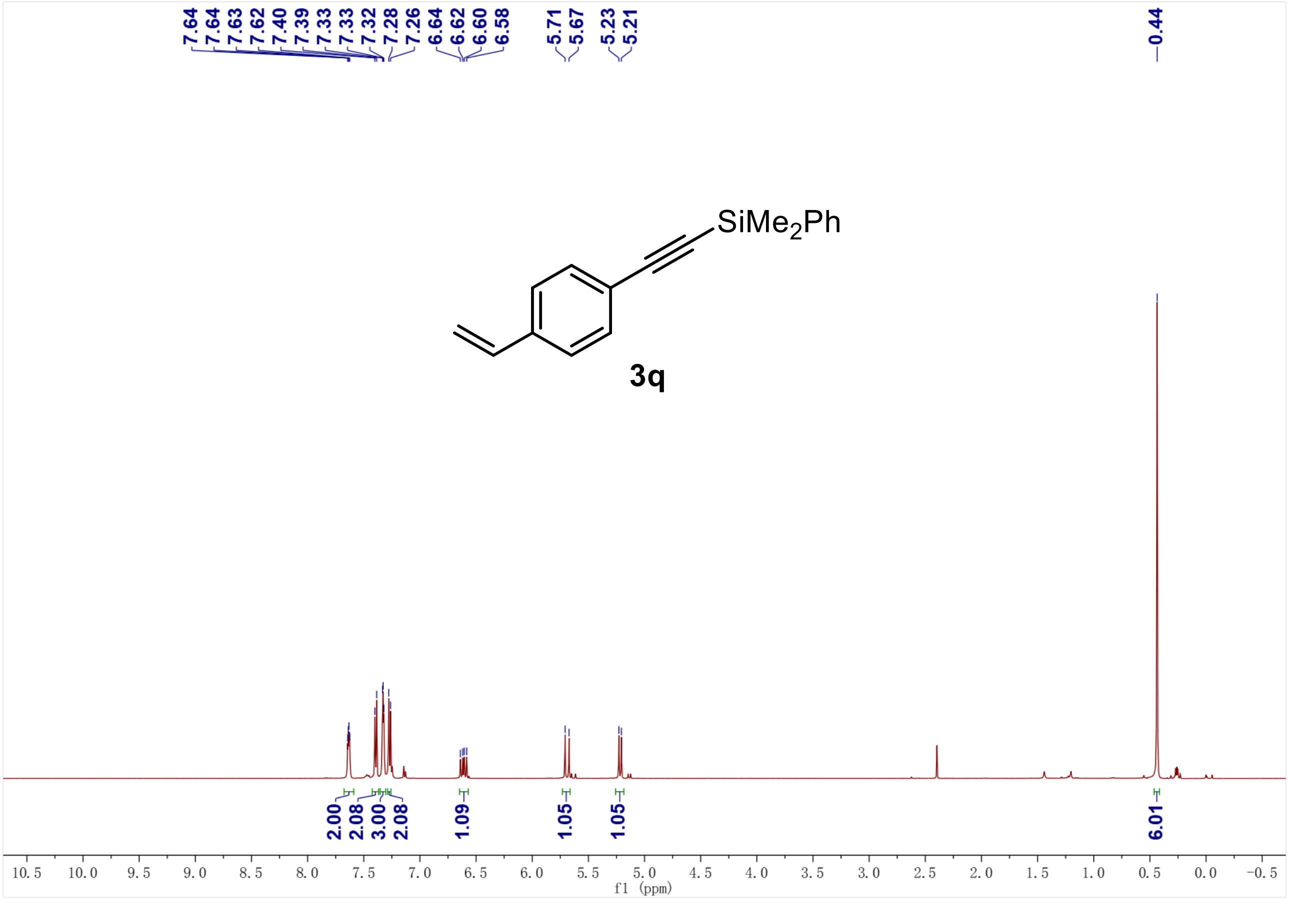

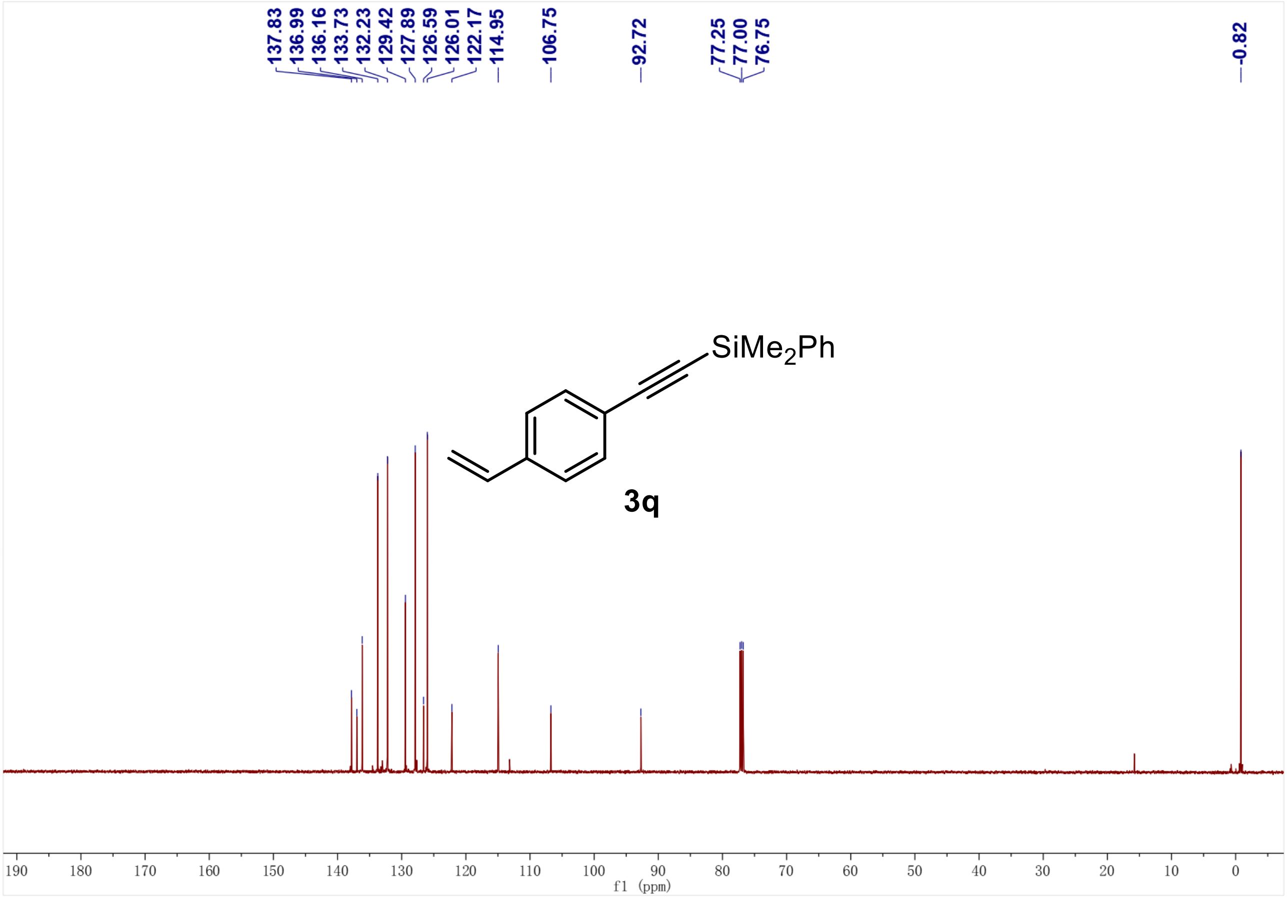

3r; ^{1}H NMR (400 MHz, $CDCl_3$); ^{13}C NMR (126 MHz, $CDCl_3$)

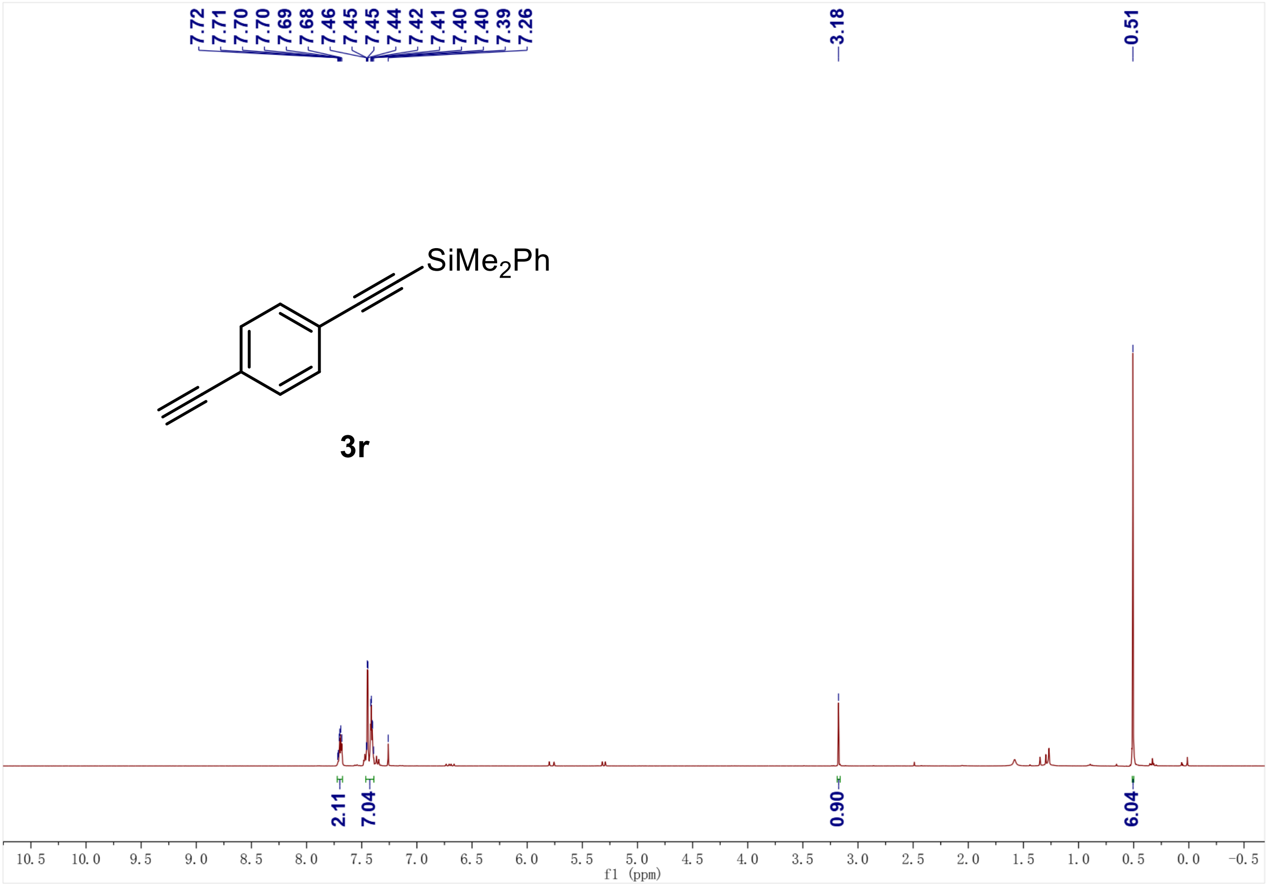

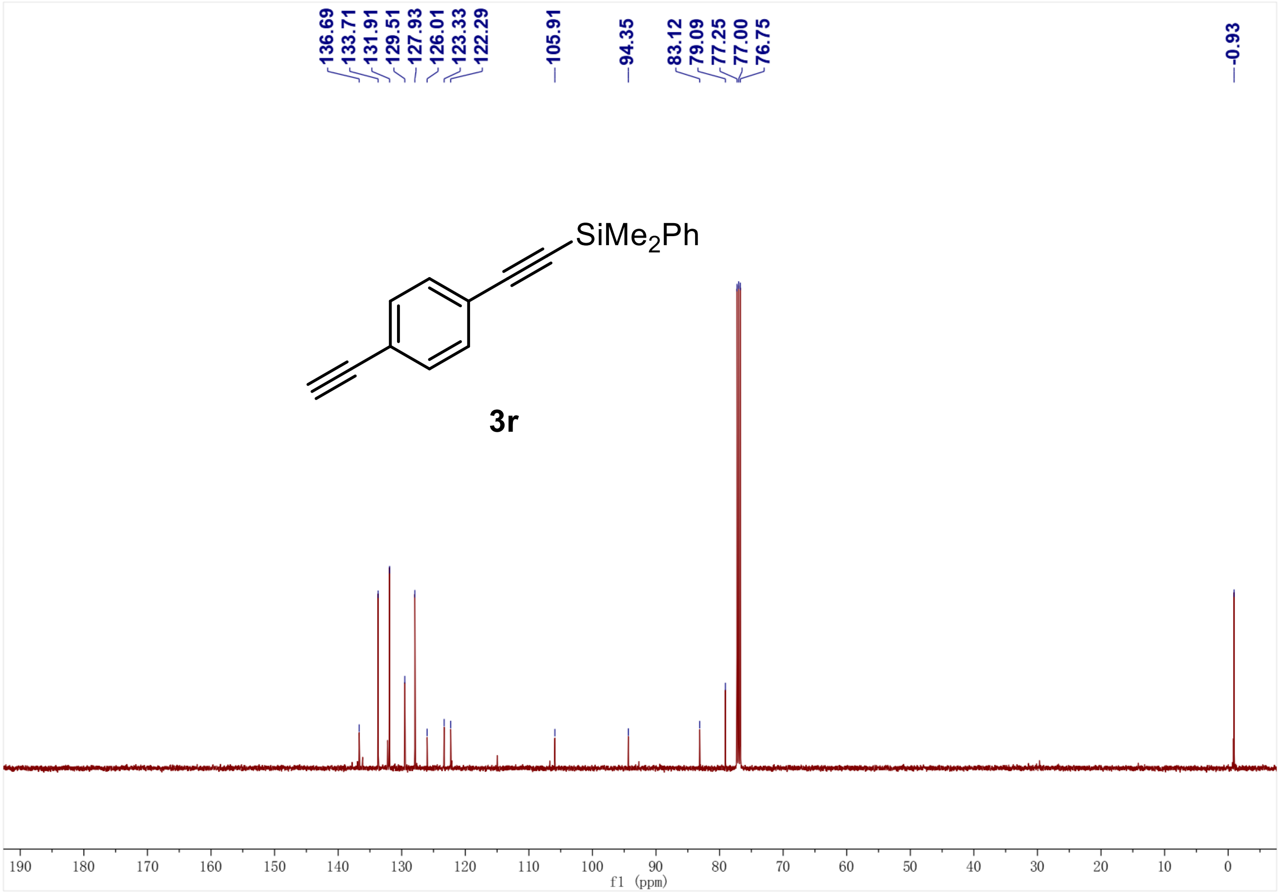

3s; ^{1}H NMR (400 MHz, $CDCl_3$); ^{13}C NMR (126 MHz, $CDCl_3$)

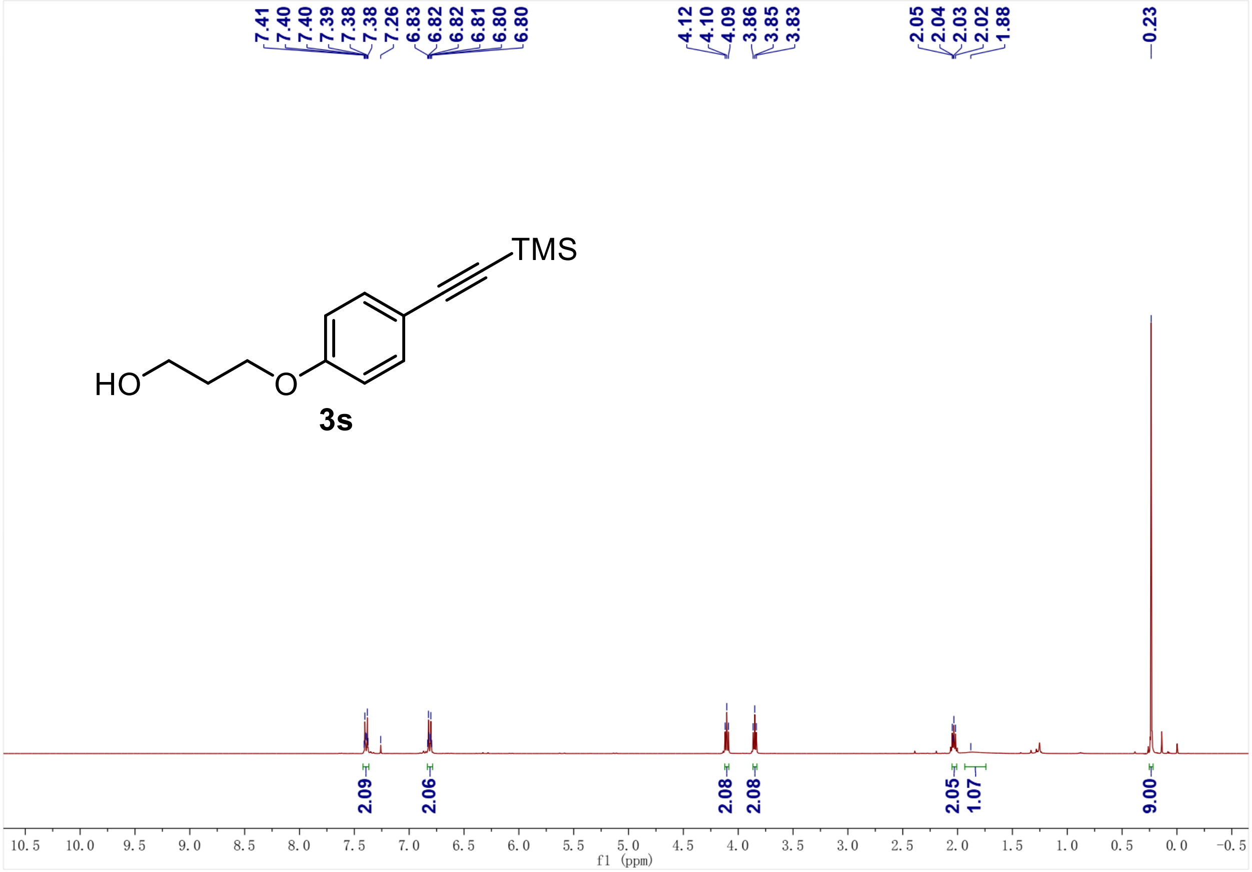

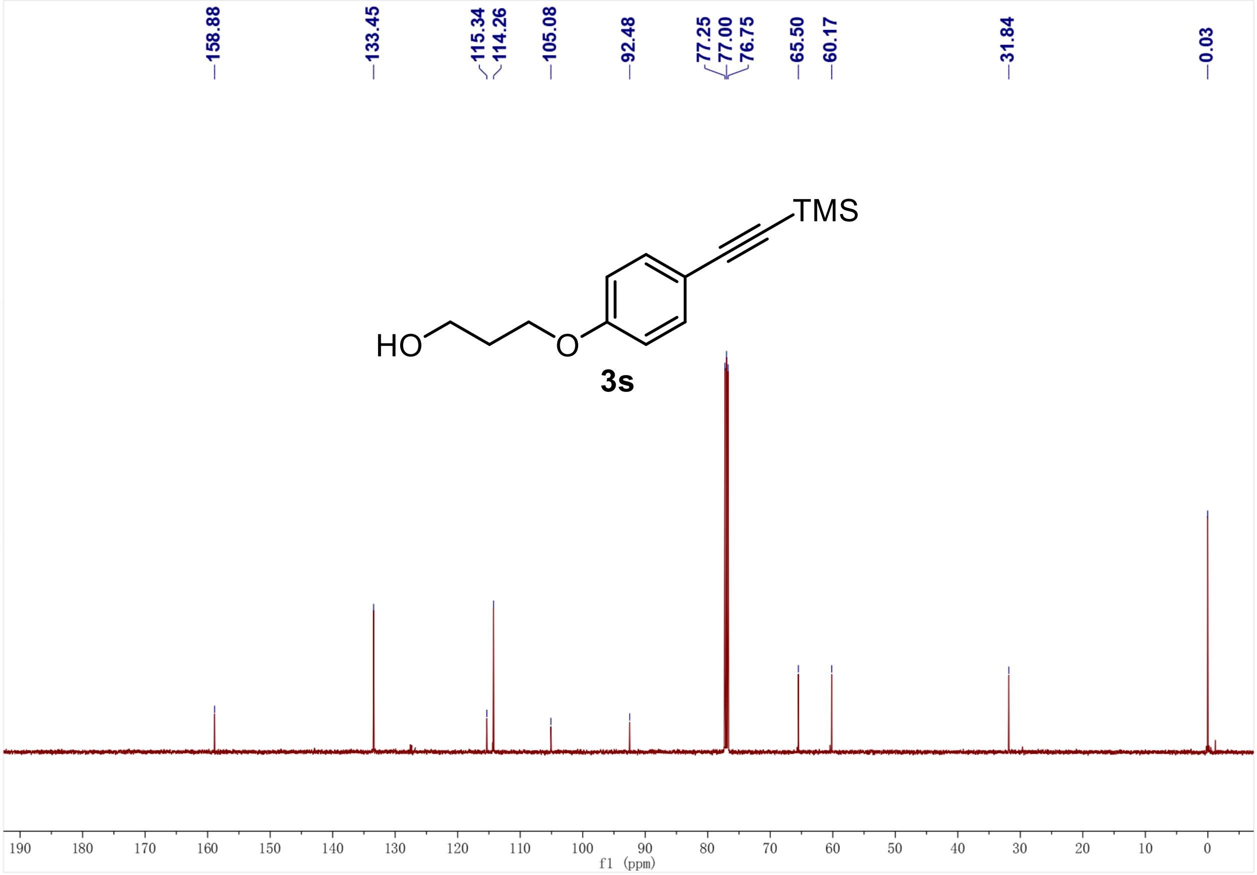

3t; ^{1}H NMR (500 MHz, $CDCl_3$); ^{13}C NMR (126 MHz, $CDCl_3$)

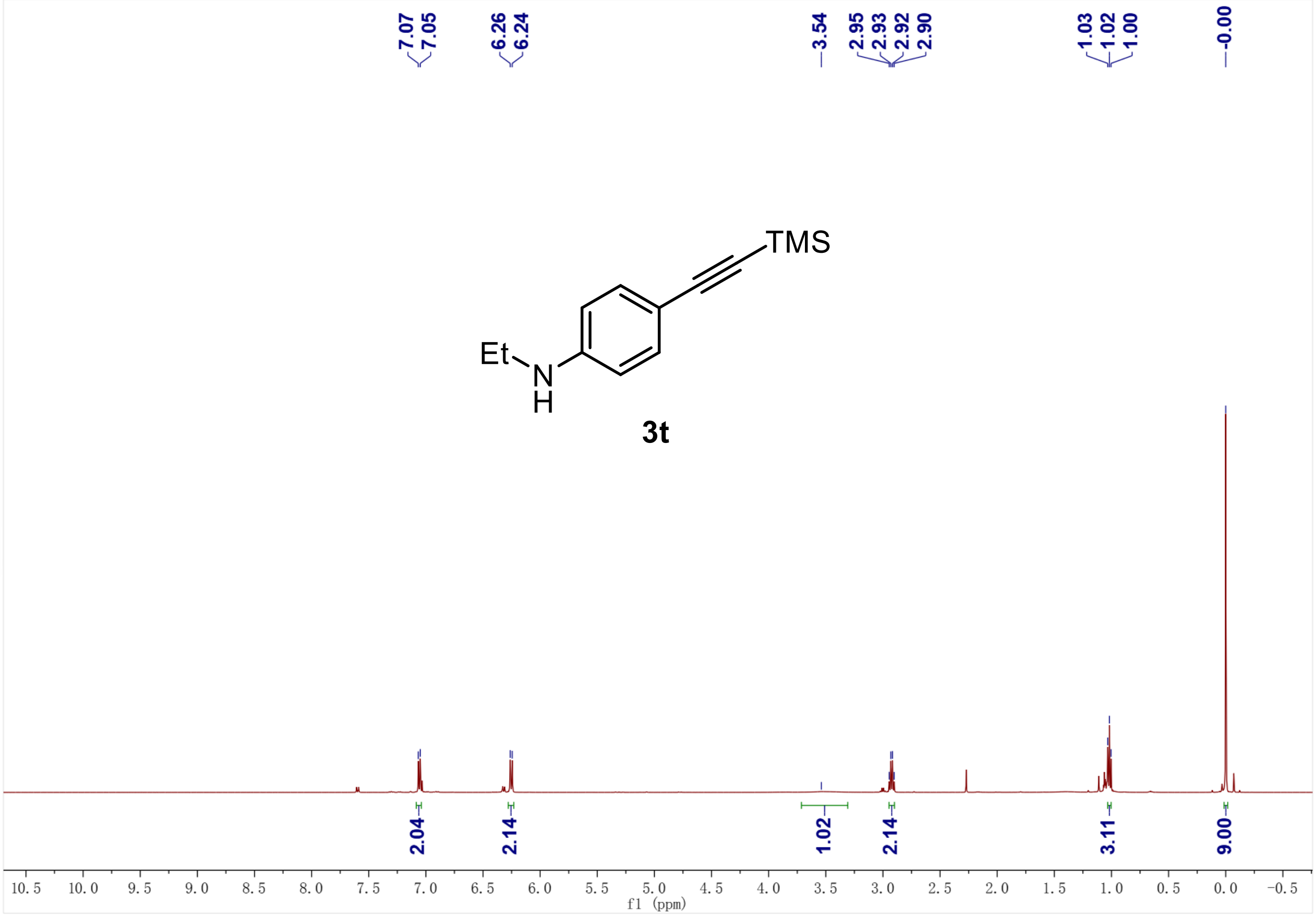

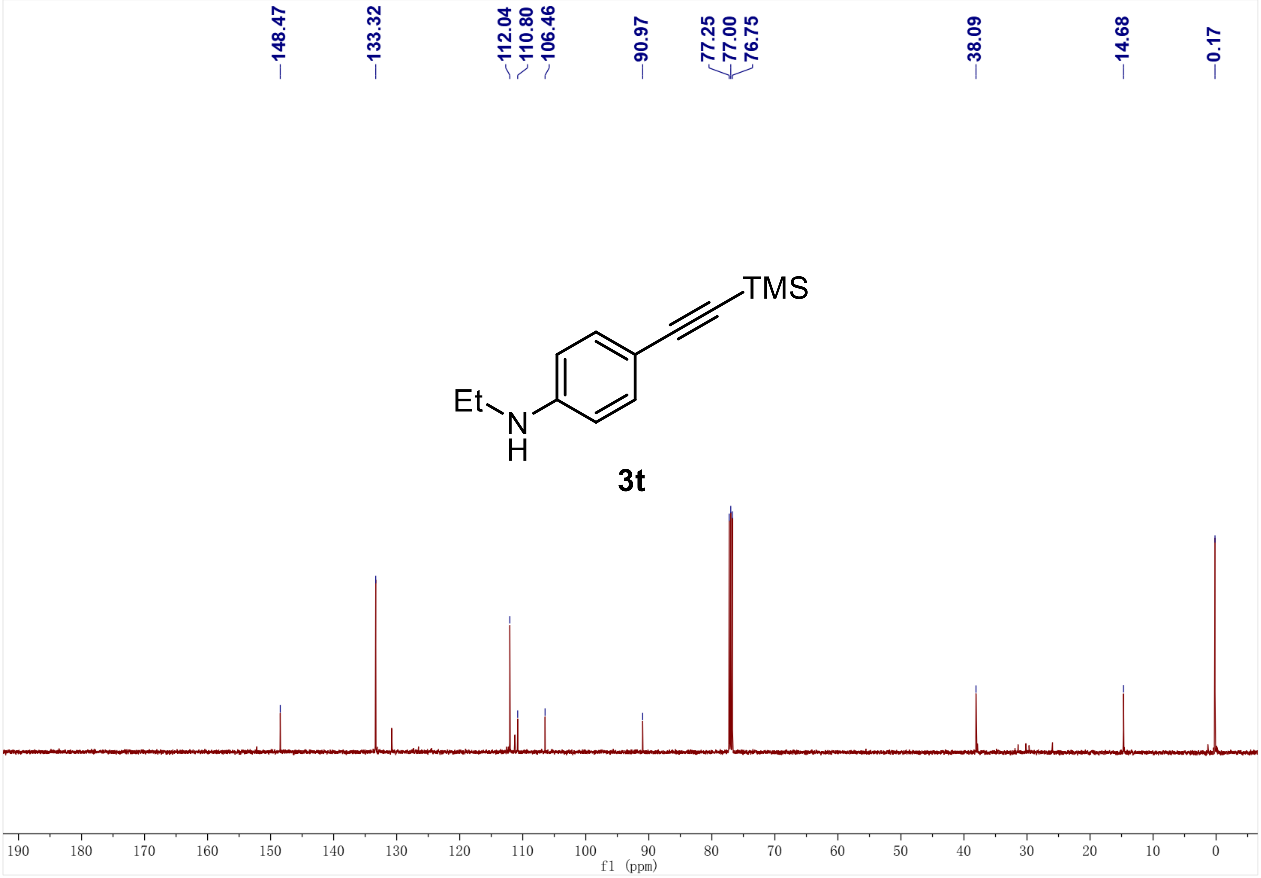

3u; ^{1}H NMR (500 MHz, $CDCl_3$); ^{13}C NMR (126 MHz, $CDCl_3$)

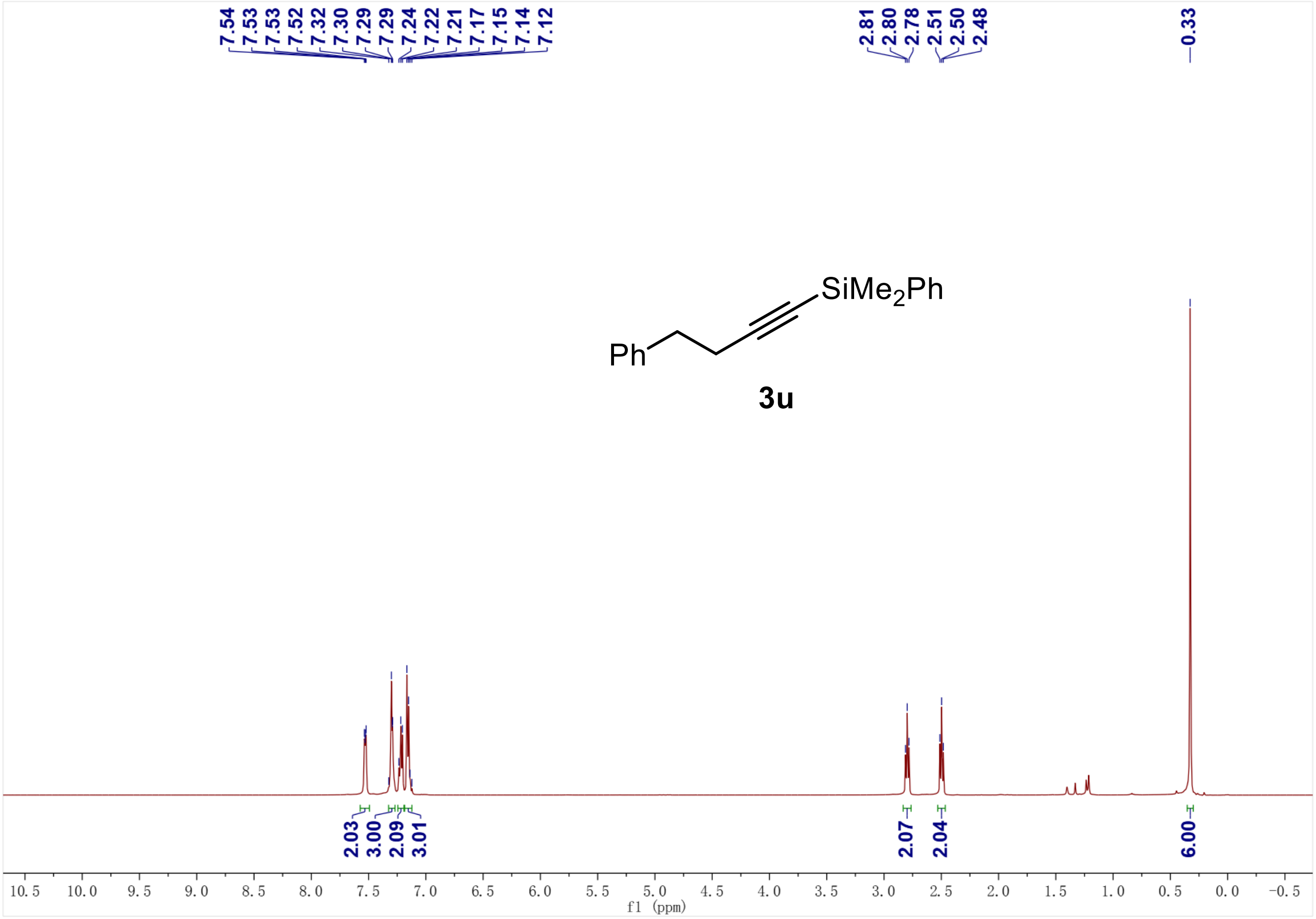

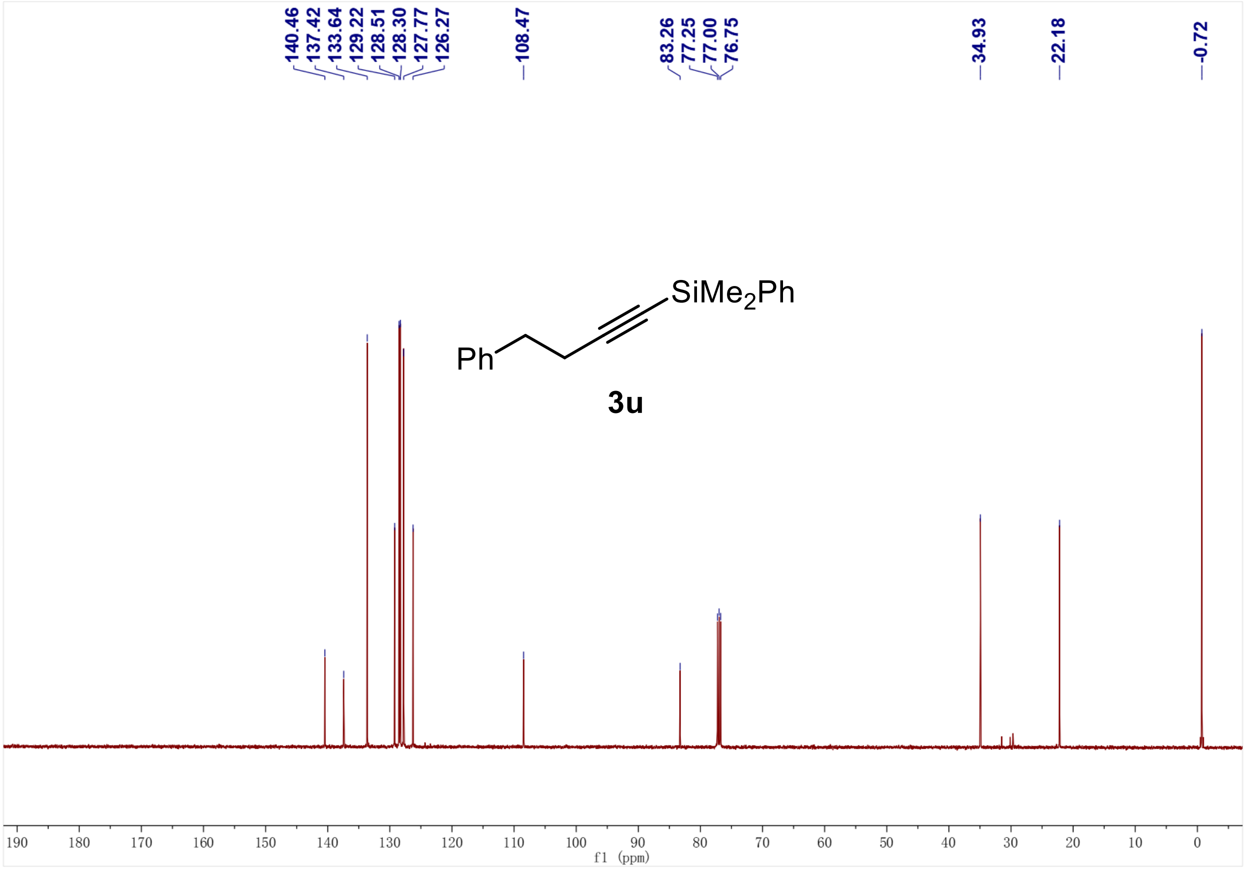

3v; ^{1}H NMR (400 MHz, $CDCl_3$); ^{13}C NMR (101 MHz, $CDCl_3$)

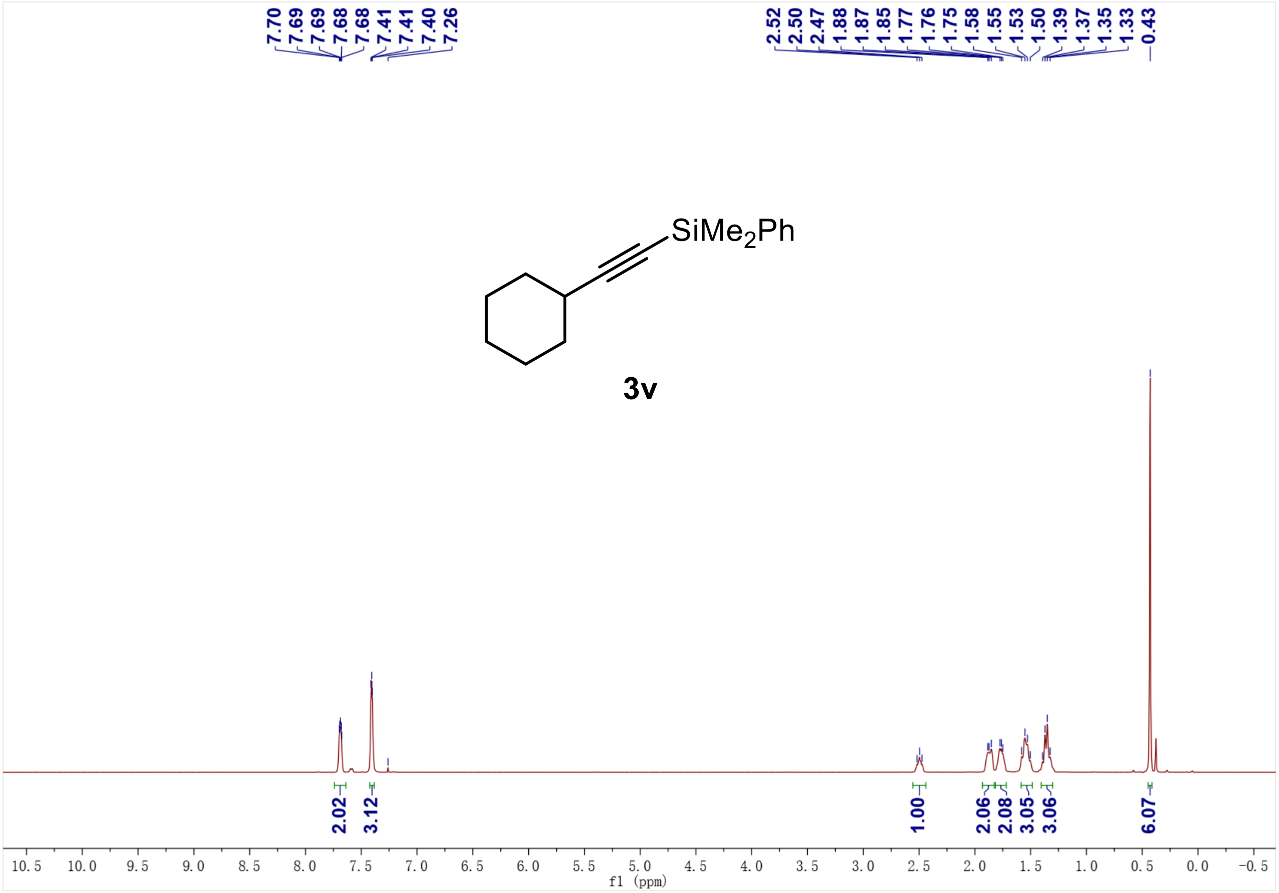

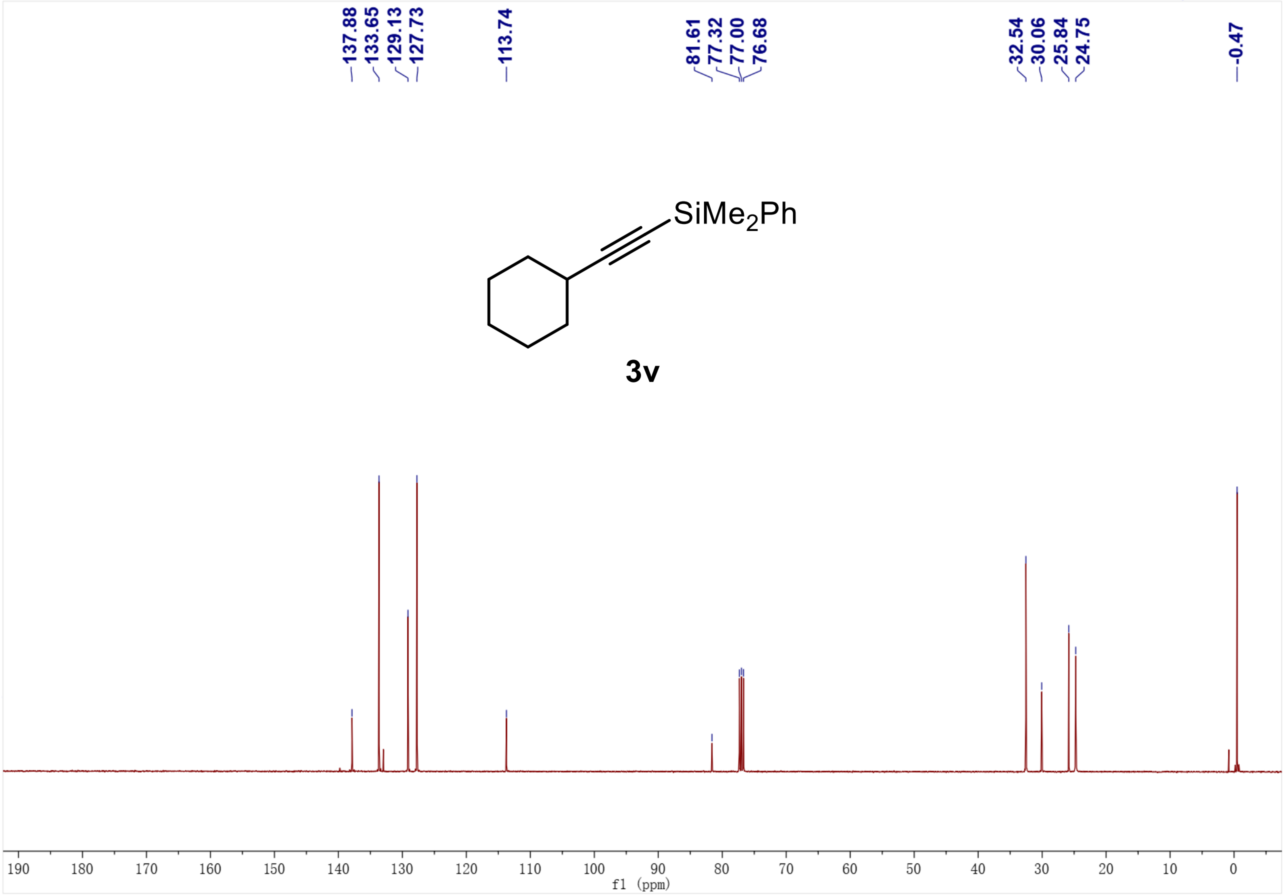

3w; ^{1}H NMR (400 MHz, $CDCl_3$); ^{13}C NMR (101 MHz, $CDCl_3$)

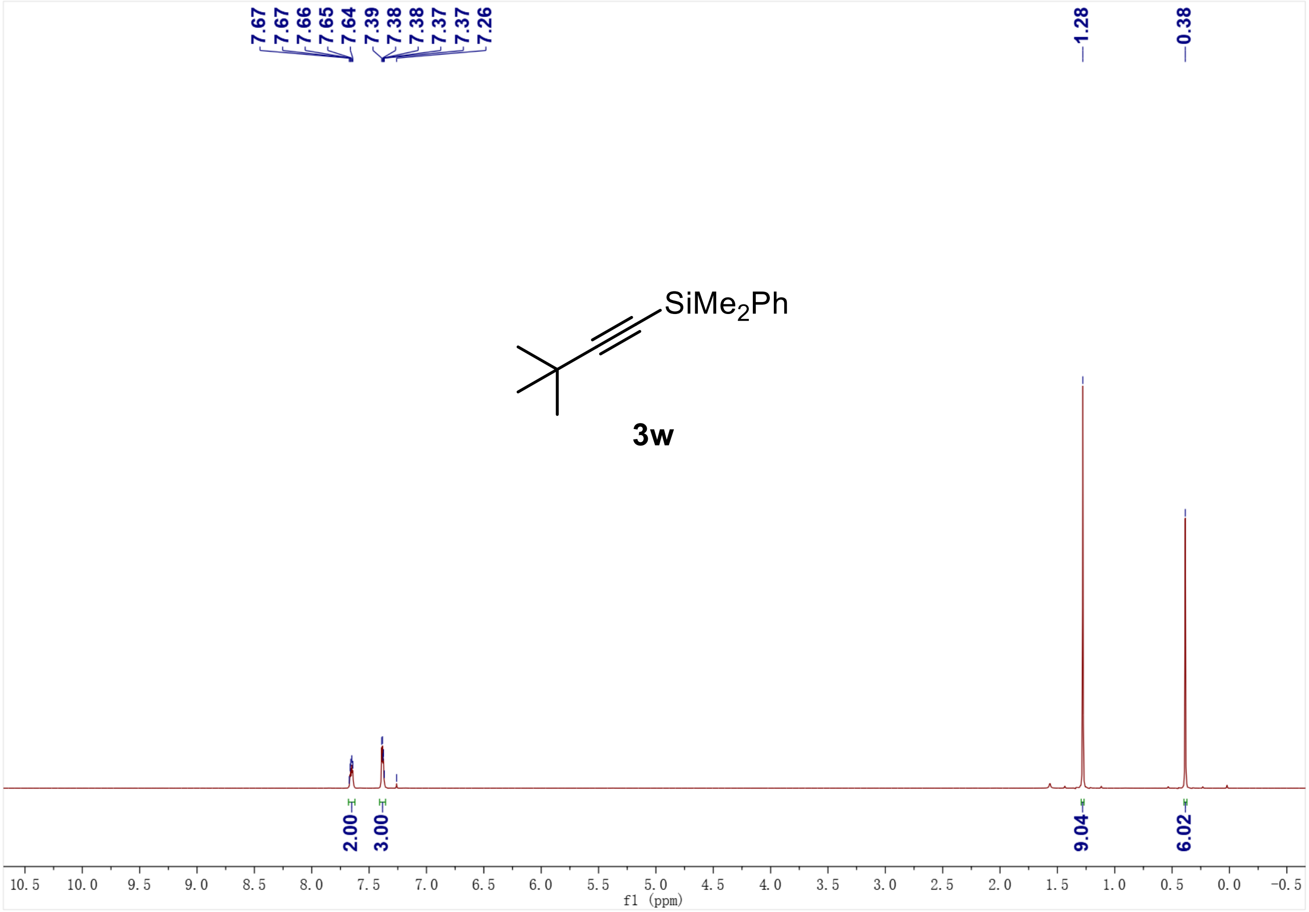

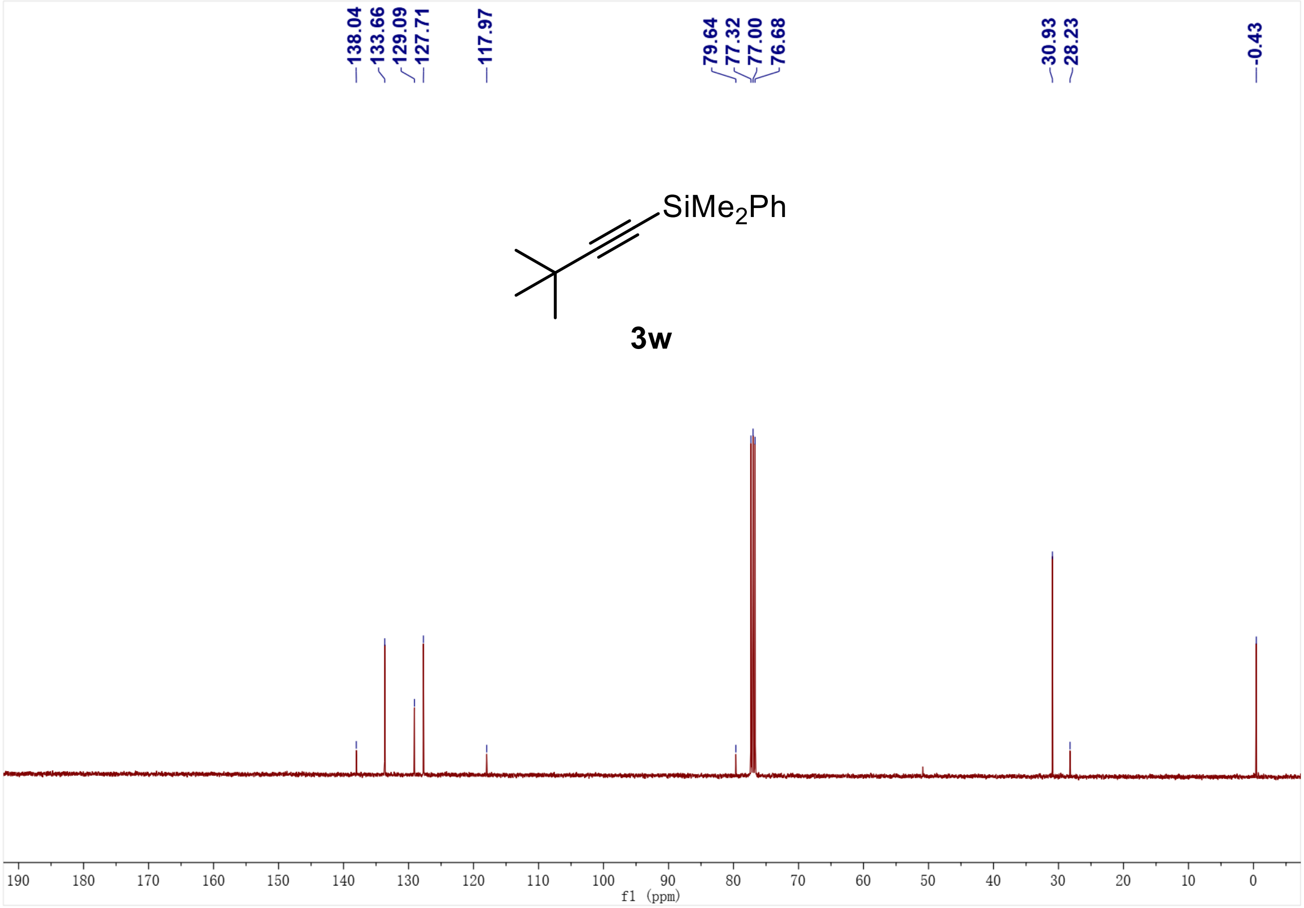

3x; ^{1}H NMR (400 MHz, $CDCl_3$); ^{13}C NMR (101 MHz, $CDCl_3$)

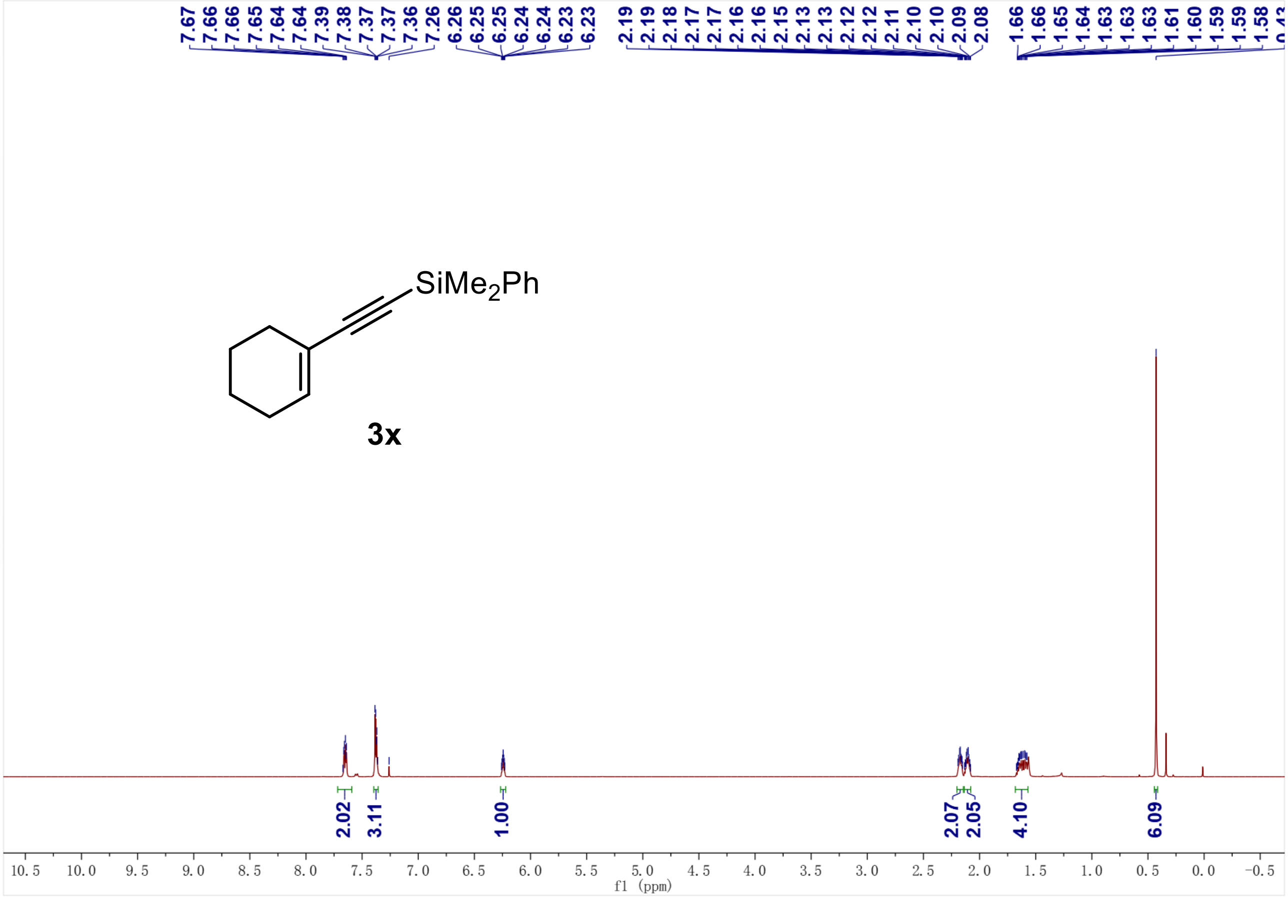

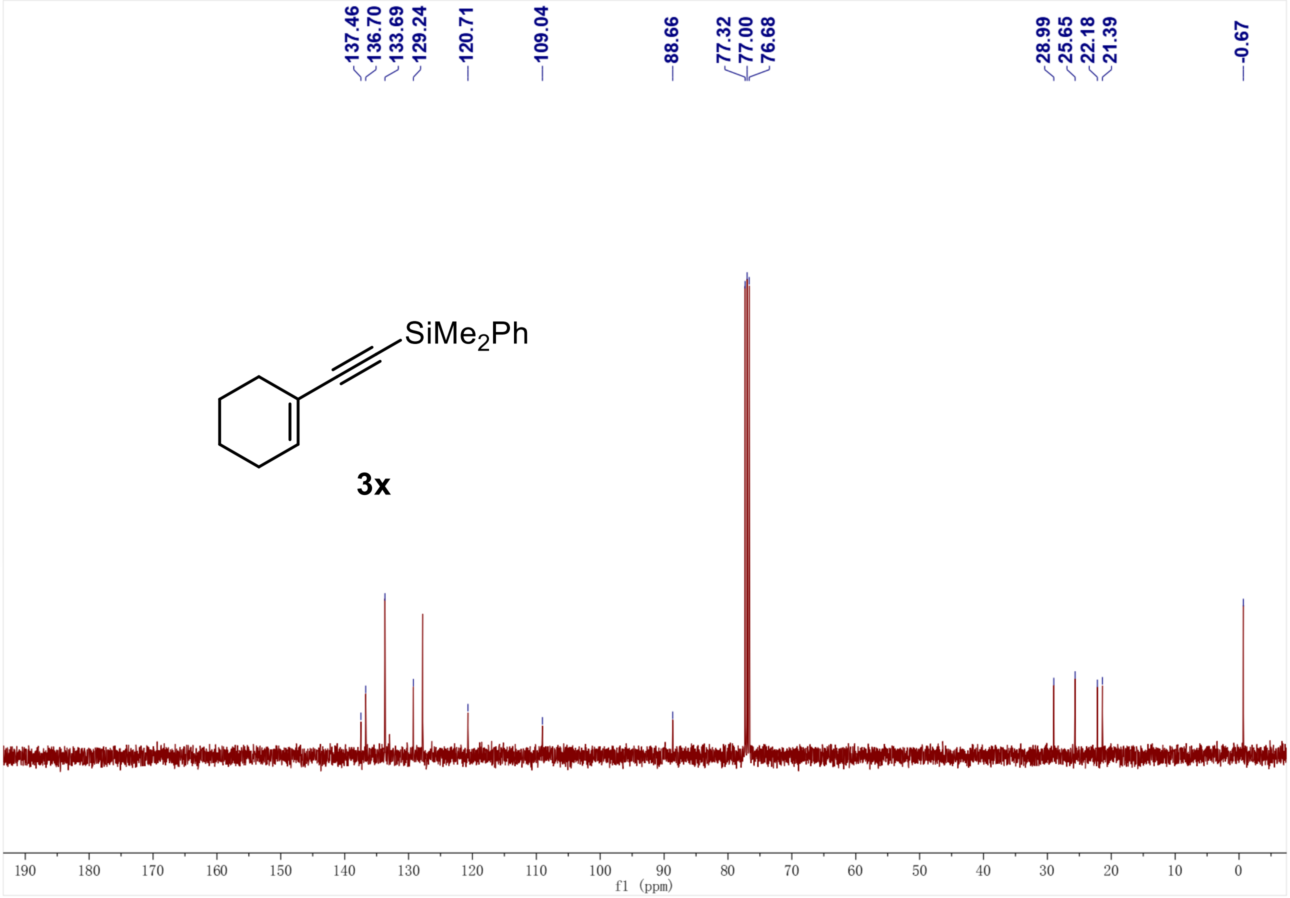

3y; ^{1}H NMR (500 MHz, $CDCl_3$); ^{13}C NMR (126 MHz, $CDCl_3$)

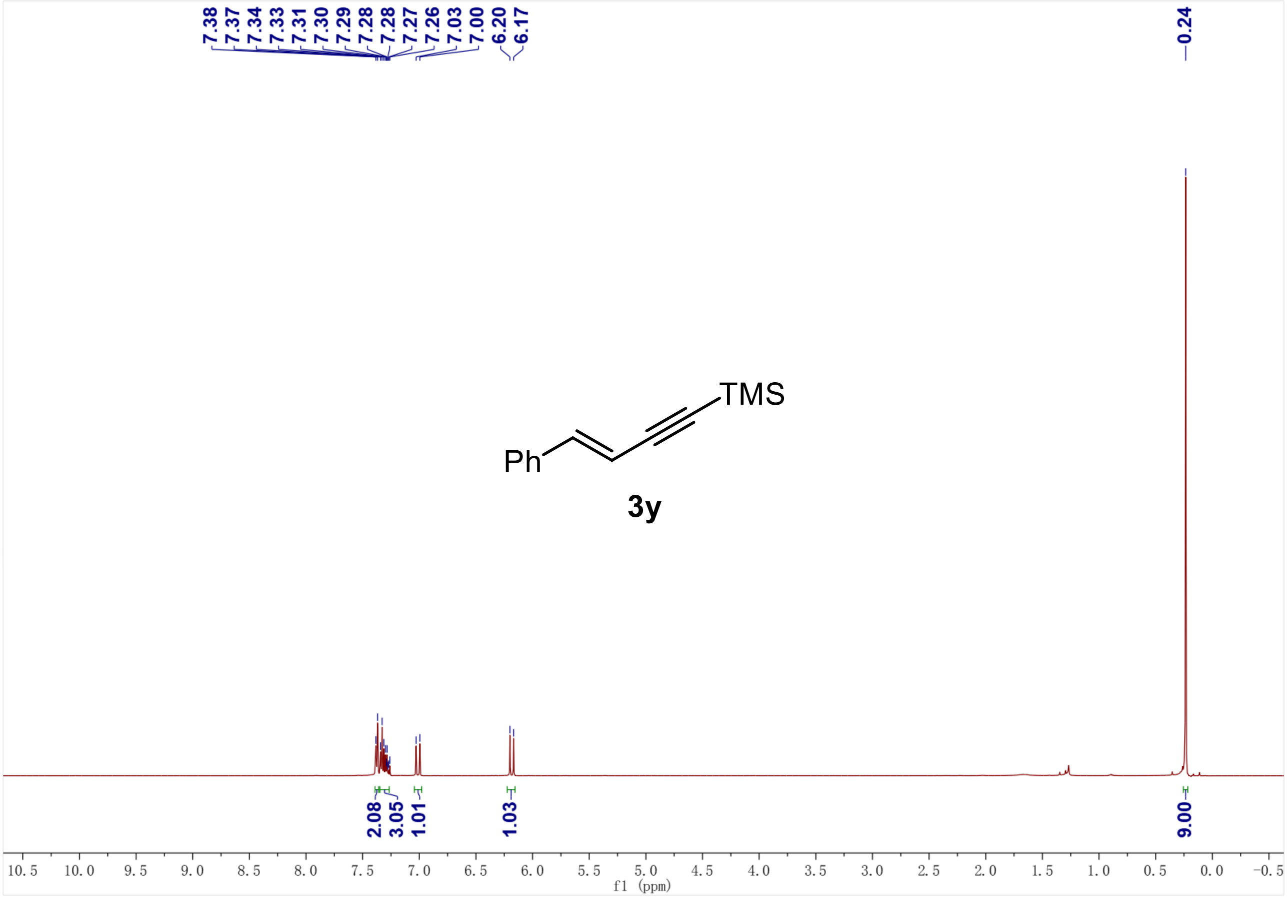

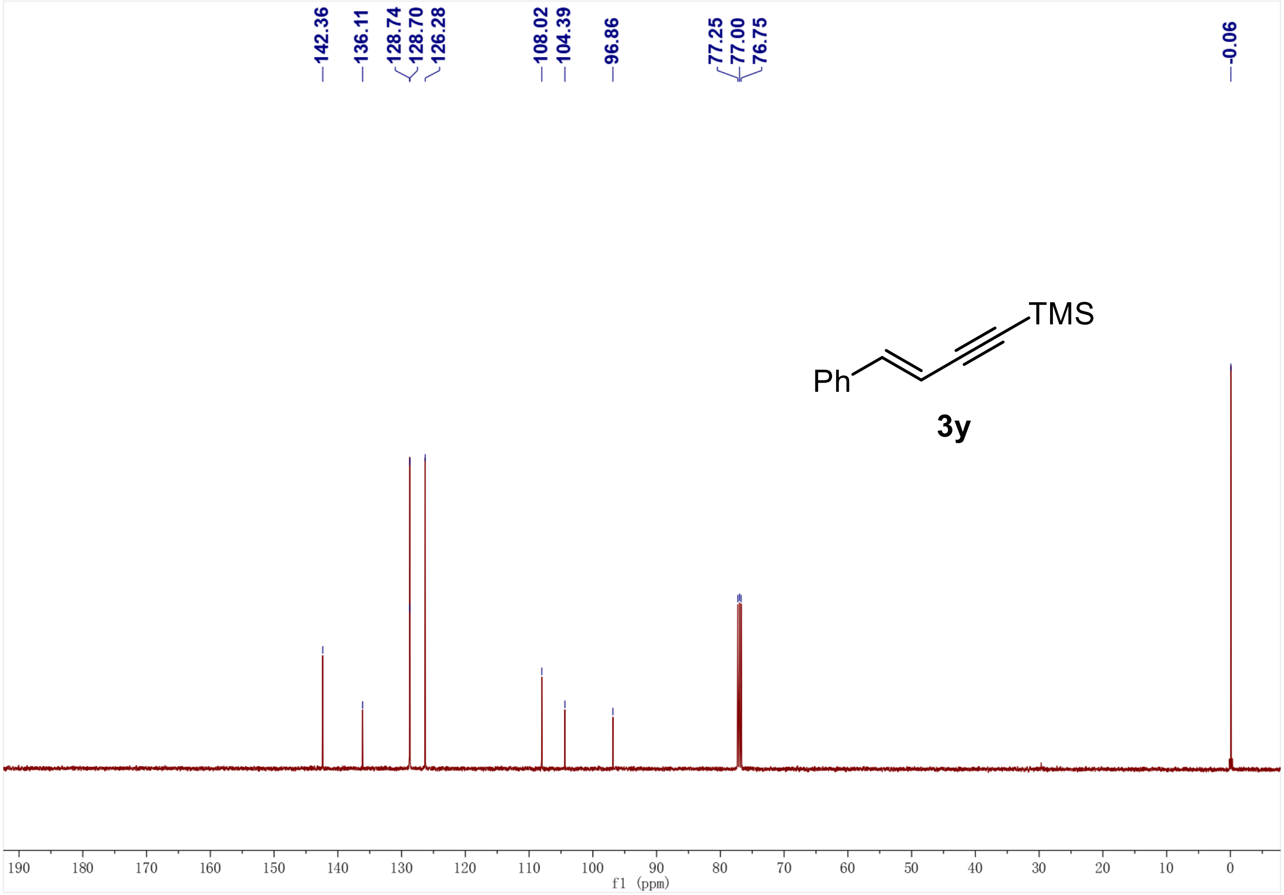

3z; ^{1}H NMR (500 MHz, $CDCl_3$); ^{13}C NMR (126 MHz, $CDCl_3$)

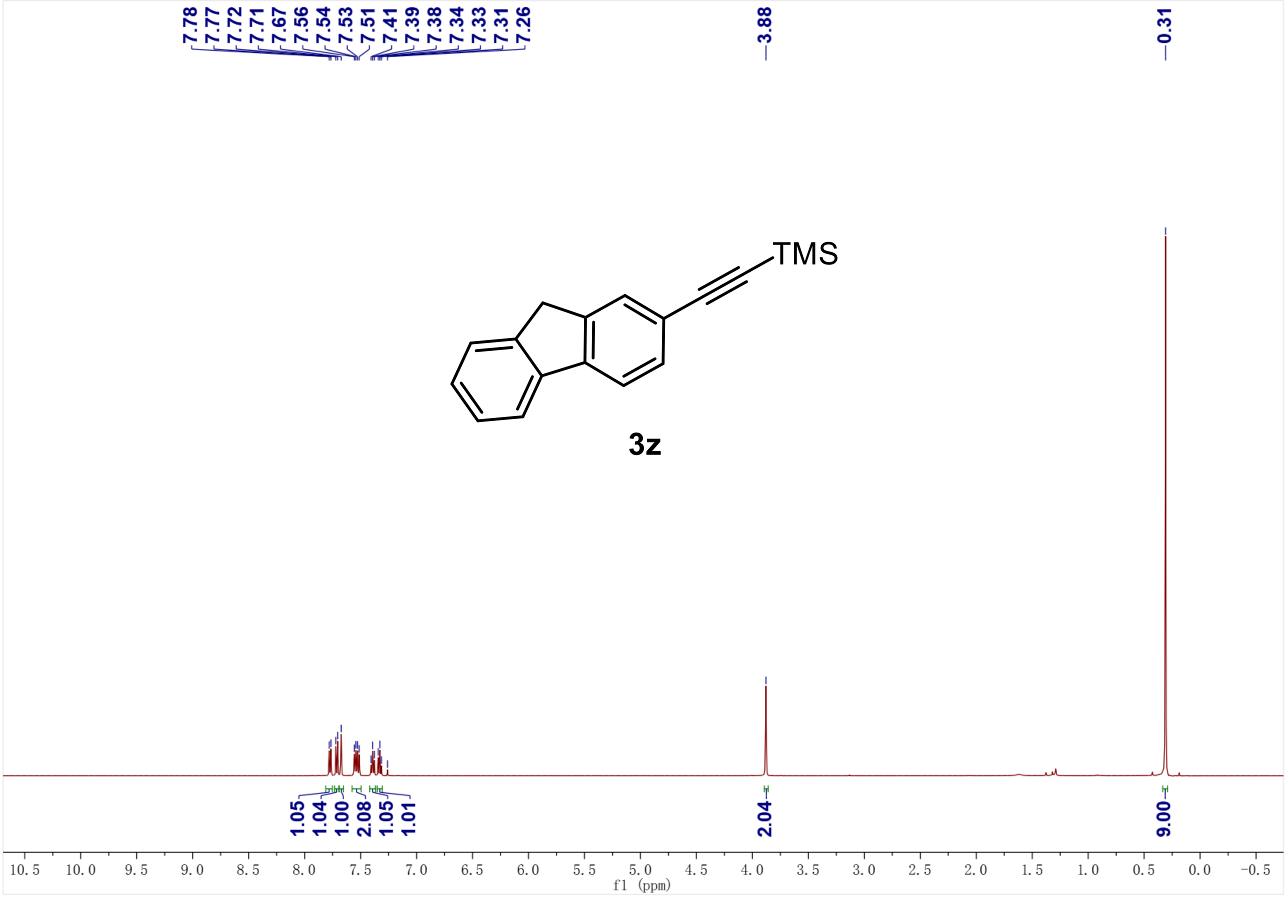

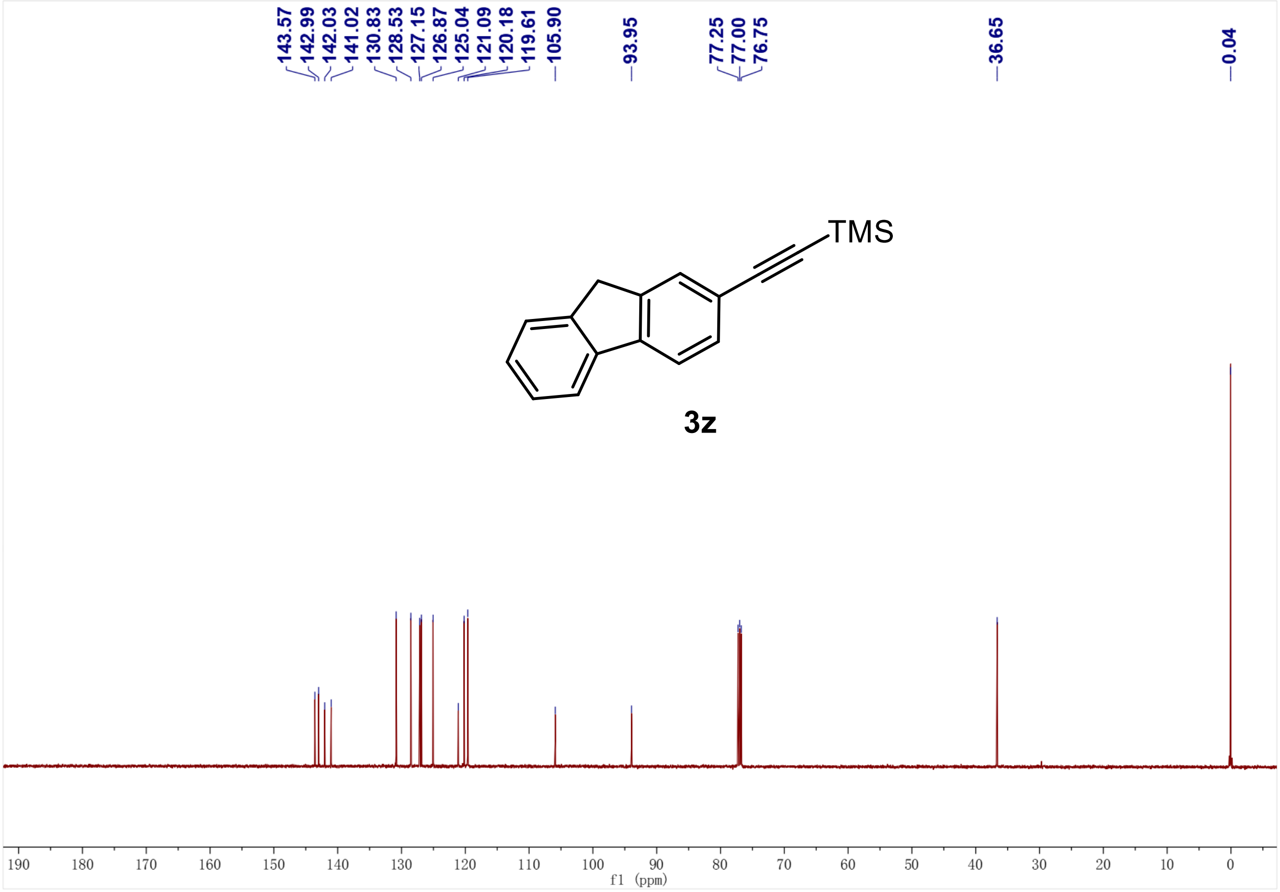

3aa; ^{1}H NMR (500 MHz, $CDCl_3$); ^{13}C NMR (126 MHz, $CDCl_3$)

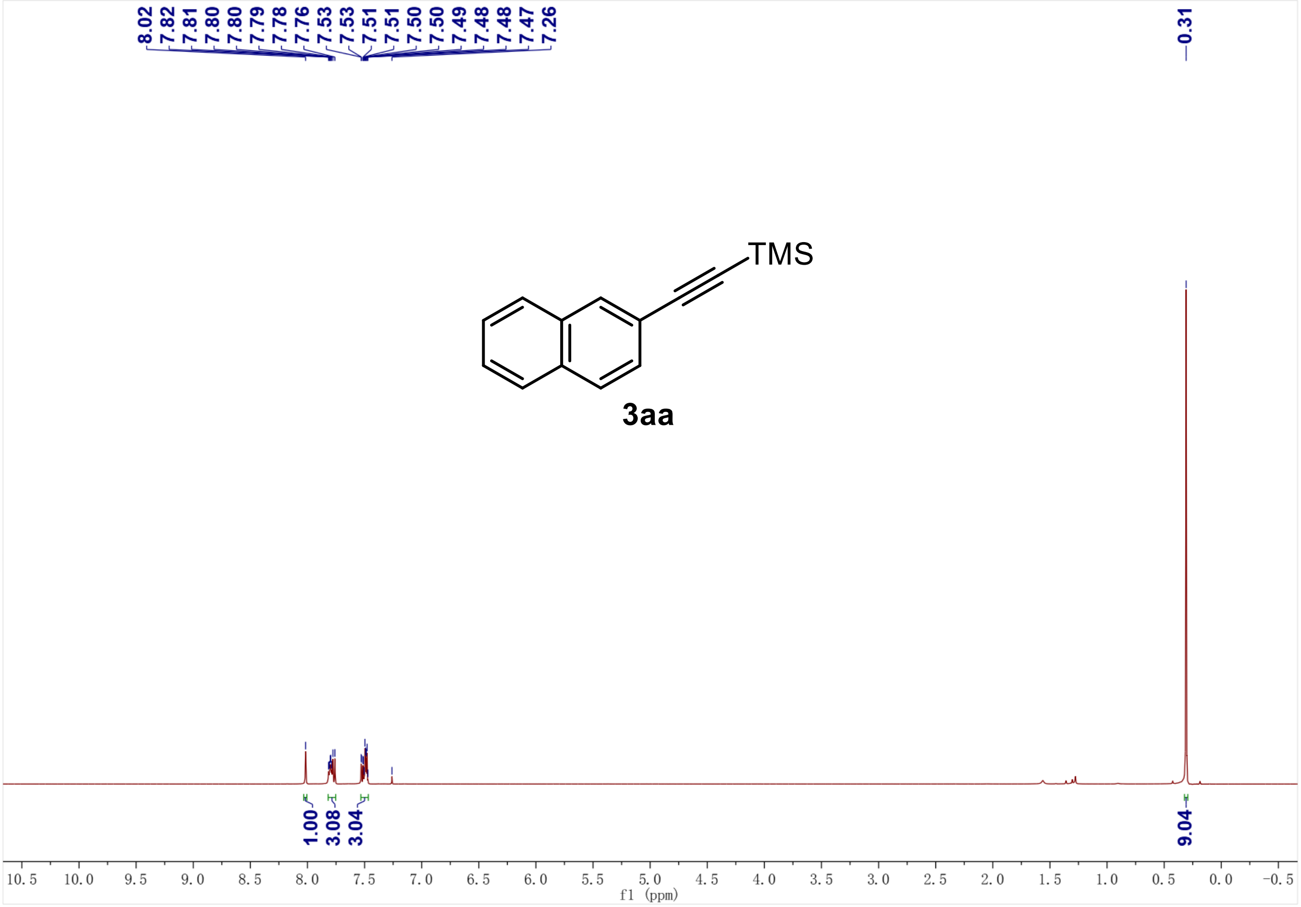

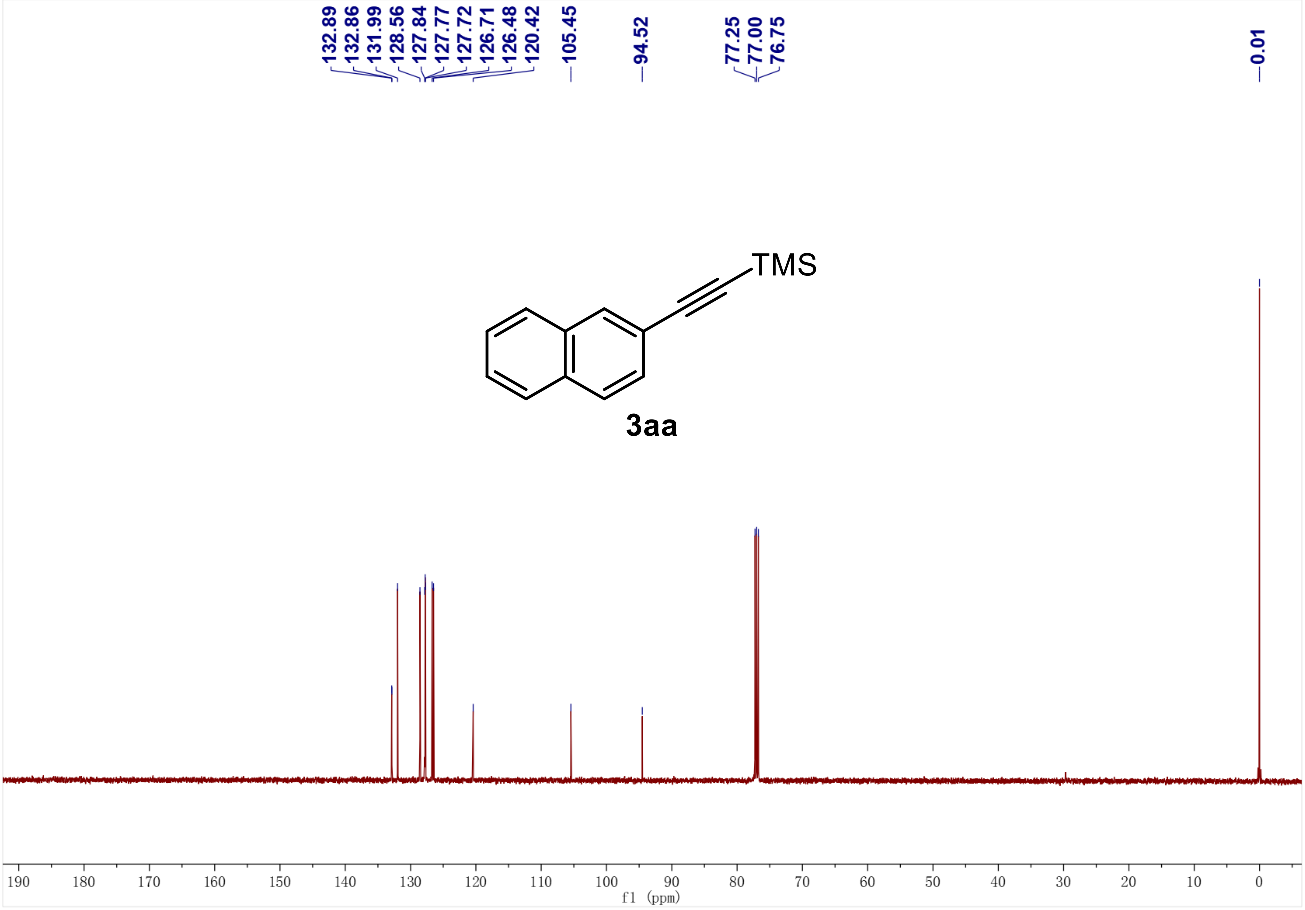

3ab; ^{1}H NMR (400 MHz, $CDCl_3$); ^{13}C NMR (126 MHz, $CDCl_3$)

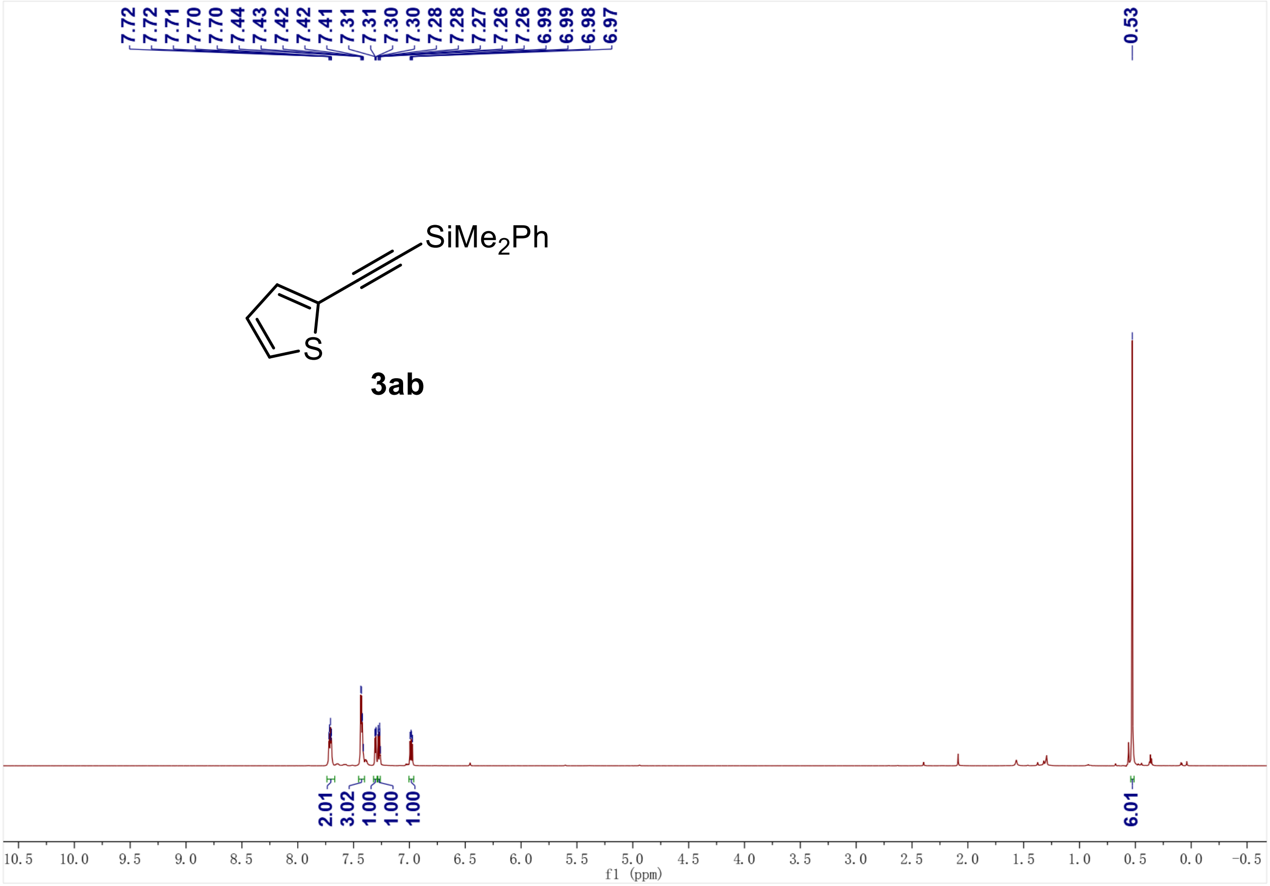

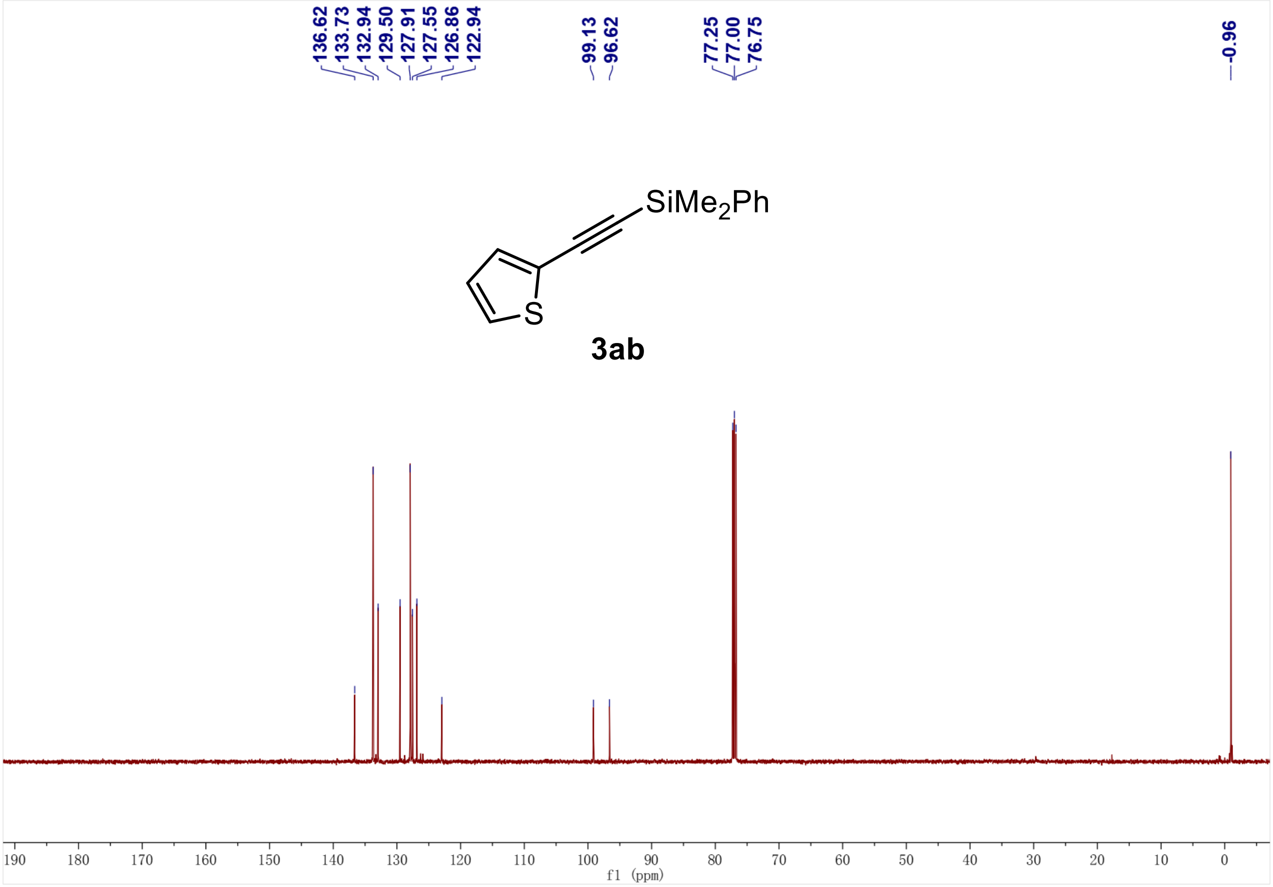

3ac; ^{1}H NMR (500 MHz, $CDCl_3$); ^{13}C NMR (126 MHz, $CDCl_3$)

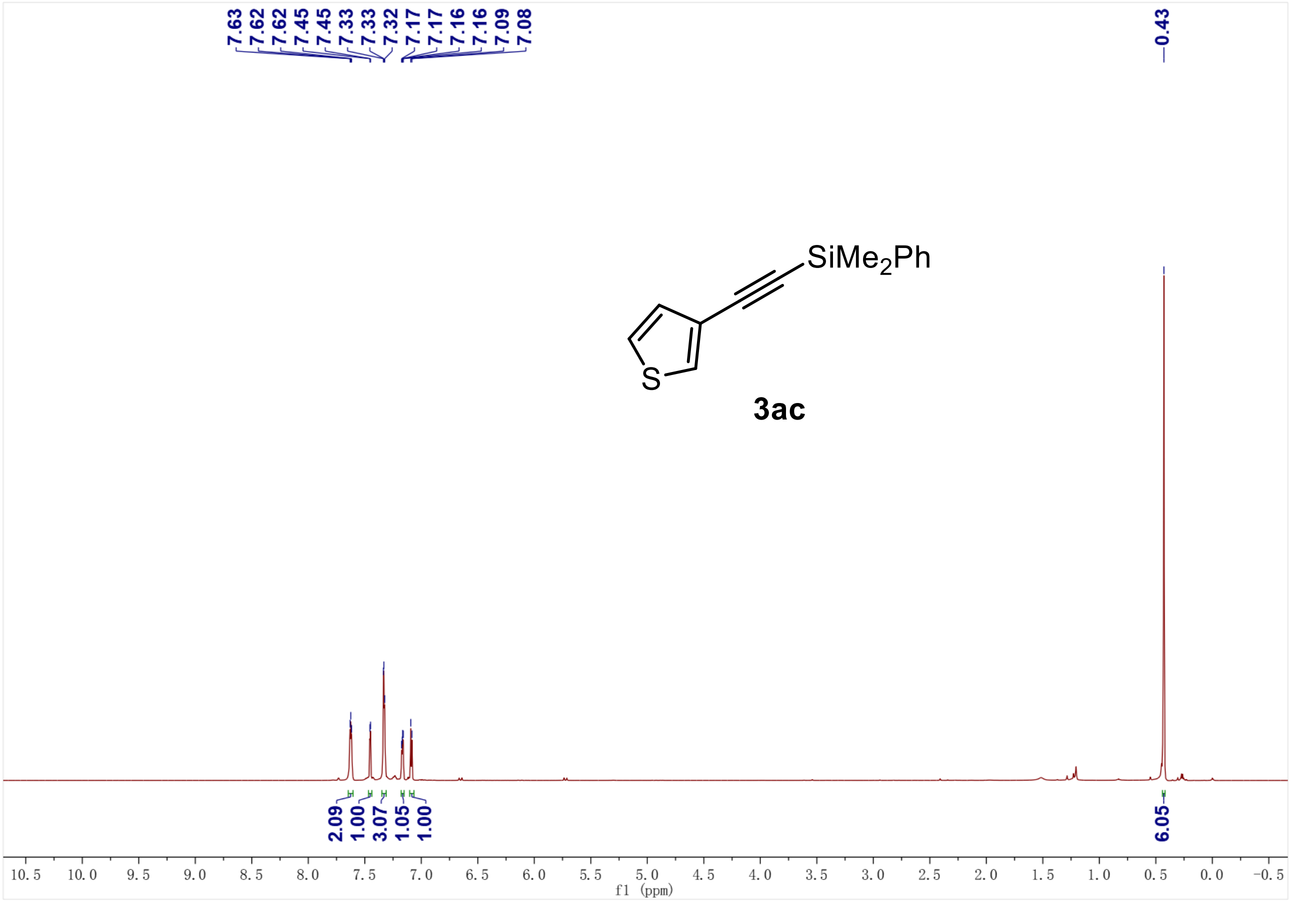

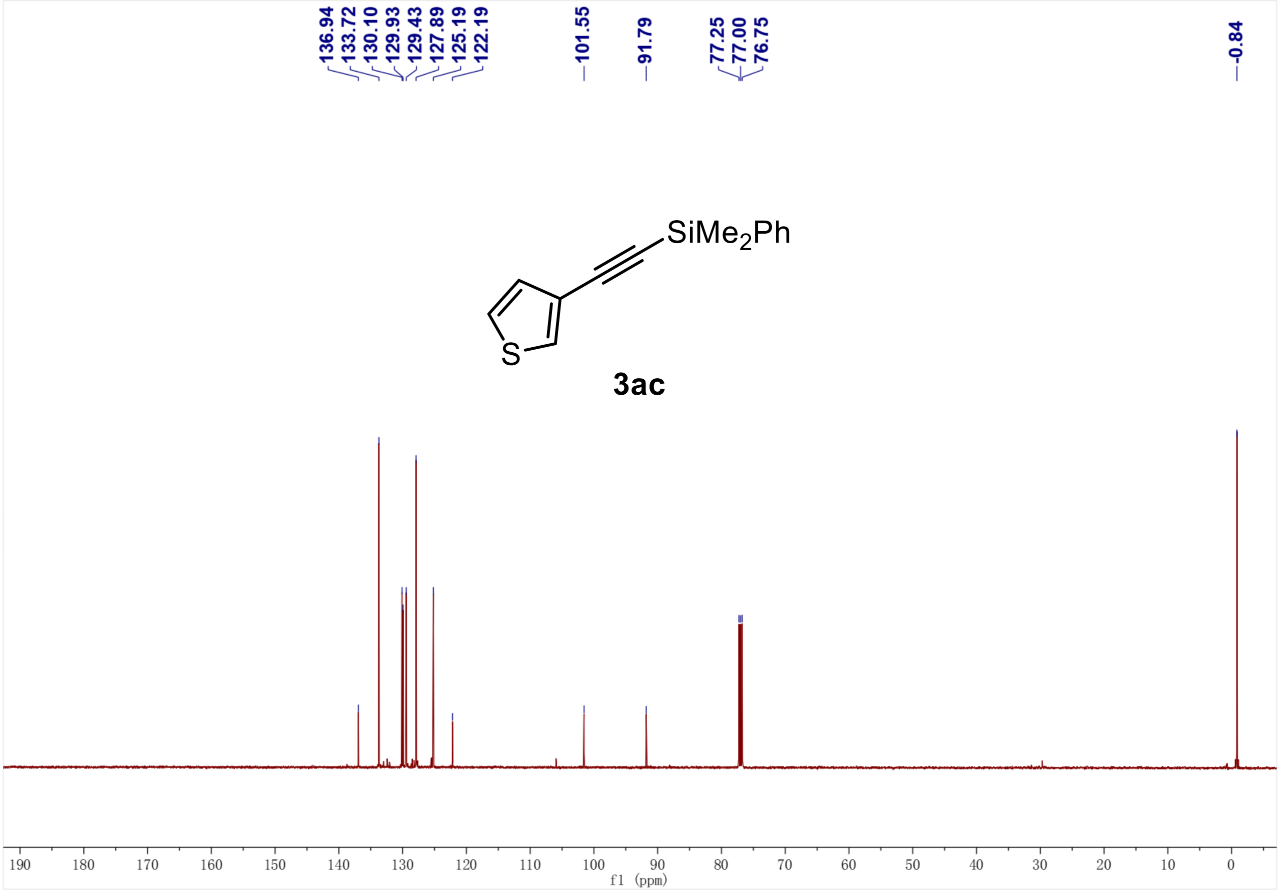

3ad; ^{1}H NMR (500 MHz, $CDCl_3$); ^{13}C NMR (126 MHz, $CDCl_3$)

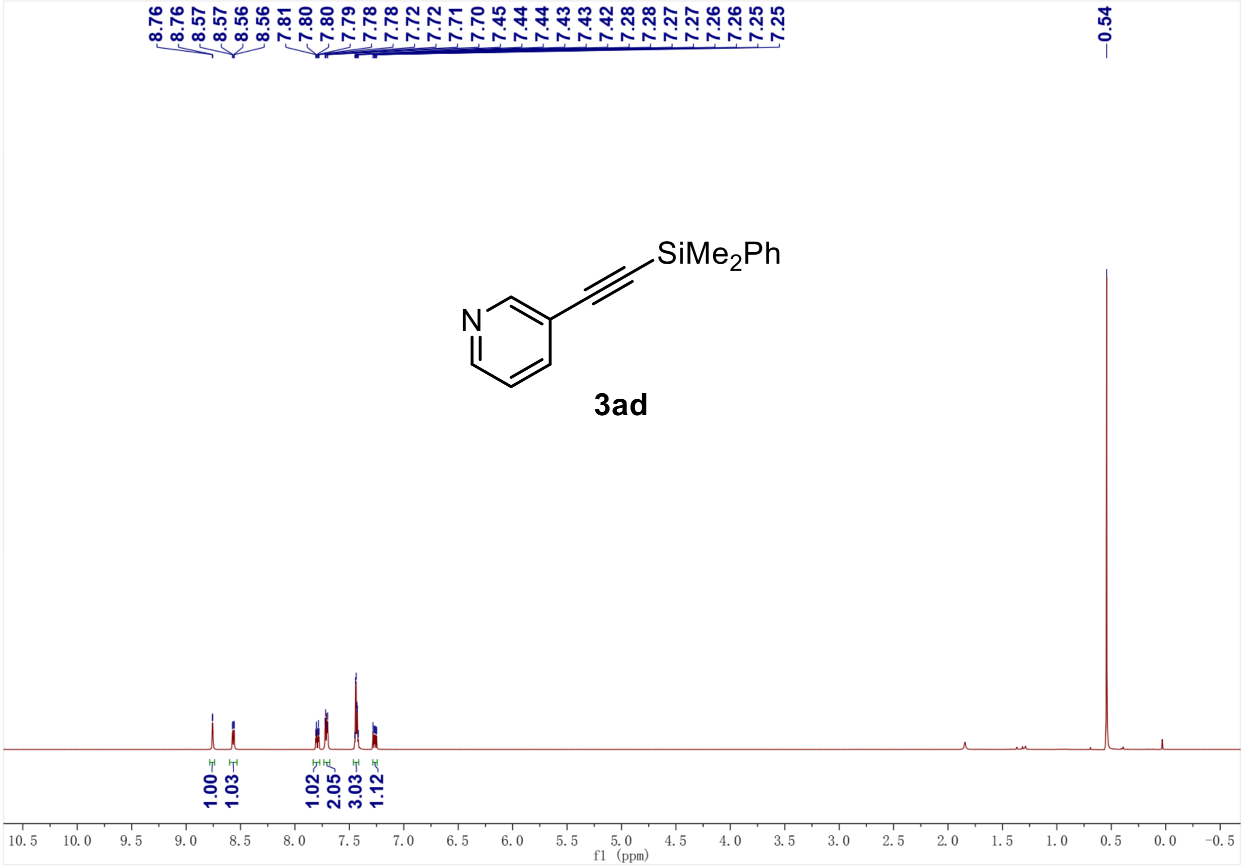

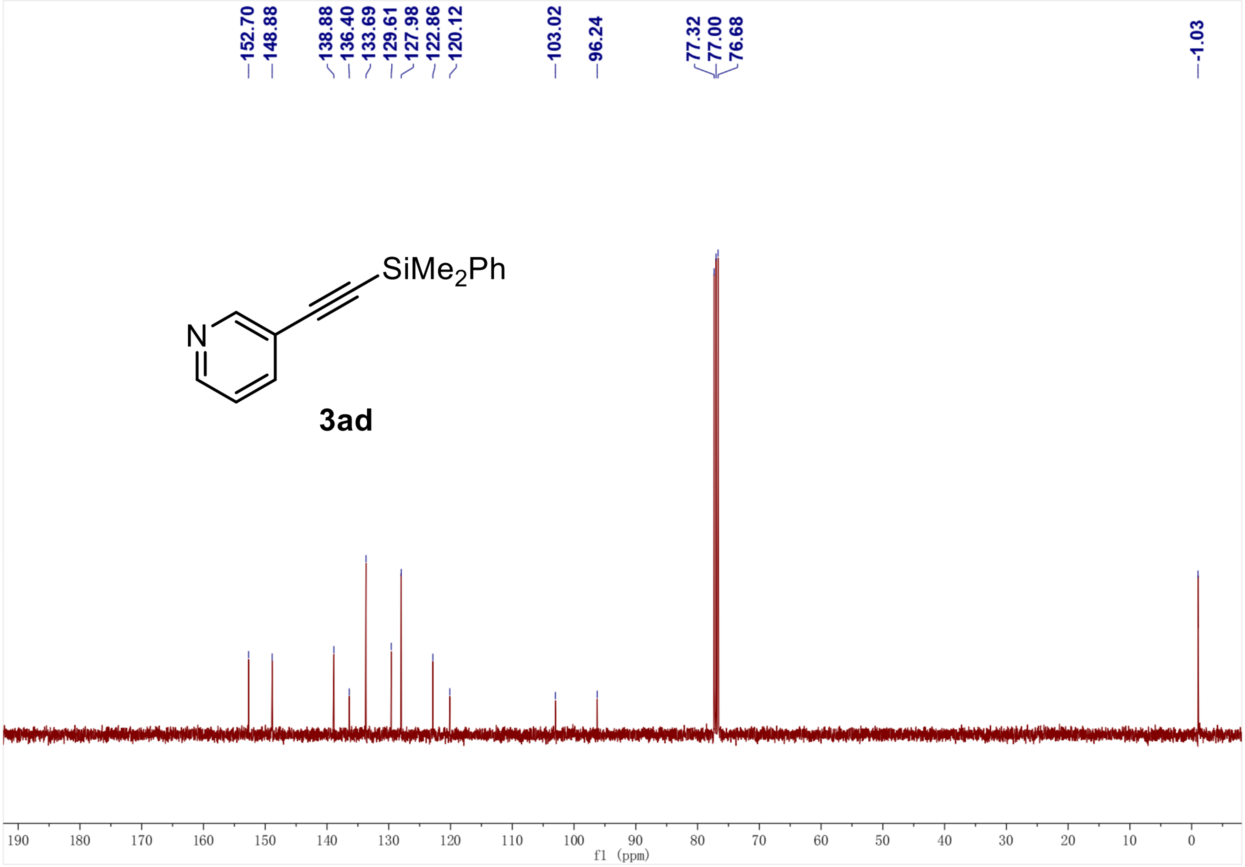

3ae; ¹H NMR (500 MHz, CDCl$_3$); ¹³C NMR (126 MHz, CDCl$_3$)

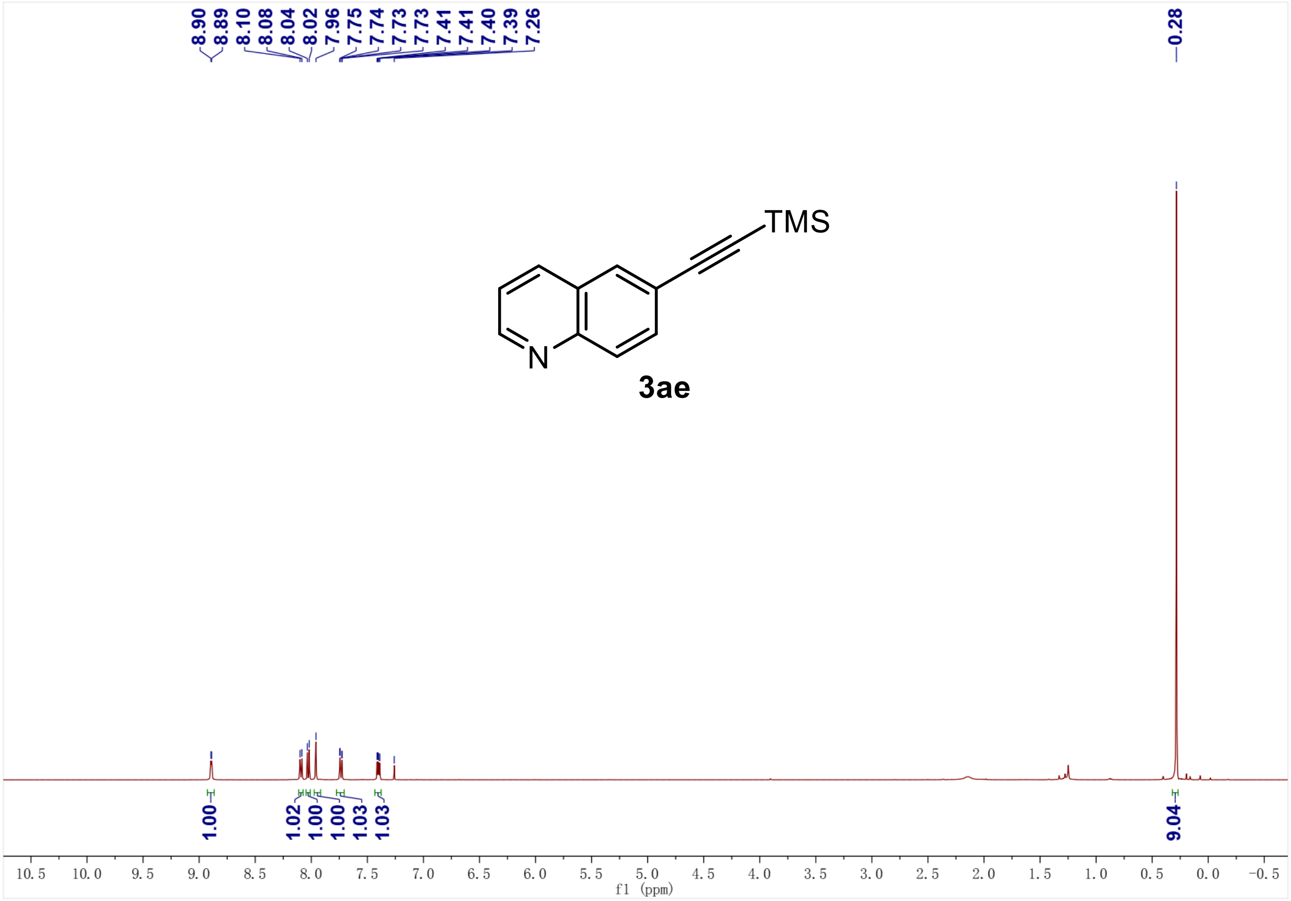

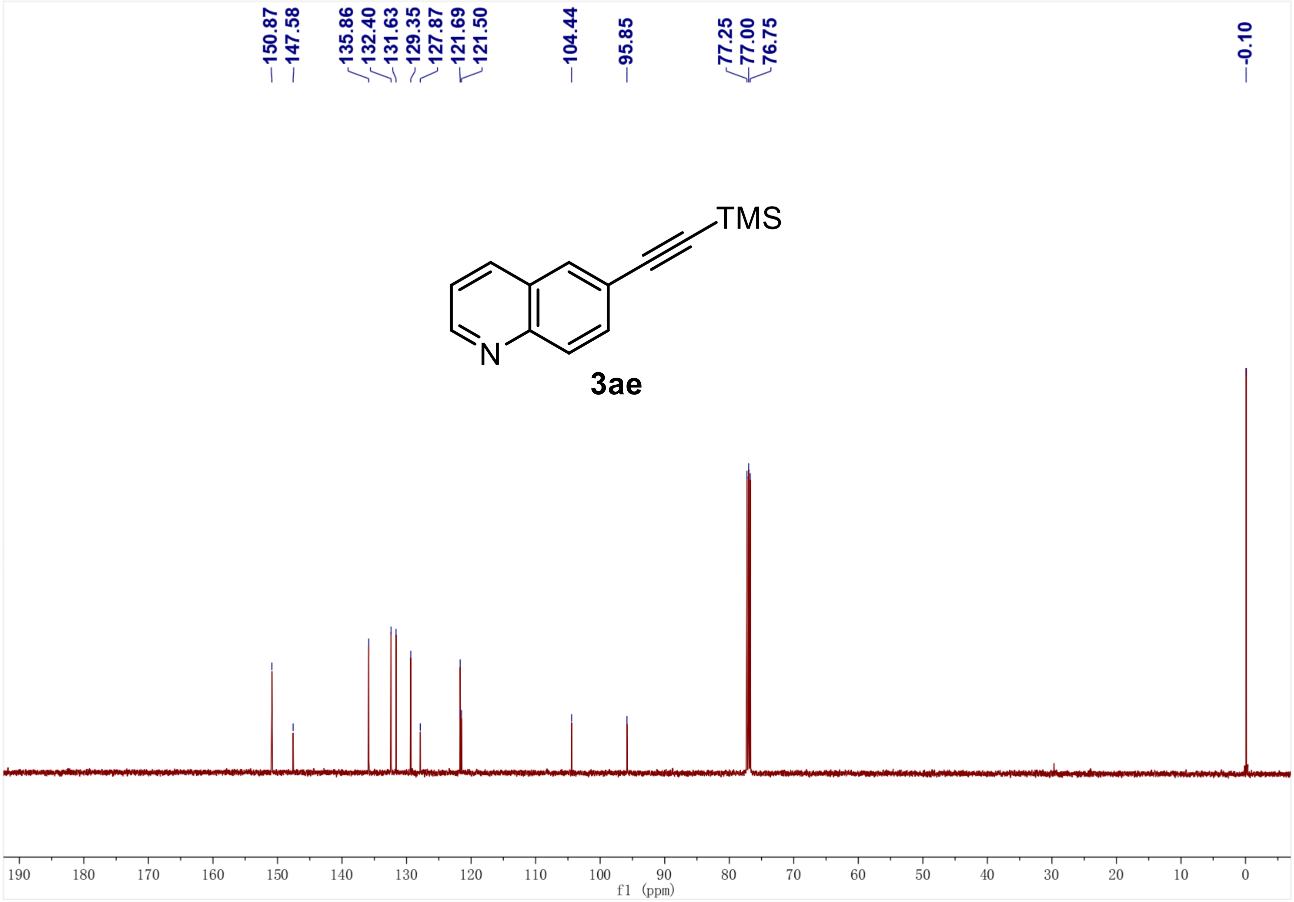

3af; ^{1}H NMR (500 MHz, $CDCl_3$); ^{13}C NMR (126 MHz, $CDCl_3$)

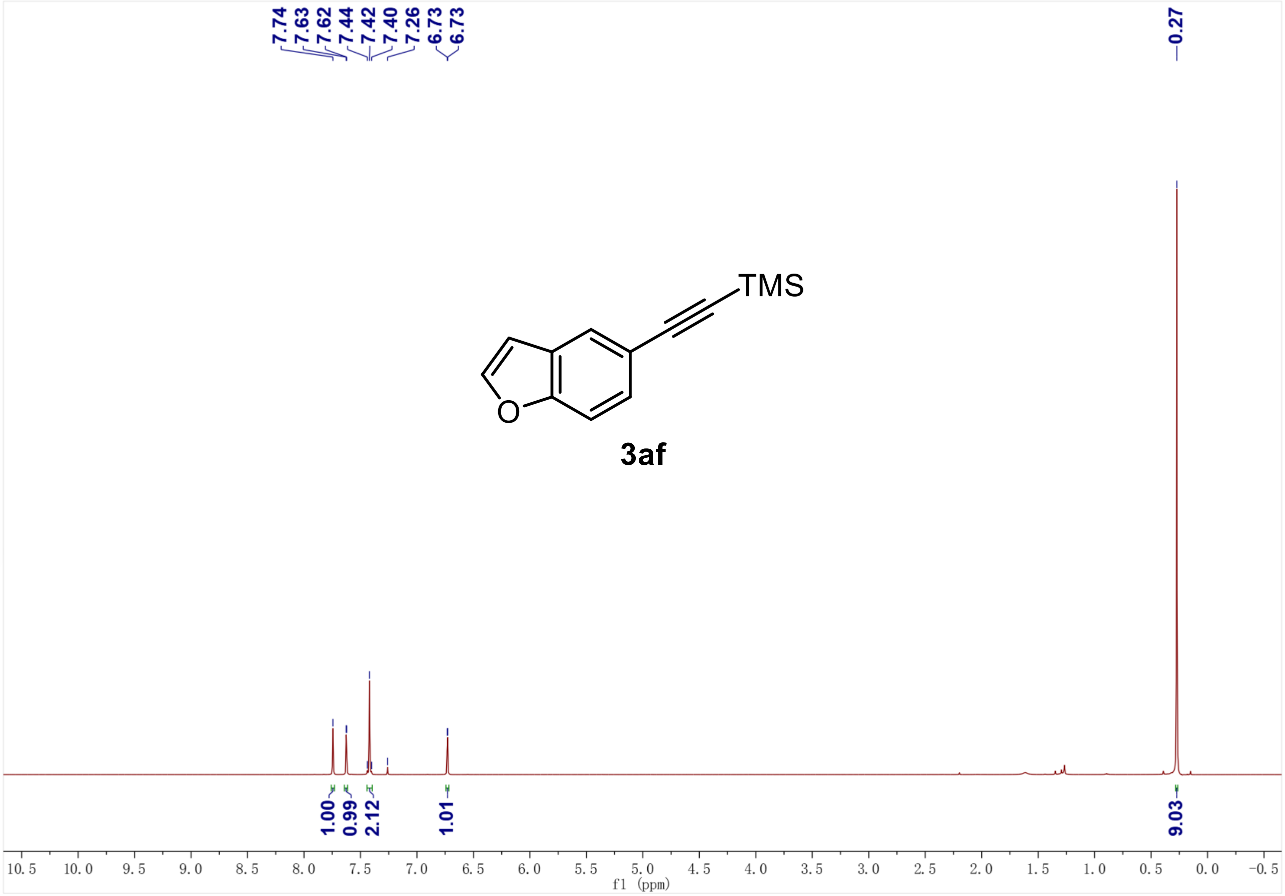

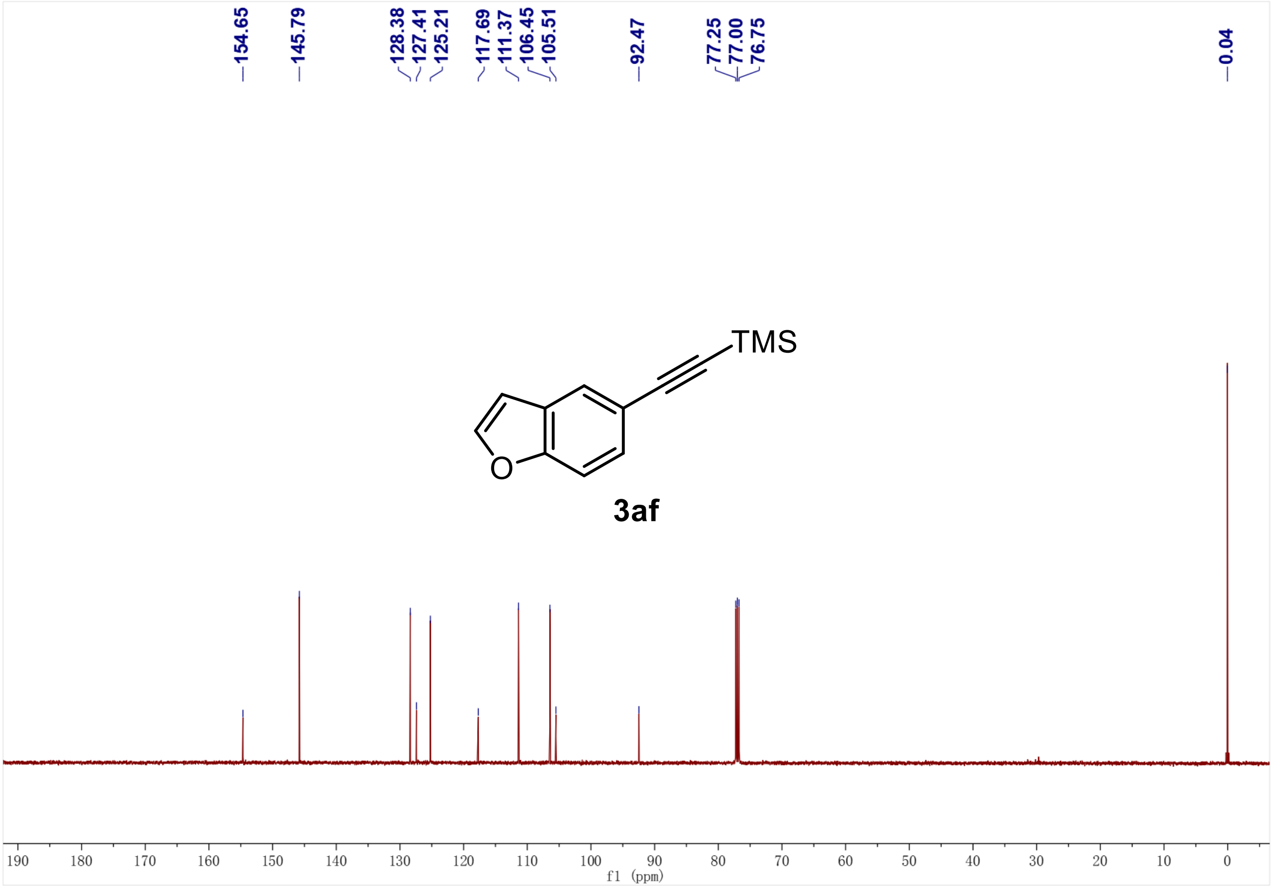

3ag; ¹H NMR (500 MHz, CDCl$_3$); ^{13}C NMR (126 MHz, CDCl$_3$)

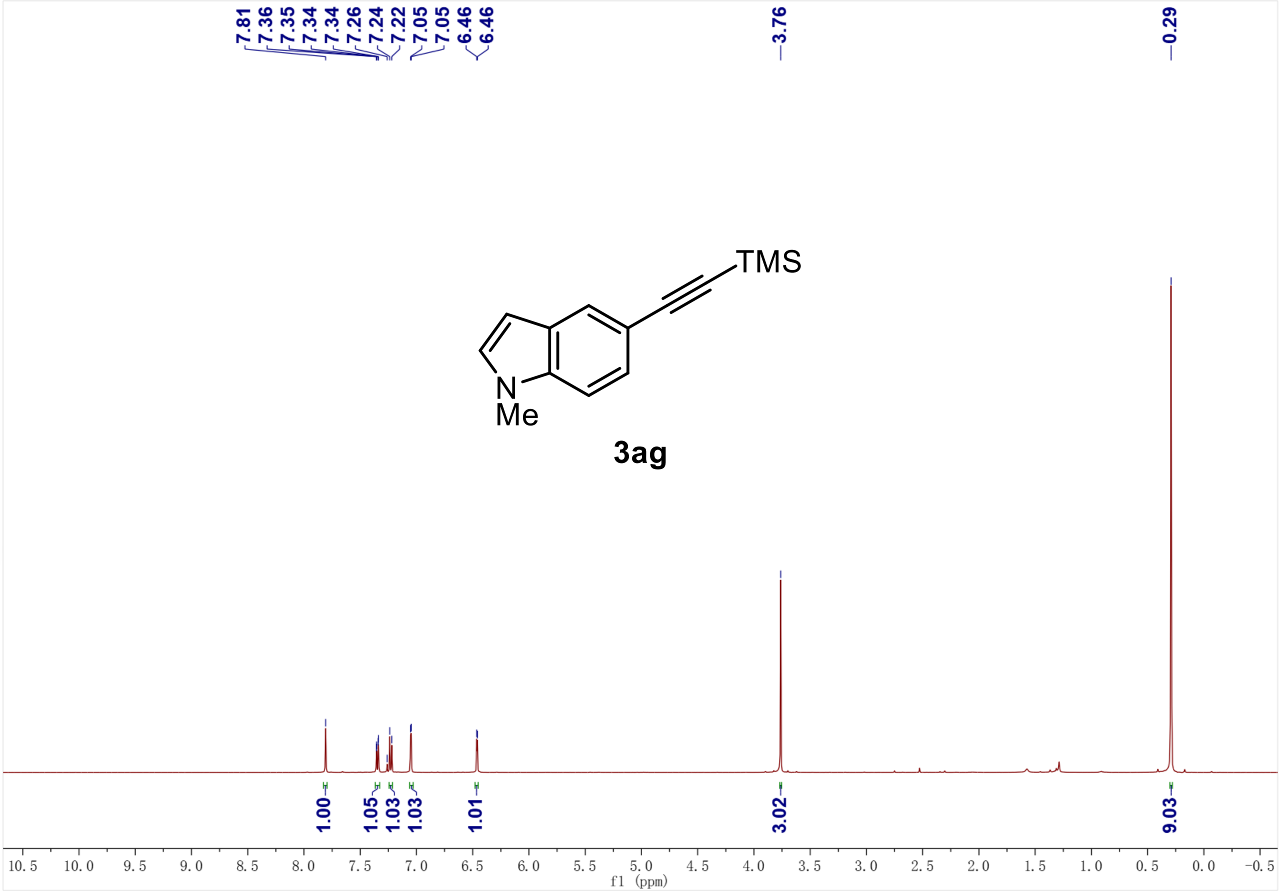

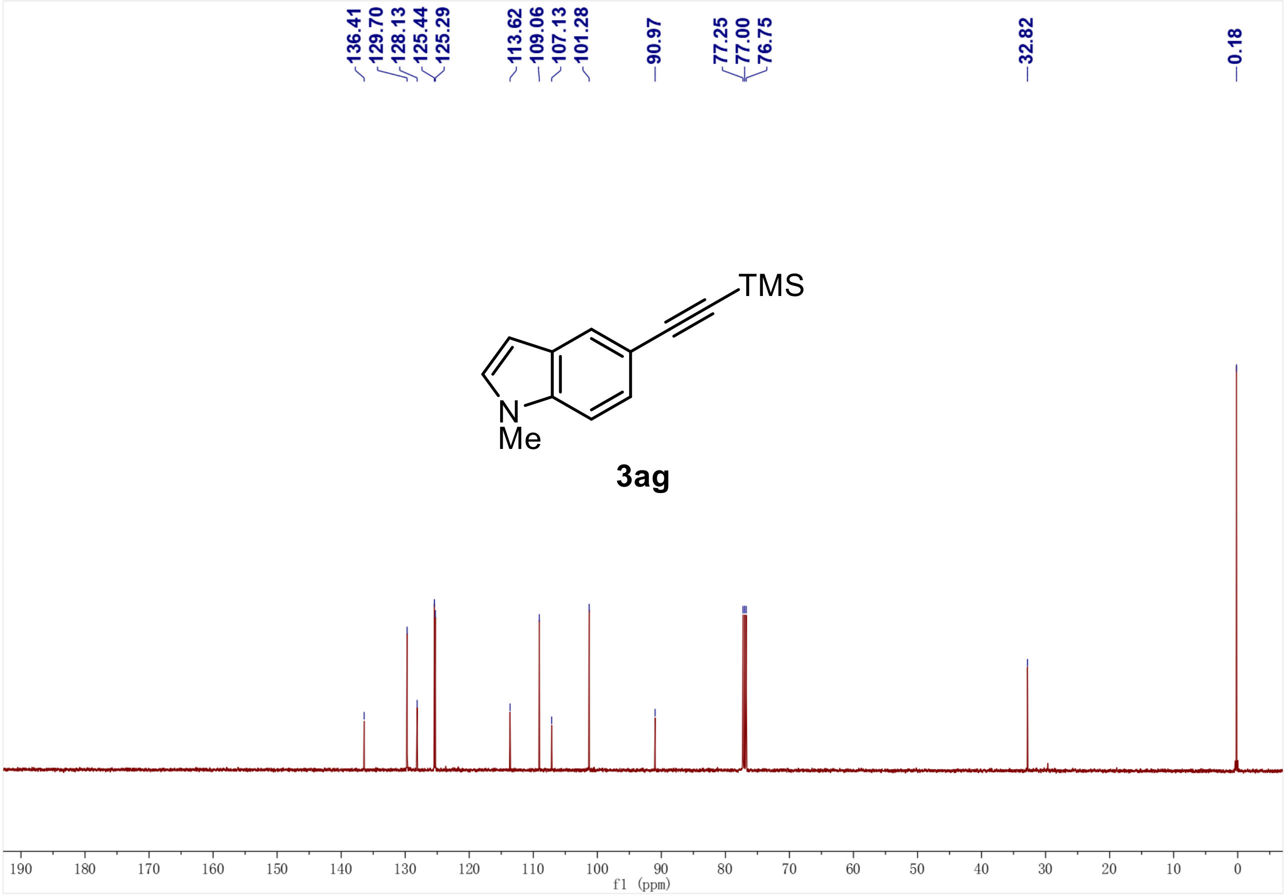

3ah; ^{1}H NMR (500 MHz, $CDCl_3$); ^{13}C NMR (126 MHz, $CDCl_3$)

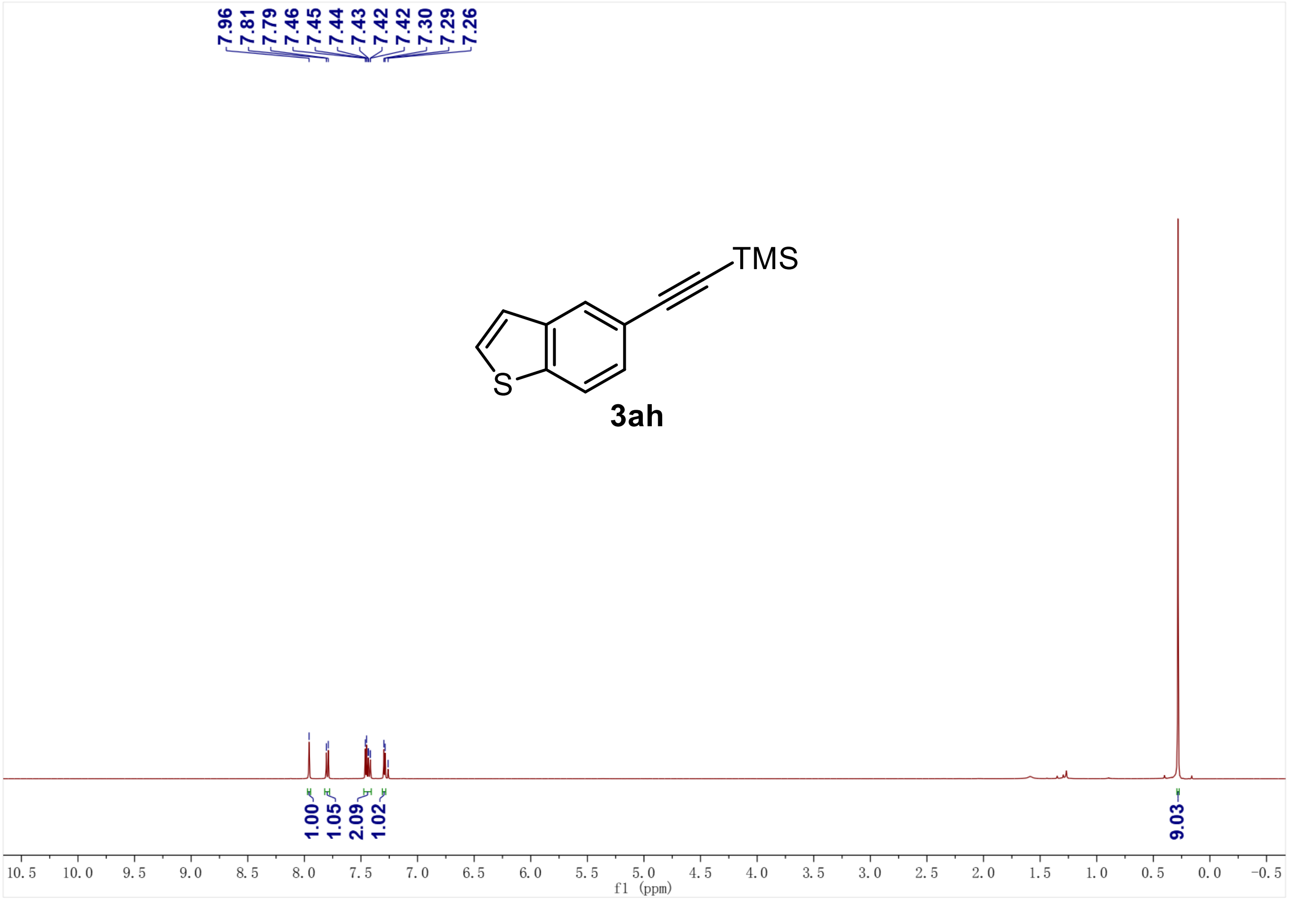

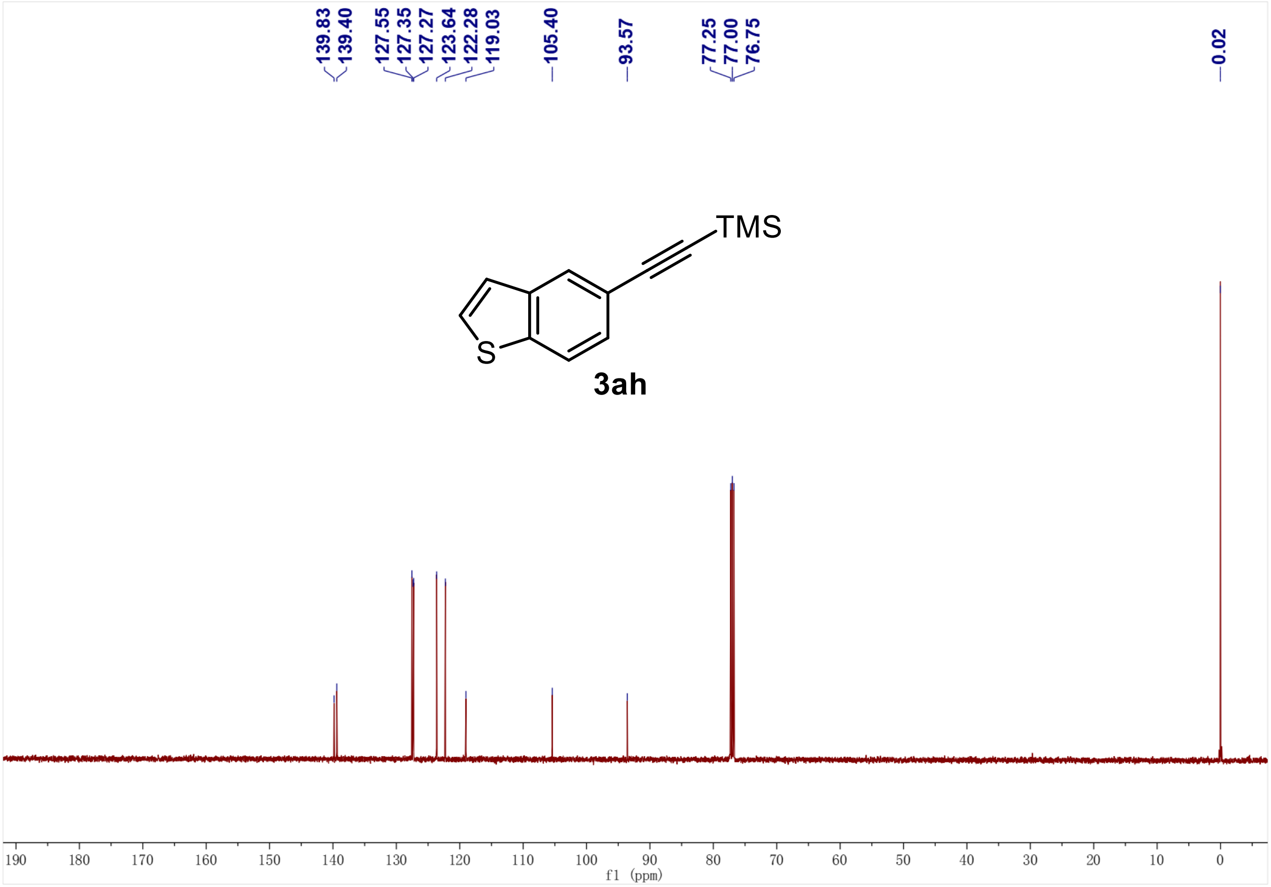

3ai; ^{1}H NMR (500 MHz, $CDCl_3$); ^{13}C NMR (126 MHz, $CDCl_3$)

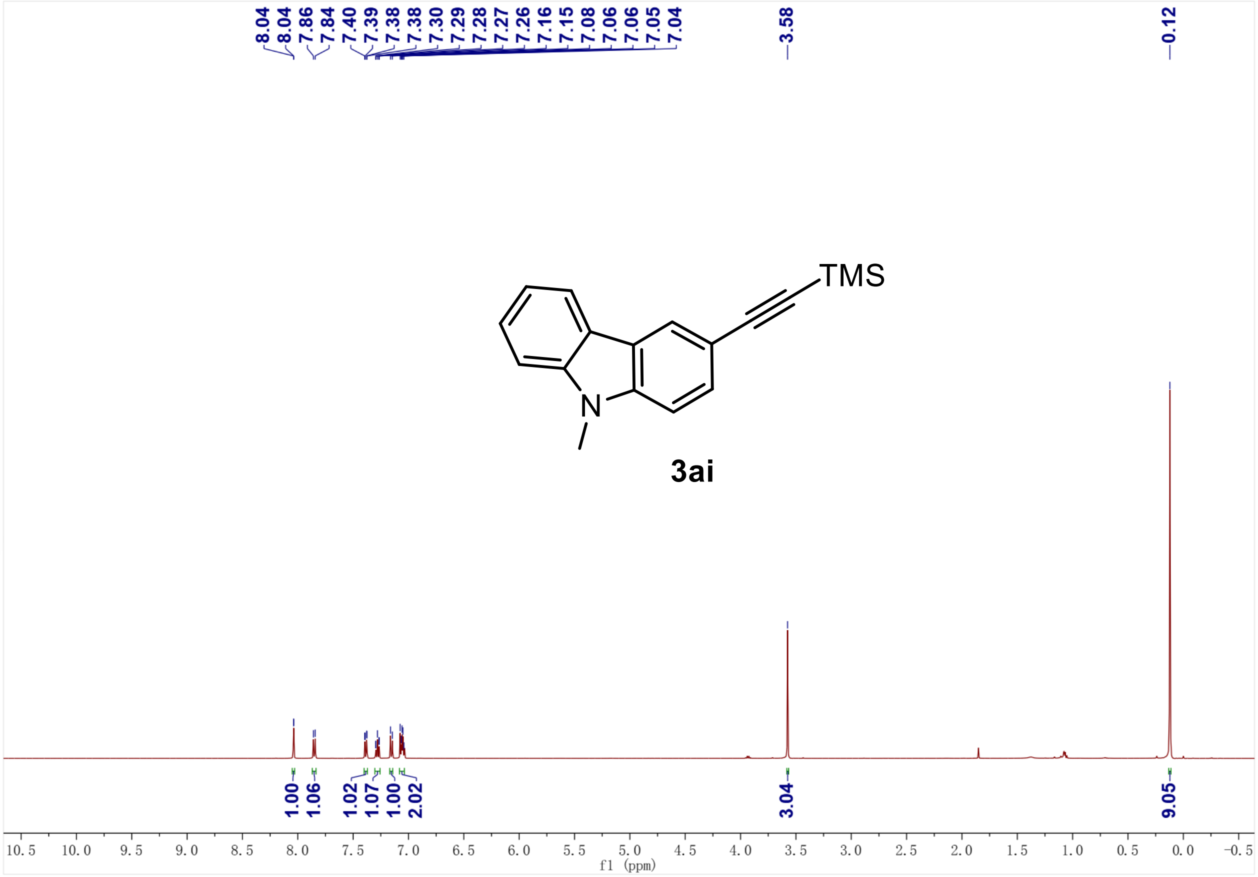

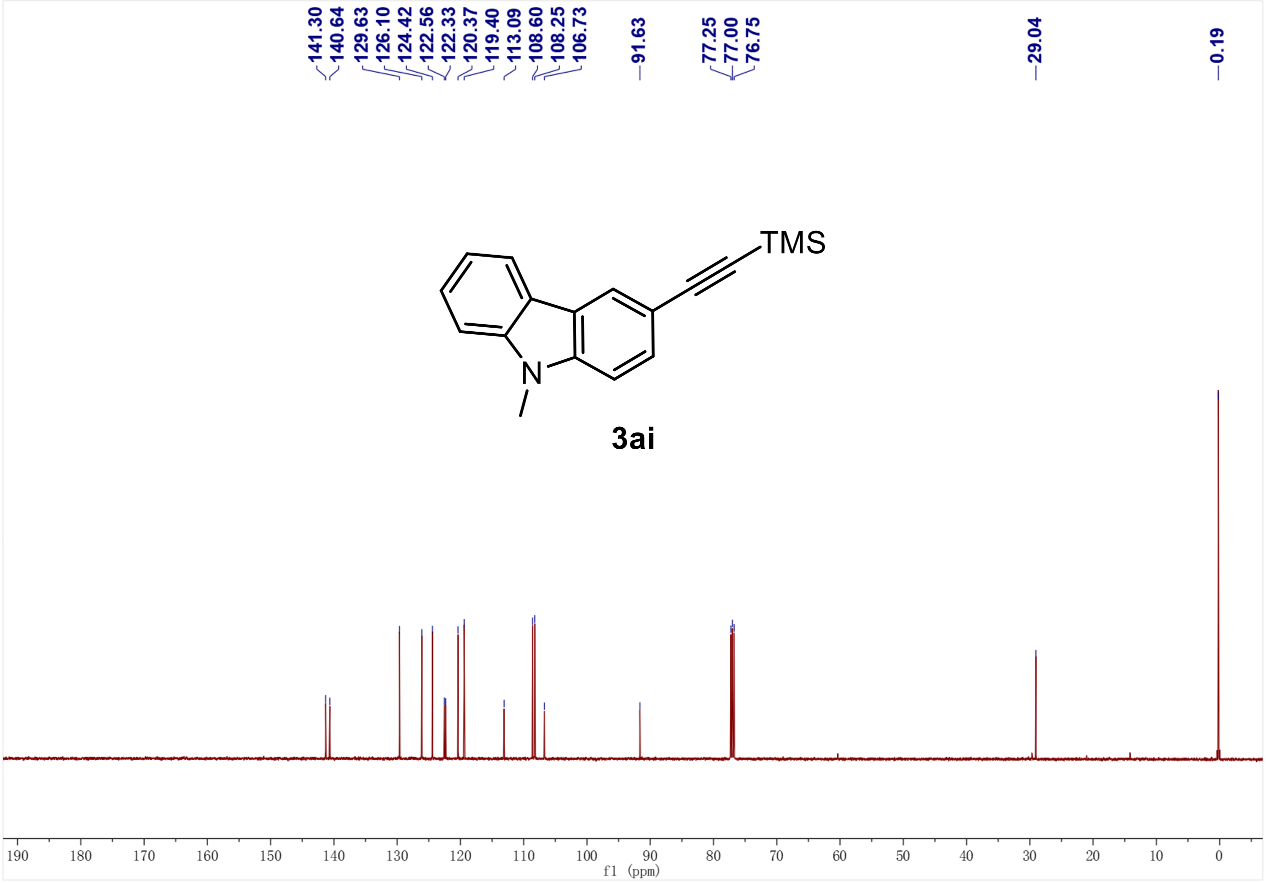

3aj; ¹H NMR (500 MHz, CDCl$_3$); ¹³C NMR (126 MHz, CDCl$_3$)

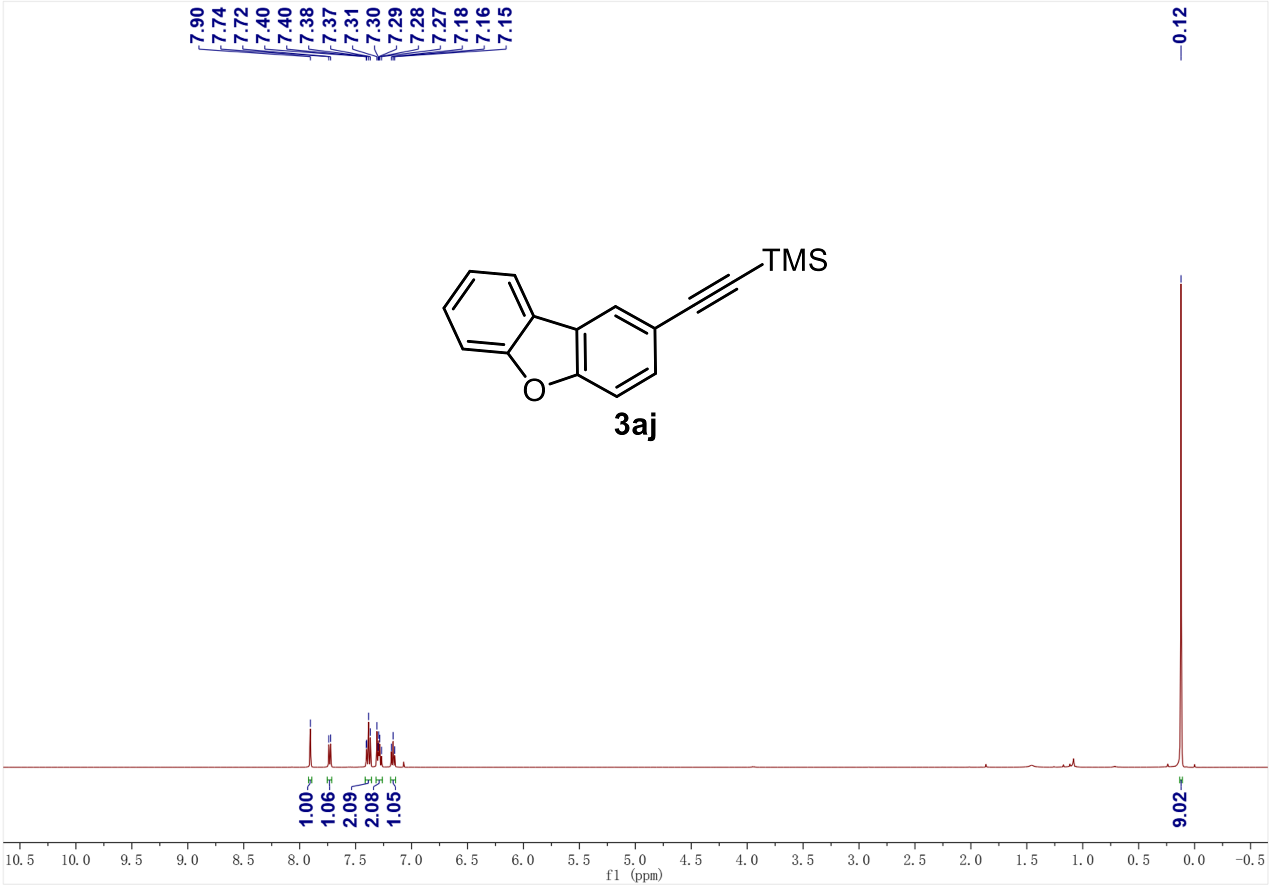

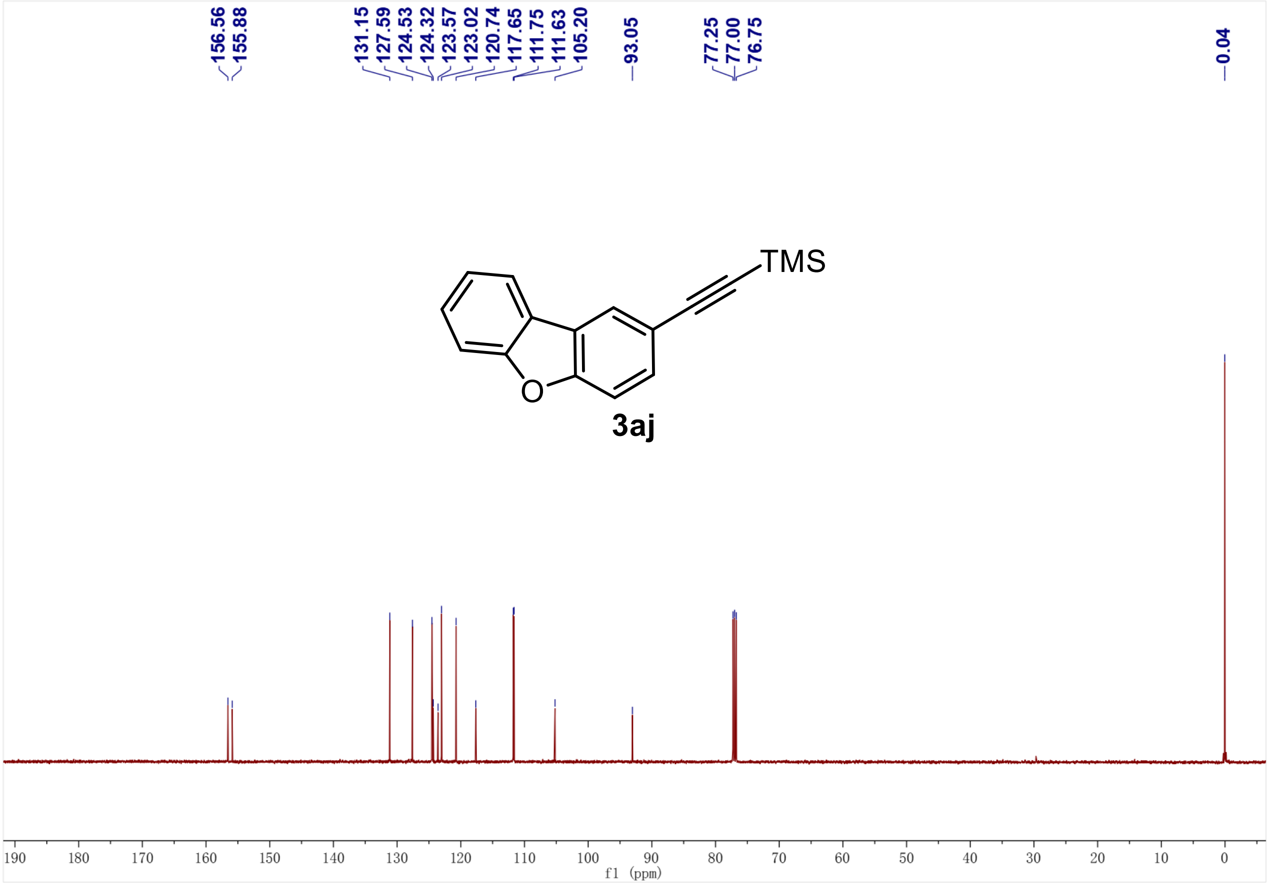

3ak; ^{1}H NMR (500 MHz, $CDCl_3$); ^{13}C NMR (126 MHz, $CDCl_3$)

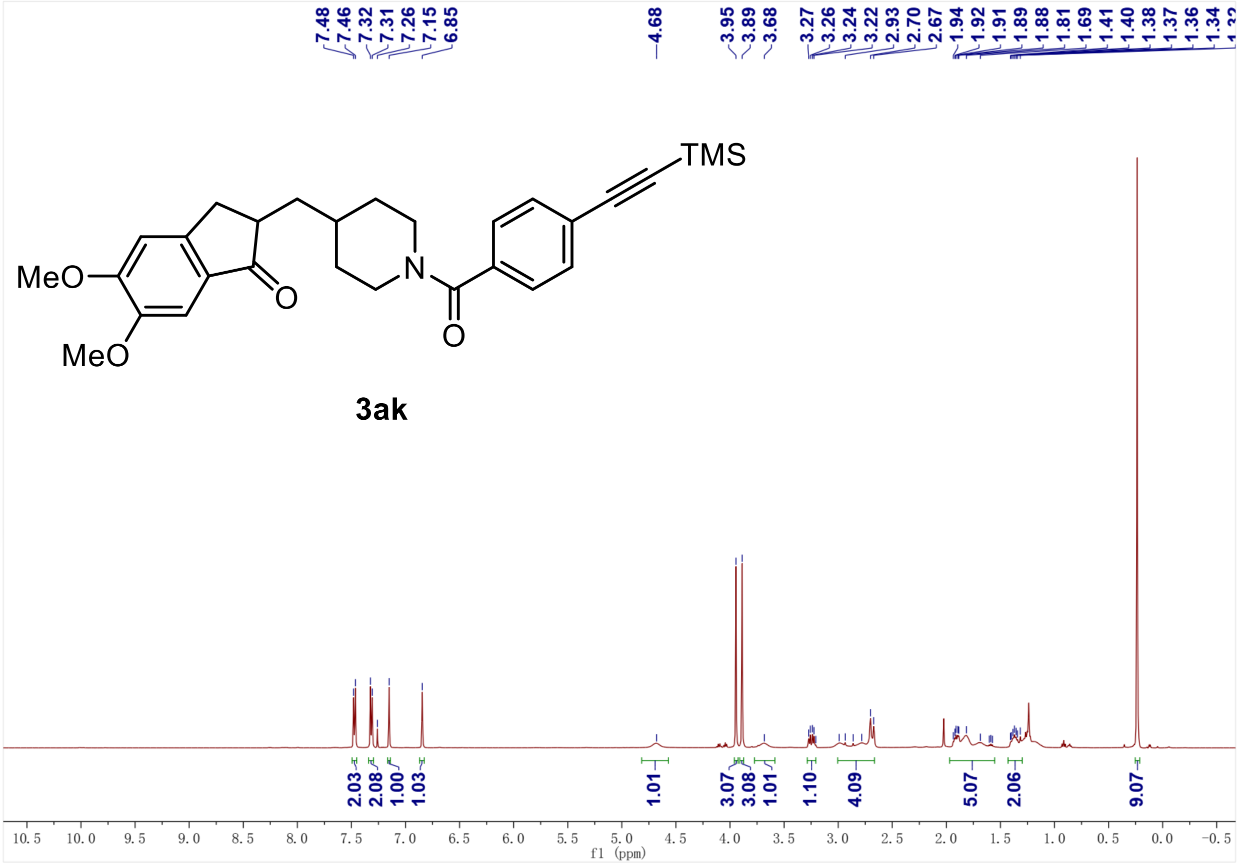

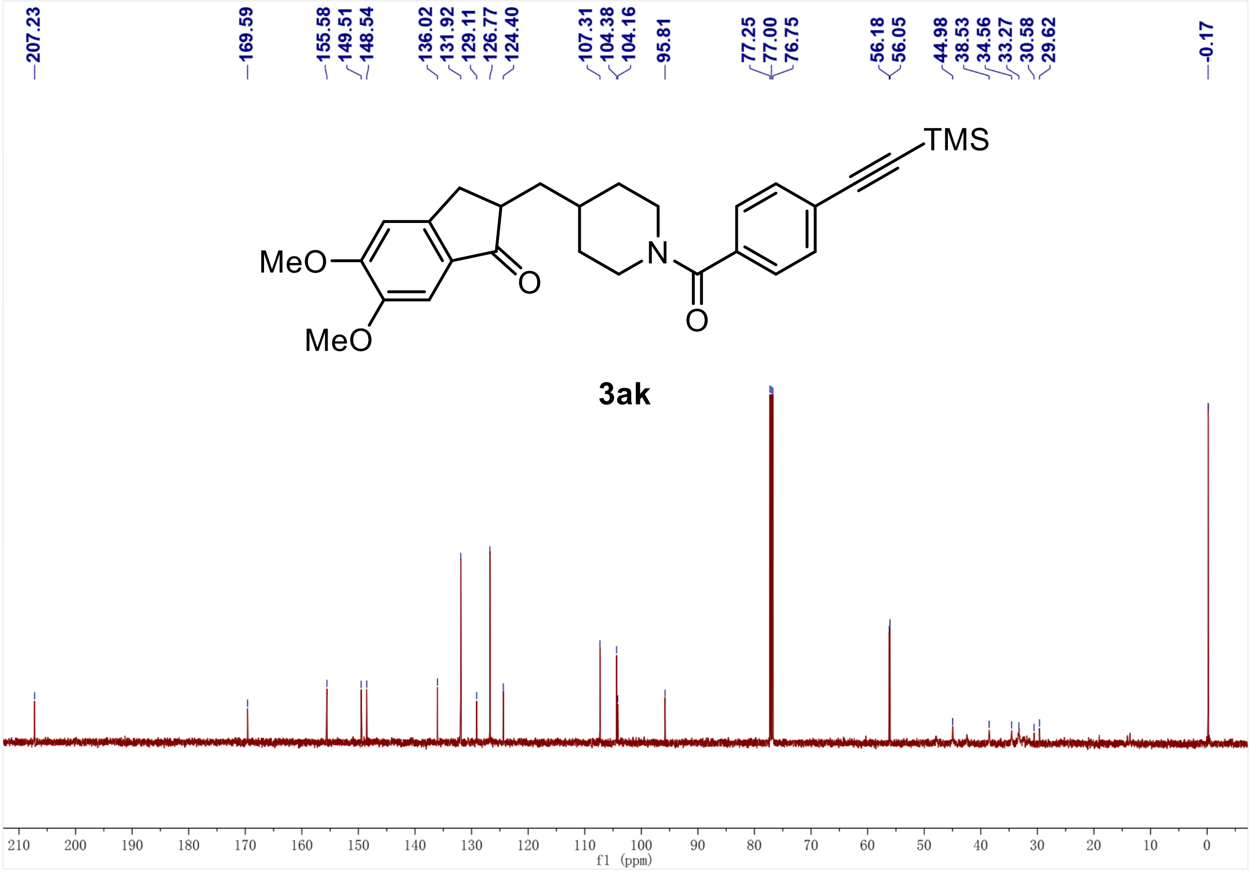

3al; ^{1}H NMR (500 MHz, $CDCl_3$); ^{13}C NMR (126 MHz, $CDCl_3$)

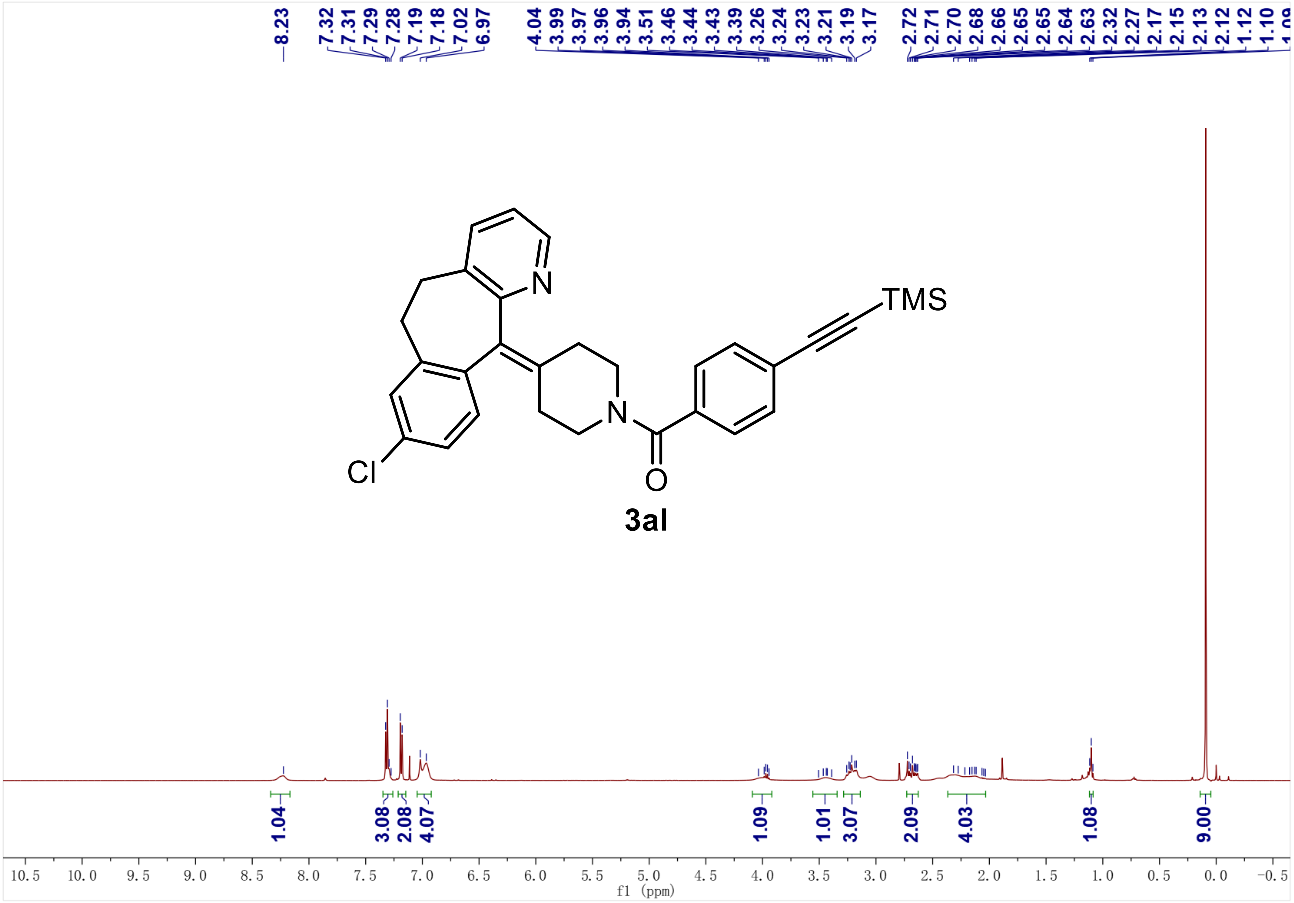

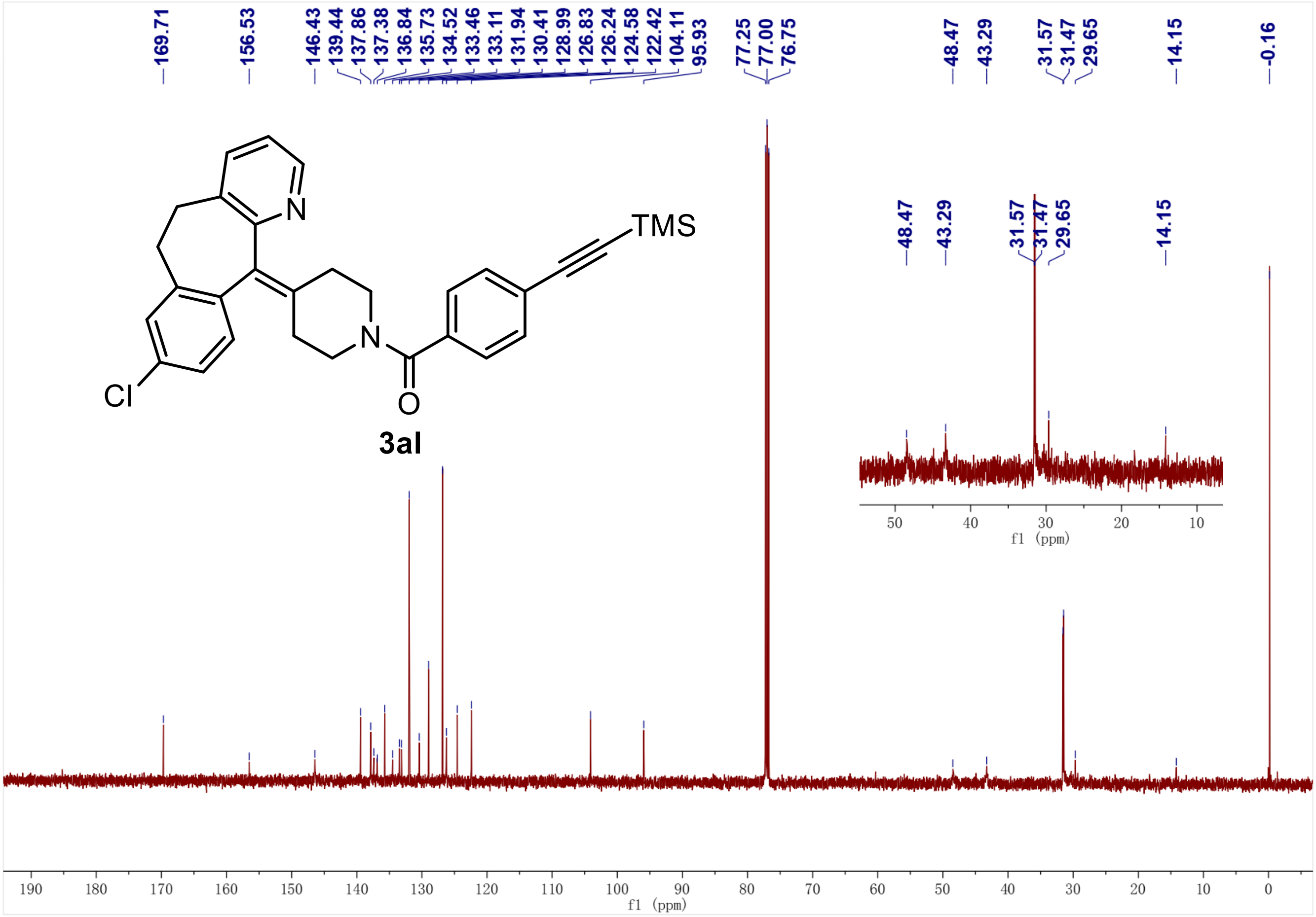

3am; ^{1}H NMR (500 MHz, $CDCl_3$); ^{13}C NMR (126 MHz, $CDCl_3$)

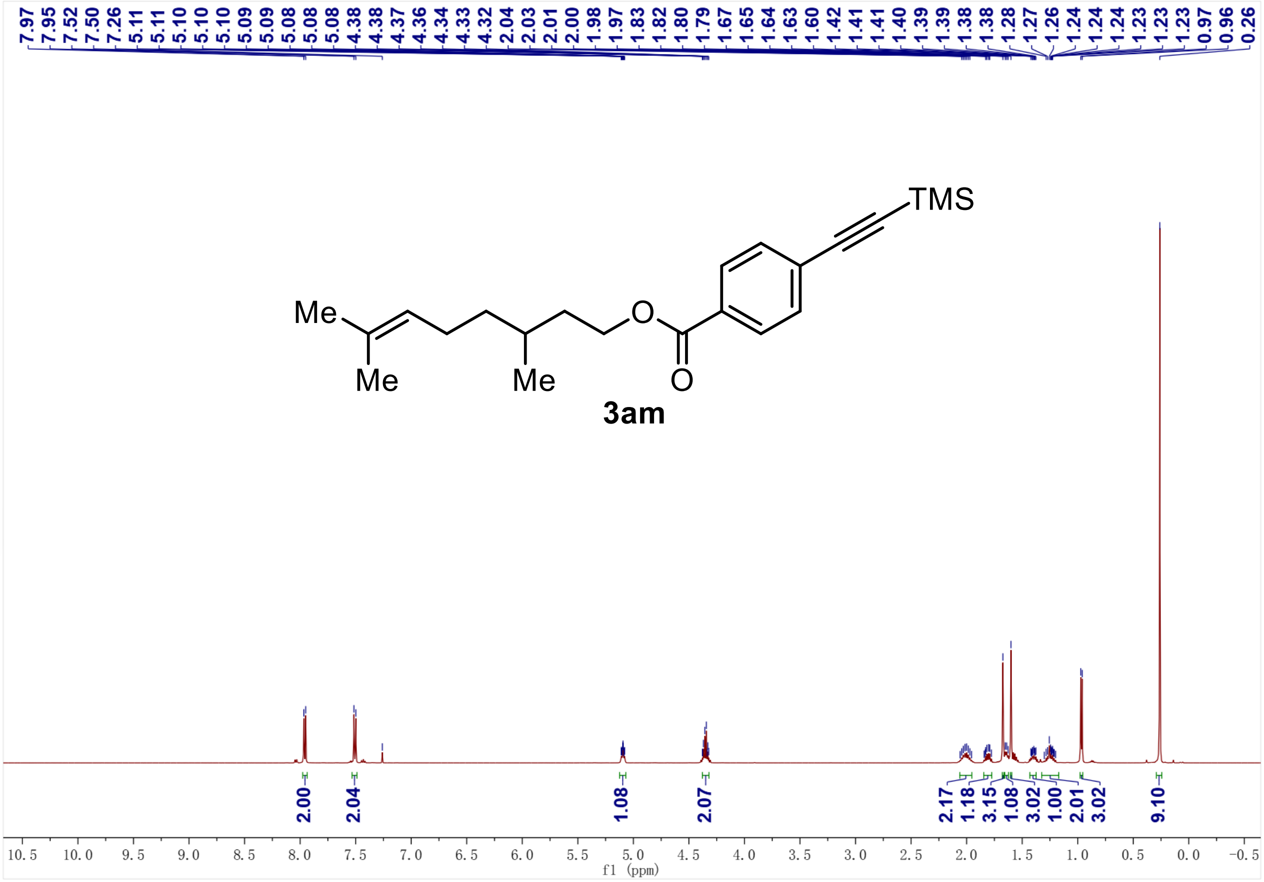

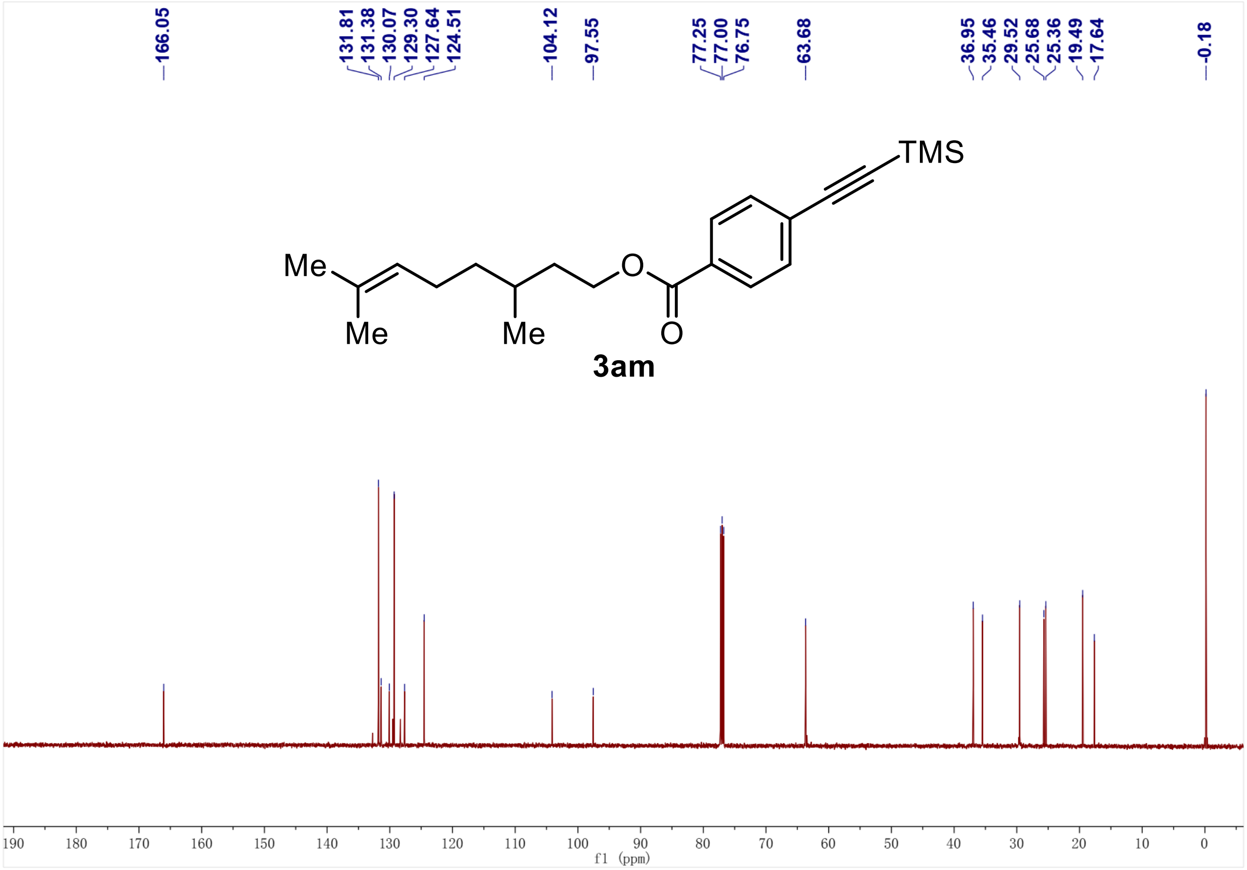

3an; ¹H NMR (500 MHz, $CDCl_3$); ¹³C NMR (126 MHz, $CDCl_3$)

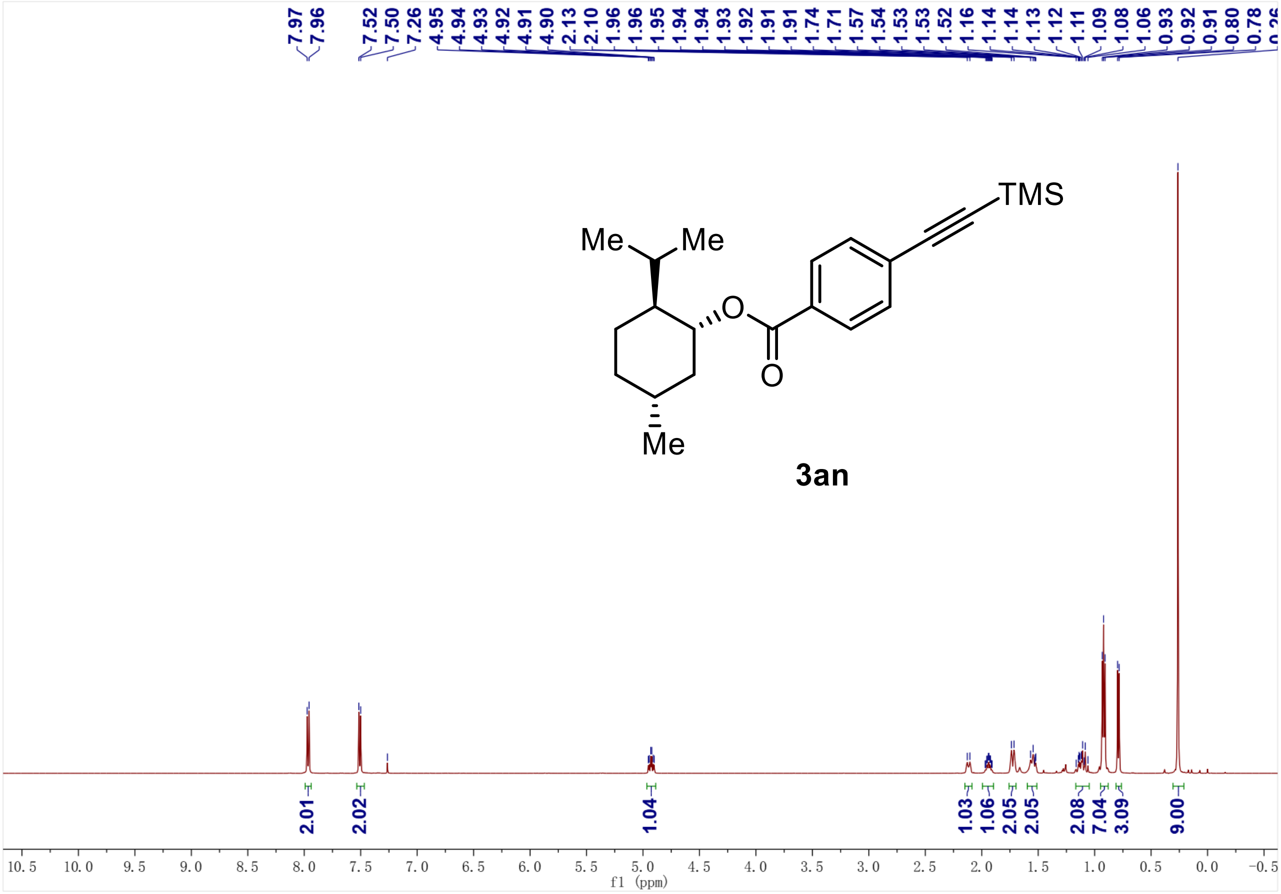

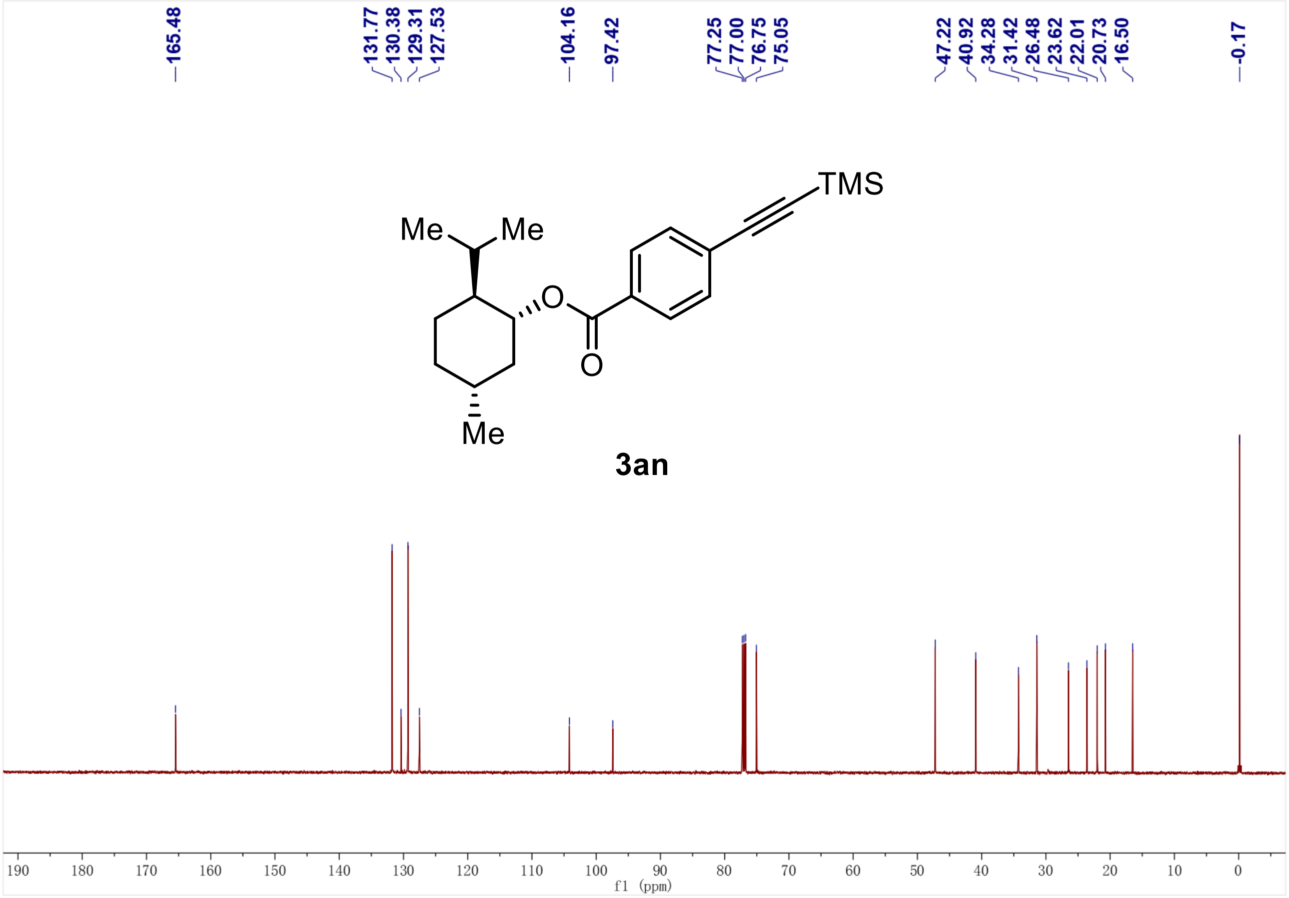

3ao; ^{1}H NMR (500 MHz, $CDCl_3$); ^{13}C NMR (126 MHz, $CDCl_3$)

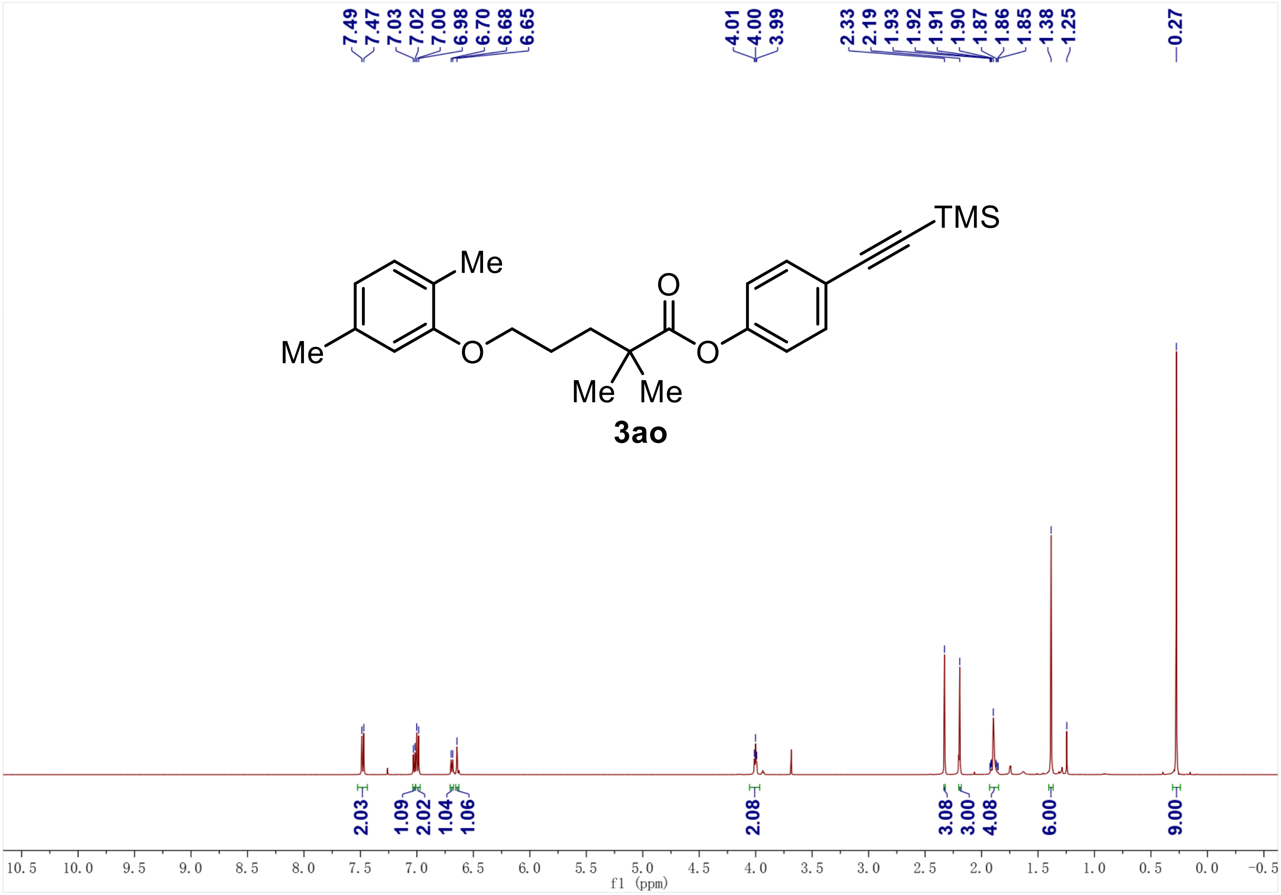

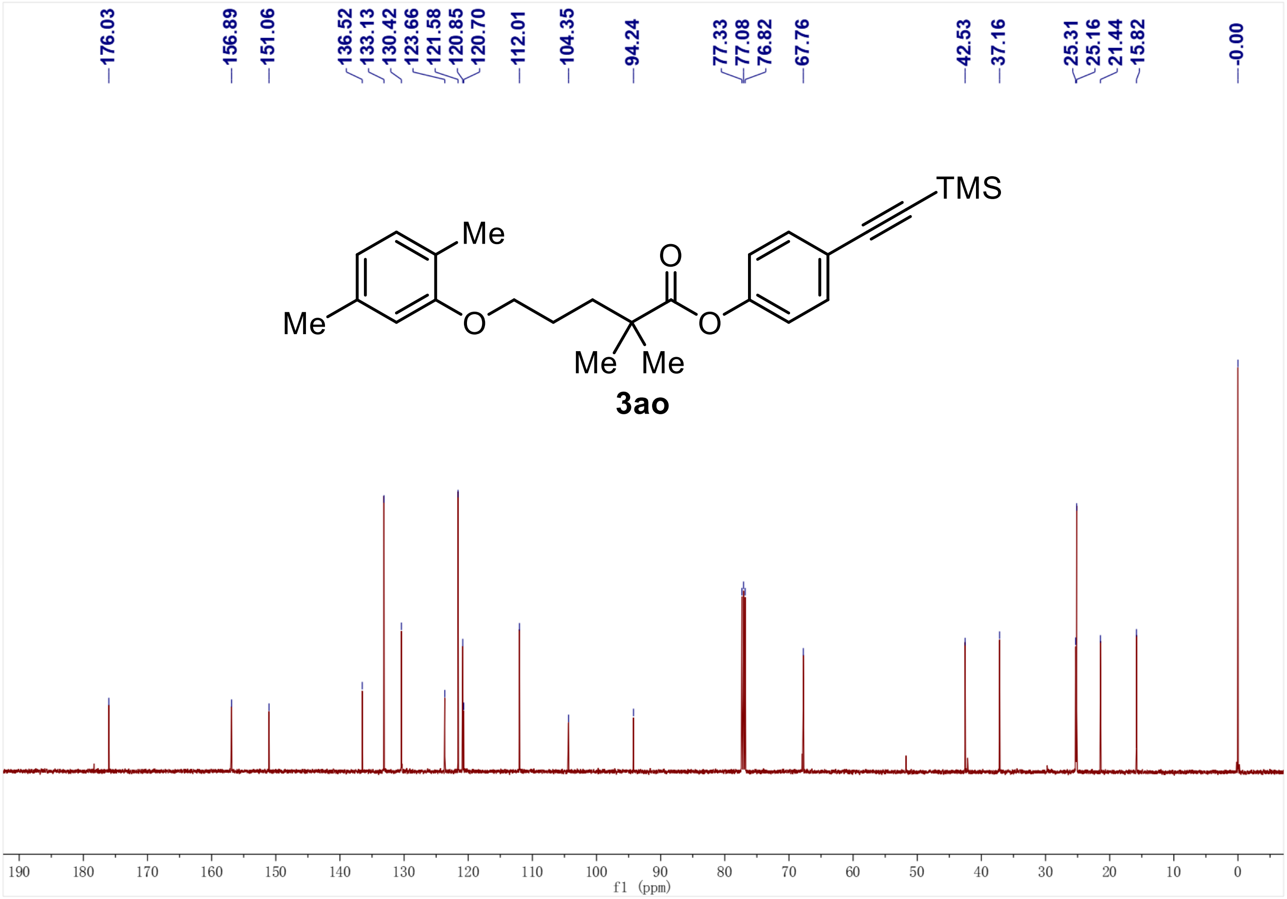

3ap; ^{1}H NMR (500 MHz, CDCl$_3$); ^{13}C NMR (126 MHz, CDCl$_3$)

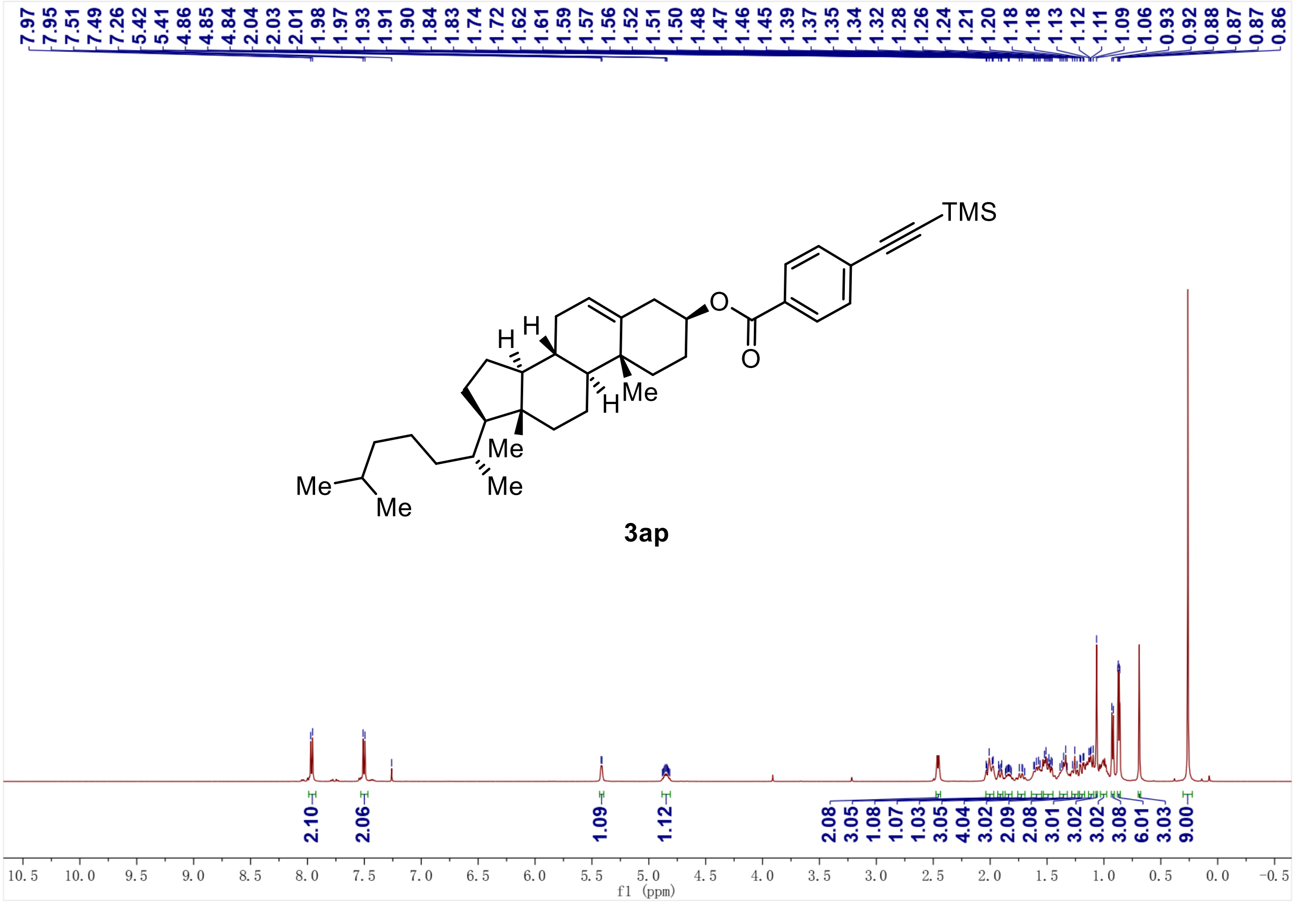

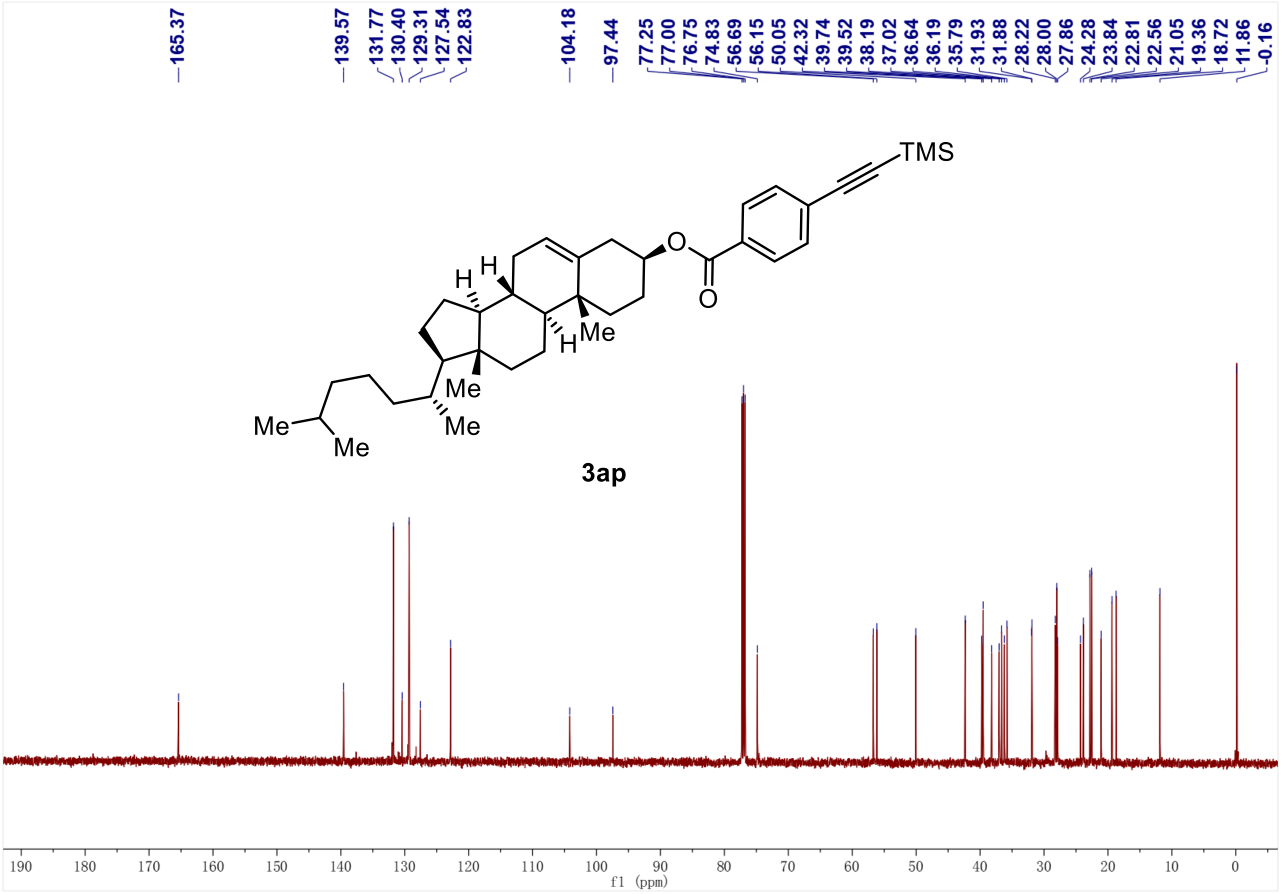

3aq; ^{1}H NMR (500 MHz, CDCl$_3$); ^{13}C NMR (126 MHz, CDCl$_3$)

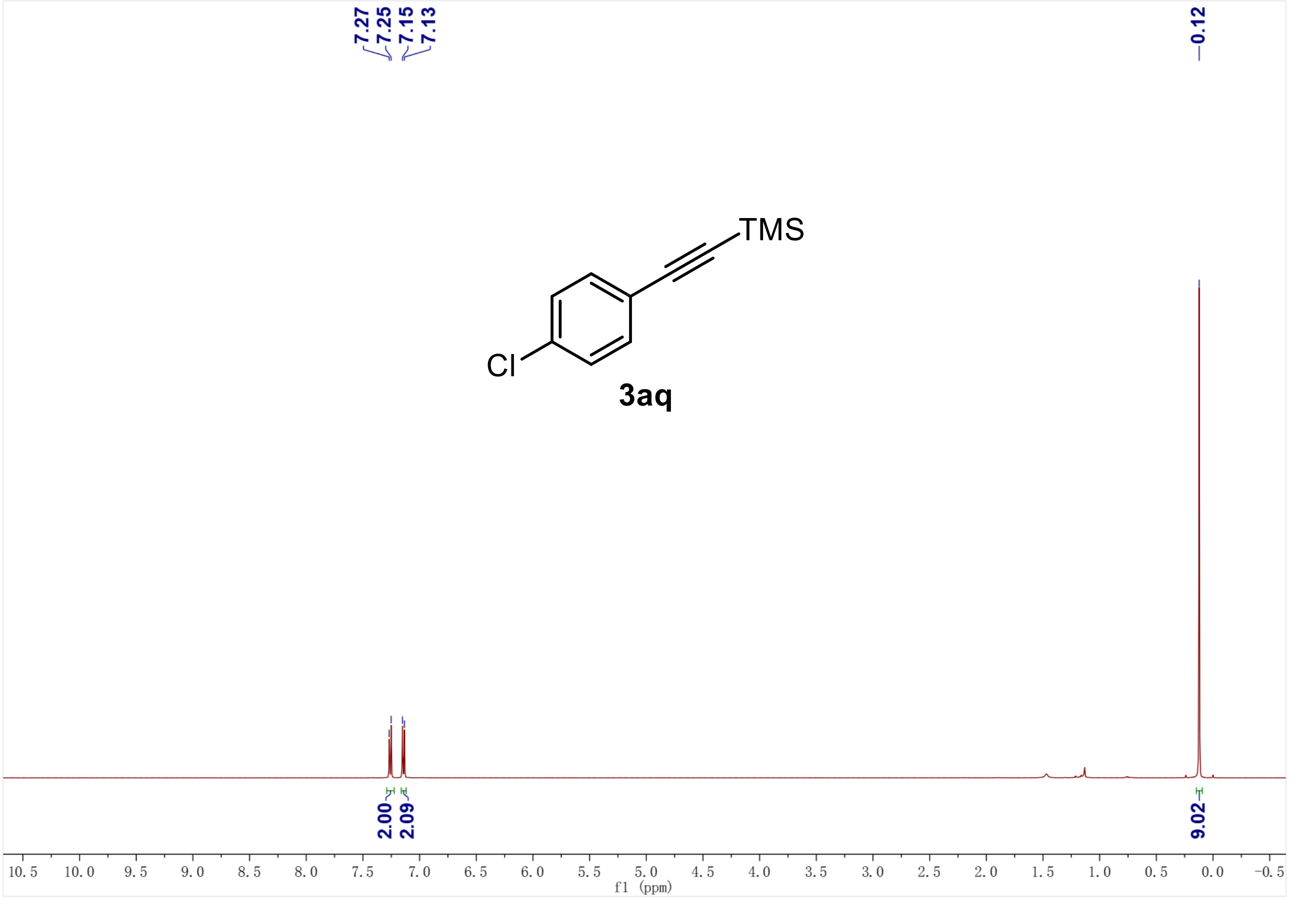

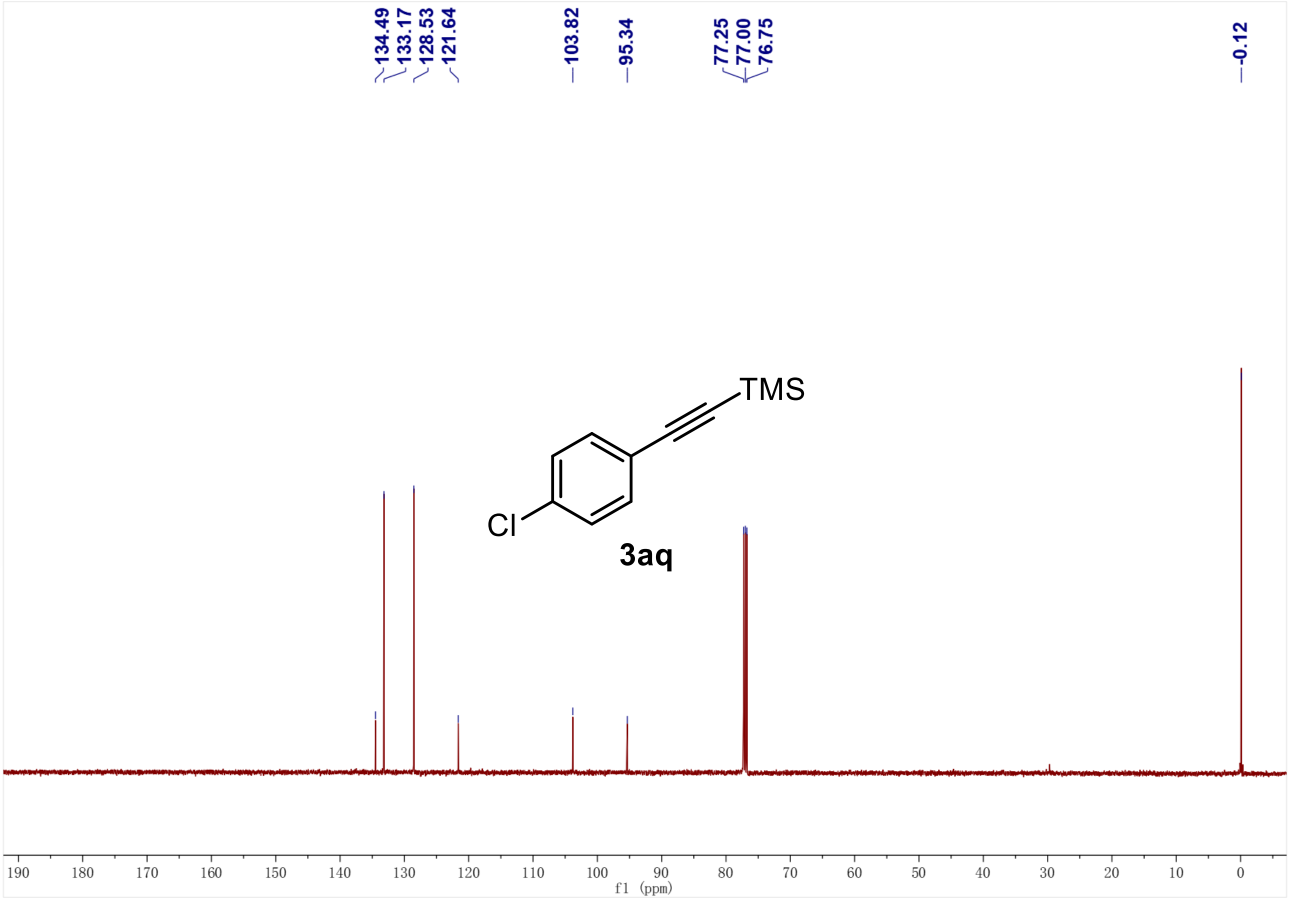

3ar; ¹H NMR (500 MHz, $CDCl_3$); ¹³C NMR (126 MHz, $CDCl_3$)

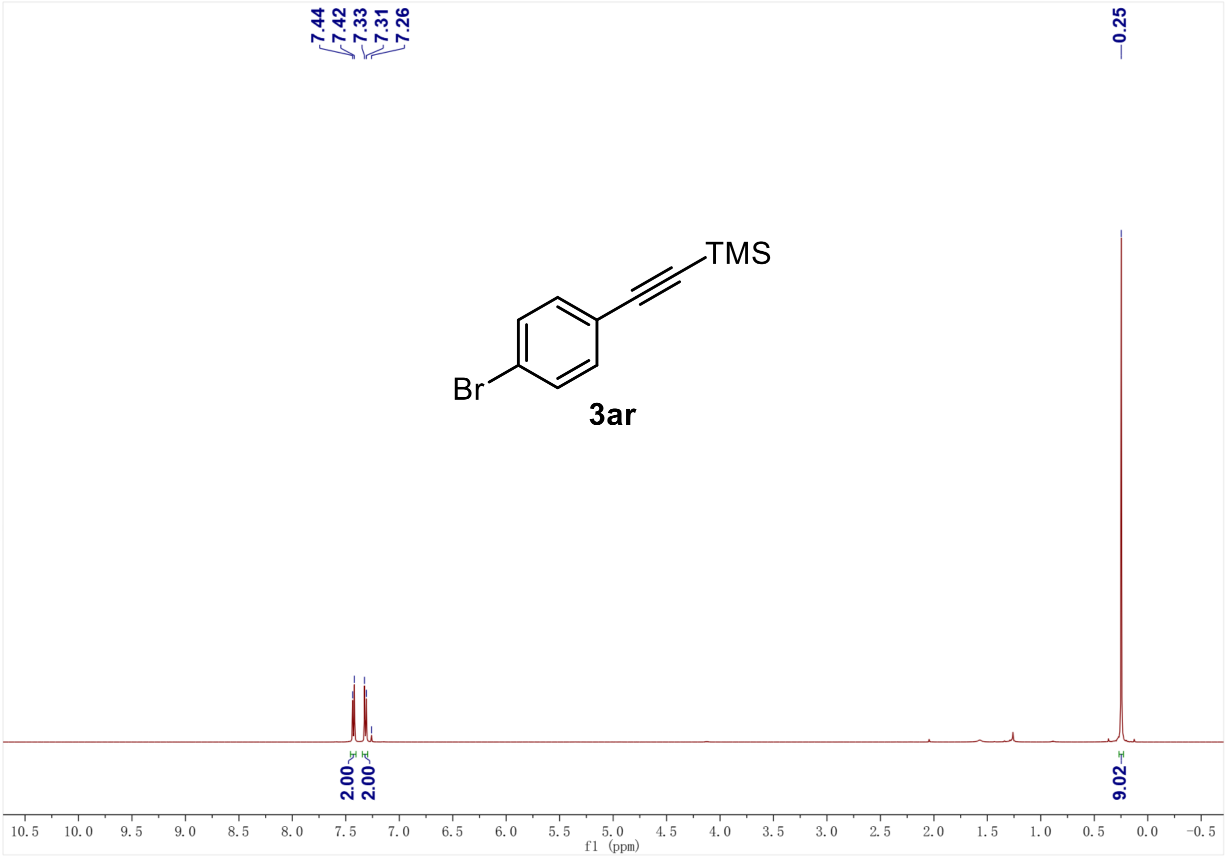

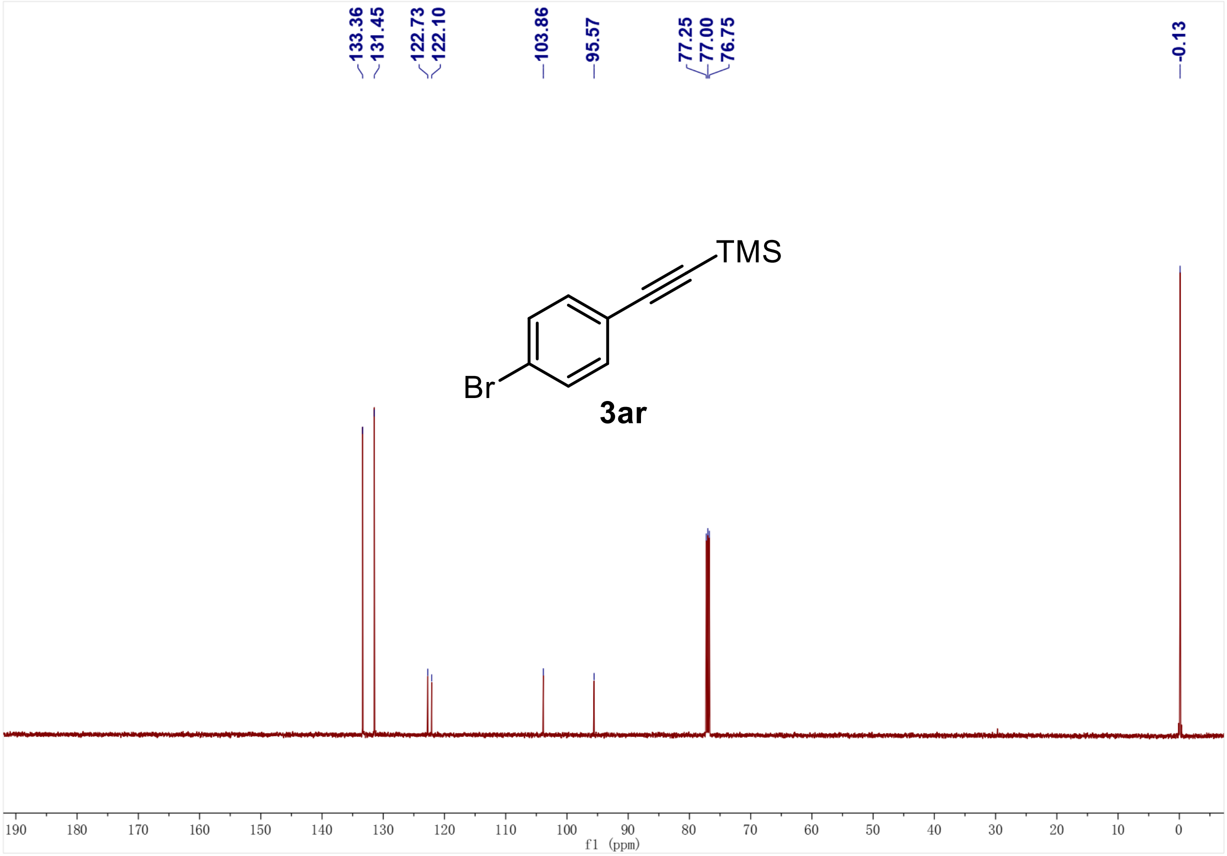

3as; ^{1}H NMR (500 MHz, $CDCl_3$); ^{13}C NMR (126 MHz, $CDCl_3$)

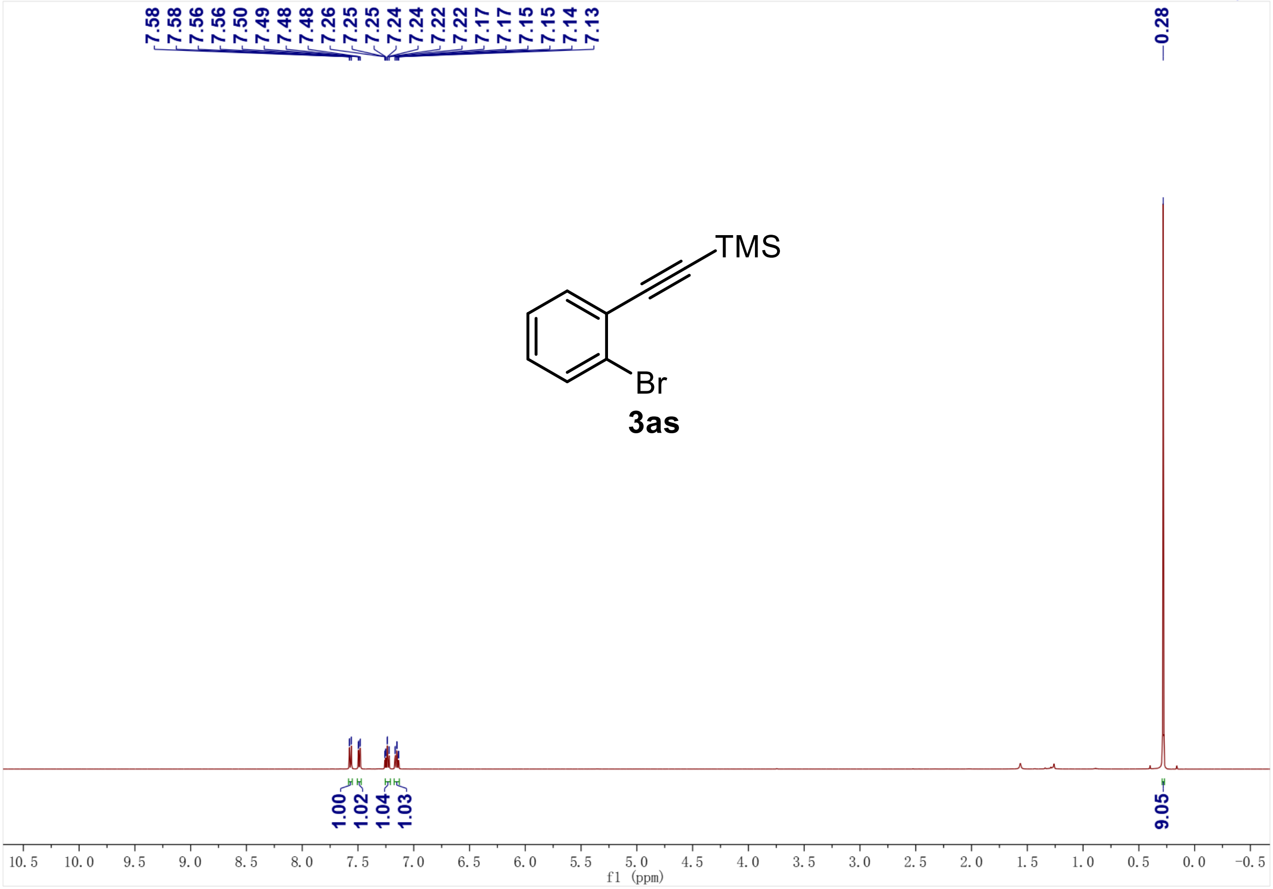

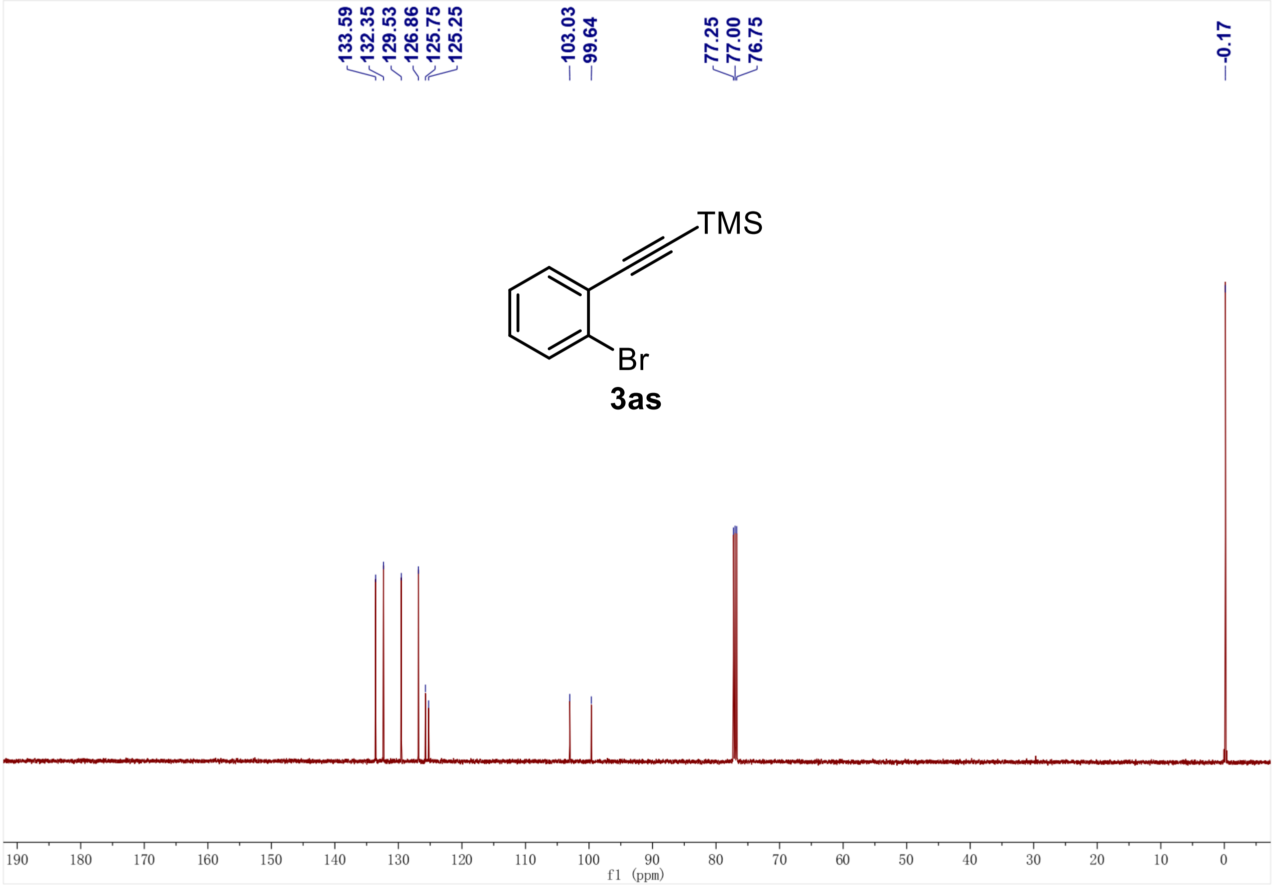

3at; ^{1}H NMR (500 MHz, $CDCl_3$); ^{13}C NMR (126 MHz, $CDCl_3$)

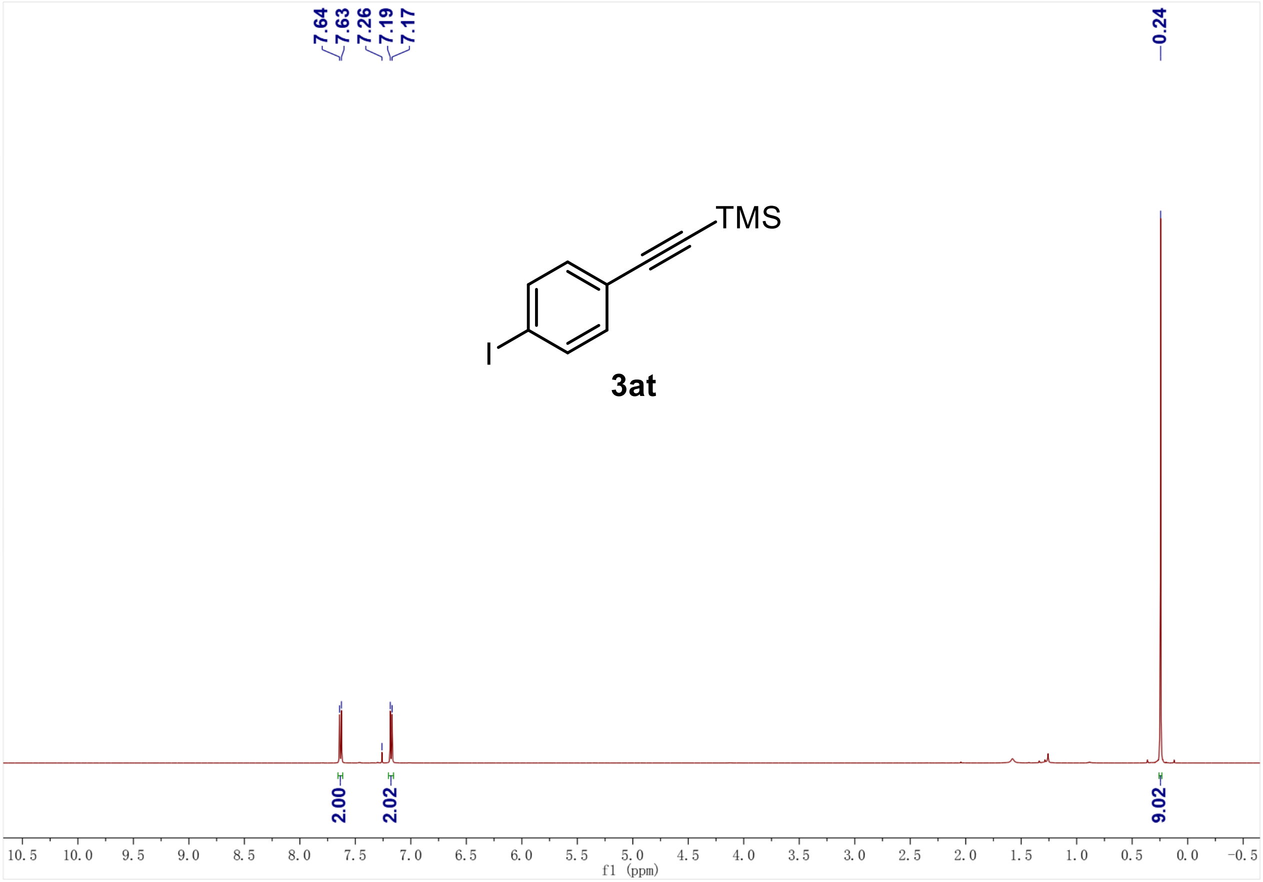

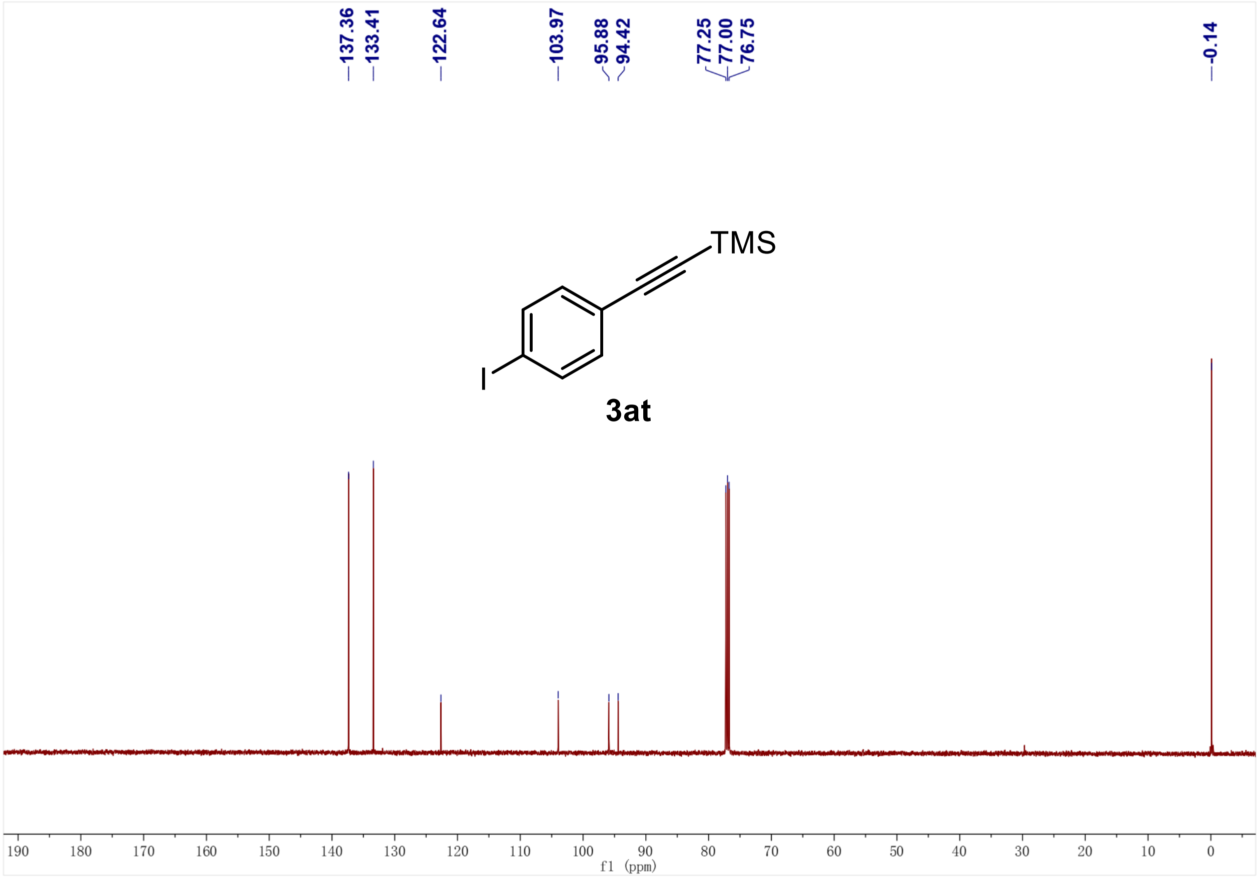

3au; ¹H NMR (400 MHz, CDCl$_3$); ¹³C NMR (101 MHz, CDCl$_3$)

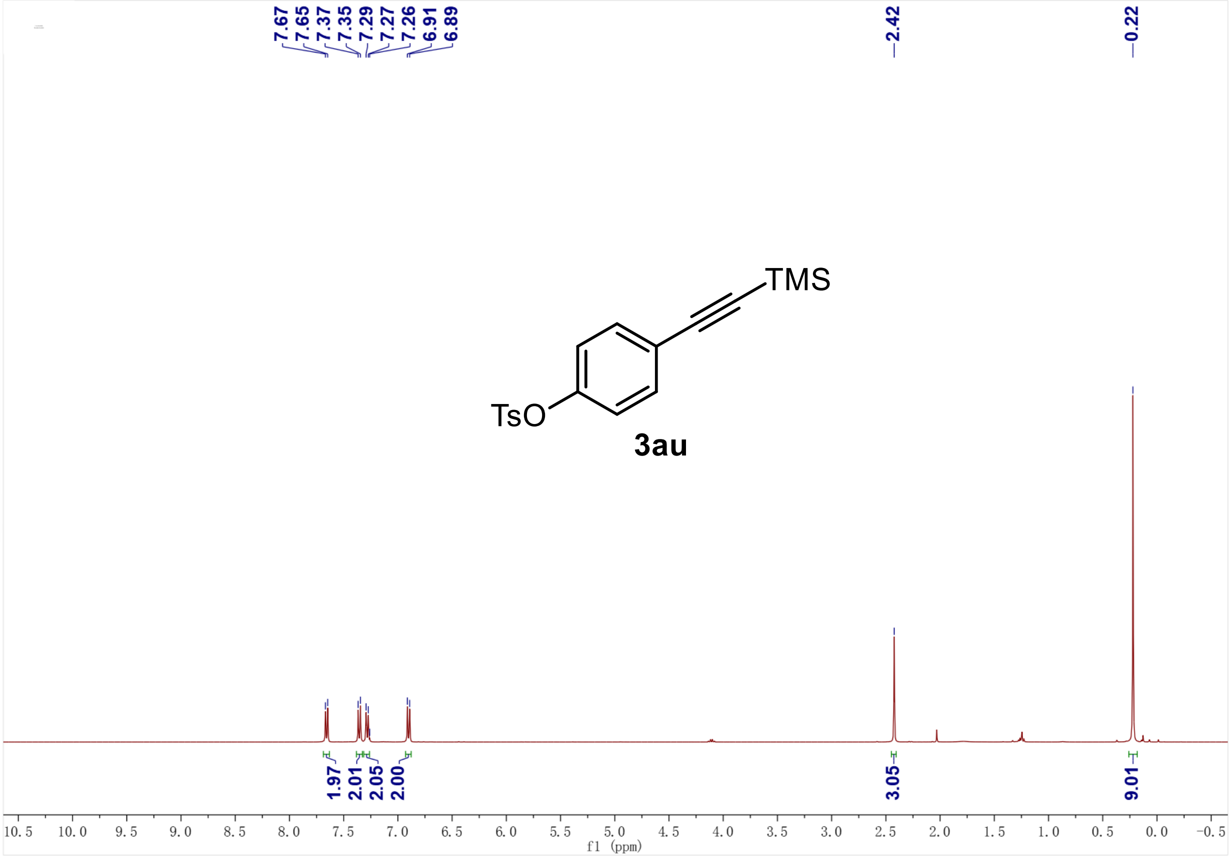

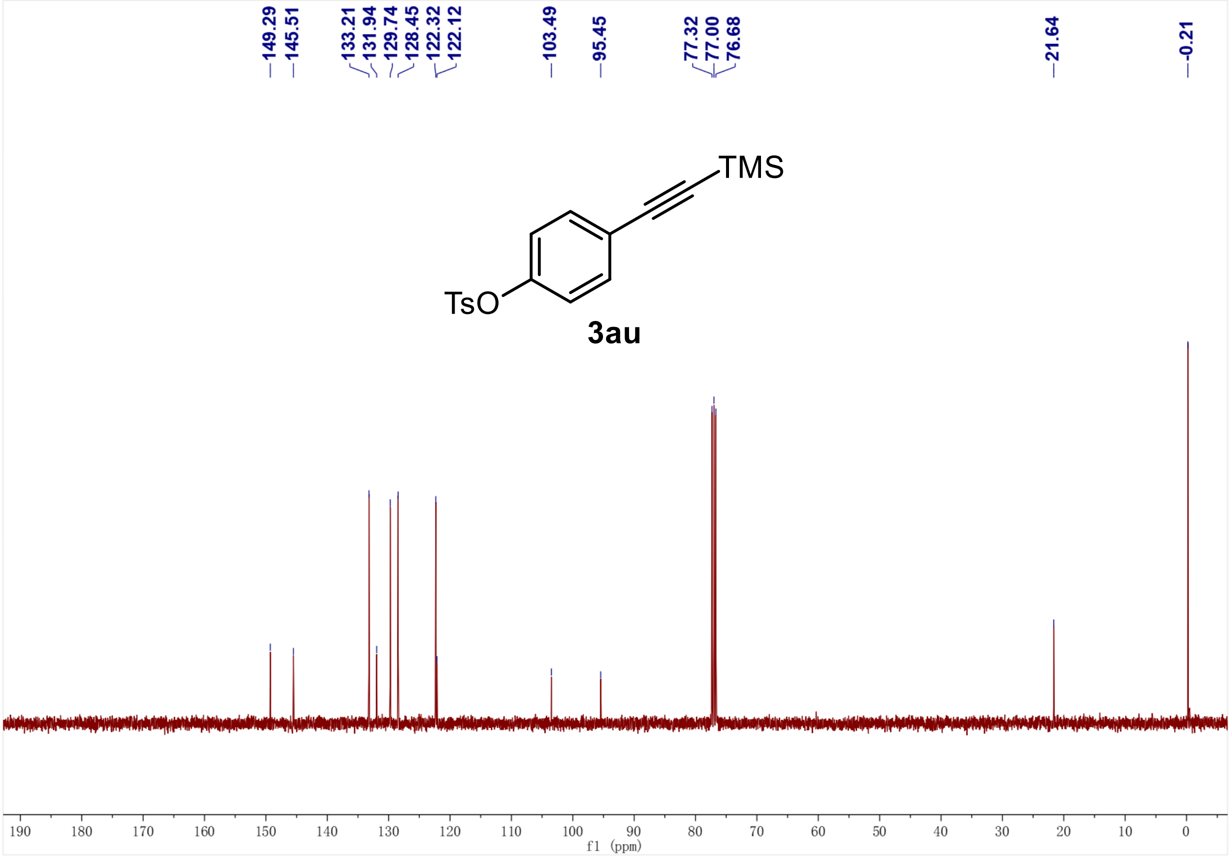

3av; ^{1}H NMR (400 MHz, $CDCl_3$); ^{13}C NMR (101 MHz, $CDCl_3$)

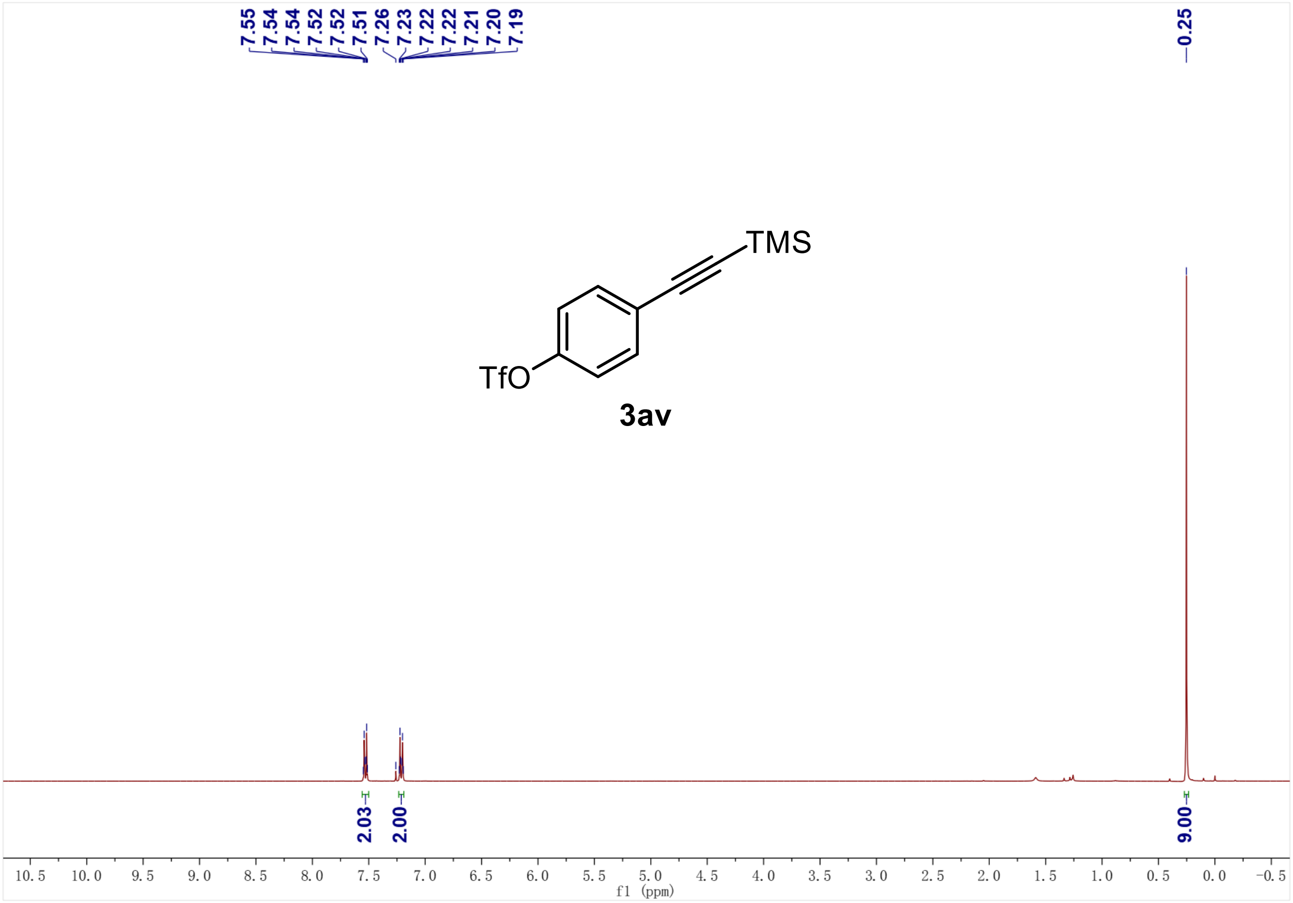

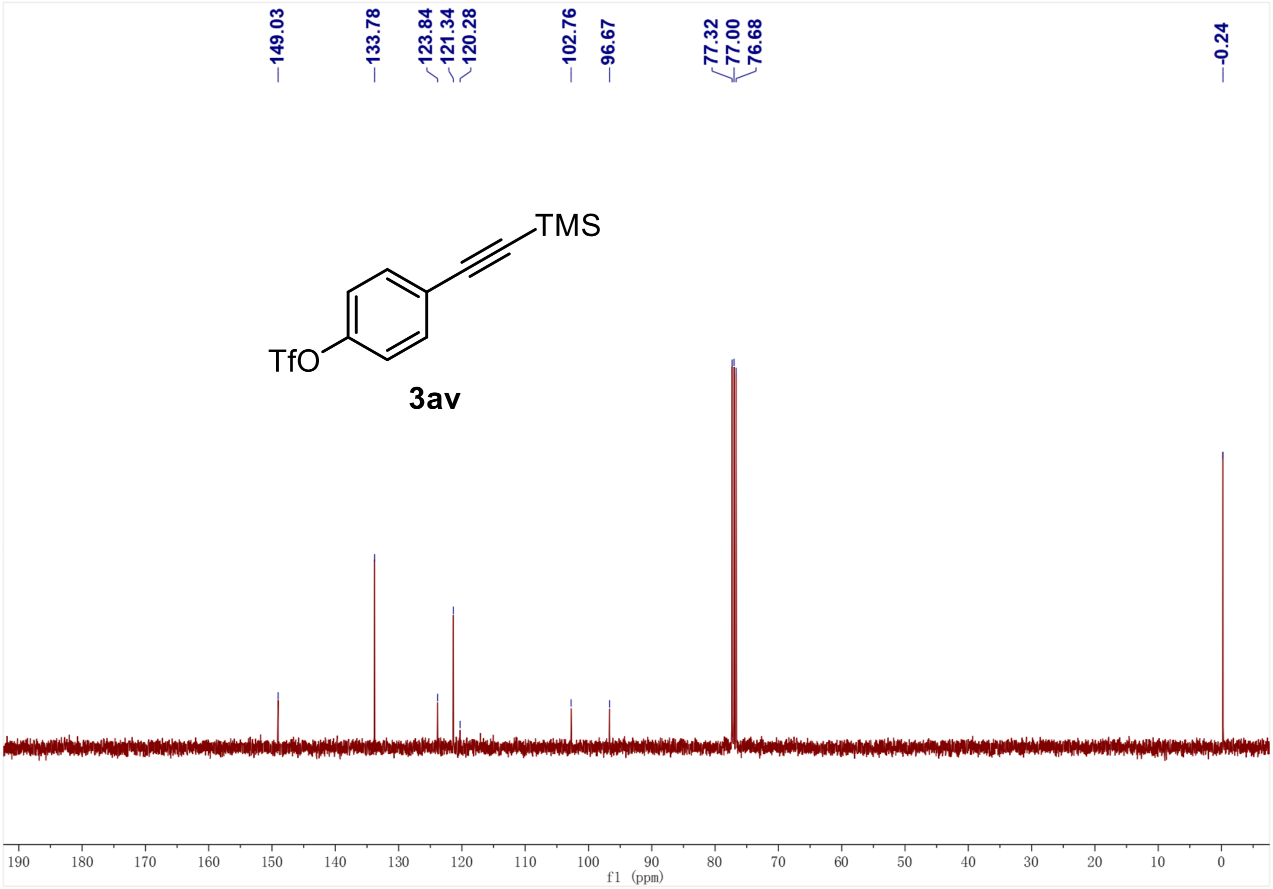

3aw; ^{1}H NMR (500 MHz, $CDCl_3$); ^{13}C NMR (126 MHz, $CDCl_3$)

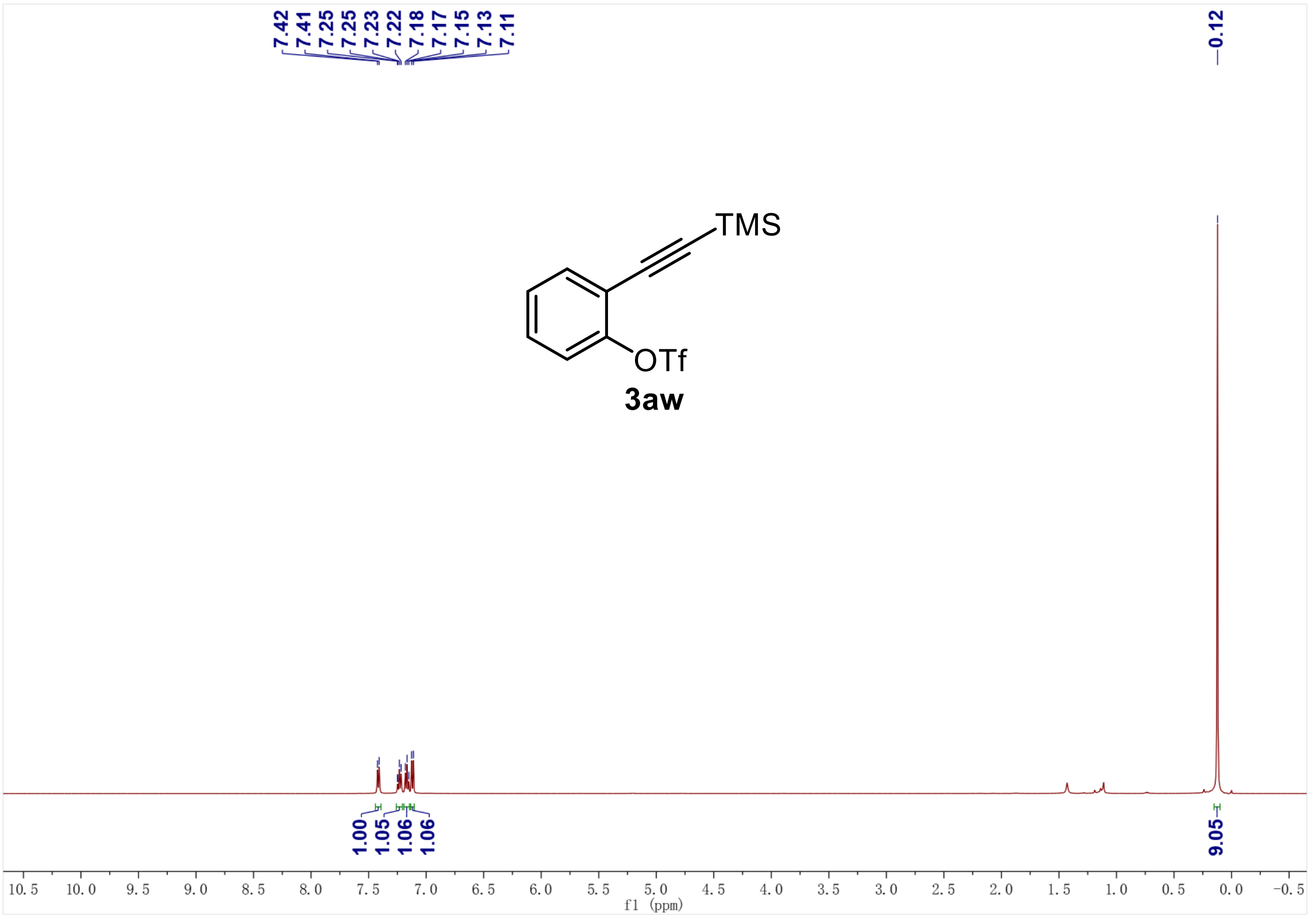

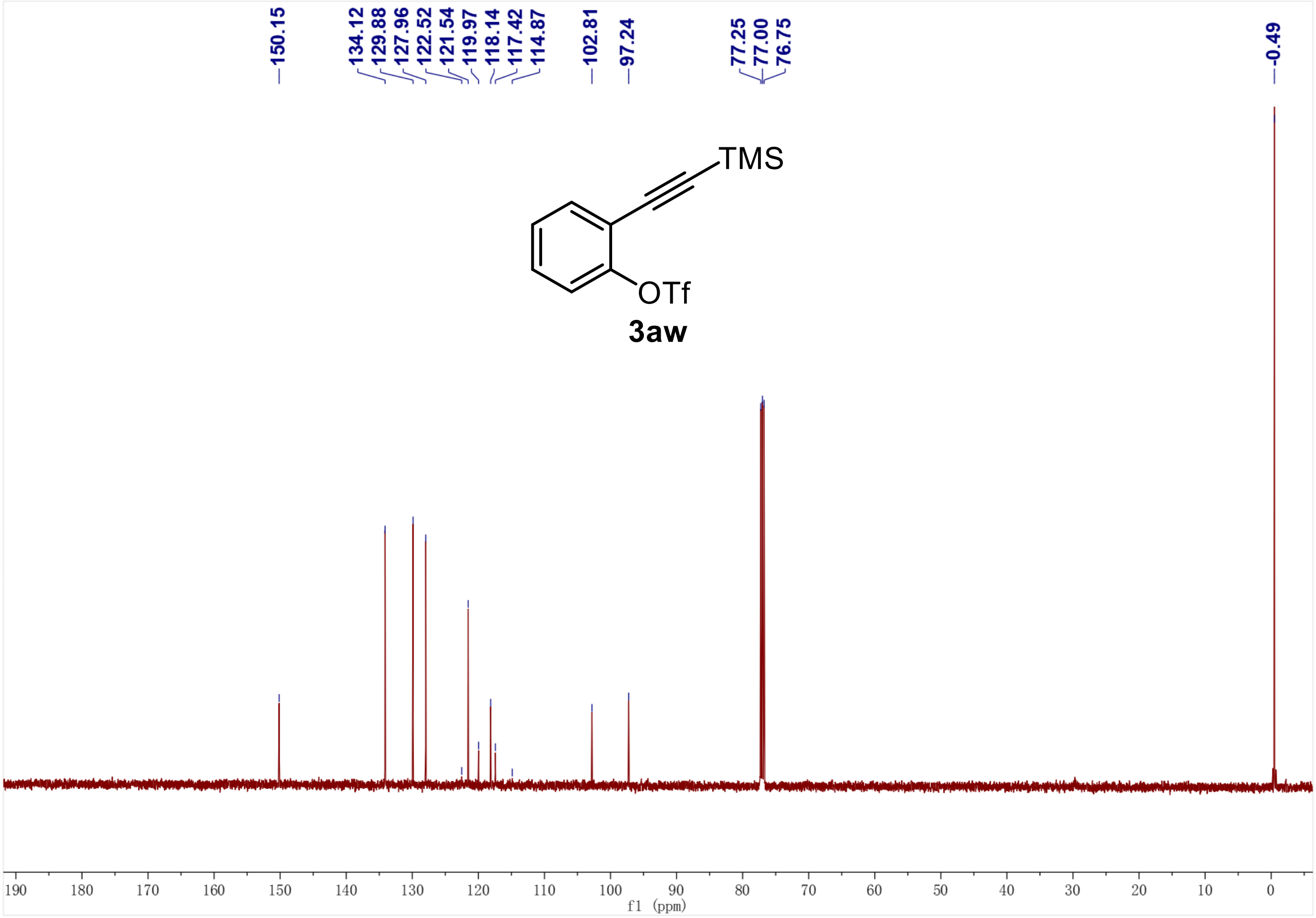

3aw; ^{19}F NMR (471 MHz, $CDCl_3$)

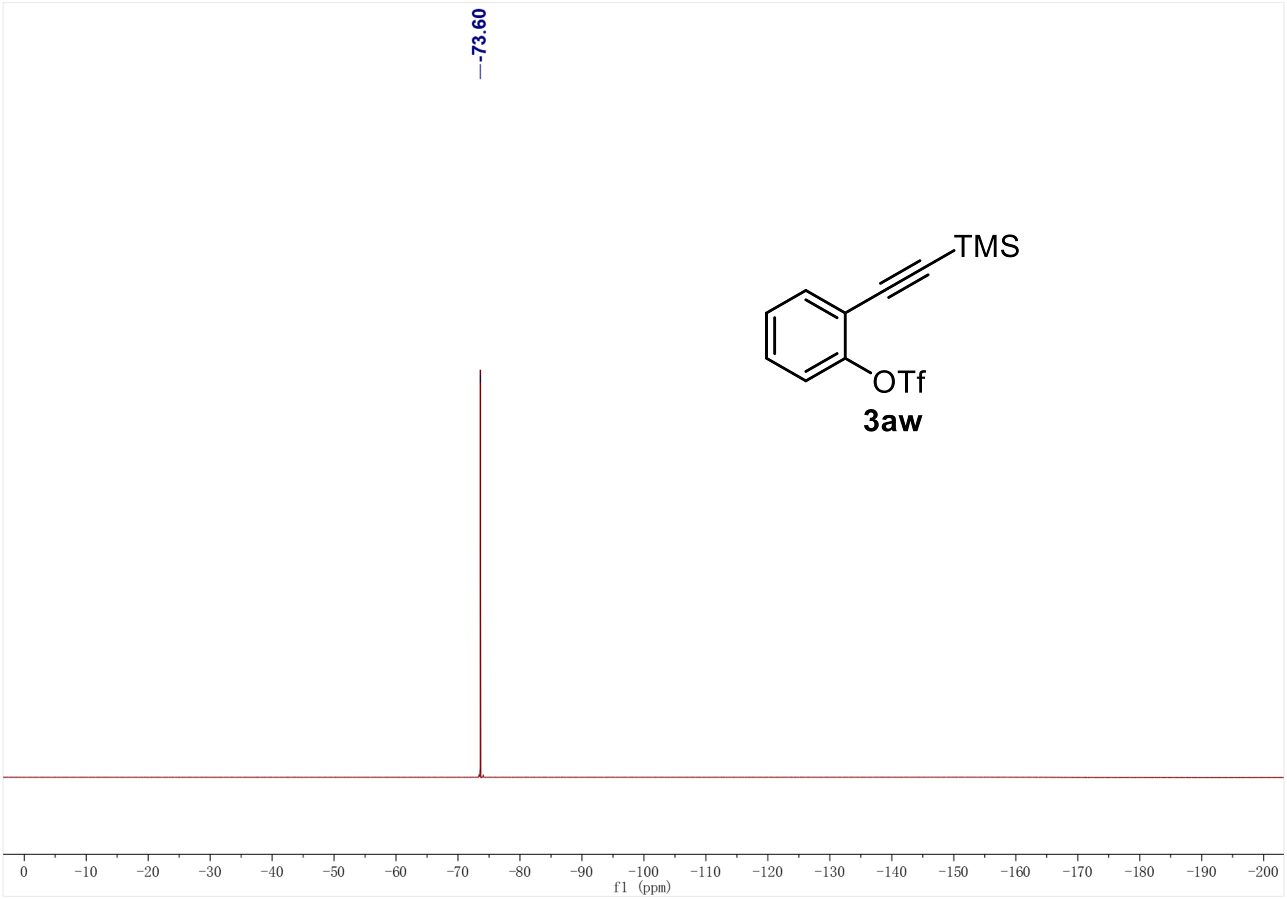

3ax; ^{1}H NMR (500 MHz, $CDCl_3$); ^{13}C NMR (126 MHz, $CDCl_3$)

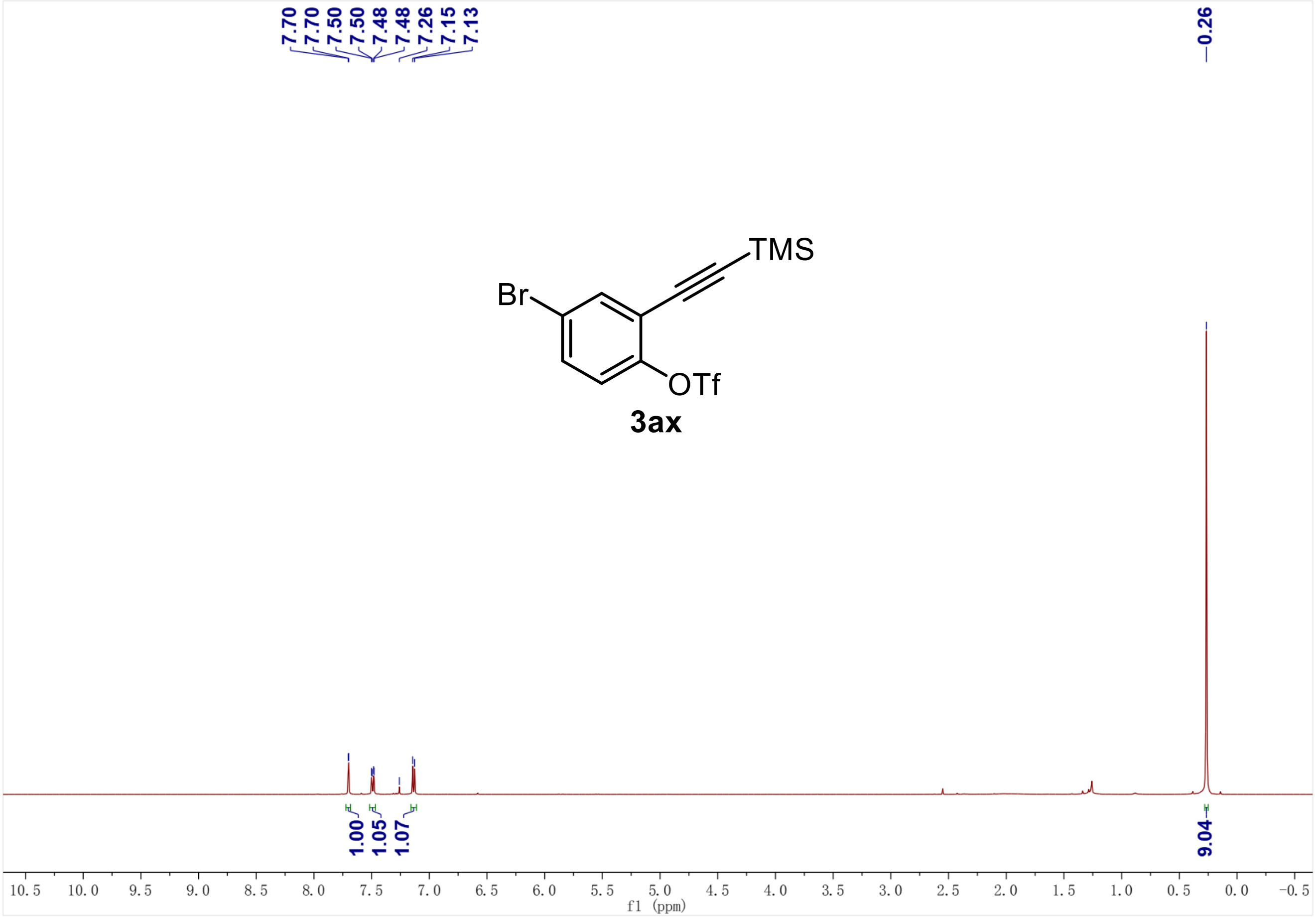

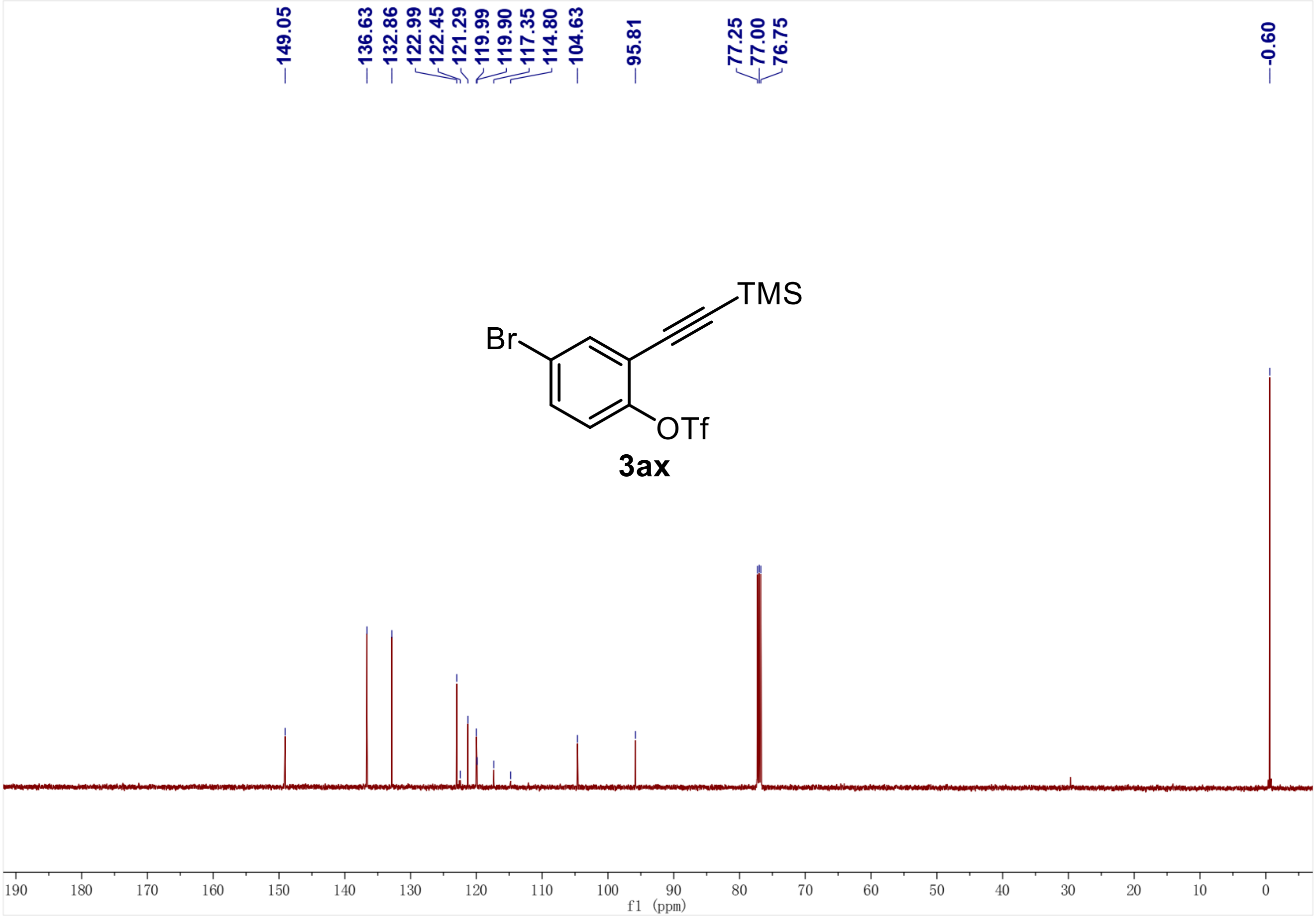

3ax; ^{19}F NMR (471 MHz, $CDCl_3$)

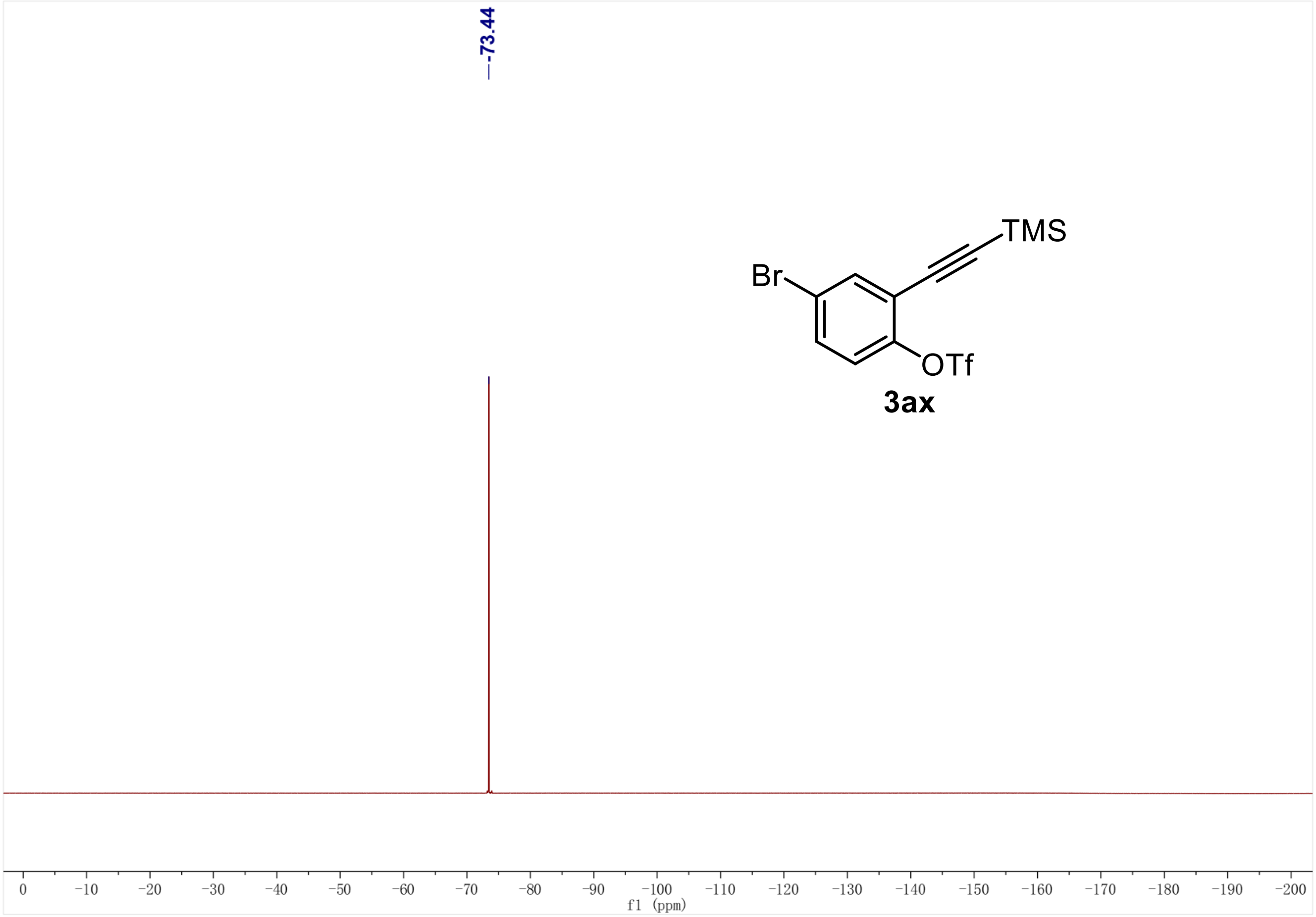

3ay; ^{1}H NMR (500 MHz, $CDCl_3$); ^{13}C NMR (126 MHz, $CDCl_3$)

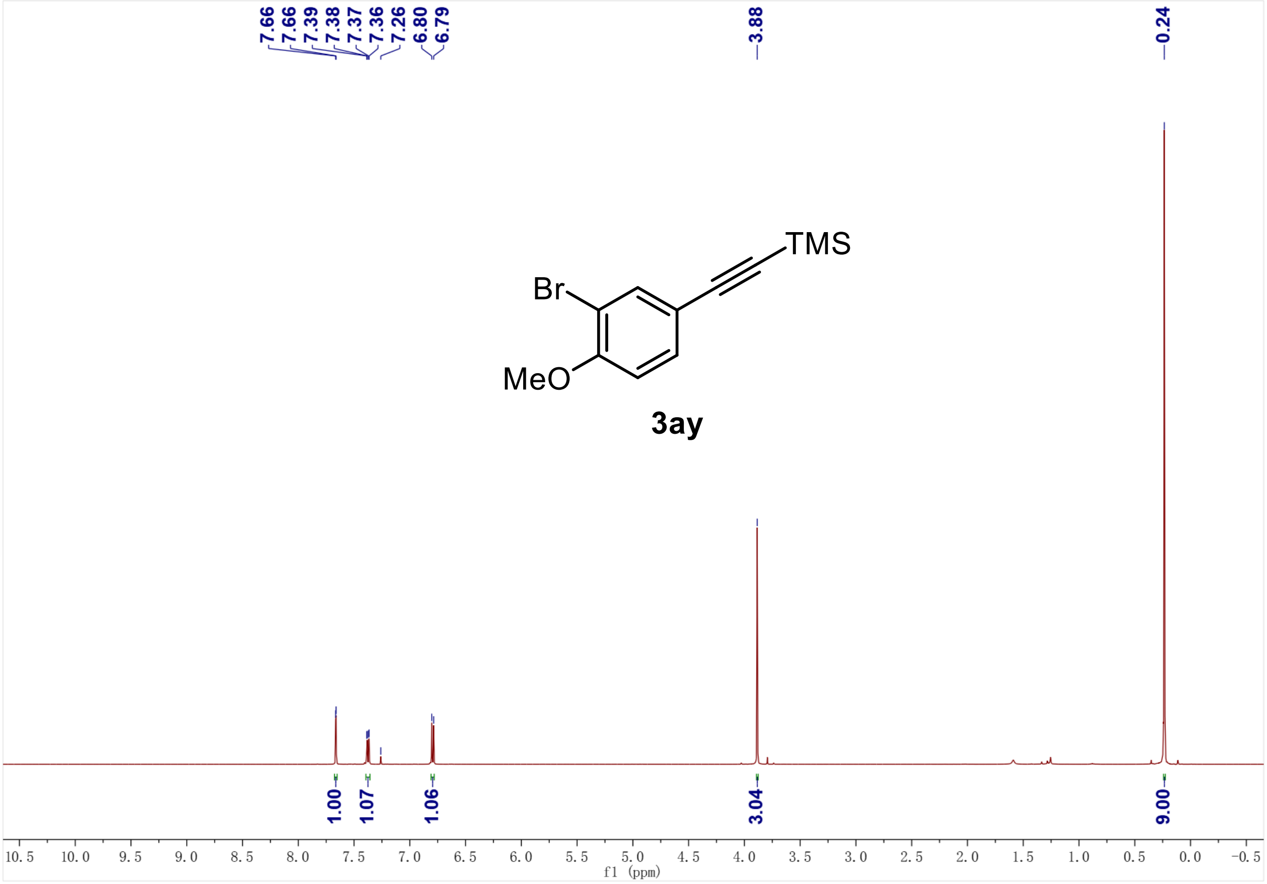

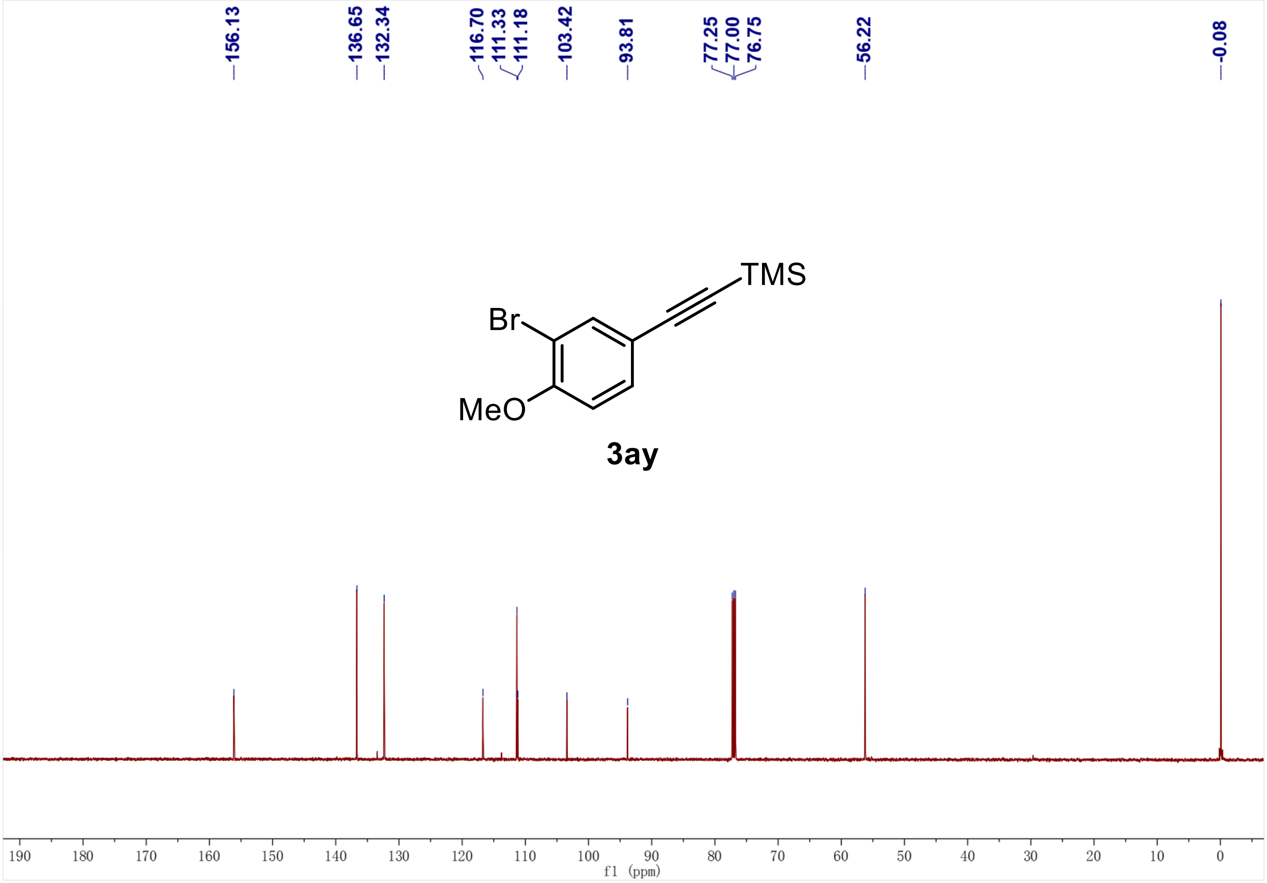

3az; ^{1}H NMR (500 MHz, $CDCl_3$); ^{13}C NMR (126 MHz, $CDCl_3$)

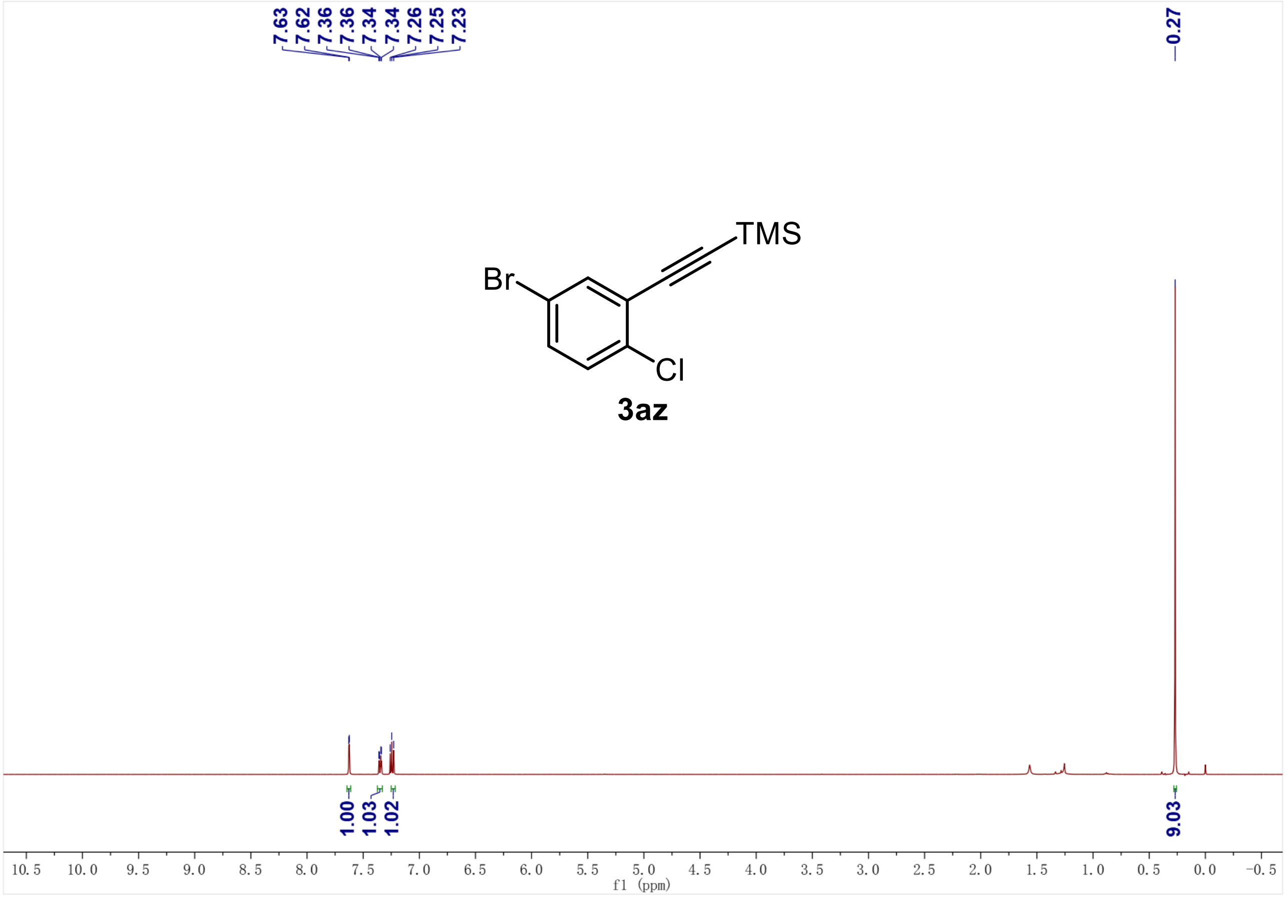

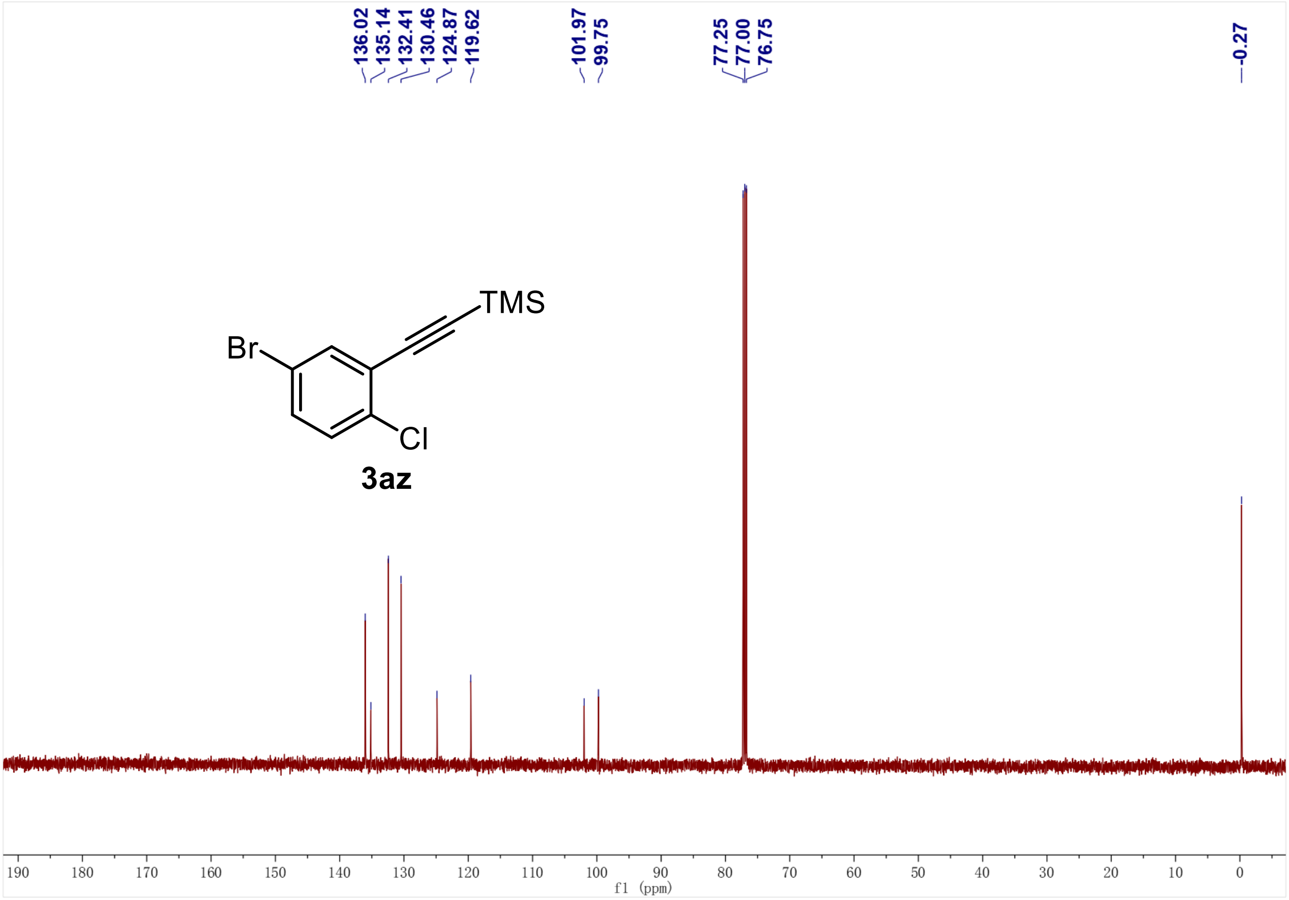

3ba; ^{1}H NMR (500 MHz, $CDCl_3$); ^{13}C NMR (126 MHz, $CDCl_3$)

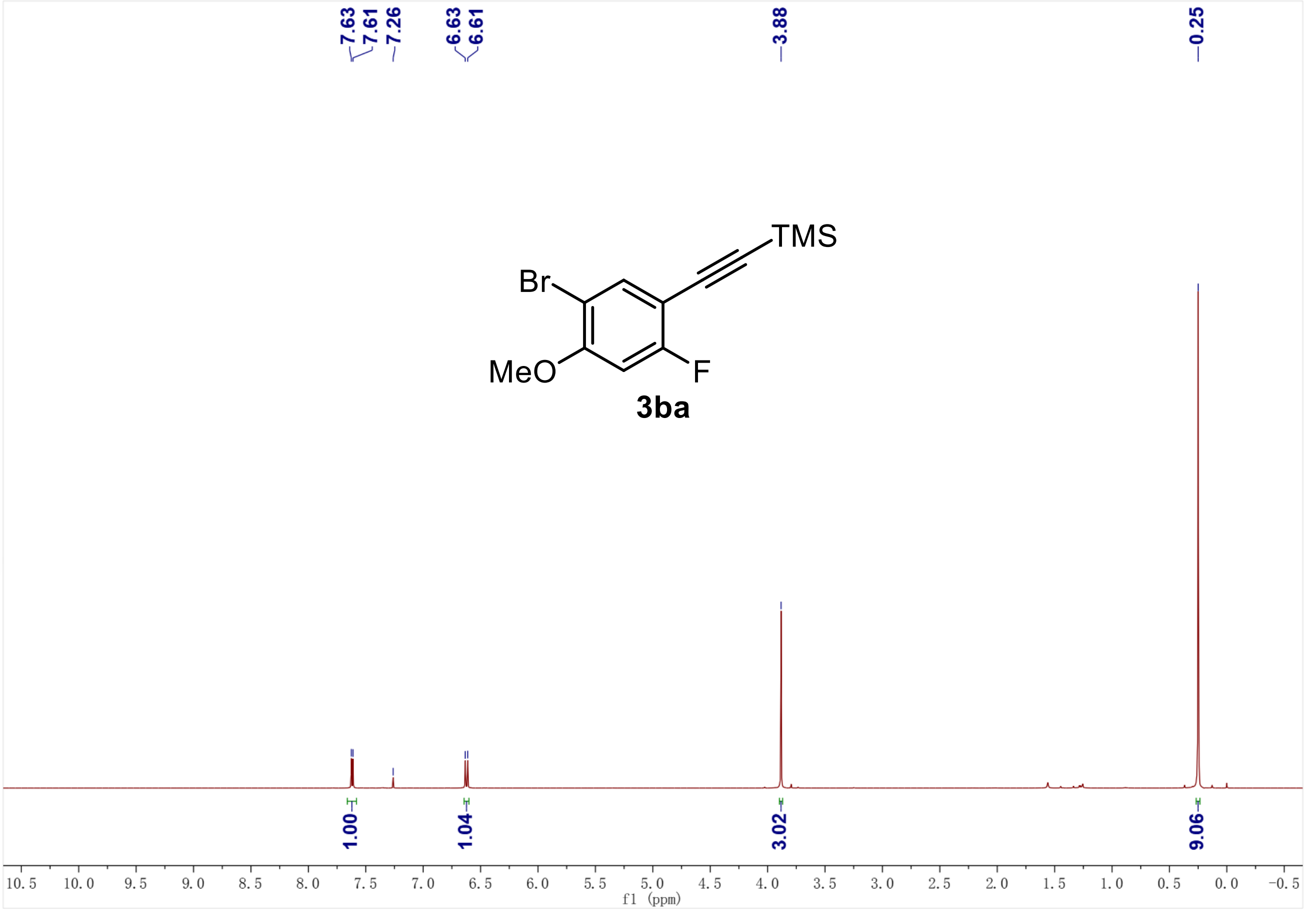

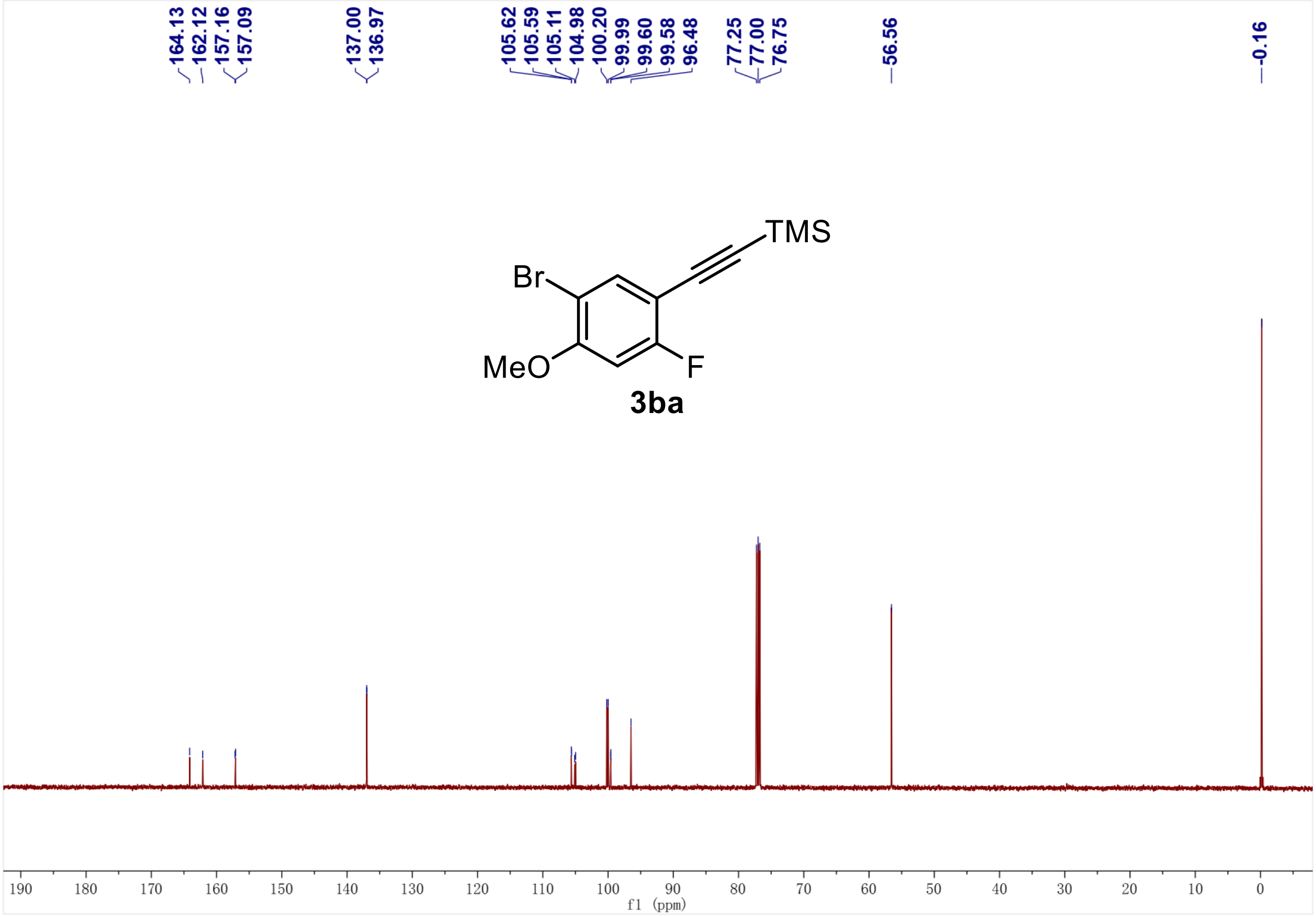

3ba; ^{19}F NMR (471 MHz, CDCl$_3$)

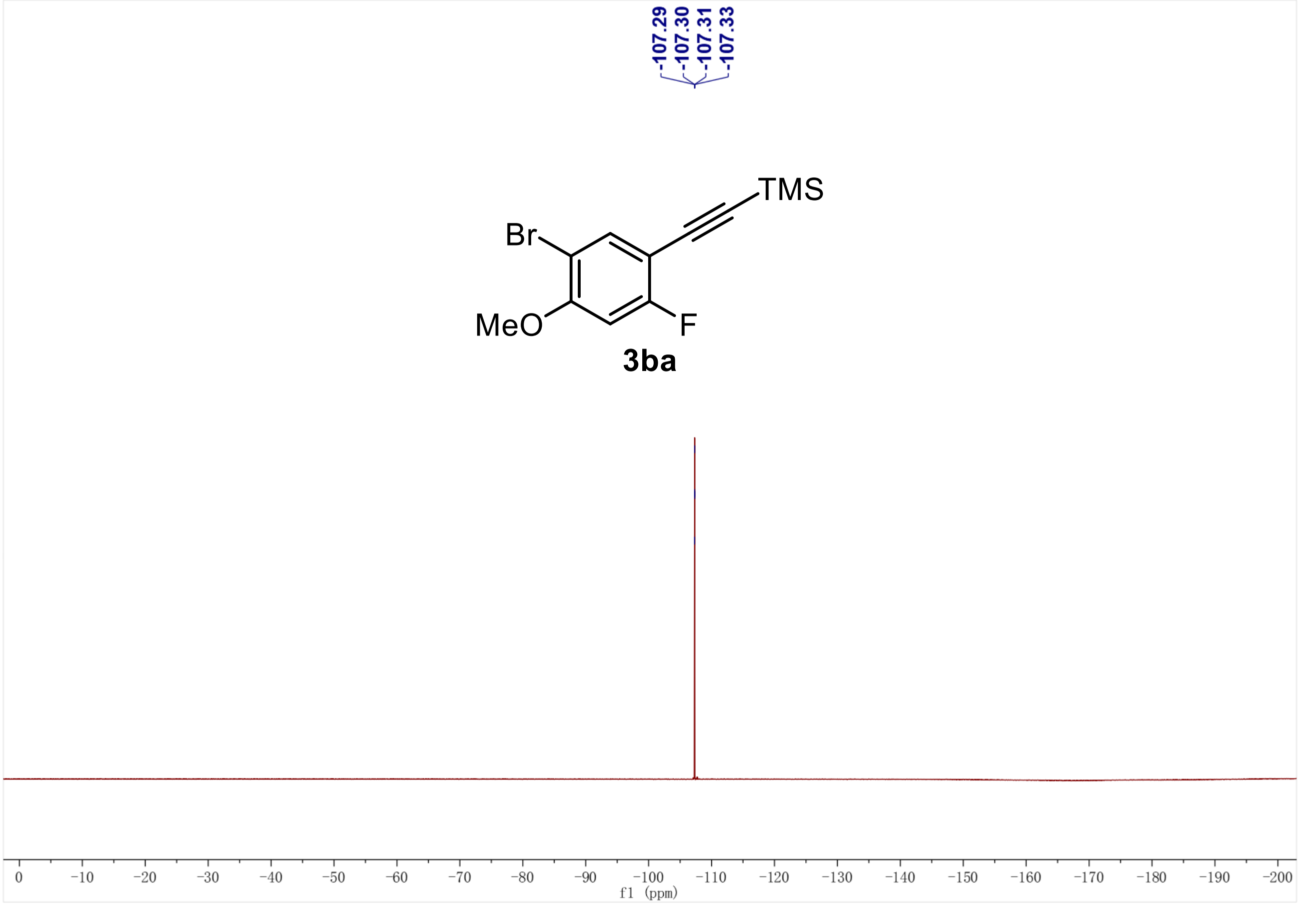

3bb; ^{1}H NMR (400 MHz, $CDCl_3$); ^{13}C NMR (126 MHz, $CDCl_3$)

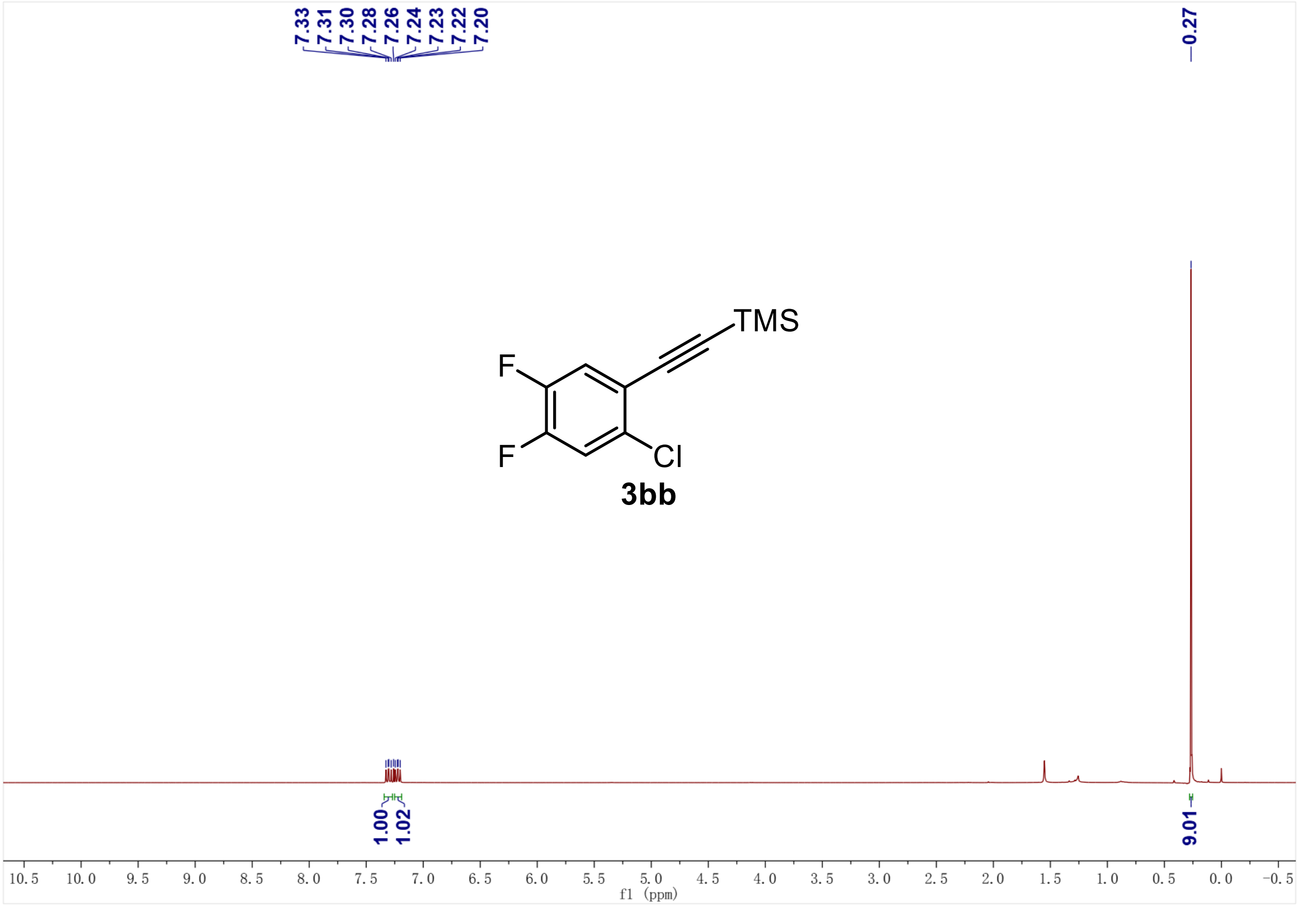

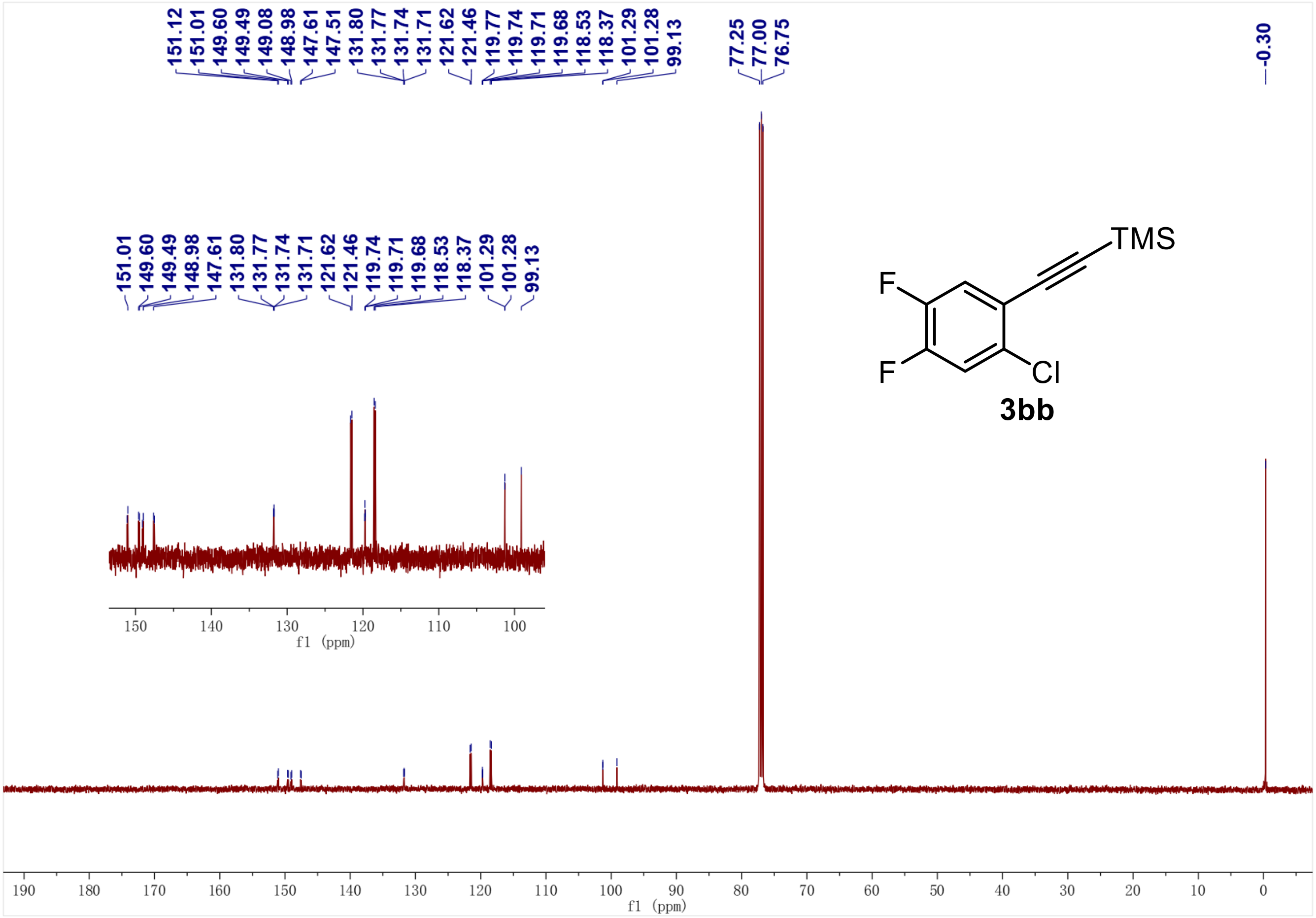

3bb; ^{19}F NMR (376 MHz, $CDCl_3$)

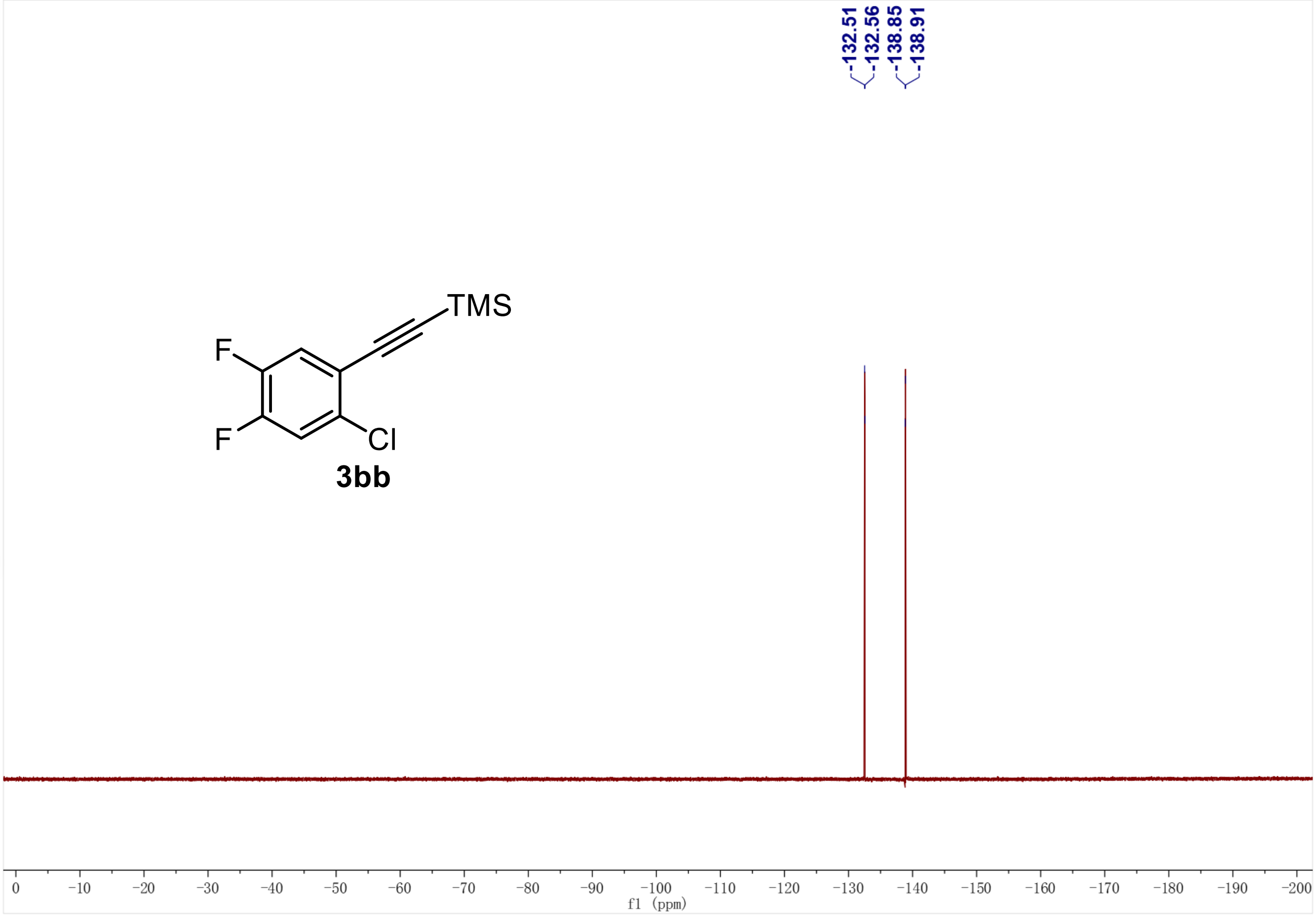

3bc; ^{1}H NMR (500 MHz, $CDCl_3$); ^{13}C NMR (126 MHz, $CDCl_3$)

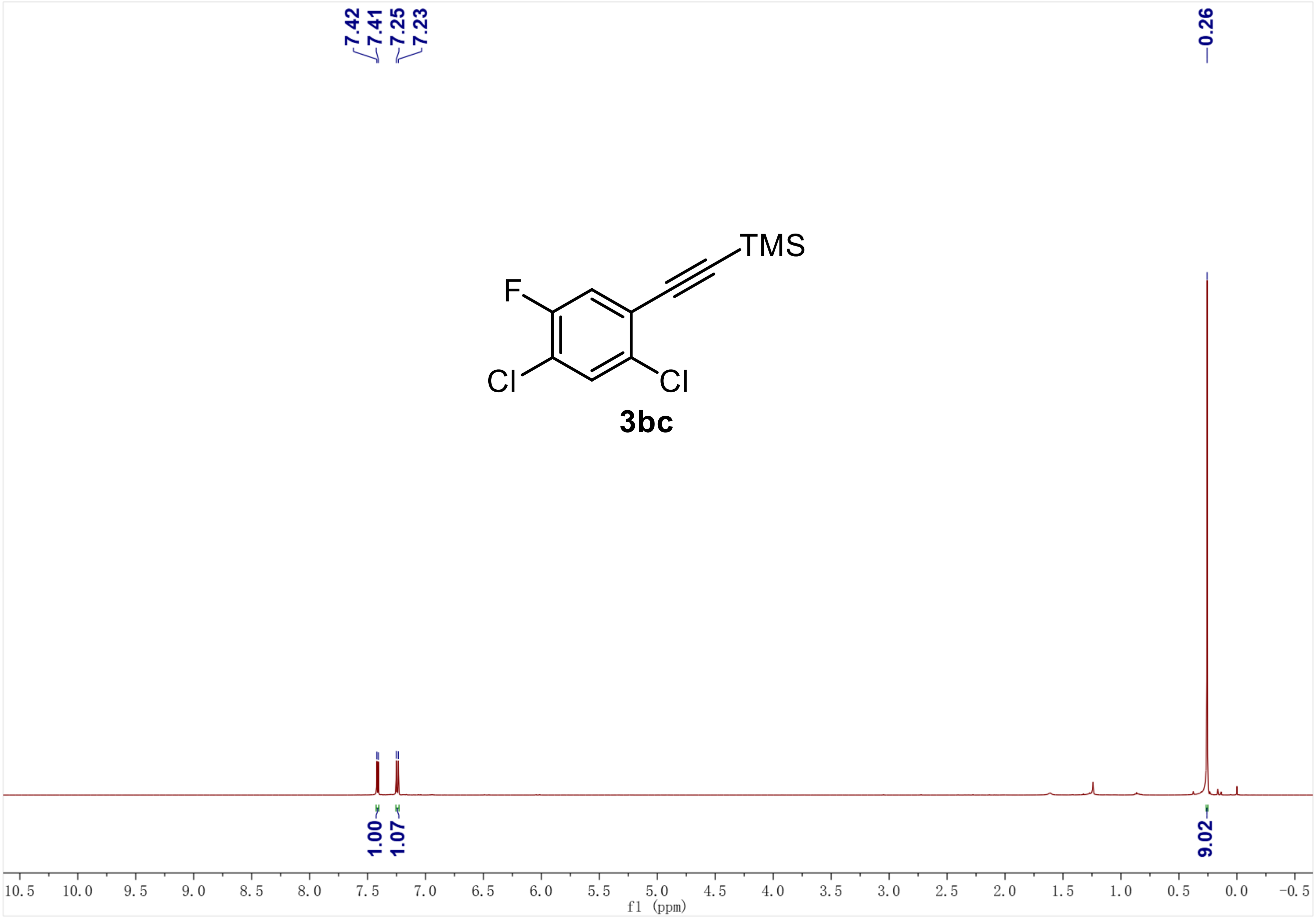

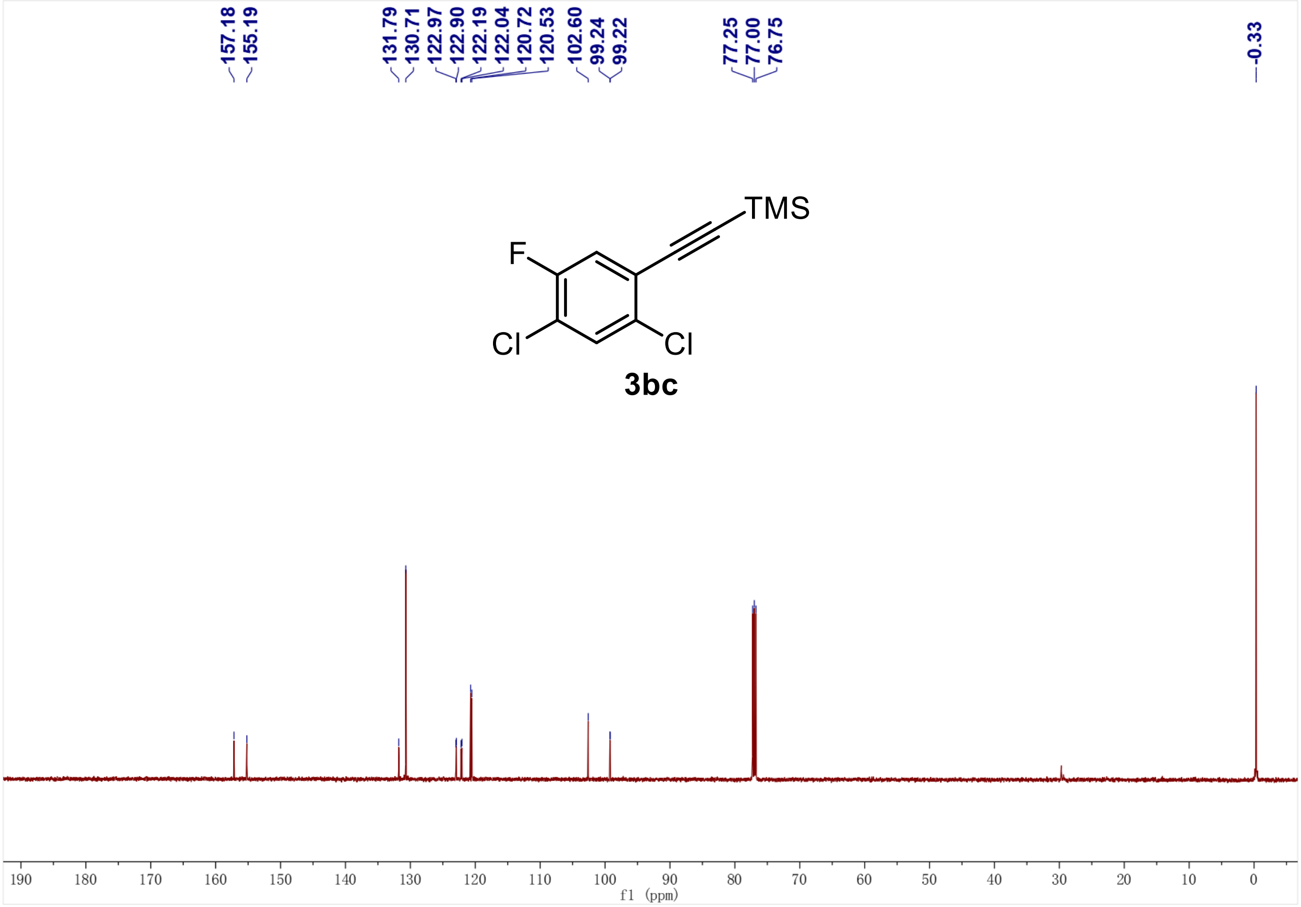

3bc; ^{19}F NMR (471 MHz, $CDCl_3$)

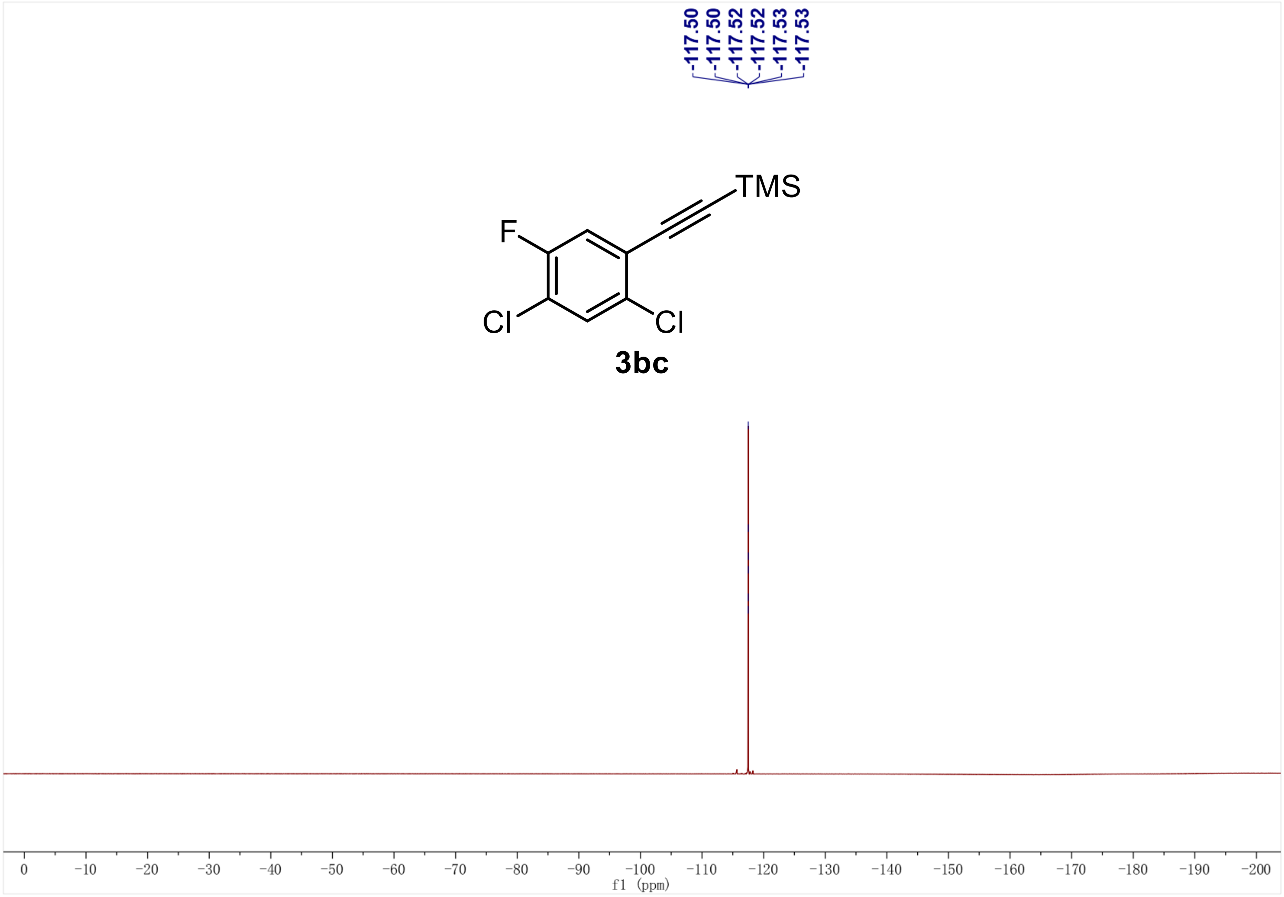

3bd; ¹H NMR (500 MHz, $CDCl_3$); ¹³C NMR (126 MHz, $CDCl_3$)

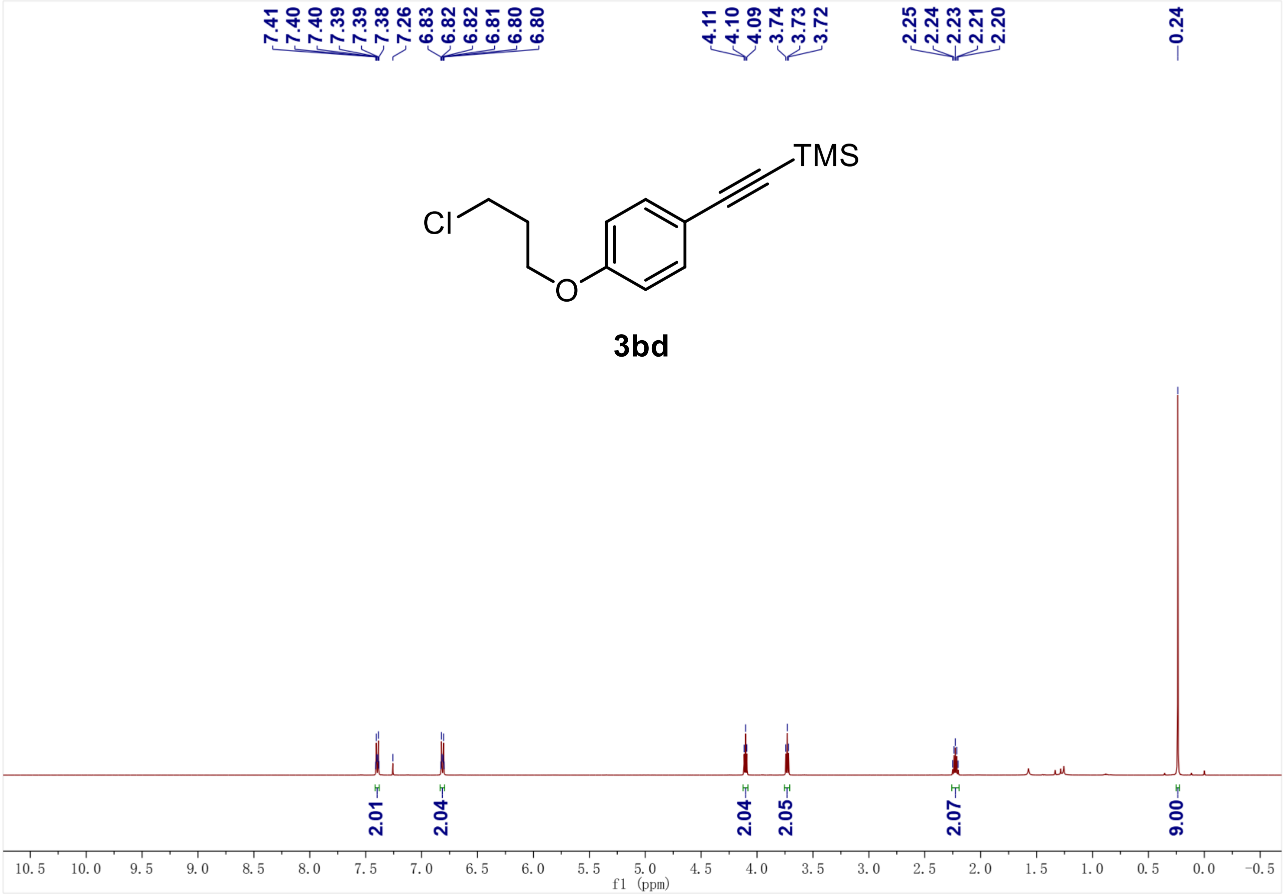

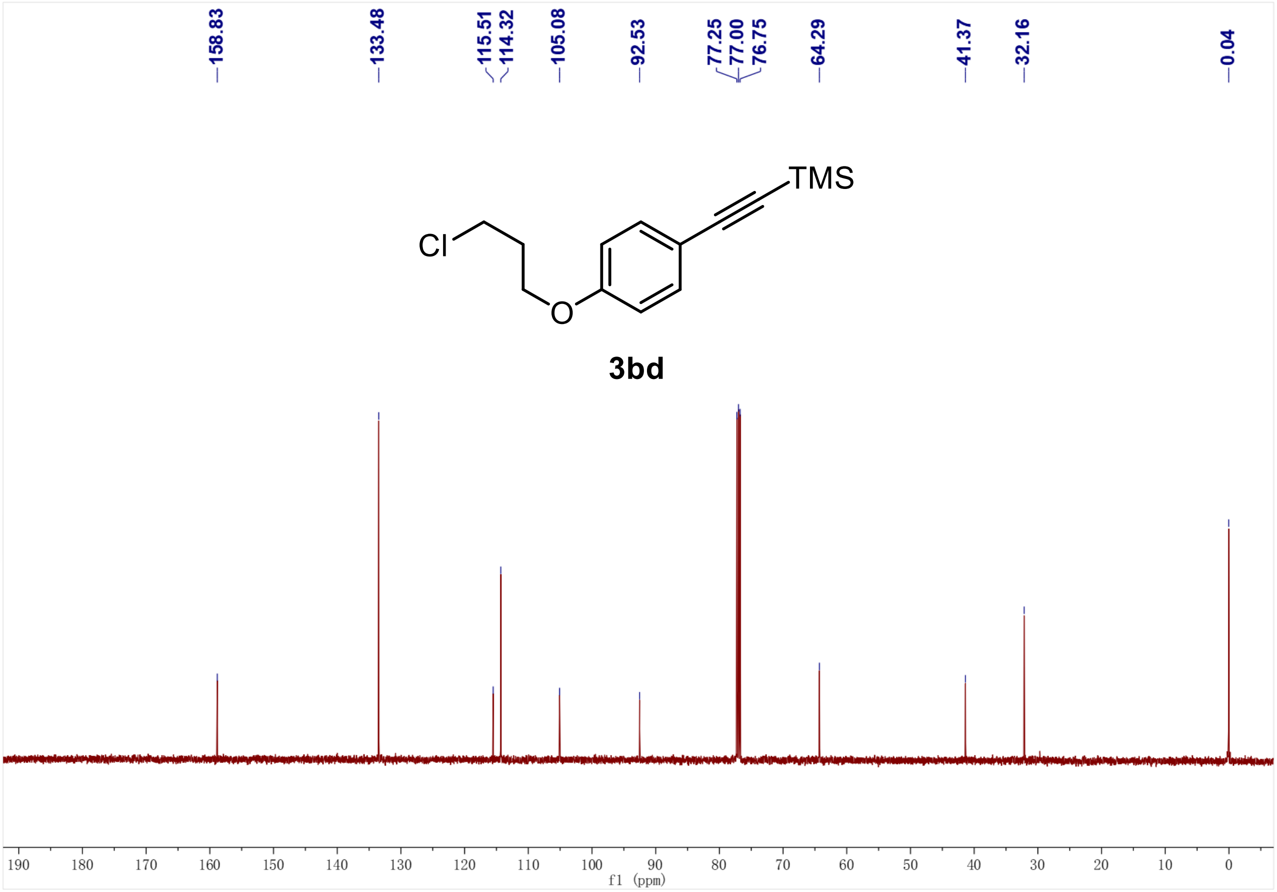

3be; ^{1}H NMR (500 MHz, $CDCl_3$); ^{13}C NMR (126 MHz, $CDCl_3$)

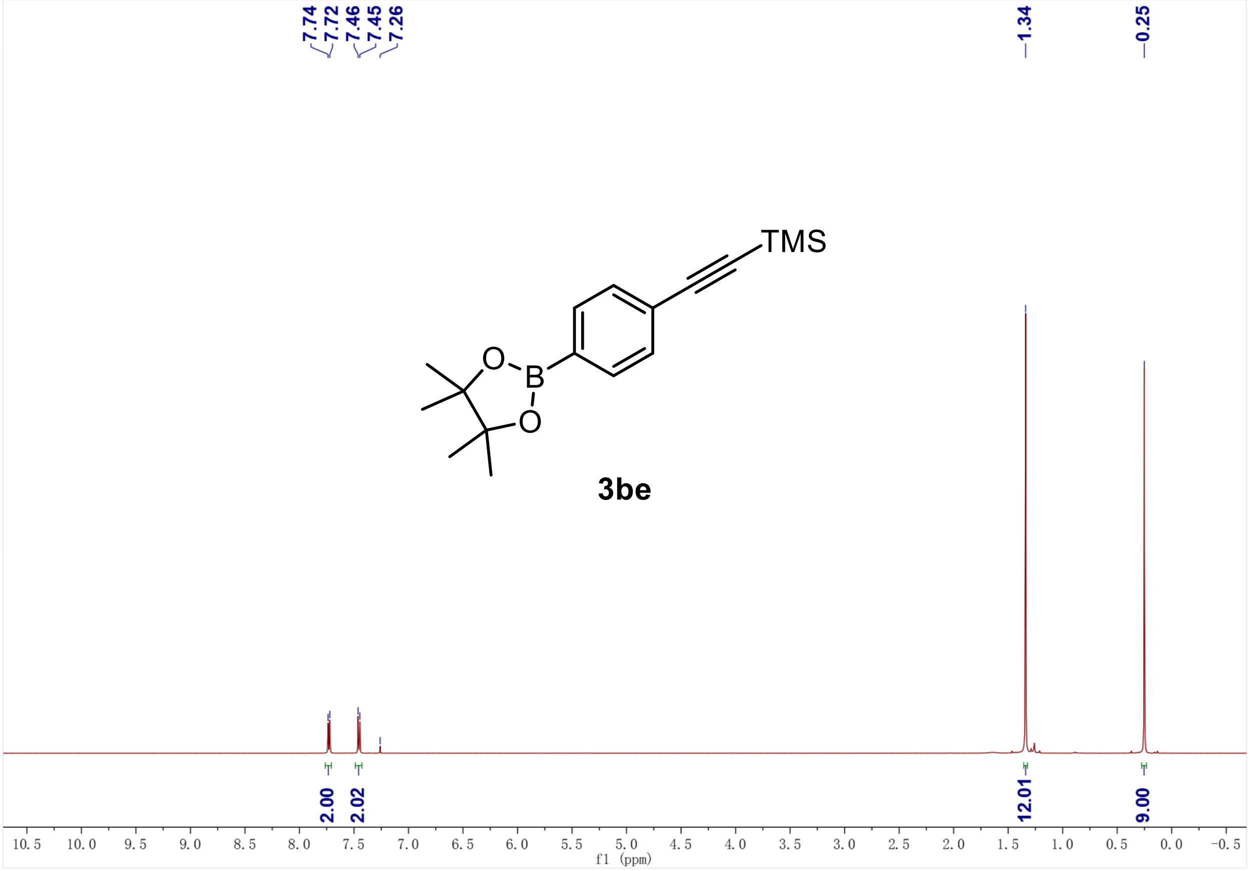

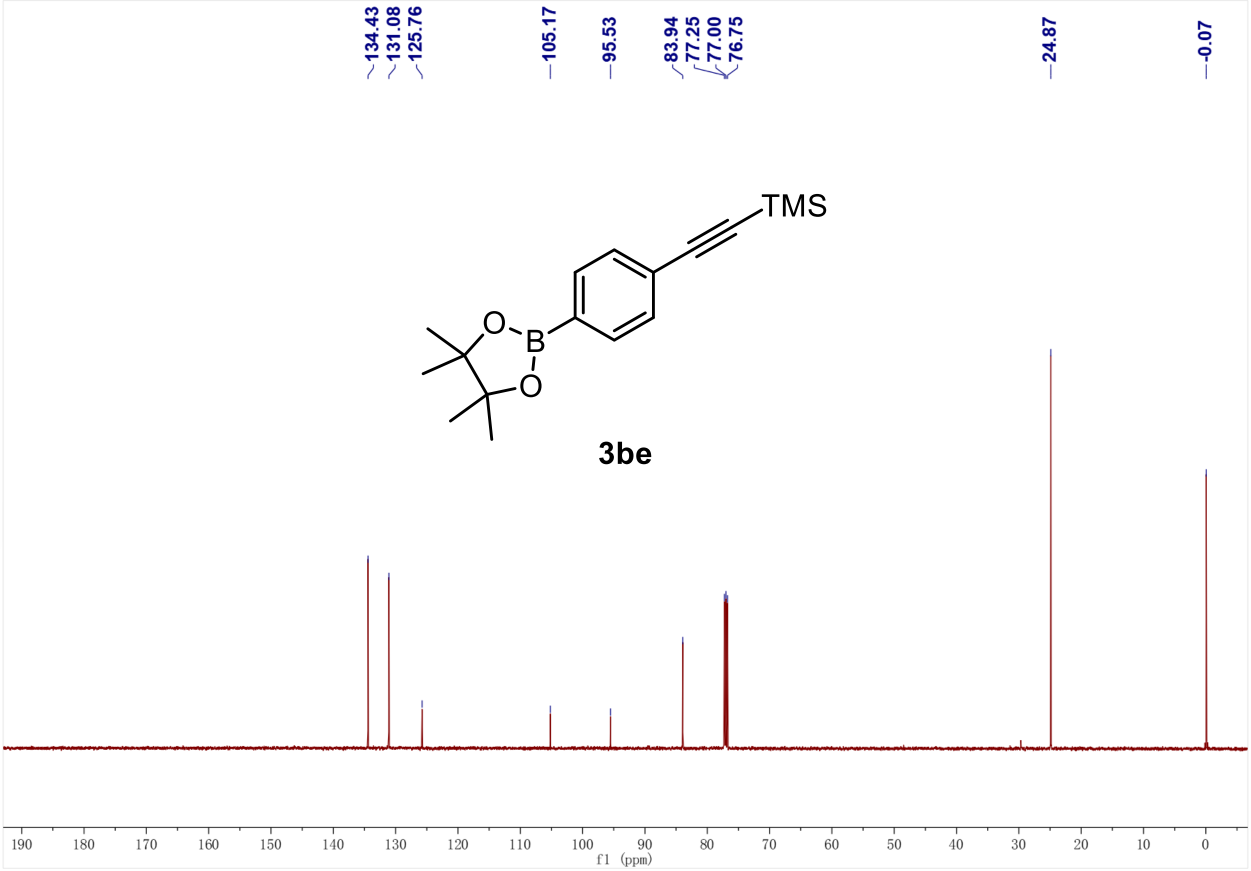

4a; ^{1}H NMR (500 MHz, $CDCl_3$); ^{13}C NMR (126 MHz, $CDCl_3$)

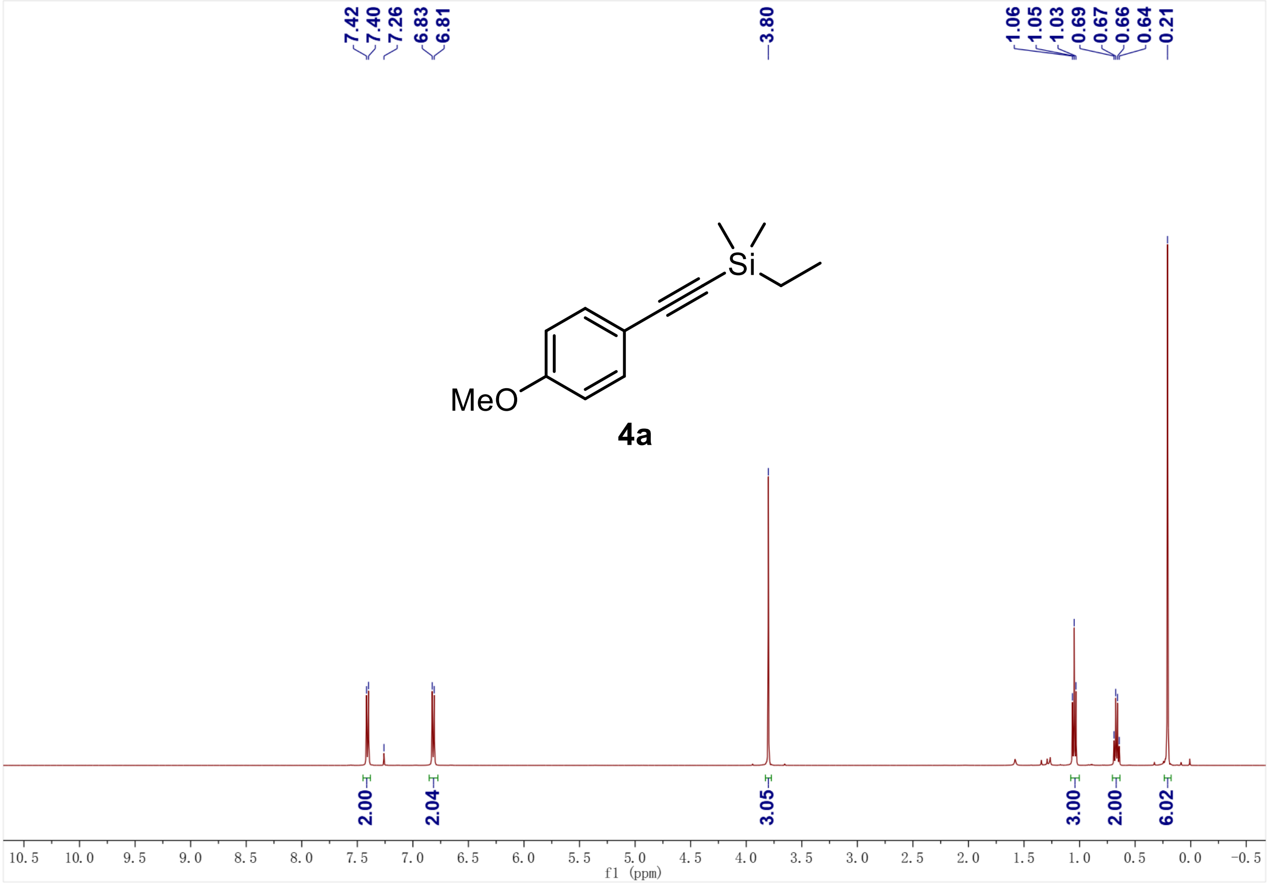

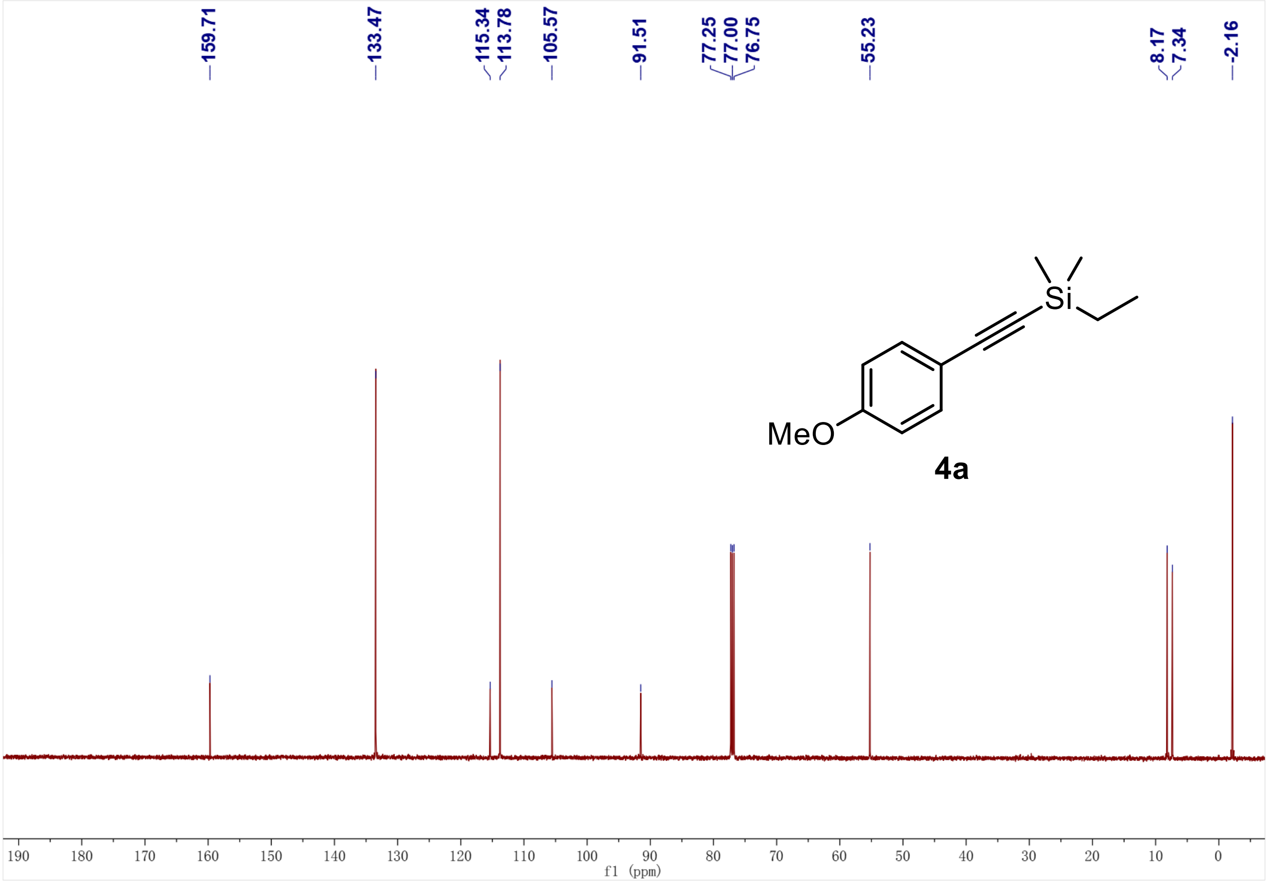

4b; ^{1}H NMR (500 MHz, $CDCl_3$); ^{13}C NMR (126 MHz, $CDCl_3$)

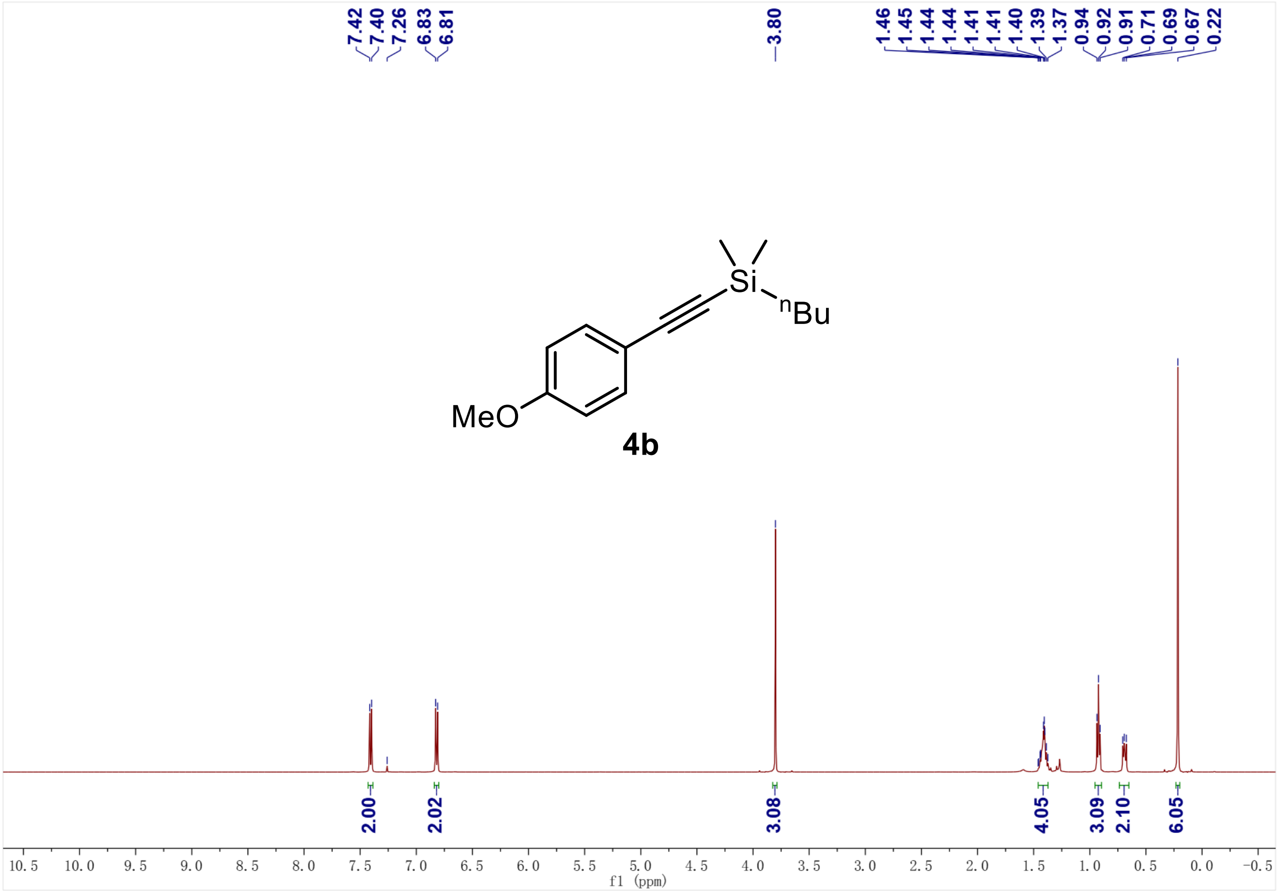

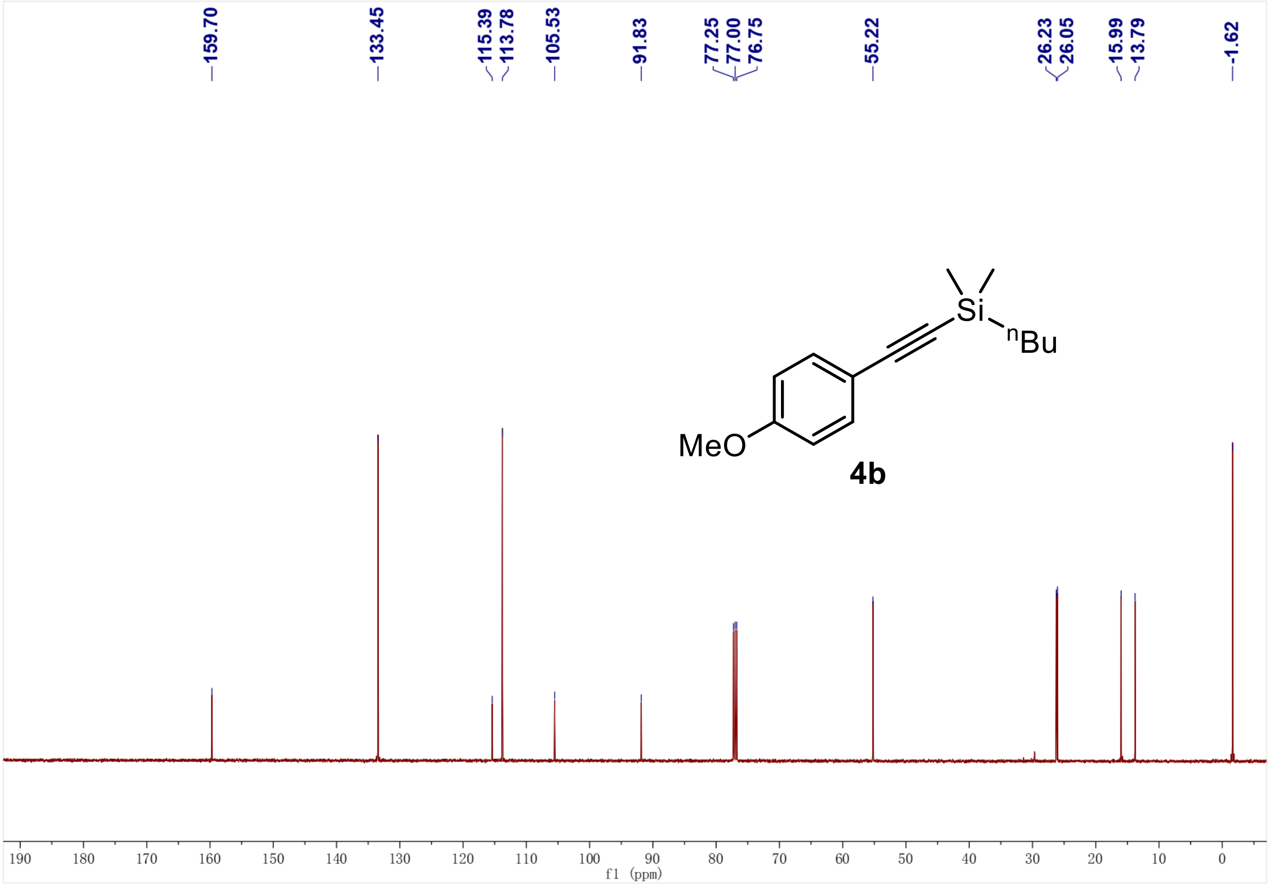

4c; ^{1}H NMR (500 MHz, $CDCl_3$); ^{13}C NMR (126 MHz, $CDCl_3$)

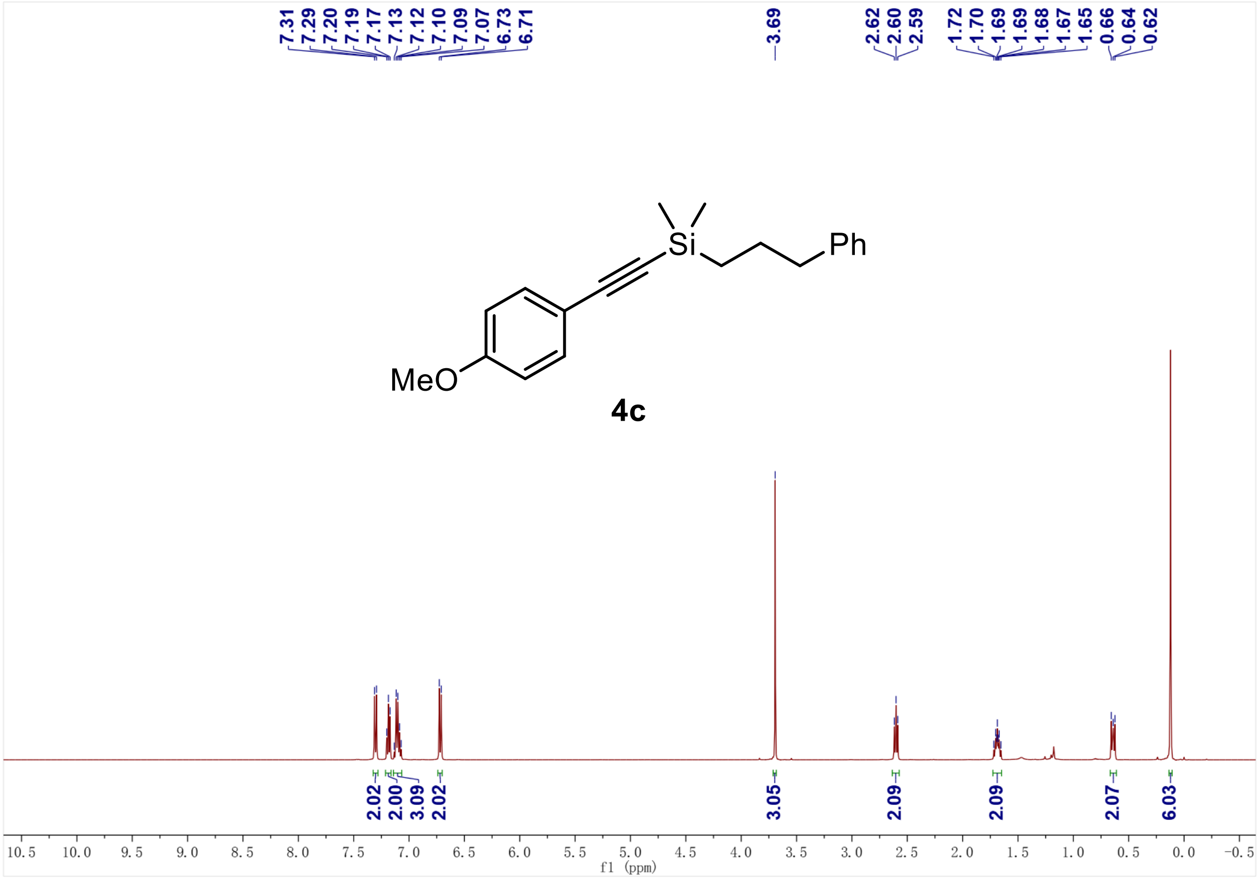

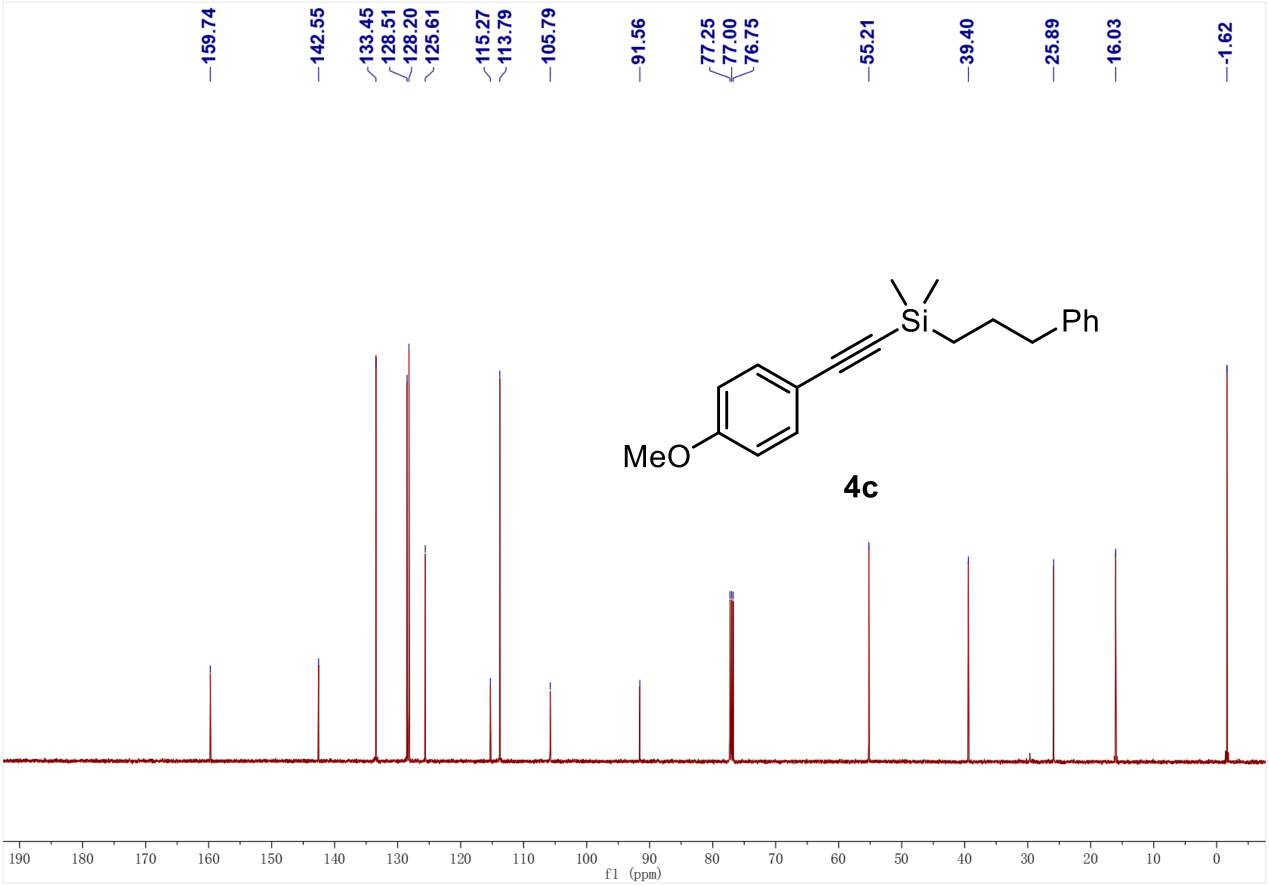

4d; ^{1}H NMR (500 MHz, $CDCl_3$); ^{13}C NMR (126 MHz, $CDCl_3$)

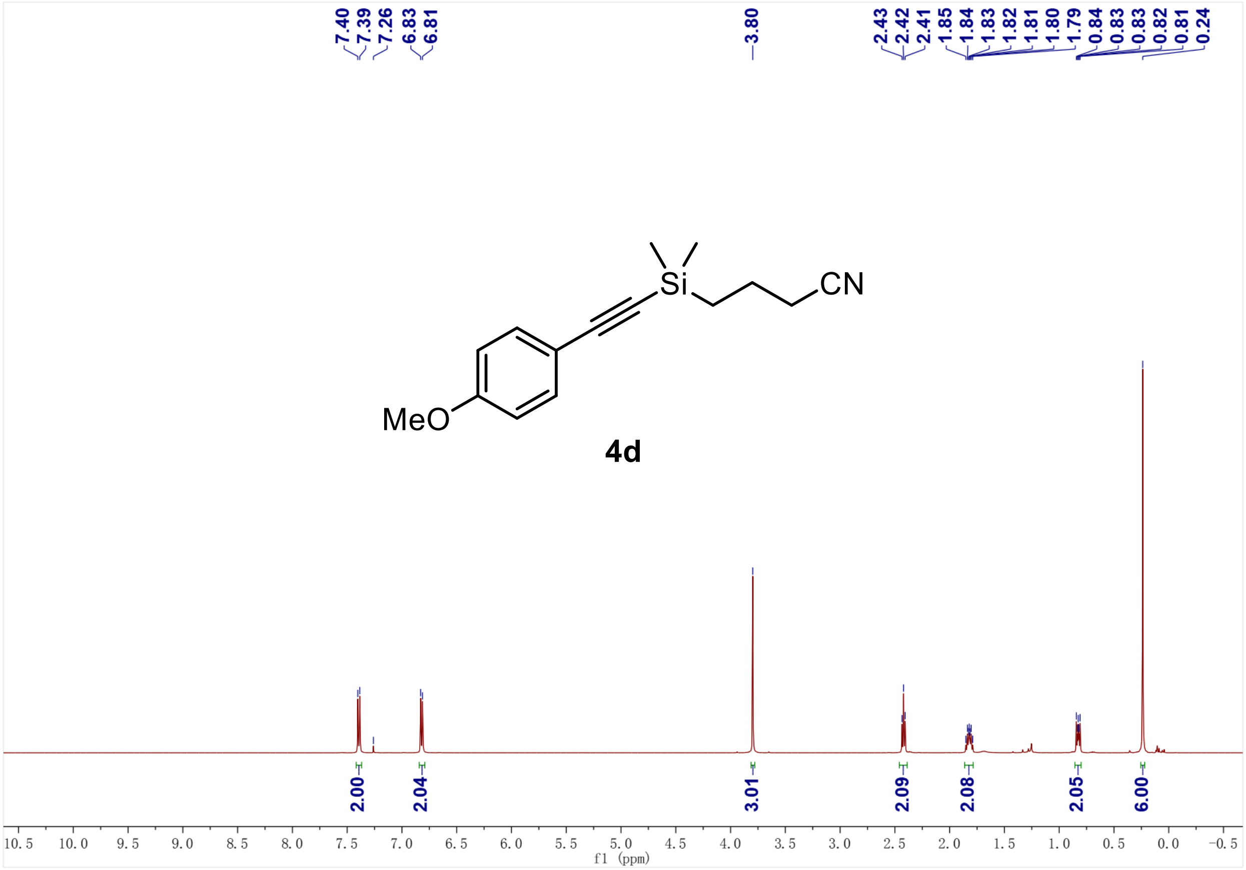

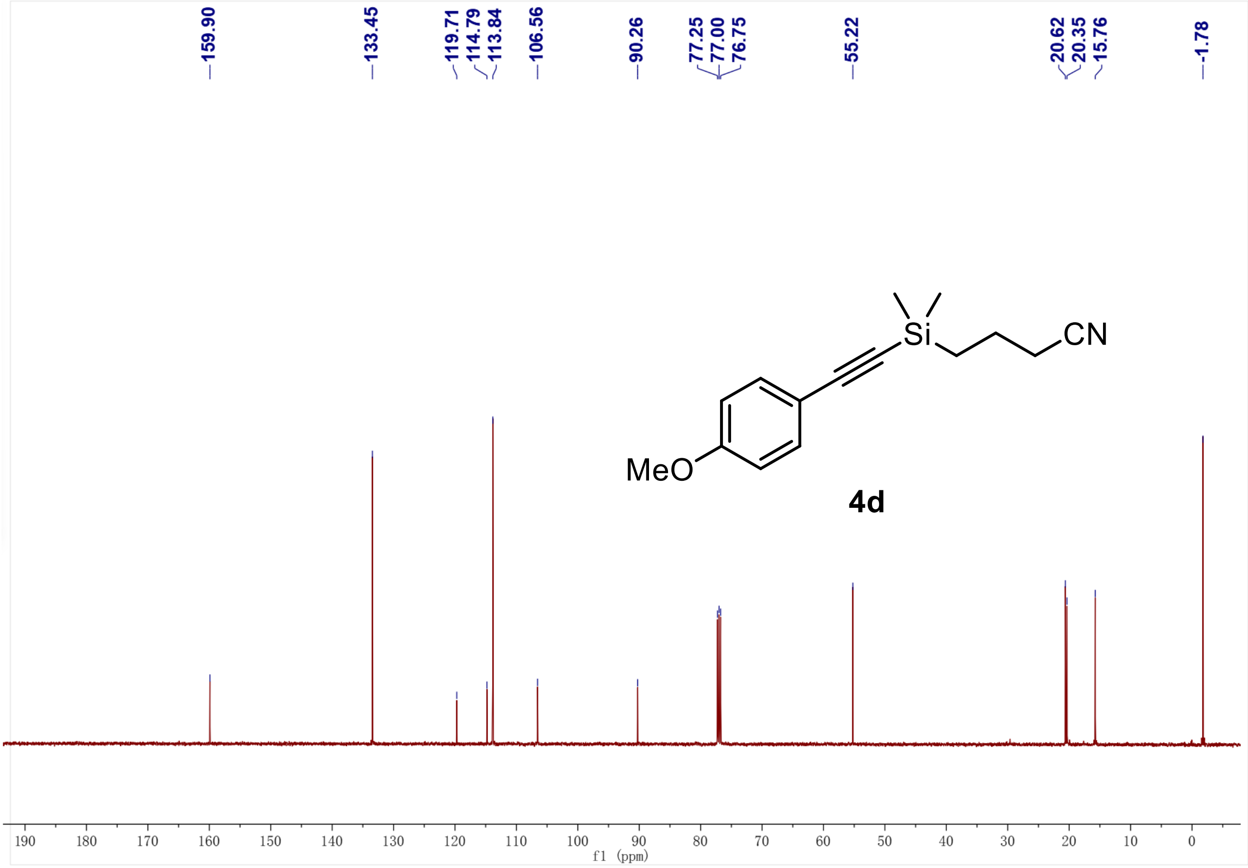

4e; ^{1}H NMR (500 MHz, $CDCl_3$); ^{13}C NMR (126 MHz, $CDCl_3$)

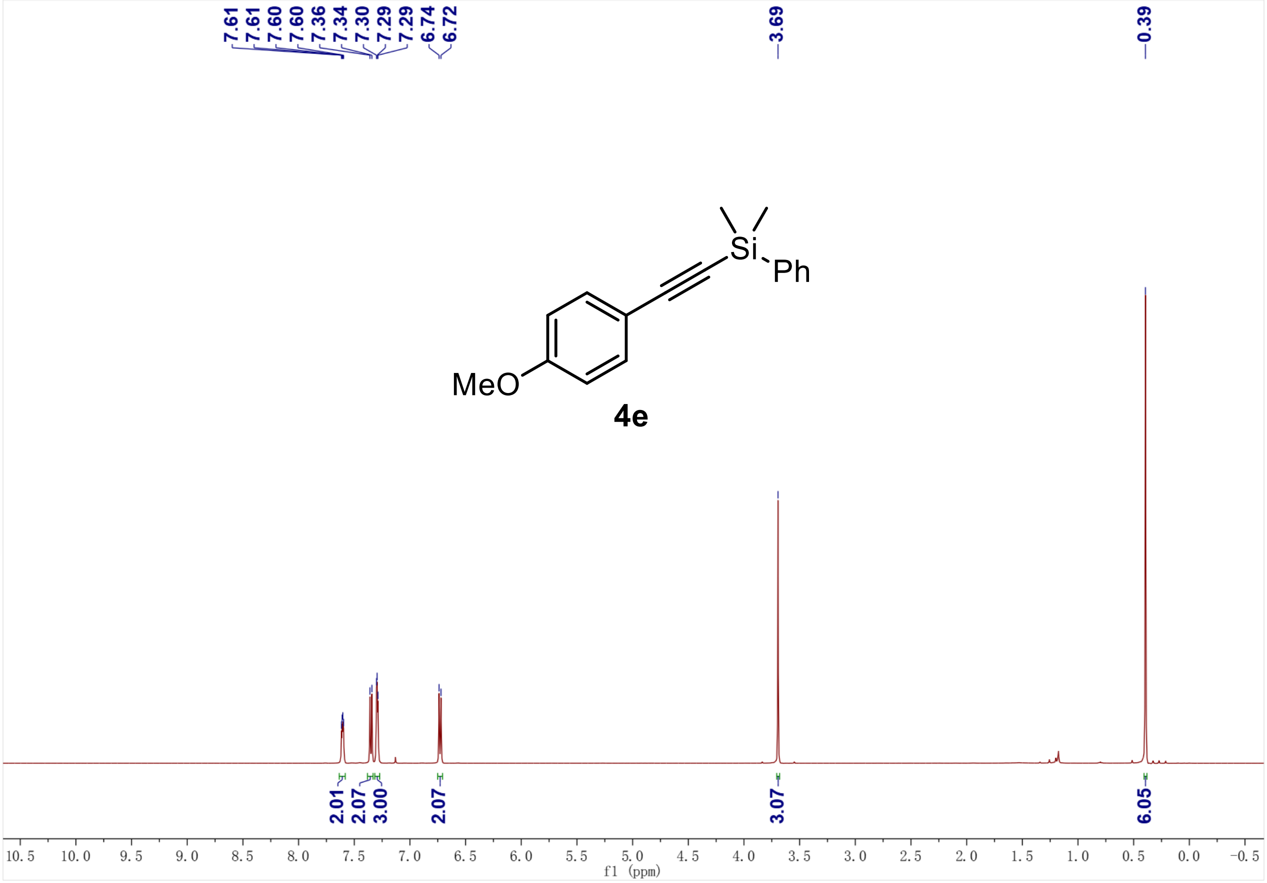

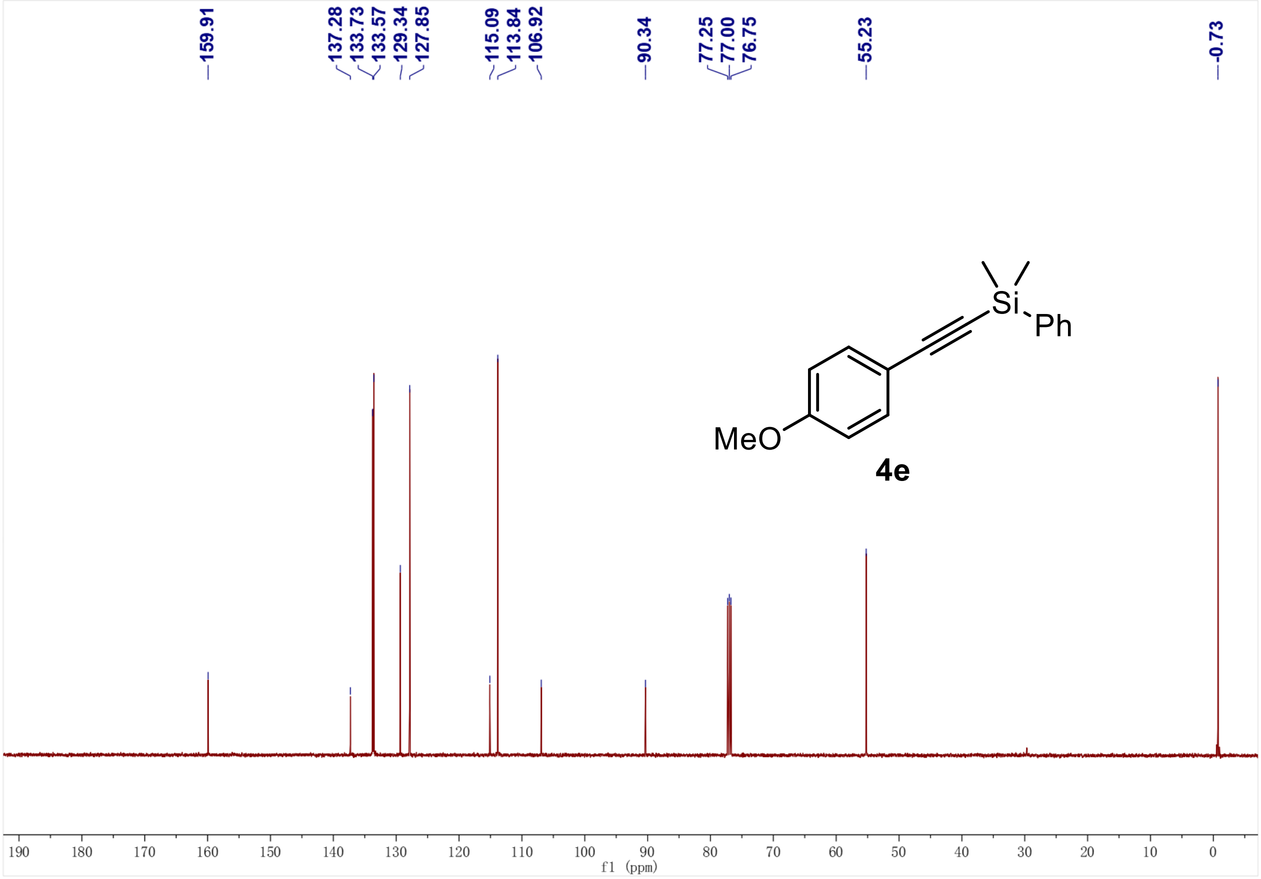

4f; ^{1}H NMR (500 MHz, $CDCl_3$); ^{13}C NMR (126 MHz, $CDCl_3$)

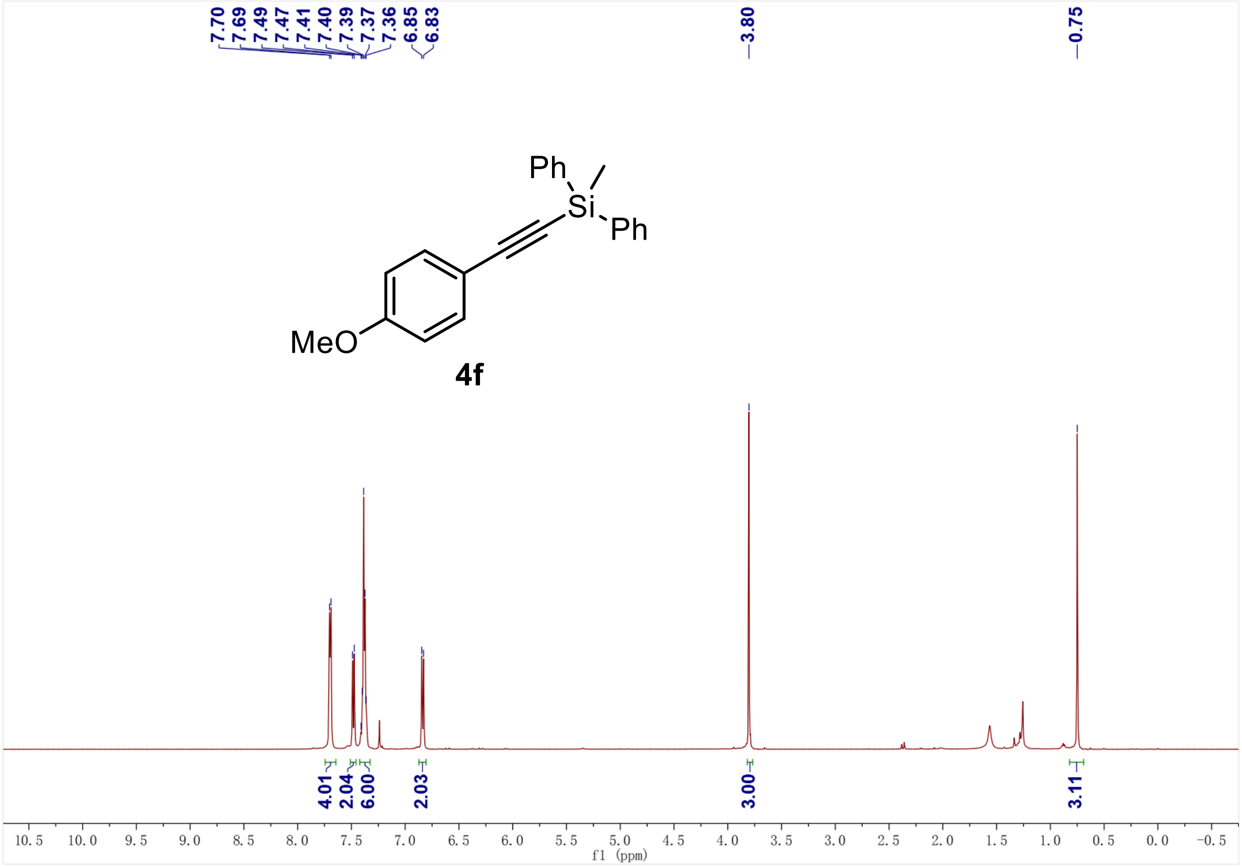

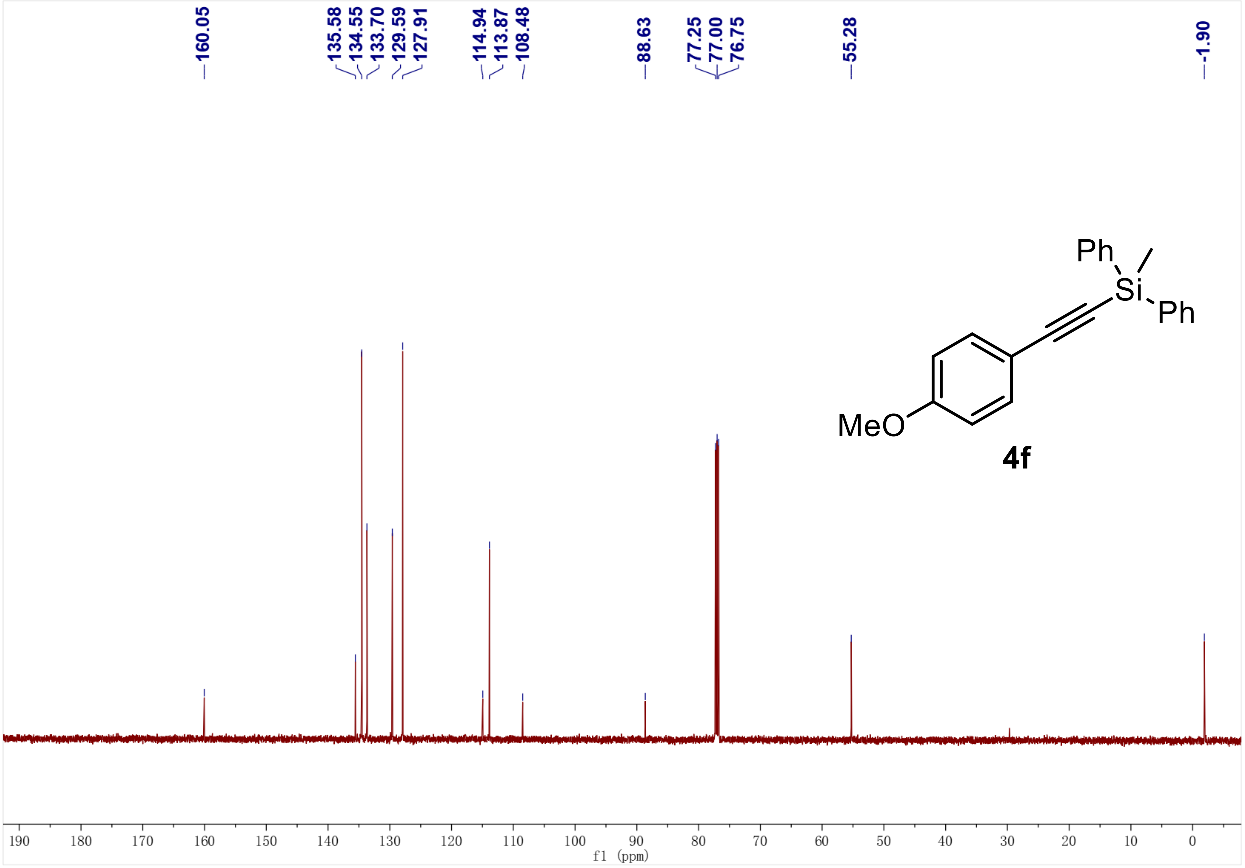

4g; ^{1}H NMR (500 MHz, $CDCl_3$); ^{13}C NMR (126 MHz, $CDCl_3$)

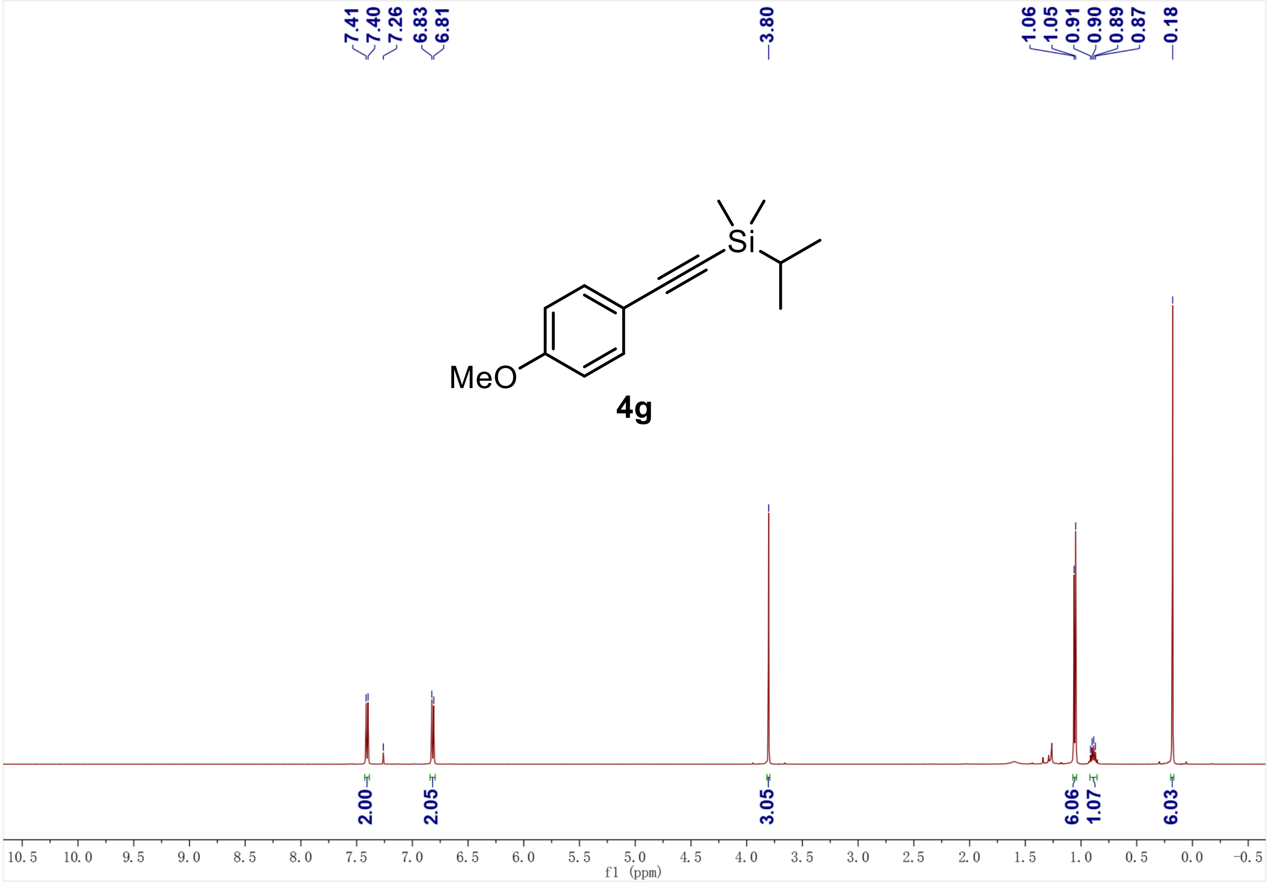

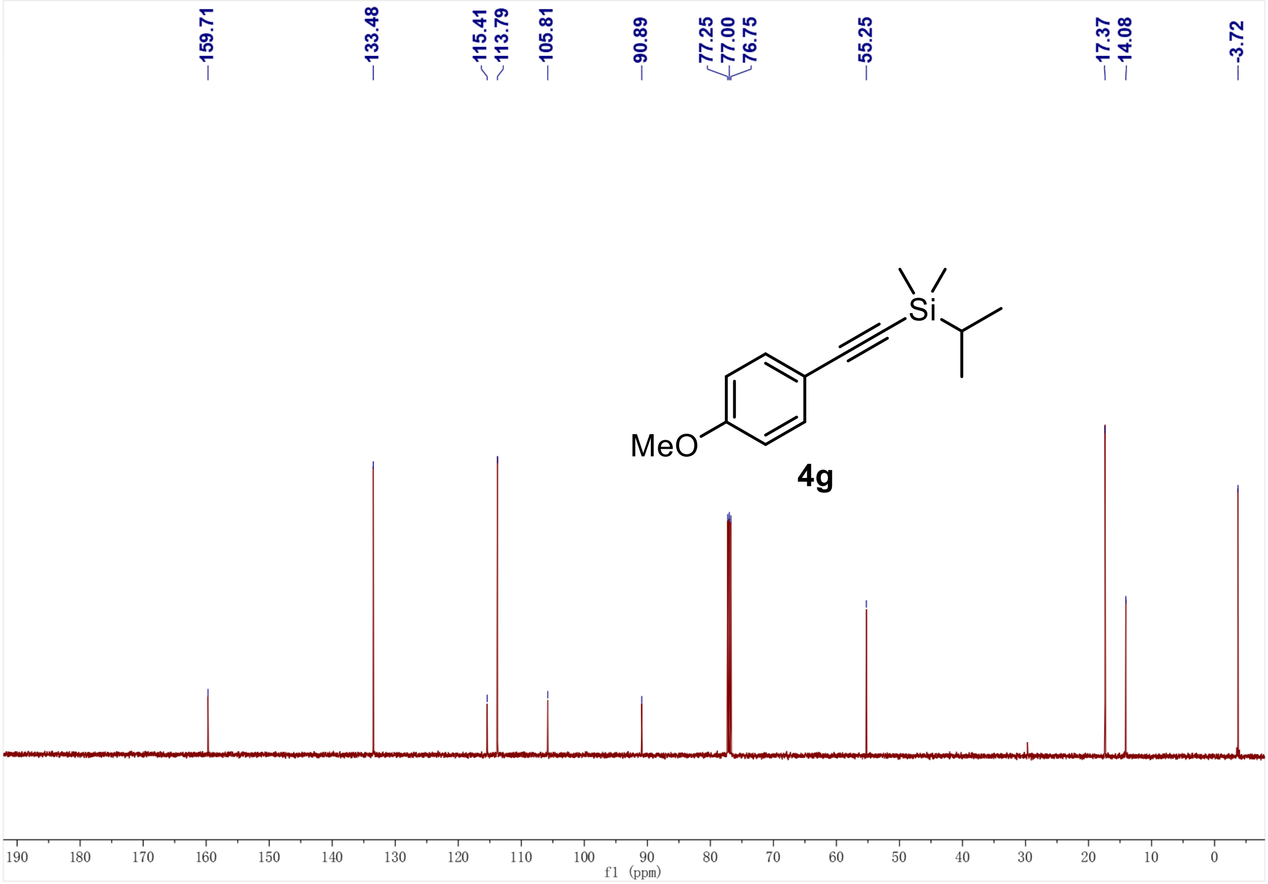

4h; ^{1}H NMR (500 MHz, $CDCl_3$); ^{13}C NMR (126 MHz, $CDCl_3$)

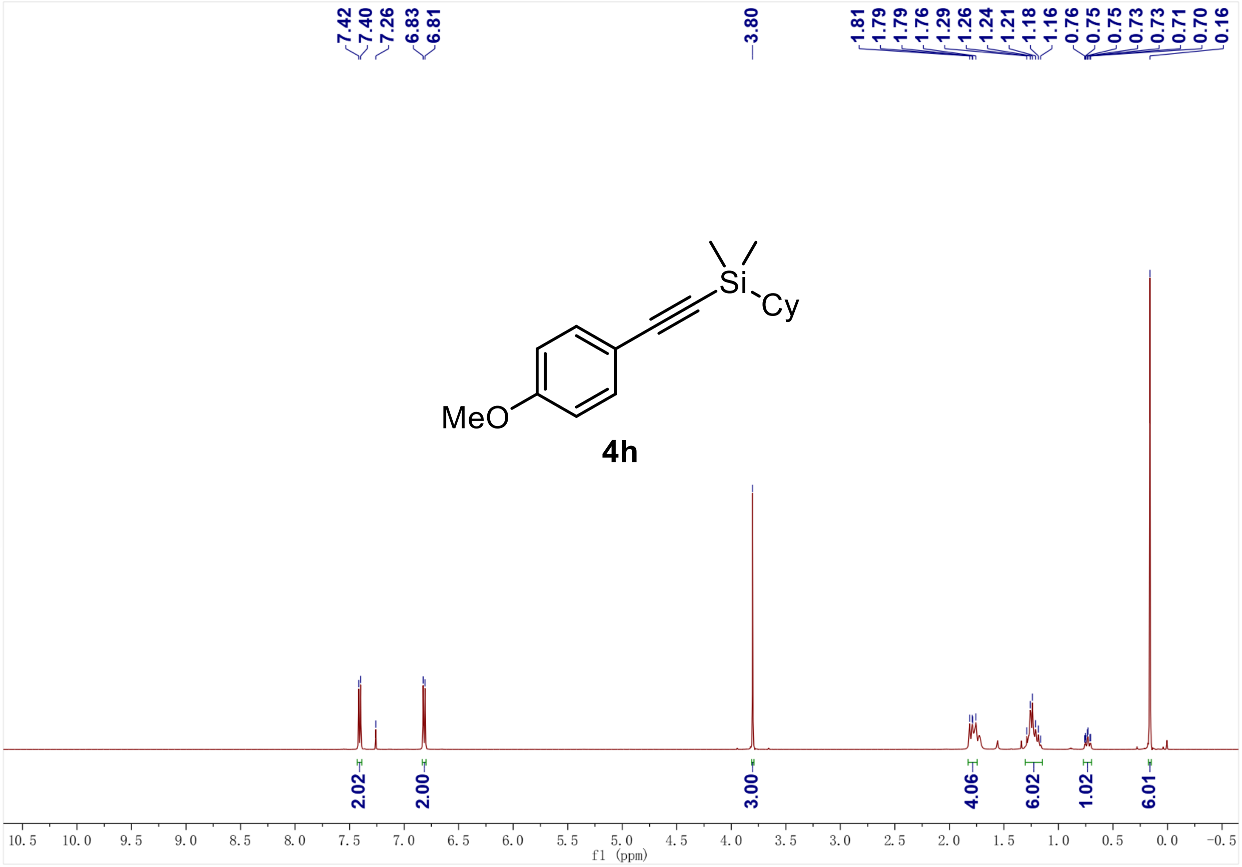

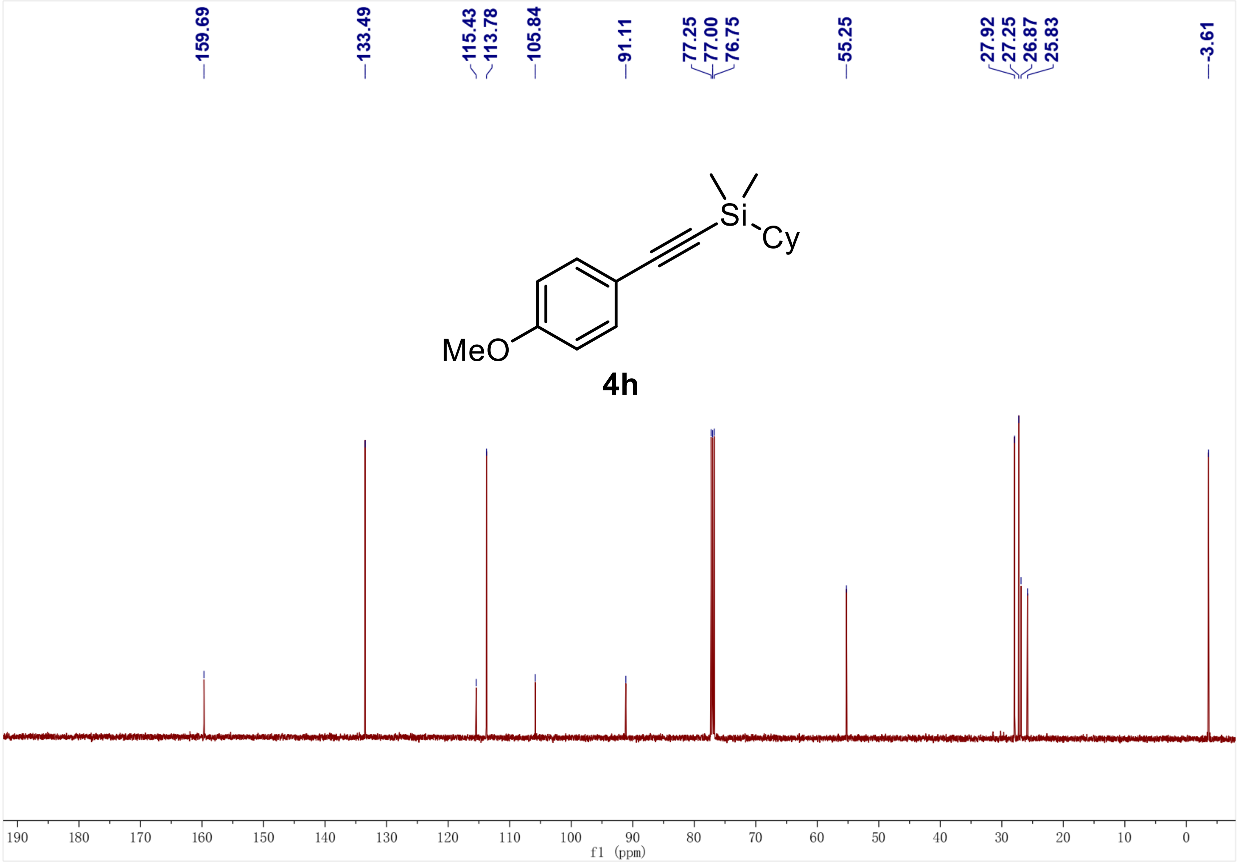

4i; ^{1}H NMR (500 MHz, $CDCl_3$); ^{13}C NMR (126 MHz, $CDCl_3$)

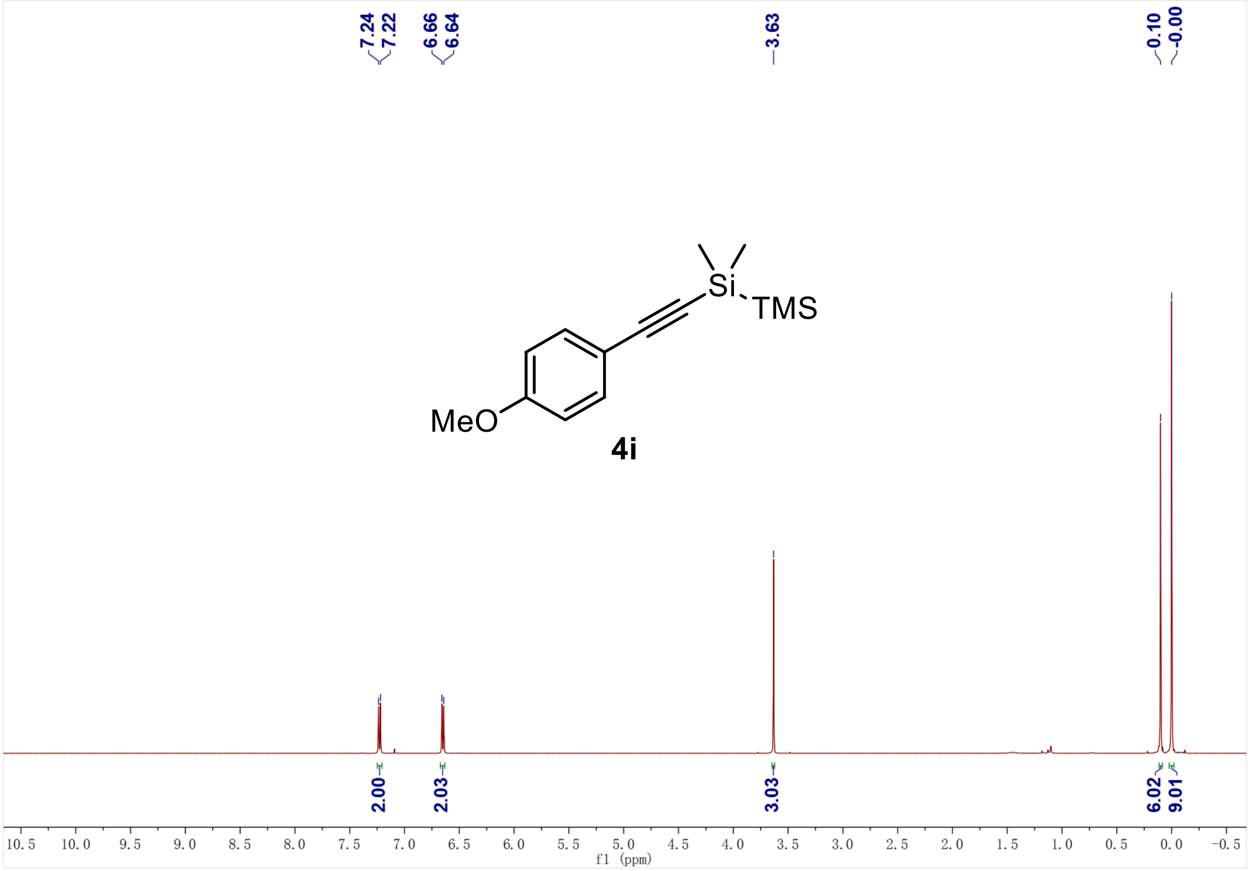

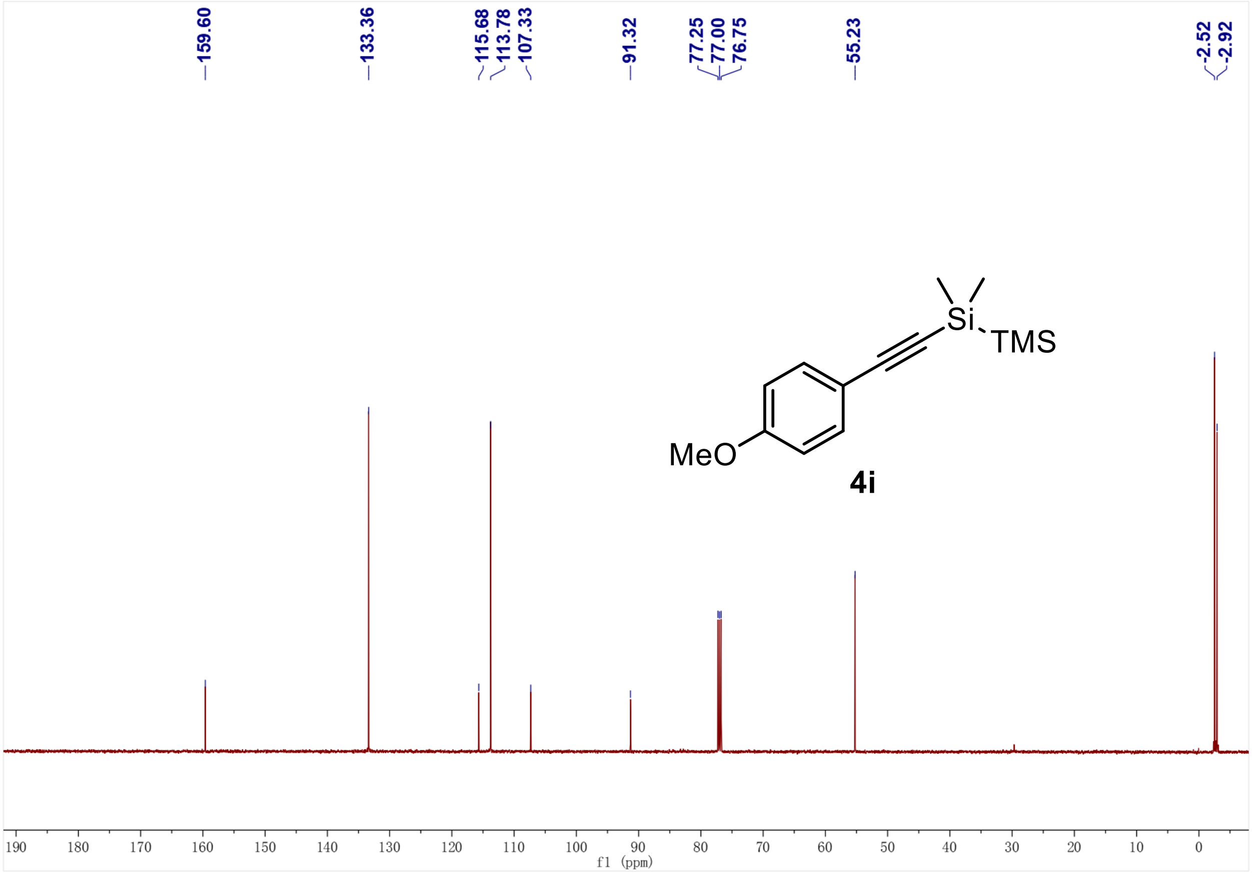

4j; ^{1}H NMR (500 MHz, $CDCl_3$); ^{13}C NMR (126 MHz, $CDCl_3$)

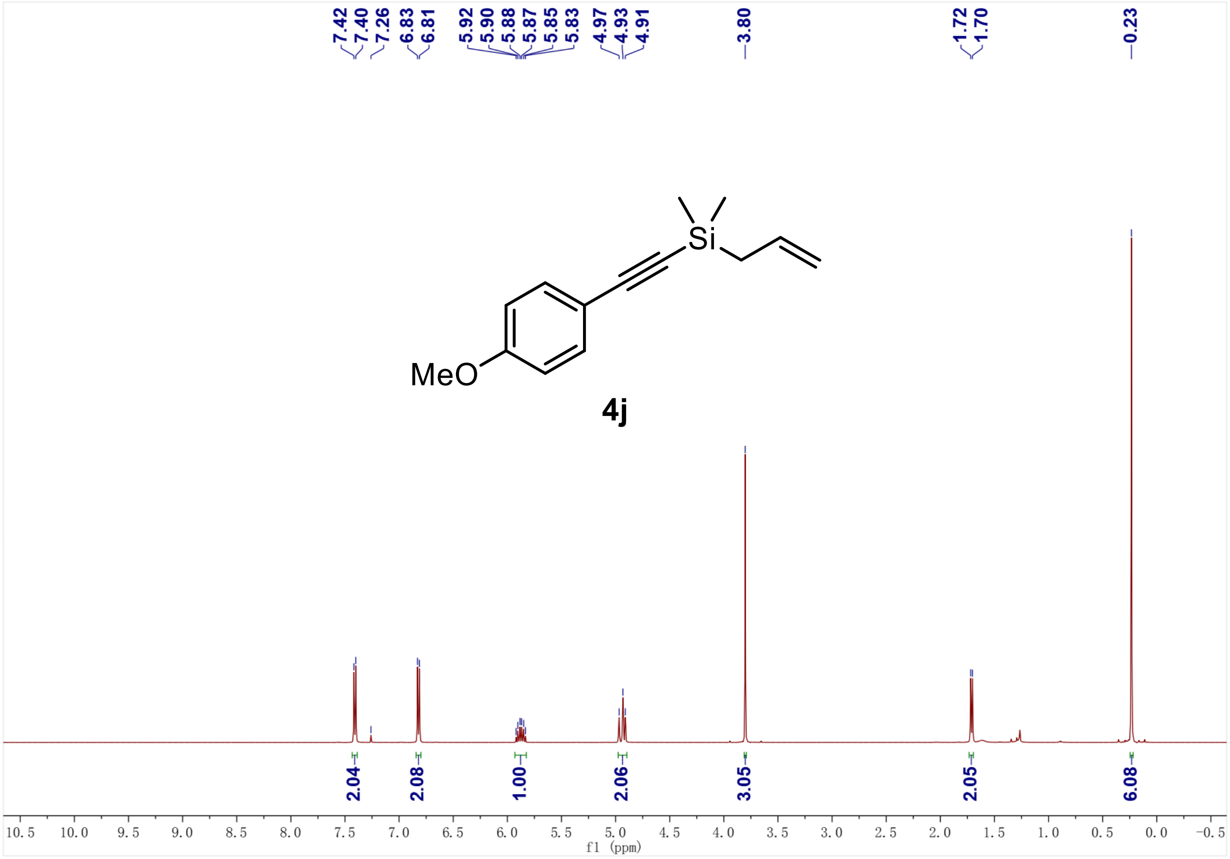

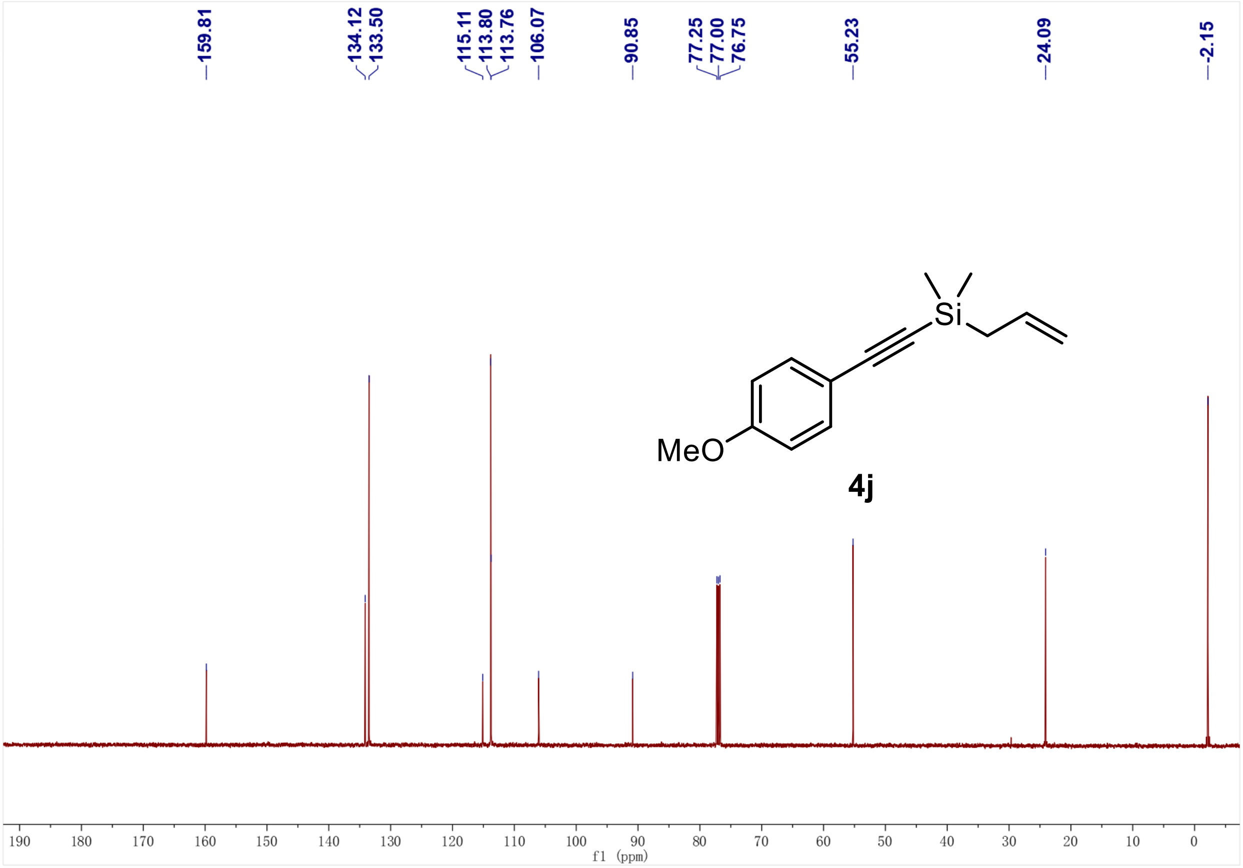

4k; ^{1}H NMR (500 MHz, $CDCl_3$); ^{13}C NMR (126 MHz, $CDCl_3$)

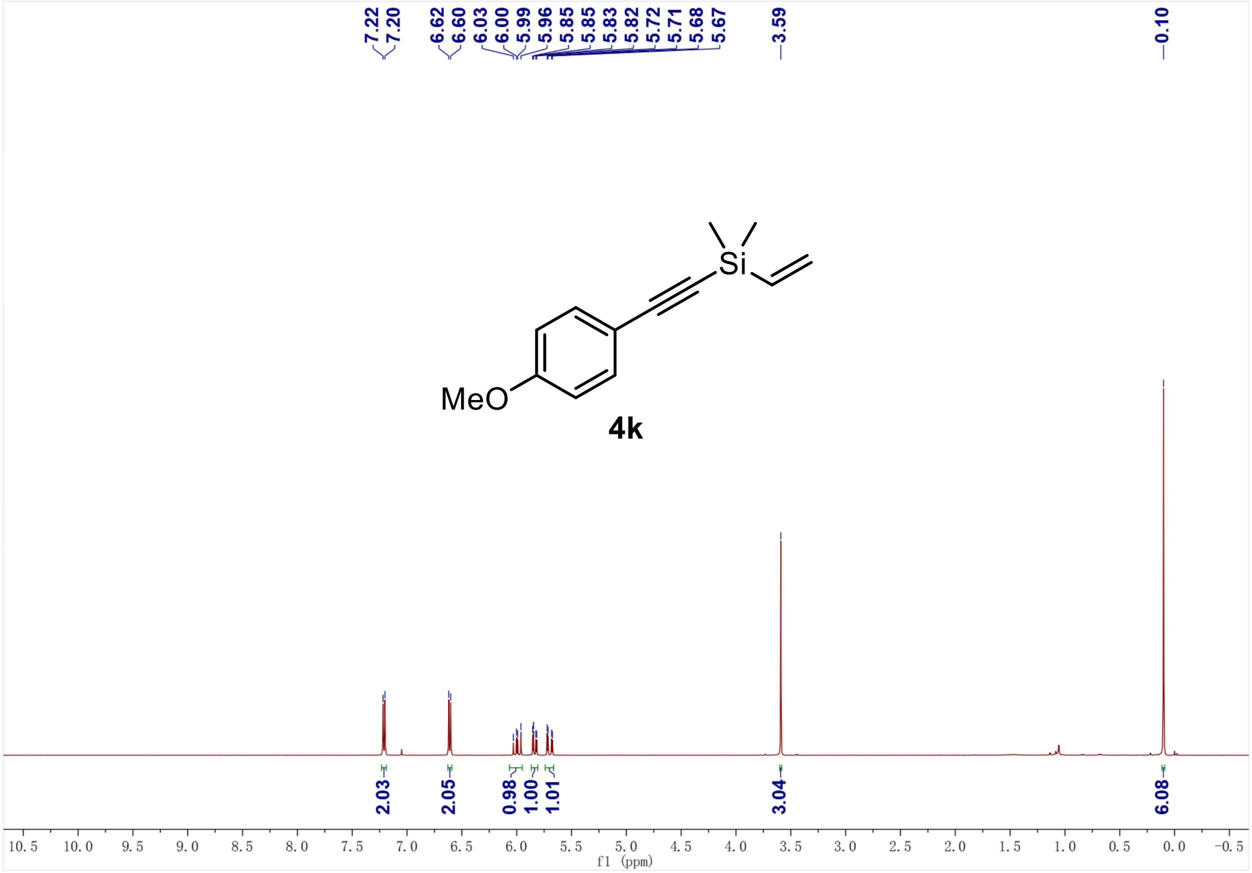

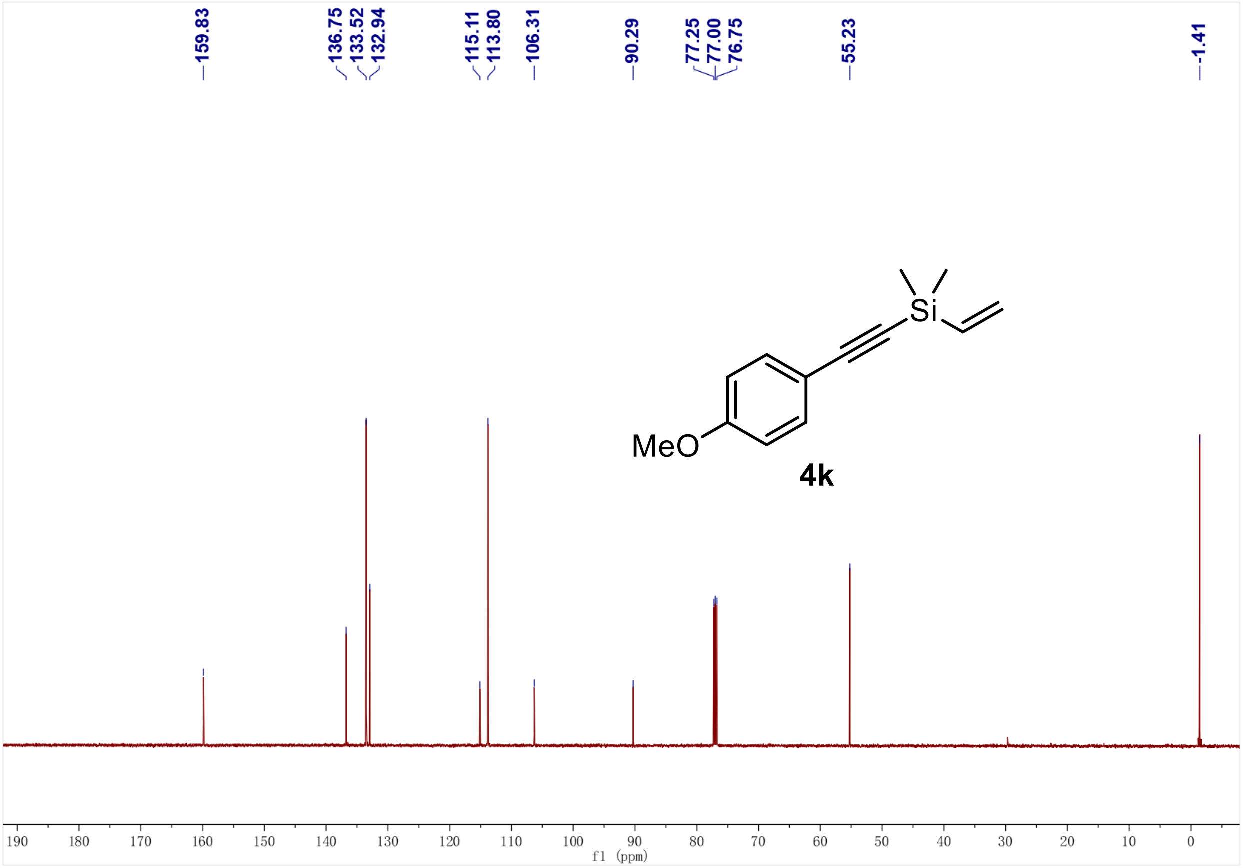

4l; ¹H NMR (500 MHz, CDCl$_3$); ¹³C NMR (126 MHz, CDCl$_3$)

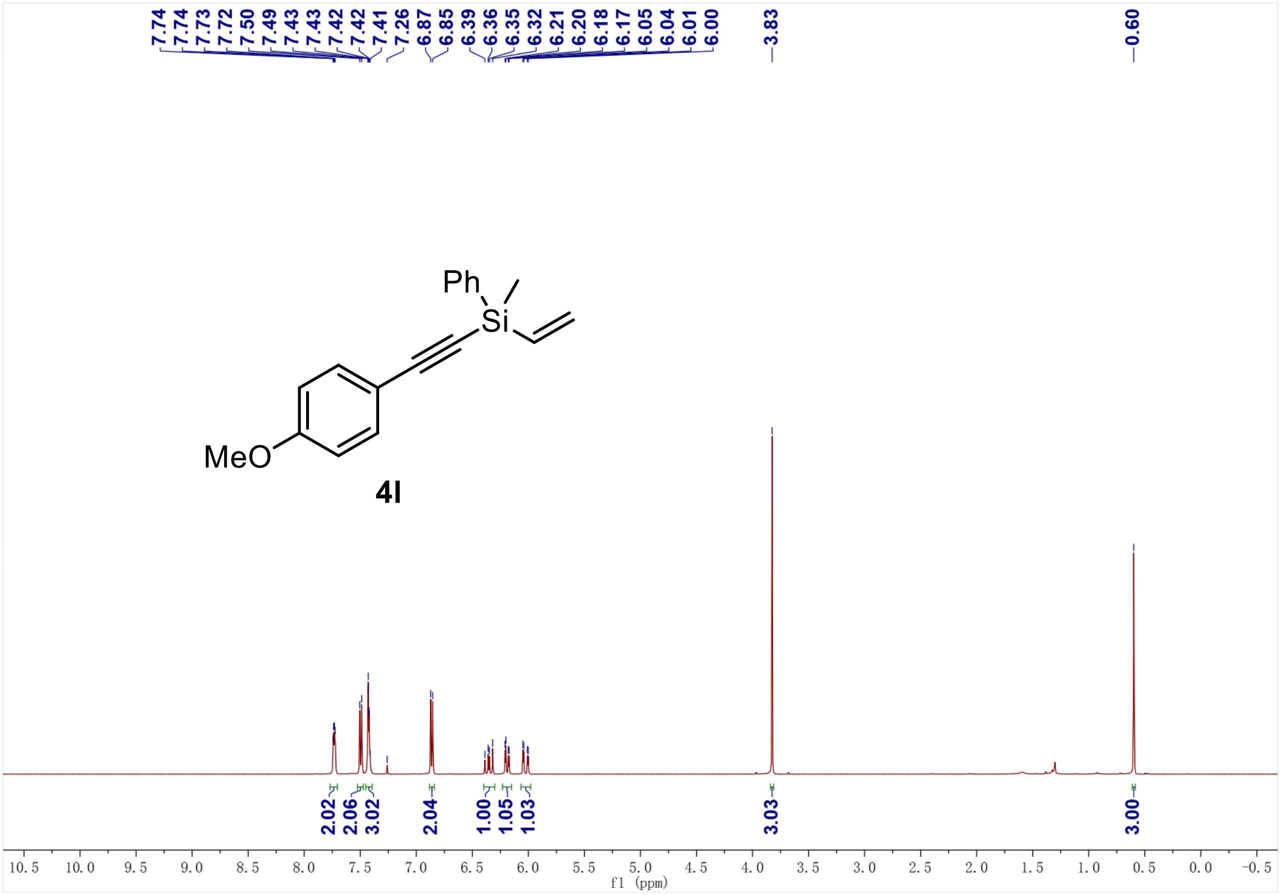

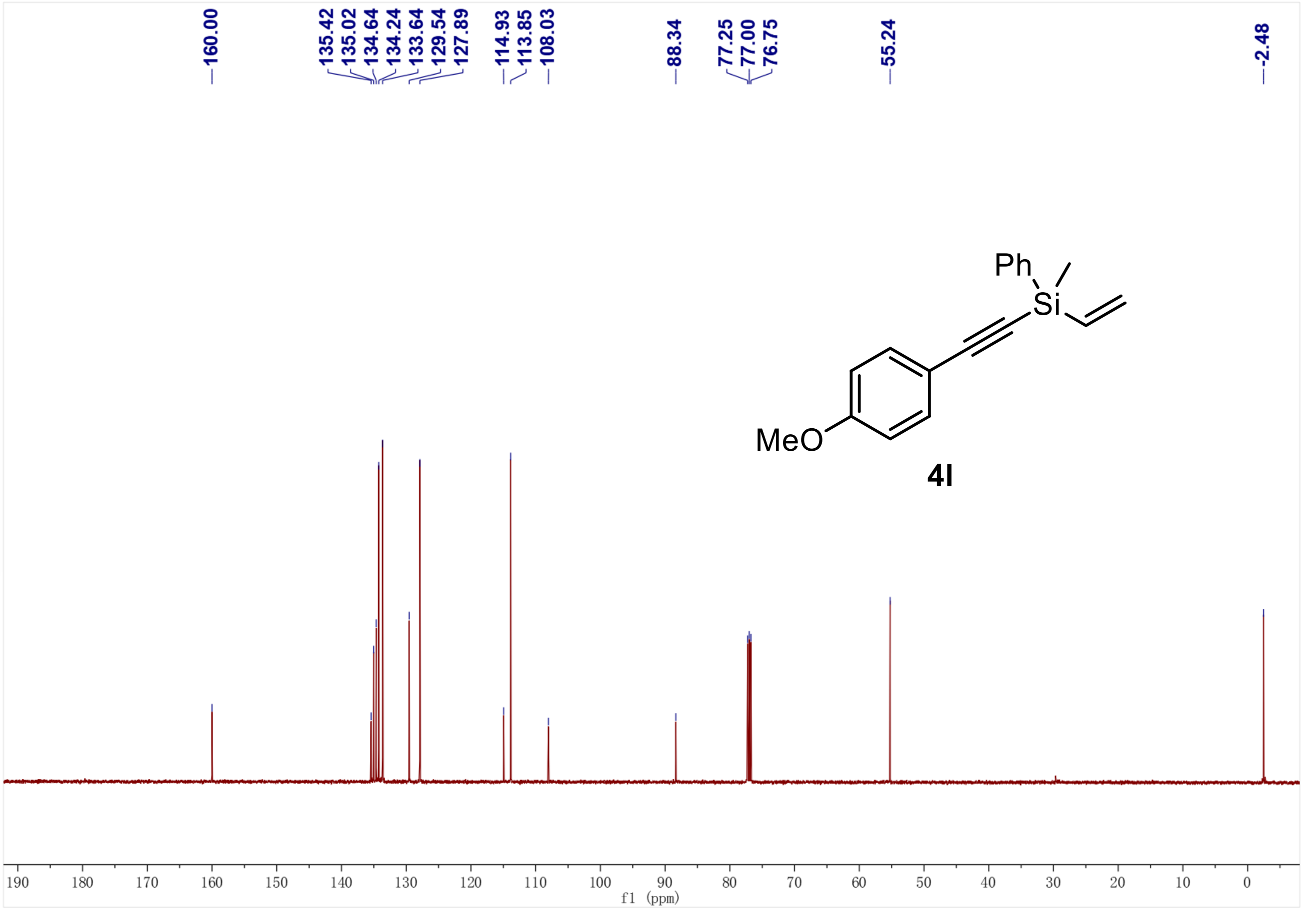

4m; ^{1}H NMR (500 MHz, $CDCl_3$); ^{13}C NMR (126 MHz, $CDCl_3$)

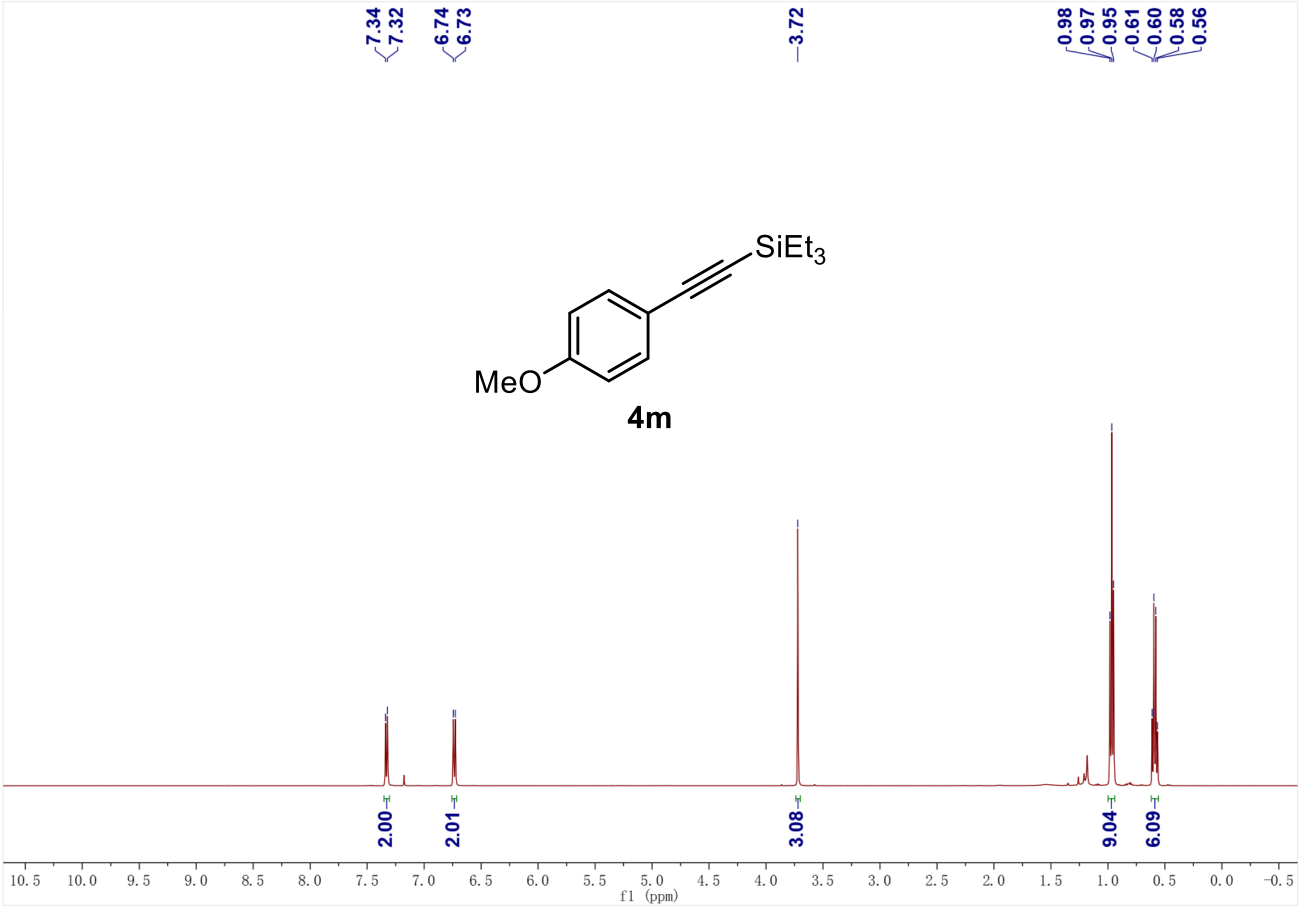

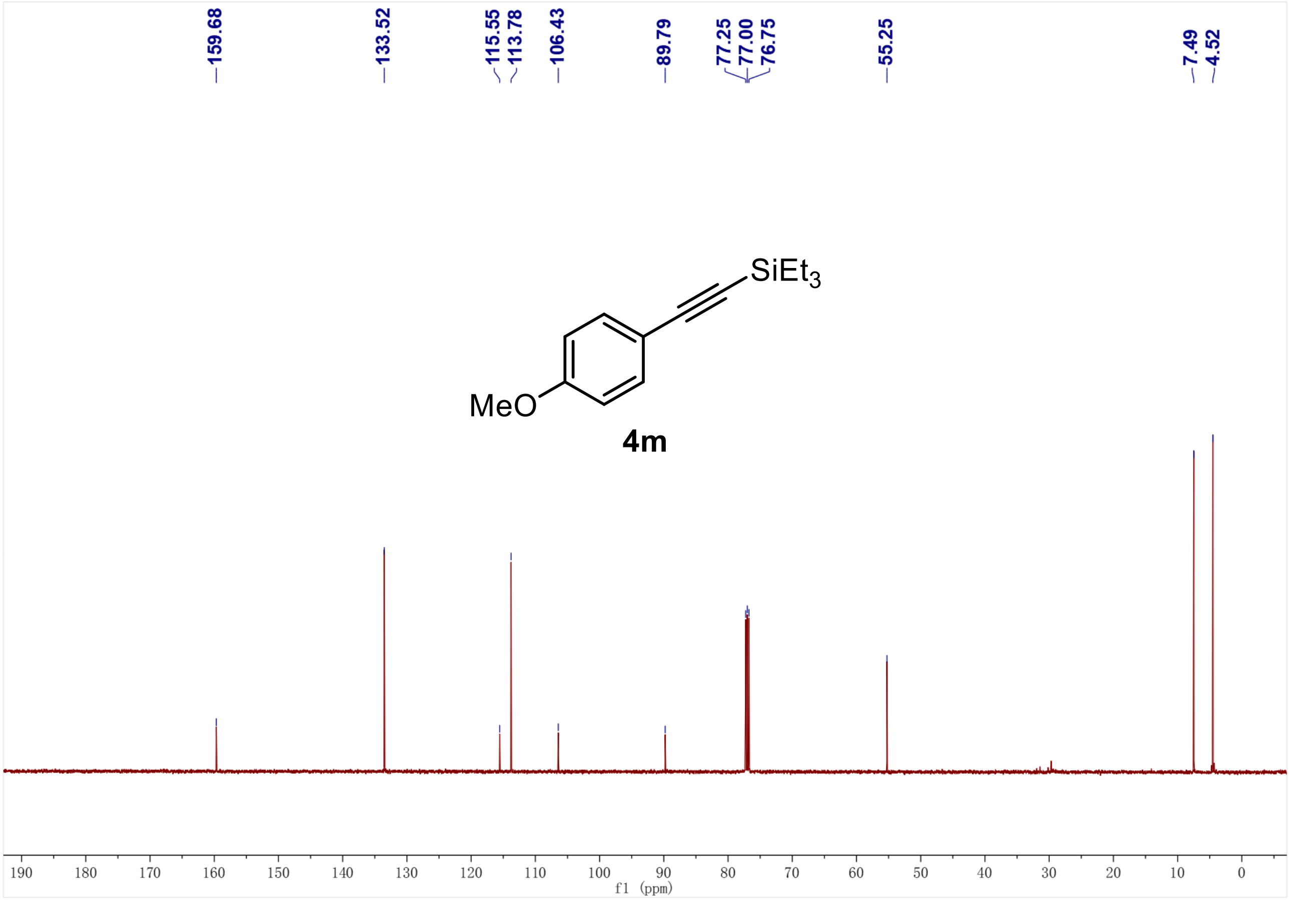

4n; ^{1}H NMR (500 MHz, $CDCl_3$); ^{13}C NMR (126 MHz, $CDCl_3$)

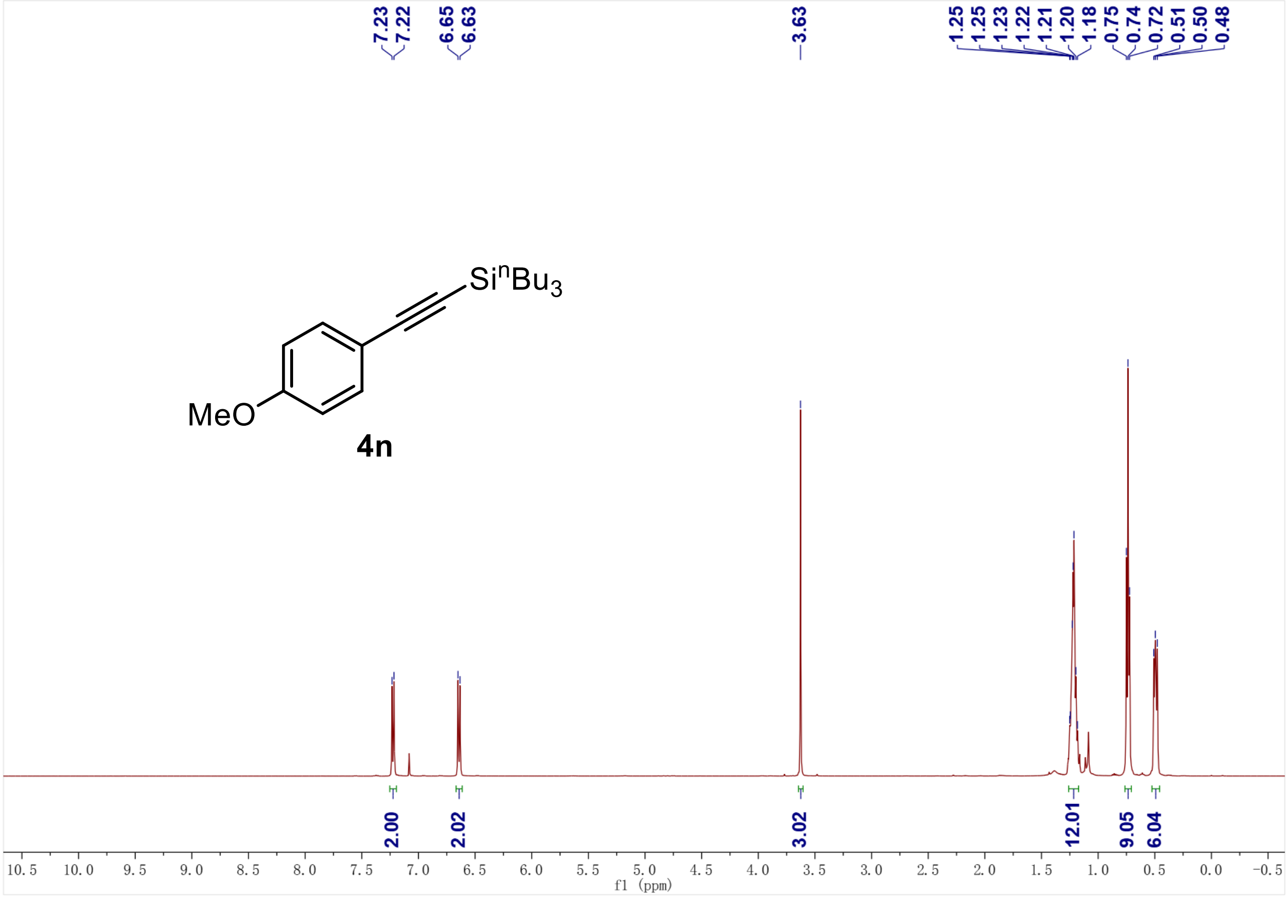

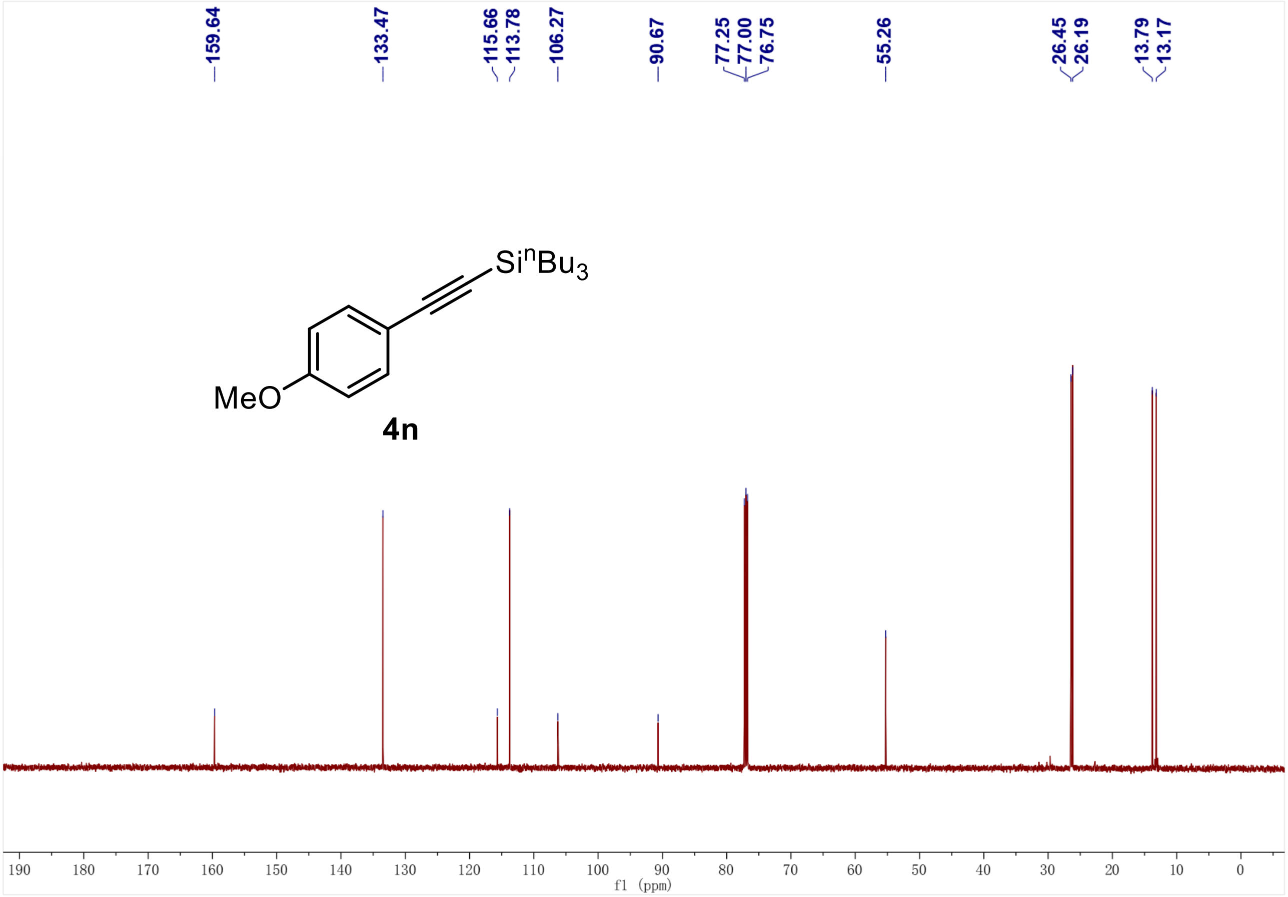

4o; ^{1}H NMR (400 MHz, $CDCl_3$); ^{13}C NMR (126 MHz, $CDCl_3$)

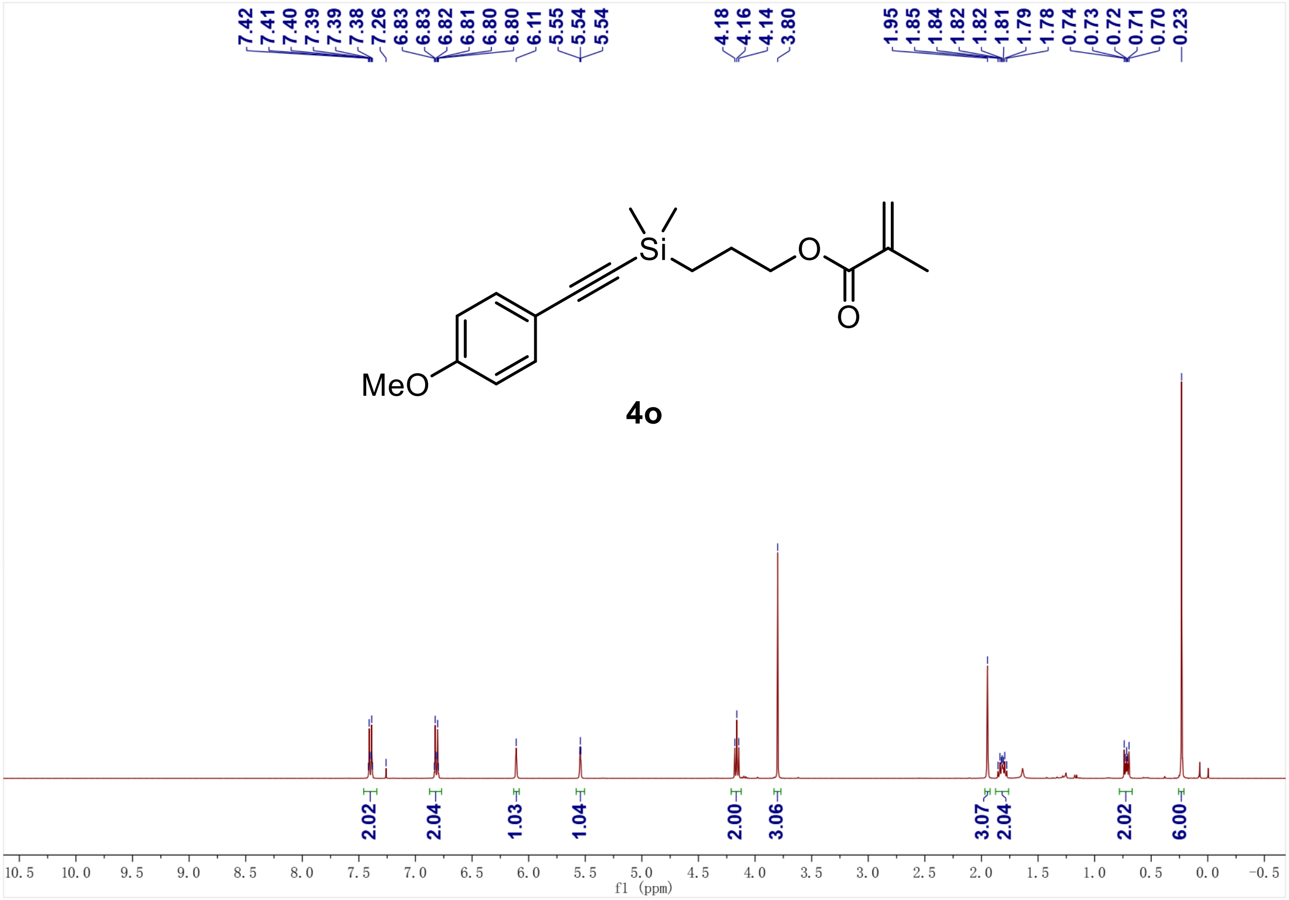

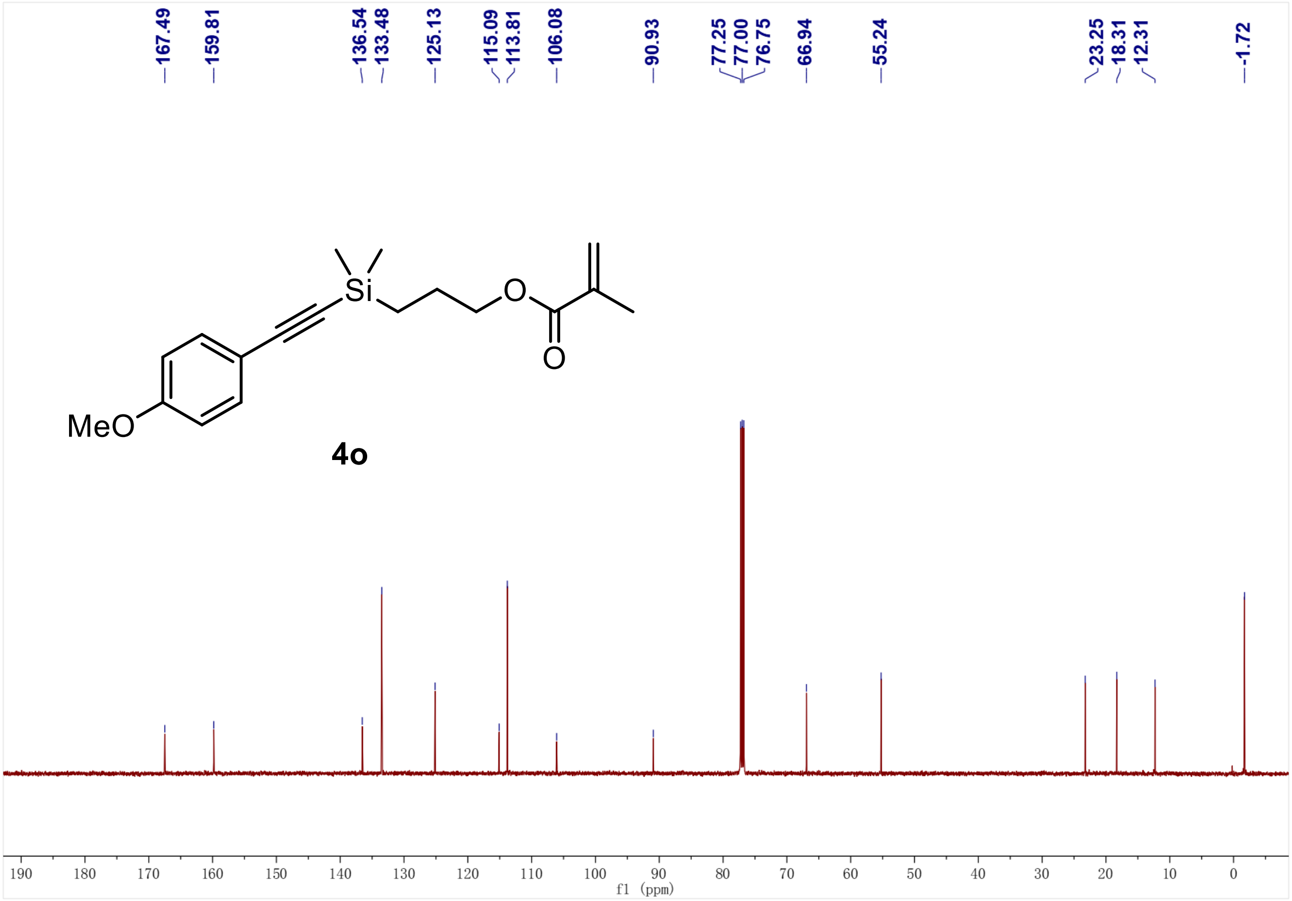

5a; ^{1}H NMR (400 MHz, $CDCl_3$); ^{13}C NMR (126 MHz, $CDCl_3$)

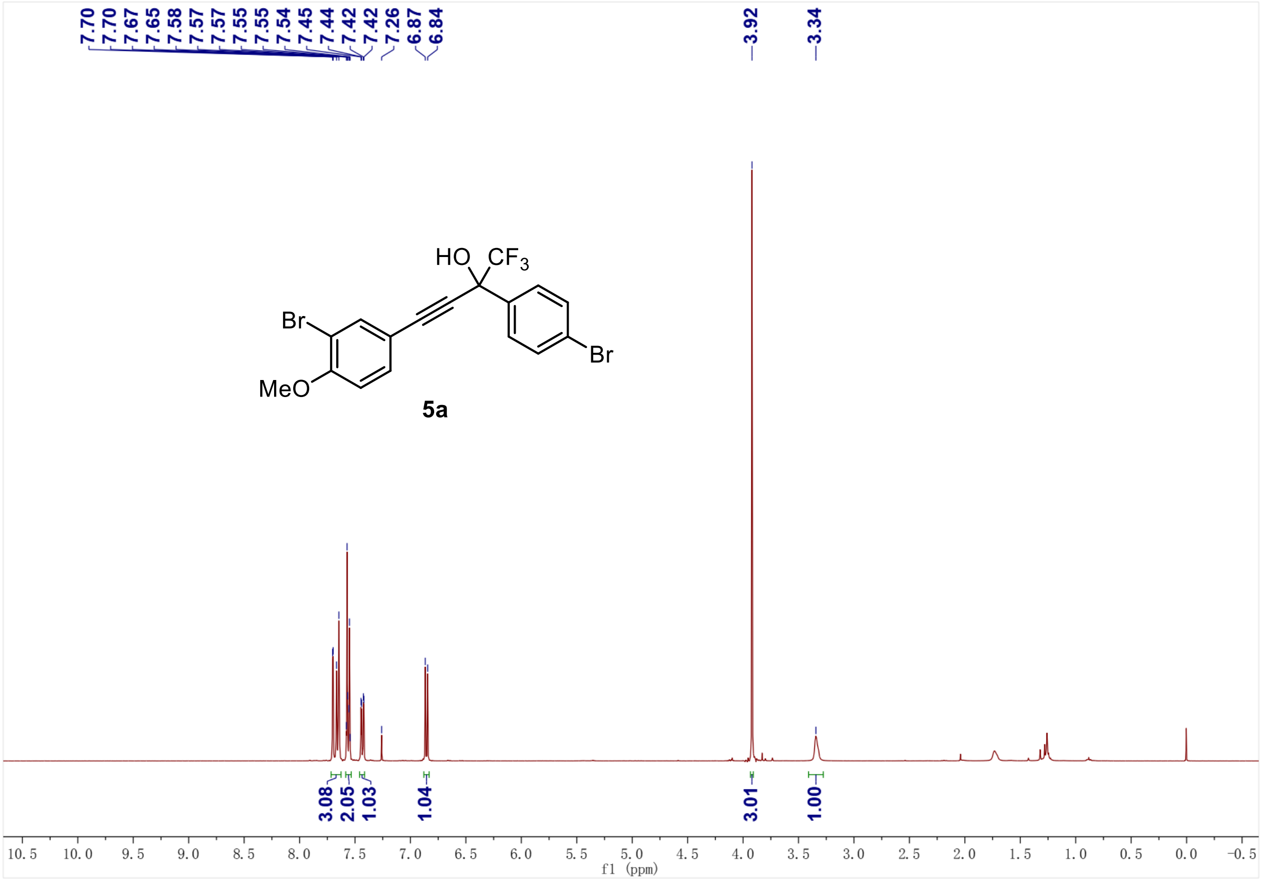

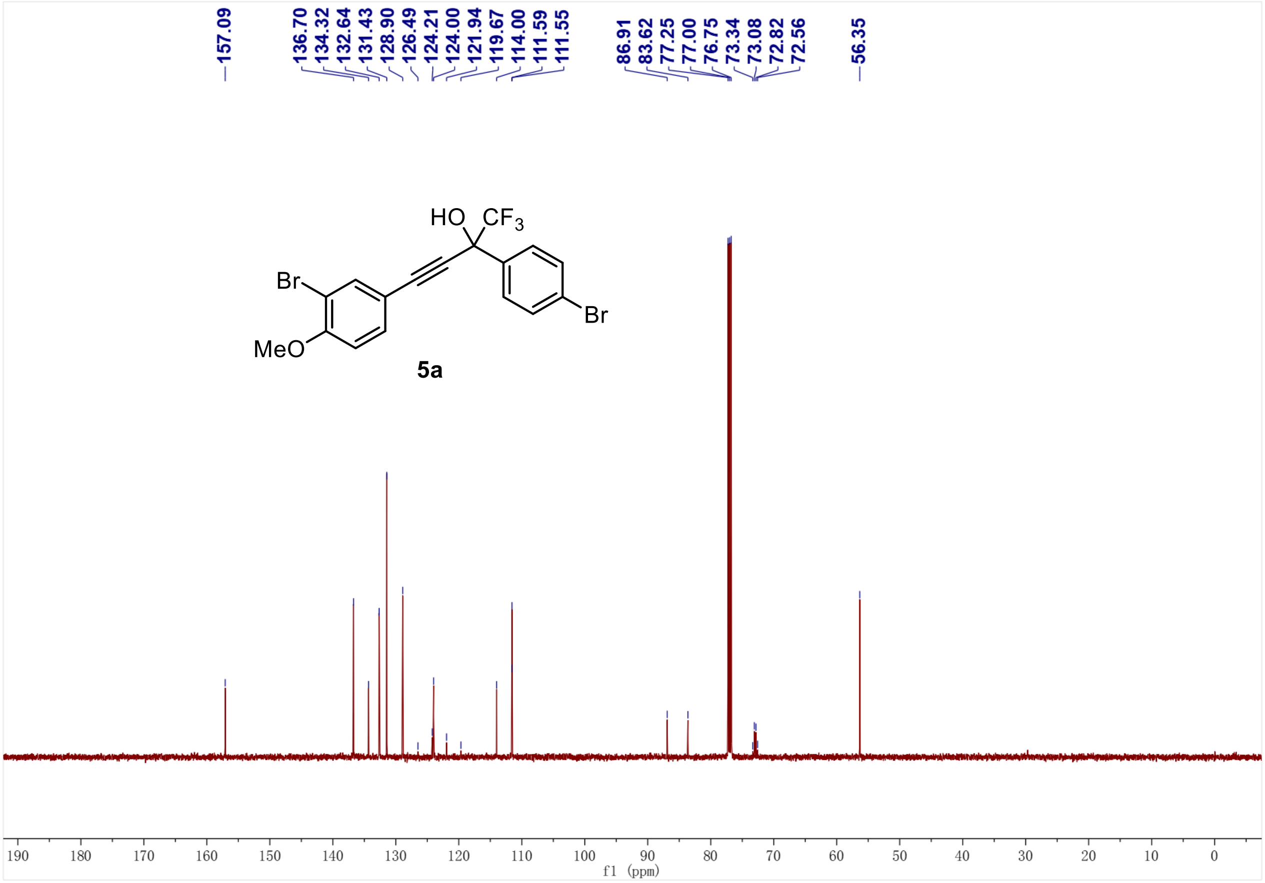

5a; ^{19}F NMR (376 MHz, $CDCl_3$)

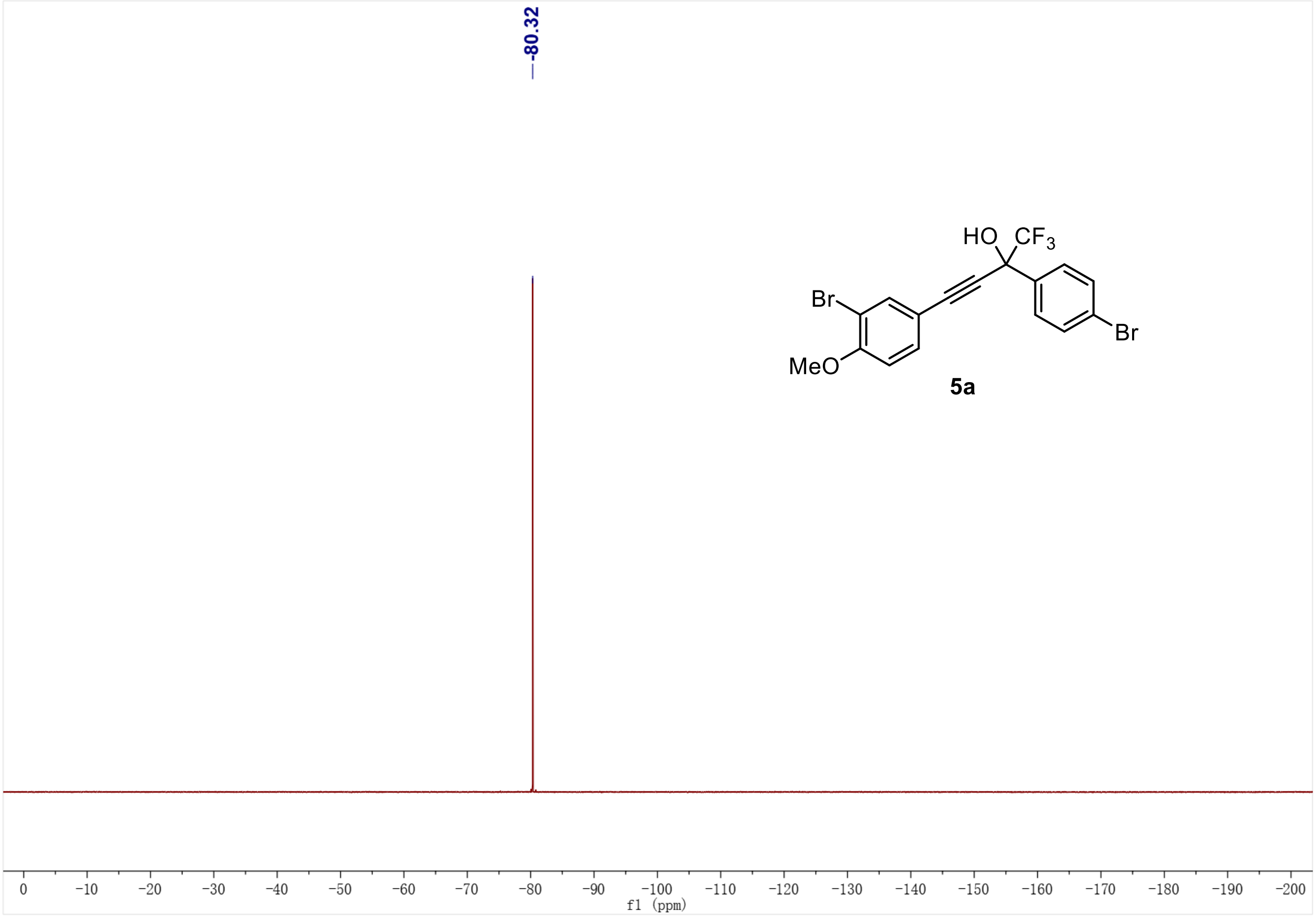

5b; ^1H NMR (400 MHz, $CDCl_3$); ^{13}C NMR (101 MHz, $CDCl_3$)

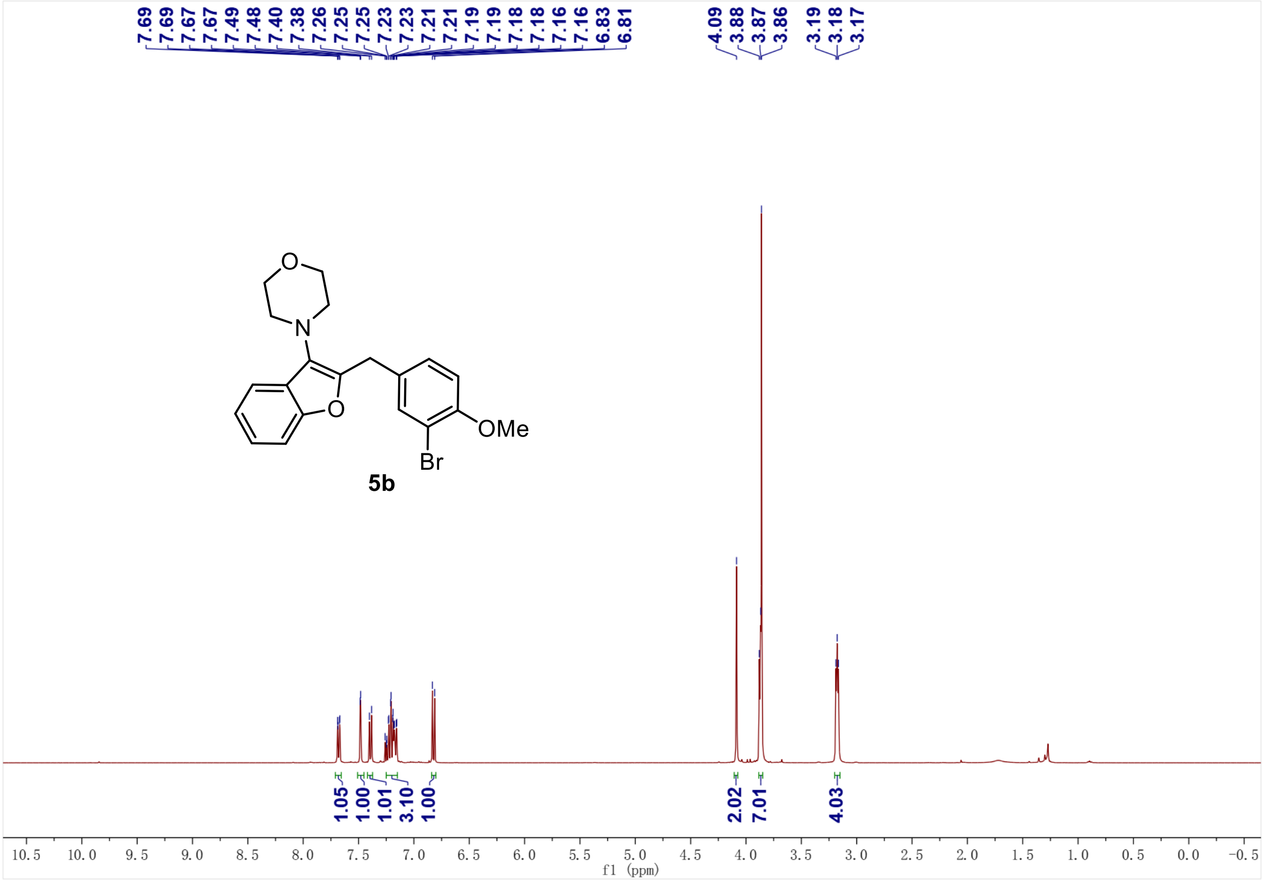

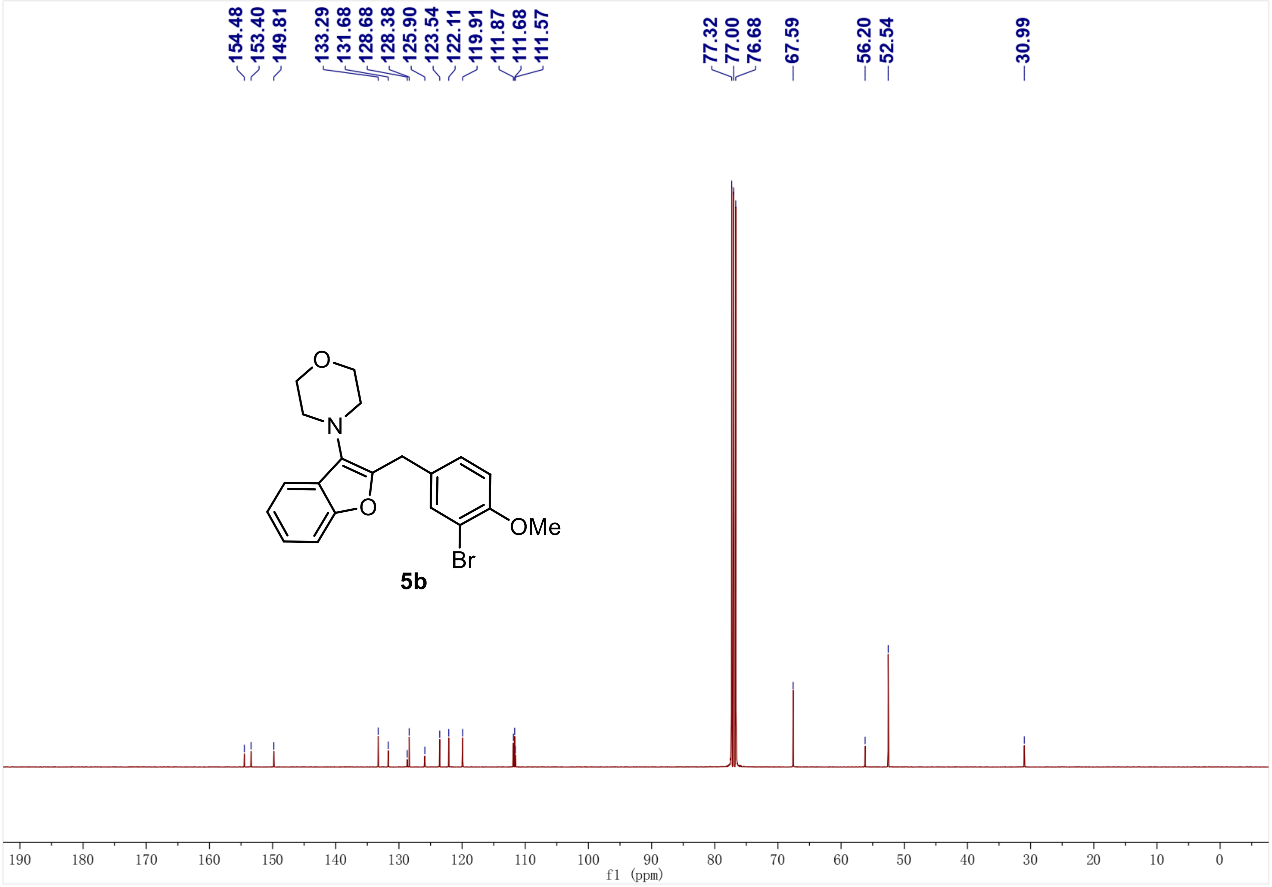

5c; ^{1}H NMR (400 MHz, $CDCl_3$); ^{13}C NMR (101 MHz, $CDCl_3$)

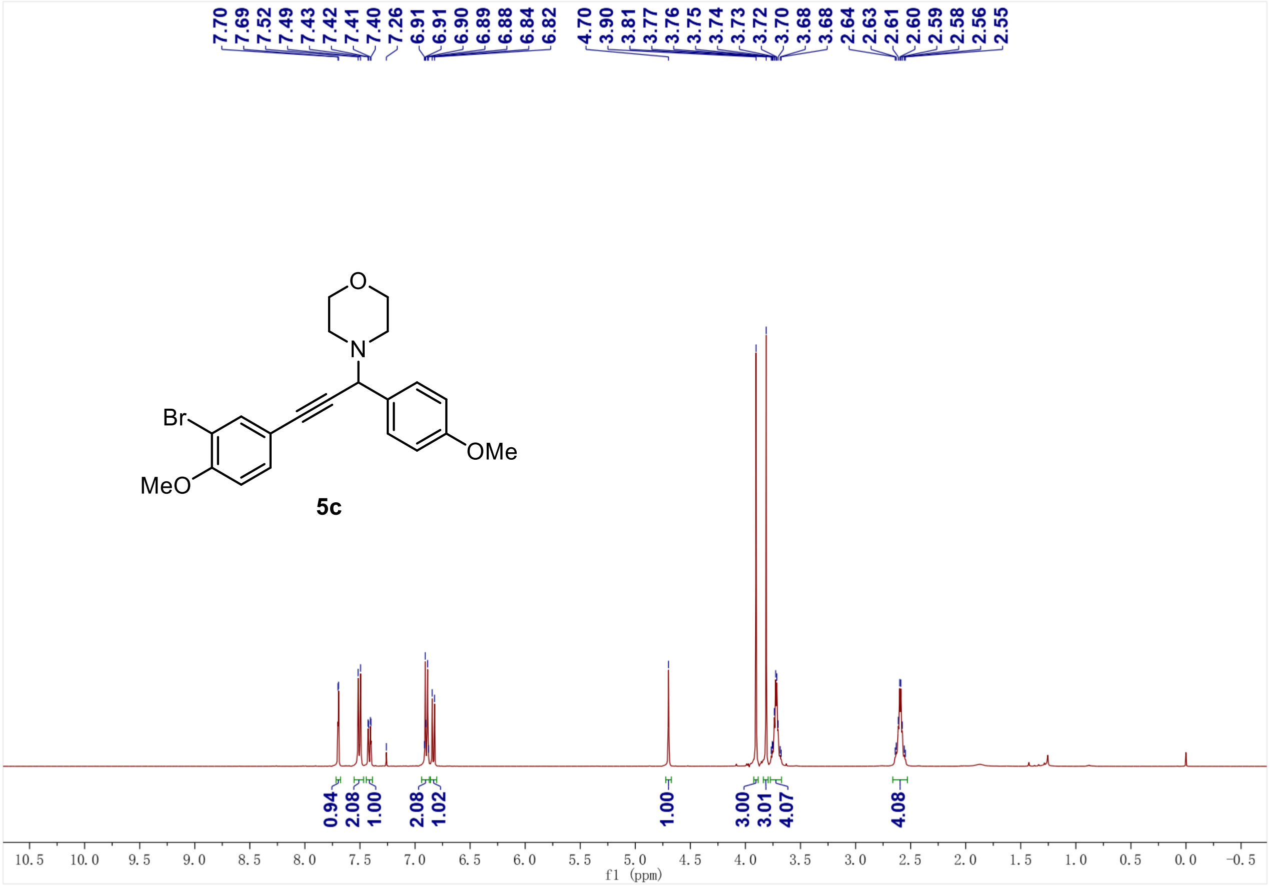

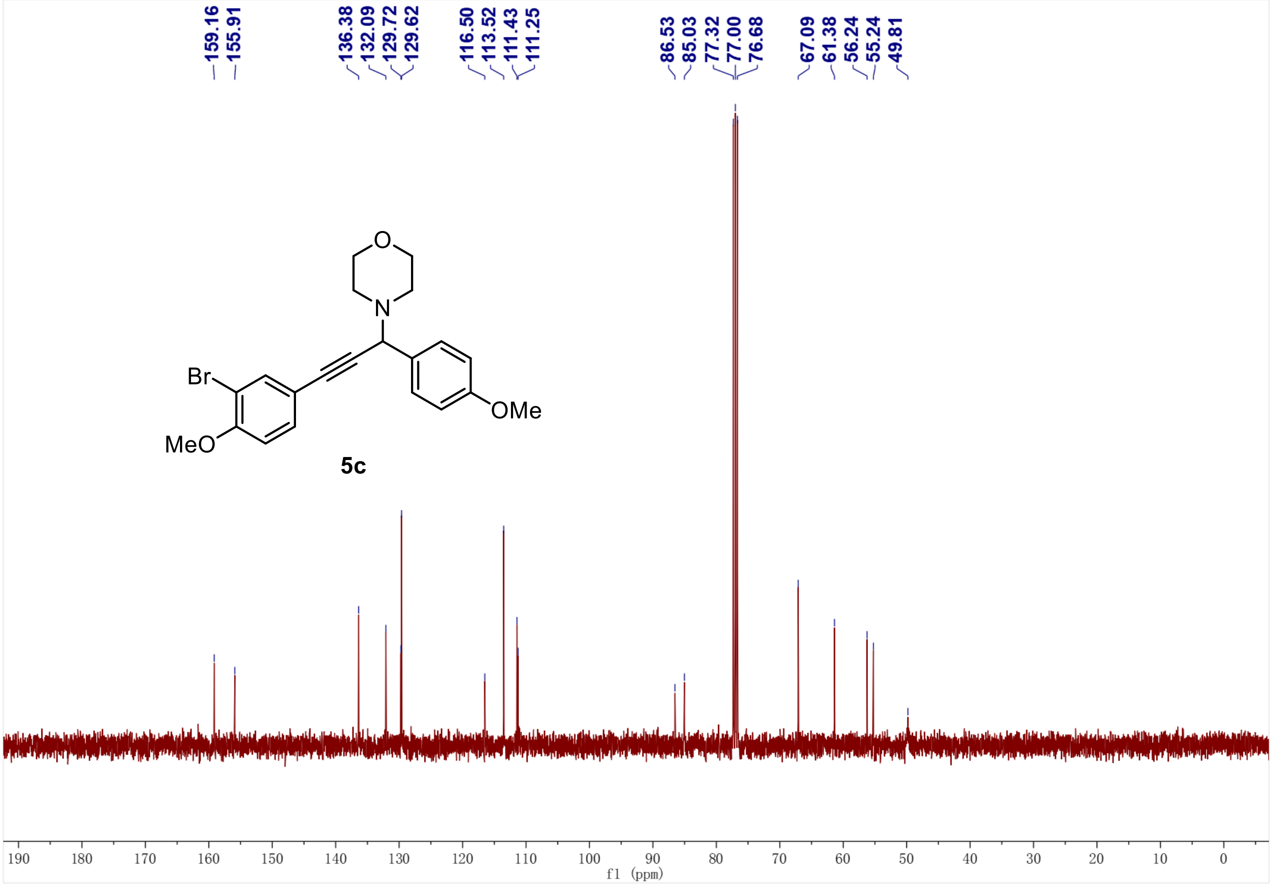

5d; ^{1}H NMR (400 MHz, $CDCl_3$); ^{13}C NMR (101 MHz, $CDCl_3$)

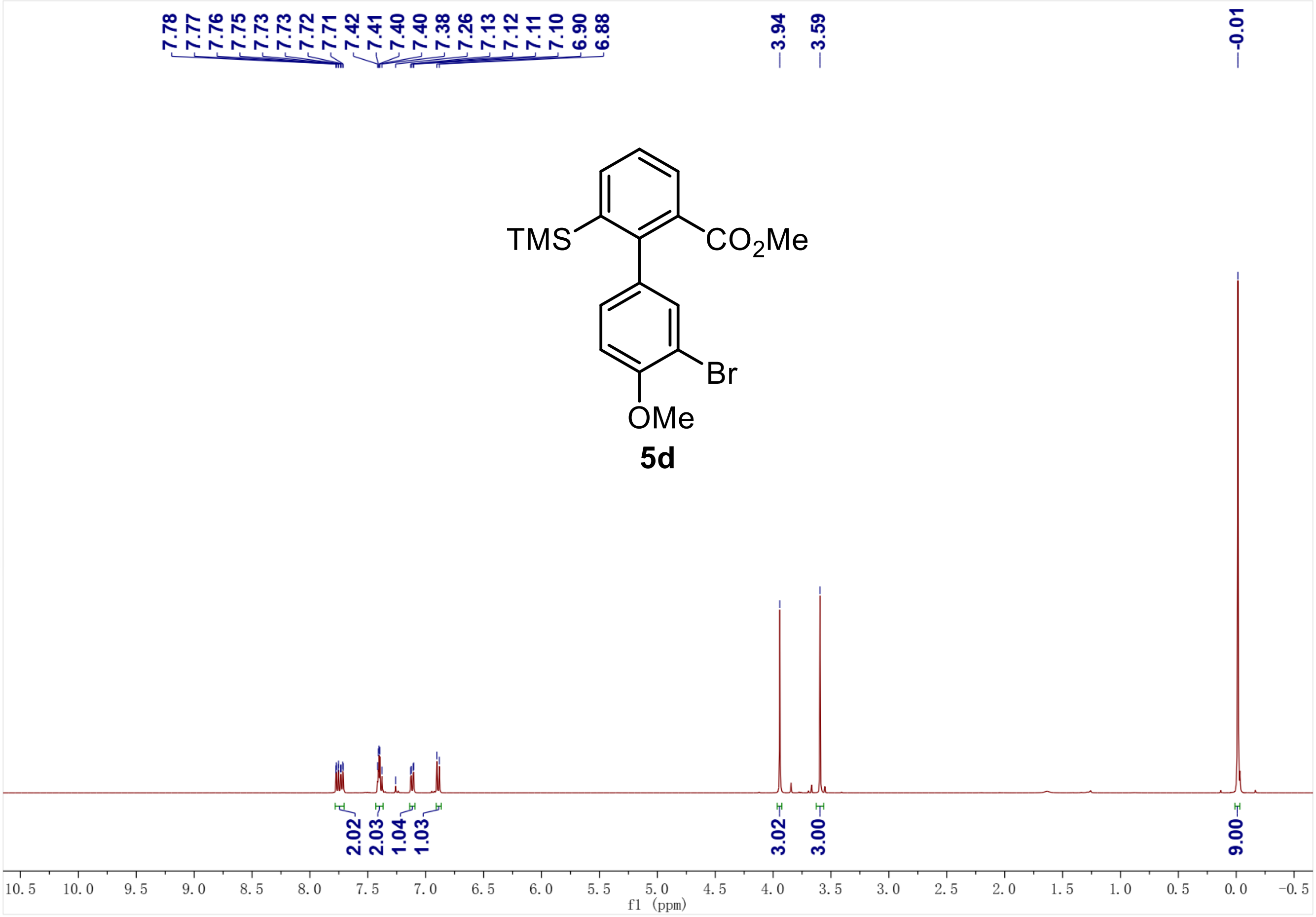

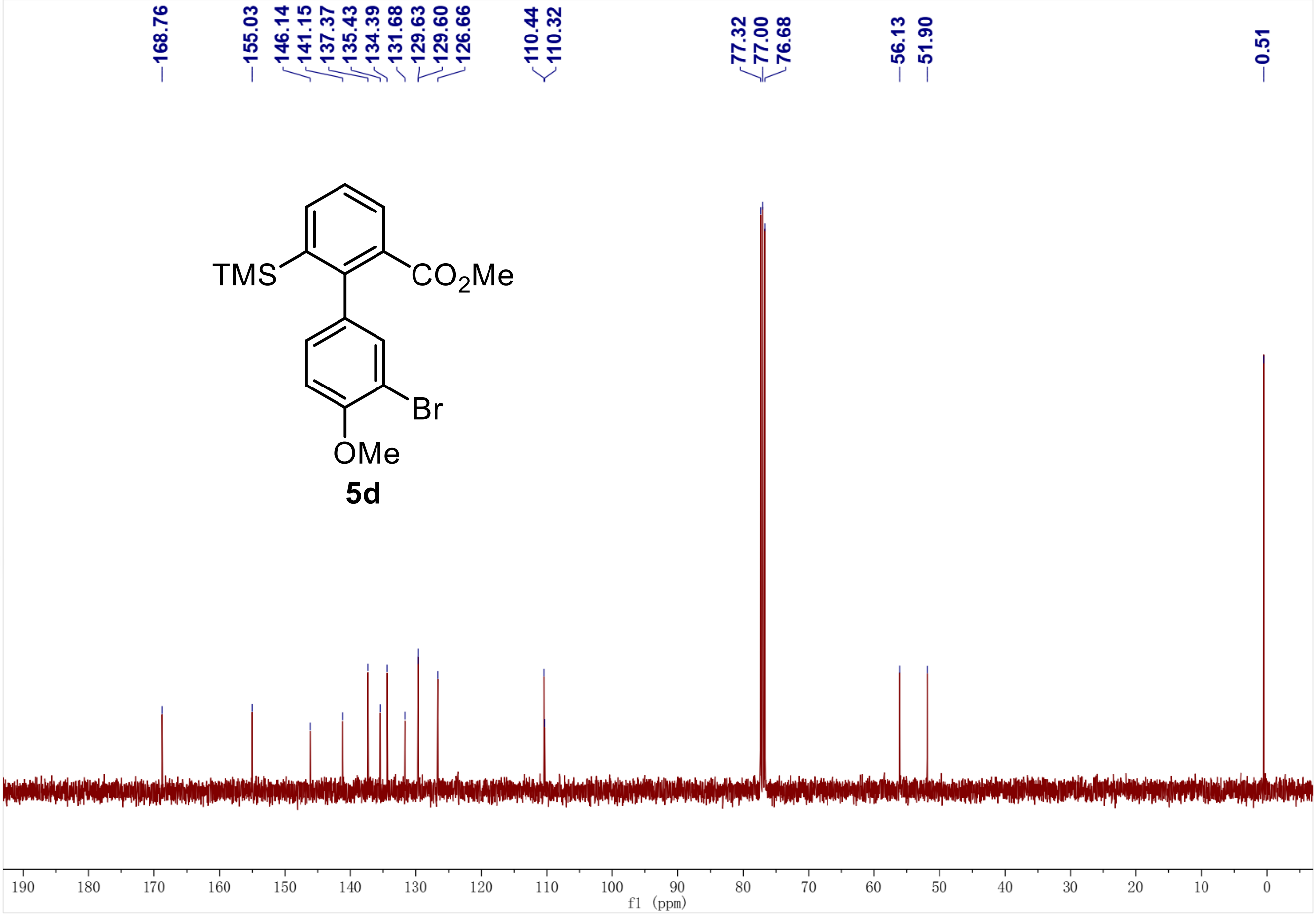

5e; ^{1}H NMR (400 MHz, $CDCl_3$); ^{13}C NMR (101 MHz, $CDCl_3$)

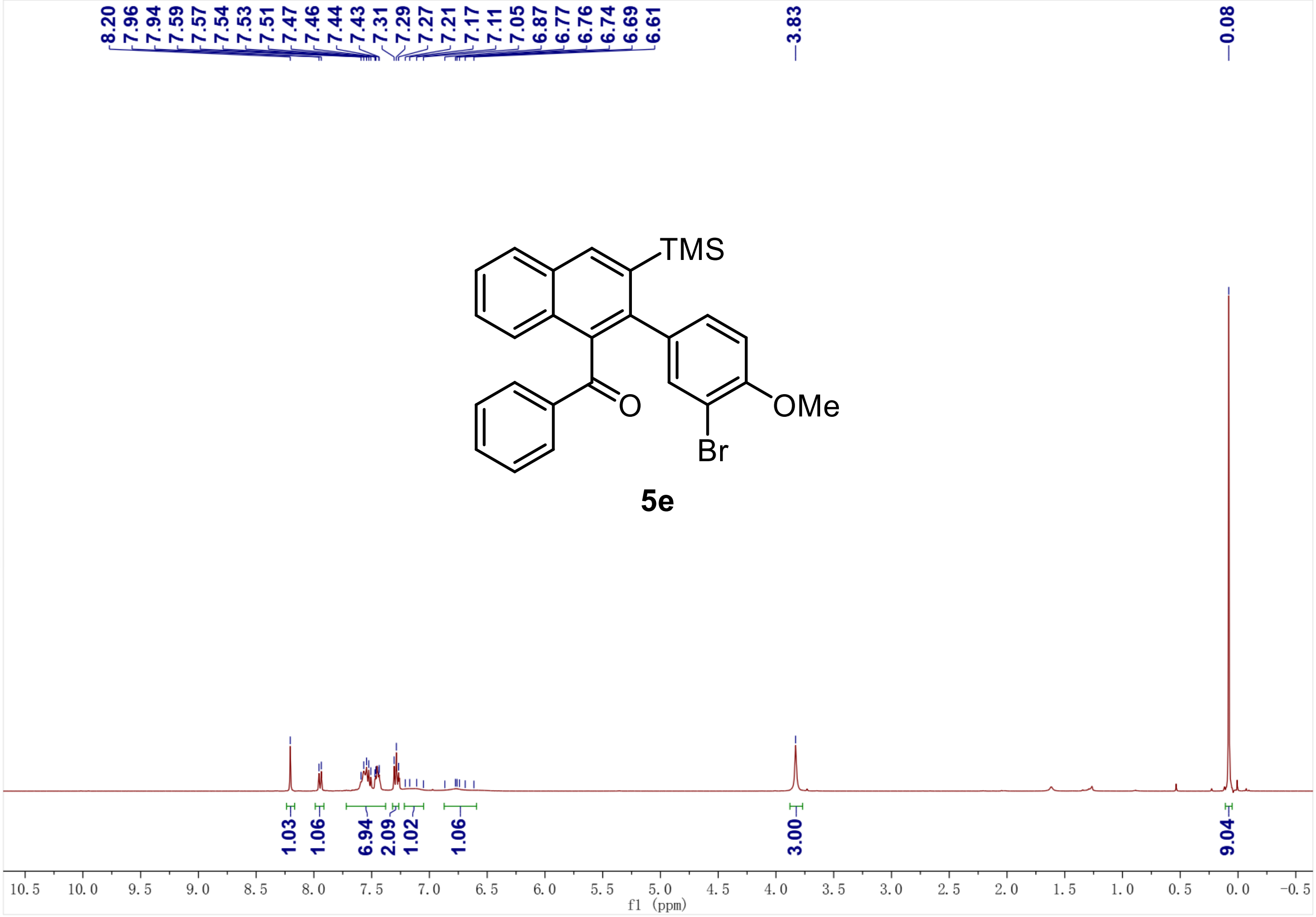

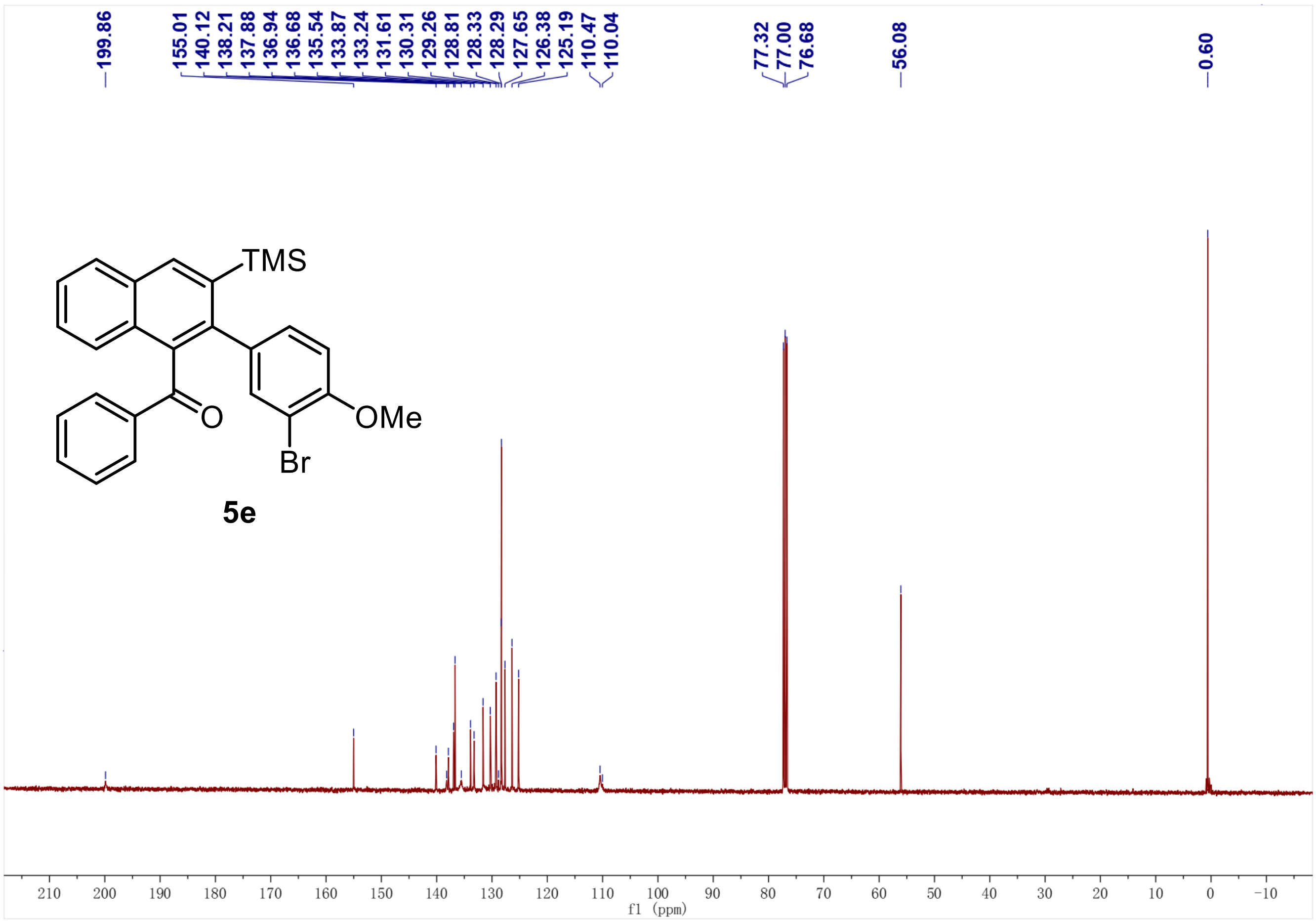

5f; ^{1}H NMR (400 MHz, CDCl$_3$); ^{13}C NMR (101 MHz, CDCl$_3$)

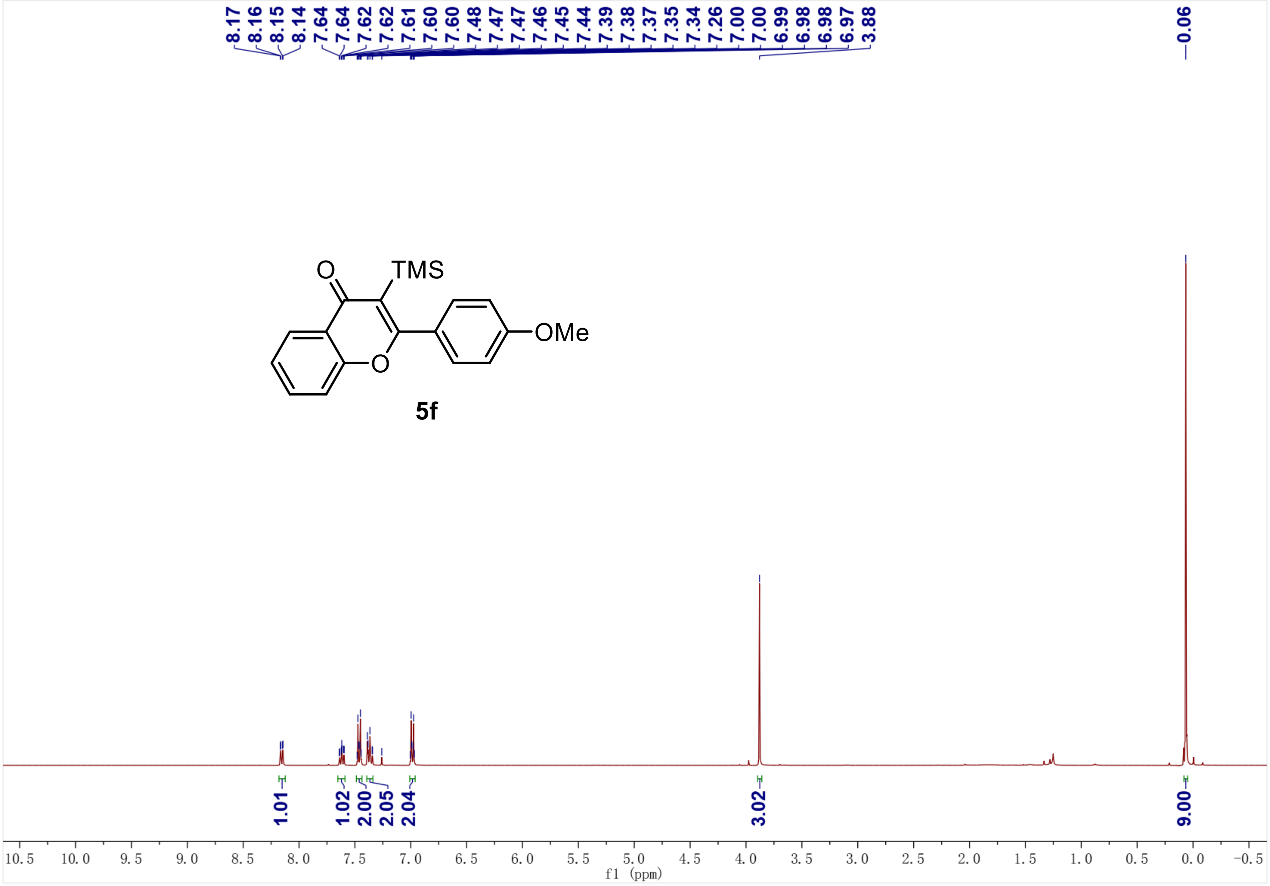

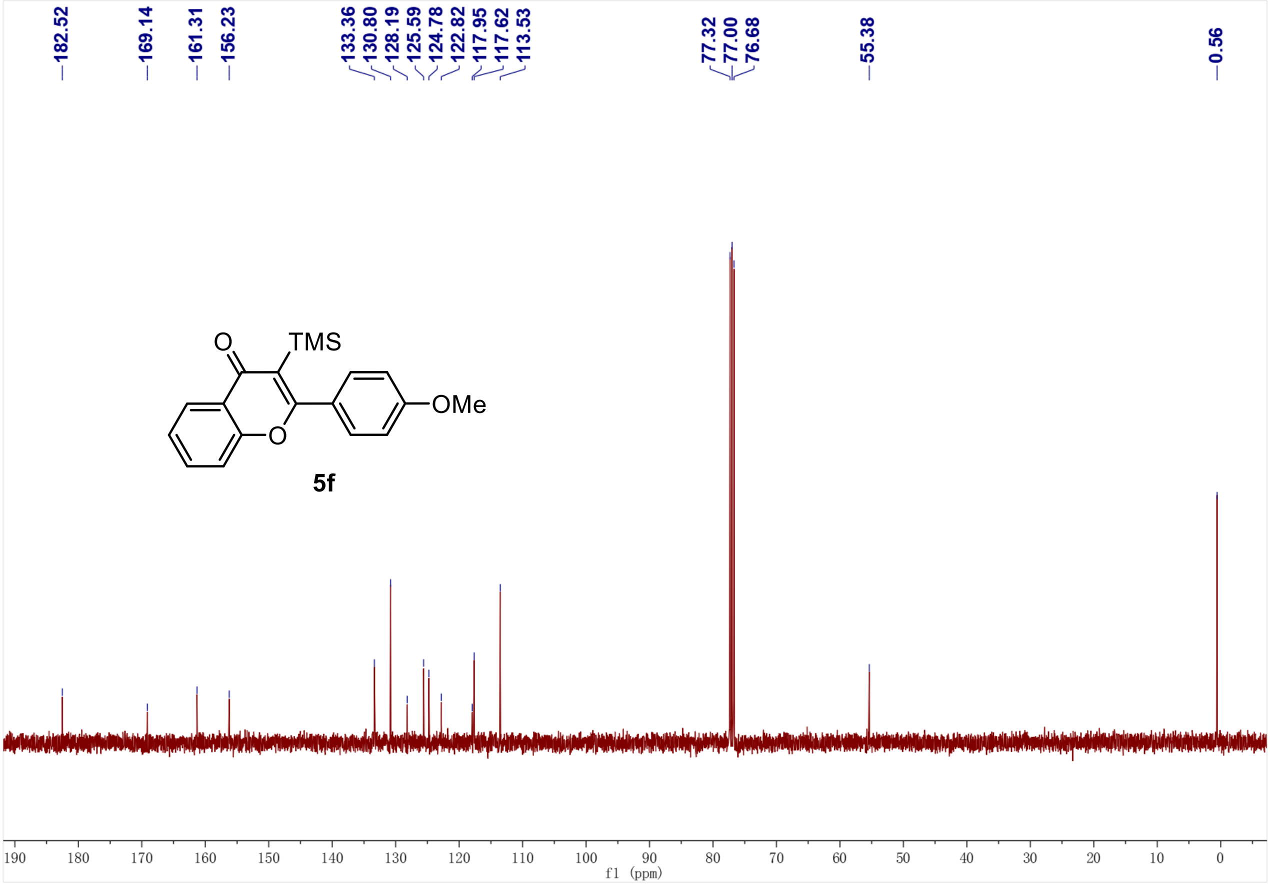

5g; ^{1}H NMR (400 MHz, $CDCl_3$); ^{13}C NMR (101 MHz, $CDCl_3$)

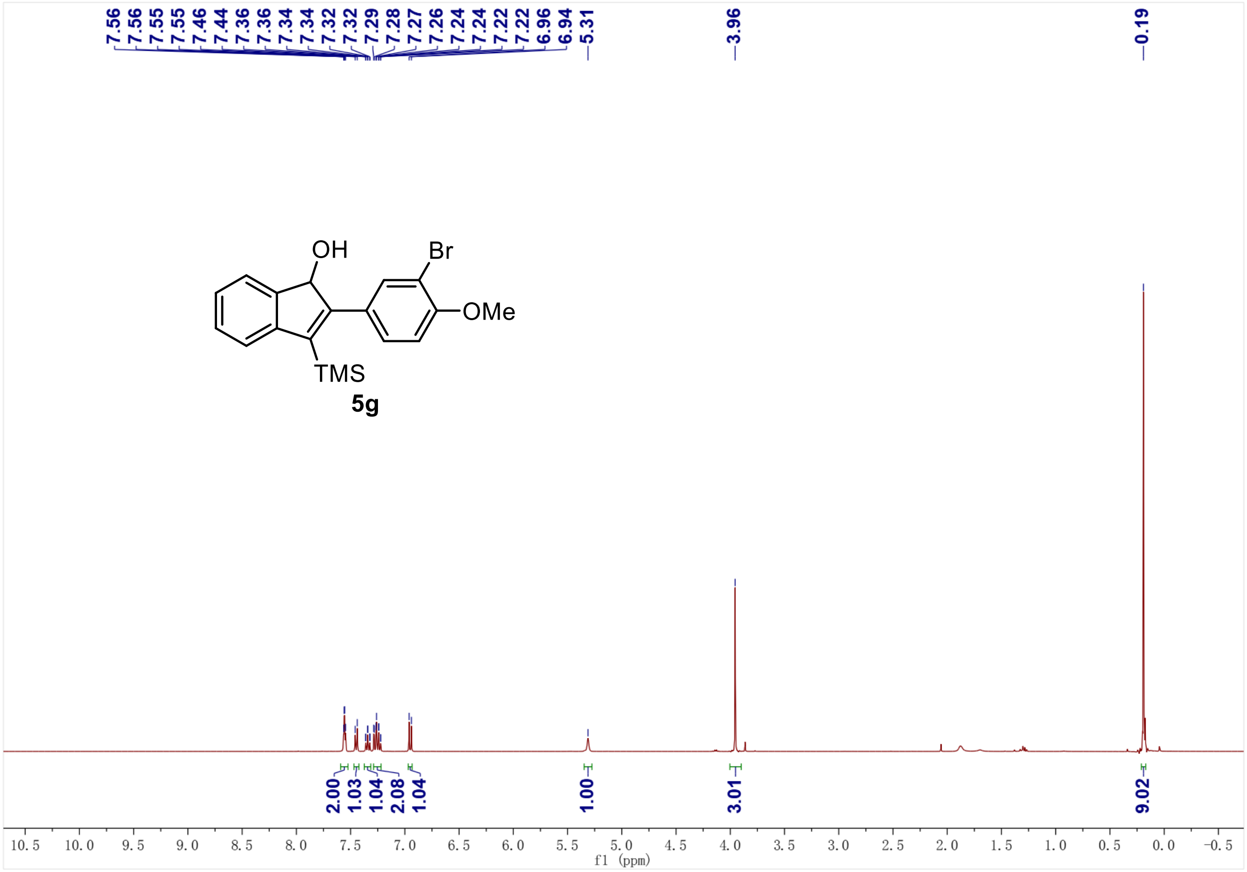

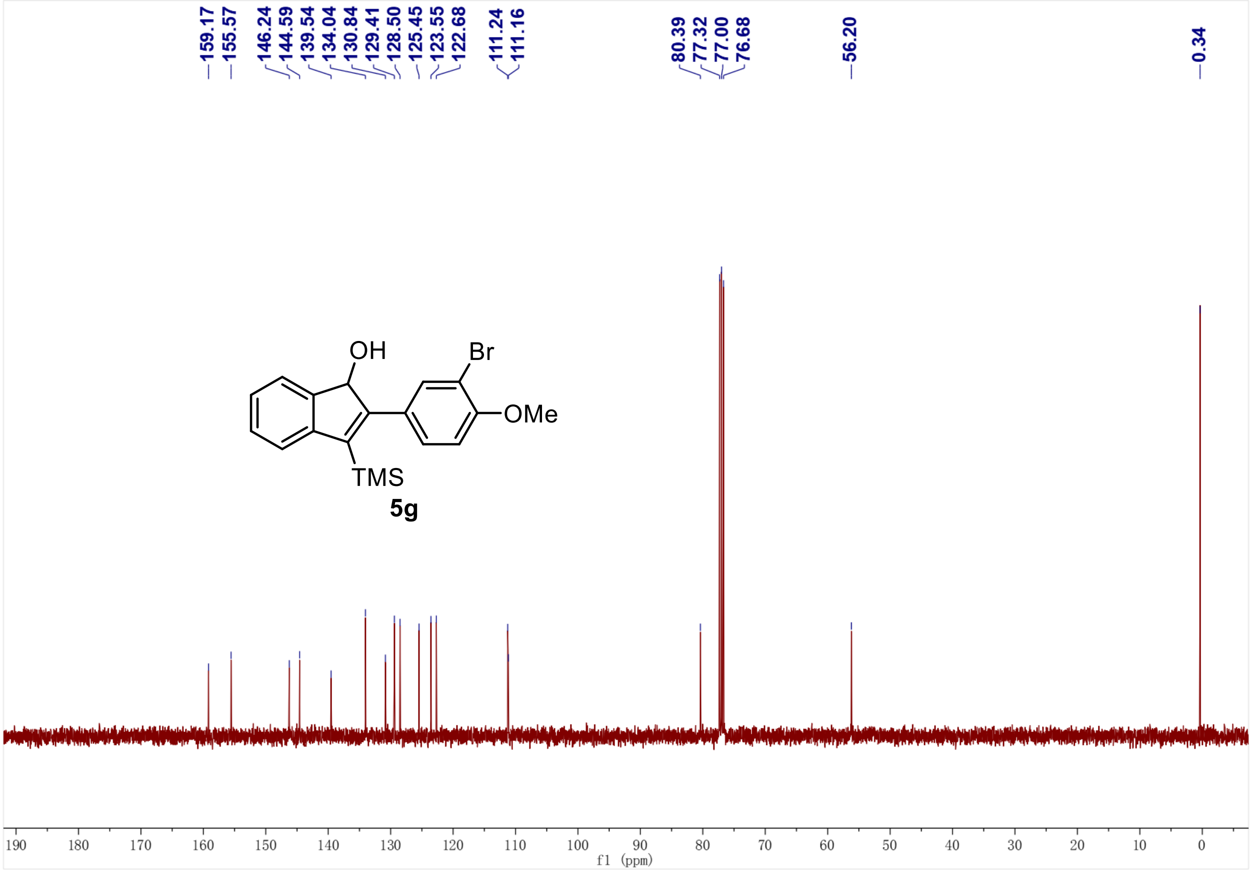

5h; ^{1}H NMR (400 MHz, $CDCl_3$); ^{13}C NMR (101 MHz, $CDCl_3$)

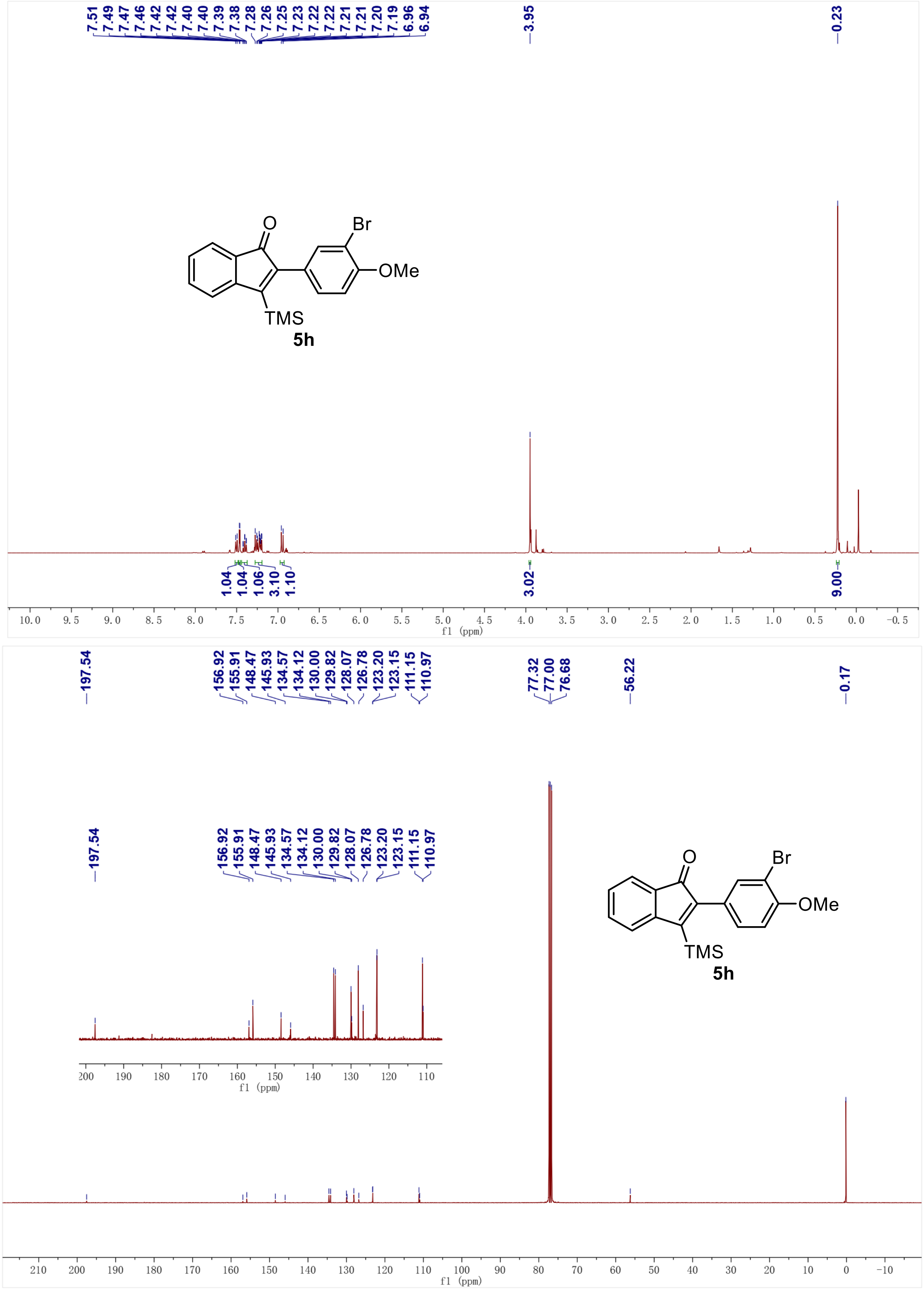

3bf; ^{1}H NMR (400 MHz, $CDCl_3$); ^{13}C NMR (126 MHz, $CDCl_3$)

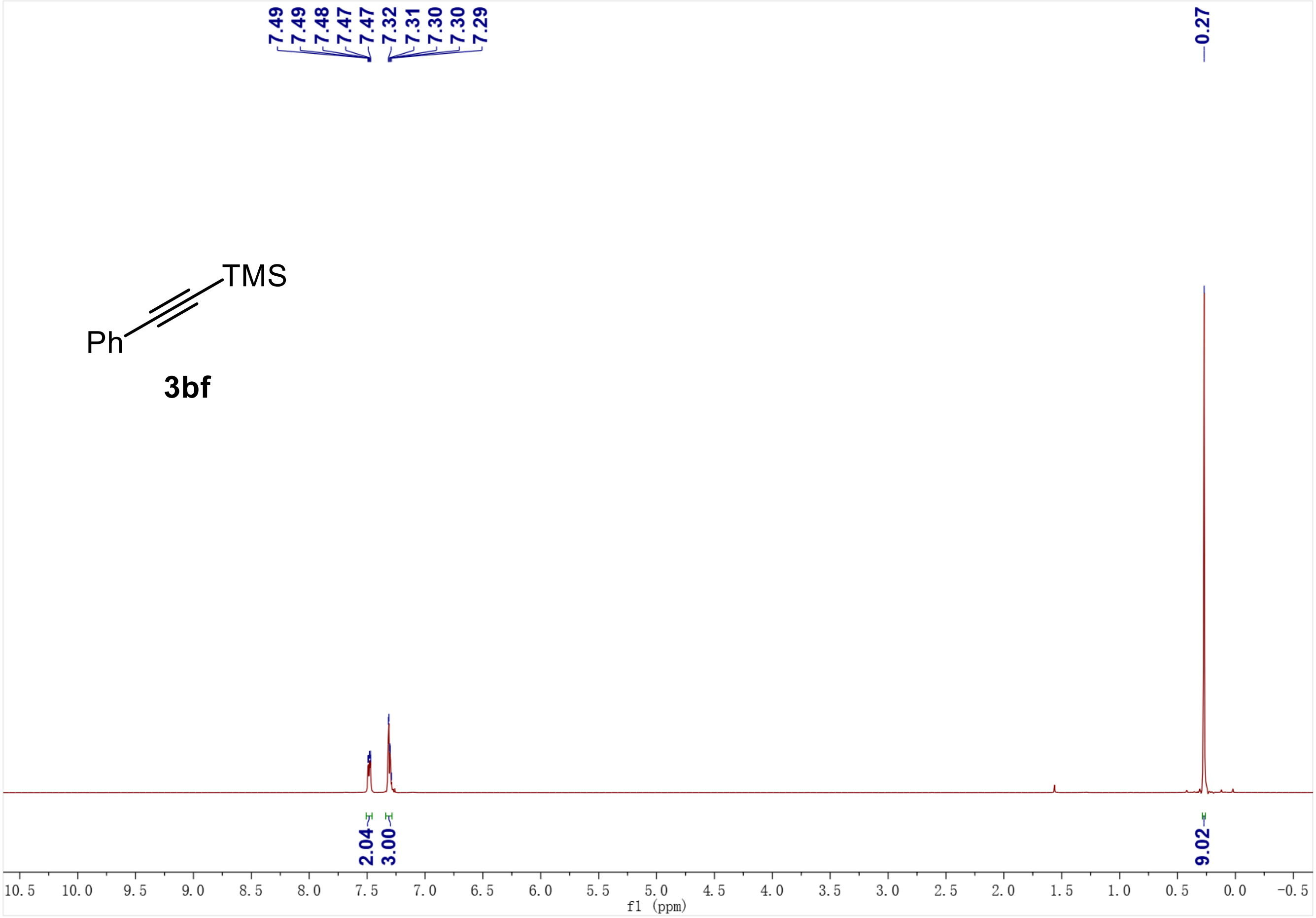

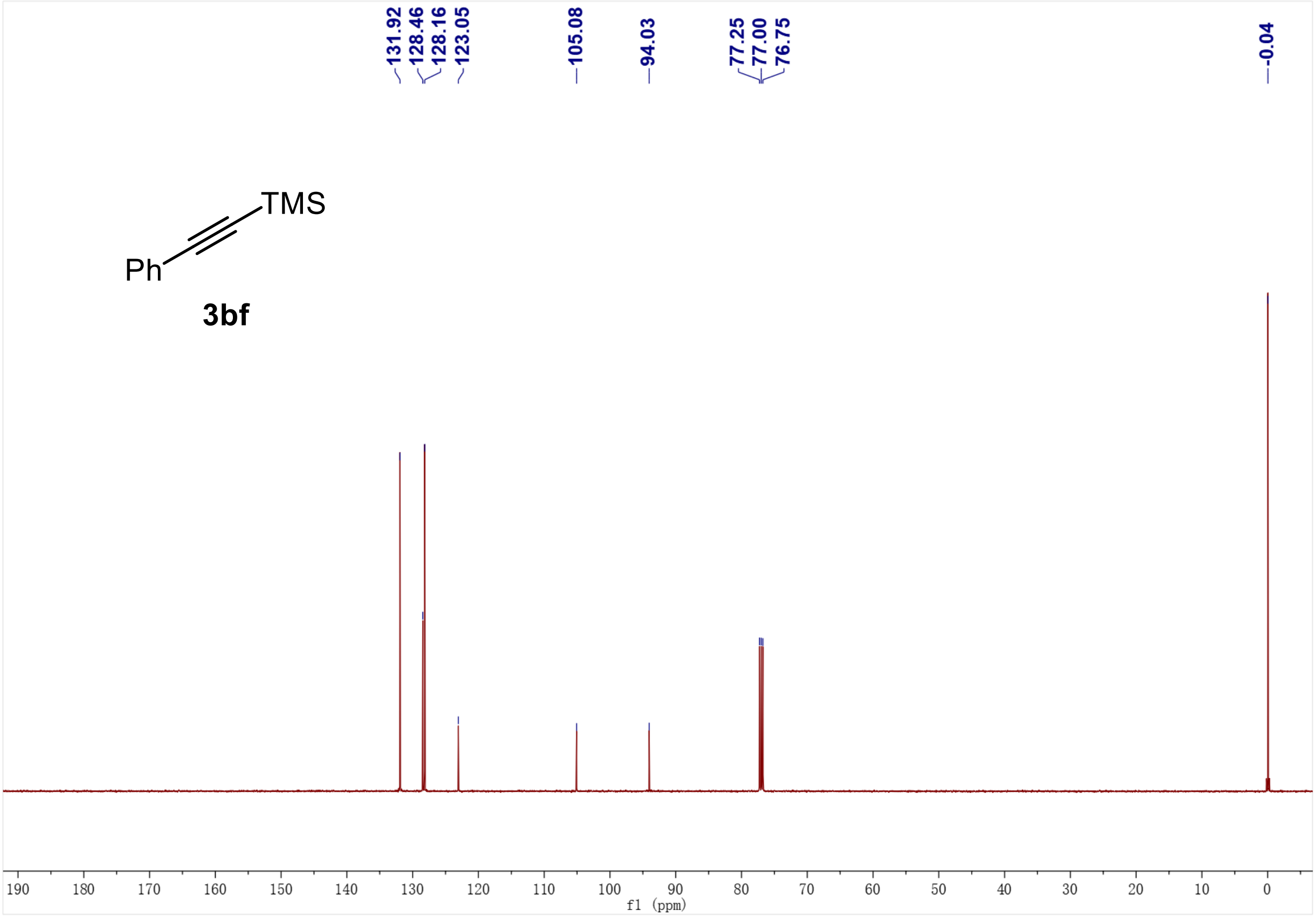

3bf-1; ^{1}H NMR (400 MHz, $CDCl_3$); ^{13}C NMR (126 MHz, $CDCl_3$)

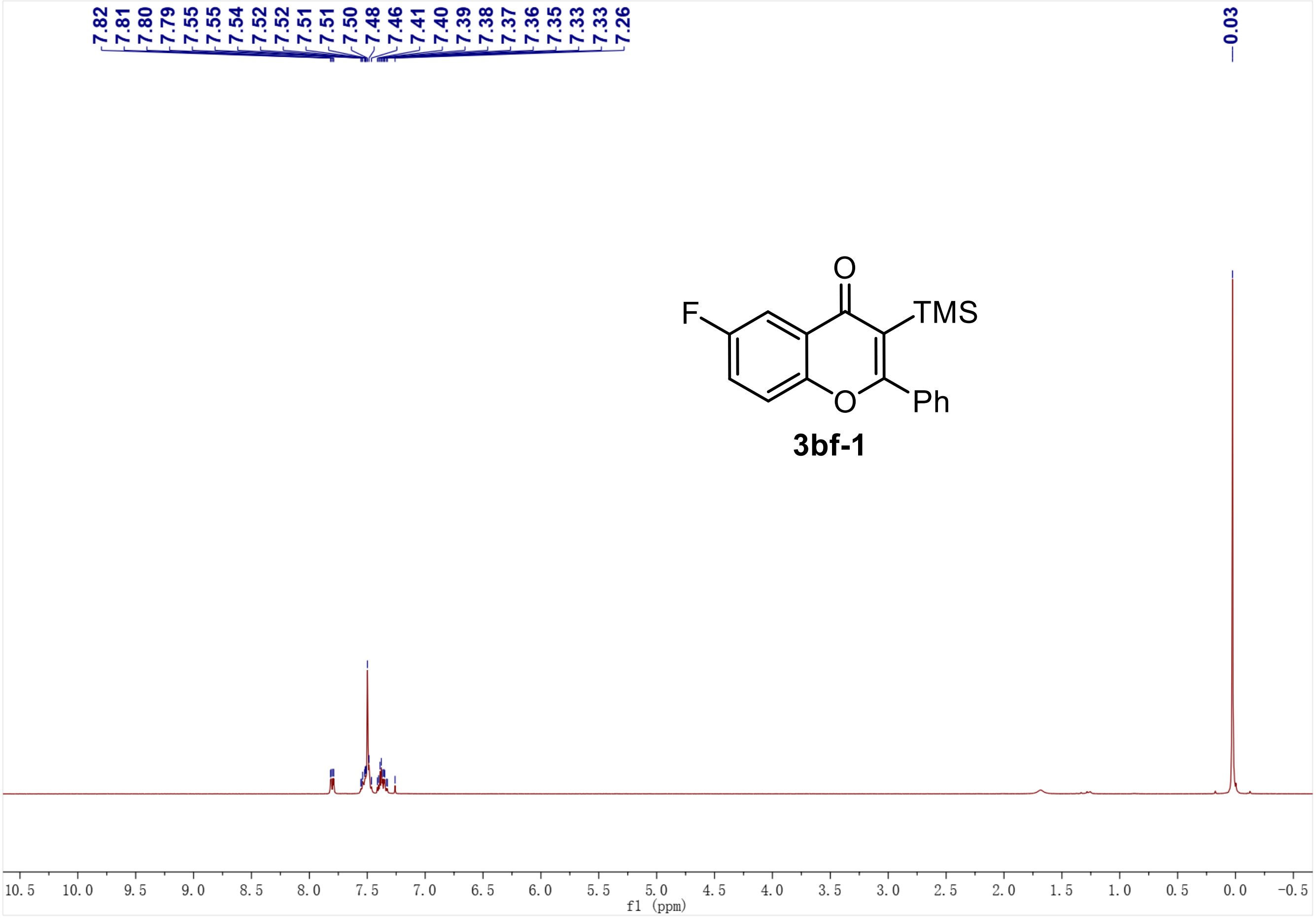

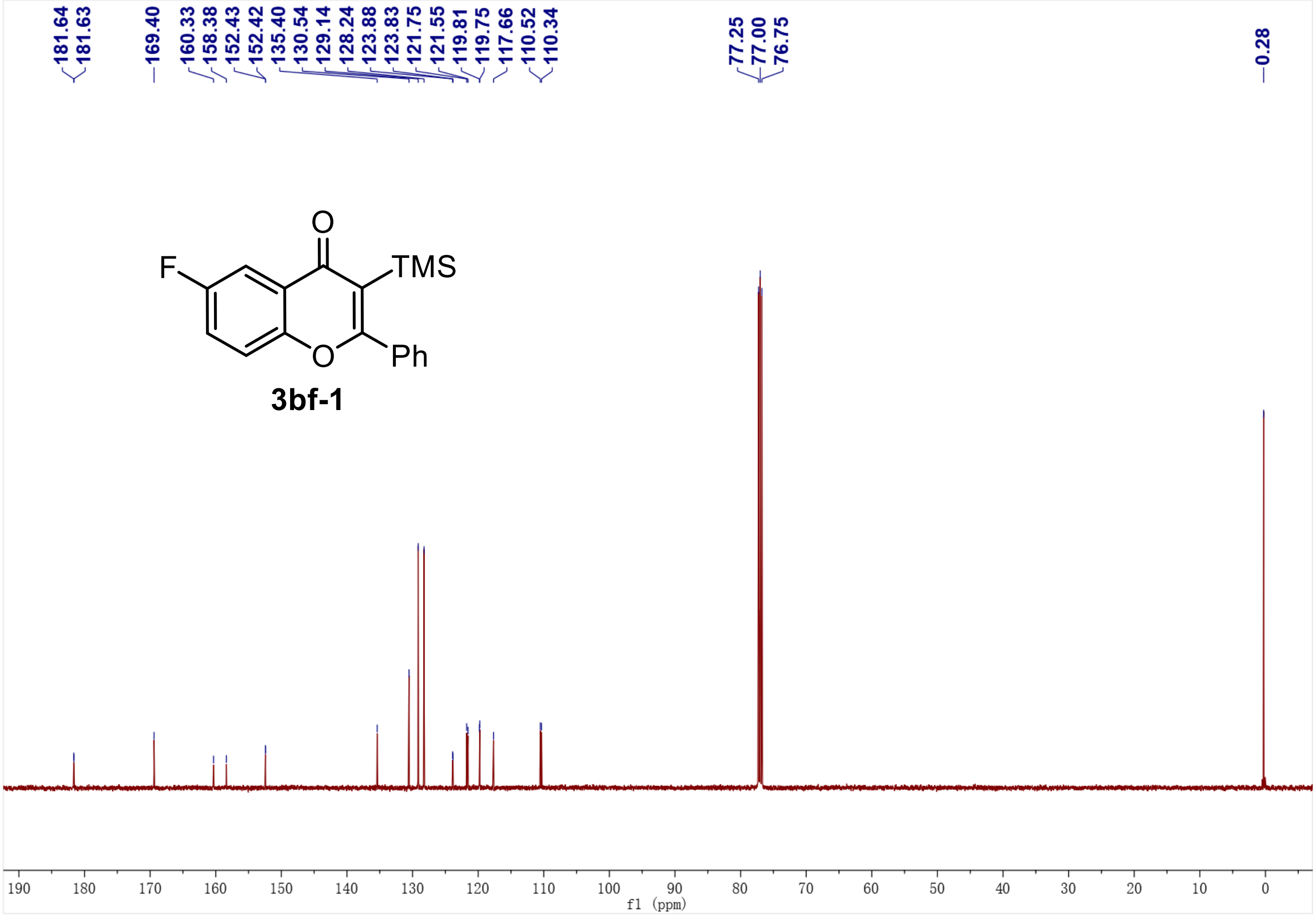

3bf-1; ^{19}F NMR (376 MHz, $CDCl_3$)

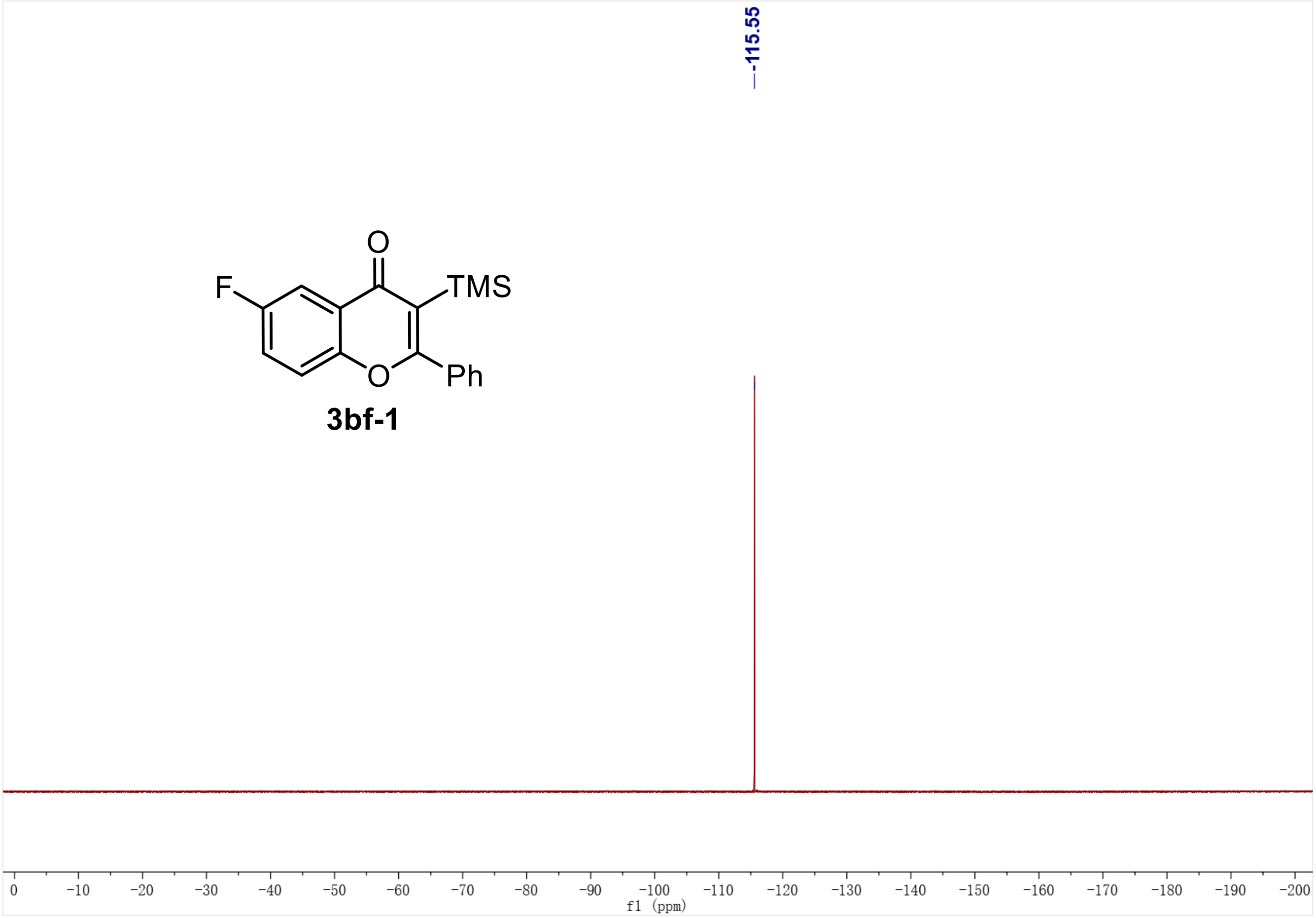

6-fluoroflavone; ¹H NMR (400 MHz, $CDCl_3$); ¹³C NMR (101 MHz, $CDCl_3$)

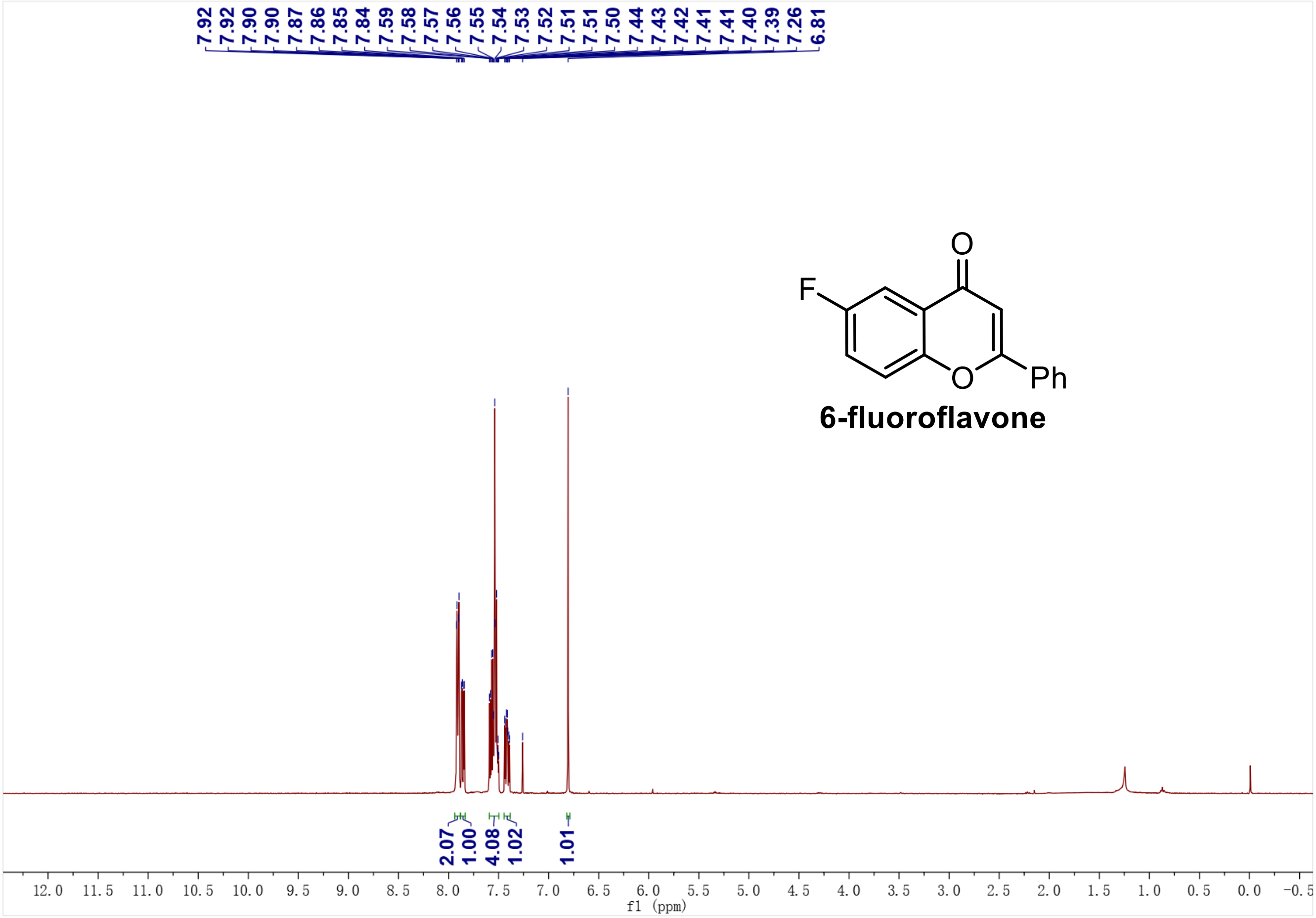

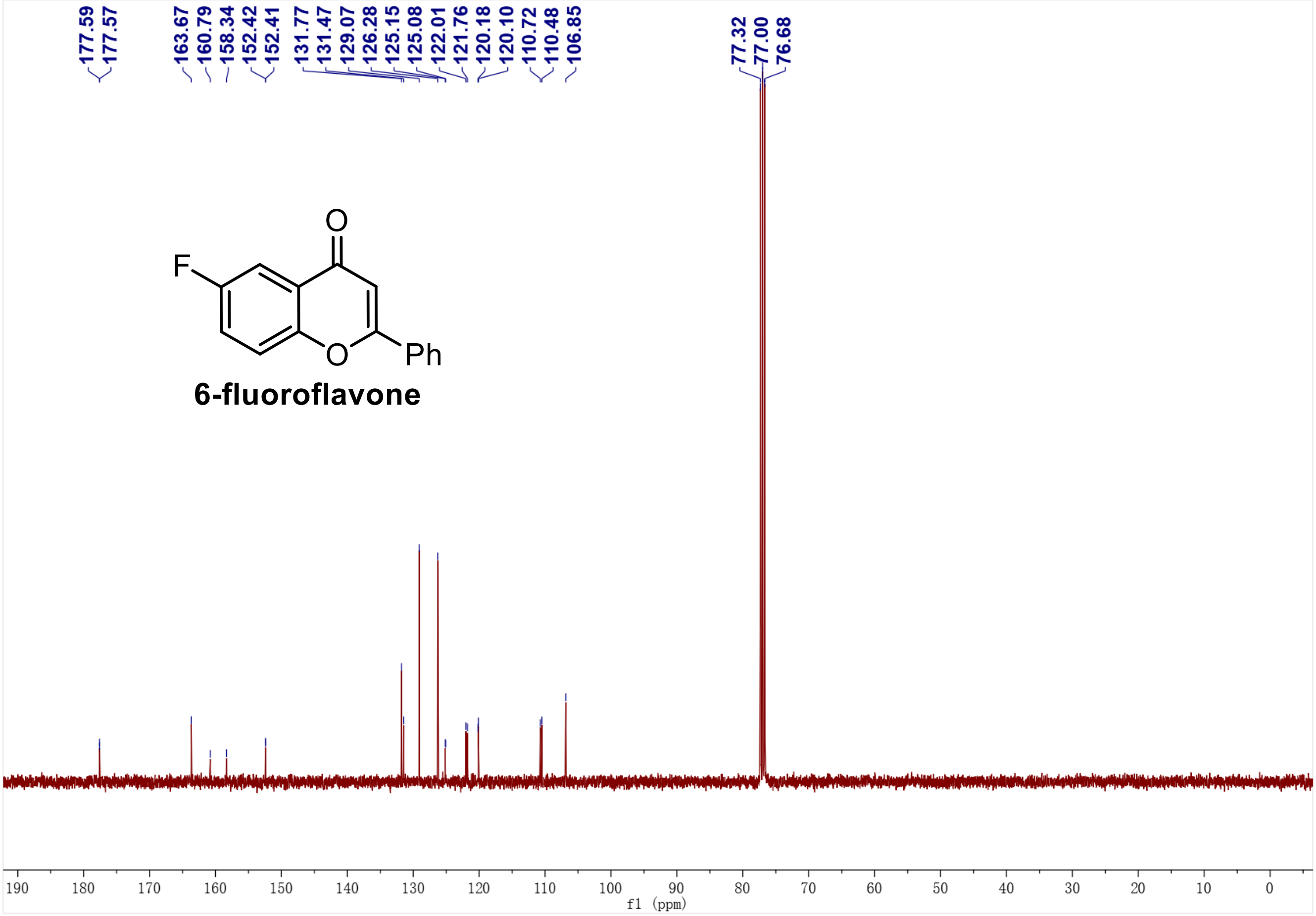

6-fluoroflavone; ^{19}F NMR (376 MHz, $CDCl_3$)

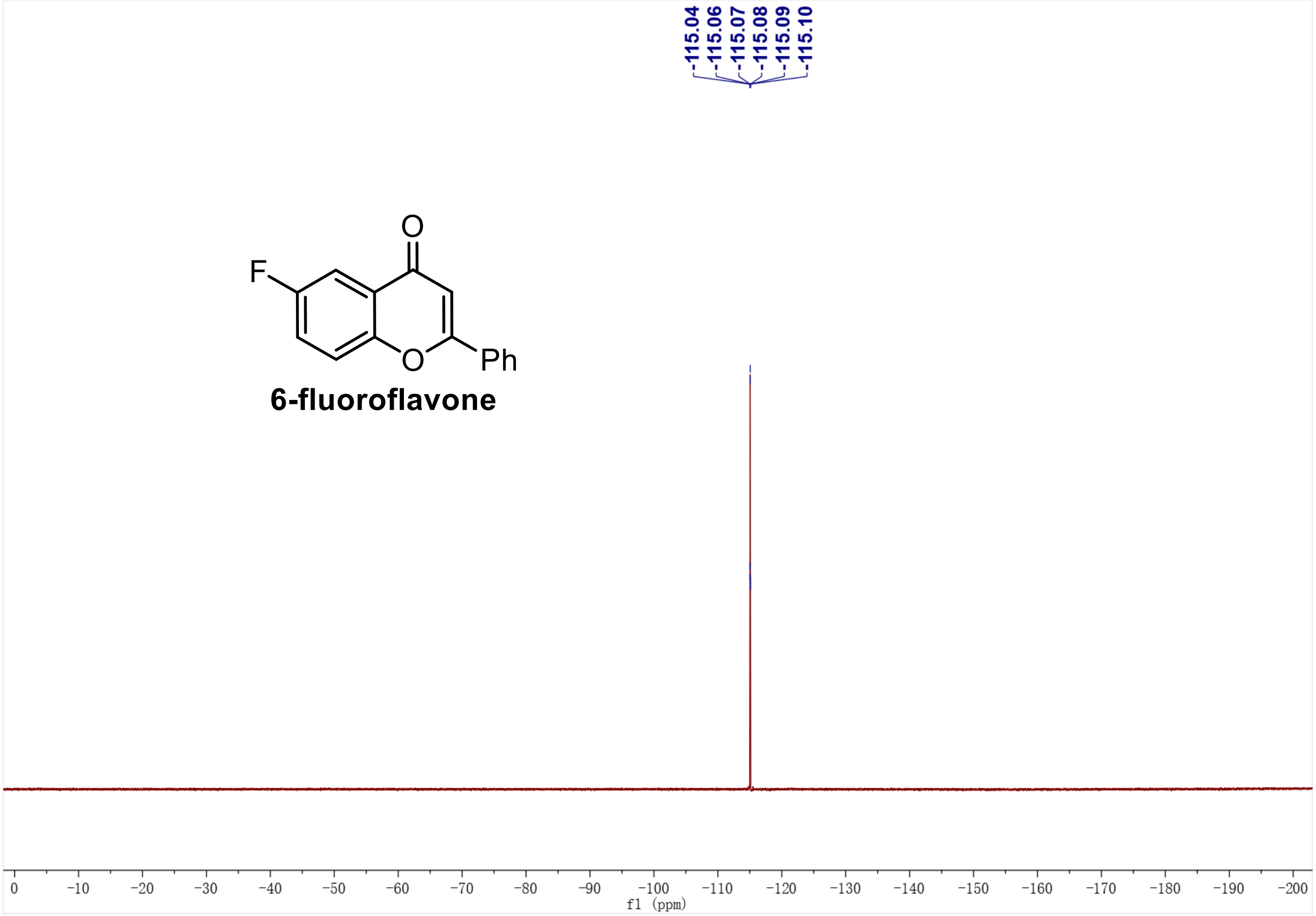

6a; ^{1}H NMR (400 MHz, CDCl$_3$); ^{13}C NMR (101 MHz, CDCl$_3$)

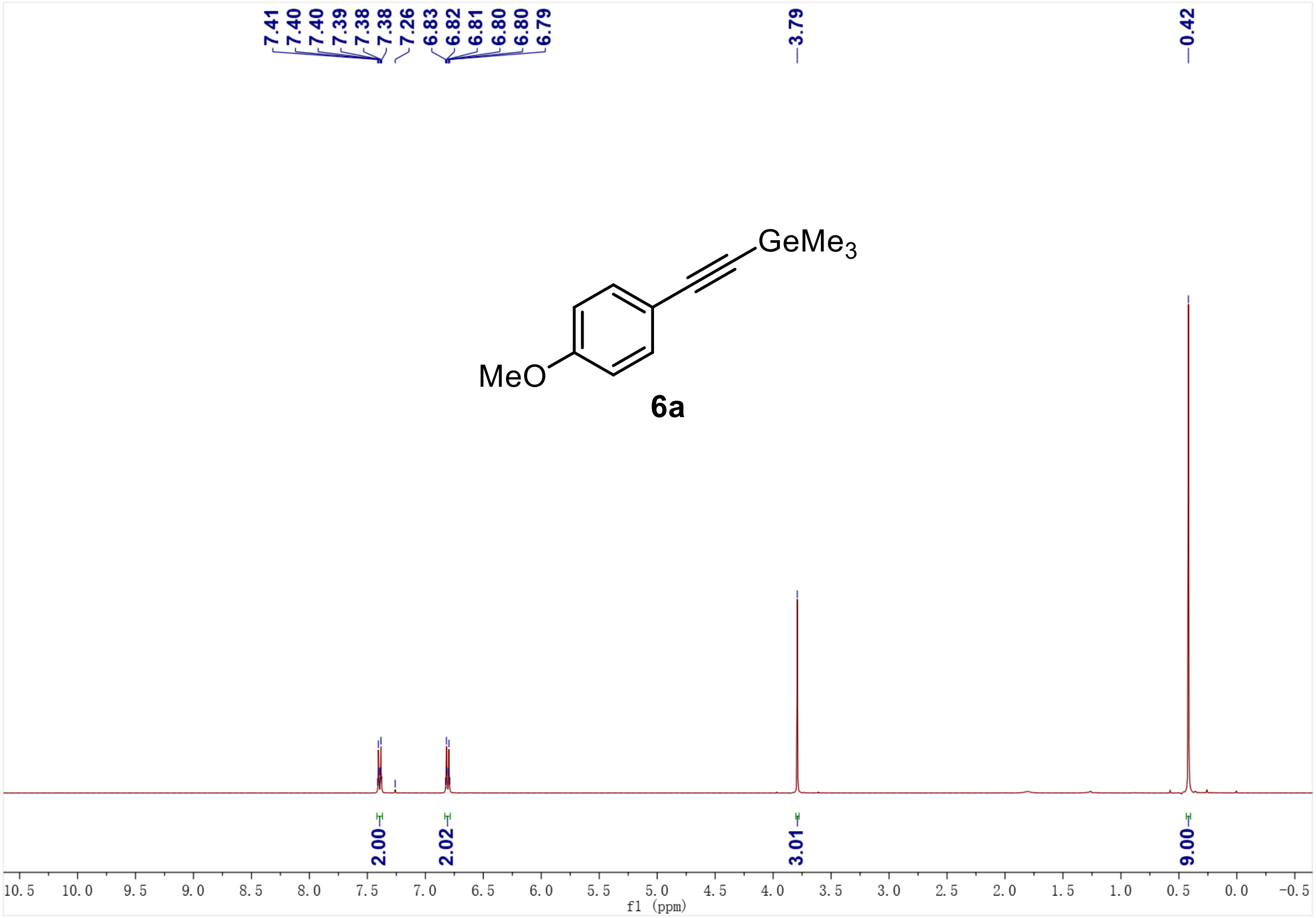

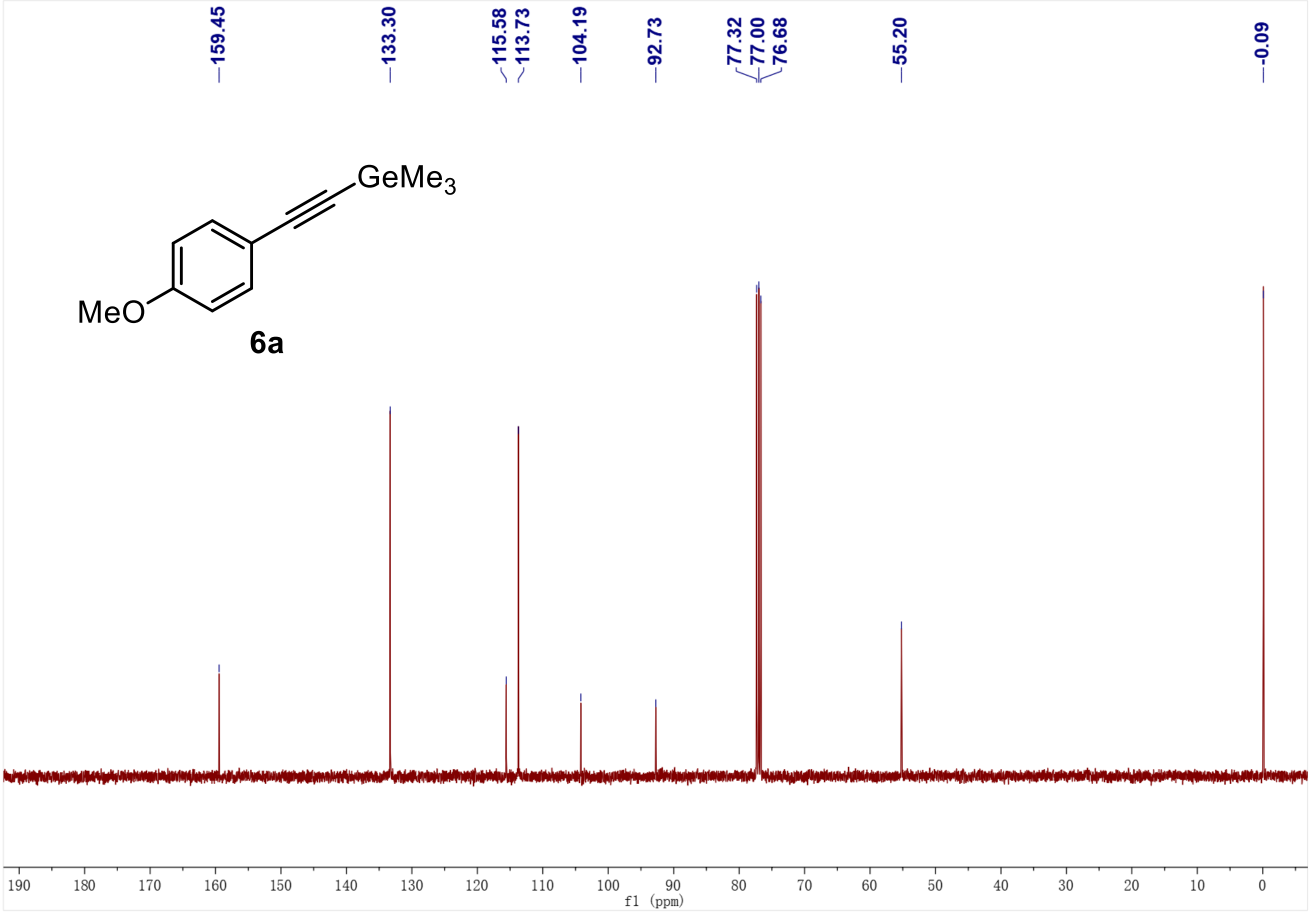

6b; ^{1}H NMR (400 MHz, CDCl$_3$); ^{13}C NMR (101 MHz, CDCl$_3$)

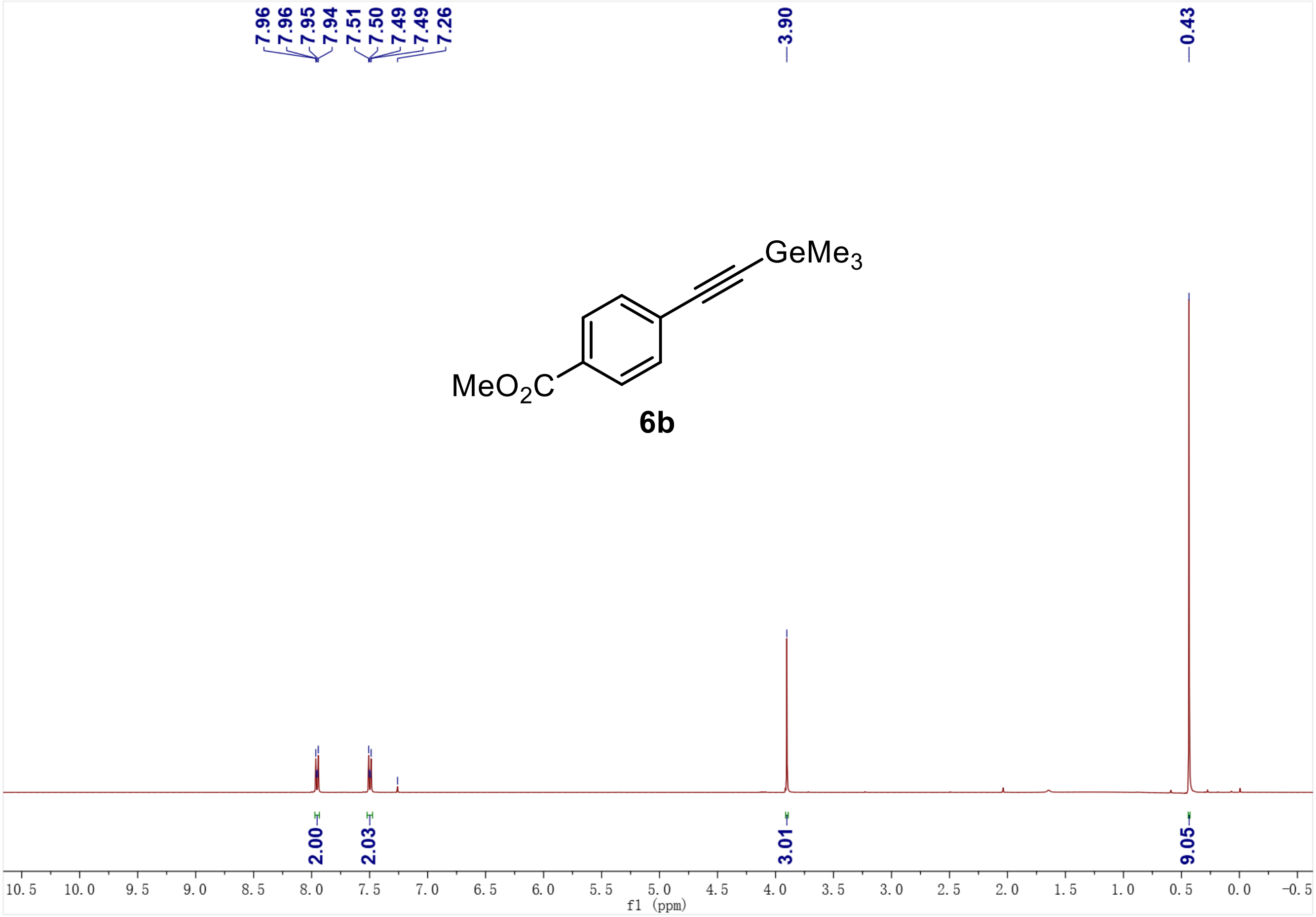

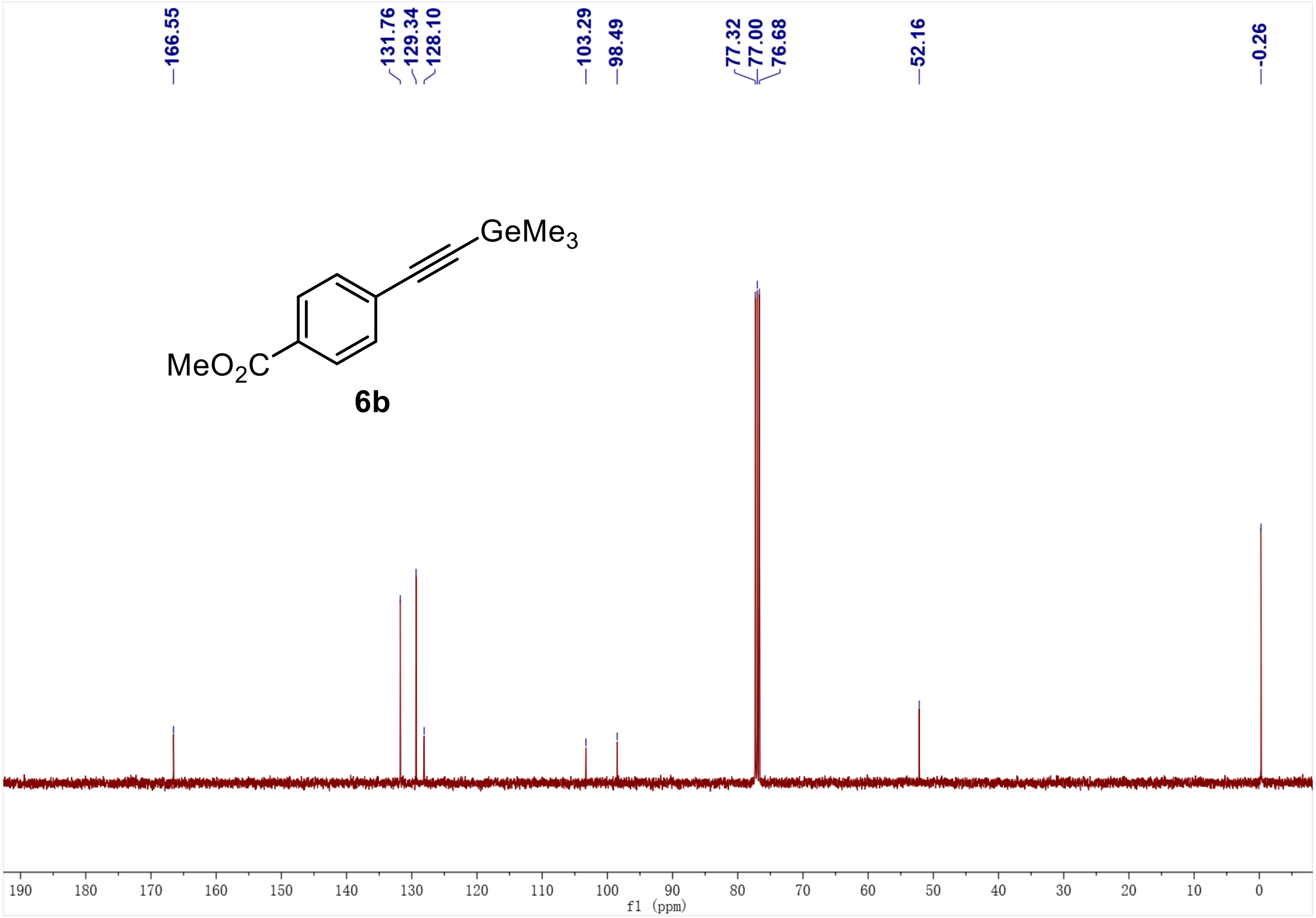

6c; ^{1}H NMR (400 MHz, $CDCl_3$); ^{13}C NMR (101 MHz, $CDCl_3$)

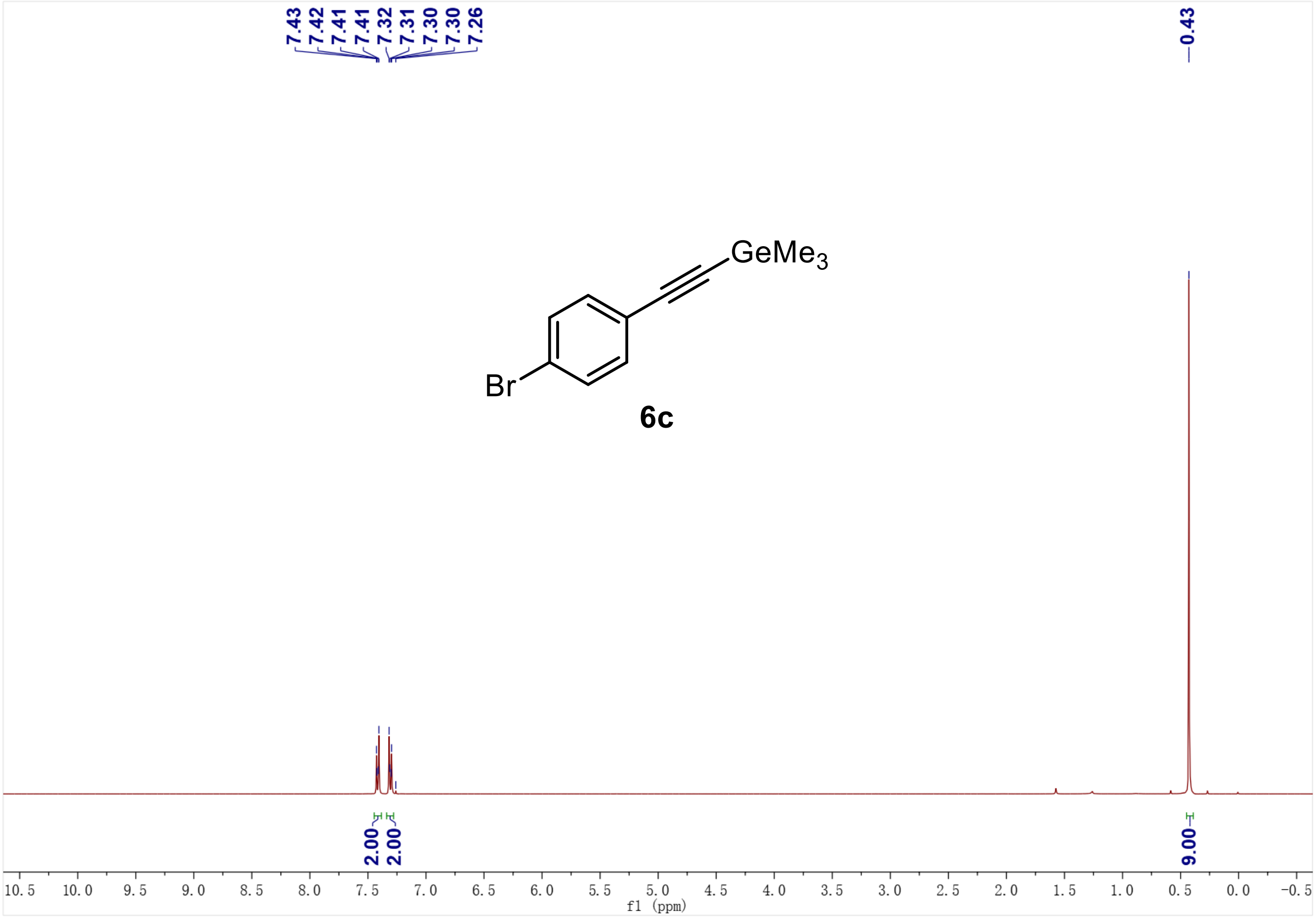

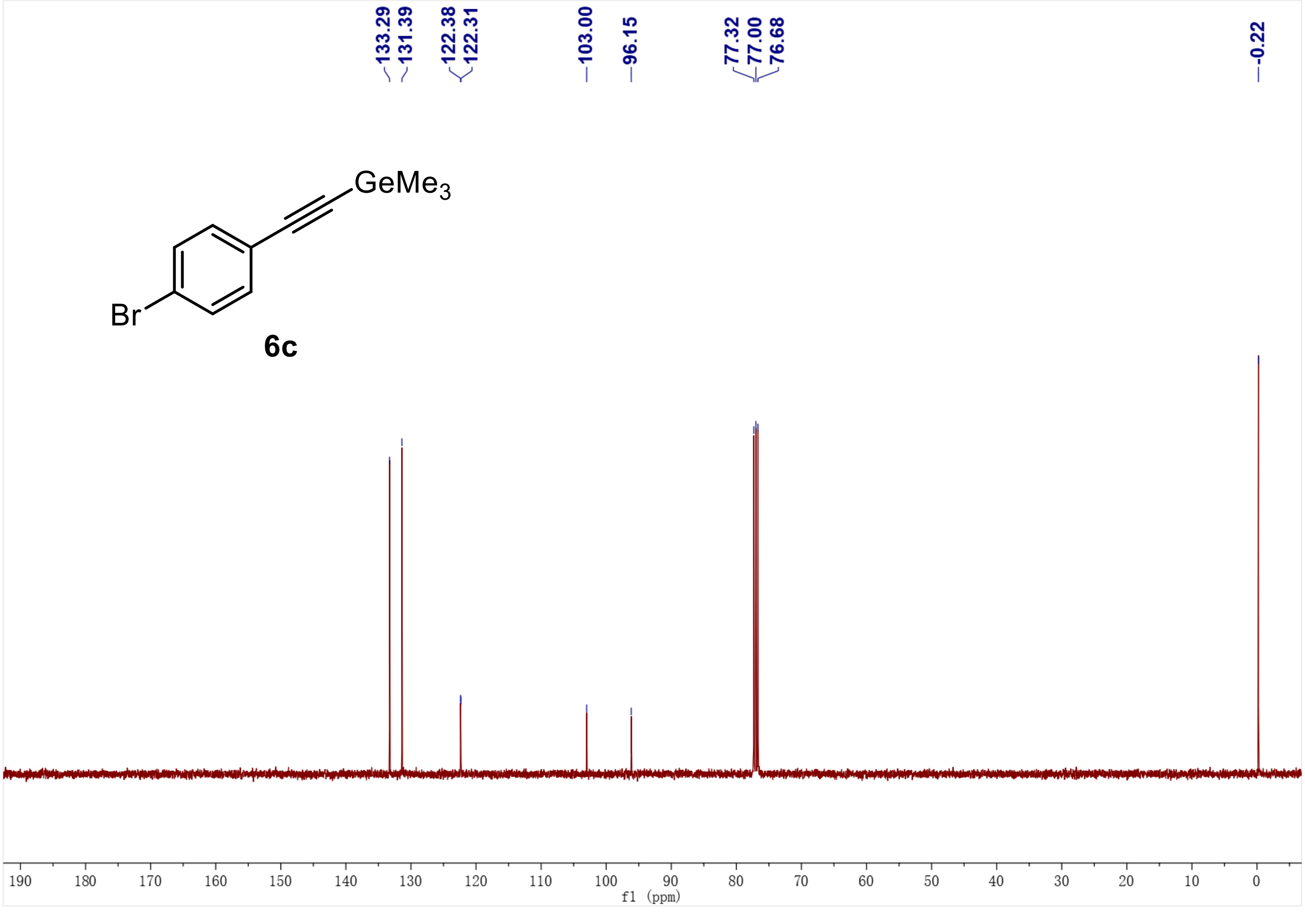

6d; ^{1}H NMR (400 MHz, $CDCl_3$); ^{13}C NMR (101 MHz, $CDCl_3$)

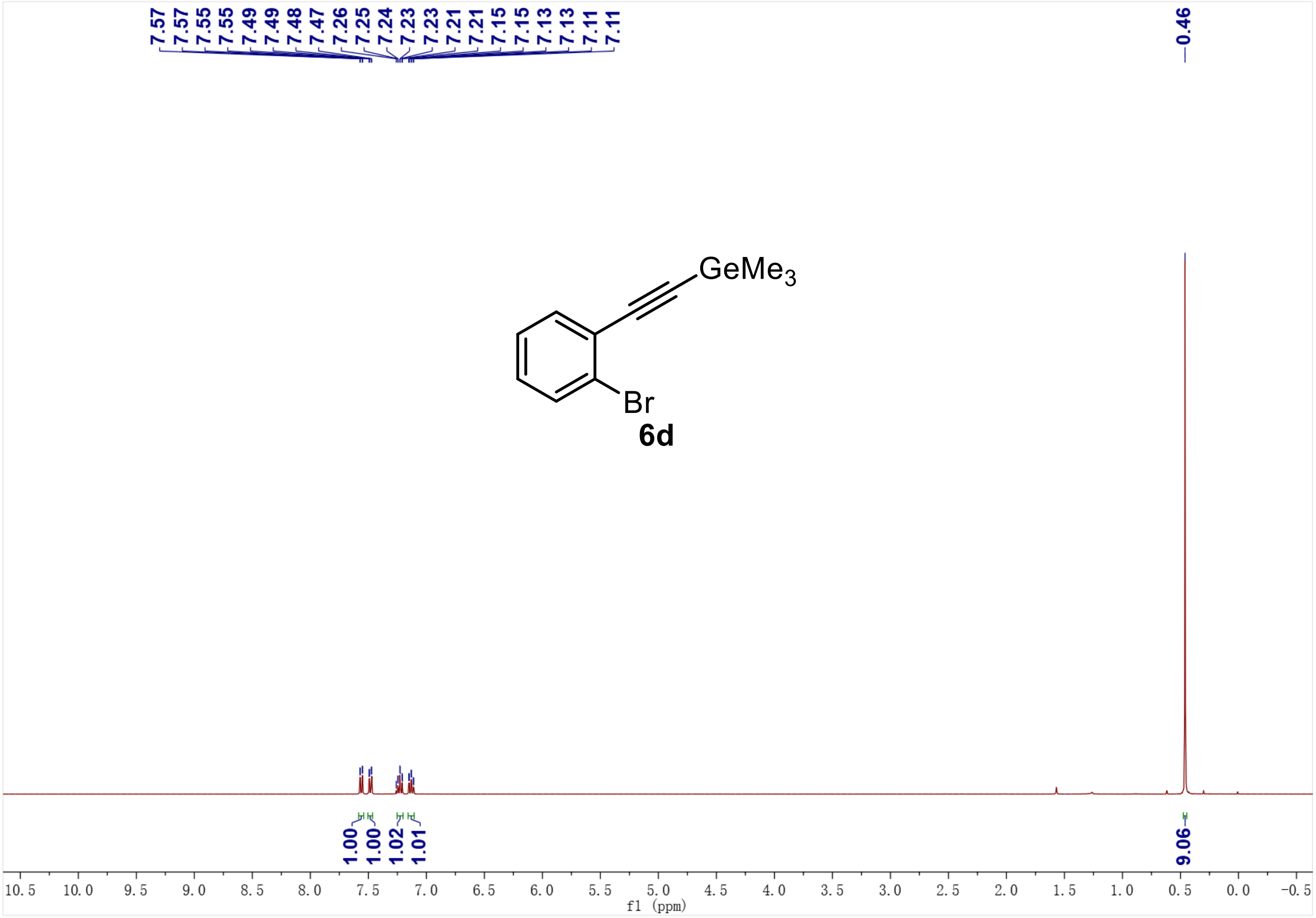

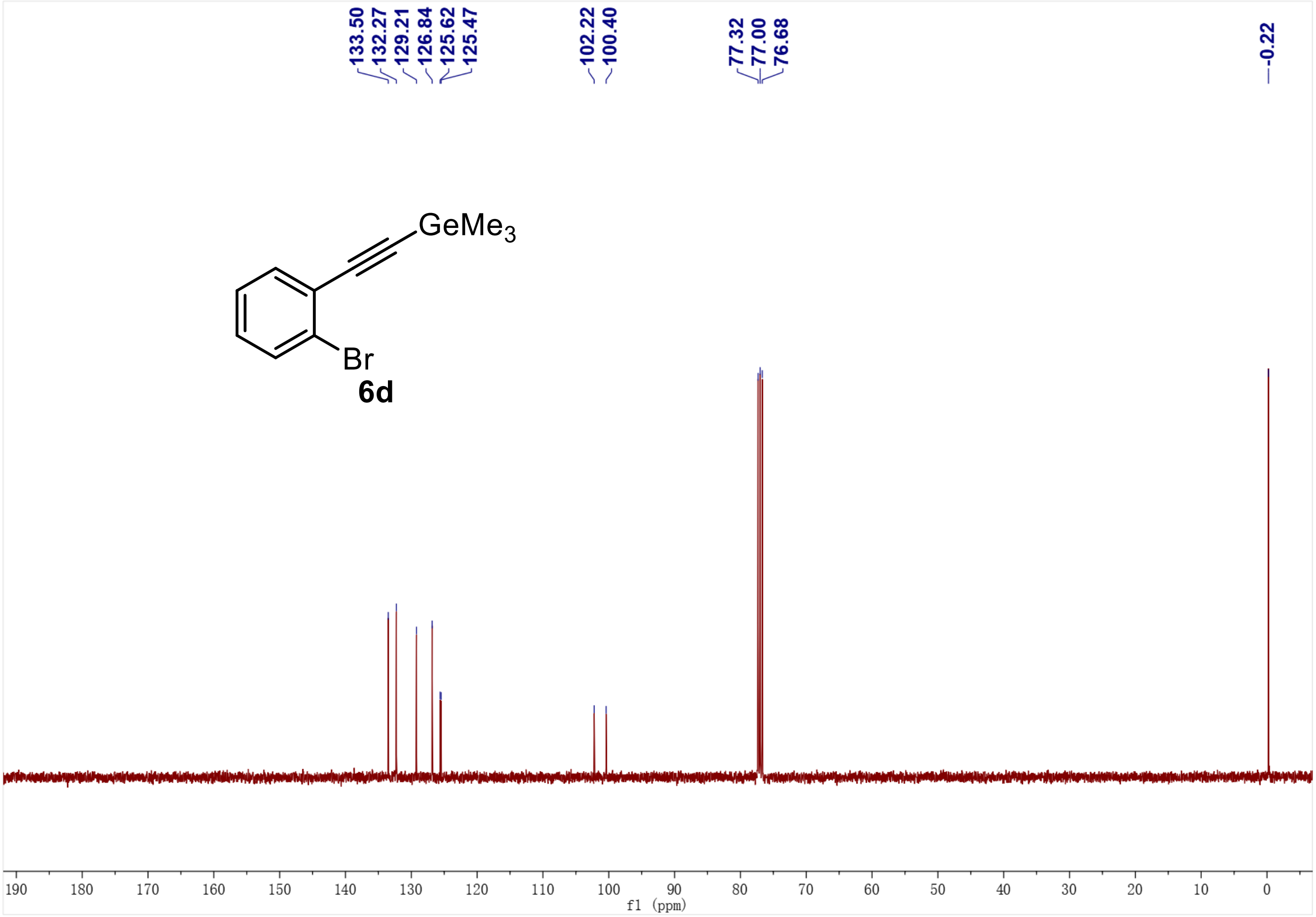

7. Reference

[1] a) S. Chang, D. Xing, Y. Zheng, L. Huang, *Org. Lett.* **2023**, *25*, 5350-5355; b) D. Xing, M. Feng, Y. Zheng, B. Huang, H. Jiang, L. Huang, *Org. Chem. Front.* **2023**, *10*, 3603-3611; c) Y. Cao, Y. Huang, P. R. Blakemore, *Eur. J. Org. Chem.* **2022**, e202200498; d) P. E. Simm, P. Sekar, J. Richardson, P. W. Davies, *ACS Catal.* **2021**, *11*, 6357-6362; e) K. Kanemoto, S. Yoshida, T. Hosoya, *Org. Lett.* **2019**, *21*, 3172-3177.

[2] J.-L. Tu, H. Gao, M. Luo, L. Zhao, C. Yang, L. Guo, W. Xia, *Green Chem.* **2022**, *24*, 5553-5558.

[3] W. Liu, Z. Chen, L. Li, H. Wang, C.-J. Li, *Chem. Eur. J.* **2016**, *22*, 5888-5893.

[4] H. Helbert, P. Visser, J. G. H. Hermens, J. Buter, B. L. Feringa, *Nat. Catal.* **2020**, *3*, 664-671.

[5] J. Oliver-Meseguer, I. Dominguez, R. Gavara, A. Leyva-Perez, A. Corma, *ChemCatChem.* **2017**, *9*, 1429-1435.

[6] I. Kownacki, B. Orwat, B. Marciniec, A. Kownacka, *Tetrahedron Lett.* **2014**, *55*, 548-550.

[7] A. A. Rajkiewicz, N. Wojciechowska, M. Kalek, *ACS Catal.* **2020**, *10*, 831-841.

[8] T. Kawatsu, S. Kataoka, N. Fukaya, J.-C. Choi, K. Sato, K. Matsumoto, *Acs Omega* **2021**, *6*, 12853-12857.

[9] S. Lv, F. Xu, Y. Fan, K. Ding, Z. Li, *J. Med. Chem.* **2023**, *66*, 2851-2864.

[10] P. Boehm, N. Kehl, B. Morandi, *Angew. Chem., Int. Ed.* **2023**, *62*, e202214071; *Angew. Chem.* **2023**, *135*, e202214071.

[11] C.-X. Xu, C.-H. Ma, F.-R. Xiao, H.-W. Chen, B. Dai, *Chin. Chem. Lett.* **2016**, *27*, 1683-1685.

[12] A. A. Toutov, K. N. Betz, D. P. Schuman, W.-B. Liu, A. Fedorov, B. M. Stoltz, R. H. Grubbs, *J. Am. Chem. Soc.* **2017**, *139*, 1668-1674.

[13] M. Wissing, A. Studer, *Chem. Eur. J.* **2019**, *25*, 5870-5874.

[14] V. Percec, J. G. Rudick, M. Peterca, M. Wagner, M. Obata, C. M. Mitchell, W. D. Cho, V. S. K. Balagurusamy, P. A. Heiney, *J. Am. Chem. Soc.* **2005**, *127*, 15257-15264.

[15] T. M. El Dine, R. Jimmidi, A. Diaconu, M. Fransolet, C. Michiels, J. De Winter, E. Gillon, A. Imberty, T. Coenye, S. P. Vincent, *J. Med. Chem.* **2021**, *64*, 14728-14744.

[16] S. Picard, E. J. Cueto-Diaz, E. Genin, G. Clermont, F. Acher, D. Ogden, M. Blanchard-Desce, *Chem. Commun.* **2013**, *49*, 10805-10807.

[17] B. Marciniec, B. Dudziec, I. Kownacki, *Angew. Chem., Int. Ed.* **2006**, *45*, 8180-8184.

[18] R. Rossi, A. Carpita, T. Messeri, *synth. Comm.,* **1991**, *21*, 1875-1888.

[19] H. Y. Cho, S. K. Woo, G. T. Hwang, *Molecules* **2012**, *17*, 12061-12071.

[20] G. Albano, F. Zinna, F. Urraci, M. A. M. Capozzi, G. Pescitelli, A. Punzi, L. Di Bari, G. M. Farinola, *Chem. Eur. J.* **2022**, *28*, e202201178.

[21] C. Koerner, P. Starkov, T. D. Sheppard, *J. Am. Chem. Soc.* **2010**, *132*, 5968-+.

[22] K. Nozawa-Kumada, M. Inagi, Y. Kondo, *Asian J. Org. Chem.* **2017**, *6*, 63-66.

[23] S. N. Greszler, H. A. Reichard, G. C. Micalizio, *J. Am. Chem. Soc.* **2012**, *134*, 2766-2774.

[24] T. Tsuchimoto, M. Fujii, Y. Iketani, M. Sekine, *Adv. Synth. Catal.* **2012**, *354*, 2959-2964.

[25] Q.-C. Gan, Z.-Q. Song, C.-H. Tung, L.-Z. Wu, *Org. Lett.* **2022**, *24*, 5192-5196.

[26] V. R. Chintareddy, K. Wadhwa, J. G. Verkade, *J. Org. Chem.* **2011**, *76*, 4482-4488.

[27] N. Sakai, N. Uchida, T. Konakahara, *Tetrahedron Lett.* **2008**, *49*, 3437-3440.

[28] N. Sakai, N. Uchida, T. Konakahara, *Synlett* **2008**, 1515-1519.

[29] M.-M. Xu, X.-Y. You, Y.-Z. Zhang, Y. Lu, K. Tan, L. Yang, Q. Cai, *J. Am. Chem. Soc.* **2021**, *143*, 8993-9001.

[30] N. Asao, K. Takahashi, S. Lee, T. Kasahara, Y. Yamamoto, *J. Am. Chem. Soc.* **2002**, *124*, 12650-12651.

[31] J. Yang, N. Yoshikai, *Angew. Chem., Int. Ed.* **2016**, *55*, 2870-2874; *Angew. Chem.* **2016**, *128*, 2920-2924.

[32] M. Ueda, T. Ueno, Y. Suyama, I. Ryu, *Tetrahedron Lett.* **2017**, *58*, 2972-2974.

[33] M. Ueda, T. Ueno, Y. Suyama, I. Ryu, *Chem. Commun.* **2016**, *52*, 13237-13240.

[34] T. Kawatsu, K. Aoyagi, Y. Nakajima, J.-C. Choi, K. Sato, K. Matsumoto, *Organometallics* **2020**, *39*, 2947-2950.

[35] D. Kim, K. Ham, S. Hong, *Org. Biomol. Chem.,* **2012**, *10*, 7305-7312.

[36] A. Krasovskiy, P. Knochel, *Synthesis* **2006**, 890-891.
